# Inverting the Regioselectivity of the Berberine Bridge Enzyme by Employing Customized Fluorine-Containing Substrates

**DOI:** 10.1002/chem.201201895

**Published:** 2012-09-07

**Authors:** Verena Resch, Horst Lechner, Joerg H Schrittwieser, Silvia Wallner, Karl Gruber, Peter Macheroux, Wolfgang Kroutil

**Affiliations:** [a] Department of Chemistry, Organic and Bioorganic Chemistry, University of GrazHeinrichstrasse 28, 8010 Graz (Austria), Fax: (+43) 316-3809840 E-mail: wolfgang.kroutil@uni-graz.at; [b] Institute of Biochemistry, Graz University of TechnologyPetersgasse 12, 8010 Graz (Austria); [c] Institute of Molecular Biosciences, University of GrazHumboldstrasse 50/III, 8010 Graz (Austria)

**Keywords:** alkaloids, biocatalysis, C–H activation, enzyme catalysis, regioselectivity

## Abstract

Fluorine is commonly applied in pharmaceuticals to block the degradation of bioactive compounds at a specific site of the molecule. Blocking of the reaction center of the enzyme-catalyzed ring closure of 1,2,3,4-tetrahydrobenzylisoquinolines by a fluoro moiety allowed redirecting the berberine bridge enzyme (BBE)-catalyzed transformation of these compounds to give the formation of an alternative regioisomeric product namely 11-hydroxy-functionalized tetrahydroprotoberberines instead of the commonly formed 9-hydroxy-functionalized products. Alternative strategies to change the regioselectivity of the enzyme, such as protein engineering, were not applicable in this special case due to missing substrate–enzyme interactions. Medium engineering, as another possible strategy, had clear influence on the regioselectivity of the reaction pathway, but did not lead to perfect selectivity. Thus, only substrate tuning by introducing a fluoro moiety at one potential reactive carbon center switched the reaction to the formation of exclusively one regioisomer with perfect enantioselectivity.

## Introduction

**Scheme 1:**
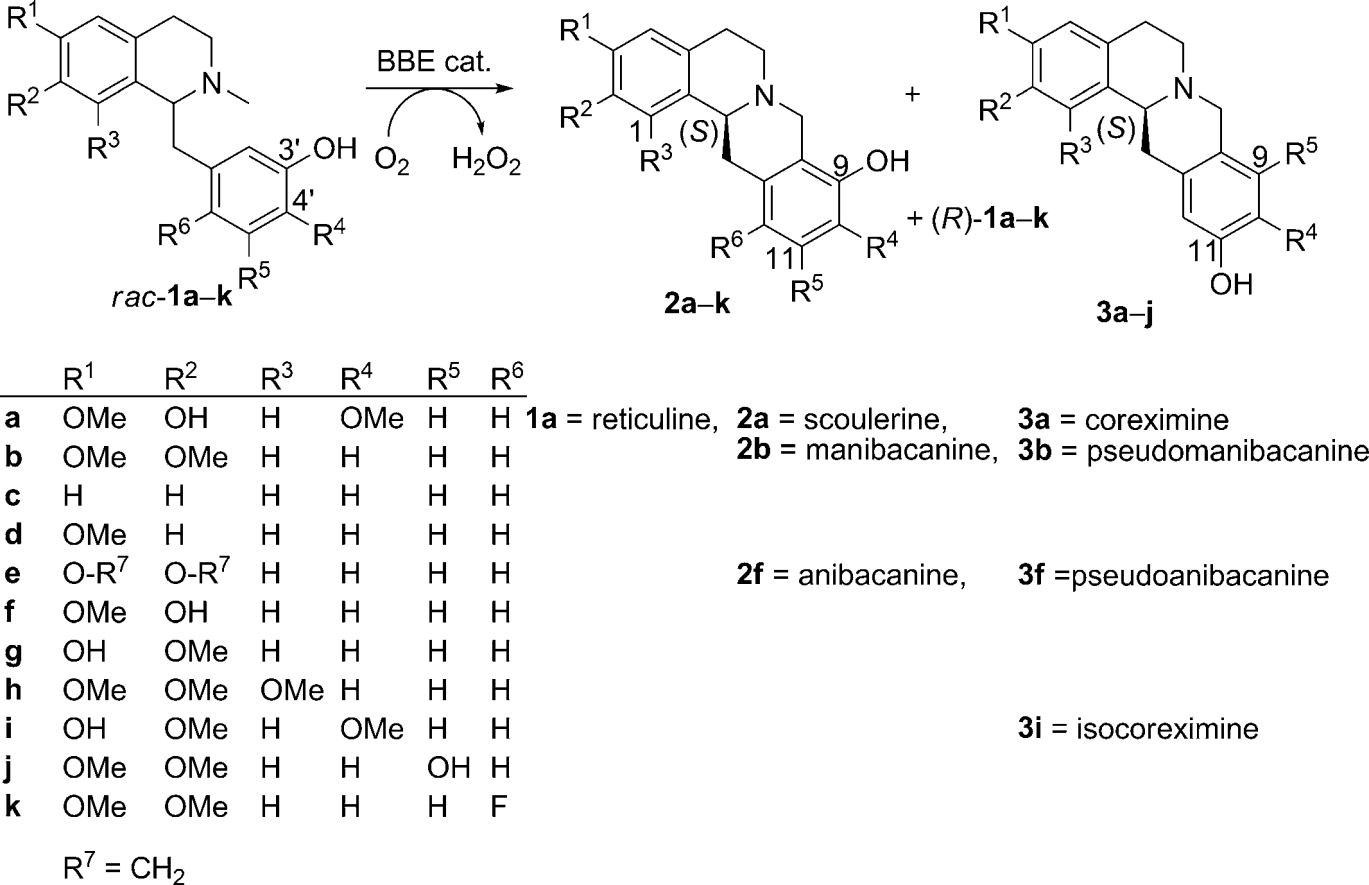
BBE-catalyzed enantioselective oxidative C–C formation at the expense of molecular oxygen.

Enzymes are regio- and enantioselective catalysts, which makes them more frequently applied complementary tools in organic synthesis.^1^, ^2^ Nevertheless, their excellent selective nature is sometimes considered as a limitation to their substrate and reaction spectrum. Consequently, several strategies were developed to widen their catalytic spectrum, preferentially by protein engineering,^3^ but also by medium engineering.^4^ The berberine bridge enzyme (BBE),^5^ a flavoenzyme naturally occurring, for example, in *Eschscholzia californica* (California poppy),^6^ catalyzes the regioselective oxidative C–C bond formation transforming the 1,2,3,4-tetrahydrobenzylisoquinoline (*S*)-reticuline **1 a** to (*S*)-scoulerine **2 a**, a 9-hydroxy functionalized tetrahydroprotoberberine derivative (Scheme 1).

## Results and Discussion

The wild-type enzyme does not give any trace of the corresponding 11-hydroxy-functionalized regioisomer (*S*)-coreximine **3 a**. However, a variant (E417Q) was described to promote the formation of (*S*)-coreximine **3 a** at 30 % of the total product formed,^7^ whereby in this case a 1500-fold reduced rate was observed compared with the natural reaction leading to (*S*)-scoulerine **2 a**. The amino acid Glu417 in the active site is responsible for the deprotonation of the 3′-hydroxyl group of (*S*)-reticuline **1 a** and, therefore, for the activation of the phenol moiety.

When various non-natural 1,2,3,4-tetrahydrobenzylisoquinolines were transformed by BBE catalysis, the formation of the corresponding regioisomers **3** was observed with the wild-type enzyme only as a minor side product (4–10 % of all substrate converted).^8^ Because various 11-hydroxy-functionalized tetrahydroprotoberberines (without an *O*-functionality in position 9) are described as natural products, such as (*S*)-coreximine,^9^ (*S*)-isocoreximine,^9^ (*S*)-corytenchine,^10^, ^11^ phellodendrine,^12^, ^13^ pseudoanibacanine,^14^ pseudomanibacanine,^10^ and many of them showed potential bioactivity,^9^–^11^, ^13^, ^15^ we searched for new asymmetric routes to access this type of product in optically pure form employing BBE.

**Figure 1:**
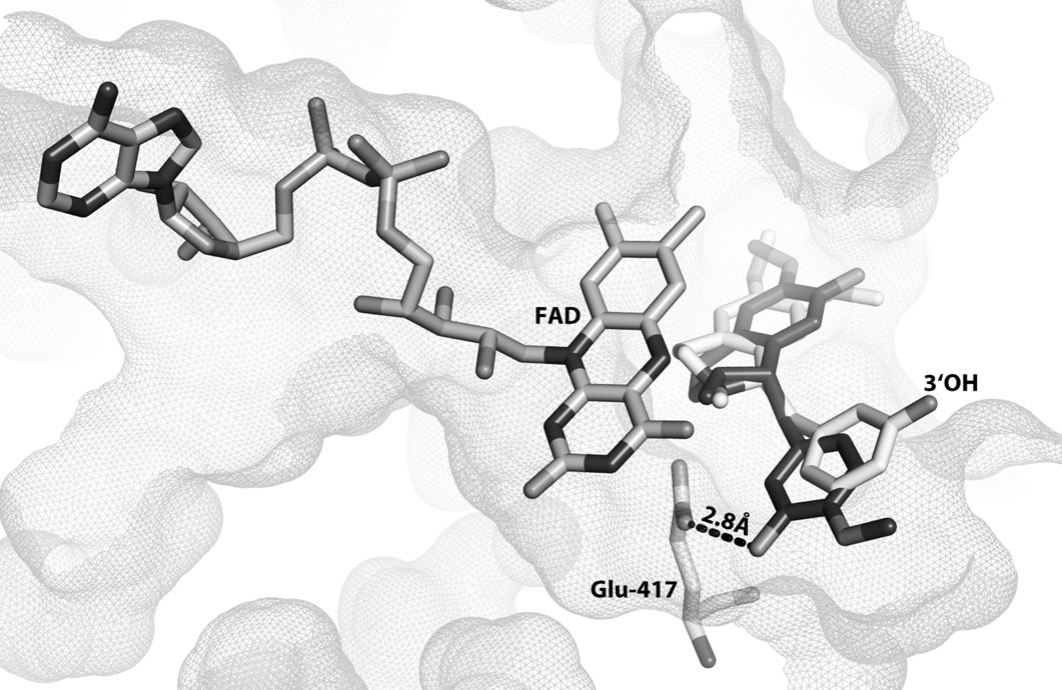
View of the active site. Overlay of the natural substrate **1 a** (black) in comparison with **1 b** (light gray). The catalytically essential residue Glu417, which is responsible for deprotonation of the 3′-phenolic OH, is shown including the distance [Å] to 3′-OH of **1 a**. The 3′-OH of **1 b** is clearly pointing away from the active site and is therefore accessible to deprotonation by the solvent.

**Figure 2:**
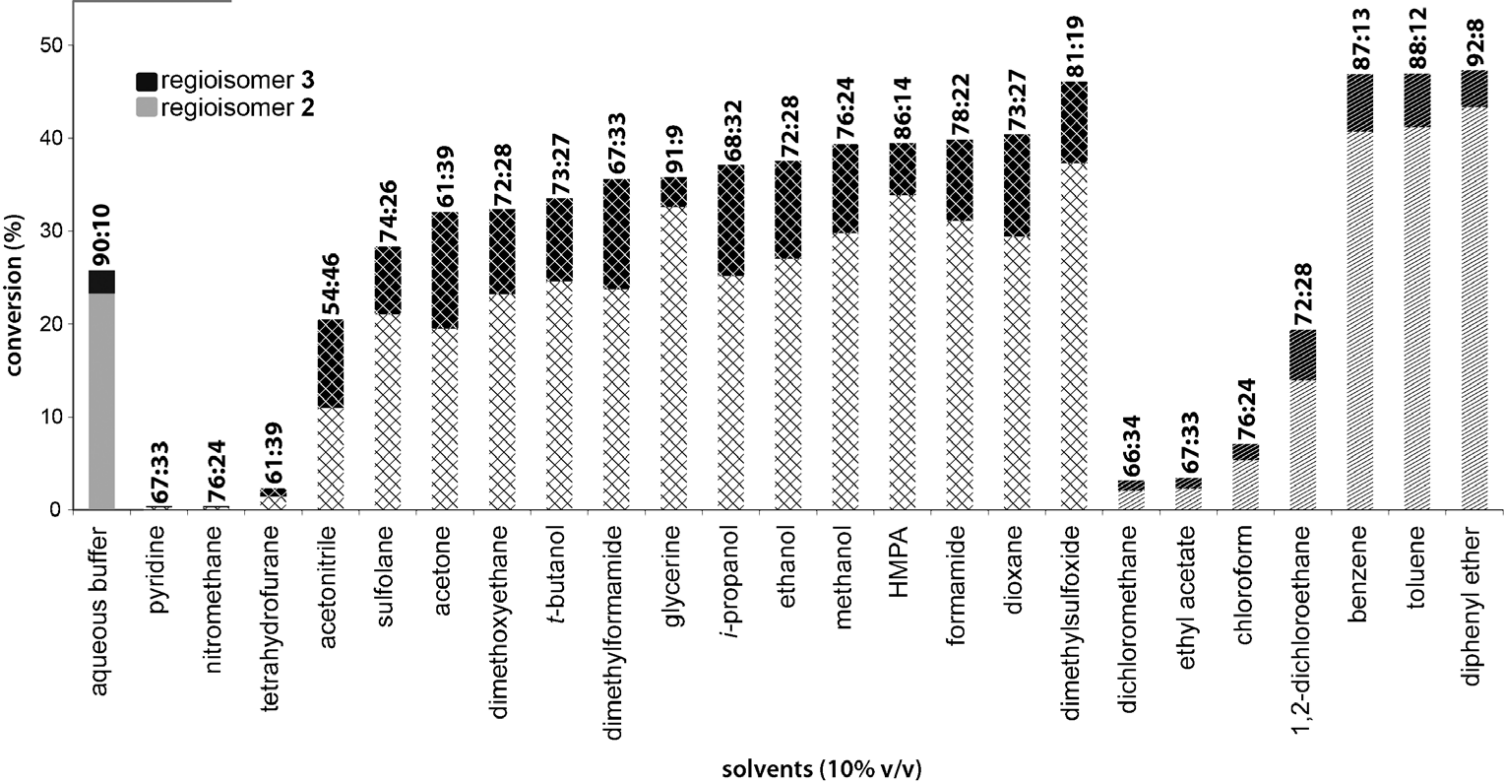
Effect of different co-solvents on regioisomer formation **2 b**/**3 b** in comparison with aqueous buffer (far left) for substrate *rac*-**1 b**. Solvents are divided into two subgroups: water miscible organic solvents (chequered) and water immiscible organic solvents (lined). Numbers above bars indicate the ratio between **2 b**/**3 b**. Reaction conditions: various solvents 10 % (v v^−1^). Reaction conditions: substrate **1 b** (2 g l^−1^, 6.5 mM), BBE (0.0017 mM), Tris-HCl (50 mM+MgCl_2_ 10 mM, pH 9), organic solvent (10 % v v^−1^), crude catalase (5 g l^−1^), 4 h, 40 °C.

The wild-type enzyme BBE transformed the racemic substrate *rac*-**1 b** to a 91:9 mixture of the regioisomers **2 b**/**3 b** in toluene/buffer 70:30 (pH 9). Testing the BBE variant E417Q with *rac*-**1 b** under the same conditions revealed that the **2 b**/**3 b** ratio did not change significantly. Docking studies confirmed previous assumptions6b that in the case of formation of regioisomer **3**, the 3′-hydroxyl moiety of the substrate **1** pointed out of the enzyme to the solvent and is not in the vicinity of any residue of the enzyme backbone (Figure 1). Thus, no amino acid of the enzyme backbone is close enough to promote deprotonation of the 3′-hydroxy moiety of benzylisoquinoline substrate rotamer, if exchanged to a residue acting as base. The base would be required to initialize the reaction, as described in the recent mechanistic studies. Consequently, enzyme engineering would not be successful to achieve selective formation of regioisomer **3** in the case of BBE. A possible alternative is “medium engineering”, first introduced by Wescott and Klibanov,^16^ which is—in contrast to protein engineering—a relatively fast method to alter the selectivity of an enzyme. Investigating various solvents at 10 % v v^−1^ at pH 9 with substrate *rac*-**1 b** and BBE showed that the formation of regioisomer **3 b** was influenced by the choice of the solvent (Figure 2).

**Figure 3:**
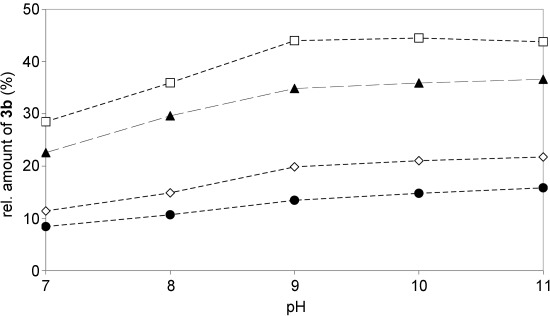
Formation of regioisomer **3 b** at varied pH values. The amount of **3 b** is plotted relative to the total amounts of products (**2 b**+**3 b**). Reaction conditions: substrate **1 a** (2 g l^−1^, 6.5 mM), BBE (0.0017 mM), Tris-HCl (50 mM+MgCl_2_ 10 mM, pH 6—13), co-solvents: DMSO (◊; 10 % v v^−1^), acetonitrile (□; 10 % v v^−1^), acetone (▴; 10 % v v^−1^), toluene (•; 70 % v v^−1^), crude catalase (5 g l^−1^), 4 h, 40 °C.

When acetonitrile was used as solvent, both regioisomers were formed almost in equal amounts (**2 b**/**3 b** 54:46), whereas in the case of acetone, 39 % of the transformed substrate gave regioisomer **3 b**. In contrast, the use of glycerin as co-solvent led to the lowest amount of **3 b** regioisomer formation (9 %) in the set of all co-solvents tested. In the subgroup of the water-immiscible solvents, 1,2-dichloroethane turned out to enhance the formation of the regioisomer **3 b** the most (28 %), whereas the use of diphenyl ether resulted in the formation of 8 % of regioisomer **3 b**. A next step would be the variation of the solvent content. This was not possible for all solvents, due to instability of BBE in the presence of some solvents (acetonitrile, THF). Nevertheless, in the case of toluene, the solvent concentration could be increased up to 99 % v v^−1^, however in this case, the formation of **3 b** decreased with increasing toluene concentration to a ratio of **2 b**/**3 b** 96:4 at 99 % toluene (see the Supporting Information, Figure S1). This seemed reasonable, because by decreasing the polarity of the solvent, the polar enzyme–substrate interactions became predominant, especially the interaction of the 3′-OH of the substrate **3 b** with the polar Glu417 residue, which is responsible for the formation of isomer **2 b**. Obviously, the solvents’ ability to deprotonate the 3′-OH of the substrate should enhance regioisomer formation **3 b**. Variation of the pH of the buffer in the presence of organic solvents confirmed that lowering the pH to 8 or 7 led to a decrease of the formation of regioisomer **3 b** (Figure 3); an increase of pH up to 11 led only to a minimal further increase of **3 b** formation compared with pH 9. Increasing the pH to 12 led to enzyme deactivation. The decrease of the formation of **3** with decreasing pH confirmed that the activation of the phenol by the 3′-moiety occurred by the solvent in the case of **3 b** regioisomer formation.

Although solvent engineering led to a significant increase of the formation of regioisomer **3 b**, in none of the cases was one single regioisomer formed exclusively, neither **2 b** nor **3 b**. Actually, **2 b** was in all cases the major component in the product mixture. Consequently, it was investigated whether the substitution pattern of the substrate had any influence on the ratio of the two isomers (Table 1).

**Table 1:** Ratios of regioisomer formation[a]

Entry	Substrate	Conversion[b] [%]	Ratio2/3
1	*rac*-**1 a**	50	>99:<1
2	*rac*-**1 b**	52	88:12
3	*rac*-**1 c**	46	86:14
4	*rac*-**1 d**	46	90:10
5	*rac*-**1 e**	51	88:12
6	*rac*-**1 f**	53	97:3
7	*rac*-**1 g**	48	70:30
8	*rac*-**1 h**	49	88:12
9	*rac*-**1 i**	53	98:2
10	*rac*-**1 j**	14	n.a.
11	*rac*-**1 k**	50	>99:<1
12	*rac*-**1 l**	<1	–
13	*rac*-**1 m**	<1	–
14	*rac*-**1 n**	48	<1:>99
15	*rac*-**1 o**	49	<1:>99
16	*rac*-**1 p**	49	<1:>99

[a] Reaction conditions: **1 a**–**k**: substrate (4 g l^−1^), BBE (0.0017 mM), Tris-HCl (50 mM+MgCl_2_ 10 mM, pH 9), DMSO (10 % v v^−1^), crude catalase (5 g l^−1^), 24 h, 40 °C; **1 l**–**o**: reaction conditions same as for **1 a**–**k**, except: BBE (0.017 mM); **1 p**: reaction conditions same as for **1 a**–**k**, except: BBE (0.034 mM).

[b] Conversion was measured by HPLC by using an achiral stationary phase. n.a.=not applicable, because regioisomers are identical.

First, it was expected that the missing methoxy group in substrate **1 b** in position 4′ of the phenolic part compared with the natural substrate **1 a** is the exclusive reason for formation of regioisomer **3 b** due to a missing interaction between substrate and enzyme. Indeed, the substrate with the natural substitution pattern at the isoquinoline part and the missing methoxy in 4′ (**1 f**) led also to very low, but measurable formation of regioisomer **3 f** with a ratio of **2 f**/**3 f** 97:3 (Table 1 entry 6). Other substrates without the methoxy in 4′ and varied substitution pattern at the isoquinoline moiety, such as without substituent (**1 c**) or one methoxy group (**1 d**), led to a similar ratio of regioisomers **2**/**3** as observed for **1 b** (Table 1, entries 2–5). Surprisingly, when the hydroxy and methoxy moiety at the isoquinoline part switched place (Table 1, substrate **1 g**, entry 7), 30 % of regioisomer **3 g** were formed. Thus, by modification of the isoquinoline part, the ratio was significantly changed. However, substrate **1 i** with the same substitution as the natural substrate, except that the hydroxy and the methoxy at the isoquinoline part switched place, was transformed with high preference to regioisomer **2 i** (**2 i**/**3 i** 98:2; Table 1, entry 9).

Substrate **1 j** is symmetrical with respect to the phenol moiety, thus in this case, no regioisomer differentiation can be made. Interestingly, compared with the substrates **1 a**–**i**, it was transformed rather slowly.

**Scheme 2:**
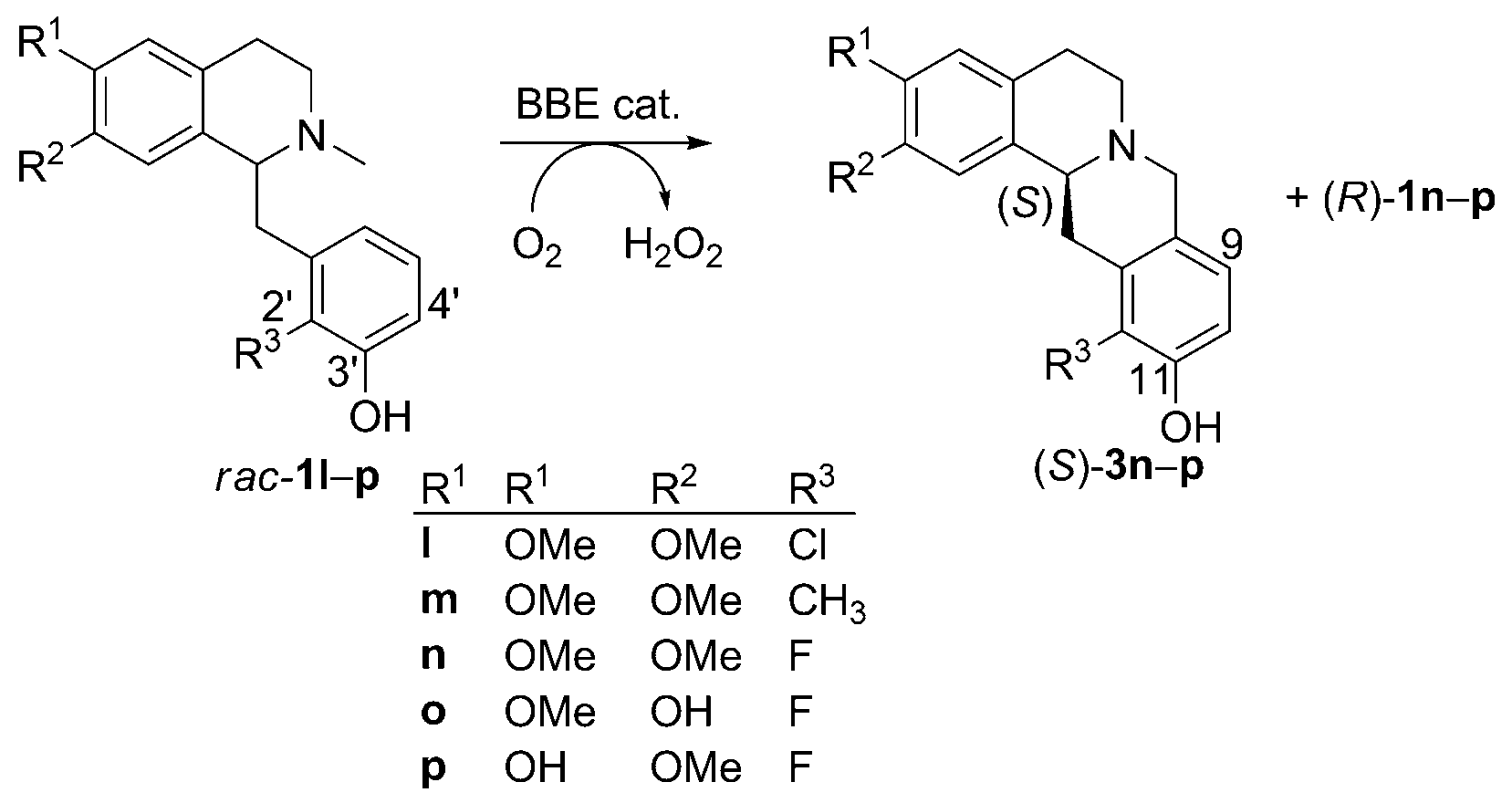
Regio- and enantioselective formation of 11-hydroxy-functionalized tetrahydroprotoberberines by oxidative C–C bond formation employing BBE by selective blocking of the 2′ position of the phenolic substrate.

**Figure 4:**
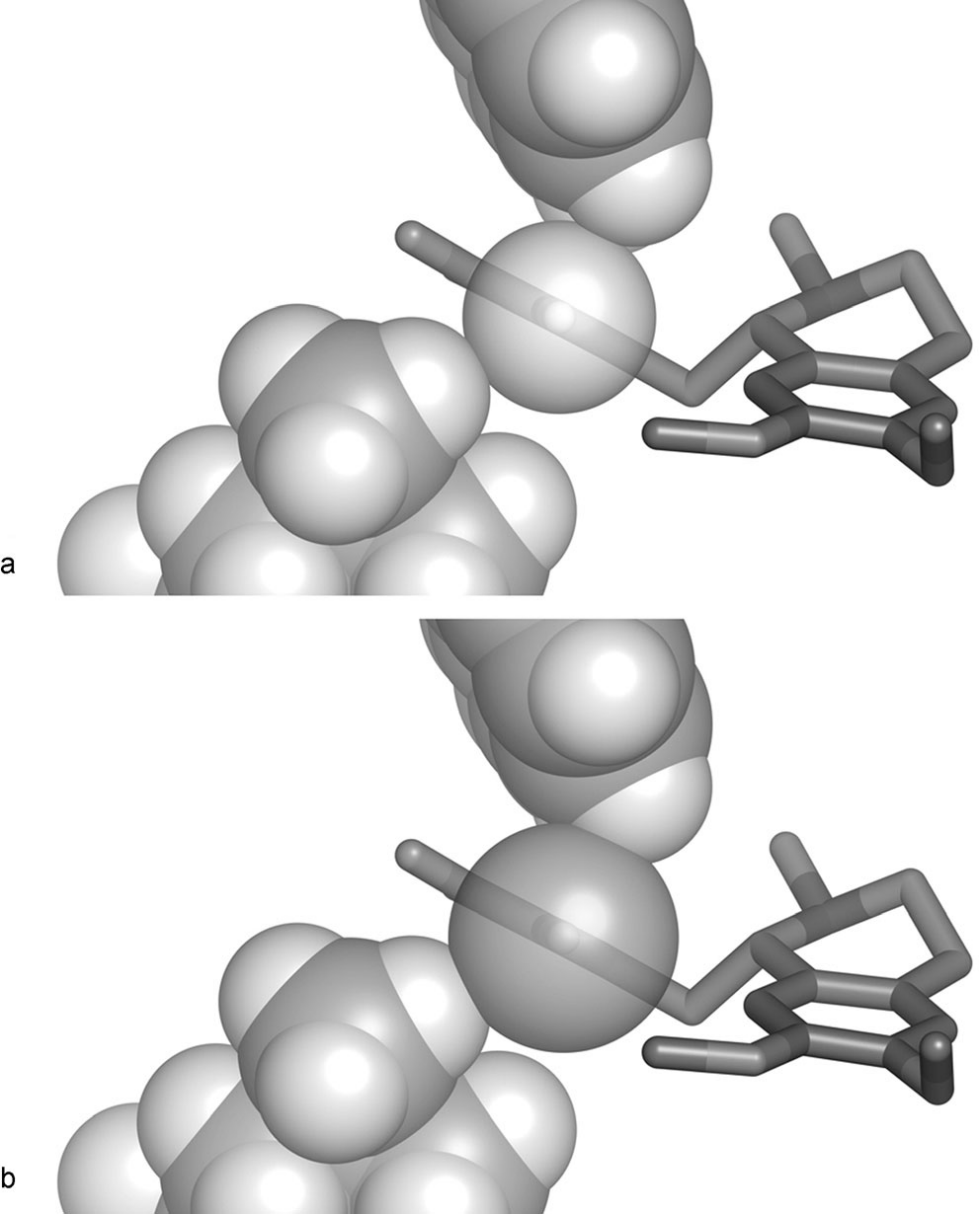
Size comparison of a) fluoro and b) chloro atoms shown as space-filling models. The two important residues that are limiting the diameter of the active site entrance are shown. In the case of the chloro atom, the collision with the residues is clearly visible. In the case of the fluoro atom, the van der Waals radius is small enough to make the substrate pass the active site entrance.

Obviously, the substitution pattern of the substrate influenced the ratio of the isomers; however, in no case perfect selectivity was observed. To force the formation of one single regioisomer, it was considered to block the position in which C–C formation should be excluded. Consequently, substrate **1 k** was constructed, in which a fluoro moiety blocks the 5′ position of the phenol. Because this substrate was accepted rather well leading exclusively to the formation of regioisomer **2 k**, the same concept was tested for the selective formation of regioisomers **3**: thus, the 2′ position of the substrate had to be blocked. Consequently, substrates **1 l**–**p** were prepared (Scheme 2). Blocking groups in 2′ position, such as chloro (substrate **1 l**) or methyl (**1 m**), were not accepted. It is likely that these substituents are too bulky for a productive orientation of the substrate in the active site of the enzyme (Figure 4). Arguably, the fluoro moiety might be better suited, because fluoro is smallest possible group to substitute hydrogen.

Thus, when fluoro was used as blocking group, substrate **1 n** was transformed exclusively to the regioisomer **3 n**. It is worth to note that this transformation occurred with perfect enantioselectivity, thus exclusively the (*S*)-enantiomer (*S*)-**1 n** was converted to (*S*)-12-fluoropseudomanibacanine (*S*)-**3 n** (enantiomeric excess (*ee*) >97 %, HPLC), leaving the (*R*)-enantiomer (*R*)-**1 n** untouched behind (Table 2).

**Table 2:** Preparative regio- and enantioselective transformation of 100 mg (*rac*-1 k, *rac*-1 n, and *rac*-1 o) and 20 mg substrates (*rac*-1 p), respectively

	Recovered (*R*)-**1**		Isolated **2** and **3**		
Substrate	Yield [mg]/[%]	*ee*[a] [%]	Sole product	Yield [mg]/[%]	*ee*[a] [%]
*rac*-**1 k**	47/47	>97	(*S*)-**2 k**	49/49	>97
*rac*-**1 n**	49/49	>97	(*S*)-**3 n**	43/43	>97
*rac*-**1 o**	42/42	>97	(*S*)-**3 o**	32/32	>97
*rac*-**1 p**	10/50	>97	(*S*)-**3 p**	8.4/42	>97

[a] The optical purity was established by HPLC on a chiral stationary phase.

To demonstrate the preparative applicability of this approach, all fluoro substrates were successfully transformed in an enantioselective kinetic resolution by BBE on a 100 mg (*rac*-**1 k**, *rac*-**1 n**, **1 o**) or 20 mg scale (*rac*-**1 p**) giving up to 49 % isolated yield, or up to 98 % isolated yield with respect to the maximal obtainable yield of the fluoro products (Table 2). All products were obtained in optically pure form.

## Conclusion

To promote the formation of 11-hydroxy-functionalized tetrahydroprotoberberines instead of the commonly formed 9-hydroxy-functionalized products from 1,2,3,4-tetrahydroisoquinolines catalyzed by BBE, a customized substrate strategy was successfully applied. This is an example, in which protein engineering was not applicable, and medium engineering showed only a moderate influence; only by blocking the common reaction site of the substrate by a fluoro substituent allowed to redirect the reaction pathway to the alternative product. In addition, introducing fluoro substituents is a common strategy to avoid the degradation of pharmaceuticals.^17^ Herein, this strategy was applied to access an alternative product pattern.

## Experimental Section

**General**: ^1^H and ^13^C NMR spectra were recorded by using a Bruker 300 MHz instrument. Chemical shifts are given in parts per million (ppm) relative to TMS (*δ*=0 ppm), and coupling constants (*J*) are reported in Hz. Melting points were determined in open capillary tubes and are uncorrected. TLC was carried out on silica-gel 60 F_254_ plates, and compounds were visualized either by spraying with Mo reagent [(NH_4_)_6_Mo_7_O_24_**⋅**4H_2_O (100 g L^−1^), Ce(SO_4_)_2_**⋅**4H_2_O (4 g L^−1^) in H_2_SO_4_ (10 %)] or by UV. Unit resolution GC-MS analyses were performed by using electron impact (EI) ionization at 70 eV and quadrupole mass selection. High-resolution MS analyses were performed by using EI ionization at 70 eV and TOF mass selection. Optical rotation values [*α*]_D_^20^ were measured at *λ*=589 nm (Na line) by using a cuvette of 1 dm path length.

Unless otherwise noted, reagents and organic solvents were obtained from commercially available sources and used without further purification. Toluene, methanol, and acetonitrile used for anhydrous reactions were dried over appropriate molecular sieves (4 Å for toluene, 3 Å for MeOH and MeCN) for at least 48 h. THF used for anhydrous reactions was distilled from potassium/benzophenone directly before use. For anhydrous reactions, flasks were oven dried and flushed with dry argon just before use. Standard syringe techniques were applied to transfer dry solvents and reagents in an inert atmosphere of dry argon.

Catalase from bovine liver was purchased from Sigma–Aldrich (Lot.: 81H7146).

**Determination of conversion**: Conversions were determined by HPLC on an achiral C18 stationary phase. Eluent: buffer (30 mM HCOONH_4_, pH 2.8)/methanol/acetonitrile 67:18:15 (isocratic); flow rate: 0.5 mL min^−1^; column temperature: 20 °C; detection wavelength: 280 nm. Retention time is given in the Supporting Information.

**Representative synthesis of 1 n**

*2,2,2-Trichloro-1-(2-fluoro-3-methoxyphenyl)ethanol:* 2-Fluoro-3-methoxybenzaldehyde (5.0 g, 32.4 mmol) was converted to 2,2,2-trichloro-1-(2-fluoro-3-methoxyphenyl)ethanol by using chloroform (6 mL), DMF (20 mL), and KOH (1.5 g, 26.7 mmol) in methanol (5 mL). 2-Fluoro-3-methoxybenzaldehyde was dissolved in DMF and chloroform, and the solution was cooled to about −10 °C by using a salt–ice bath. KOH in methanol was added dropwise under an argon atmosphere with cooling. The reaction was was continued for an additional 2 h before quenching the mixture, still under cooling, to pH 1 with concentrated aqueous HCl solution. After stirring for additional 30 min at −10 °C, the reaction was allowed to warm to RT, and phase separation was performed. The aqueous phase was extracted with toluene (30 mL). The combined organic layers were washed with water (30 mL) and brine (30 mL) and the solvent was removed under reduced pressure. No further purification steps were necessary. Yield: 8.3 g (93 %). *R*_f_ (hexanes/ethyl acetate (+1 % of acetic acid) 3:1)=0.23; ^1^H NMR (300 MHz, CDCl_3_): *δ*=7.36–7.28 (1 H, m, Ar), 7.06 (1 H, t, *J*=8.1 Hz, Ar), 6.93 (1 H, t, *J*=8.0 Hz, Ar), 5.59 (1 H, s, Ar-C*H*), 3.83 ppm (3 H, s, O-C*H_3_*); ^13^C NMR (75 MHz, CDCl_3_): *δ*=152.0, 150.3 (d, *J*_CF_=248.1 Hz), 147.1 (d, *J*_CF_=11.3 Hz), 125.1 (d, *J*_CF_=9.8 Hz), 123.4 (d, *J*_CF_=4.6 Hz), 120.8 (d, *J*_CF_=1.7 Hz), 113.6 (d, *J*_CF_=1.9 Hz), 102.9, 56.2 ppm.

*2-(2-Fluoro-3-methoxyphenyl)acetic acid*: For the synthesis of 2-(2-fluoro-3-methoxyphenyl)acetic acid, oxygen-free ethanol was needed; therefore, ethanol (60 mL) was purged with argon for 60 min. After purging, diphenyl diselenide (9.9 g, 31.8 mmol) was added and solubilized followed by the addition of NaBH_4_ (2.4 g, 63.5 mmol) in portions under constant argon flow. After completed addition, the previously orange solution turned colorless and was stirred for 30 min at RT. After 30 min, the substrate **2** (8.3 g, 30.2 mmol) was added and dissolved followed by the addition of NaOH (7.3 g, 181.4 mmol). The reaction was heated to 40 °C and left overnight. After all starting material was consumed, as was shown by TLC, the solvent was removed under reduced pressure. The remaining solid was dissolved in water (30 mL) and extracted under basic conditions with ethyl acetate (30 mL). After extraction, the aqueous phase was adjusted to pH 1 under cooling with concentrated aqueous HCl solution. Then extraction with ethyl acetate (5×30 mL) was performed, and the combined organic layers were dried over Na_2_SO_4_. Ethyl acetate was removed under reduced pressure, and the product was purified by silica-gel column chromatography by using a gradient of hexanes (to remove the nonreacted diphenyl diselenide and apolar by-products) and hexanes/acetone 1:1 to elute the product. Yield: 2.8 g (50 %). M.p.: 106–108 °C; *R*_f_ (hexanes/ethyl acetate (one drop of acetic acid) 3:1)=0.21; ^1^H NMR (300 MHz, CDCl_3_): *δ*=6.89–7.01 (3 H, m, Ar), 3.87 (3 H, s, O-CH_3_), 3.69 ppm (2 H, s, CH_2_-COOH); ^13^C NMR (75 MHz, CDCl_3_): *δ*=170.9, 150.8 (d, *J*_CF_=244.6 Hz), 147.8 (d, *J*_CF_=10.8 Hz), 123.8 (d, *J*_CF_=4.8 Hz), 123.0 (d, *J*_CF_=13.4 Hz), 122.7 (d, *J*_CF_=3.1 Hz), 112.3 (d, *J*_CF_=1.8 Hz), 55.5 (d, *J*_CF_=2.7 Hz), 33.6 ppm (d, *J*_CF_=3.8 Hz).

*2-(2-Fluoro-3-hydroxyphenyl)acetic acid*: 2-(2-Fluoro-3-methoxyphenyl)acetic acid (2.8 g, 15.2 mmol) was dissolved in bromic acid (48 % in water, 30 mL) and heated to 105 °C by using an oil bath. After a reaction time of 5 h, the reaction was allowed to cool to RT and extracted with ethyl acetate (3×30 mL). The combined organic layers were dried over Na_2_SO_4_ and concentrated under reduced pressure. No further purification was necessary. Yield: 2.2 g (86 %). M.p.: 110–112 °C; *R*_f_ (hexanes/ethyl acetate (+1 drop of acetic acid) 3:1)=0.13; ^1^H NMR (300 MHz, CDCl_3_): *δ*=6.70–6.95 (3 H, m, Ar), 3.64 (2 H, d, *J*_HF_=1.5 Hz, C*H*_2_-COOH); ^13^C NMR (75 MHz, CDCl_3_): *δ*=173.2, 150.2 (d, *J*_CF_=240.3 Hz), 144.8 (d, *J*_CF_=13.2 Hz), 123.6 (d, *J*_CF_=4.5 Hz), 122.7 (d, *J*_CF_=13.7 Hz), 121.2 (d, *J*_CF_=2.7 Hz), 116.3 (d, *J*_CF_=2.8 Hz), 33.7 ppm (d, *J*_CF_=3.8 Hz).

*2-(3-(Benzyloxy)-2-fluorophenyl)acetic acid*: The benzylation of 2-(2-fluoro-3-hydroxyphenyl)acetic acid was performed by using 2.2 g (12.9 mmol) of substrate. The substrate was dissolved in ethanol (50 mL), than KOH (1.7 g, 30.8 mmol), benzyl bromide (2.6 g, 16.5 mmol), and NaI (0.1 g, 0.4 mmol) were added. This reaction mixture was allowed to react at RT overnight. After control by TLC, the mixture was filtrated through pad of Celite, and ethanol was removed under reduced pressure. The residue was redissolved in water (20 mL) and acidified with acetic acid. The aqueous phase was extracted with ethyl acetate (3×30 mL), and the combined organic layers were dried over Na_2_SO_4_ followed by the removal of the solvents under reduced pressure. Purification was performed by using silica-gel column chromatography with hexane/ethyl acetate 3:1 as eluent. Yield after purification: 2.8 g (83 %). M.p.: 95–96 °C; *R*_f_ (hexane/ethyl acetate (+1 % acetic acid) 3:1)=0.25; ^1^H NMR (300 MHz, CDCl_3_): *δ*=7.29–7.46 (5 H, m, Ar), 6.84–7.08 (3 H, m, Ar), 5.14 (2 H, s, *CH_2_*-O), 3.66 ppm (2 H, d, *J*_HF_=1.6 Hz, C*H*_2_-COOH); ^13^C NMR (75 MHz, CDCl_3_): *δ*=173.0, 151.3 (d, *J*_CF_=245.2 Hz), 146.7 (d, *J*_CF_=11.0 Hz), 136.9, 128.1, 127.6, 127.2, 123.4 (d, *J*_CF_=4.8 Hz), 122.9 (d, *J*_CF_=3.1 Hz), 114.3 (d, *J*_CF_=1.7 Hz), 70.8, 33.6 ppm (d, *J*_CF_=3.9 Hz).

*2-(3-(Benzyloxy)-2-fluorophenyl)acetyl chloride*: For the synthesis of 2-(3-(benzyloxy)-2-fluorophenyl)acetyl chloride, 2-(3-(benzyloxy)-2-fluorophenyl)acetic acid (0.91 g, 3.3 mmol) was dissolved in try toluene (20 mL). To this mixture, oxalyl chloride (0.7 g, 5.4 mmol) and one drop of DMF were added. The reaction was allowed to proceed for 3 h, than toluene and remaining oxalyl chloride were removed under reduced pressure. The product, 2-(3-(benzyloxy)-2-fluorophenyl)acetyl chloride, was directly used for the next step without further purification.

*2-(3-(Benzyloxy)-2-fluorophenyl)-N-(3,4-dimethoxyphenethyl)-N-methylacetamide*: 2-(3-(Benzyloxy)-2-fluorophenyl)-*N*-(3,4-dimethoxyphenethyl)-*N*-methylacetamide was synthesized by the use of 2-(3-(benzyloxy)-2-fluorophenyl)acetyl chloride (0.95 g, 3.3 mmol) and 2-(3,4-dimethoxyphenyl)-*N*-methylethanamine (0.7 g, 3.3 mmol). 2-(3,4-Dimethoxyphenyl)-*N*-methylethanamine was dissolved in chloroform (10 mL), and NaOH aqueous solution (3 %, 10 mL) was added. This mixture was cooled in an ice bath, and 2-(3-(benzyloxy)-2-fluorophenyl)acetyl chloride dissolved in chloroform (10 mL) was added dropwise under cooling. After complete addition of 2-(3-(benzyloxy)-2-fluorophenyl)acetyl chloride, the ice bath was removed, and the reaction was allowed to proceed at RT for 16 h. For work-up, the phases were separated, and the aqueous phase was extracted with chloroform (3×10 mL). The combined organic layers were washed with aqueous HCl solution (1 M, 10 mL) and dried over Na_2_SO_4_. Chloroform was removed under reduced pressure, and the crude product (1.2 g) was purified by silica-gel chromatography (hexanes/ethyl acetate 1:1). Yield: 1.0 g (70 %). M.p.: 104–106 °C; *R*_f_ (hexanes/ethyl acetate=1:1)=0.22; NMR spectroscopy revealed that the product is a mixture of isomers (*trans*/*cis* 1.09:1). Based on the peak intensities as well as the distortionless enhancement by polarization transfer (DEPT), COSY, and heteronuclear single-quantum coherence (HSQC) spectra, the NMR signals were assigned to the isomers as follows: ^1^H NMR (300 MHz, CDCl_3_): *trans: δ*=7.39–7.24 (m, 6 H, Ar), 6.91–6.59 (m, 6 H, Ar), 5.05 (s, 2 H, Ar-CH_2_-O), 3.78 (s, 3 H, O-CH_3_), 3.77 (s, 3 H, O-CH_3_), 3.62 (s, 2 H, CO-C*H_2_*-Ar), 3.52 (t, 2 H, *J*=7.5 Hz, Ar-CH_2_-C*H_2_*-N), 2.86 (s, 3 H, N-CH_3_), 2.72 ppm (t, 2 H, *J*=7.5 Hz, Ar-C*H_2_*-CH_2_-N); *cis: δ*=7.39–7.24 (m, 5 H, Ar), 6.91–6.59 (m, 7 H, Ar), 5.02 (s, 2 H, Ar-CH_2_-O), 3.77 (s, 3 H, O-CH_3_), 3.76 (s, 3 H, O-CH_3_), 3.46–3.41 (m, 4 H, Ar-CH_2_-C*H_2_*-N, CO-C*H_2_*-Ar), 2.91 (s, 3 H, N-CH_3_), 2.66 ppm (t, 2 H, *J*=7.3 Hz, Ar-C*H_2_*-CH_2_-N); ^13^C NMR (75 MHz, CDCl_3_): *trans: δ*=169.8. 150.8 (d, *J*_CF_=245.1 Hz), 148.9, 147.5, 146.7, 136.6, 131.6, 128.6, 128.1, 127.4, 123.9 (d, *J*_CF_=3.0 Hz), 123.6 (d, *J*_CF_=8.0 Hz), 122.6 (d, *J*_CF_=3.0 Hz), 120.7, 114.0 (d, *J*_CF_=1.7 Hz), 112.0, 111.2, 71.3, 55.9, 50.3, 36.4, 33.7, 33.2 ppm. *cis: δ*=170.1, 150.6 (d, *J*_CF_=244.1 Hz), 149.1, 147.9, 146.8, 136.5, 130.5, 128.6, 128.1, 127.4, 123.8 (d, *J*_CF_=3.0 Hz), 123.4 (d, *J*_CF_=8.2 Hz), 122.5 (d, *J*_CF_=2.9 Hz), 120.8, 113.9 (d, *J*_CF_=1.7 Hz), 111.8, 111.4, 71.3, 55.9, 52.1, 34.4, 33.8, 32.9 ppm.

*1-(3-(Benzyloxy)-2-fluorobenzyl)-6,7-dimethoxy-2-methyl-1,2,3,4-tetrahydroisoquinoline*: For the cyclization of 2-(3-(benzyloxy)-2-fluorophenyl)-*N*-(3,4-dimethoxyphenethyl)-*N*-methylacetamide, the following procedure was used. By using dried glassware, 2-(3-(benzyloxy)-2-fluorophenyl)-*N*-(3,4-dimethoxyphenethyl)-*N*-methylacetamide (1.0 g, 2.3 mmol) was dissolved in dry acetonitrile (25 mL), and phosphoryl chloride (1.1 g, 7.2 mmol) was added under constant argon flow. The reaction was heated at reflux for 3 h, after which the solvent was removed under reduced pressure. The resulting residue was used for the next step without further purification. In the next step, the residue was dissolved in dry methanol (25 mL), and the solution was cooled in an ice bath. Under cooling and a constant flow of argon, NaBH_4_ (0.82 g, 22.2 mmol) was added in portions. After completion of addition of NaBH_4_, the ice bath was removed, and the reaction was allowed to proceed at RT for 16 h. The methanol was removed under reduced pressure. The residue was redissolved in half-saturated Na_2_CO_3_ aqueous solution (10 mL) and extracted with dichloromethane (3×10 mL). The combined organic layers were dried over Na_2_SO_4_, and dichloromethane was removed under reduced pressure. Purification was performed by using silica-gel chromatography (DCM/MeOH/NH_3_OH 98:1:1). Yield: 0.82 g (86 %). *R*_f_ (DCM/MeOH/NH_3_OH 90:9:1): 0.35; ^1^H NMR (300 MHz, CDCl_3_): *δ*=7.46–7.31 (m, 5 H, Ar), 6.92–6.84 (m, 2 H, Ar), 6.65–6.56 (m, 2 H, Ar), 6.56 (s, 1 H, Ar), 6.07 (s, 1 H, Ar), 5.12 (s, 2 H, O-C*H_2_*-Ar), 3.83 (s, 3 H, O-C*H_3_*), 3.81–3.76 (m, 1 H, N-C*H*), 3.54 (s, 3 H, O-C*H_3_*), 3.26–3.17 (m, 2 H, N-CH-C*H_2_*), 2.90–2.76 (m, 3 H, C*H_2_*-C*H_2_*), 2.64–2.56 (m, 1 H, C*H_2_*-C*H_2_*), 2.52 ppm (s, 3 H, N-C*H_3_*); ^13^C NMR (75 MHz, CDCl_3_): *δ*=151.7 (d, *J*_CF_=244.3), 147.3, 146.6 (d, *J*_CF_=11.3), 146.4, 136.7, 129.3, 128.6, 128.4 (d, *J*_CF_=13.1), 128.1, 127.4, 125.9, 124.1 (d, *J*_CF_=4.1), 123.2 (d, *J*_CF_=4.7), 113.4 (d, *J*_CF_=1.4), 111.2, 110.8, 71.3, 63.2, 55.4, 46.4, 42.6, 34.4, 30.9, 25.4 ppm.

**Representative biocatalytic transformation**: Substrate **1 b** (100 mg, 0.3 mmol, final concentration: 2 g L^−1^=6.5 mM) was dissolved in DMSO (5 mL) and buffer (45 mL, Tris-HCl, 10 mM, pH 9, 10 mM MgCl_2_) containing BBE (429 μL enzyme solution, final concentration: 0.3 g L^−1^=0.0049 mM) and catalase (125 mg crude preparation). The mixture was shaken in a light-shielded round bottom flask (50 mL) in an Incubator mini shaker (VWR, rotary, orbit 3 mm) at 200 rpm and 40 °C for 24 h. The reaction was stopped by phase separation followed by extraction of the aqueous phase employing ethyl acetate (3×10 mL). Combined organic phases were dried over Na_2_SO_4_, and the organic solvents were removed under reduced pressure. The crude product was purified by using silica-gel chromatography (silica gel 60, 0.040–0.063 mm, Merck, Lot.: 1.09385.9025) giving 49 mg of (*R*)-**1 b** (50 % yield, >97 % *ee*) and 43 mg (*S*)-**3 b** (43 % yield, >97 % *ee*). (*R*)-**1 b**: M.p.: 149–151 °C; optical rotation: [*α*]_D_^20^=−59.5; *c*=1.25 (g 100 mL^−1^) in chloroform; HPLC: column: chiralcel O*J*; eluent: *n-*heptane/2-propanol 80:20+0.1 % TFA (isocratic); 0.5 mL min^−1^; column temperature: 40 °C; detection wavelength: 280 nm. Retention time: 25.5 min; *R*_f_ (DCM/MeOH/NH_3_ (aqueous) 90:9:1)=0.34; ^1^H NMR (300 MHz, CDCl_3_): *δ*=6.79–6.74 (m, 1 H, Ar), 6.70–6.64 (m, 1 H, Ar), 6.50 (s, 1 H, Ar), 6.48–6.43 (m, 1 H, Ar), 5.94 (s, 1 H, Ar), 3.80–3.78 (m, 1 H, *N-*CH), 3.76 (s, 3 H, O-CH_3_), 3.47 (s, 3 H, O-CH_3_), 3.28–3.12 (m, 2 H, CH_2_), 2.90–2.71 (m, 3 H, CH_2_), 2.60–2051 (m, 1 H, CH_2_), 2.46 ppm (s, 3 H, *N-*CH_3_). ^13^C NMR (75 MHz, CDCl_3_): *δ*=150.4 (d, *J*_CF_=237.0), 147.5, 146.4, 144.4 (d, *J*_CF_=14.5), 128.5, 127.1 (d, *J*_CF_=13.0), 125.1, 123.8 (d, *J*_CF_=4.2), (d, *J*_CF_=3.5), 122.4, 115.9 (d, *J*_CF_=3.8), 111.2, 110.9, 63.1, 55.7, 55.4, 45.6, 41.9, 34.4, 24.5 ppm; HRMS calcd for C_19_H_22_FNO_3_: 330.1505 [*M*^+^−H]; found: 330.1524; (*S*)-**3 b**: M.p.: 207–209 °C; optical rotation: [*α*]_D_^20^=−205.3; *c*=0.62 (g 100 mL^−1^) in chloroform; HPLC: column: chiralcel OD-H; eluent: *n-*heptane/2-propanol 70:30+0.1 % TFA (isocratic); 0.50 mL min^−1^; column temperature: 18 °C; detection wavelength: 280 nm; retention time: 30.5 min; *R*_f_ (DCM/MeOH/NH_3_ (aqueous) 90:9:1)=0.51; ^1^H NMR (300 MHz, CDCl_3_): *δ*=6.72–6.63 (m, 3 H, Ar), 6.55 (s, 1 H, Ar), 3.89 (d, 1 H, *J*=14.7 Hz, *N-*CH_2_-Ar), 3.84 (s, 3 H, O-CH_3_), 3.80 (s, 3 H, O-CH_3_), 3.57 (d, 1 H, *J*=15.8 Hz, *N-*CH_2_-Ar), 3.53–3.49 (m, 1 H, CH_2_), 3.33 (dd, 1 H, *J*_1_=16.8 Hz, *J*_2_=4.2 Hz, CH_2_), 3.13–3.02 (m, 2 H, CH_2_), 2.69–2.51 ppm (m, 2 H, CH_2_); ^13^C NMR (75 MHz, CDCl_3_): *δ*=149.1 (d, *J*_CF_=235.3), 147.6, 147.5, 141.5 (d, *J*_CF_=14.2), 129.1, 127.3 (d, *J*_CF_=3.9), 126.6, 122.4 (d, *J*_CF_=16.0), 121.6 (d, *J*_CF_=3.8), 115.2 (d, *J*_CF_=1.6), 111.3, 108.5, 58.9, 57.8, 56.2, 55.8, 51.4, 30.0 ppm; HRMS calcd for C_19_H_20_FNO_3_: 329.1427; found: 329.1452 [*M*^+^−H].

## Supplementary material

Detailed facts of importance to specialist readers are published as ”Supporting Information”. Such documents are peer-reviewed, but not copy-edited or typeset. They are made available as submitted by the authors.
