# Supplementary material for: Inverting the Regioselectivity of the Berberine Bridge Enzyme by Employing Customized Fluorine-Containing Substrates
Source: Chemistry. 2012 Sep 7;18(41):13173–9. doi: 10.1002/chem.201201895 (PMC3533790; doi:10.1002/chem.201201895)
Supplement: Supplementary file 1 [file chem0018-13173-SD1.pdf]

CHEMISTRY
A EUROPEAN JOURNAL

Supporting Information

© Copyright Wiley-VCH Verlag GmbH & Co. KGaA, 69451 Weinheim, 2012

Inverting the Regioselectivity of the Berberine Bridge Enzyme by Employing Customized Fluorine-Containing Substrates

Verena Resch,[a] Horst Lechner,[a] Joerg H. Schrittwieser,[a] Silvia Wallner,[b] Karl Gruber,[c] Peter Macheroux,[b] and Wolfgang Kroutil*[a]

chem_201201895_sm_miscellaneous_information.pdf

Content

1. General Methods 2
2. Regioisomer formation 2
3. Analytics 3
4. Synthesis 5
4.1. Synthesis of **1g** 5
4.2. Synthesis of **1i** 9
4.3. Synthesis of **1j** 12
4.4. Synthesis of **1k** 15
4.5. Synthesis of **1l** 19
4.6. Synthesis of **1m** 22
4.7. Synthesis of **1n** 26
4.8. Synthesis of **1o** 29
4.9. Synthesis of **1p** 31
5. Biocatalytic preparative transformations: 34
6. Spectra 38
7. Literature 200

1. General Methods

^{1}H and ^{13}C-NMR spectra were recorded using a 300 MHz instrument. Chemical shifts are given in parts per million (ppm) relative to TMS (δ = 0 ppm) and coupling constants (*J*) are reported in Hertz (Hz). Melting points were determined in open capillary tubes and are uncorrected. Thin layer chromatography was carried out on silica gel 60 F_{254} plates and compounds were visualized either by spraying with Mo reagent [$(NH_4)_6Mo_7O_{24} \cdot 4H_2O$ (100 g/L), $Ce(SO_4)_2 \cdot 4H_2O$ (4 g/L) in H_2SO_4 (10%)] or by UV. Unit resolution GC-MS analyses were performed using electron impact (EI) ionisation at 70 eV and quadrupole mass selection. High resolution MS analyses were performed using electron impact (EI) ionisation at 70 eV and TOF mass selection. Optical rotation values $[\alpha]_D^{20}$ were measured at 589 nm (Na line) using a cuvette of 1 dm path length.
Unless otherwise noted, reagents and organic solvents were obtained from commercially available sources and used without further purification. Toluene, methanol and acetonitrile used for anhydrous reactions were dried over appropriate molecular sieves (4Å for toluene, 3Å for MeOH and MeCN) for at least 48 hours. THF used for anhydrous reactions was distilled from potassium/benzophenone directly before use. For anhydrous reactions, flasks were oven-dried and flushed with dry argon just before use. Standard syringe techniques were applied to transfer dry solvents and reagents in an inert atmosphere of dry argon.
Catalase from bovine liver was purchased from *Sigma-Aldrich* (Lot.: 81H7146).

Docking: Molecular models of the substrates were built and optimized using the program Maestro V.9.2 (Schrodinger Inc.) and were docked into the active site of two BBE structures (pdb-codes: 3D2D and 3FW9) using the program Yasara v11.6.16 (http://www.yasara.org). Before docking the bound ligand as well as the water molecules were removed from the protein structures. Default parameters (as defined in the dock_run macro) were used in the docking calculations except for the number of docking runs, which was set to 50, and the ligand RMSD for clustering, which was set to 5 Å. The resulting structures were evaluated concerning their binding energy and their mechanistic feasibility, whereby non-reactive states were excluded. Afterwards, an energy minimization of the modelled complex within a water box was performed using YASRARA. All structures were analyzed using the program PyMOL 1.1val (http://www.pymol.org).

2. Regioisomer formation

The formation of regioisomer **3b** was investigated in varied concentration of toluene at pH 9.0 (Figure 1).

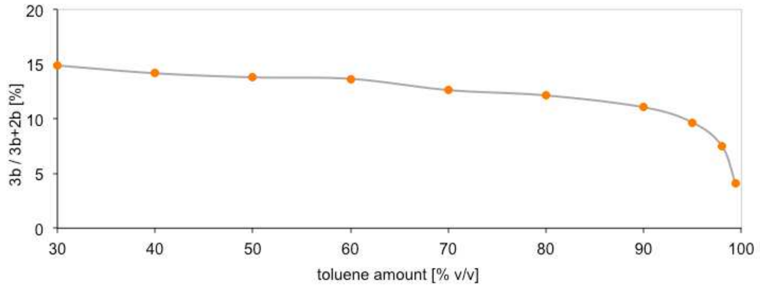

Figure S1: Effect of varied amounts of toluene on the formation of regioisomer **3b**. Reaction conditions: Toluene from 30% to 100% (v v^{-1}), 4 g L^{-1} substrate **1b** (13 mM), BBE (0.0017 mM), Tris-HCl 50 mM + $MgCl_2$ 10 mM, pH 9, 5 g L^{-1} crude catalase, 4 h, 40 °C, shaking at 300 rpm.

3. ANALYTICS

Determination of conversion: Conversions were determined by HPLC on an achiral C18 stationary phase. Eluent: buffer (30 mM $HCOONH_4$, pH 2.8)/methanol/acetonitrile = 67/18/15 (isocratic); flow rate: 0.5 mL/min; column temperature: 20 °C; detection wavelength: 280 nm. Retention times (min):

Substrate no.	retention time [min]		
	regioisomer **3**	substrate **1**	main product **2**
**1a**	-	10.2	15.4
**1b**	11.5	18.1	20.9
**1c**	13.8	21.8	27.3
**1d**	9.8	12.8	15.7
**1e**	12.2	18.0	22.4
**1f**	7.5	9.0	10.0
**1g**	7.5	8.9	10.7
**1h**	10.1	16.4	19.0
**1i**	9.1	10.6	15.9
**1j**	7.1	7.7	-
**1k**	7.1	9.9	-
**1l**	-	7.4	-
**1m**	10.5	12.0	-
**1n**	-	7.9	10.1
**1o**	10.9	14.5	-
**1p**	-	12.5	-

Table 1: Conversions were calculated from peak areas considering the different ε of the substrate and product.

Determination of enantiomeric excess for substrates:

Methods:

1. column: Chiracel OJ; Eluent: *n*-heptane/2-propanol = 80/20 + 0.1% TFA (isocratic); 0.50 mL/min; column temperature: 40 °C; detection wavelength: 280 nm
2. column: Chiracel OJ; Eluent: *n*-heptane/2-propanol = 70/30 + 0.1% TFA (isocratic); 0.35 mL/min; column temperature: 18 °C; detection wavelength: 280 nm
3. column: Chiracel OJ; Eluent: *n*-heptane/2-propanol = 70/30 + 0.1% TFA (isocratic); 0.50 mL/min; column temperature: 18 °C; detection wavelength: 280 nm
4. column: Chiracel OJ; Eluent: *n*-heptane/2-propanol = 70/30 + 0.1% TFA (isocratic); 0.20 mL/min; column temperature: 18 °C; detection wavelength: 280 nm

Substrate no.	retention time [min]		
	(*S*)-enantiomer	(*R*)-enantiomer	method no.
**1a**	29.5	32.8	3
**1b**	21.9	25.7	1
**1c**	31.7	34.6	1
**1d**	17.3	24.5	1
**1e**	53.3	57.9	4
**1f**	32.9	40.0	2
**1g**	22.0	29.0	2
**1h**	15.7	19.4	3
**1i**	28.6	32.6	3
**1j**	24.2	31.0	2
**1k**	25.8	34.8	3
**1n**	20.5	31.4	2
**1o**	17.7	21.4	1
**1m**	21.9	25.1	1

Table 2: Retention times of (*S*)- and (*R*)-enantiomers of differed substrates including method used.

Determination of enantiomeric excess for products obtained on preparative scale:

A mixture of the berbine **2n**–**2p** and **2k** (10 mg, 29.3–35.5 µmol), 2,4-dimethyl-3-pentanol (2.5 µL, 17.8 µ mol) and Shvo' s complex [1-hydroxytetraphenylcyclopentadienyl(tetraphenyl-2,4-cyclopentadien-1-one)-µ-hydro-tetracarbonyldiruthenium(II); 4.3 mg, 4.0 µmol] in dry toluene (0.5 mL) was stirred at 110 °C under argon for 24 h in a closed reaction vessel (glass vial). The reaction mixture was then loaded directly onto a silica gel column and the product was purified by gradient elution with CH_2Cl_2 → CH_2Cl_2 : MeOH : NH_3(aq) = 96:3:1. Purity and identity of the product were verified by GC-MS and the enantiomeric excess was determined by HPLC on a chiral stationary phase, indicating that all isolated compounds were racemic.

Methods:

1. column: Chiralcel OD-H; Eluent: *n*-heptane/2-propanol = 70/30 + 0.1% TFA (isocratic); 0.50 mL/min; column temperature: 18 °C; detection wavelength: 280 nm

No.	retention time [min]		
	(*S*)-enantiomer	(*R*)-enantiomer	method no.
**3k**	33.9	25.6	1
**2n**	30.2	53.1	1
**2o**	28.0	50.1	1
**2p**	-[1]	-[1]	1

Table 3: [1]The racemisation method was for this product not applicable. Due to decomposition of the compound under these reaction conditions, no racemic reference material was available for **2p**.

4. SYNTHESIS

The synthesis of compounds **1a**–**1f, 1h** was described previously.[1,2]

4.1. Synthesis of 1g:

1-(3-hydroxybenzyl)-7-methoxy-2-methyl-1,2,3,4-tetrahydroisoquinolin-6-ol

3-Benzyloxy-4-methoxybenzaldehyde[3]

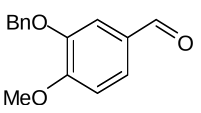

K_2CO_3 (20.1 g, 0.145 mol) and benzyl bromide (22.5 g, 0.131 mol) were added to a stirred solution of isovanillin (20.0 g, 0.131 mol) in ethanol (120 mL). The mixture was stirred for 20 h at room temperature under argon atmosphere. The solution was filtered through Celite, washed with CH_2Cl_2 (3 × 100 mL) and the solvent was evaporated under reduced pressure. The residue was taken up in CH_2Cl_2 (200 mL), washed with 5% aqu. NaOH solution (100 mL) and dried over K_2CO_3. Evaporation of the solvent under reduced pressure yielded 31.3 g of a yellow solid. Recrystallization from ethanol gave 3-benzyloxy-4-methoxybenzaldehyde (29.2 g, 91%) as a white solid.

mp: 62–63 °C

TLC (hexanes : ethyl acetate = 3:1): R_f = 0.29.

^{1}H-NMR (300 MHz, $CDCl_3$) [ppm]: 9.84 (1H, s, CHO), 7.47–7.50 (4H, m, Ar), 7.33–7.42 (3H, m, Ar), 7.01 (1H, d, *J* = 8.7 Hz, Ar), 5.21 (2H, s, $PhCH_2O$), 3.98 (3H, s, OCH_3)

^{13}C-NMR (75 MHz, $CDCl_3$) [ppm]: 190.8, 155.1, 148.7, 136.3, 130.0, 128.6, 128.1, 127.5, 126.9, 111.4, 110.8, 70.9, 56.2.

MS (EI, 70 eV): *m/z* = 242 (M^+, 13), 91 (100), 65 (9).

The NMR data are in accordance with literature.[4]

3-Benzyloxy-4-methoxy-*β*-nitrostyrene[5]

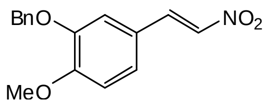

A solution of 3-benzyloxy-4-methoxy-benzaldehyde (27.9 g, 0.115 mol), nitromethane (22.3 g, 0.366 mol) and NH_4OAc (22.5 g, 0.292 mol) in AcOH (300 mL) was refluxed for 4 h. The mixture was poured onto ice water, resulting in the formation of a yellow precipitate. CH_2Cl_2 was added for dissolving the solid, the phases were separated and the aqueous phase was extracted with CH_2Cl_2 (3 × 50 mL). The combined organic phases were washed with water (100 mL), half-saturated aqu. Na_2CO_3 solution (50 mL) and brine (50 mL), dried over Na_2SO_4 and evaporated under reduced pressure to give a brownish solid. Recrystallization from EtOH afforded 21.95 g (67%) of 3-benzyloxy-4-methoxy-*β*-nitrostyrene as a yellow solid.
mp: 128–130°C

TLC (hexanes : ethyl acetate = 1:1): R_f = 0.77.

^{1}H-NMR (300 MHz, $CDCl_3$) [ppm]: 7.92 (1H, d, *J* = 13.5 Hz, C*H*=CH-NO_2), 7.33–7.48 (6H, m, Ar), 7.19 (1H, d, *J* = 8.4 Hz, Ar), 7.06 (1H, s, Ar), 6.95 (1H, d, *J* = 8.1 Hz, Ar), 5.19 (2H, s, $PhCH_2O$), 3.96 (3H, s, OCH_3).

^{13}C-NMR (75 MHz, $CDCl_3$) [ppm]: 153.5, 148.6, 139.3, 136.3, 135.1, 128.8, 128.2, 127.3, 124.9, 122.7, 113.2, 111.8, 71.2, 56.1.

MS (EI, 70 eV): *m/z* = 285 (M^+, 11), 91 (100).

The NMR data are in accordance with literature.[6]

3-Benzyloxy-4-methoxyphenethylamine

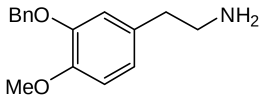

A solution of 3-benzyloxy-4-methoxy-*β*-nitrostyrene (21.3 g, 74.7 mmol) in dry THF (200 mL) was added dropwise to a suspension of $LiAlH_4$ (14.2 g, 374 mmol) in dry THF (100 mL) under argon atmosphere over 1 h. The reaction mixture was refluxed for 16 h, then diluted with THF (100 mL) and cooled to 0 °C on an ice bath. Water (14 mL), 15% aqu. NaOH solution (14 mL) and again water (42 mL) were added to the vigorously stirred mixture, the ice bath was removed and stirring continued for 1 h at room temperature. The resulting suspension was filtered through Celite, washed with THF, dried over Na_2SO_4 and evaporated under reduced pressure to give 17.7 g (92%) of 3-benzyloxy-4-methoxyphenethylamine as a brownish liquid.

TLC (CH_2Cl_2 : MeOH : NH_3(aq) = 90:9:1): R_f = 0.38.

^{1}H-NMR (300 MHz, $CDCl_3$) [ppm]: 7.31–7.47 (5H, m, Ar), 6.75–6.87 (3H, m, Ar), 5.16 (2H, s, $PhCH_2O$), 3.88 (3H, s, OCH_3), 2.88 (2H, t, J = 6.8 Hz, CH_2-CH_2-NH_2), 2.64 (2H, t, J = 6.8 Hz, CH_2-CH_2-NH_2), 1.01 (2H, br s, NH_2).

^{13}C-NMR (75 MHz, $CDCl_3$) [ppm]: 148.2, 148.0, 137.2, 132.4, 128.5, 127.8, 127.4, 121.5, 115.1, 112.0, 71.1, 56.1, 43.6, 39.5.

MS (EI, 70 eV): m/z = 257 (M^+, 7), 228 (45), 137 (25), 91 (100).

Ethyl 3-benzyloxy-4-methoxyphenethylcarbamate

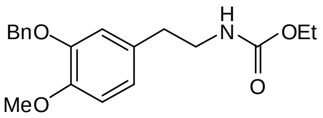

Triethylamine (7.08 g, 70.0 mmol) and ethyl chloroformate (8.19 g, 75.5 mmol) were added to a solution of 3-benzyloxy-4-methoxy-phenethylamine (16.4 g, 63.8 mmol) in dichloromethane (200 mL) and the mixture was stirred for 3 h at room temperature. Water (100 mL) was added, the phases were separated and the aqueous phase was extracted with CH_2Cl_2 (2 × 30 mL). The combined organic phases were dried over Na_2SO_4 and evaporated under reduced pressure to give 20.7 g (98%) of ethyl 3-benzyloxy-4-methoxyphenethylcarbamate as a yellowish solid.

mp: 104–105 °C

TLC (CH_2Cl_2 : MeOH : NH_3(aq) = 90:9:1): R_f = 0.93.

^{1}H-NMR (300 MHz, $CDCl_3$) [ppm]: 7.29–7.47 (5H, m, Ar), 6.76–6.88 (3H, m, Ar), 5.15 (2H, s, $PhCH_2O$), 4.64 (1H, br s, NH), 4.12 (2H, q, J = 6.9 Hz, OCH_2CH_3), 3.88 (3H, s, OCH_3), 3.37 (2H, dt, J_1 = 6.5 Hz, J_2 = 6.3 Hz, CH_2CH_2-N), 2.71 (2H, t, J = 6.8 Hz, CH_2CH_2-N), 1.25 (3H, t, J = 7.1 Hz, OCH_2CH_3).

^{13}C-NMR (300 MHz, $CDCl_3$) [ppm]: 156.6, 148.4, 148.2, 137.1, 131.3, 128.5, 127.9, 127.4, 121.4, 114.9, 112.1, 71.1, 60.7, 56.1, 42.1, 35.6, 14.7.

MS (EI, 70 eV): m/z = 329 (M^+, 7), 283 (9), 240 (8), 91 (100).

HRMS calcd for $C_{19}H_{23}NO_4$: 329.1627; found: 329.1644.

3-Benzyloxy-4-methoxy-*N*-methylphenethylamine

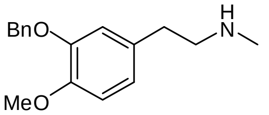

A solution of ethyl 3-benzyloxy-4-methoxyphenethylcarbamate (19.8 g, 60.1 mmol) in anhydrous THF (200 mL) under argon atmosphere was cooled to 0 °C on an ice bath. $LiAlH_4$ (11.4 g, 301 mmol) was added in portions to the stirred solution, the ice bath was removed and the mixture was refluxed for 4 h. The suspension was diluted with THF (100 mL) and cooled to 0 °C on an ice bath. To the vigorously stirred mixture water (11.4 mL), 15% aqu. NaOH solution (11.4 mL) and again water (34.2 mL) were added, the ice bath was removed and stirring continued for 1 h at room temperature. The resulting suspension was filtered through Celite, washed with THF, dried over Na_2SO_4 and evaporated under reduced pressure to give 15.3 g of a

brownish liquid. Flash chromatography (silica; CH_2Cl_2 : MeOH : NH_3(aq) = 90:9:1) afforded 3-benzyloxy-4-methoxy-*N*-methylphenethylamine (11.3 g, 69%) as a yellowish liquid which crystallized upon standing in the fridge to a yellowish solid.

mp: 49–50 °C

TLC (CH_2Cl_2 : MeOH : NH_3(aq) = 90:9:1): R_f = 0.21.

^{1}H-NMR (300 MHz, $CDCl_3$) [ppm]: 7.31–7.47 (5H, m, Ar), 6.76–6.86 (3H, m, Ar), 5.17 (2H, s, $PhCH_2O$), 3.88 (3H, s, OCH_3), 2.67–2.79 (4H, m, CH_2CH_2N), 1.28 (1H, br s, NH), 2.39 (3H, s, NCH_3).

^{13}C-NMR (75 MHz, $CDCl_3$) [ppm]: 148.2, 148.0, 137.2, 132.6, 128.5, 127.8, 127.4, 121.3, 115.0, 112.0, 71.0, 56.1, 53.3, 36.4, 35.6.

MS (EI, 70 eV): *m/z* = 271 (M^+, <1), 228 (69), 137 (10), 91 (73), 65 (10), 58 (51), 44 (100).

HRMS calcd for $C_{17}H_{21}NO_2$: 271.1572; found: 271.1563.

N-(3-Benzyloxy-4-methoxyphenethyl)-2-(3-benzyloxyphenyl)acetamide

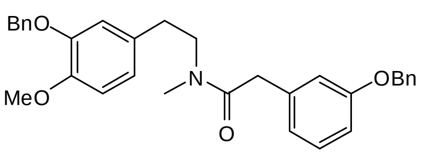

3-Benzyloxy-4-methoxy-*N*-methylphenethylamine (4.14 g, 15.3 mmol) was dissolved in $CHCl_3$ (30 mL). Aqueous NaOH solution (3%, 150 mL) was added and the mixture was cooled to 0 °C on an ice bath. A solution of 3-benzyloxyphenylacetyl chloride[1] (4.12 g, 15.8 mmol) in chloroform (20 mL) was added dropwise over 1 h to the vigorously stirred mixture. The ice bath was removed and stirring was continued for 16 h at room temperature. The phases were separated and the aqueous phase was extracted with $CHCl_3$ (50 mL). The combined organic phases were washed with 1 M HCl solution (100 mL), then water (100 mL) and dried over Na_2SO_4. Evaporation of the solvent under reduced pressure yielded 7.23 g of a yellow liquid. Flash chromatography (silica; hexanes : ethyl acetate = 1:1) afforded *N*-(3-benzyloxy-4-methoxyphenethyl)-2-(3-benzyloxyphenyl)acetamide (5.17 g, 68%) as a pale yellowish liquid.

TLC (hexanes : ethyl acetate = 1:1): R_f = 0.32.

MS (EI, 70 eV): *m/z* = 495 (M^+, 4), 240 (52), 91 (100).

HRMS calcd for $C_{32}H_{33}NO_4$: 495.2410; found: 495.2441.

NMR spectroscopy reveals that the product is a mixture of isomers (ratio *trans*/*cis* = 1.09/1). Based on the peak intensities as well as the DEPT, COSY and HSQC spectra, the NMR signals were assigned to the isomers as follows:

trans-*N*-(3-Benzyloxy-4-methoxyphenethyl)-2-(3-benzyloxyphenyl)acetamide.
^{1}H-NMR (300 MHz, $CDCl_3$) [ppm]: 7.16–7.40 (11H, m, Ar), 6.60–6.88 (6H, m, Ar), 5.09 (2H, s, Ph-CH_2-O), 5.03 (2H, s, Ph-CH_2-O), 3.83 (3H, s, OCH_3), 3.60 (2H, s, CH_2-CO), 3.51 (2H, t, *J* = 7.2 Hz, CH_2-CH_2-N), 2.76 (5H, t + s overlap, *J* = 7.4 Hz, CH_2-CH_2-N + NCH_3).

^{13}C-NMR (75 MHz, $CDCl_3$) [ppm]: 170.6, 159.0, 148.3, 148.0, 137.2, 137.0, 136.6, 131.7, 129.7, 128.6, 128.5, 128.0, 127.8, 127.5, 127.4, 121.5, 121.4, 115.3, 114.8, 113.2, 111.9, 70.9, 69.9, 56.1, 50.3, 41.3, 36.6, 33.2.

cis-*N*-(3-benzyloxy-4-methoxyphenethyl)-2-(3-benzyloxyphenyl)acetamide.
^{1}H-NMR (300 MHz, $CDCl_3$) [ppm]: 7.16–7.40 (11H, m, Ar), 6.60–6.88 (6H, m, Ar), 5.11 (2H, s, Ph-CH_2-O), 5.01 (2H, s, Ph-CH_2-O), 3.83 (3H, s, OCH_3), 3.37 (4H, t + s overlap, *J* = 7.1 Hz, CH_2-CH_2-N + CH_2-CO), 2.91 (3H, s, NCH_3), 2.56 (2H, t, *J* = 7.2 Hz, CH_2-CH_2-N),

^{13}C-NMR (75 MHz, $CDCl_3$) [ppm]: 170.8, 159.0, 148.7, 148.3, 137.1, 137.0, 136.9, 130.6, 129.6, 128.6, 127.9, 127.8, 127.5, 127.3, 121.6, 121.4, 115.3, 114.9, 113.2, 112.1, 71.2, 69.9, 56.1, 52.1, 40.7, 34.1, 33.6.

1-(3-Benzyloxybenzyl)-6-benzyloxy-7-methoxy-2-methyl-1,2,3,4-tetrahydroisoquinoline

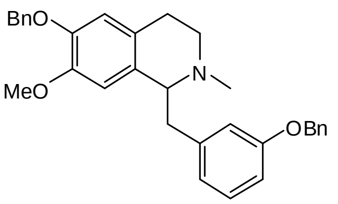

A solution of *N*-(3-benzyloxy-4-methoxyphenethyl)-2-(3-benzyloxyphenyl)acetamide (4.22 g, 8.51 mmol) and $POCl_3$ (3.83 g, 25.0 mmol) in dry acetonitrile (80 mL) was refluxed for 3 h under argon atmosphere. The solvent and excess $POCl_3$ were evaporated under reduced pressure and the residue was dissolved in dry methanol (50 mL), put under argon and cooled to −5 °C on an ice/NaCl bath. $NaBH_4$ (2.42 g, 64.0 mmol) was added in portions to the stirred mixture. The ice bath was then removed and stirring was continued for 16 h at room temperature. The solvent was evaporated and the residue was treated with half-saturated aqu. Na_2CO_3 solution (60 mL). The product was extracted with CH_2Cl_2 (3 × 30 mL), the combined organic phases were dried over Na_2SO_4 and evaporated under reduced pressure to give 4.31 g of a brownish liquid. Flash chromatography (silica; CH_2Cl_2 : MeOH : NH_3(aq) = 96:3:1) afforded 1-(3-benzyloxybenzyl)-6-benzyloxy-7-methoxy-2-methyl-1,2,3,4-tetrahydroisoquinoline (3.72 g, 91%) as a pale yellowish liquid.

TLC (CH_2Cl_2 : MeOH : NH_3(aq) = 90:9:1): R_f = 0.65.

^{1}H-NMR (300 MHz, $CDCl_3$) [ppm]: 7.25–7.43 (10H, m, Ar), 7.17 (1H, t, J = 7.8 Hz, Ar), 6.70–6.82 (3H, m, Ar), 6.59 (1H, s, Ar), 6.04 (1H, s, Ar), 5.08 (2H, s, Ph-CH_2O), 5.00 (2H, s, Ph-CH_2O), 3.71 (1H, dd, J_1 = 7.4 H, J_2 = 5.4 Hz, CH), 3.53 (3H, s, OCH_3), 3.10–3.19 (2H, m, CH_2), 2.71–2.81 (3H, m, CH_2), 2.51–2.57 (1H, m, CH_2), 2.51 (3H, s, NCH_3).

^{13}C-NMR (75 MHz, $CDCl_3$) [ppm]: 158.7, 147.0, 146.5, 141.8, 137.4, 137.1, 130.0, 129.1, 128.6, 128.5, 127.9, 127.7, 127.5, 127.3, 125.8, 122.6, 116.4, 113.9, 112.3, 111.6, 71.0, 69.9, 64.8, 55.6, 46.8, 42.7, 41.2, 25.5.

MS (EI, 70 eV): m/z = 478 (M^+–H, <1), 282 (100), 190 (33), 91 (28).

HRMS calcd for $C_{32}H_{32}NO_3$ (M^+–H): 478.2382; found: 478.2400.

1-(3-hydroxybenzyl)-7-methoxy-2-methyl-1,2,3,4-tetrahydroisoquinolin-6-ol

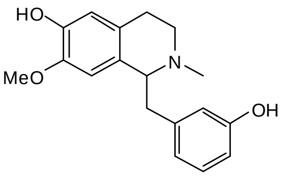

A mixture of 1-(3-benzyloxybenzyl)-6-benzyloxy-7-methoxy-2-methyl-1,2,3,4-tetrahydroisoquinoline (3.52 g, 7.33 mmol), Pd 10% on activated charcoal (0.26 g), acetic acid (0.95 g, 15.7 mmol) and dry methanol (50 mL) was stirred under H_2 atmosphere (1 atm) for 16 h. The mixture was filtered through Celite, washed with methanol (100 mL) and evaporated under reduced pressure. The residue was dissolved in CH_2Cl_2 (50 mL) and washed with half-saturated aqu. $NaHCO_3$ solution (100 mL). The organic phase was dried over Na_2SO_4 and evaporated under reduced pressure to afford 1-(3-hydroxybenzyl)-6-hydroxy-7-methoxy-2-methyl-1,2,3,4-tetrahydroisoquinoline (2.14 g, 98%) as a slightly yellowish solid.

mp: 119–121 °C
TLC (CH_2Cl_2 : MeOH : NH_3(aq) = 90:9:1): R_f = 0.28.

^{1}H-NMR (300 MHz, $CDCl_3$) [ppm]: 7.06 (3H, t + br s overlap, J = 8.0 Hz, Ar), 6.65 (1H, d, J = 8.4 Hz, Ar), 6.59 (1H, s, Ar), 6.50–6.52 (2H, m, Ar), 5.80 (1H, s, Ar), 3.74–3.79 (1H, m, CH), 3.43 (3H, s, OCH_3), 3.16–3.28 (2H, m, CH_2), 2.83–2.93 (2H, m, CH_2), 2.56–2.68 (2H, m, CH_2), 2.51 (3H, s, NCH_3).
^{13}C-NMR (75 MHz, $CDCl_3$) [ppm]: 157.0, 144.3, 141.0, 129.4, 127.4, 125.2, 121.5, 116.8, 114.5, 113.8, 110.8, 64.7, 55.4, 45.6, 41.7, 40.8, 24.1.

MS (EI, 70 eV): m/z = 298 (M^+–H, <1), 192 (100), 177 (19), 148 (5).
HRMS calcd for $C_{18}H_{20}NO_3$ (M^+–H): 298.1443; found: 298.1452.

4.2. Synthesis of 1i:

1-(3-hydroxy-4-methoxybenzyl)-7-methoxy-2-methyl-1,2,3,4-tetrahydroisoquinolin-6-ol.

3-(Benzyloxy)-4-methoxybenzaldehyde

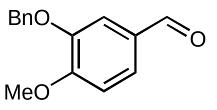

The benzylation[7] of 3-hydroxy-4-methoxybenzaldehyde (isovanillin) was carried out using 20.0 g (131.5 mmol) of substrate. The substrate was dissolved in 120 mL of ethanol and K_2CO_3 (20.1 g) and benzyl bromide (22.50 g, 131.55 mmol) were added. The reaction mixture was allowed to react at room temperature over night. After reaction control via TLC, the reaction was stopped by filtration through celite and the removal of ethanol under reduced pressure. The residue was dissolved in dichloromethane and washed three times with aqu. NaOH solution (5%; 50 mL). The combined organic layers were dried over Na_2SO_4 and the solvent was removed under reduced pressure. Purification: Recrystallization from EtOH. Yield after purification: 29.7 g (93%).

TLC (hexanes : ethyl acetate = 3:1): R_f = 0.45.

^{1}H-NMR (300 MHz, $CDCl_3$) [ppm]: 9.84 (1H, s, CHO), 7.47–7.50 (4H, m, Ar), 7.33–7.42 (3H, m, Ar), 7.01 (1H, d, J = 8.7 Hz, Ar), 5.21 (2H, s, $PhCH_2O$), 3.98 (3H, s, OCH_3).

^{13}C-NMR (75 MHz, $CDCl_3$) [ppm]: 190.8, 155.1, 148.7, 136.3, 130.0, 128.6, 128.1, 127.5, 126.9, 111.4, 110.8, 70.9, 56.2.

The NMR data are in accordance with literature.[4]

1-(3-(Benzyloxy)-4-methoxyphenyl)-2,2,2-trichloroethanol[8]

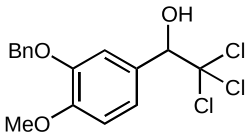

The benzyl-protected isovanillin (29.7 g, 122.6 mmol) was converted to 1-(3-(benzyloxy)-4-methoxyphenyl)-2,2,2-trichloroethanol using chloroform (35 mL), DMF (119 mL) and KOH (8.88 g, 158.3 mmol) in 27 mL of methanol. KOH in methanol was added dropwise under argon atmosphere and cooling using a salt-ice bath (approx. –10 °C). The reaction mixture was allowed to react for 2 h at –9 °C before quenching the mixture still under cooling with 1 M HCl (270 mL). After stirring for additional 30 min at –10 °C the reaction was allowed to warm to ambient temperature and phase separation was performed. The organic layer was washed with water (50 mL) and brine (50 mL) and the solvent was removed under reduced pressure after drying with sodium sulfate. No further purification steps were necessary and the product was directly used for the next step. Yield: 42.9 g (97%).

TLC (hexanes : ethyl acetate = 3:1): R_f = 0.53.

^{1}H-NMR (300 MHz, $CDCl_3$) [ppm]: 7.54 – 7.10 (m, 7H, Ar), 6.89 (d, J = 8.7, 1H, Ar), 5.21 (s, 2H, O-CH_2-Ar), 5.09 (s, 1H, CH-OH), 3.92 (s, 3H, O-CH_3).

^{13}C-NMR (75 MHz, $CDCl_3$) [ppm]: 150.5, 147.1, 137.9, 137.0, 128.6, 127.3, 122.6, 115.1, 110.6, 103.4, 84.1, 71.0, 56.0.

HRMS calcd for $C_{16}H_{15}Cl_3O_3$360.0087; found: 360.0117.

2-(3-(Benzyloxy)-4-methoxyphenyl)acetic acid

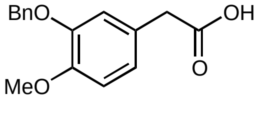

For the synthesis of 2-(3-(benzyloxy)-4-methoxyphenyl)acetic acid, oxygen-free ethanol was needed, therefore 300 mL of ethanol were purged with argon for 60 min. After purging, diphenyl diseleninde (36.9 g, 118.2 mmol) was added and solubilized followed by the addition of $NaBH_4$ (8.9 g, 235.6 mmol) in portions under constant argon flow. After completed addition, the previously orange solution turned colorless and this

solution was stirred for 30 min at ambient temperature. After 30 min the substrate 1-(3-(benzyloxy)-4-methoxyphenyl)-2,2,2-trichloroethanol (40.8 g, 112.8 mmol) was added and dissolved followed by the addition of NaOH (27.1 g, 677.5 mmol). The reaction was heated up to 40 °C and was allowed to react over night. After monitoring *via* TLC and when all starting material was consumed, the reaction was stopped by removal of solvent under reduced pressure. The remaining solid was dissolved in water (50 mL) and adjusted to pH 1 under cooling with concentrated aqu. HCl solution. Then extraction with ethyl acetate (5 times 100 mL) was performed and the combined organic layers were dried over Na_2SO_4. Ethyl acetate was removed under reduced pressure and product was purified by recrystallization from acetone/hexanes. Yield: 11.0 g (36%).

TLC (hexanes : ethyl acetate = 3:1): R_f = 0.19.

^{1}H-NMR (300 MHz, $CDCl_3$) [ppm]: 7.60 – 7.26 (m, 5H, Ar), 7.06 – 6.86 (m, 3H, Ar), 5.07 (s, 2H, O-CH_2-Ar), 3.82 (s, 3H, O-CH_3), 3.49 (s, 2H. Ar-CH_2-CO).

^{13}C-NMR (75 MHz, $CDCl_3$) [ppm]: 172.6, 148.7, 148.0, 137.3, 128.0, 127.8, 127.4, 122.1, 115.6, 112.0, 70.9, 55.2, 40.5.

2-(3-(Benzyloxy)-4-methoxyphenyl)acetyl chloride

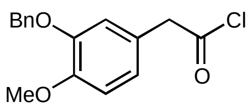

For the synthesis of 2-(3-(benzyloxy)-4-methoxyphenyl)acetyl chloride, 2-(3-(benzyloxy)-4-methoxyphenyl)acetic acid (1.7 g, 6.24 mmol) was dissolved in dry toluene (40 mL) and oxalyl chloride (1.2 g, 9.4 mmol) and one drop of DMF were added. The reaction was allowed to react for 3 hours and was stopped by removing toluene and remaining oxalyl chloride under reduced pressure. The product was directly used for the next step without further purification.

N-(3-(Benzyloxy)-4-methoxyphenethyl)-2-(3-(benzyloxy)-4-methoxyphenyl)-*N*-methylacetamide

N-(3-(benzyloxy)-4-methoxyphenethyl)-2-(3-(benzyloxy)-4-methoxyphenyl)-*N*-methylacetamide was synthesized by the use of 2-(3-(benzyloxy)-4-methoxyphenyl)acetyl chloride (1.2 g, 4.1 mmol) and amino-part 2-(3-(benzyloxy)-4-methoxyphenyl)-*N*-methylethanamine (1.1 g, 4.2 mmol; synthesized as described previously[1, 2]). Sub-strate 2-(3-(benzyloxy)-4-methoxyphenyl)-*N*-methylethanamine was dissolved in chloroform (10 mL) and aqu. NaOH solution (3%, 10 mL) was added. This mixture was cooled using an ice bath and 2-(3-(benzyloxy)-4-methoxyphenyl)acetyl chloride, dissolved in chloroform (10 mL), was added dropwise under cooling. After completed addition of 2-(3-(benzyloxy)-4-methoxyphenyl)acetyl chloride, the ice bath was removed and the reaction was allowed to proceed at room temperature for 16 h. For work-up the phases were separated and the aqueous phase was extracted with chloroform (3 times 10 mL). The combined organic layers were washed with 1 M aqu. HCl solution (30 mL) and dried over Na_2SO_4. Chloroform was removed under reduced pressure and the crude product (3.0 g) was purified using silica gel chromatography (hexanes : ethyl acetate = 1:1). Yield: 1.79 g (83%).

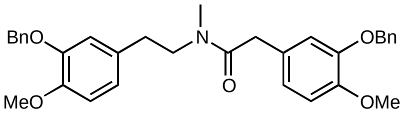

TLC (hexanes : ethyl acetate = 1:1): R_f = 0.25.

NMR spectroscopy reveals that the product is a mixture of isomers (ratio *trans*/*cis* = 1.11/1). Based on the peak intensities as well as the DEPT, COSY and HSQC spectra, the NMR signals were assigned to the isomers as follows:

^{1}H-NMR (300 MHz, $CDCl_3$) [ppm]: *trans*-isomer: 7.41 – 7.13 (m, 10H, Ar), 6.79 – 6.47 (m, 6H, Ar), 5.09 – 4.97 (m, 4H, Ph-CH_2-O), 3.77 (s, 6H, O-CH_3), 3.44 (s, 2H, CO-CH_2-Ar), 3.39 (t, *J* = 7.4, 2H, Ar-CH_2-CH_2-N), 2.65 – 2.56 (m, 5H, Ar-CH_2-CH_2-N, N-CH_3).
cis-isomer: 7.41 – 7.13 (m, 10H, Ar), 6.79 – 6.47 (m, 6H, Ar), 5.09 – 4.97 (m, 4H, Ph-CH_2-O), 3.77 (s, 6H, O-CH_3), 3.26-3.22 (m, 4H, CO-CH_2-Ar, Ar-CH_2-CH_2-N), 2.79 (s, 3H, N-CH_3), 2.44 (t, *J* = 7.0, 2H, Ar-CH_2-CH_2-N).

^{13}C-NMR (75 MHz, $CDCl_3$) [ppm]: *trans*-isomer: 170.9, 148.5, 148.2, 148.2, 148.0, 137.1, 137.1, 131.7, 130.7, 128.5, 128.5, 127.9, 127.8, 127.4, 127.3, 121.5, 121.4, 114.9, 114.7, 112.1, 112.0, 70.9, 70.9, 56.1, 56.0, 50.2, 40.8, 36.5, 33.2.
cis-isomer: 171.1, 148.6, 148.5, 148.2, 148.2, 137.2, 137.1, 131.7, 130.7, 128.6, 128.5, 127.8, 127.7, 127.4, 127.4, 121.5, 121.5, 115.0, 114.6, 112.0, 112.0, 71.2, 70.9, 56.1, 56.0, 51.9, 40.2, 33.6, 34.1.

HRMS calcd for $C_{33}H_{35}NO_5$: 525.2515; found: 525.2542.

6-(Benzyloxy)-1-(3-(benzyloxy)-4-methoxybenzyl)-7-methoxy-2-methyl-1,2,3,4-tetrahydroisoquinoline

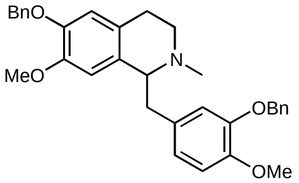

For the cyclization of *N*-(3-(benzyloxy)-4-methoxyphenethyl)-2-(3-(benzyloxy)-4-methoxyphenyl)-*N*-methylacetamide, the following procedure was used. Employing dried glassware, *N*-(3-(benzyloxy)-4-methoxyphenethyl)-2-(3-(benzyloxy)-4-methoxyphenyl)-*N*-methylacetamide (1.8 g, 3.4 mmol) was dissolved in dry acetonitrile (50 mL) and phosphoryl chloride (1.8 g, 11.9 mmol) was added under constant argon flow. The reaction was heated to reflux for 3 h and stopped by removing the solvent under reduced pressure. The resulting residue was used for the next step without further purification.
In the next step the residue was dissolved in dry methanol (50 mL) and the solution was cooled using an ice bath. Under cooling and a constant flow of argon, $NaBH_4$ (1.3 g, 33.3 mmol) was added in portions. After completion of addition of $NaBH_4$, the ice bath was removed and the reaction was allowed to proceed at room temperature for 16 h. The reaction was stopped by removal of methanol under reduced pressure. The residue was redissolved in half-saturated aqu. sodium carbonate solution (10 mL) and extracted with CH_2Cl_2 (3 times 20 mL). The combined organic layers were dried over Na_2SO_4 and CH_2Cl_2 was removed under reduced pressure. Purification was carried out using silica gel chromatography (CH_2Cl_2 : MeOH : NH_3OH = 98:1:1). Yield: 1.5 g (85%).

TLC (CH_2Cl_2 : MeOH : NH_3OH = 90:9:1): R_f = 0.43.

^{1}H-NMR (300 MHz, $CDCl_3$) [ppm]: 7.53 – 7.19 (m, 10H, Ar), 6.77 (d, *J* = 8.1, 1H, Ar), 6.70 – 6.52 (m, 3H, Ar), 6.03 (s, 1H, Ar), 5.08 (s, 2H, O-C*H₂*-Ar), 5.05 (s, 2H, O-C*H₂*-Ar) 3.84 (s, 3H), 3.57 (s+m overlap, 4H, O-C*H₃* + N-C*H*), 3.13 – 3.02 (m, 2H, N-CH-C*H₂*), 2.83 – 2.60 (m, 3H, N-C*H₂*-C*H₂*), 2.54 – 2.48 (m, 1H, N-C*H₂*-C*H₂*), 2.46 (s, 3H, N-C*H₃*).

^{13}C-NMR (75 MHz, $CDCl_3$) [ppm]: 148.0, 147.7, 147.0, 146.5, 137.3, 132.5, 130.0, 128.5, 127.8, 127.3, 126.1, 122.6, 115.9, 113.9, 111.6, 71.0, 64.8, 56.1, 55.7, 47.0, 42.7, 40.6, 25.6.

HRMS calcd for $C_{33}H_{35}NO_4$ (M^+–H): 508.2488; found: 508.2513.

1-(3-Hydroxy-4-methoxybenzyl)-7-methoxy-2-methyl-1,2,3,4-tetrahydroisoquinolin-6-ol

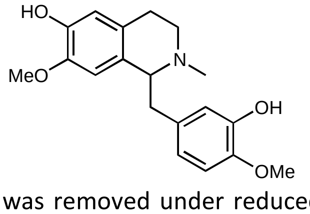

Deprotection of 6-(benzyloxy)-1-(3-(benzyloxy)-4-methoxybenzyl)-7-methoxy-2-methyl-1,2,3,4-tetrahydroisoquinoline was carried out using palladium on charcoal under hydrogen atmosphere. Therefore substrate (1.45 g, 2.84 mmol) was dissolved in dry methanol (50 mL) and acetic acid (0.38 mL). Palladium on charcoal (0.15 g) was added and hydrogen (1 atm) was applied after evacuation.
After 16 h of reaction time the mixture was filtered through celite and methanol was removed under reduced pressure. The residue was dissolved in dichloromethane and washed with half-saturated aqu. sodium bicarbonate solution (10 mL) and the organic phase was dried over Na_2SO_4. The solvent was again removed under reduced pressure. No further purification was necessary. Yield: 0.93 g (99%).

mp: 88–90 °C

TLC (CH_2Cl_2 : MeOH : NH_3OH = 90:9:1): R_f = 0.23.

^{1}H-NMR (300 MHz, $CDCl_3$) [ppm]: 6.80 – 6.28 (m, 4H, Ar), 5.92 (s, 1H, Ar), 3.82 (s, 3H, O-C*H*$_3$), 3.74 – 3.62 (m, 1H, N-C*H*), 3.51 (s, 3H, O-C*H*$_3$), 3.19 – 3.09 (m, 2H, N-CH-C*H*$_2$), 2.86 – 2.76 (m, 2H, C*H*$_2$-C*H*$_2$-N), 2.70 – 2.52 (m, 2H, C*H*$_2$-C*H*$_2$-N), 2.50 (s, 3H, N-C*H*$_3$).

^{13}C-NMR (75 MHz, $CDCl_3$) [ppm]: 145.7, 145.4, 144.4, 144.2, 133.0, 128.1, 125.9, 121.2, 116.3, 114.5, 110.8, 110.7, 64.9, 56.0, 55.5, 46.1, 42.1, 40.6, 24.6.

HRMS calcd for $C_{19}H_{23}NO_4$ (M^+–H): 328.1549; found: 328.1566.

4.3. Synthesis of 1j:

6,7-Dimethoxy-1-(3,5-dihydroxybenzyl)-2-methyl-1,2,3,4-tetrahydroisoquinoline

Methyl (3,5-bisbenzyloxy)phenylacetate

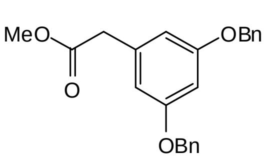

A mixture of methyl 3,5-dihydroxyphenylacetate (2.50 g, 13.7 mmol), benzyl bromide (6.30 g, 36.2 mmol), K_2CO_3 (5.00 g, 36.2 mmol) and KI (0.1 g, 0.6 mmol) in acetone (50 mL) was refluxed for 16 h. The solvent was evaporated under reduced pressure and the residue was dissolved in water (100 mL). The product was extracted with EtOAc (2 × 50 mL), the combined organic phases were dried over Na_2SO_4 and evaporated under reduced pressure to give 6.13 g of a yellow liquid. Flash chromatography (silica; petrol ether/EtOAc = 9/1) afforded methyl (3,5-bisbenzyloxy)phenylacetate (3.93 g, 79%) as a yellowish liquid.

TLC (petrol ether/EtOAc = 1/1): R_f = 0.80.

^{1}H-NMR ($CDCl_3$, 300 MHz): δ = 3.59 (2H, s, C*H*$_2$-$COOCH_3$), 3.72 (3H, s, OCH_3), 5.05 (4H, s, Ph-CH_2-O), 6.58 (3H, s, Ar), 7.15–7.44 (10H, m, Ar).

^{13}C-NMR ($CDCl_3$, 75 MHz): δ = 41.5, 52.1, 70.1, 100.9, 108.5, 127.6, 128.0, 128.6, 136.1 136.8, 160.1, 171.8. The NMR data are in accordance with literature.[9]

(3,5-Bisbenzyloxy)phenylacetic acid

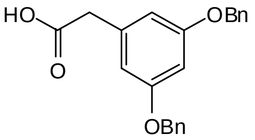

A suspension of methyl (3,5-bisbenzyloxy)phenyl-acetate (3.90 g, 10.8 mmol) in 2 M aqueous NaOH solution (50 mL) was refluxed for 16 h. The mixture was cooled on an ice bath and acidified by addition of conc. hydrochloric acid. The product was extracted with EtOAc (2 × 50 mL), the combined organic phases were dried over Na_2SO_4 and evaporated under reduced pressure to give 3.72 g of a yellowish solid. Recrystallisation from petrol ether/acetone afforded (3,5-bisbenzyloxy)phenylacetic acid (3.11 g, 82%) as an off-white solid.

mp: 101–102 °C (ref.[10] 108 °C).

TLC (petrol ether/EtOAc = 1/1 + 1 drop AcOH): R_f = 0.51.

^{1}H-NMR ($CDCl_3$, 300 MHz): δ = 3.62 (2H, s, C*H*$_2$-COOH), 5.05 (4H, s, Ph-C*H*$_2$-O), 6.59 (3H, s, Ar), 7.28–7.46 (10H, m, Ar).

^{13}C-NMR ($CDCl_3$, 75 MHz): δ = 41.3, 70.1, 101.0, 108.7, 127.6, 128.0, 128.6, 135.4, 136.8, 160.0, 177.4. The NMR data are in accordance with literature.[9]

(3,5-Bisbenzyloxy)phenylacetyl chloride

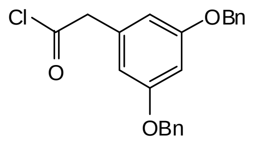

A solution of (3,5-bisbenzyloxy)phenylacetic acid (3.08 g, 8.85 mmol), oxalyl chloride (1.35 g, 10.6 mmol) and one drop of DMF in dry toluene (40 mL) was stirred at room temperature under argon for 1 h. The solvent was evaporated under reduced pressure to give 3.25 g (quant.) of (3,5-bisbenzyloxy)phenylacetyl chloride as a brownish liquid that was used in the following transformation without further purification.

^{1}H-NMR ($CDCl_3$, 300 MHz): δ = 4.08 (2H, s, C*H₂*-COCl), 5.05 (4H, s, Ph-CH_2-O), 6.54 (2H, d, J = 2.1 Hz, Ar), 6.62 (H, t, J = 1.8 Hz, Ar), 7.28–7.46 (10H, m, Ar).

^{13}C-NMR ($CDCl_3$, 75 MHz): δ = 53.2, 70.2, 101.7, 108.8, 127.6, 128.1, 128.7, 133.2, 136.6, 160.3, 171.7.

2-(3,5-Bisbenzyloxyphenyl)-*N*-(3,4-dimethoxyphenethyl)-*N*-methylacetamide

N-Methylhomoveratrylamine (1.66 g, 8.51 mmol) was dissolved in $CHCl_3$ (20 mL). An aqueous 3% NaOH

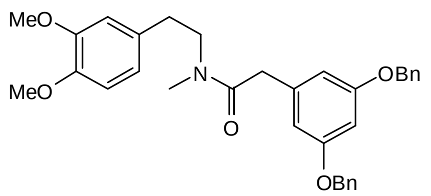

solution (100 mL) was added and the mixture was cooled to 0 °C on an ice bath. A solution of (3,5-bisbenzyloxy)phenylacetyl chloride (3.23 g, 8.80 mmol) in chloroform (20 mL) was added dropwise over 1 h to the vigorously stirred mixture. The ice bath was removed and stirring was continued for 2 h. The phases were separated and the aqueous phase was extracted with $CHCl_3$ (50 mL). The combined organic phases were washed with dilute HCl solution (100 mL), then water (50 mL) and dried over Na_2SO_4. Evaporation of the solvent under reduced pressure yielded 4.39 g of a highly viscous yellow liquid. Flash chromatography (silica; petrol ether/EtOAc = 1/1) afforded 2-(3,5-bisbenzyloxy-phenyl)-*N*-(3,4-dimethoxyphenethyl)-*N*-methylacetamide (3.05 g, 66%) as a yellowish liquid.

TLC (petrol ether/EtOAc = 1/1): R_f = 0.21.

MS (EI, 70 eV): m/z = 525 (M^+, 6), 270 (6), 164 (100), 151 (7), 91 (50).
HRMS calcd for $C_{33}H_{35}NO_5$: 525.2515; found: 525.2532.

NMR spectroscopy reveals that the product is a mixture of isomers (ratio *trans*/*cis* = 1.08/1). Based on the peak intensities as well as the DEPT, COSY and HMQC spectra, the NMR signals can be assigned to the isomers as follows:

trans: ^{1}H-NMR ($CDCl_3$, 300 MHz): δ = 2.76 (2H, t, J = 7.5 Hz, CH_2-CH$_2$-N), 2.92 (3H, s, N-CH_3), 3.41 (2H, s, CO-CH_2-Ar), 3.55 (2H, t, J = 7.5 Hz, CH_2-N), 3.82 (6H, s, 2 × OCH_3), 5.01 (4H, s, Ar-CH_2-O), 6.43–6.77 (6H, m, Ar), 7.25–7.42 (10H, m, Ar). ^{13}C-NMR ($CDCl_3$, 75 MHz): δ = 33.3, 33.6, 41.2, 52.2, 55.9, 70.0, 100.5, 107.9, 111.2, 112.0, 120.7, 127.6, 128.0, 128.6, 131.7, 136.8, 137.2, 147.9, 149.1, 160.1, 170.7.

cis: ^{1}H-NMR ($CDCl_3$, 300 MHz): δ = 2.59 (2H, t, J = 7.4 Hz, CH_2-CH$_2$-N), 2.95 (3H, s, N-CH_3), 3.42 (2H, t , J = 7.2, CH_2-N), 3.62 (2H, s, CO-C*H2*-Ar), 3.82 (6H, s, 2 × OCH_3), 4.99 (4H, s, Ar-CH_2-O), 6.43–6.77 (6H, m, Ar), 7.25–7.42 (10H, m, Ar). ^{13}C-NMR ($CDCl_3$, 75 MHz): δ = 34.2, 36.7, 41.7, 50.4, 55.9, 70.0, 100.5, 107.8, 111.4, 111.9, 120.8, 127.6, 128.0, 128.6, 130.7, 136.8, 137.6, 147.5, 148.9, 160.1, 170.5.

1-(3,5-Bisbenzyloxy)benzyl-6,7-dimethoxy-2-ethyl-1,2,3,4-tetrahydroisoquinoline

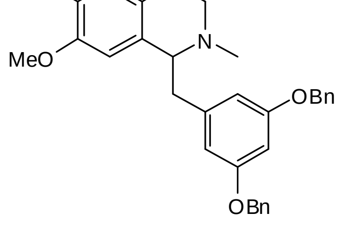

A solution of 2-(3,5-bisbenzyloxyphenyl)-*N*-(3,4-dimethoxyphen-ethyl)-*N*-methylacetamide (3.00 g, 5.71 mmol) and $POCl_3$ (2.66 g, 17.4 mmol) in dry acetonitrile (50 mL) was refluxed for 3 h under argon atmosphere. The solvent and excess $POCl_3$ were evaporated under reduced pressure and the residue was dissolved in dry methanol (40 mL), put under argon and cooled to 0 °C on an ice bath. $NaBH_4$ (2.11 g, 55.8 mmol) was added in portions to the stirred mixture. The ice bath was then removed and stirring continued for 16 h at room temperature. The solvent was evaporated and the residue was treated with half-saturated Na_2CO_3 solution (50 mL). The product was extracted with

CH_2Cl_2 (3 × 15 mL), the combined organic phases were dried over Na_2SO_4 and evaporated under reduced pressure to give 2.84 g of a yellow liquid. Flash chromatography (silica; CH_2Cl_2/MeOH/NH_3(aq) = 96/3/1) afforded 1-(3,5-bisbenzyloxy)benzyl-6,7-dimethoxy-2-ethyl-1,2,3,4-tetrahydroisoquinoline (2.37 g, 82%) as a yellowish liquid.

TLC (CH_2Cl_2/MeOH/NH_3(aq) = 90/9/1): *R*f = 0.63.

^{1}H-NMR ($CDCl_3$, 300 MHz): δ = 2.54 (3H, s, NCH_3), 2.58–2.92 (4H, m, CH_2), 3.13–3.22 (2H, m, CH_2), 3.59 (3H, s, OCH_3), 3.73 (1H, dd, J_1 = 7.4 Hz, J_2 = 3.7 Hz, CH), 3.86 (3H, s, OCH_3), 5.00 (4H, s, $PhCH_2O$), 6.10 (1H, s, Ar), 6.42 (2H, s, Ar), 6.50 (1H, s, Ar), 6.58 (1H, s, Ar), 7.31–7.44 (10H, m, Ar).

^{13}C-NMR ($CDCl_3$, 75 MHz): δ = 25.5, 41.6, 42.7, 46.8, 55.5, 55.8, 64.6, 70.0, 99.8, 109.0, 111.0, 111.2, 125.8, 127.6, 128.0, 128.6, 129.4, 137.0, 142.6, 146.4, 147.3, 159.7.

MS (EI, 70 eV): *m/z* = 508 (M^+–H, <1), 206 (100), 190 (9), 91 (13). HRMS calcd for $C_{33}H_{34}NO_4$ (M^+–H): 508.2488; found: 508.2511.

1-(3,5-Dihydroxybenzyl)-6,7-dimethoxy-2-ethyl-1,2,3,4-tetrahydroisoquinoline

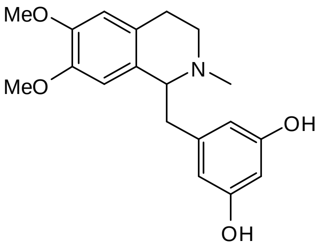

A mixture of 1-(3,5-bisbenzyloxy)benzyl-6,7-dimethoxy-2-ethyl-1,2,3,4-tetrahydroisoquinoline (2.36 g, 4.63 mmol), Pd 10% on activated charcoal (0.25 g), acetic acid (0.80 g, 13.3 mmol) and dry methanol (50 mL) was stirred under H_2 atmosphere (balloon) for 16 h. The mixture was filtered through Celite, washed with methanol (50 mL) and evaporated under reduced pressure. The residue was dissolved in EtOAc (50 mL) and washed with half-saturated $NaHCO_3$ solution (40 mL). [**NOTE**: In halogenated solvents such as dichloromethane and chloroform, the substance will oligomerize, as indicated by heavily broadened NMR signals.] The organic phase was dried over Na_2SO_4 and evaporated under reduced pressure to afford 1-(3,5-dihydroxybenzyl)-6,7-dimethoxy-2-ethyl-1,2,3,4-tetrahydroisoquinoline (1.22 g, 80%) as an off-white solid.

mp: 108–110 °C.

TLC (CH_2Cl_2/MeOH/NH_3(aq) = 90/9/1): *R*f = 0.38.

^{1}H-NMR (acetone-d_6, 300 MHz): δ = 2.45 (3H, s, NCH_3), 2.52–2.81 (4H, m, CH_2), 3.01 (1H, dd, J_1 = 13.6 Hz, J_2 = 5.7 Hz, CH_2), 3.09–3.17 (1H, m, CH_2), 3.59 (3H, s, OCH_3), 3.67 (1H, t, J = 6.2 Hz, CH), 3.75 (3H, s, OCH_3), 6.20 (3H, s, Ar), 6.35 (1H, s, Ar), 6.61 (1H, s, Ar).

^{13}C-NMR (acetone-d_6, 75 MHz): δ = 25.4, 40.9, 42.2, 54.8, 55.0, 64.6, 100.1, 108.3, 111.6, 111.7, 126.1, 129.9, 142.8, 147.0, 147.7, 158.1.

MS (EI, 70 eV): *m/z* = 328 (M^+–H, <1), 206 (100), 190 (13).
HRMS calcd for $C_{19}H_{22}NO_4$ (M^+–H): 328.1549; found: 328.1558.

4.4. Synthesis of 1k:

3-((6,7-Dimethoxy-2-methyl-1,2,3,4-tetrahydroisoquinolin-1-yl)methyl)-4-fluorophenol

2,2,2-Trichloro-1-(2-fluoro-5-methoxyphenyl)ethanol[8]

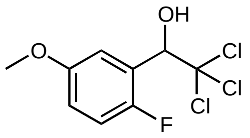

2-Fluoro-5-methoxybenzaldehyde (5.0 g, 32.4 mmol) was converted to 2,2,2-trichloro-1-(2-fluoro-5-methoxyphenyl)ethanol using chloroform (6 mL), DMF (15 mL) and KOH (1.5 g) in 5 mL of methanol. 2-Fluoro-5-methoxybenzaldehyde was dissolved in DMF and chloroform and cooled to approx. –10 °C using a salt-ice bath. KOH in methanol was added dropwise under argon atmosphere and cooling was continued for additional 2 h before quenching the mixture still under cooling with concentrated aqu. HCl solution to a pH of 1. After stirring for additional 30 min at –10 °C the reaction was allowed to warm to ambient temperature and phase separation was performed. The aqueous phase was extracted with toluene (3 times 30 mL). Combined organic layers were washed with water (30 mL) and brine (30 mL) and the solvent was removed under reduced pressure. No further purification steps were necessary. Yield: 8.6 g (97%).

TLC (Eluent: hexanes : ethyl acetate = 3:1): R_f = 0.25.

^{1}H-NMR (300 MHz, $CDCl_3$) [ppm]: 7.31-7.27 (m, 1H, Ar), 7.02 (t, *J* = 9.2 Hz, 1H, Ar), 6.94-6.89 (m, 1H, Ar), 5.60 (s, 1H, C*H*-OH), 3.12 (s, 3H, C*H₃*), 3.35 (s, 1H, OH)

^{13}C-NMR (75 MHz, $CDCl_3$) [ppm]: 155.3 (d, J_{CF} = 2.0 Hz), 154.9 (d, J_{CF} = 241.4 Hz), 123.4 (d, J_{CF} = 13.6 Hz), 116.6 (d, J_{CF} = 8.3 Hz), 115.8 (d, J_{CF} = 24.6 Hz), 114.1 (d, J_{CF} = 2.7 Hz), 102.6, 77.3, 55.9.

HRMS calcd for $C_9H_8Cl_3FO_2$: 271.9574; found: 271.9588.

2-(2-Fluoro-5-methoxyphenyl)acetic acid

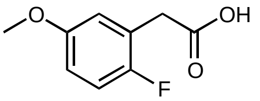

For the synthesis of 2-(2-fluoro-5-methoxyphenyl)acetic acid, oxygen-free ethanol was needed, therefore 100 mL of ethanol were purged with argon for 60 min. After purging, diphenyl diselenide (10.6 g, 34.0 mmol) was added and solubilized followed by the addition of $NaBH_4$ (2.6 g, 68.0 mmol) in portions under constant argon flow. After complete addition, the previously orange solution turned colorless and this solution was stirred for 30 min at ambient temperature. After 30 min 2,2,2-trichloro-1-(2-fluoro-5-methoxyphenyl)ethanol (8.6 g, 31.3 mmol) was added and dissolved followed by the addition of NaOH (7.5 g, 178.9 mmol). The reaction was heated up to 40 °C and was allowed to react over night.
After monitoring *via* TLC and when all starting material was consumed, the reaction was stopped by removal of solvent under reduced pressure. The remaining solid was dissolved in water (20 mL) and extracted under basic conditions with ethyl acetate (30 mL). After this extraction the aqueous phase was adjusted to pH 1 under cooling with concentrated aqu. HCl solution. Then extraction with ethyl acetate (5 times 30 mL) was performed and the combined organic layers were dried over Na_2SO_4. Ethyl acetate was removed under reduced pressure and the product was purified by silica gel column chromatography using a gradient of hexanes (to remove the non-reacted diphenyl diselenide and apolar by-products) and hexanes : aceton = 1:1 to elute the product. Yield: 3.1 g (54%).

TLC (hexanes : ethyl acetate = 3:1 + acetic acid): R_f = 0.24.

^{1}H-NMR (300 MHz, MeOH-d4) [ppm]: 7.00 (t, *J* = 9.1 Hz, 1H, Ar), 6.88-6.79 (m, 2H, Ar), 3.77 (s, 3H, O-C*H₃*), 3.63 (s, 2H, Ar-C*H₂*-COOH),

^{13}C-NMR (75 MHz, MeOH-d4) [ppm]: 173.1, 155.7 (d, J_{CF} = 2.0 Hz), 155.5 (d, J_{CF} = 237.1 Hz), 155.5 (d, J_{CF} = 18.1 Hz), 113.3 (d, J_{CF} = 3.9 Hz), 115.1 (d, J_{CF} = 24.1 Hz), 113.3 (d, J_{CF} = 8.3 Hz), 54.8, 33.8.

2-(2-Fluoro-5-hydroxyphenyl)acetic acid

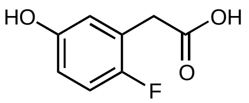

2-(3-Methoxy-2-methylphenyl)acetic acid (3.1 g, 16.8 mmol) was dissolved in bromic acid (48% in water, 25 mL) and heated up to 105 °C using an oil bath. After a reaction time of 5 h the reaction was allowed to cool down to room temperature and extracted with ethyl acetate (3 times 30 mL). The combined organic layers were dried over Na_2SO_4 and concentrated under reduced pressure. No further purification was necessary. Yield: 2.8 g (99%).

mp: 99–102 °C

TLC (hexanes : ethyl acetate = 3:1 + acetic acid): R_f = 0.14.

^{1}H-NMR (300 MHz, MeOH-d4) [ppm]: 6.89 (t, *J* = 9.1, 1H, Ar), 6.73 – 6.64 (m, 2H, Ar), 4.97 (s, 1H, OH), 3.58 (s, 2H, Ar-CH_3-COOH).

^{13}C-NMR (75 MHz, MeOH-d4) [ppm]: 173.8, 154.8 (d, J_{CF} = 235.3 Hz), 153.1 (d, J_{CF} = 2.2 Hz), 122.3 (d, J_{CF} = 17.9 Hz), 117.3 (d, J_{CF} = 3.9 Hz), 115.0 (d, J_{CF} = 23.0 Hz), 114.6 (d, J_{CF} = 8.0 Hz), 33.7.

2-(5-(Benzyloxy)-2-fluorophenyl)acetic acid

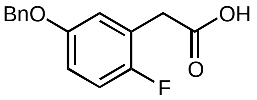

The benzylation of 2-(2-fluoro-5-hydroxyphenyl)acetic acid was carried out using 2.8 g (16.5 mmol) of substrate. The substrate was dissolved in 50 mL of ethanol and KOH (2.3 g, 40.1 mmol), benzyl bromide (3.4 g, 19.8 mmol) and NaI (0.08 g, 0.56 mmol) were added. This reaction mixture was allowed to react at room temperature over night.

After reaction control *via* TLC, the reaction was stopped by filtration through celite and removal of ethanol under reduced pressure. The residue was redissolved in water (30 mL) and acidified with acetic acid. The aqueous phase was extracted with ethyl acetate (3 times 30 mL) and the combined organic layers were dried over Na_2SO_4 followed by the removal of the solvents under reduced pressure. Purification was carried out using silica gel column chromatography with hexanes: ethyl acetate = 3:1 as an eluent system. Yield after purification: 4.0 g (91%).

mp: 120–122 °C

TLC (hexanes : ethyl acetate = 3:1 + acetic acid): R_f = 0.24.

^{1}H-NMR (300 MHz, methanol-d4) [ppm]: 7.45-7.33 (m, 5H, Ar), 7.03-6.88 (m, 3H, Ar), 5.05 (s, 2H, O-CH_2-Ar), 3.63 (s, 2H, Ar-CH_2-COOH).

^{13}C-NMR (75 MHz, methanol-d4) [ppm]: 173.0, 155.7 (d, J_{CF} = 237.9 Hz), 154.9, 137.2, 128.1, 127.5, 127.2, 122.6 (d, J_{CF} = 17.7 Hz), 117.5 (d, J_{CF} = 3.9 Hz), 115.1 (d, J_{CF} = 24.0 Hz), 114.4 (d, J_{CF} = 7.6 Hz), 70.2, 33.9.

2-(5-(Benzyloxy)-2-fluorophenyl)acetyl chloride

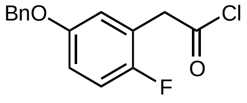

For the synthesis of 2-(5-(benzyloxy)-2-fluorophenyl)acetyl chloride, 2-(5-(benzyloxy)-2-methylphenyl)acetic acid (1.1 g, 3.5 mmol) was dissolved in dry toluene (10 mL) and oxalyl chloride (0.89 g, 7.0 mmol) and one drop of DMF were added. The reaction was allowed to proceed for 3 h and was stopped by removing toluene and remaining oxalyl chloride under reduced pressure. The product 2-(5-(benzyloxy)-2-methylphenyl)acetyl chloride was directly used for the next step without further purification.

2-(5-(Benzyloxy)-2-fluorophenyl)-*N*-(3,4-dimethoxyphenethyl)-*N*-methylacetamide

2-(5-(Benzyloxy)-2-fluorophenyl)-*N*-(3,4-dimethoxyphenethyl)-*N*-methylacetamide was synthesized by the use of 2-(5-(benzyloxy)-2-fluorophenyl)acetyl chloride (1.2 g, 3.8 mmol) and previously prepared amino-part 2-(3,4-dimethoxyphenyl)-*N*-methylethanamine (0.75 g, 3.8 mmol). 2-(3,4-dimethoxyphenyl)-*N*-methylethanamine was dissolved in chloroform (10 mL) and aqueous NaOH-solution (3%, 50 mL) was added. This mixture was cooled using an ice bath and 2-(5-(benzyloxy)-2-fluorophenyl)acetyl chloride, dissolved in chloroform (10 mL), was added dropwise under cooling. After complete addition of 2-(5-(benzyloxy)-2-fluorophenyl)acetyl chloride, the ice bath was removed and the reaction was allowed to proceed at room temperature for 16 h. For work-up the phases were separated and the aqueous phase was extracted with chloroform (3 times 30 mL). The combined organic layers were washed with 1 M aqu. HCl solution and dried over Na_2SO_4. Chloroform was removed under reduced pressure and the crude product was purified using silica gel chromatography (hexanes : ethyl acetate = 1:1). Yield: 0.75 g (45%).

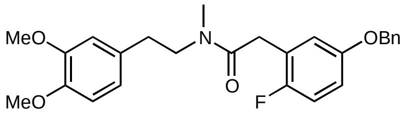

TLC (hexanes : ethyl acetate = 1:1): R_f = 0.23.

NMR spectroscopy reveals that the product is a mixture of isomers (ratio *trans*/*cis* = 1.04/1). Based on the peak intensities as well as the DEPT, COSY and HSQC spectra, the NMR signals were assigned to the isomers as follows:

^{1}H-NMR (300 MHz, $CDCl_3$) [ppm]:
trans: 7.36-7.24 (m, 5H, Ar), 6.92-6.82 (m, 2H, Ar), 6.77-6.57 (m, 4H, Ar), 4.94 (s, 2H, O-CH_2-Ar), 3.80-3.77 (m, 6H, 2x O-CH_3), 3.58 (s, 2H, CH-CH_2-Ar), 3.50 (t, 2H, *J*=7.5 Hz, Ar-CH_2-C*H*$_2$-N), 2.84 (s, 3H, N-CH_3), 2.72 (t, 2H, *J*=7.7 Hz, Ar-C*H*$_2$-CH_2-N).
cis: 7.21-7.37 (m, 5H, Ar), 6.82-6.94 (m, 1H, Ar), 6.57-6.77 (m, 5H, Ar), 4.92 (s, 2H, O-CH_2-Ar), 3.80-3.77 (m, 6H, 2x O-CH_3), 3.41 (t, 2H, *J*=7.2 Hz, Ar-CH_2-C*H*$_2$-N), 3.36 (s, 2H, CO-CH_2-Ar), 2.90 (s, 3H, N-CH_3), 2.64 (t, 2H, *J*=7.2 Hz, Ar-C*H*$_2$-CH_2-N).

^{13}C-NMR (75 MHz, $CDCl_3$) [ppm]:
trans: 169.7, 155.2 (d, J_{CF} = 238 Hz), 154.9, 149.1, 147.9, 136.8, 131.6, 128.6, 128.0, 127.5, 123.1 (d, J_{CF} = 5.6 Hz), 120.7, 116.7 (d, J_{CF} = 3.9 Hz), 115.6 (d, J_{CF} = 1.3 Hz), 114.9, 112.0, 111.2, 70.5, 55.9, 50.6, 36.4, 34.3, 33.3.
cis: 170.0, 154.9 (d, J_{CF} = 237 Hz), 154.9, 148.9, 147.5, 136.8, 130.5, 128.5, 128.0, 127.5, 122.9 (d, J_{CF} = 5.4 Hz), 120.8, 116.4 (d, J_{CF} = 4.0 Hz), 115.9 (d, J_{CF} = 1.3 Hz), 114.7, 111.9, 111.4, 70.5, 55.9, 52.1, 34.0, 33.8, 33.2.

HRMS calcd for $C_{26}H_{28}FNO_4$: 437.2002; found: 437.2024.

1-(5-(Benzyloxy)-2-fluorobenzyl)-6,7-dimethoxy-2-methyl-1,2,3,4-tetrahydroisoquinoline

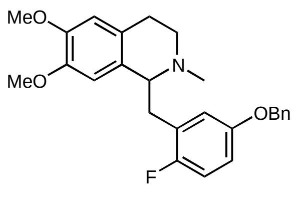

For the cyclization of 2-(5-(benzyloxy)-2-fluorophenyl)-*N*-(3,4-dimethoxyphenethyl)-*N*-methylacetamide, the following procedure was used employing dried glassware: 2-(5-(benzyloxy)-2-fluorophenyl)-*N*-(3,4-dimethoxyphenethyl)-*N*-methylacetamide (0.73 g, 0.17 mmol) was dissolved in dry acetonitrile (20 mL) and phosphoryl chloride (0.81 g, 5.3 mmol) was added under constant argon flow. The reaction was heated to reflux for 3 h and stopped by removing solvents under reduced pressure. The resulting residue was used for the next step without further purification. In the next step the residue was dissolved in dry methanol (20 mL) and is cooled using an ice bath. Under cooling and a constant flow of argon, $NaBH_4$ (0.63 g, 16.8 mmol) was added in portions. After completion of addition of $NaBH_4$, the ice bath was removed and the reaction was allowed to proceed at room temperature for 16 h. The reaction was stopped by removal of methanol under reduced pressure. The residue was dissolved in half-saturated aqu. sodium carbonate solution and extracted with CH_2Cl_2 (3 times 30 mL). The combined organic layers were dried over Na_2SO_4 and CH_2Cl_2 was removed under reduced pressure. Purification was carried out using silica gel chromatography (CH_2Cl_2 : MeOH : NH_3OH = 98:1:1). Yield: 0.59 g (85%).

TLC (CH_2Cl_2 : MeOH : NH_3OH = 90:9:1): R_f = 0.32.

^{1}H-NMR (300 MHz, $CDCl_3$) [ppm]: 7.42 – 7.33 (m, 5H, Ar), 6.96 (t, *J* = 9.1, 1H, Ar), 6.80-6.75 (m, 1H, Ar), 6.72-6.69 (m, 1H, Ar), 6.58 (s, 1H, Ar), 6.12 (s, 1H, Ar), 4.96 (s, 2H, O-C*H₂*-Ar), 3.85 (s, 3H, Ar-O-C*H₃*), 3.82-3.77 (m, 1H, N-C*H*-Ar), 3.61 (s, 3H, Ar-O-C*H₃*), 3.26-3.12 (m, 2H, CH_2), 2.93-2.75 (m, 3H, CH_2), 2.65-2.58 (m, 1H, CH_2), 2.53 (s, 3H, N-C*H₃*)

^{13}C-NMR (75 MHz, $CDCl_3$) [ppm]: 156.1 (d, J_{CF} = 237.6 Hz), 154.4 (d, J_{CF} = 1.9 Hz), 147.4, 146.5, 136.9, 129.0, 128.6, 128.0, 127.8 (d, J_{CF} = 17.0 Hz), 127.7, 125.9, 118.1 (d, J_{CF} = 4.8 Hz), 115.5 (d, J_{CF} = 24.4 Hz), 113.5 (d, J_{CF} = 8.0 Hz), 110.7, 70.5, 63.3, 55.7, 55.5, 46.4, 42.5, 34.3, 25.3.

HRMS calcd for $C_{26}H_{26}FNO_3$ (M^+–H): 420.1975; found: 420.2007.

3-((6,7-Dimethoxy-2-methyl-1,2,3,4-tetrahydroisoquinolin-1-yl)methyl)-4-fluorophenol

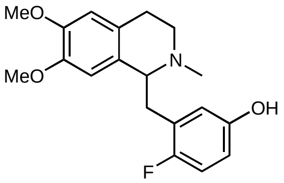

Deprotection of 1-(5-(Benzyloxy)-2-fluorobenzyl)-6,7-dimethoxy-2-methyl-1,2,3,4-tetrahydroisoquinoline was carried out using palladium on charcoal under hydrogen atmosphere. The substrate (0.59 g, 1.4 mmol) were dissolved in dry methanol (10 mL) and acetic acid (0.10 mL). Palladium on charcoal (0.20 g) was added and hydrogen (1 atm) was applied after evacuation.

After 16 h of reaction time the mixture was filtered through celite and methanol was removed under reduced pressure. The residue was dissolved in dichloromethane (10 mL) and washed with half-saturated aqu. sodium bicarbonate solution (5 mL) and the organic phase was dried (Na_2SO_4). The solvent was again removed under reduced pressure. No further purification was necessary. Yield: 0.45 (99%).

mp: 65–67 °C

TLC (CH_2Cl_2 : MeOH : NH_3OH = 90:9:1): R_f = 0.33.

^{1}H-NMR (300 MHz, $CDCl_3$) [ppm]: 6.71 (t, *J* = 9.2, 1H, Ar), 6. 50 (s, 1H, Ar), 6.42-6.33 (m, 2H, Ar), 6.13 (s, 1H, Ar), 3.81 (m, 1H, N-C*H*-Ar), 3.74 (s, 3H, Ar-O-C*H₃*), 3.56 (s, 3H, Ar-O-C*H₃*), 3.25-3.17 (m, 1H, CH_2), 2.93-2.75 (m, 4H, CH_2), 2.61-2.53 (m, 4H, CH_2), 2.41 (s, 3H, N-CH_3)

^{13}C-NMR (75 MHz, $CDCl_3$) [ppm]: 155.2 (d, J_{CF} = 235.5 Hz), 152.5 (d, J_{CF} = 2.0 Hz), 147.6, 146.8, 128.3, 126.7 (d, J_{CF} = 16.7 Hz), 124.9, 118.5 (d, J_{CF} = 4.5 Hz), 115.7 (d, J_{CF} = 24.0 Hz), 114.4 (d, J_{CF} = 8.0 Hz), 111.2, 110.7, 63.3, 55.6, 45.3, 35.0, 29.7, 23.8.

HRMS calcd for $C_{19}H_{22}FNO_3$ (M^+–H): 330.1505; found: 330.1518.

4.5. Synthesis of 1l:

2-Chloro-3-((6,7-dimethoxy-2-methyl-1,2,3,4-tetrahydroisoquinolin-1-yl)methyl)phenol

3-(Benzyloxy)-2-chlorobenzaldehyde

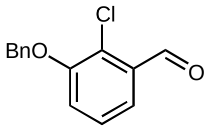

2-Chloro-3-hydroxybenzaldehyde (5.0 g, 31.9 mmol) was used as a substrate for the benzylation. The substrate was dissolved in 50 mL of ethanol and K_2CO_3 (4.9 g, 35.1 mmol) was added. After addition of K_2CO_3, benzyl bromide (5.5 g , 31.9 mmol) was added and the reaction mixture was allowed to react over night.

After the reaction was completed, the mixture was filtered through celite and the celite was washed with ethanol (3 times 20 mL). Ethanol was removed under reduced pressure and the residue was dissolved in dichloromethane and washed with aqu. NaOH solution (5%, 30 mL). Organic layers were combined and dried over Na_2SO_4 and the solvent was removed under reduced pressure. The remaining residue was purified by recrystallization from ethanol. Yield after purification: 4.4 g (56%).

mp: 100–102 °C

TLC (hexanes : ethyl acetate = 3:1): R_f = 0.63.

^{1}H-NMR (300 MHz, $CDCl_3$) [ppm]: 10.56 (s, 1H, O-CH), 5.14 (2H, s, CH_2-O), 7.57-7.20 (m, 8H, Ar), 5.22 (s, 2H, O-CH_2-Ar)
^{13}C-NMR (75 MHz, $CDCl_3$) [ppm]: 190.1, 154.7, 135.7, 133.7, 128.7, 128.3, 127.5, 127.4, 127.1, 121.1, 119.0, 71.3.

1-(3-(Benzyloxy)-2-chlorophenyl)-2,2,2-trichloroethanol[8]

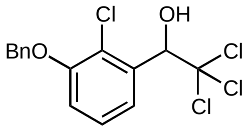

3-(Benzyloxy)-2-chlorobenzaldehyde (4.3 g, 17.5 mmol) was converted to 1-(3-(benzyloxy)-2-chlorophenyl)-2,2,2-trichloroethanol using chloroform (6 mL), DMF (20 mL) and KOH (1.5 g) in 5 mL of methanol. 3-(Benzyloxy)-2-chlorobenzaldehyde was dissolved in DMF and chloroform and cooled to approx. –10 °C using a salt-ice bath. KOH in methanol was added dropwise under argon atmosphere and cooling was continued for additional 2 h before quenching the mixture still under cooling with concentrated aqu. HCl solution to pH 1. After stirring for additional 30 min at –10 °C the reaction was allowed to warm to ambient temperature and phase separation was performed. The aqueous phase was extracted with toluene (3x 30 mL). The combined organic layers were washed with water (30 mL) and brine (30 mL) and the solvent was removed under reduced pressure. No further purification steps were necessary. Yield: 6.3 g (99%).

TLC (hexanes : ethyl acetate = 3:1): R_f = 0.20.

^{1}H-NMR (300 MHz, $CDCl_3$) [ppm]: 7.54-7.07 (m, 8H, Ar), 6.00 (s, 1H, Ar-C*H*-OH), 5.19 (s, 2H, Ar-CH_2-O) 3.46 (s, 1H, Ar-CH_2-O*H*).

^{13}C-NMR (75 MHz, $CDCl_3$) [ppm]: 154.0, 136.3, 135.2, 128.7, 128.1, 127.1, 126.8, 124.6, 121.6, 114.6, 102.8, 79.6, 71.1.

HRMS calcd for $C_{15}H_{12}Cl_4O_2$ (M^+–H): 365.9563; found: 365.9592.

2-(3-(Benzyloxy)-2-chlorophenyl)acetic acid

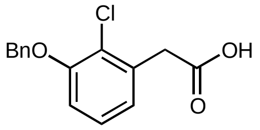

For the synthesis of 2-(3-(benzyloxy)-2-chlorophenyl)acetic acid, oxygen-free ethanol was needed, therefore 60 mL of ethanol were purged with argon for 60 min. After purging, diphenyl diselenide (5.7 g, 18.3 mmol) was added and solubilized followed by the addition of $NaBH_4$ (1.4 g, 36.5 mmol) in portions under constant argon flow. After complete addition the previously orange solution turned colorless and this solution was stirred for 30 min at ambient temperature. After 30 min 1-(3-(benzyloxy)-2-chlorophenyl)-2,2,2-trichloroethanol (6.3 g, 17.3 mmol) was added and dissolved followed by the addition of NaOH (4.2g, 104.1 mmol). The reaction was heated up to 40 °C and was allowed to react over night.

After monitoring *via* TLC and when all starting material was consumed, the reaction was stopped by removal of solvent under reduced pressure. The remaining solid was dissolved in water and extracted under basic conditions with ethyl acetate (30 mL). After this extraction the aqueous phase was adjusted to pH 1 under cooling with concentrated aqu. HCl solution. Extraction with ethyl acetate (5 times 30 mL) was performed and the combined organic layers were dried (Na_2SO_4). Ethyl acetate was removed under reduced pressure and the product was purified by silica gel column chromatography using a gradient of hexanes (to remove the non-reacted diphenyl diselenide and apolar by-products) and hexanes : acetone = 1:1 to elute the product. Yield: 2.7 g (56%).

mp: 148–151 °C

TLC (hexanes : ethyl acetate (+ 1 drop of acetic acid) = 3:1): R_f = 0.24.

^{1}H-NMR (300 MHz, acetone-d6) [ppm]: 7.56-7.53 (m, 2H, Ar), 7.45-7.22 (m, 4H, Ar), 7.13 (dd, 1H, J_1=8.4, J_2=1.5), 7.03 (dd, 1H, J_1=7.5, J_2=1.5), 5.23 (s, 2H, O-CH_2-Ar), 3.82 (s, 2H, Ar-C*H₂*-COOH).
^{13}C-NMR (75 MHz, acetone-d6) [ppm]: 205.3, 170.7, 154.4, 137.0, 134.9, 128.4, 127.8, 127.3, 127.1, 123.7, 112.5, 70.4, 38.5.

HRMS calcd for $C_{15}H_{13}ClO_3$: 276.0553; found: 276.0573.

2-(3-(Benzyloxy)-2-chlorophenyl)acetyl chloride

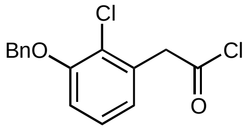

For the synthesis of 2-(3-(benzyloxy)-2-chlorophenyl)acetyl chloride, 2-(3-(benzyloxy)-2-chlorophenyl)acetic acid (1.0 g, 3.7 mmol) was dissolved in dry toluene (20 mL) and oxalyl chloride (0.93 g, 7.3 mmol) and one drop of DMF were added. The reaction was allowed to proceed for 3 h and was stopped by removing toluene and remaining oxalyl chloride under reduced pressure. The product 2-(3-(benzyloxy)-2-chlorophenyl)acetyl chloride was directly used for the next step without further purification.

2-(3-(Benzyloxy)-2-chlorophenyl)-*N*-(3,4-dimethoxyphenethyl)-*N*-methylacetamide

2-(3-(Benzyloxy)-2-chlorophenyl)-*N*-(3,4-dimethoxyphenethyl)-*N*-methylacetamide was synthesized from 2-(3-(benzyloxy)-2-chlorophenyl)acetyl chloride (0.98 g, 3.5 mmol) and 2-(3,4-dimethoxyphenyl)-N-methylethanamine (0.72 g, 3.7 mmol). 2-(3,4-Dimethoxyphenyl)-*N*-methylethanamine was dissolved in chloroform (10 mL) and aqu. NaOH solution (3%, 10 mL) was added. This mixture was cooled using an ice bath and 2-(3-(benzyloxy)-2-chlorophenyl)acetyl chloride, dissolved in chloroform (10 mL), was added dropwise under cooling. After complete addition of 2-(3-(benzyloxy)-2-chlorophenyl)acetyl chloride, the ice bath was removed and the reaction was allowed to proceed at room temperature for 16 h. For work-up the phases were separated and the aqueous phase was extracted with chloroform (3 times 10 mL). The combined organic layers were washed with 1 M aqu. HCl solution and dried (Na_2SO_4). Chloroform was removed under reduced pressure and the crude product (1.2 g) was purified using silica gel chromatography (hexanes : ethyl acetate = 1:1). Yield: 1.3 g (75%).

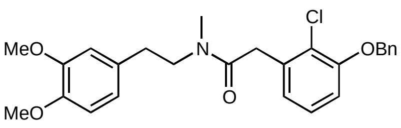

TLC (hexanes : ethyl acetate = 1:1): R_f = 0.24.

NMR spectroscopy reveals that the product is a mixture of isomers (ratio *trans*/*cis* = 1.11/1). Based on the peak intensities as well as the DEPT, COSY and HSQC spectra, the NMR signals were assigned to the isomers as follows:

^{1}H-NMR (300 MHz, $CDCl_3$) [ppm]:
trans: 7.41-7.21 (m, 5H, Ar), 7.05 (td, 2H, J_1=8.0 Hz, J_2=2.2 Hz, Ar), 6.83-6.67 (m, 4H, Ar), 5.07 (s, 2H, Ar-CH_2-O), 3.77 (s, 6H, O-CH_3), 3.73 (s, 2H, Ar-CH_2-CO), 3.55 (t, 2H, *J*=7.7 Hz, N-CH_2-CH_2-Ar), 2.87 (s, 3H, N-CH_3), 2.74 (t, 2H, *J*=7.6 Hz, N-CH_2-CH_2-Ar).
cis: 7.41-7.21 (m, 5H, Ar), 6.83-6.67 (m, 4H, Ar), 6.61-6.56 (m, 2H, Ar), 5.05 (s, 2H, Ar-CH_2-O), 3.78 (s, 6H, O-CH_3), 3.56 (s, 2H, Ar-CH_2-CO), 3.40 (t, 2H, *J*=7.3 Hz, N-CH_2-CH_2-Ar), 2.92 (s, 3H, N-CH_3), 2.66 (t, 2H, *J*=7.3 Hz, N-CH_2-CH_2-Ar).

^{13}C-NMR (75 MHz, $CDCl_3$) [ppm]:
trans: 169.9, 154.4, 148.9, 147.6, 136.6, 135.2, 131.6, 128.6, 127.9, 127.1, 123.1, 122.8, 120.7, 112.4, 112.1, 111.3, 70.9, 55.9, 50.2, 38.7, 36.3, 33.3.
cis: 170.2, 154.4, 149.2, 147.9, 136.5, 135.2, 130.6, 128.6, 127.9, 127.1, 122.9, 122.6, 120.9, 112.3, 112.0, 111.5, 70.9, 55.9, 52.2, 38.0, 34.4, 33.7.

HRMS calcd for $C_{26}H_{28}ClNO_4$: 453.1707; found: 453.1726.

1-(3-(Benzyloxy)-2-chlorobenzyl)-6,7-dimethoxy-2-methyl-1,2,3,4-tetrahydroisoquinoline

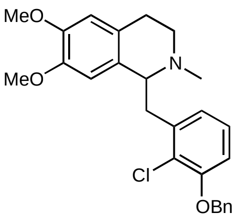

For the cyclization of 2-(3-(benzyloxy)-2-chlorophenyl)-*N*-(3,4-dimethoxyphenethyl)-*N*-methylacetamide, the following procedure was used employing dried glassware: 2-(3-(Benzyloxy)-2-chlorophenyl)-*N*-(3,4-dimethoxyphenethyl)-*N*-methylacetamide (1.2 g, 2.7 mmol) was dissolved in dry acetonitrile (25 mL) and phosphoryl chloride (1.3 g, 8.2 mmol) was added under constant argon flow. The reaction was heated to reflux for 3 h and stopped by removing solvents under reduced pressure. The resulting residue was used for the next step without further purification. In the next step the residue was dissolved in dry methanol (25 mL) and the solution was cooled using an ice bath. Under cooling and a constant flow of argon, $NaBH_4$ (1.0 g, 27.3 mmol) was added in portions. After completion of addition of $NaBH_4$, the ice bath was removed and the reaction was allowed to proceed at room temperature for 16 h. The reaction was stopped by removal of methanol under reduced pressure. The residue was dissolved in half-saturated aqu. sodium carbonate solution and extracted with CH_2Cl_2 (3 times 10 mL). The combined organic layers were dried over Na_2SO_4 and CH_2Cl_2 was removed under reduced pressure. Purification was carried out using silica gel chromatography (CH_2Cl_2 : MeOH : NH_3OH = 98:1:1). Yield: 1.1 g (93%).

mp: 70–72 °C

TLC (CH_2Cl_2: MeOH : NH_3OH = 90:9:1): R_f = 0.37.

^{1}H-NMR (300 MHz, $CDCl_3$) [ppm]: 7.52-7.30 (m, 5H, Ar), 7.05 (t, 1H, J=7.9 Hz, Ar), 6.87 (dd, 1H, J_1=8.3 Hz, J_2=1.5 Hz, Ar), 6.64 (dd, 1H, J_1=7.6 Hz, J_2=1.4 Hz, Ar), 6.59 (s, 1H, Ar), 5.96 (s, 1H, Ar), 5.18 (s, 2H, O-CH_2-Ar), 3.93 (dd, 1H, J_1=7.9 Hz, J_2=5.9 Hz, N-CH-Ar), 3.85 (s, 3H, O-CH_3), 3.49 (s, 3H, O-CH_3), 3.42-3.25 (m, 2H, CH_2), 3.00-2.81 (m, 3H, CH_2), 2.68-2.61 (m, 1H, CH_2), 2.56 (s, 3H, N-CH_3).

^{13}C-NMR (75 MHz, $CDCl_3$) [ppm]: 154.3, 147.3, 146.2, 139.4, 136.4, 129.1, 128.6, 127.9, 127.0, 126.3, 125.7, 124.9, 123.6, 111.7, 111.2, 111.2, 70.9, 62.1, 55.8, 55.4, 46.0, 42.5, 38.9, 25.1.

HRMS calcd for $C_{26}H_{28}ClNO_3$ (M^+–H): 436.1679; found: 436.1715.

2-Chloro-3-((6,7-dimethoxy-2-methyl-1,2,3,4-tetrahydroisoquinolin-1-yl)methyl)phenol

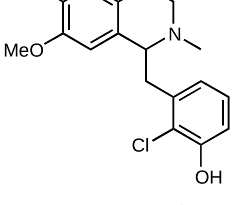

Deprotection of 1-(3-(benzyloxy)-2-chlorobenzyl)-6,7-dimethoxy-2-methyl-1,2,3,4-tetrahydroisoquinoline was carried out using palladium on charcoal under hydrogen atmosphere. The, substrate (1.1 g, 0.32 mmol) was dissolved in dry methanol (20 mL) and acetic acid (0.20 mL). Palladium on charcoal (0.10 g) was added and hydrogen (1 atm) was applied after evacuation.

After 16 h of reaction time the mixture was filtered through celite and methanol was removed under reduced pressure. The residue was dissolved in dichloromethane and washed with half-saturated aqu. sodium bicarbonate solution and the organic phase was dried over Na_2SO_4. At that point GC-MS analysis showed, that hydrogenation not only caused the removal of the benzyl group, but also dehalogenation occurred. Only a small amount of deprotected but halogenated product was available. Deprotected and dehalogenated substance were separated by silica gel chromatography (CH_2Cl_2 : MeOH : NH_3OH = 98:1:1). Yield: 0.08 g (9%).

mp: 65–67 °C

TLC (CH_2Cl_2 : MeOH : NH_3OH = 90:9:1): R_f = 0.40.

^{1}H-NMR (300 MHz, $CDCl_3$) [ppm]: 7.05 (t, 1H, *J*=7.8 Hz, Ar), 6.89 (dd, J_1=8.1 Hz, J_2=1.5 Hz, Ar), 6.62 (dd, 1H, J_1=7.5 Hz, J_2=1.5 Hz, Ar), 6.58 (s, 1H, Ar), 5.88, (s, 1H, Ar), 3.87-3.82 (m, 4H, N-CH-Ar, O-CH_3), 3.51 (s, 3H, O-CH_3), 3.33-3.23 (m, 2H, CH_2), 2.99-2.80 (m, 3H, CH_2), 2.67-2.59 (m, 1H, CH_2), 2.55 (s, 3H, N-CH_3).
^{13}C-NMR (75 MHz, $CDCl_3$) [ppm]: 151.6, 147.4, 146.2, 138.6, 128.3, 127.1, 125.6, 124.0, 120.9, 113.9, 111.2, 111.0, 62.5, 55.8, 55.4, 45.9, 42.4, 38.7, 24.9.

HRMS calcd for $C_{19}H_{22}ClNO_3$ (M^+–H): 346.1210; found: 346.1217.

4.6. Synthesis of 1m:

3-((6,7-Dimethoxy-2-methyl-1,2,3,4-tetrahydroisoquinolin-1-yl)methyl)-2-methylphenol

3-Methoxy-2-methylbenzaldehyde

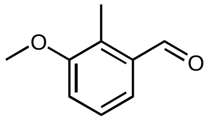

For the synthesis of 3-methoxy-2-methylbenzaldehyde, anisaldehyde (8.0 g, 7.2 mmol) was used as a cheap and easily available substrate. To insert the methyl group at the 2-position *N*,*N*,*N'*-trimethylethylenediamine, *n*-BuLi and methyl iodide as an electrophile were used (see Figure 2).

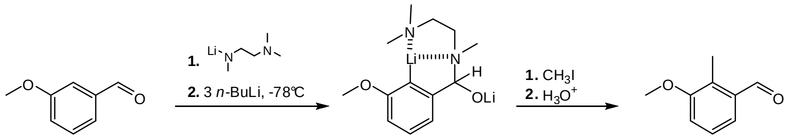

Figure 2: Methylation in 2-position using *N,N,N'*-trimethylethylenediamine, *n*-BuLi and methyl iodide.

n-BuLi (25.1 mL of a 2.5 M solution, 62.7 mmol) was added to *N,N,N'*-trimethylethylenediamine (8.2 mL, 6.4 g, 62.7 mmol) dissolved in freshly distilled, anhydrous THF (100 mL) at –20 °C under argon. After completion of addition of *n*-BuLi, the reaction was allowed to proceed for 15 min at –20 °C, followed by the dropwise addition of anisaldehyde. After 15 min the second portion of *n*-BuLi (75.2 mL of a 2.5 M solution, 188.0 mmol) was added again dropwise and again under cooling (–20 °C) and constant flow of argon. The reaction vessel was closed tightly and put in the freezer (–23 °C) over night. On the next day the reaction mixture was cooled to –40 °C and methyl iodide (21.9 mL, 50.0 g, 352.6 mmol) was added dropwise again under constant flow of argon. This mixture was allowed to react for 10 h at –40 °C. The reaction was stopped by pouring on ice-cold aqu. HCl solution (10%, 150 mL) and extracted with ethyl acetate (5 times 30 mL). The combined organic layers were dried over Na_2SO_4 and the solvent was removed under reduced pressure. The crude product was purified using silica gel purification to remove remaining substrate (eluent system: hexanes : ethyl acetate = 99 : 1). Yield: 4.53 g (51%).

TLC (hexanes : ethyl acetate = 99:1): R_f = 0.13.

^{1}H-NMR (300 MHz, $CDCl_3$) [ppm]: 10.34 (s, 1H, C*H*O), 7.44 (dd, J1 = 7.7, J2 = 1.1, 1H, Ar), 7.32 (t, J = 7.9, 1H, Ar), 7.09 (dd, J = 8.1, 0.5, 1H, Ar), 3.89 (s, 3H, OC*H*$_3$), 2.55 (s, 3H, Ar-C*H*$_3$).
^{13}C-NMR (75 MHz, $CDCl_3$) [ppm]: 192.7, 158.1, 135.1, 129.7, 126.6, 123.0, 115.3, 55.9, 10.4.

2,2,2-Trichloro-1-(3-methoxy-2-methylphenyl)ethanol[8]

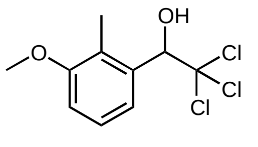

3-Methoxy-2-methylbenzaldehyde (4.5 g, 29.9 mmol) was converted to 2,2,2-trichloro-1-(3-methoxy-2-methylphenyl)ethanol using chloroform (10 mL), DMF (15 mL) and KOH (1.5 g) in 5 mL of methanol. 3-Methoxy-2-methylbenzaldehyde was dissolved in DMF and chloroform and cooled to –10 °C using a salt-ice bath. KOH in methanol was added dropwise under argon atmosphere and cooling was continued for additional 2 h before quenching the mixture still under cooling with concentrated aqu. HCl solution to a pH of 1. After stirring for additional 30 min at –10 °C the reaction was allowed to warm to ambient temperature and phase separation was performed. The aqueous phase was extracted with toluene (3 times 30 mL). The combined organic layers were washed with water (30 mL) and brine (30 mL) and the solvent was removed under reduced pressure. No further purification steps were necessary. Yield: 7.5 g (93%).

TLC (Eluent: hexanes : ethyl acetate = 3:1): R_f = 0.59.

^{1}H-NMR (300 MHz, $CDCl_3$) [ppm]: 7.46 (dd, J_1 = 8.1, J_2 = 1.2, 1H, Ar), 7.26 (t, J=8.1, 1H, Ar), 6.91 (d, J = 8.1, 1H, Ar), 5.64 (s, 1H, Ar-C*H*-OH), 3.86 (s, 3H, O-C*H₃*), 3.36 (s, 1H, O*H*), 2.36 (s, 3H, Ar-C*H₃*).
^{13}C-NMR (75 MHz, $CDCl_3$) [ppm]: 157.4, 135.3, 126.8, 125.9, 119.9, 110.7, 103.7, 79.8, 55.6, 12.8.

HRMS calcd for $C_{10}H_{11}Cl_3O_2$: 267.9825; found: 267.9825.

2-(3-Methoxy-2-methylphenyl)acetic acid

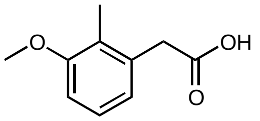

For the synthesis of 2-(3-methoxy-2-methylphenyl)acetic acid, oxygen-free ethanol was needed, therefore 100 mL of ethanol were purged with argon for 60 min. After purging, diphenyl diselenide (9.0 g, 28.9 mmol) was added and solubilized followed by the addition of $NaBH_4$ (2.2 g, 57.4 mmol) in portions under constant argon flow. After complete addition, the previously orange solution turned colorless and was stirred for 30 min at ambient temperature. After 30 min 2,2,2-trichloro-1-(3-methoxy-2-methylphenyl)ethanol (7.5 g, 5.6 mmol) was added and dissolved followed by the addition of NaOH (6.6 g, 165.2 mmol). The reaction was heated up to 40 °C and was allowed to react over night.
After monitoring *via* TLC and when all starting material was consumed, the reaction was stopped by removal of solvent under reduced pressure. The remaining solid was dissolved in water and extracted under basic conditions with ethyl acetate (30 mL). After this extraction the aqueous phase was adjusted to pH 1 under cooling with concentrated aqu. HCl solution. Then extraction with ethyl acetate (5 times 30 mL) was performed and the combined organic layers were dried over Na_2SO_4. Ethyl acetate was removed under reduced pressure and the product was purified by silica gel column chromatography using a gradient of hexanes (to remove the non-reacted diphenyl diselenide and apolar by-products) and hexanes : aceton = 1:1 to elute the product. Yield: 3.3 g (65%).

mp: 112–114 °C

TLC (hexanes : ethyl acetate = 3:1 + acetic acid): R_f = 0.25.

^{1}H-NMR (300 MHz, $CDCl_3$) [ppm]: 7.16 (t, J = 7.9, 1H, Ar), 6.89 – 6.79 (m, 2H, Ar), 3.85 (s, 3H, O-C*H₃*), 3.70 (s, 2H, Ar-C*H₃*), 2.21 (s, 3H, Ar-C*H₂*).

^{13}C-NMR (75 MHz, $CDCl_3$) [ppm]: 177.8, 157.8, 133.2, 126.4, 125.8, 122.6, 109.5, 55.6, 39.1, 11.7.

2-(3-Hydroxy-2-methylphenyl)acetic acid

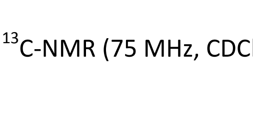

2-(3-Methoxy-2-methylphenyl)acetic acid (3.3 g, 17.8 mmol) was dissolved in bromic acid (48% in water, 30 mL) and heated up to 105 °C using an oil bath. After a reaction time of 5 h the reaction was allowed to cool down to room temperature and extracted with ethyl acetate (3 times 30 mL). The combined organic layers were dried over Na_2SO_4 and concentrated under reduced pressure. No further purification was necessary. Yield: 3.0 g (99%).

mp: 159–161 °C

TLC (hexanes : ethyl acetate = 3:1 + acetic acid): R_f = 0.13.

^{1}H-NMR (300 MHz, $CDCl_3$) [ppm]: 6.95 (t, J = 7.8, 1H, Ar), 6.80-6.72 (m, 2H, Ar), 3.64 (s, 2H, Ar-C*H₂*), 2.18 (s, 3H, Ar-C*H₃*).

^{13}C-NMR (75 MHz, $CDCl_3$) [ppm]: 205.5, 171.8, 155.2, 135.0, 125.8, 121.7, 113.4, 38.6, 10.9.

2-(3-(Benzyloxy)-2-methylphenyl)acetic acid

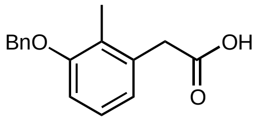

The benzylation of 2-(3-hydroxy-2-methylphenyl)acetic acid was carried out using 3.0 g (18.1 mmol) of substrate. The substrate was dissolved in 50 mL of ethanol and KOH (2.4 g, 43.1 mmol), benzyl bromide (3.9 g, 23.1 mmol) and NaI (0.08 g, 0.56 mmol) were added. This reaction mixture was allowed to react at room temperature over night.

After reaction control *via* TLC, the reaction was stopped by filtration through celite and the removal of ethanol under reduced pressure. The residue was redissolved in water (30 mL) and acidified with acetic acid. The aqueous phase was extracted with ethyl acetate (3 times 30 mL) and the combined organic layers were dried over Na_2SO_4 followed by the removal of the solvents under reduced pressure. Purification was carried out using silica gel column chromatography with hexanes : ethyl acetate = 3:1 as an eluent system. Yield after purification: 3.6 g (78%).

mp: 122–124 °C

TLC (hexanes : ethyl acetate = 3:1 + acetic acid): R_f = 0.25.

^{1}H-NMR (300 MHz, acetone-d4) [ppm]: 7.54-7.50 (m, 2H, Ar), 7.44-7.33 (m, 3H, Ar), 7.11 (t, J = 7.9, 1H, Ar), 6.96 (dd, J_1 = 8.3, J_2 = 1.2, 1H, Ar), 6.88 (dd, J_1 = 7.7, J_2 = 1.1, 1H, Ar), 5.13 (s, 2H, O-C*H₂*), 3.68 (s, 2H, Ar-C*H₂*), 2.24 (s, 3H, Ar-C*H₃*).

^{13}C-NMR (75 MHz, acetone-d4) [ppm]: 171.7, 156.9, 137.8, 134.9, 128.4, 127.6, 127.3, 126.1, 125.7, 122.9, 110.6, 69.8, 38.6, 11.2.

HRMS calcd for $C_{16}H_{16}O_3$ (M^+–H): 256.1100; found: 256.1126.

2-(3-(Benzyloxy)-2-methylphenyl)acetyl chloride

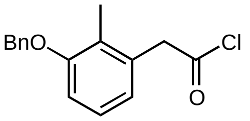

For the synthesis of 2-(3-(benzyloxy)-2-methylphenyl)acetyl chloride, 2-(3-(benzyloxy)-2-methylphenyl)acetic acid (3.6 g, 14.0 mmol) was dissolved in dry toluene (60 mL) and oxalyl chloride (2.7 g, 21.0 mmol) and one drop of DMF were added. The reaction was allowed to proceed for 3 h and was stopped by removing toluene and remaining oxalyl chloride under reduced pressure. The product 2-(3-(benzyloxy)-2-methylphenyl)acetyl chloride was directly used for the next step without further purification.

2-(3-(Benzyloxy)-2-methylphenyl)-*N*-(3,4-dimethoxyphenethyl)-*N*-methylacetamide

2-(3-(Benzyloxy)-2-methylphenyl)-*N*-(3,4-dimethoxyphenethyl)-*N*-methylacetamide was synthesized by the use

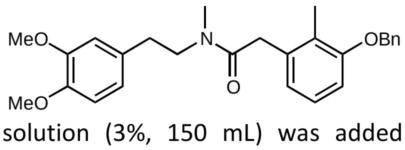

of 2-(3-(benzyloxy)-2-methylphenyl)acetyl chloride (3.8 g, 13.8 mmol) and previously prepared amino-part 2-(3,4-dimethoxyphenyl)-*N*-methylethanamine (3.3 g, 16.8 mmol). 2-(3,4-Dimethoxyphenyl)-*N*-methylethanamine was dissolved in chloroform (30 mL) and aqu. NaOH solution (3%, 150 mL) was added. This mixture was cooled using an ice bath and 2-(3-(benzyloxy)-2-methylphenyl)acetyl chloride, dissolved in chloroform (10 mL), was added dropwise under cooling. After complete addition of 2-(3-(benzyloxy)-2-methylphenyl)acetyl chloride, the ice bath was removed and the reaction was allowed to proceed at room temperature for 16 h. For work-up the phases were separated and the aqueous phase was extracted with chloroform (3 times 30 mL). The combined organic layers were washed with 1 M aqu. HCl solution and dried over Na_2SO_4. Chloroform was removed under reduced pressure and the crude product was purified using silica gel chromatography (hexanes : ethyl acetate = 1:1). Yield: 4.6 g (76%).

TLC (hexanes : ethyl acetate = 1:1): R_f = 0.25.

NMR spectroscopy reveals that the product is a mixture of isomers (ratio *trans*/*cis* = 1.22/1). Based on the peak intensities as well as the DEPT, COSY and HSQC spectra, the NMR signals were assigned to the isomers as follows:

^{1}H-NMR (300 MHz, $CDCl_3$) [ppm]:
trans: 7.21-7.38 (m, 5H, Ar), 6.97-7.03 (m, 1H, Ar), 6.49-6.77 (m, 5H, Ar), 4.98 (s, 2H, O-CH_2-Ar), 3.76-3.77 (m, 6H, overlap, O-CH_3, O-CH_3), 3.53-3.59 (m, 4H, overlap, Ar-CH_2-CO, Ar-CH_2-C*H*$_2$-N), 2.83 (s, 3H, N-CH_3), 2.75 (t, 2H, *J*=7.5 Hz, Ar-C*H*$_2$-CH_2-N),
cis: 7.21-7.38 (m, 5H, Ar), 6.97-7.03 (m, 1H, Ar), 6.49-6.77 (m, 5H, Ar), 4.97 (s, 2H, O-CH_2-Ar), 3.76-3.77 (m, 6H, overlap O-CH_3, O-CH_3), 3.35-3.40 (m, 4H, overlap Ar-CH_2-CO, Ar-CH_2-C*H*$_2$-N), 2.94 (s, 3H, N-CH_3), 2.62 (t, 2H, *J*=7.1 Hz, Ar-C*H*$_2$-CH_2-N).
^{13}C-NMR (75 MHz, $CDCl_3$) [ppm]:
trans: 170.8, 156.9, 148.9, 147.5, 137.5, 135.1, 131.6, 128.5, 127.7, 127.2, 126.3, 125.6, 121.6, 120.7, 112.1, 111.3, 110.4, 70.2, 55.9, 50.1, 39.0, 36.3, 33.3, 11.8.
cis: 171.1, 156.9, 149.1, 147.9, 137.4, 135.3, 130.7, 128.5, 127.7, 127.2, 126.3, 125.5, 121.5, 120.8, 111.9, 111.5, 110.3, 70.2, 55.9, 52.2, 38.5, 34.3, 33.6, 14.2.

1-(3-(Benzyloxy)-2-methylbenzyl)-6,7-dimethoxy-2-methyl-1,2,3,4-tetrahydroisoquinoline

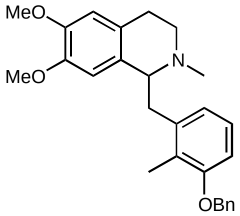

For the cyclization of 2-(3-(benzyloxy)-2-methylphenyl)-*N*-(3,4-dimethoxyphenethyl)-*N*-methylacetamide, the following procedure was used. Employing dried glassware, 2-(3-(Benzyloxy)-2-methylphenyl)-*N*-(3,4-dimethoxyphenethyl)-*N*-methylacetamide (4.6 g, 10.6 mmol) was dissolved in dry acetonitrile (75 mL) and phosphoryl chloride (4.9 g, 31.8 mmol) was added under constant argon flow. The reaction was heated to reflux for 3 h and stopped by removing the solvent under reduced pressure. The resulting residue was used for the next step without further purification. In the next step the residue was dissolved in dry methanol (75 mL) and the solution was cooled using an ice bath. Under cooling and a constant flow of argon, $NaBH_4$ (3.9 g, 104.2 mmol) was added in portions. After completion of addition of $NaBH_4$, the ice bath was removed and the reaction was allowed to proceed at room temperature for 16 h. The reaction was stopped by removal of methanol under reduced pressure. The residue was redissolved in half-saturated aqu. sodium carbonate solution and extracted with CH_2Cl_2 (3 times 30 mL). The combined organic layers were dried over Na_2SO_4 and CH_2Cl_2 was removed under reduced pressure. Purification was carried out using silica gel chromatography (CH_2Cl_2 : MeOH : NH_3OH = 98:1:1). Yield: 3.6 g (82%).

TLC (CH_2Cl_2 : MeOH : NH_3OH = 90:9:1): R_f = 0.44.

^{1}H-NMR (300 MHz, $CDCl_3$) [ppm]: 7.47 – 7.30 (m, 5H, Ar), 7.06 (t, *J* = 7.9, 1H, Ar), 6.80 (d, *J* = 8.0, 1H, Ar), 6.67 (d, *J* = 7.5, 1H, Ar), 6.59 (s, 1H, Ar), 5.71 (s, 1H, Ar), 5.08 (s, 2H, O-C*H*$_2$-Ar), 3.86 (s, 3H, O-C*H*$_3$), 3.77 – 3.72 (m, 1H, N-C*H*), 3.40 (s, 3H, O-C*H*$_3$-Ar), 3.36 – 3.22 (m, 2H, CH_2), 3.01 – 2.81 (m, 3H, CH_2), 2.70 – 2.63 (m, 1H, CH_2), 2.60 (s, 3H, N-CH_3), 2.08 (s, 3H, Ar-C*H*$_3$).
^{13}C-NMR (75 MHz, $CDCl_3$) [ppm]: 156.9, 147.3, 146.0, 139.6, 137.5, 128.6, 128.5, 127.7, 127.1, 126.2, 125.7, 125.3, 123.7, 111.3, 111.1, 109.6, 70.2, 63.6, 55.8, 55.2, 46.0, 42.4, 38.2, 25.1, 11.7

HRMS calcd for $C_{27}H_{31}NO_3$ (M^+–H): 416.2226; found: 416.2217; (M^+–2H): 415.2148; found: 415.2155.

3-((6,7-Dimethoxy-2-methyl-1,2,3,4-tetrahydroisoquinolin-1-yl)methyl)-2-methylphenol

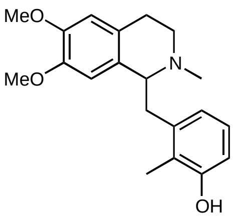

Deprotection of 1-(3-(benzyloxy)-2-methylbenzyl)-6,7-dimethoxy-2-methyl-1,2,3,4-tetrahydroisoquinoline was carried out using palladium on charcoal under hydrogen atmosphere. Thesubstrate (3.6 g, 8.6 mmol) was dissolved in dry methanol (100 mL) and acetic acid (0.86 mL). Palladium on charcoal (0.50 g) was added and hydrogen (1 atm) was applied after evacuation.

After 16 h of reaction time the mixture was filtered through celite and methanol was removed under reduced pressure. The residue was dissolved with dichloromethane and washed with half-saturated aqu. sodium bicarbonate solution and the organic phased were dried over Na_2SO_4. The solvent was again removed under reduced pressure. No further purification was necessary. Yield: 2.5 (90%).

mp: 64–66 °C

TLC (CH_2Cl_2 : MeOH : NH_3OH = 90:9:1): R_f = 0.34.

^{1}H-NMR (300 MHz, $CDCl_3$) [ppm]: 6.96-6.89 (m, 1H, Ar), 6.72 (d, 1H, J=7.9 Hz, Ar), 6.57-6.54 (m, 2H, Ar), 5.67 (s, 1H, Ar), 3.82 (s, 3H, O-CH_3), 3.72 (dd, 1H, J_1=9.8 Hz, J_2=4.5 Hz, N-CH-Ar), 3.40 (s, 3H, O-CH_3), 3.35-3.29 (m, 1H, CH_2), 3.22-3.18 (m, 1H, CH_2), 3.01-2.84 (m, 1H, CH_2), 2.81 (dd, 1H, J_1=13.0 Hz, J_2=9.8 Hz, CH_2), 2.70-2.62 (m, 1H, CH_2), 2.57 (s, 3H, N-CH_3).
^{13}C-NMR (75 MHz, $CDCl_3$) [ppm]: 154.8, 147.3, 145.9, 139.3, 128.4, 125.9, 125.0, 123.6, 122.7, 113.0, 111.4, 111.1, 63.6, 55.7, 55.2, 45.7, 42.1, 38.1, 24.7, 11.4.

HRMS calcd for $C_{20}H_{25}NO_3$ (M^+–H): 326.1756; found: 326.1748.

4.7. Synthesis of 1n:

3-((6,7-Dimethoxy-2-methyl-1,2,3,4-tetrahydroisoquinolin-1-yl)methyl)-2-fluorophenol

2,2,2-Trichloro-1-(2-fluoro-3-methoxyphenyl)ethanol[8]

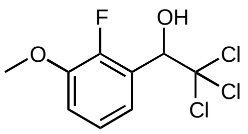

2-Fluoro-3-methoxybenzaldehyde (5.0g, 32.4 mmol) was converted to 2,2,2-trichloro-1-(2-fluoro-3-methoxyphenyl)ethanol using chloroform (6 mL), DMF (20 mL) and KOH (1.5 g, 26.7 mmol) in 5 mL of methanol. 2-Fluoro-3-methoxybenzaldehyde was dissolved in DMF and chloroform and the solution was cooled to approx. –10 °C using a salt-ice bath. KOH in methanol was added dropwise under argon atmosphere and cooling was continued for additional 2 h before quenching the mixture still under cooling with concentrated aqu. HCl solution to a pH of 1. After stirring for additional 30 min at –10 °C the reaction was allowed to warm to ambient temperature and phase separation was performed. The aqueous phase was extracted with toluene (3 times 30 mL). The combined organic layers were washed with water (30 mL) and brine (30 mL) and the solvent was removed under reduced pressure. No further purification steps were necessary. Yield: 8.3 g (93%).

TLC (hexanes : ethyl acetate (+ 1 drop of acetic acid) = 3:1): R_f = 0.23.

^{1}H-NMR (300 MHz, $CDCl_3$) [ppm]: 7.36-7.28 (1H, m, Ar), 7.06 (1H, t, J = 8.1 Hz, Ar), 6.93 (1H, t, J = 8.0 Hz, Ar), 5.59 (1H, s, Ar-C*H*), 3.83 (3H, s, O-C*H*$_3$).
^{13}C-NMR (75 MHz, $CDCl_3$) [ppm]: 152.0, 150.3 (d, J_{CF} = 248.1 Hz), 147.1 (d, J_{CF} = 11.3 Hz), 125.1 (d, J_{CF} = 9.8 Hz), 123.4 (d, J_{CF} = 4.6 Hz), 120.8 (d, J_{CF} = 1.7 Hz), 113.6 (d, J_{CF} = 1.9 Hz), 102.9, 56.2.

2-(2-Fluoro-3-methoxyphenyl)acetic acid

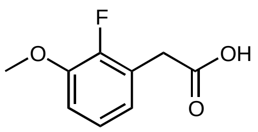

For the synthesis of 2-(2-fluoro-3-methoxyphenyl)acetic acid, oxygen-free ethanol was needed, therefore 60 mL of ethanol were purged with argon for 60 min. After purging, diphenyl diselenide (9.9 g, 31.8 mmol) was added and solubilized followed by the addition of $NaBH_4$ (2.4 g, 63.5 mmol) in portions under constant argon flow. After completed addition, the previously orange solution turned colorless and this solution was stirred for 30 min at ambient temperature. After 30 min the substrate **2** (8.3 g, 30.2 mmol) was added and dissolved followed by the addition of NaOH (7.3 g, 181.4 mmol). The reaction was heated up to 40 °C and was allowed to react over night.
After monitoring *via* TLC and when all starting material was consumed, the reaction was stopped by removal of solvent under reduced pressure. The remaining solid was dissolved in water (30 mL) and extracted under basic conditions with ethyl acetate (30 mL). After this extraction the aqueous phase was adjusted to pH 1 under cooling with concentrated aqu. HCl solution. Then extraction with ethyl acetate (5 times 30 mL) was performed and the combined organic layers were dried over Na_2SO_4. Ethyl acetate was removed under reduced pressure and the product was purified by silica gel column chromatography using a gradient of hexanes (to remove the non-reacted diphenyl diselenide and apolar by-products) and hexanes : acetone = 1:1 to elute the product. Yield: 2.8 g (50%).

mp: 106–108 °C
TLC (hexanes : ethyl acetate (1 trop of acetic acid) = 3:1): *R*f = 0.21.

^{1}H-NMR (300 MHz, $CDCl_3$) [ppm]: 6.89–7.01 (3H, m, Ar), 3.87 (3H, s, O-CH_3), 3.69 (2H, s, CH_2-COOH).
^{13}C-NMR (75 MHz, $CDCl_3$) [ppm]: 170.9, 150.8 (d, J_{CF} = 244.6 Hz), 147.8 (d, J_{CF} = 10.8 Hz), 123.8 (d, J_{CF} = 4.8 Hz), 123.0 (d, J_{CF} = 13.4 Hz), 122.7 (d, J_{CF} = 3.1 Hz), 112.3 (d, J_{CF} = 1.8 Hz), 55.5 (d, J_{CF} = 2.7 Hz), 33.6 (d, J_{CF} = 3.8 Hz).

2-(2-Fluoro-3-hydroxyphenyl)acetic acid

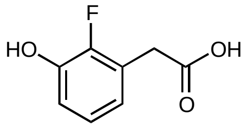

2-(2-Fluoro-3-methoxyphenyl)acetic acid (2.8 g, 15.2 mmol) was dissolved in bromic acid (48% in water, 30 mL) and heated up to 105 °C using an oil bath. After a reaction time of 5 h the reaction was allowed to cool down to room temperature and extracted with ethyl acetate (3 times 30 mL). The combined organic layers were dried over Na_2SO_4 and concentrated under reduced pressure. No further purification was necessary. Yield: 2.2 g (86%).

mp: 110–112 °C

TLC (hexanes : ethyl acetate (+ 1 drop of acetic acid) = 3:1): R_f = 0.13.

^{1}H-NMR (300 MHz, $CDCl_3$) [ppm]: 6.70-6.95 (3H, m, Ar), 3.64 (2H, d, J_{HF} = 1.5 Hz, CH_2-COOH).
^{13}C-NMR (75 MHz, $CDCl_3$) [ppm]: 173.2, 150.2 (d, J_{CF} = 240.3 Hz), 144.8 (d, J_{CF} = 13.2 Hz), 123.6 (d, J_{CF} = 4.5 Hz), 122.7 (d, J_{CF} = 13.7 Hz), 121.2 (d, J_{CF} = 2.7 Hz), 116.3 (d, J_{CF} = 2.8 Hz), 33.7 (d, J_{CF} = 3.8 Hz).

2-(3-(Benzyloxy)-2-fluorophenyl)acetic acid

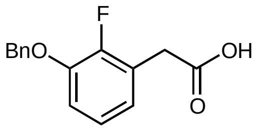

The benzylation of 2-(2-fluoro-3-hydroxyphenyl)acetic acid was carried out using 2.2 g (12.9 mmol) of substrate. The substrate was dissolved in 50 mL of ethanol and KOH (1.7 g, 30.8 mmol), benzyl bromide (2.6 g, 16.5 mmol) and NaI (0.1 g, 0.4 mmol) were added. This reaction mixture was allowed to react at room temperature over night. After reaction control *via* TLC, the reaction was stopped by filtration through celite and the removal of ethanol under reduced pressure. The residue was redissolved in water (20 mL) and acidified with acetic acid. The aqueous phase was extracted with ethyl acetate (3 times 30 mL) and the combined organic layers were dried over Na_2SO_4 followed by the removal of the solvents under reduced pressure. Purification was carried out using silica gel column chromatography with hexanes : ethyl acetate = 3:1 as eluent system. Yield after purification: 2.8 g (83%).

mp: 95–96 °C

TLC (hexanes : ethyl acetate (+ 1 drop of acetic acid) = 3:1): R_f = 0.25.

^{1}H-NMR (300 MHz, $CDCl_3$) [ppm]: 7.29–7.46 (5H, m, Ar), 6.84–7.08 (3H, m, Ar), 5.14 (2H, s, CH_2-O), 3.66 (2H, d, J_{HF} = 1.6 Hz, CH_2-COOH).

^{13}C-NMR (75 MHz, $CDCl_3$) [ppm]: 173.0, 151.3 (d, J_{CF} = 245.2 Hz), 146.7 (d, J_{CF} = 11.0 Hz), 136.9, 128.1, 127.6, 127.2, 123.4 (d, J_{CF} = 4.8 Hz), 122.9 (d, J_{CF} = 3.1 Hz), 114.3 (d, J_{CF} = 1.7 Hz), 70.8, 33.6 (d, J_{CF} = 3.9 Hz).

HRMS calcd for $C_{15}H_{13}FO_3$: 260.0849; found: 260.0879.

2-(3-(Benzyloxy)-2-fluorophenyl)acetyl chloride

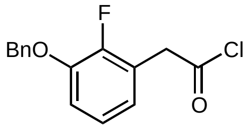

For the synthesis of 2-(3-(benzyloxy)-2-fluorophenyl)acetyl chloride (0.91 g, 3.3 mmol) of 2-(3-(benzyloxy)-2-fluorophenyl)acetic acid was dissolved in try toluene (20 mL) and oxalyl chloride (0.7 g, 5.4 mmol) and one drop of DMF was added. The reaction was allowed to proceed for 3 hours and was stopped by removing toluene and remaining oxalyl chloride under reduced pressure. The product 2-(3-(benzyloxy)-2-fluorophenyl)acetyl chloride was directly used for the next step without further purification.

2-(3-(Benzyloxy)-2-fluorophenyl)-*N*-(3,4-dimethoxyphenethyl)-*N*-methylacetamide

2-(3-(Benzyloxy)-2-fluorophenyl)-*N*-(3,4-dimethoxyphenethyl)-*N*-methylacetamide was synthesized by the use

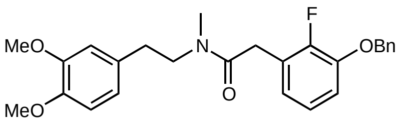

of 2-(3-(benzyloxy)-2-fluorophenyl)acetyl chloride (0.95 g, 3.3 mmol) and 2-(3,4-dimethoxyphenyl)-*N*-methylethanamine (0.7 g, 3.3 mmol). 2-

(3,4-Dimethoxyphenyl)-*N*-methylethanamine was dissolved in chloroform (10 mL) and aqu. NaOH solution (3%, 10 mL) was added. This mixture was cooled using an ice bath and 2-(3-(benzyloxy)-2-fluorophenyl)acetyl chloride, dissolved in chloroform (10 mL), was added dropwise under cooling. After complete addition of 2-(3-(benzyloxy)-2-fluorophenyl)acetyl chloride, the ice bath was removed and the reaction was allowed to proceed at room temperature for 16 hours. For work-up the phases were separated and the aqueous phase was extracted with chloroform (3 times 10 mL). The combined organic layers were washed with 1 M aqu. HCl solution (10 mL) and dried over Na_2SO_4. Chloroform was removed under reduced pressure and the crude product (1.2 g) was purified using silica gel chromatography (hexanes : ethyl acetate = 1:1). Yield: 1.0 g (70%).

mp: 104–106 °C

TLC (hexanes : ethyl acetate = 1:1): R_f = 0.22.

NMR spectroscopy reveals that the product is a mixture of isomers (ratio *trans*/*cis* = 1.09/1). Based on the peak intensities as well as the DEPT, COSY and HSQC spectra, the NMR signals were assigned to the isomers as follows:

^{1}H-NMR (300 MHz, $CDCl_3$) [ppm]:
trans: 7.39-7.24 (m, 6H, Ar), 6.91-6.59 (m, 6H, Ar), 5.05 (s, 2H, Ar-CH_2-O), 3.78 (s, 3H, O-CH_3), 3.77 (s, 3H, O-CH_3), 3.62 (s, 2H, CO-C*H_2*-Ar), 3.52 (t, 2H, *J*=7.5 Hz, Ar-CH_2-C*H_2*-N), 2.86 (s, 3H, N-CH_3), 2.72 (t, 2H, *J*=7.5 Hz, Ar-C*H_2*-CH_2-N).
cis: 7.39-7.24 (m, 5H, Ar), 6.91-6.59 (m, 7H, Ar), 5.02 (s, 2H, Ar-CH_2-O), 3.77 (s, 3H, O-CH_3), 3.76 (s, 3H, O-CH_3), 3.46-3.41 (m, 4H, Ar-CH_2-C*H_2*-N, CO-C*H_2*-Ar), 2.91 (s, 3H, N-CH_3), 2.66 (t, 2H, *J*=7.3 Hz, Ar-C*H_2*-CH_2-N),
^{13}C-NMR (75 MHz, $CDCl_3$) [ppm]:
trans: 169.8. 150.8 (d, J_{CF}=245.1 Hz), 148.9, 147.5, 146.7, 136.6, 131.6, 128.6, 128.1, 127.4, 123.9 (d, J_{CF}=3.0 Hz), 123.6 (d, J_{CF}=8.0 Hz), 122.6 (d, J_{CF}=3.0 Hz), 120.7, 114.0 (d, J_{CF}=1.7 Hz), 112.0, 111.2, 71.3, 55.9, 50.3, 36.4, 33.7, 33.2.
cis: 170.1, 150.6 (d, J_{CF}=244.1 Hz), 149.1, 147.9, 146.8, 136.5, 130.5, 128.6, 128.1, 127.4, 123.8 (d, J_{CF}=3.0 Hz), 123.4 (d, J_{CF}=8.2 Hz), 122.5 (d, J_{CF}=2.9 Hz), 120.8, 113.9 (d, J_{CF}=1.7 Hz), 111.8, 111.4, 71.3, 55.9, 52.1, 34.4, 33.8, 32.9.

HRMS calcd for $C_{26}H_{28}FNO_4$: 437.2002; found: 437.2028.

1-(3-(Benzyloxy)-2-fluorobenzyl)-6,7-dimethoxy-2-methyl-1,2,3,4-tetrahydroisoquinoline

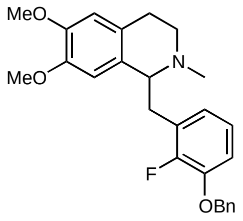

For the cyclization of 2-(3-(benzyloxy)-2-fluorophenyl)-*N*-(3,4-dimethoxyphenethyl)-*N*-methylacetamide, the following procedure was used. Employing dried glassware, 2-(3-(Benzyloxy)-2-fluorophenyl)-*N*-(3,4-dimethoxyphenethyl)-*N*-methylacetamide (1.0 g, 2.3 mmol) was dissolved in dry acetonitrile (25 mL) and phosphoryl chloride (1.1 g, 7.2 mmol) was added under constant argon flow. The reaction was heated to reflux for 3 h and stopped by removing the solvent under reduced pressure. The resulting residue was used for the next step without further purification. In the next step the residue was dissolved in dry methanol (25 mL) and the solution was cooled using an ice bath. Under cooling and a constant flow of argon, $NaBH_4$ (0.82 g, 22.2 mmol) was added in portions. After completion of addition of $NaBH_4$, the ice bath was removed and the reaction was allowed to proceed at room temperature for 16 h. The reaction was stopped by removal of methanol under reduced pressure. The residue was redissolved in half-saturated aqu. sodium carbonate solution (10 mL) and extracted with CH_2Cl_2 (3 times 10 mL). The combined organic layers were dried over Na_2SO_4 and CH_2Cl_2 was removed under reduced pressure. Purification was carried out using silica gel chromatography (CH_2Cl_2 : MeOH : NH_3OH = 98:1:1). Yield: 0.82 g (86%).

TLC (CH_2Cl_2 : MeOH : NH_3OH = 90:9:1): R_f = 0.35.

^{1}H-NMR (300 MHz, $CDCl_3$) [ppm]: 7.46-7.31 (m, 5H, Ar), 6.92-6.84 (m, 2H, Ar), 6.65-6.56 (m, 2H, Ar), 6.56 (s, 1H, Ar), 6.07 (s, 1H, Ar), 5.12 (s, 2H, O-C*H_2*-Ar), 3.83 (s, 3H, O-C*H_3*), 3.81-3.76 (m, 1H, N-C*H*), 3.54 (s, 3H, O-C*H_3*), 3.26-3.17 (m, 2H, N-CH-C*H_2*), 2.90-2.76 (m, 3H, C*H_2*-C*H_2*), 2.64-2.56 (m, 1H, C*H_2*-C*H_2*), 2.52 (s, 3H, N-C*H_3*).

^{13}C-NMR (75 MHz, $CDCl_3$) [ppm]: 151.7 (d, J_{CF} = 244.3), 147.3, 146.6 (d, J_{CF} = 11.3), 146.4, 136.7, 129.3, 128.6, 128.4 (d, J_{CF} = 13.1), 128.1, 127.4, 125.9, 124.1 (d, J_{CF} = 4.1), 123.2 (d, J_{CF} = 4.7), 113.4 (d, J_{CF} = 1.4), 111.2, 110.8, 71.3, 63.2, 55.4, 46.4, 42.6, 34.4, 30.9, 25.4.

HRMS calcd for $C_{26}H_{28}FNO_3$ (M^+–H): 420.1975; found: 420.1992.

3-((6,7-Dimethoxy-2-methyl-1,2,3,4-tetrahydroisoquinolin-1-yl)methyl)-2-fluorophenol

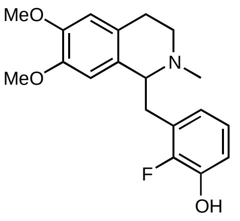

Deprotection of 1-(3-(benzyloxy)-2-fluorobenzyl)-6,7-dimethoxy-2-methyl-1,2,3,4-tetrahydroisoquinoline was carried out using palladium on charcoal under hydrogen atmosphere. Therefore substrate (0.79 g, 2.0 mmol) was dissolved in dry methanol (20 mL) and acetic acid (0.19 mL). Palladium on charcoal (0.15 g) was added and hydrogen (1 atm) was applied after evacuation.
After 16 h of reaction time the mixture was filtered through celite and methanol was removed under reduced pressure. The residue was dissolved in dichloromethane and washed with half-saturated aqu. sodium bicarbonate solution (10 mL) and the organic phase was dried over Na_2SO_4. The solvent was again removed under reduced pressure. No further purification was necessary. Yield: 0.62 g (99%).

mp: 67–69 °C

TLC (CH_2Cl_2 : MeOH : NH_3OH = 90:9:1): R_f = 0.34.

^{1}H-NMR (300 MHz, $CDCl_3$) [ppm]: 8.30 (s, 1H, OH), 6.75-6.66 (m, 2H, Ar), 6.48 (s, 1H, Ar), 6.40-6.35 (m, 1H, Ar), 5.86 (m, 1H, Ar), 3.80 (dd, 1H, J_1=8.6 Hz, J_2=5.1, N-CH-Ar), 3.73 (s, 1H, O-CH_3), 3.41 (s, 1H, O-CH_3), 3.28-3.13 (m, 3H, CH_2), 2.91-2.77 (m, 2H, CH_2), 2.72 (dd, 1H, J_1=13.3 Hz, J_2=8.8, CH_2), 2.61-2.52 (m, 1H, CH_2), 2.45 (s, 3H, N-CH_3).

^{13}C-NMR (75 MHz, $CDCl_3$) [ppm]: 150.7 (d, J_{CF} = 237.9), 147.5, 146.3, 145.1 (d, J_{CF} = 13.8), 128.2, 126.9 (d, J_{CF} = 13.2), 124.8, 123.7 (d, J_{CF} = 3.9), 122.0 (d, J_{CF} = 3.4), 116.4 (d, J_{CF} = 2.4), 111.2, 111.0, 62.9, 55.7, 55.4, 45.4, 41.7, 34.3, 24.3.

HRMS calcd for $C_{19}H_{22}FNO_3$ (M^+–H): 330.1505; found: 330.1521.

4.8. Synthesis of 1o:

1-(2-Fluoro-3-hydroxybenzyl)-6-methoxy-2-methyl-1,2,3,4-tetrahydroisoquinolin-7-ol

2-(2-Fluoro-3-hydroxyphenyl)acetic acid was synthesized as described for **1n**.

2-(2-Fluoro-3-hydroxyphenyl)acetyl chloride

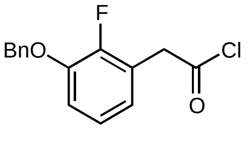

For the synthesis of 2-(3-(benzyloxy)-2-fluorophenyl)acetyl chloride, 2-(2-fluoro-3-hydroxyphenyl)acetic acid (0.82 g, 3.0 mmol) was dissolved in try toluene (20 mL) and oxalyl chloride (0.65 g, 5.1 mmol) and one drop of DMF were added. The reaction was allowed to proceed for 3 h and was stopped by removing toluene and remaining oxalyl chloride under reduced pressure. The obtained 2-(3-(benzyloxy)-2-fluorophenyl)acetyl chloride was directly used for the next step without further purification.

2-(3-(benzyloxy)-2-fluorophenyl)-N-(4-(benzyloxy)-3-methoxyphenethyl)-*N*-methylacetamide

2-(3-(Benzyloxy)-2-fluorophenyl)-*N*-(4-hydroxy-3-methoxyphenethyl)-*N*-methylacetamide was syn-thesized by

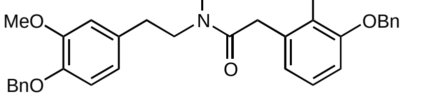

the use of 2-(2-fluoro-3-hydroxyphenyl)acetyl chloride (0.87 g, 3.2 mmol) and previously prepared amino-part 2-(4-(benzyloxy)-3-methoxyphenyl)-*N*-methylethanamine (1.0 g, 3.8 mmol).
2-(4-(benzyloxy)-3-methoxyphenyl)-*N*-methylethanaminewas dissolved in chloroform (10 mL) and aqu. NaOH solution (3%, 10 mL) was added. This mixture was cooled using an ice

bath and 2-(3-(benzyloxy)-2-fluorophenyl)acetyl chloride, dissolved in chloroform (10 mL), was added dropwise under cooling. After complete addition of 2-(3-(benzyloxy)-2-fluorophenyl)acetyl chloride, the ice bath was removed and the reaction was allowed to proceed at room temperature for 16 h. For work-up the phases were separated and the aqueous phase was extracted with chloroform (3 times10 mL). The combined organic layers were washed with 1 M aqu. HCl solution and dried over Na_2SO_4. Chloroform was removed under reduced pressure and the crude product was purified using silica gel chromatography (hexanes : ethyl acetate = 1:1). Yield: 1.3 g (83%)

TLC (hexanes : ethyl acetate = 1:1): R_f = 0.31.

NMR spectroscopy reveals that the product is a mixture of isomers (ratio *trans*/*cis* = 1.08/1). Based on the peak intensities as well as the DEPT, COSY and HSQC spectra, the NMR signals were assigned to the isomers as follows:

^{1}H-NMR (300 MHz, $CDCl_3$) [ppm]:
trans: 7.20-7.37 (10H, m, Ar), 6.70-6-92 (4H, m, Ar), 6.68 (1H, d, *J* = 1.9 Hz, Ar), 6.53 (1H, dd, J_1=8.1 Hz, J_2=2.0 Hz, Ar), 5.02-5.04 (4H, m, O-CH_2-Ar), 3.76 (3H, s, O-CH_3), 3.61 (2H, s, CO-CH_2-Ar), 3.48-3.53 (2H, t, *J*=7.5 Hz, Ar-CH_2-C*H₂*-N), 2.83 (3H, s, N-CH_3), 2.71 (2H, t, *J*=7.5 Hz, Ar-CH_2-CH2-N).
cis: 7.20-7.37 (10H, m, Ar), 6.70-6-92 (4H, m, Ar), 6.61 (1H, d, *J*=2.0 Hz, Ar), 6.53 (1H, dd, J_1=8.1 Hz, J_2=2.0 Hz, Ar), 5.02-5.04 (4H, m, O-CH_2-Ar), 3.78 (3H, s, O-CH_3), 3.40-3.44 (4H, m, overlap of CO-CH_2-Ar and Ar-CH2-CH_2-N), 2.89 (3H, s, N-CH_3), 2.64 (2H, t, J = 7.2Hz, Ar-CH_2-CH_2-N).

^{13}C-NMR ($CDCl_3$, 75 MHz) [ppm]:
trans: 169.8, 150.9 (d, J_{CF} = 244Hz), 149.7, 146.8, 146.7, 137.3, 136.6, 132.3, 128.6, 128.5, 128.1, 127.8, 127.4, 127.3, 123.9, 123.4 (d, J_{CF} = 9.8 Hz), 122.6, 120.7, 114.3, 114.1 (s, J_{CF} = 1.5 Hz), 112.6, 71.3, 71.2, 56.0, 50.3, 36.4, 33.7, 33.3.
cis: 170.0, 150.9 (d, J_{CF} = 244Hz), 149.9, 147.0, 146.7, 137.2, 136.5, 131.3, 128.6, 128.5, 128.1, 127.8, 127.4, 127.3, 123.8, 123.6 (d, J_{CF} = 9.6Hz), 122.6, 120.8, 114.5, 114.0 (d, J_{CF} = 1.0Hz), 112.5, 71.3, 71.2, 56.0, 52.1, 34.4, 33.8, 32.9.

HRMS calcd for $C_{32}H_{32}FNO_4$: 513.2315; found: 513.2346.

7-(benzyloxy)-1-(3-(benzyloxy)-2-fluorobenzyl)-6-methoxy-2-methyl-1,2,3,4-tetrahydroisoquinoline

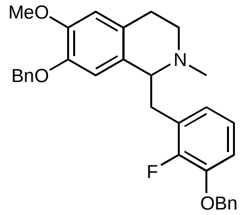

For the cyclization of 7-(benzyloxy)-1-(3-(benzyloxy)-2-fluorobenzyl)-6-methoxy-2-methyl-1,2,3,4-tetrahydroisoquinoline, the following procedure was used. Employing dried glassware, 2-(3-(Benzyloxy)-2-fluorophenyl)-*N*-(4-hydroxy-3-methoxyphenethyl)-*N*-methylacetamide (1.3 g, 2.6 mmol) was dissolved in dry acetonitrile (30 mL) and phosphoryl chloride (1.2 g, 7.7 mmol) was added under constant argon flow. The reaction was heated to reflux for 3 h and stopped by removing the solvent under reduced pressure. The resulting residue was used for the next step without further purification.
In the next step the residue was dissolved in dry methanol (25 mL) and the solution was cooled using an ice bath. Under cooling and a constant flow of argon, $NaBH_4$ (0.95 g, 25.1 mmol) was added in portions. After completion of addition of $NaBH_4$, the ice bath was removed and the reaction was allowed to proceed at room temperature for 16 h.
The reaction was stopped by removal of methanol under reduced pressure. The residue was redissolved in half-saturated aqu. sodium carbonate solution (20 mL) and extracted with CH_2Cl_2 (3 times 10 mL). The combined organic layers were dried over Na_2SO_4 and CH_2Cl_2 was removed under reduced pressure. Purification was carried out using silica gel chromatography (CH_2Cl_2 : MeOH : NH_3OH = 98:1:1); Yield: 0.83 g (65%)

TLC (CH_2Cl_2 : MeOH : NH_3OH = 90:9:1): R_f = 0.45.

^{1}H-NMR (300 MHz, $CDCl_3$) [ppm]: 7.38-7.17 (m, 10H, Ar), 6.82-6.79 (m, 2H, Ar), 6.56-6.51 (m, 2H, Ar), 6.12 (s, 1H, Ar), 5.03 (s, 2H, O-CH_2-Ar), 4.76 (d, *J* = 12.1, 1H, O-CH_2-Ar), 4.70 (d, *J* = 12.1, 1H, O-CH_2-Ar), 3.76 (s, 3H, O-CH_3), 3.68-3.63 (m, 1H, N-C*H*), 3.16- 3.03 (m, 2H, CH_2), 2.83-2.64 (m, 3H, CH_2), 2.59-2.47 (m, 1H, CH_2), 2.42 (s, 3H, N-CH_3).

^{13}C-NMR (75 MHz, $CDCl_3$) [ppm]: 151.68 (d, J_{CF} = 244.6), 148.1, 146.7 (d, J_{CF} = 11.4), 145.8, 137.3, 136.7, 129.4, 128.6, 128.4, 128.4 (d, J_{CF} = 13.0), 128.1, 127.7, 127.4, 127.4, 126.6, 124.1 (d, J_{CF} = 4.1), 123.2 (d, J_{CF} = 4.6), 113.7, 113.4 (d, J_{CF} = 1.4), 111.8, 71.4, 70.9, 63.2, 56.0, 46.5, 42.6, 34.3, 25.8.

HRMS calcd for $C_{32}H_{32}FNO_3$ (M^+–H): 497.2288; found: 496.2289.

1-(2-Fluoro-3-hydroxybenzyl)-6-methoxy-2-methyl-1,2,3,4-tetrahydroisoquinolin-7-ol

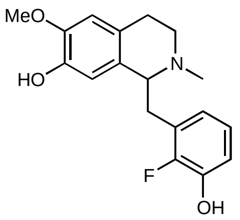

Deprotection of 7-(benzyloxy)-1-(3-(benzyloxy)-2-fluorobenzyl)-6-methoxy-2-methyl-1,2,3,4-tetrahydroisoquinoline was carried out using palladium on charcoal under hydrogen atmosphere. The substrate (0.80 g, 1.6 mmol) was dissolved in dry methanol (20 mL) and acetic acid (0.19 mL). Palladium on charcoal (0.15 g) was added and hydrogen (1 atm) was applied after evacuation.
After 16 h of reaction time the mixture was filtered through celite and methanol was removed under reduced pressure. The residue was dissolved in dichloromethane and washed with half-saturated aqu. sodium bicarbonate solution and the organic phase was dried over Na_2SO_4. The solvent was again removed under reduced pressure. No further purification was necessary. Yield: 0.51 g (98%)

mp: 92–95 °C

TLC (CH_2Cl_2 : MeOH : NH_3OH = 90:9:1): R_f = 0.57.

^{1}H-NMR (300 MHz, $CDCl_3$) [ppm]: 6.75-6.70 (m, 1H, Ar), 6.56-6.45 (m, 3H, Ar), 6.26 (s, 1H, Ar), 3.81-3.79 (m, 1H, N-CH-Ar), 3.77 (s, 3H, O-CH_3), 3.27-3.17 (m, 1H, CH_2), 3.12-3.05 (m, 1H, CH_2), 2.90-2.71 (m, 3H, CH_2), 2.57-2.48 (m, 2H, CH_2), 2.41 (s, 3H, N-CH_3)
^{13}C-NMR (75 MHz, $CDCl_3$) [ppm]: 150.5, (d, J_{CF} = 236.4) 145.4, 144.5 (d, J_{CF} = 14.4), 143.4, 129.6, 126.8 (d, J_{CF} = 13.3), 124.6, 123.8 (d, J_{CF} = 4.3), 121.9 (d, J_{CF} = 3.2), 116.1 (d, J_{CF} = 1.4), 113.7, 110.7, 63.0, 55.8, 45.5, 41.6, 34.3, 24.2.

HRMS calcd for $C_{18}H_{20}FNO_3$ (M^+–H): 316.1349; found: 316.1375.

4.9. Synthesis of 1p:

1-(2-Fluoro-3-hydroxybenzyl)-7-methoxy-2-methyl-1,2,3,4-tetrahydroisoquinolin-6-ol

2-(2-Fluoro-3-hydroxyphenyl)acetic acid was synthesized as described for **1j**.
2-(3-(Benzyloxy)-4-methoxyphenyl)-*N*-methylethanamine was synthesized as described previously.[1, 2]

2-(3-(Benzyloxy)-2-fluorophenyl)acetyl chloride

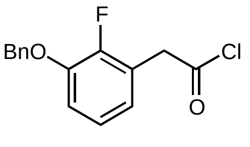

For the synthesis of 2-(3-(benzyloxy)-2-fluorophenyl)acetyl chloride, 2-(2-fluoro-3-hydroxyphenyl)acetic acid (0.82 g, 2.95 mmol) was dissolved in dry toluene (20 mL) and oxalyl chloride (0.65 g, 5.10 mmol) and one drop of DMF were added. The reaction was allowed to prceed for 3 h and was stopped by removing toluene and remaining oxalyl chloride under reduced pressure. The obtained 2-(3-(benzyloxy)-2-fluorophenyl)acetyl chloride was directly used for the next step without further purification.

2-(3-(Benzyloxy)-2-fluorophenyl)-*N*-(3-(benzyloxy)-4-methoxyphenethyl)-*N*-methylacetamide

2-(3-(Benzyloxy)-2-fluorophenyl)-*N*-(4-hydroxy-3-methoxyphenethyl)-*N*-methylacetamide was synthesized by

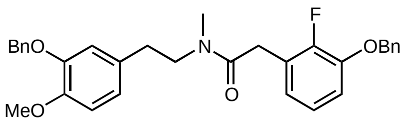

the use of 2-(2-fluoro-3-hydroxyphenyl)acetyl chloride (0.87 g, 3.2 mmol) and previously prepared amino-part 2-(3-(benzyloxy)-4-methoxyphenyl)-*N*-methylethanamine (0.87 g, 3.2 mmol). Substrate 2-(3-(benzyloxy)-4-methoxyphenyl)-*N*-methylethanamine was dissolved in chloroform (10 mL) and aqu. NaOH solution (3%, 10 mL) was added. This mixture was cooled using an ice bath and 2-(3-(benzyloxy)-2-fluorophenyl)acetyl chloride, dissolved in chloroform (10 mL), was added dropwise

under cooling. After complete addition of 2-(3-(benzyloxy)-2-fluorophenyl)acetyl chloride, the ice bath was removed and the reaction was allowed to proceed at room temperature for 16 h. For work-up the phases were separated and the aqueous phase was extracted with chloroform (3 times 10 mL). The combined organic layers were washed with 1 M aqu. HCl solution and dried over Na_2SO_4. Chloroform was removed under reduced pressure and the crude product was purified using silica gel chromatography (hexanes : ethyl acetate = 1:1). Yield: 1.3 g (95%)

TLC (hexanes : ethyl acetate = 1:1): R_f = 0.30.

NMR spectroscopy reveals that the product is a mixture of isomers (ratio *trans*/*cis* = 1.13/1). Based on the peak intensities as well as the DEPT, COSY and HSQC spectra, the NMR signals were assigned to the isomers as follows:

^{1}H-NMR (300 MHz, $CDCl_3$) [ppm]:
trans: 7.20-7.37 (m, 10H, Ar), 6.70-6-92 (m, 4H, Ar), 6.68 (d, 1H, *J*=1.9 Hz, Ar), 6.53 (dd, 1H, J_1=8.1 Hz, J_2=2.0 Hz, Ar), 5.02-5.04 (m, 4H, O-CH_2-Ar), 3.76 (s, 3H, O-CH_3), 3.61 (s, 2H, CO-CH_2-Ar), 3.48-3.53 (t, 2H, *J*=7.5 Hz, Ar-CH_2-C*H*$_2$-N), 2.83 (s, 3H, N-CH_3), 2.66 (t, 2H, *J*=7.5 Hz, Ar-C*H*$_2$-CH_2-N).
cis: 7.20-7.37 (m, 10H, Ar), 6.70-6-92 (m, 4H, Ar), 6.61 (d, 1H, *J* = 2.0Hz, Ar), 6.53 (dd, 1H, J_1=8.1 Hz, J_2=2.0 Hz, Ar), 5.02-5.04 (m, 4H, O-CH_2-Ar), 3.78 (s, 3H, O-CH_3), 3.40-3.44 (m, 4H, overlap of CO-CH_2-Ar and Ar-CH_2-C*H*$_2$-N), 2.89 (s, 3H, N-CH_3), 2.64 (t, 2H, *J*=7.2 Hz, Ar-C*H*$_2$-CH_2-N).

^{13}C-NMR ($CDCl_3$, 75 MHz) [ppm]:
cis: 170.0, 150.9 (d, J_{CF} = 244Hz), 149.9, 147.0, 146.7, 137.2, 136.5, 131.3, 128.6, 128.5, 128.1, 127.8, 127.4, 127.3, 123.8, 123.6 (d, J_{CF} = 9.6Hz), 122.6, 120.8, 114.5, 114.0 (d, J_{CF} = 1.0Hz), 112.5, 71.3, 71.2, 56.0, 52.1, 34.4, 33.8, 32.9.
trans: 169.8, 150.9 (d, J_{CF} = 244Hz), 149.7, 146.8, 146.7, 137.3, 136.6, 132.3, 128.6, 128.5, 128.1, 127.8, 127.4, 127.3, 123.9, 123.4 (d, J_{CF} = 9.8Hz), 122.6, 120.7, 114.3, 114.1 (d, J_{CF} = 1.5Hz), 112.6, 71.3, 71.2, 56.0, 50.3, 36.4, 33.7, 33.3.
HRMS calcd for $C_{32}H_{32}FNO_4$: 513.2315; found: 513.2332.

6-(Benzyloxy)-1-(3-(benzyloxy)-2-fluorobenzyl)-7-methoxy-2-methyl-1,2,3,4-tetrahydroisoquinoline

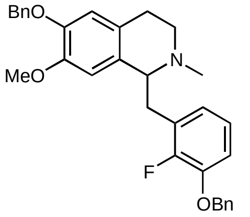

For the cyclization of 2-(3-(benzyloxy)-2-fluorophenyl)-*N*-(3-hydroxy-4-methoxyphenethyl)-*N*-methylacetamide, the following procedure was used. Employing dried glassware, 2-(3-(Benzyloxy)-2-fluorophenyl)-*N*-(3-hydroxy-4-methoxyphenethyl)-*N*-methylacetamide (1.3 g, 2.5 mmol) was dissolved in dry acetonitrile (30 mL) and phosphoryl chloride (1.15 g, 7.51 mmol) was added under constant argon flow. The reaction was heated to reflux for 3 h and stopped by removing solvents under reduced pressure. The resulting residue was used for the next step without further purification.
In the next step the residue was dissolved in dry methanol (25 mL) and the solution was cooled using an ice bath. Under cooling and a constant flow of argon, $NaBH_4$ (0.93 g, 24.5 mmol) was added in portions. After completion of addition of $NaBH_4$, the ice bath was removed and the reaction was allowed to proceed at room temperature for 16 h.
The reaction was stopped by removal of methanol under reduced pressure. The residue was redissolved in half-saturated aqu. sodium carbonate solution and extracted with CH_2Cl_2 (3 times 10 mL). The combined organic layers were dried over Na_2SO_4 and CH_2Cl_2 was removed under reduced pressure. Purification was carried out using silica gel chromatography (CH_2Cl_2 : MeOH : NH_3OH = 98:1:1); Yield: 1.3 g (87%)

TLC (CH_2Cl_2 : MeOH : NH_3OH = 90:9:1): R_f = 0.42.

^{1}H-NMR (300 MHz, $CDCl_3$) [ppm]: 7.49-7.31 (m, 10H, Ar), 6.94-6.86 (m, 2H, Ar), 6.68-6.63 (m, 2H, Ar), 6.12 (s, 1H, Ar), 5.15 (s, 2H, O-CH_2-Ar), 5.11 (s, 2H, O-CH_2-Ar), 3.84-3.81 (m, 1H, N-CH-Ar), 3.57 (s, 3H, O-CH_3), 3.27-3.18 (m, 2H, CH_2), 2.89-2.74 (m, 3H, CH_2), 2.62-2.58 (m, 1H, CH_2), 2.54 (s, 3H, N-CH_3).
^{13}C-NMR (75 MHz, $CDCl_3$) [ppm]: 151.7 (d, J_{CF} = 244.8), 147.2, 146.6, 146.6 (d, J_{CF} = 11.4), 137.3, 136.7, 130.0, 128.6, 128.5, 128.3 (d, J_{CF} = 13.1), 128.1, 127.7, 127.4, 127.3, 125.8, 124.1 (d, J_{CF} = 4.3), 123.2 (d, J_{CF} = 4.6), 114.0, 113.5 (d, J_{CF} = 1.1), 111.4, 71.4, 71.0, 63.2, 55.6, 46.3, 42.6, 34.3, 25.3.
HRMS calcd for $C_{32}H_{32}FNO_3$ (M^+–H): 496.2888; found: 496.2281.

1-(2-Fluoro-3-hydroxybenzyl)-7-methoxy-2-methyl-1,2,3,4-tetrahydroisoquinolin-6-ol

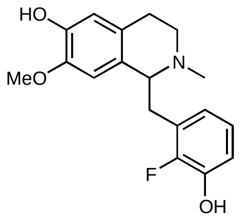

Deprotection of 6-(benzyloxy)-1-(3-(benzyloxy)-2-fluorobenzyl)-7-methoxy-2-methyl-1,2,3,4-tetrahydroisoquinoline was carried out using palladium on charcoal under hydrogen atmosphere. The substrate (1.3 g, 2.7 mmol) was dissolved in dry methanol (20 mL) and acetic acid (0.32 mL). Palladium on charcoal (0.25 g) was added and hydrogen (1 atm) was applied after evacuation.
After 16 h of reaction time the mixture was filtered through celite and methanol was removed under reduced pressure. The residue was dissolved in dichloromethane (10 mL) and washed with half-saturated aqu. sodium bicarbonate solution (5 mL) and the organic phase was dried over Na_2SO_4. The solvent was again removed under reduced pressure. No further purification was necessary. Yield: 0.8 g (99%)

mp: 85–88 °C

TLC (CH_2Cl_2 : MeOH : NH_3OH = 90:9:1): R_f = 0.56.

^{1}H-NMR (300 MHz, DMSO-d6) [ppm]: 9.64 (s, 1H, OH), 8.72 (s, 1H, OH), 6.86-6.74 (m, 2H, Ar), 6.65-6.57 (m, 2H, Ar), 6.46 (s, 1H, Ar), 6.22 (s, 1H, Ar), 3.66 (t, 1H, J = 6.7 Hz, N-CH-Ar), 3.53 (s, 3H, O-CH_3), 3.13-2.97 (m, 2H, CH_2), 2.81-2.71 (m, 1H, CH_2), 2.67-2.59 (m, 2H, CH_2), 2.43-2.41 (m, 1H, CH_2), 3.35 (s, 3H, N-CH_3).

^{13}C-NMR (75 MHz, DMSO-d6) [ppm]: 150.1 (d, J_{CF} = 239.3 Hz), 145.8, 145.1 (d, J_{CF} = 13.0 Hz), 145.0, 128.2 (d, J_{CF} = 13.0 Hz), 126.2, 123.8 (d, J_{CF} = 4.0 Hz), 121.8 (d, J_{CF} = 3.4 Hz), 115.7 (d, J_{CF} = 2.1 Hz), 115.5, 111.7, 63.4, 55.6, 46.1, 42.5, 34.0, 24.4.

HRMS calcd for $C_{18}H_{20}FNO_3$ (M^+–H): 316.1349; found: 316.1393.

5. BIOCATALYTIC PREPARATIVE TRANSFORMATIONS:

Reaction conditions for substrates **1k**:
Substrate **1k** (100 mg, 0.3 mmol, final concentration: 2 g L^{-1} = 6.5 mM) was dissolved in DMSO (5 mL) and buffer (45 mL, Tris-HCl, 10 mM, pH 9, 10 mM $MgCl_2$) containing BBE (429 µL enzyme solution, final concentration: 0.3 g L^{-1} = 0.0051 mM) and catalase (125 mg crude preparation). The mixture was shaken in a light shielded round bottom flask (50 mL) in an Incubator Mini Shaker (VWR, rotary, orbit 3 mm) at 200 rpm and 40 °C for 24 h. The reaction was stopped by phase separation followed by extraction of the aqueous phase employing ethyl acetate (3 × 10 mL). Combined organic phases were dried (Na_2SO_4) and the organic solvents were removed under reduced pressure. The crude product was purified using silica gel chromatography (silica gel 60, 0.040- 0.063mm, Merck, Lot.: 1.09385.9025) yielding 47 mg of (*R*)-**1k** (47% yield, >97% *ee*) and 49 mg (*S*)-**2k** (49% yield, >97% *ee*)

Characterisation:

(*R*)-**1k**:
mp: 121–123 °C

Optical rotation:
$[\alpha]_D^{20}$ = -59.5; *c*=1.55 (g/100 mL); chloroform

HPLC:
column: Chiralcel OJ; Eluent: *n*-heptane/2-propanol = 80/20 + 0.1% TFA (isocratic); 0.50 mL/min; column temperature: 40 °C; detection wavelength: 280 nm
Retention time: 21.8 min

TLC (CH_2Cl_2 : MeOH : NH_3(aq) = 90:9:1): *R*f = 0.33.

NMR:
^{1}H-NMR (300 MHz, $CDCl_3$) [ppm]: 6.71 (t, *J* = 9.2, 1H, Ar), 6. 50 (s, 1H, Ar), 6.42-6.33 (m, 2H, Ar), 6.13 (s, 1H, Ar), 3.81 (m, 1H, N-C*H*-Ar), 3.74 (s, 3H, Ar-O-C*H₃*), 3.56 (s, 3H, Ar-O-C*H*$_3$), 3.25-3.17 (m, 1H, CH_2), 2.93-2.75 (m, 4H, CH_2), 2.61-2.53 (m, 4H, CH_2), 2.41 (s, 3H, N-CH_3)

^{13}C-NMR (75 MHz, $CDCl_3$) [ppm]: 155.2 (d, J_{CF} = 235.5 Hz), 152.5 (d, J_{CF} = 2.0 Hz), 147.6, 146.8, 128.3, 126.7 (d, J_{CF} = 16.7 Hz), 124.9, 118.5 (d, J_{CF} = 4.5 Hz), 115.7 (d, J_{CF} = 24.0 Hz), 114.4 (d, J_{CF} = 8.0 Hz), 111.2, 110.7, 63.3, 55.6, 45.3, 35.0, 29.7, 23.8.

HRMS calcd for $C_{19}H_{22}FNO_3$ (M^+–H): 330.1505; found: 330.1522.

(*S*)-**2k**:
mp: 91–93 °C
Optical rotation:
$[\alpha]_D^{20}$ = -141.3; *c*=1.11 (g/100 mL); chloroform

HPLC:
column: Chiralcel OD-H; Eluent: n-heptane/2-propanol = 70/30 + 0.1% TFA (isocratic); 0.50 mL/min; column temperature: 18 °C; detection wavelength: 280 nm
Retention time: 33.7 min

TLC (CH_2Cl_2 : MeOH : NH_3(aq) = 90:9:1): R_f = 0.51.

NMR:
^{1}H-NMR (300 MHz, $CDCl_3$) [ppm]: 6.69 (s, 1H, Ar), 6.60-6.54 (m, 1H, Ar), 6.31 (dd, 1H, J_1=8.7 Hz, J_2=4.3 Hz, Ar), 4.09 (d, 1H, J=15.8 Hz, N-CH_2-Ar), 3.83 (s, 3H, O-CH_3), 3.80 (s, 3H, O-CH_3), 3.52 (dd, 1H, J_1=11.3 Hz, J_2=3.8 Hz, CH_2), 3.35 (d, 1H, J=15.6 Hz, N-CH_2-Ar), 3.38-3.29 (m, 1H, CH_2), 3.17-3.04 (m, 2H, CH_2), 2.66-2.55 (m, 2H, CH_2).

^{13}C-NMR (75 MHz, $CDCl_3$) [ppm]: 154.8 (d, J_{CF} = 236.1 Hz), 148.0 (d, J_{CF} = 2.2 Hz), 147.7, 147.6, 128.9, 126.5, 123.7 (d, J_{CF} = 4.9 Hz), 122.7 (d, J_{CF} = 19.8 Hz), 113.3 (d, J_{CF} = 8.2 Hz), 112.4 (d, J_{CF} = 23.1 Hz), 111.3, 108.4, 58.5, 56.1, 55.8, 53.7, 51.5, 30.1, 28.7.

HRMS calcd for $C_{19}H_{20}FNO_3$: 329.1427; found: 329.1423; (M^+–H): 328.1349; found: 328.1362.

Reaction conditions for substrates **1n**:
Substrate **1n** (100 mg, 0.3 mmol, final concentration: 2 g L^{-1} = 6.5 mM) was dissolved in DMSO (5 mL) and buffer (45 mL, Tris-HCl, 10 mM, pH 9, 10 mM $MgCl_2$) containing BBE (429 µL enzyme solution, final concentration: 0.3 g L^{-1} = 0.0049 mM) and catalase (125 mg crude preparation). The mixture was shaken in a light shielded round bottom flask (50 mL) in an Incubator Mini Shaker (VWR, rotary, orbit 3 mm) at 200 rpm and 40 °C for 24 h. The reaction was stopped by phase separation followed by extraction of the aqueous phase employing ethyl acetate (3 × 10 mL). Combined organic phases were dried (Na_2SO_4) and the organic solvents were removed under reduced pressure. The crude product was purified using silica gel chromatography (silica gel 60, 0.040- 0.063mm, Merck, Lot.: 1.09385.9025) yielding 49 mg of (*R*)-**1n** (50% yield, >97% *ee*) and 43 mg (*S*)-**2n** (43% yield, >97% *ee*)

Characterization:

(*R*)-**1n**:
mp: 149–151 °C
Optical rotation:
$[\alpha]_D^{20}$ = -59.5; *c*=1.25 (g/100 mL); chloroform
HPLC:
column: Chiralcel OJ; Eluent: *n*-heptane/2-propanol = 80/20 + 0.1% TFA (isocratic); 0.5 mL/min; column temperature: 40 °C; detection wavelength: 280 nm
Retention time: 25.5 min
TLC (CH_2Cl_2 : MeOH : NH_3(aq) = 90:9:1): R_f = 0.34.
NMR:
^{1}H-NMR (300 MHz, $CDCl_3$) [ppm]: 6.79-6.74 (m, 1H, Ar), 6.70-6.64 (m, 1H, Ar), 6.50 (s, 1H, Ar), 6.48-6.43 (m, 1H, Ar), 5.94 (s, 1H, Ar), 3.80-3.78 (m, 1H, N-CH), 3.76 (s, 3H, O-CH_3), 3.47 (s, 3H, O-CH_3), 3.28-3.12 (m, 2H, CH_2), 2.90-2.71 (m, 3H, CH_2), 2.60-2051 (m, 1H, CH_2), 2.46 (s, 3H, N-CH_3).
^{13}C-NMR (75 MHz, $CDCl_3$) [ppm]: 150.4 (d, J_{CF} = 237.0), 147.5, 146.4, 144.4 (d, J_{CF} = 14.5), 128.5, 127.1 (d, J_{CF} = 13.0), 125.1, 123.8 (d, J_{CF} = 4.2), (d, J_{CF} = 3.5), 122.4, 115.9 (d, J_{CF} = 3.8), 111.2, 110.9, 63.1, 55.7, 55.4, 45.6, 41.9, 34.4, 24.5.
HRMS calcd for $C_{19}H_{22}FNO_3$(M^+–H): 330.1505; found: 330.1524.

(*S*)-**3n**:
mp: 207–209 °C
Optical rotation:
$[\alpha]_D^{20}$ = -205.3; *c*=0.62 (g/100 mL); chloroform
HPLC:
column: Chiralcel OD-H; Eluent: n-heptane/2-propanol = 70/30 + 0.1% TFA (isocratic); 0.50 mL/min; column temperature: 18 °C; detection wavelength: 280 nm
Retention time: 30.5 min
TLC (CH_2Cl_2 : MeOH : NH_3(aq) = 90:9:1): R_f = 0.51.
NMR:
^{1}H-NMR (300 MHz, $CDCl_3$) [ppm]: 6.72-6.63 (m, 3H, Ar), 6.55 (s, 1H, Ar), 3.89 (d, 1H, *J*=14.7 Hz, N-CH_2-Ar), 3.84 (s, 3H, O-CH_3), 3.80 (s, 3H, O-CH_3), 3.57 (d, 1H, *J*=15.8 Hz, N-CH_2-Ar), 3.53-3.49 (m, 1H, CH_2), 3.33 (dd, 1H, J_1=16.8 Hz, J_2=4.2 Hz, CH_2), 3.13-3.02 (m, 2H, CH_2), 2.69-2.51 (m, 2H, CH_2).
^{13}C-NMR (75 MHz, $CDCl_3$) [ppm]: 149.1 (d, J_{CF} = 235.3), 147.6, 147.5, 141.5 (d, J_{CF} = 14.2), 129.1, 127.3 (d, J_{CF} = 3.9), 126.6, 122.4 (d, J_{CF} = 16.0), 121.6 (d, J_{CF} = 3.8), 115.2 (d, J_{CF} = 1.6), 111.3, 108.5, 58.9, 57.8, 56.2, 55.8, 51.4, 30.0.
HRMS calcd for $C_{19}H_{20}FNO_3$: 329.1427; found: 329.1452; (M^+–H): 328.1349; found: 328.1366.

Reaction conditions for substrates **1o**:
Substrate **1o** (100 mg, 0.3 mmol, final concentration: 2 g L^{-1} = 6.5 mM) was dissolved in DMSO (5 mL) and buffer (45 mL, Tris-HCl, 10 mM, pH 9, 10 mM $MgCl_2$) containing BBE (400 µL enzyme solution, final concentration: 0.3 g L^{-1} = 0.0046 mM) and catalase (125 mg crude preparation). The mixture was shaken in a light shielded round bottom flask (50 mL) in an Incubator Mini Shaker (VWR, rotary, orbit 3 mm) at 200 rpm and 40 °C for 24 h. The reaction was stopped by phase separation followed by extraction of the aqueous phase employing ethyl acetate (3 × 10 mL). Combined organic phases were dried (Na_2SO_4) and the organic solvents were removed under reduced pressure. The crude product was purified using silica gel chromatography (silica gel 60, 0.040- 0.063mm, Merck, Lot.: 1.09385.9025) yielding 42 mg of (*R*)-**1o** (42% yield, >97% *ee*) and 32 mg (*S*)-**2o** (32% yield, >97% *ee*)

Characterisation:

(*R*)-**1o**:
mp: 149–151 °C
Optical rotation:
$[\alpha]_D^{20}$ = -59.5; *c*=1.25 (g/100 mL); chloroform
HPLC:
column: Chiracel OJ; Eluent: *n*-heptane/2-propanol = 70/30 + 0.1% TFA (isocratic); 0.50 mL/min; column temperature: 18 °C; detection wavelength: 280 nm
Retention time: 34.8 min
TLC (CH_2Cl_2 : MeOH : NH_3(aq) = 90:9:1): R_f = 0.57.
NMR:
^{1}H-NMR (300 MHz, DMSO-d6) [ppm]: 9.56 (s, 1H, OH), 8.68 (s, 1H, OH), 6.86-6.72 (m, 2H, Ar), 6.63-6.57 (m, 1H, Ar), 6.45 (s, 1H, Ar), 6.23 (s, 1H, Ar), 3.65 (t, 1H, *J*=6.6 Hz, N-CH-Ar), 3.53 (s, 3H, O-CH_3), 3.18-2.97 (m, 2H, CH_2), 2.81-2.58 (m, 3H, CH_2), 2.43-2,40 (m, 1H, CH_2), 2.34 (s, 3H, N-CH_3).
^{13}C-NMR (75 MHz, DMSO-d6) [ppm]: 150.5 (d, J_{CF} = 239.4), 145.8, 145.1, 145.1 (d, J_{CF} = 13.2), 128.3 (d, J_{CF} = 12.7), 126.3, 123.8 (d, J_{CF} = 4.0), 121.8 (d, J_{CF} = 3.4), 115.6, 115.6 (d, J_{CF} = 6.5), 111.7, 63.4, 55.6, 46.1, 42.6, 34.0, 24.4.

HRMS calcd for $C_{18}H_{20}FNO_3$ (M^+–H): 316.1349; found: 316.1371.

(*S*)-**3o**:
mp: 207–209 °C
Optical rotation:
$[\alpha]_D^{20}$ = -205.3; *c*=0.62 (g/100 mL); chloroform
HPLC:
column: Chiralcel OD-H; Eluent: n-heptane/2-propanol = 70/30 + 0.1% TFA (isocratic); 0.50 mL/min; column temperature: 18 °C; detection wavelength: 280 nm
Retention time: 28.3 min
TLC (CH_2Cl_2 : MeOH : NH_3(aq) = 90:9:1): R_f = 0.52.
NMR:
^{1}H-NMR (300 MHz, DMSO-d6) [ppm]: 9.53 (s, 1H, OH), 8.81 (s, 1H, OH), 6.89 (s, 1H, Ar), 6.78-6.71 (m, 2H, Ar), 6.51 (s, 1H, Ar), 3.94-3.86 (m, 1H, N-CH_2-Ar), 3.77 (s, 3H, O-CH_3), 3.51-3.44 (m, 3H, overlay N-CH_2-Ar, CH_2), 3.07-3.02 (m, 1H, CH_2), 2.92-2.81 (m, 1H, CH_2), 2.55-2.36 (m, 2H, CH_2).
^{13}C-NMR (75 MHz, DMSO-d6) [ppm]: 149.1 (d, J_{CF} = 238.9), 146.7, 142.9 (d, J_{CF} = 12.6), 128.4 (d, J_{CF} = 8.2), 127.0, 126.9, 123.1 (d, J_{CF} = 16.6), 121.6 (d, J_{CF} = 3.5), 115.6 (d, J_{CF} = 2.0), 115.5, 110.2, 58.9, 57.5, 56.4, 51.3, 30.3, 28.6.

HRMS calcd for $C_{18}H_{18}FNO_3$: 315.1281; found: 315.1276; (M^+–H): 314.1192; found: 314.1217.

Reaction conditions for substrates **1p**:
Substrate **1p** (20 mg, 0.03 mmol, final concentration: 0.2 g L^{-1} = 0.6 mM) was dissolved in DMSO (5 mL) and buffer (45 mL, Tris-HCl, 10 mM, pH 9, 10 mM $MgCl_2$) containing BBE (450 µL enzyme solution, final

concentration: 0.3 g L^{-1} = 0.0047 mM) and catalase (125 mg crude preparation). The mixture was shaken in a light shielded round bottom flask (50 mL) in an Incubator Mini Shaker (VWR, rotary, orbit 3 mm) at 200 rpm and 40 °C for 24 h. The reaction was stopped by phase separation followed by extraction of the aqueous phase employing ethyl acetate (3 × 10 mL). Combined organic phases were dried (Na_2SO_4) and the organic solvents were removed under reduced pressure. The crude product was purified using silica gel chromatography (silica gel 60, 0.040- 0.063mm, Merck, Lot.: 1.09385.9025) yielding 10 mg of (*R*)-**1p** (50% yield, >97% *ee*) and 8.4 mg (*S*)-**2p** (42% yield, >97% *ee*)

Characterisation:

(*R*)-**1p**:
mp: liquid
Optical rotation:
$[\alpha]_D^{20}$ = -29.4; *c*=0.50 (g/100 mL); chloroform
HPLC:
column: Chiralcel OJ; Eluent: *n*-heptane/2-propanol = 70/30 + 0.1% TFA (isocratic); 0.35 mL/min; column temperature: 18 °C; detection wavelength: 280 nm
Retention time: 31.6 min
TLC (CH_2Cl_2 : MeOH : NH_3(aq) = 90:9:1): R_f = 0.56.
NMR:
^{1}H-NMR (300 MHz, $CDCl_3$) [ppm]: 6.75-6.70 (m, 1H, Ar), 6.56-6.45 (m, 3H, Ar), 6.26 (s, 1H, Ar), 3.81-3.79 (m, 1H, N-CH-Ar), 3.77 (s, 3H, O-CH_3), 3.27-3.17 (m, 1H, CH_2), 3.12-3.05 (m, 1H, CH_2), 2.90-2.71 (m, 3H, CH_2), 2.57-2.48 (m, 2H, CH_2), 2.41 (s, 3H, N-CH_3)
^{13}C-NMR (75 MHz, $CDCl_3$) [ppm]: 150.4 (d, J_{CF} = 236.4), 145.4, 144.4 (d, J_{CF} = 14.4), 143.4, 129.6, 126.8 (d, J_{CF} = 13.3), 124.6, 123.8 (d, J_{CF} = 4.3), 121.9 (d, J_{CF} = 3.2), 116.1 (d, J_{CF} = 1.3), 113.7, 110.7, 63.0, 55.9, 45.5, 41.6, 34.3, 24.2.
HRMS calcd for $C_{18}H_{20}FNO_3$ (M^+–H): 316.1349; found: 316.1370.

(*S*)-**3p**:
mp: 238–240 °C
Optical rotation:
$[\alpha]_D^{20}$ = -120.5; *c*=0.42 (g/100 mL); chloroform
HPLC:
no chiral analytic possible
TLC (CH_2Cl_2 : MeOH : NH_3(aq) = 90:9:1): R_f = 0.43.
NMR:
^{1}H-NMR (300 MHz, DMSO-d6) [ppm]: 9.51 (s, 1H, OH), 8.73 (s, 1H, OH), 6.76-6.73 (m, 3H, Ar), 6.64 (s, 1H, Ar), 3.85 (d, 1H, *J*=14.6 Hz, N-CH_2-Ar), 3.74 (s, 3H, O-CH_3), 3.43 (d, 1H, *J*=14.4 Hz, N-CH_2-Ar), 3.41-3.27 (m, 1H, CH_2), 3.06-2.84 (m, 2H, CH_2), 2.60-2.38 (m, 3H, CH_2).
^{13}C-NMR (75 MHz, DMSO-d6) [ppm]: 149.2 (d, J_{CF} = 238.8), 146.6, 145.1, 142.8 (d, J_{CF} = 12.3), 130.1, 126.8 (d, J_{CF} = 3.2), 125.2, 123.1 (d, J_{CF} = 15.3), 121.6 (d, J_{CF} = 3.9), 115.6 (d, J_{CF} = 2.8), 112.8, 112.3, 58.6, 57.5, 55.9, 51.5, 30.5, 28.9.

6. SPECTRA

Synthesis of **1g**:

Provided Material:

3-Benzyloxy-4-methoxybenzaldehyde
^{1}H-NMR spectrum, ^{13}C-NMR spectrum, MS spectrum

3-Benzyloxy-4-methoxy-*β*-nitrostyrene
^{1}H-NMR spectrum, ^{13}C-NMR spectrum, MS spectrum

3-Benzyloxy-4-methoxyphenethylamine
^{1}H-NMR spectrum, ^{13}C-NMR spectrum, MS spectrum

Ethyl 3-benzyloxy-4-methoxyphenethylcarbamate
^{1}H-NMR spectrum, ^{13}C-NMR spectrum, MS spectrum, HRMS results

3-Benzyloxy-4-methoxy-*N*-methylphenethylamine
^{1}H-NMR spectrum, ^{13}C-NMR spectrum, MS spectrum, HRMS results

***N*-(3-Benzyloxy-4-methoxyphenethyl)-2-(3-benzyloxyphenyl)acetamide**
^{1}H-NMR spectrum, ^{13}C-NMR spectrum, ^{13}C-NMR DEPT135 spectrum, ^{13}C-NMR DEPT90 spectrum, COSY spectrum, HSQC spectrum, MS spectrum, HRMS results

1-(3-Benzyloxybenzyl)-6-benzyloxy-7-methoxy-2-methyl-1,2,3,4-tetrahydroisoquinoline
^{1}H-NMR spectrum, ^{13}C-NMR spectrum, MS spectrum, HRMS results

1-(3-Hydroxybenzyl)-6-hydroxy-7-methoxy-2-methyl-1,2,3,4-tetrahydroisoquinoline
^{1}H-NMR spectrum, ^{13}C-NMR spectrum, MS spectrum, HRMS results

3-Benzyloxy-4-methoxybenzaldehyde

^{1}H-NMR spectrum

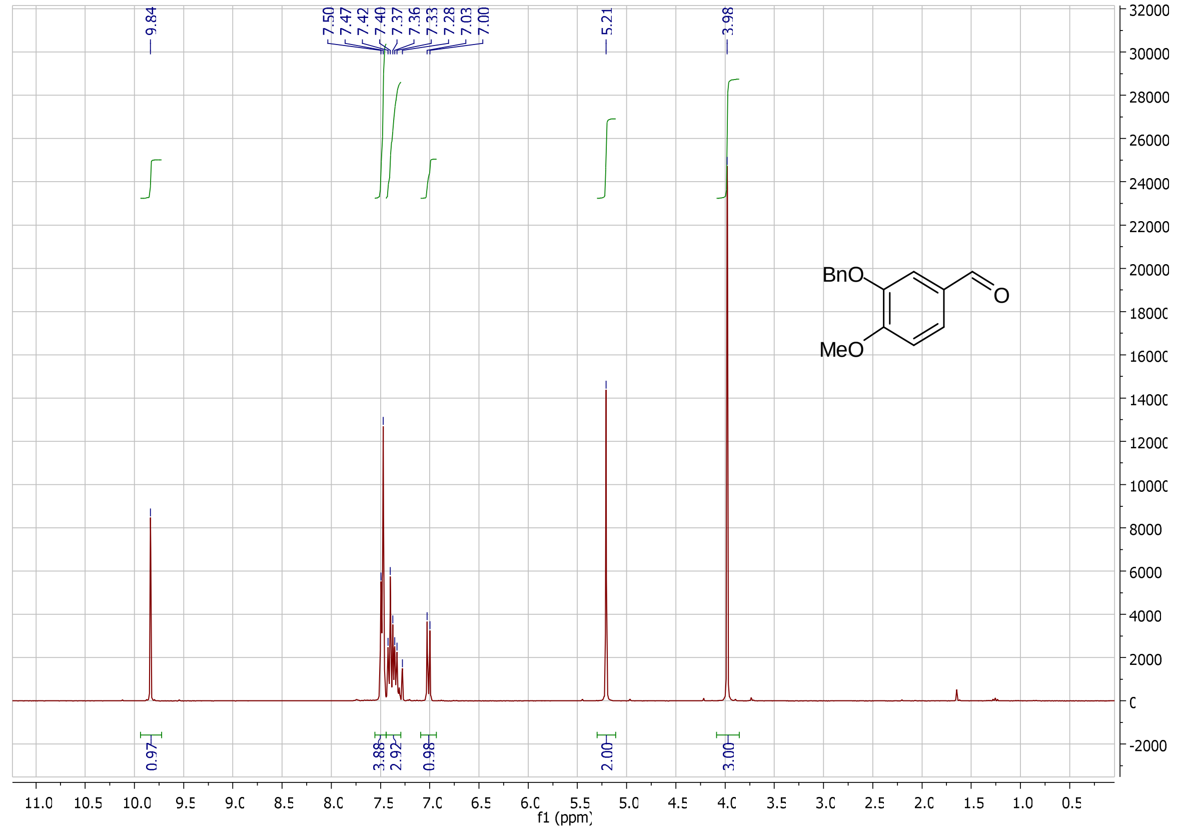

^{13}C-NMR spectrum

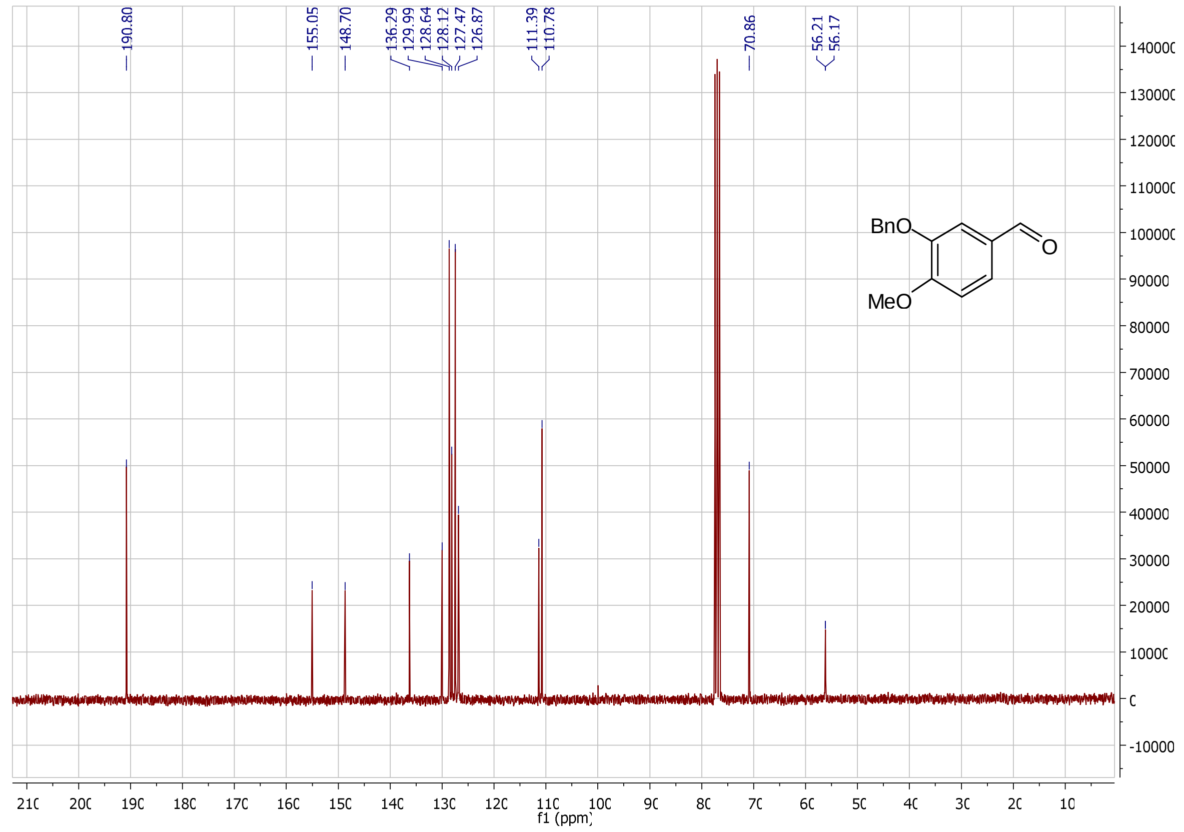

MS spectrum

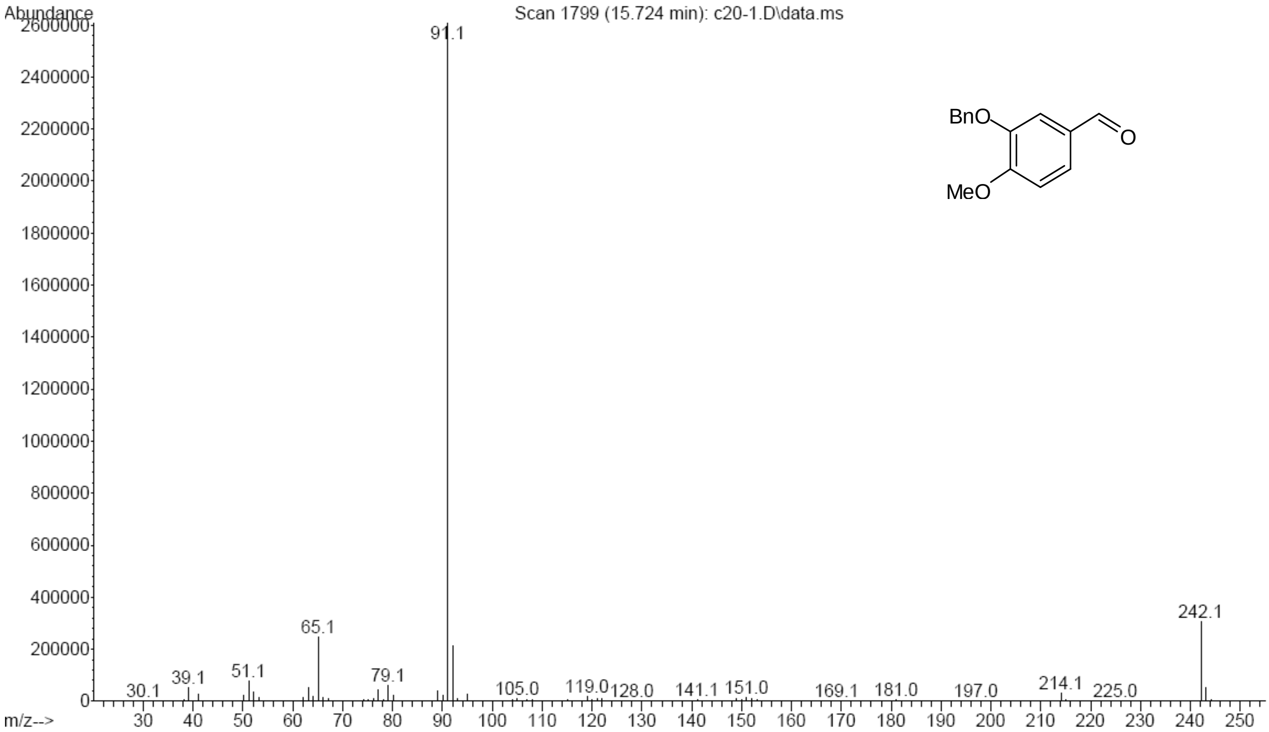
Abundance
Scan 1799 (15.724 min): c20-1.D\data.ms
BnO
MeO
O
91.1
65.1
242.1
30.1
39.1
51.1
79.1
105.0
119.0
128.0
141.1
151.0
169.1
181.0
197.0
214.1
225.0
m/z-->

3-Benzyloxy-4-methoxy-*β*-nitrostyrene

^{1}H-NMR spectrum

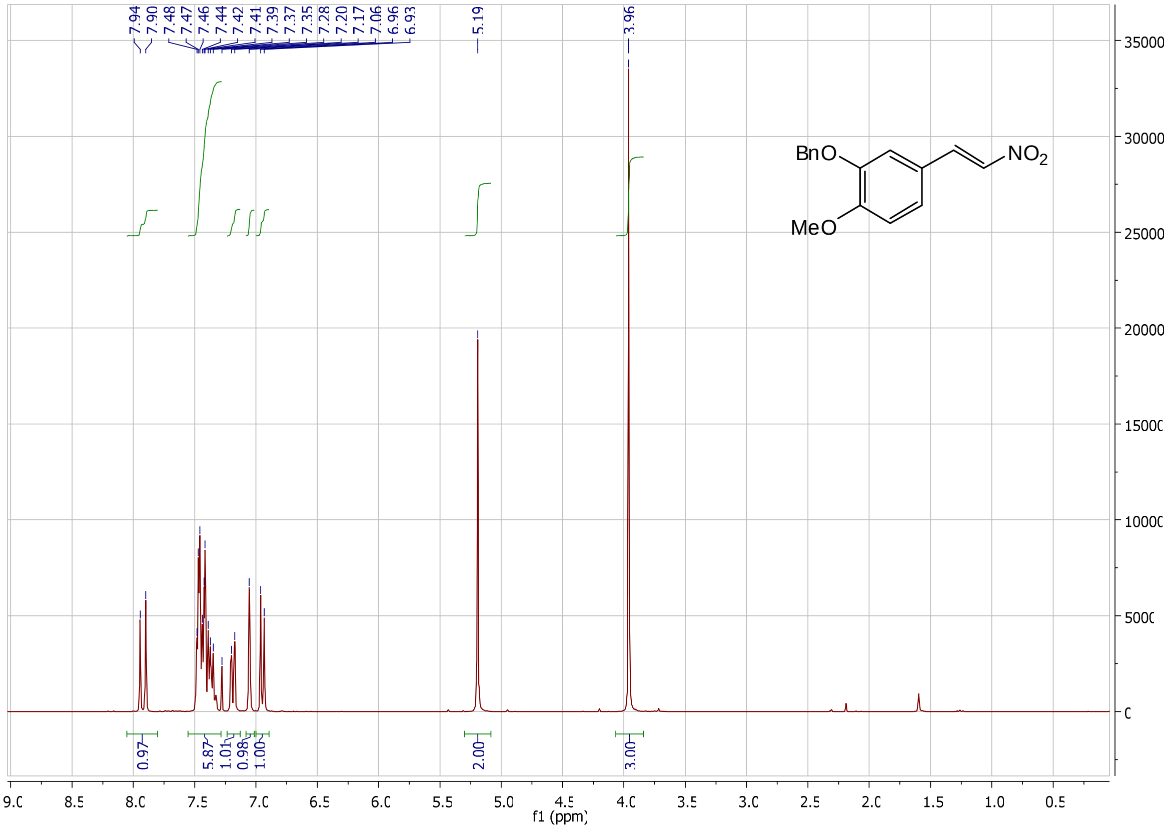

^{13}C-NMR spectrum

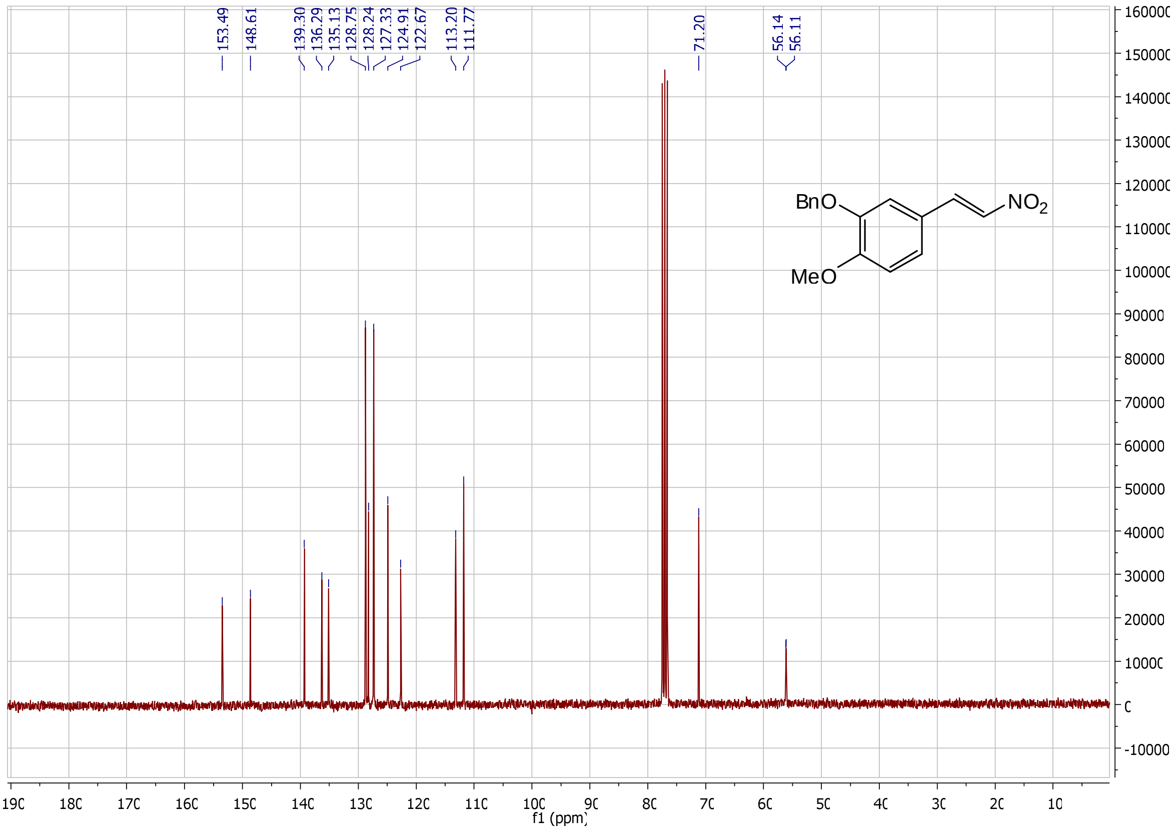

MS spectrum

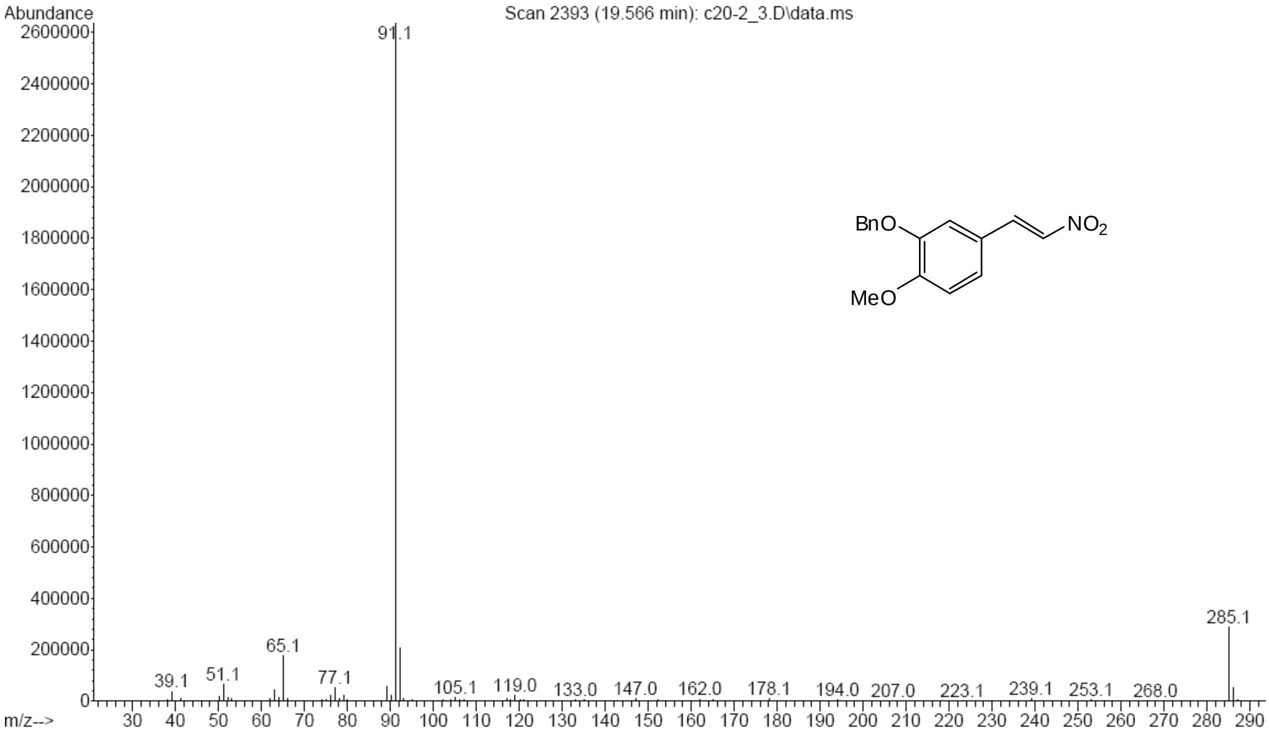
Abundance
Scan 2393 (19.566 min): c20-2_3.D\data.ms
m/z-->
91.1
65.1
39.1
51.1
77.1
105.1
119.0
133.0
147.0
162.0
178.1
194.0
207.0
223.1
239.1
253.1
268.0
285.1
BnO
MeO
NO2

3-Benzyloxy-4-methoxyphenethylamine

^{1}H-NMR spectrum

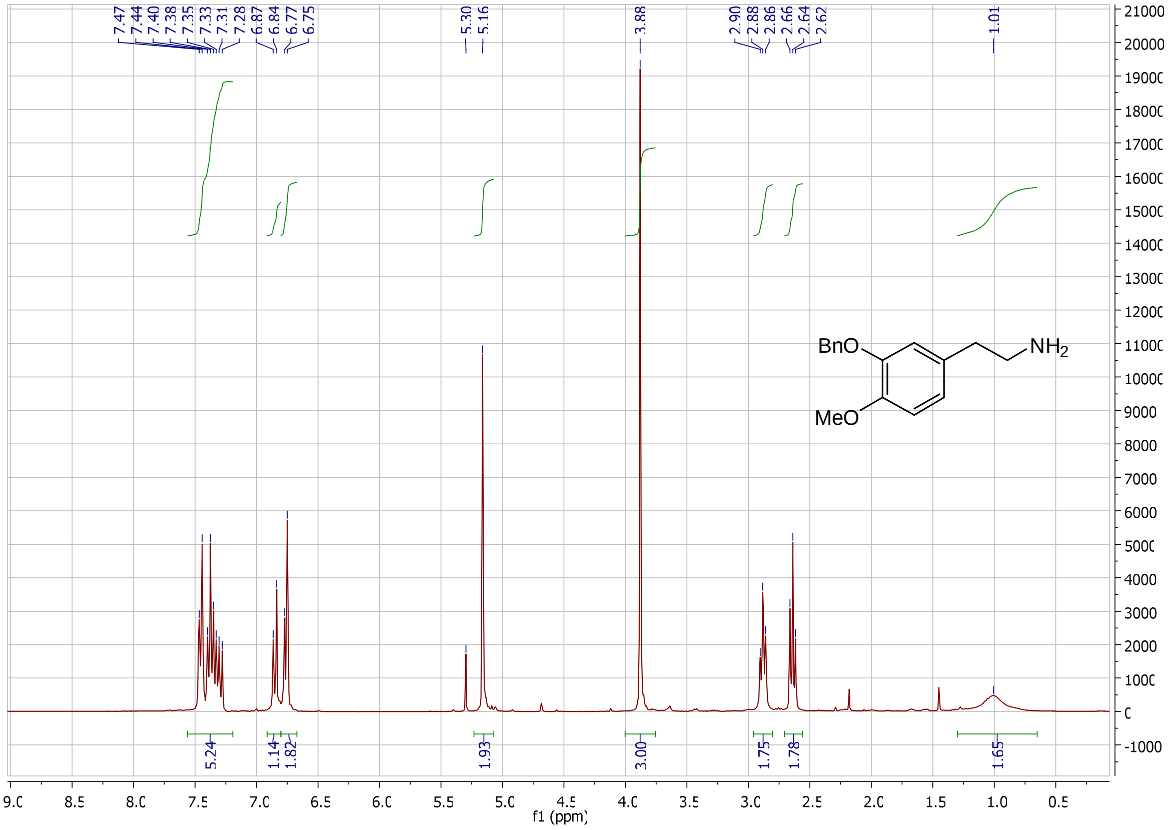

^{13}C-NMR spectrum

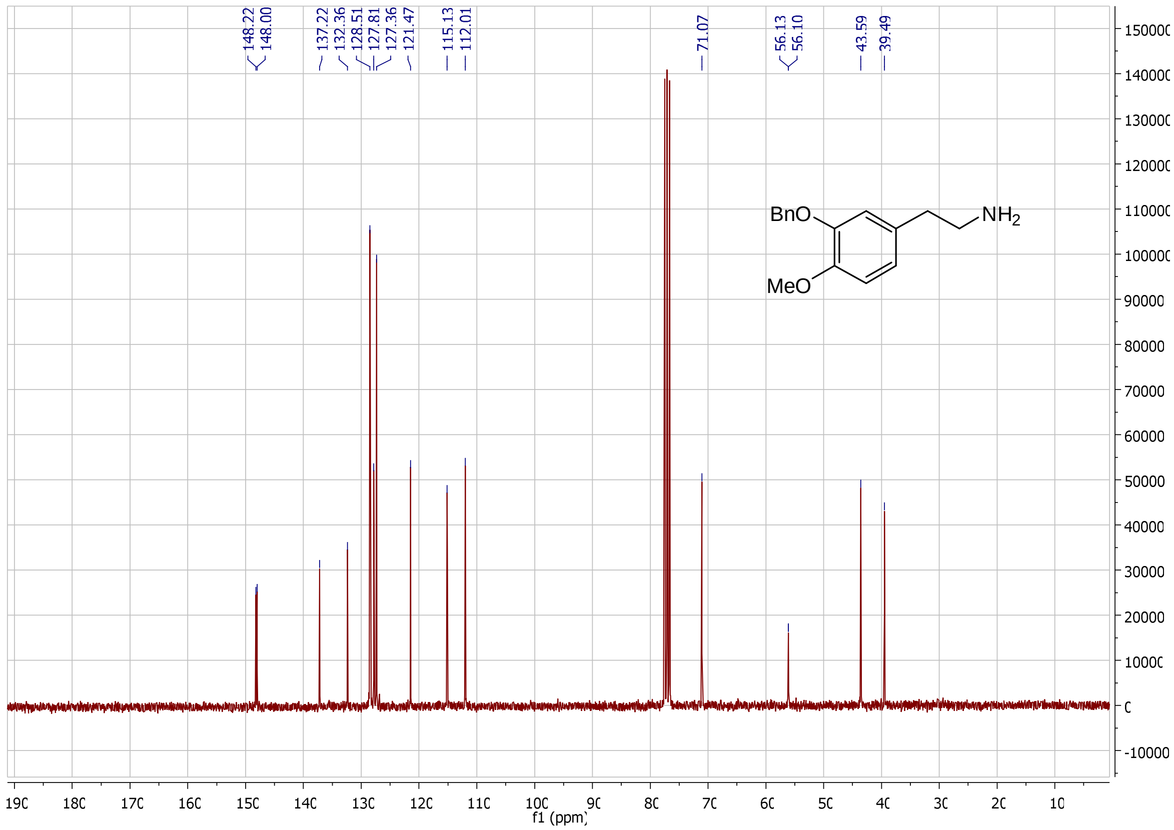

MS spectrum

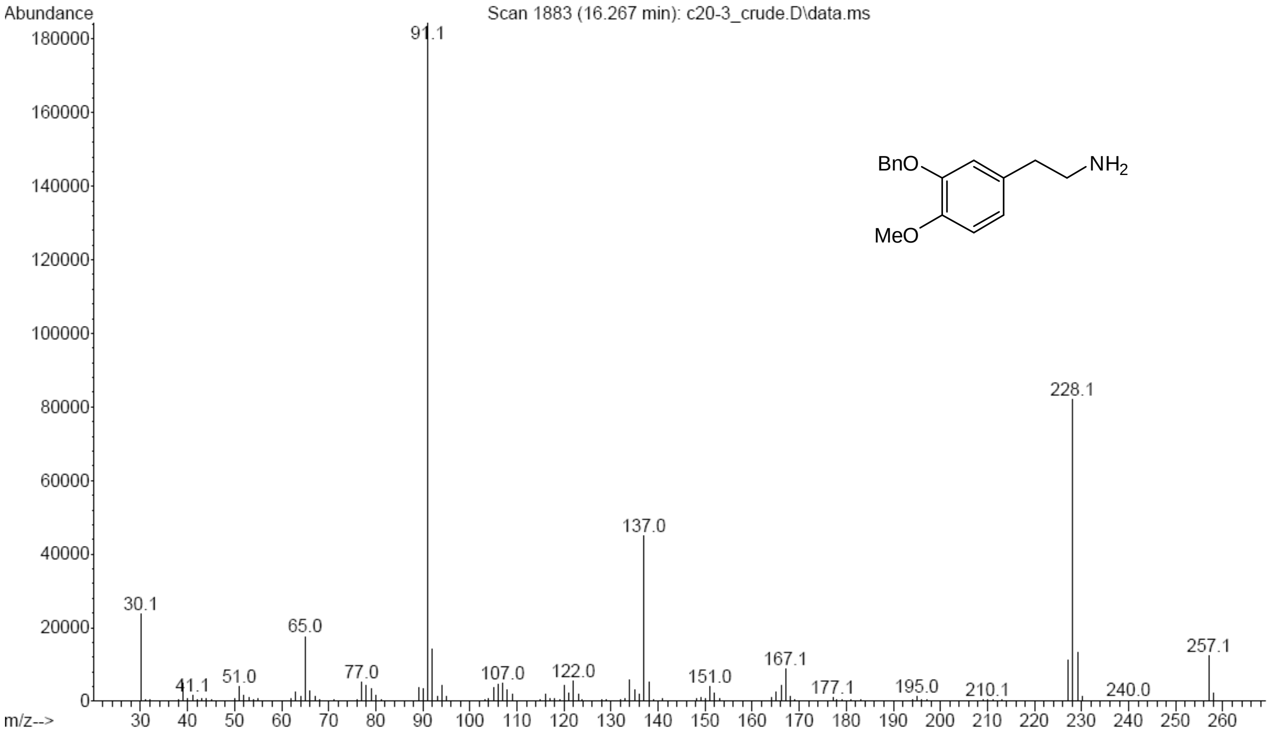
Abundance
Scan 1883 (16.267 min): c20-3_crude.D\data.ms
180000
160000
140000
120000
100000
80000
60000
40000
20000
0
91.1
228.1
137.0
30.1
65.0
257.1
41.1
51.0
77.0
107.0
122.0
151.0
167.1
177.1
195.0
210.1
240.0
m/z-->
30
40
50
60
70
80
90
100
110
120
130
140
150
160
170
180
190
200
210
220
230
240
250
260
BnO
MeO
NH2

Ethyl 3-benzyloxy-4-methoxyphenethylcarbamate

^{1}H-NMR spectrum

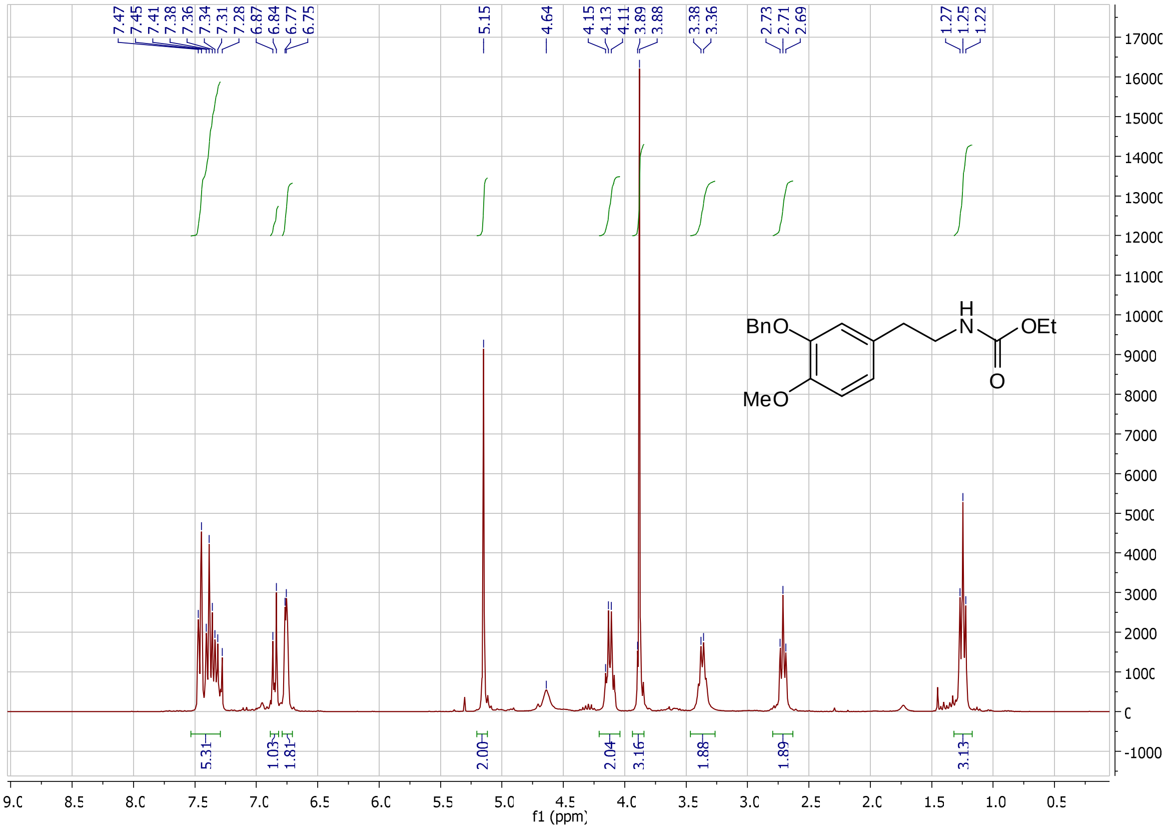

^{13}C-NMR spectrum

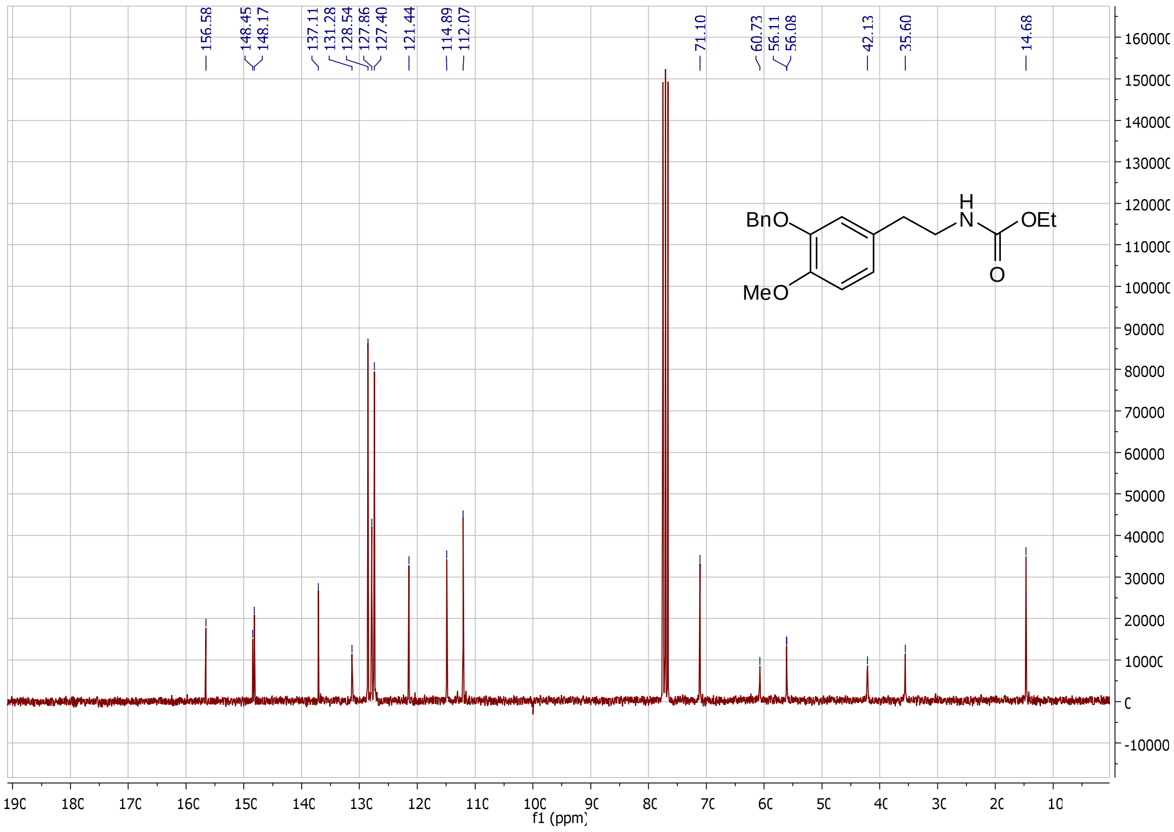

HRMS results

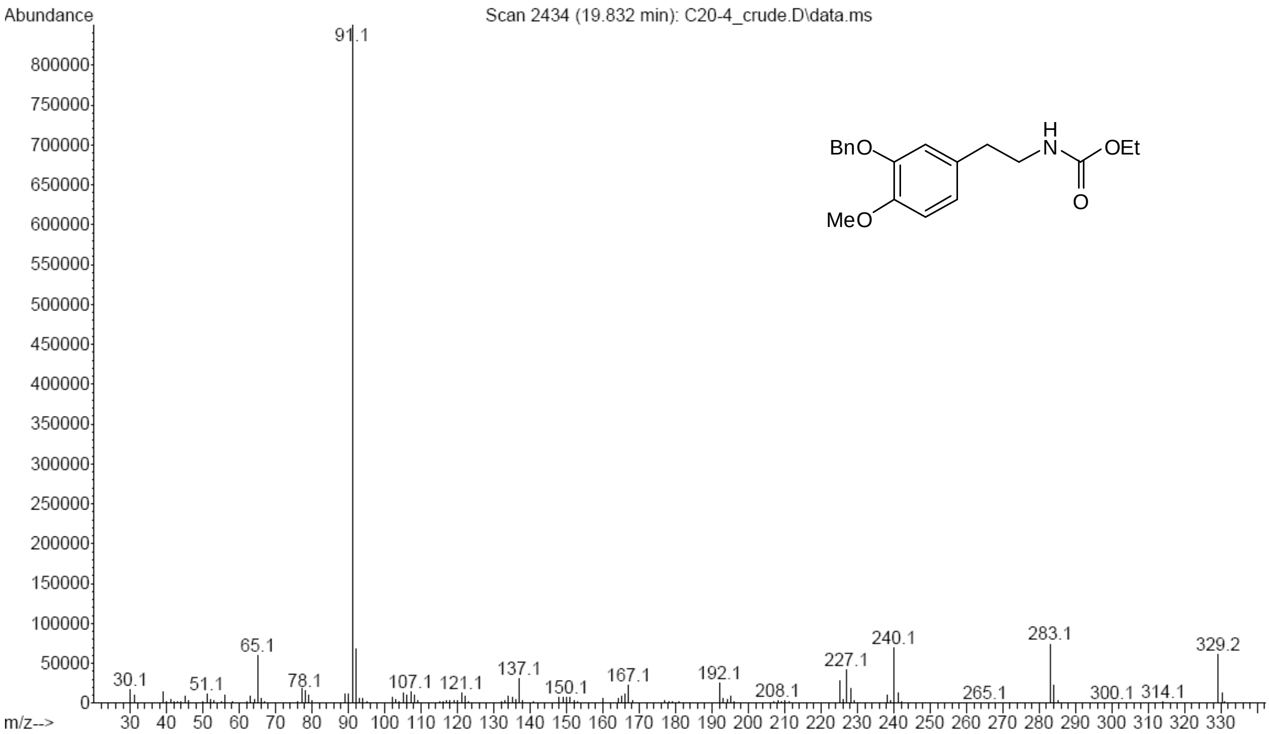

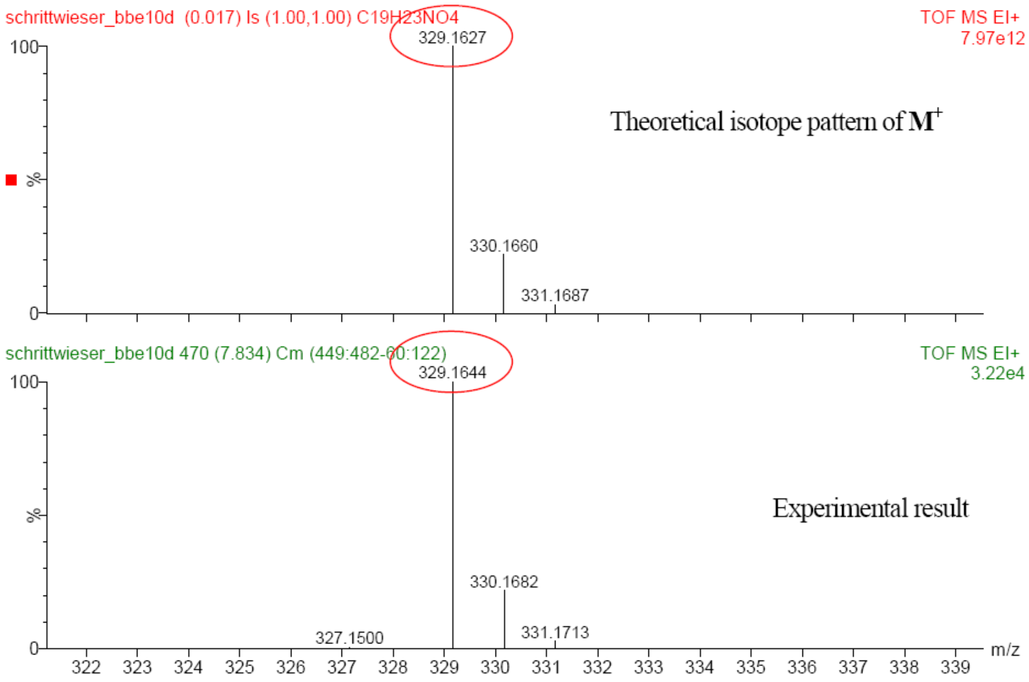

3-Benzyloxy-4-methoxy-*N*-methylphenethylamine

^{1}H-NMR spectrum

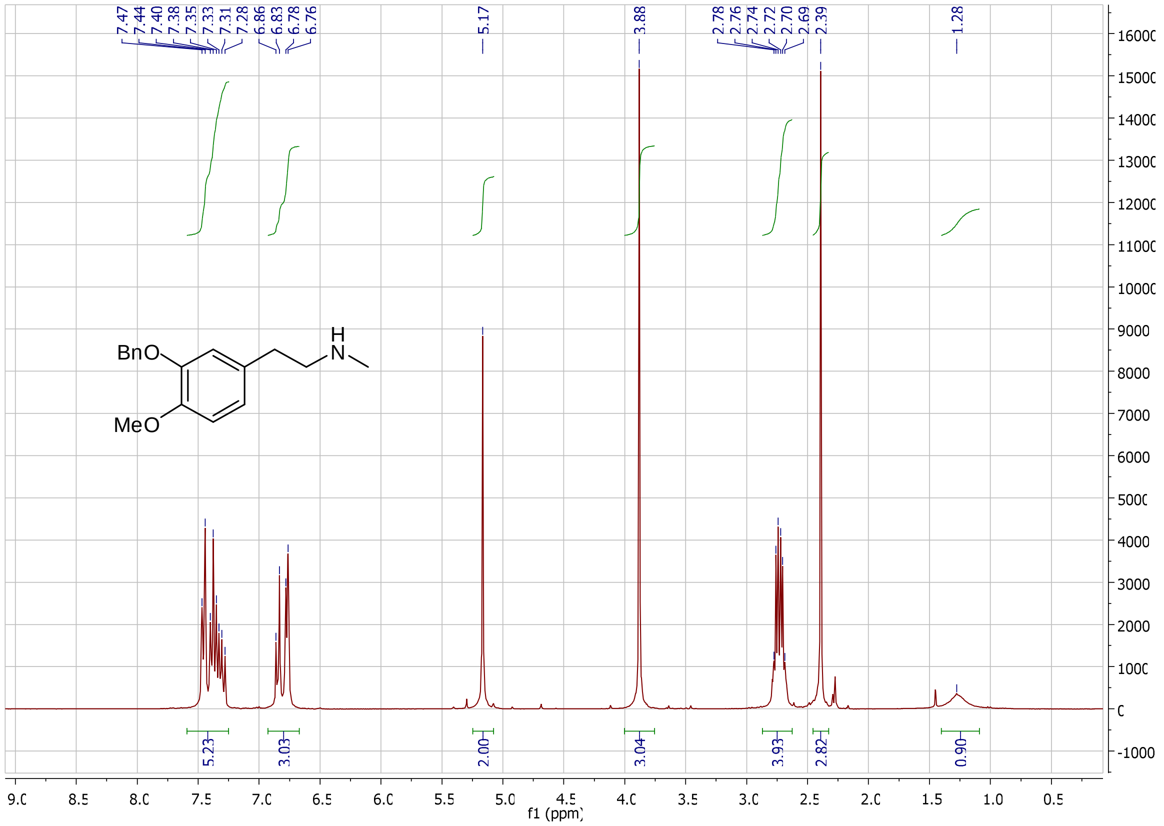

^{13}C-NMR spectrum

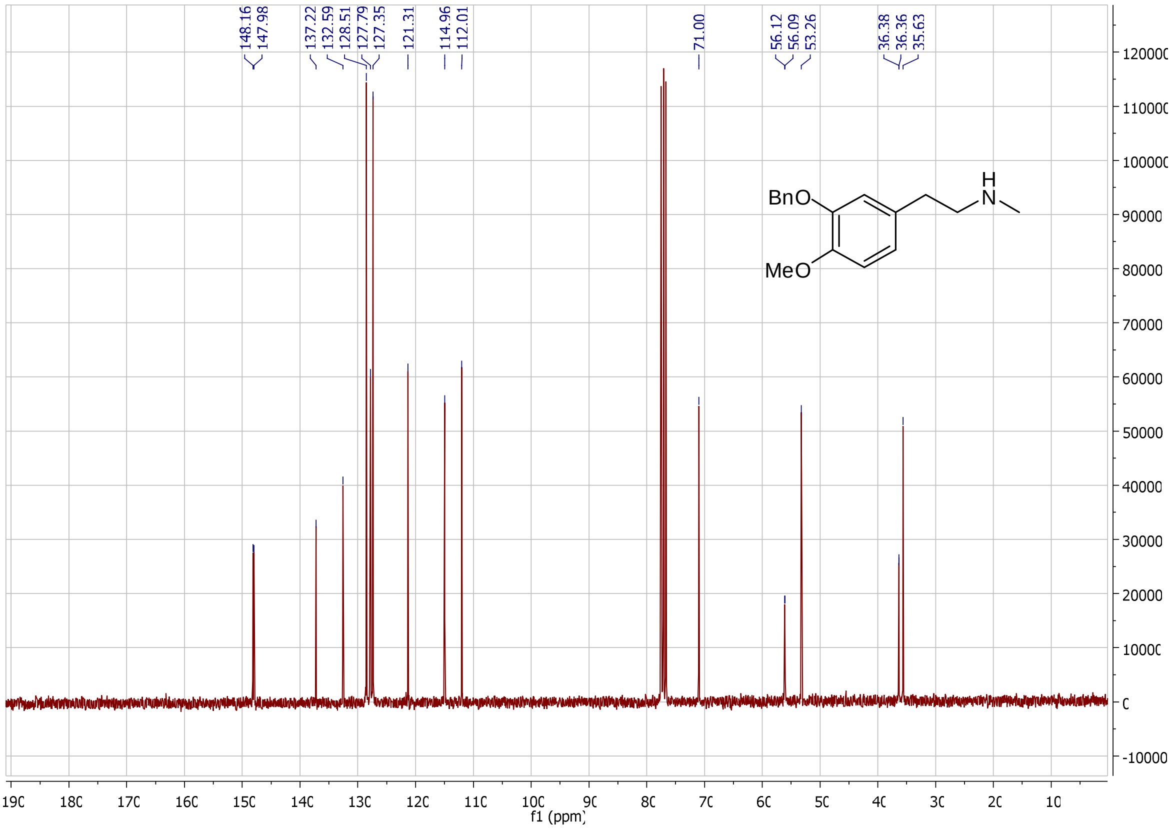

HRMS results

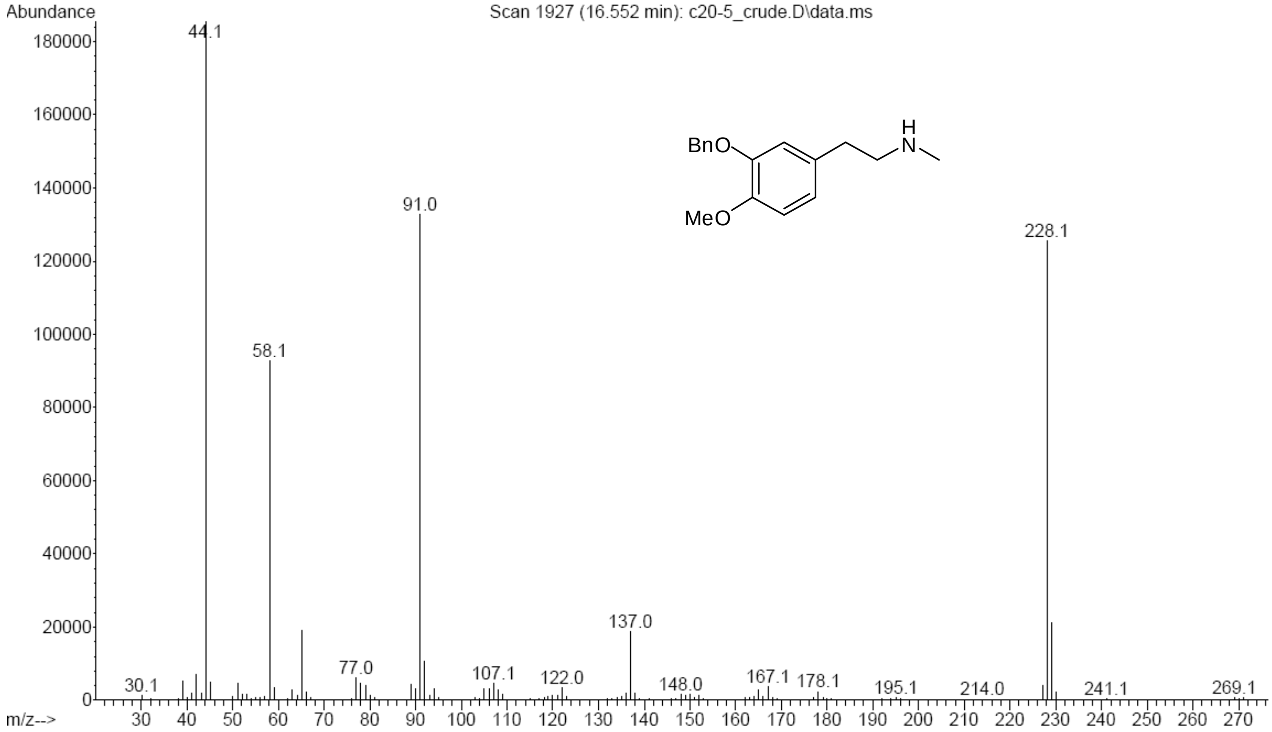

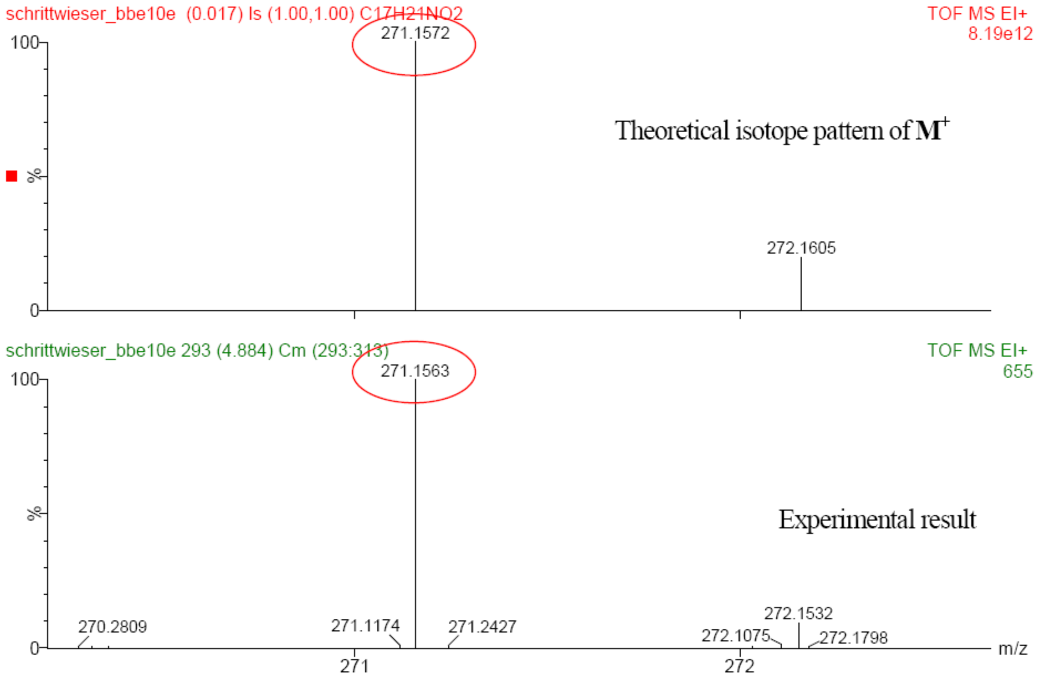

N-(3-Benzyloxy-4-methoxyphenethyl)-2-(3-benzyloxyphenyl)acetamide

^{1}H-NMR spectrum

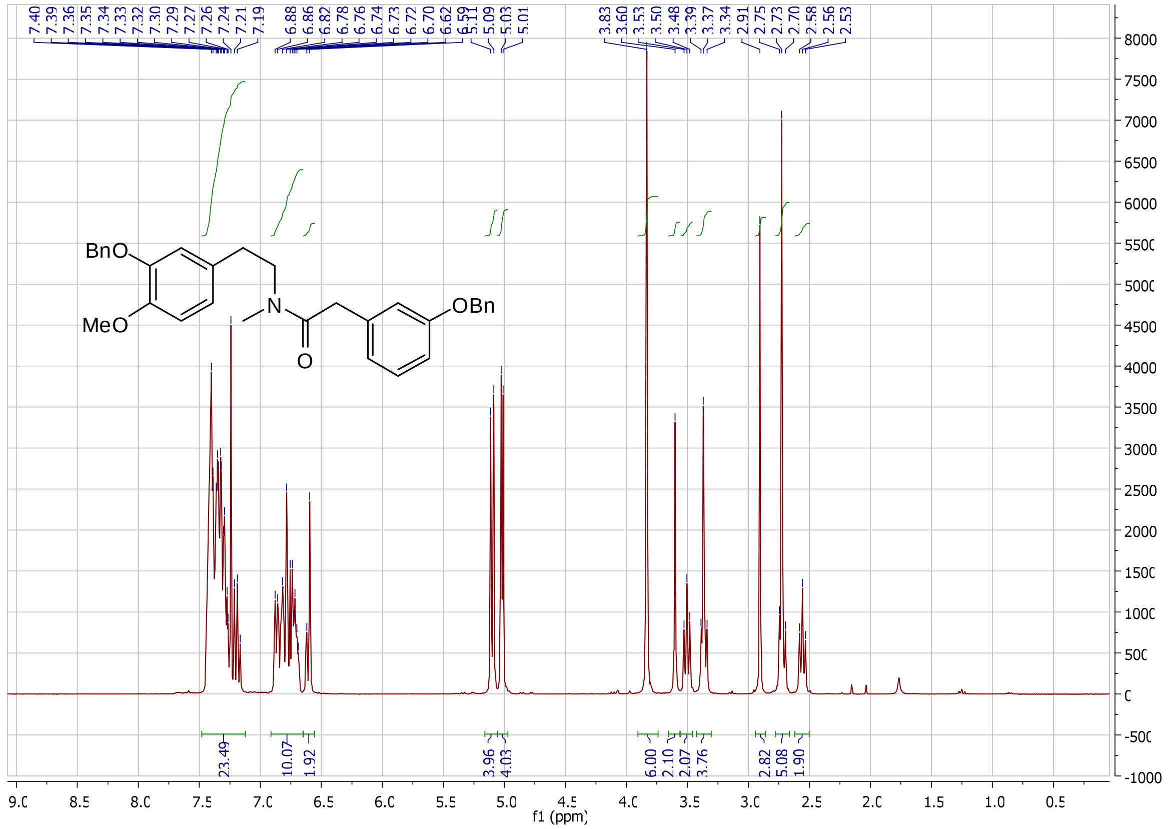

^{13}C-NMR spectrum

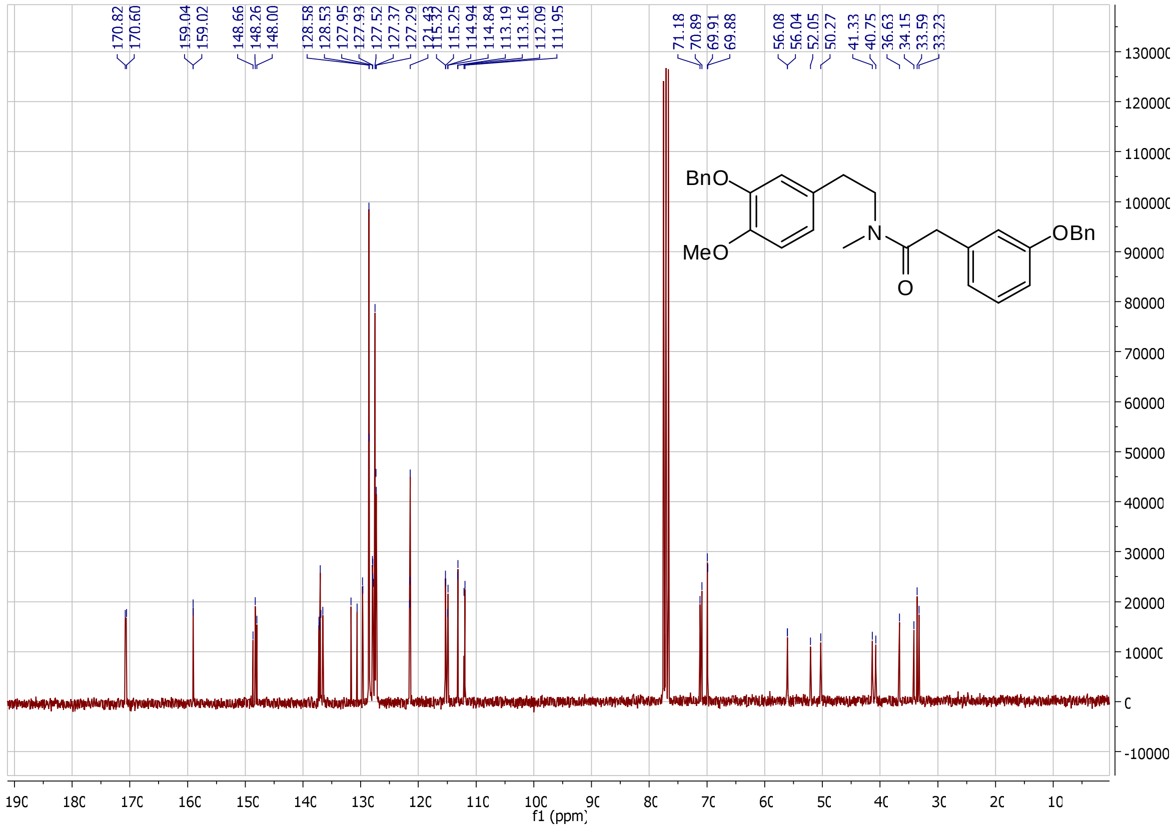

^{13}C-NMR DEPT135 spectrum

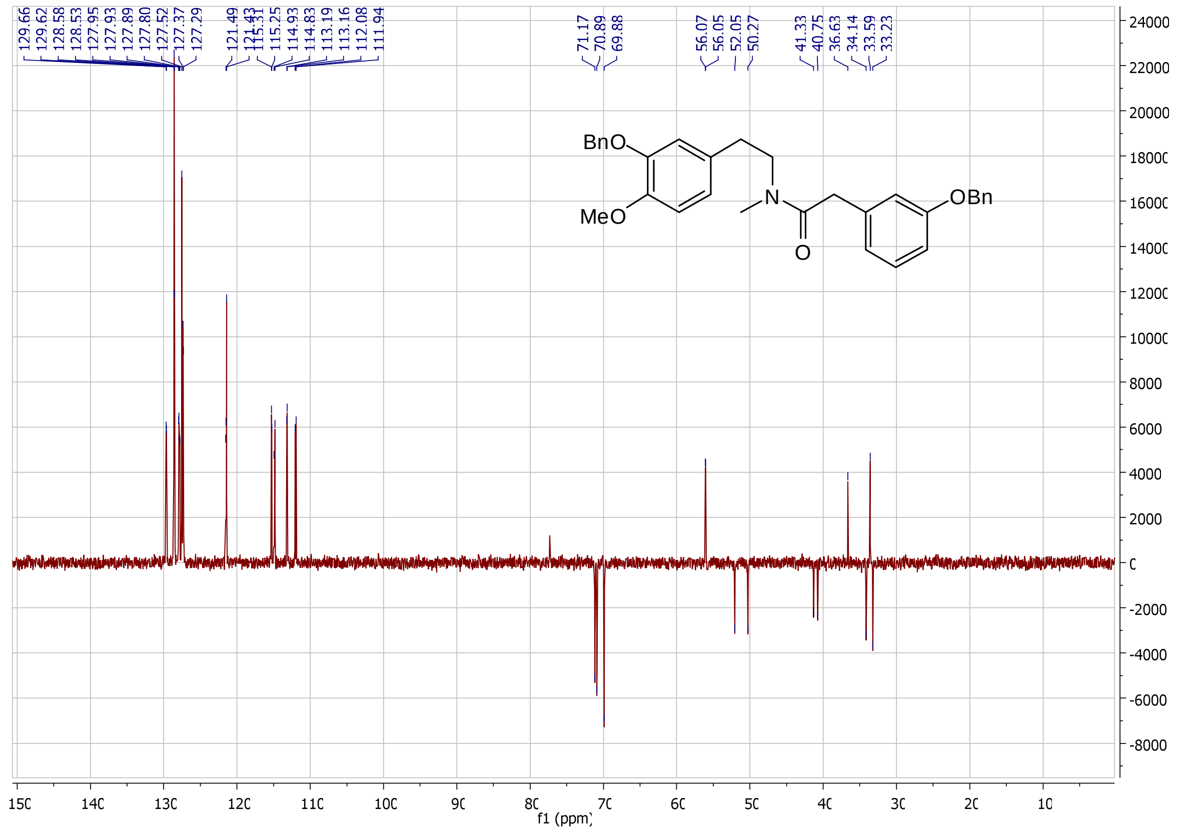

^{13}C-NMR DEPT90 spectrum

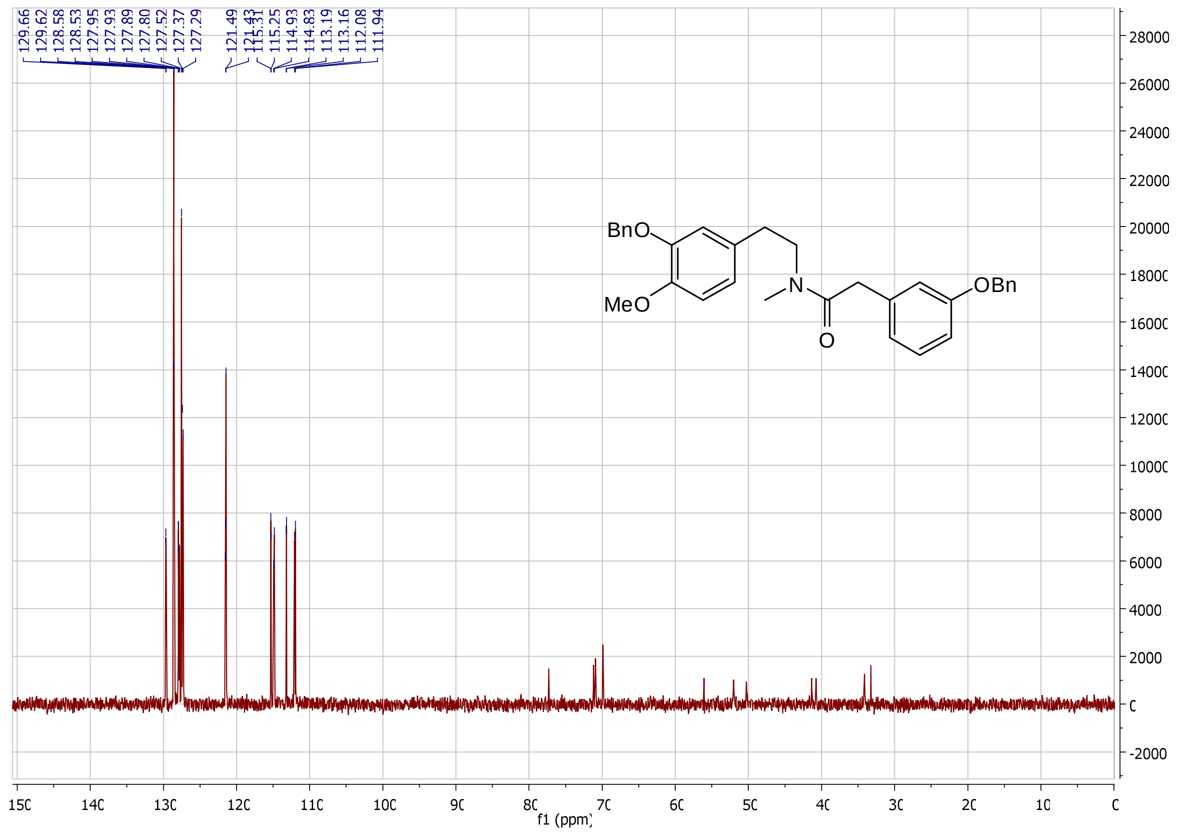

COSY spectrum

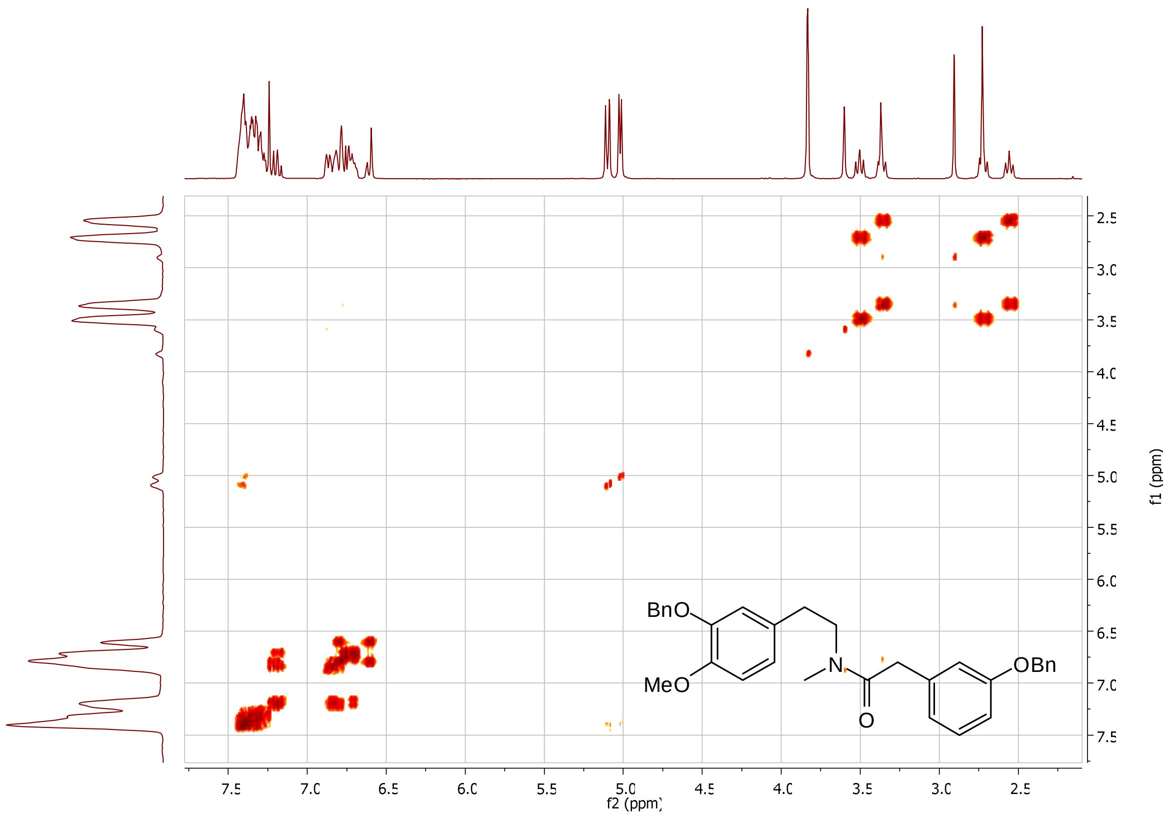

HSQC spectrum

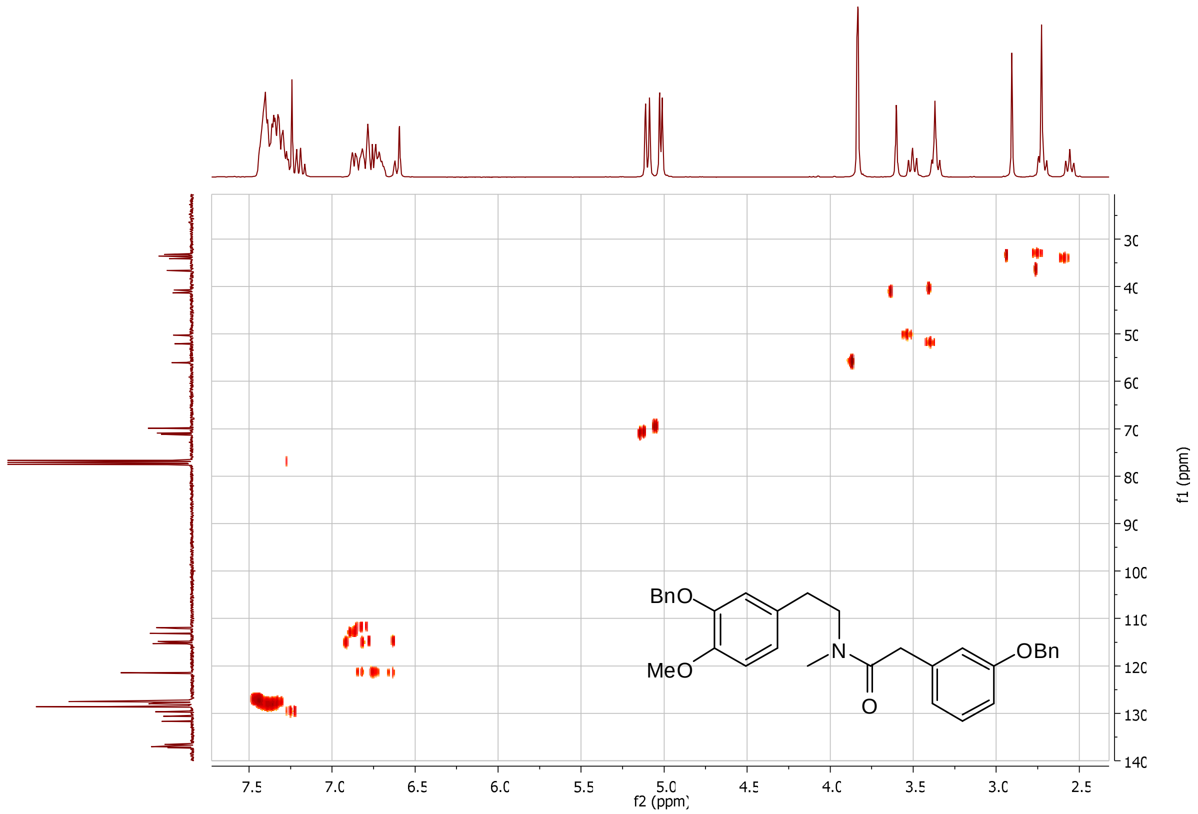

HRMS results

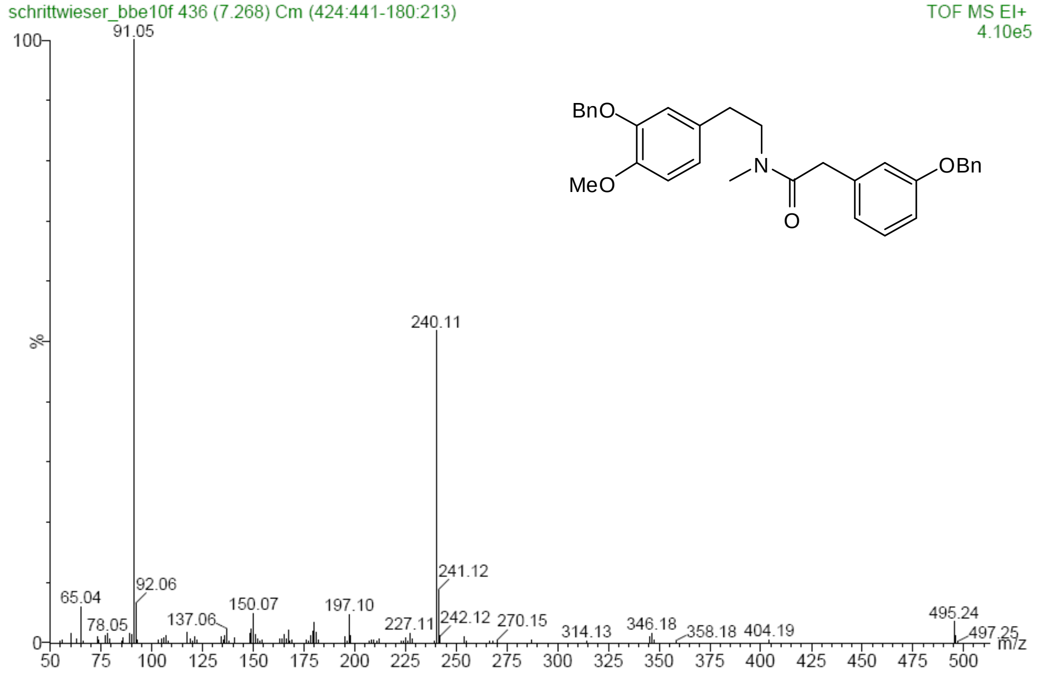

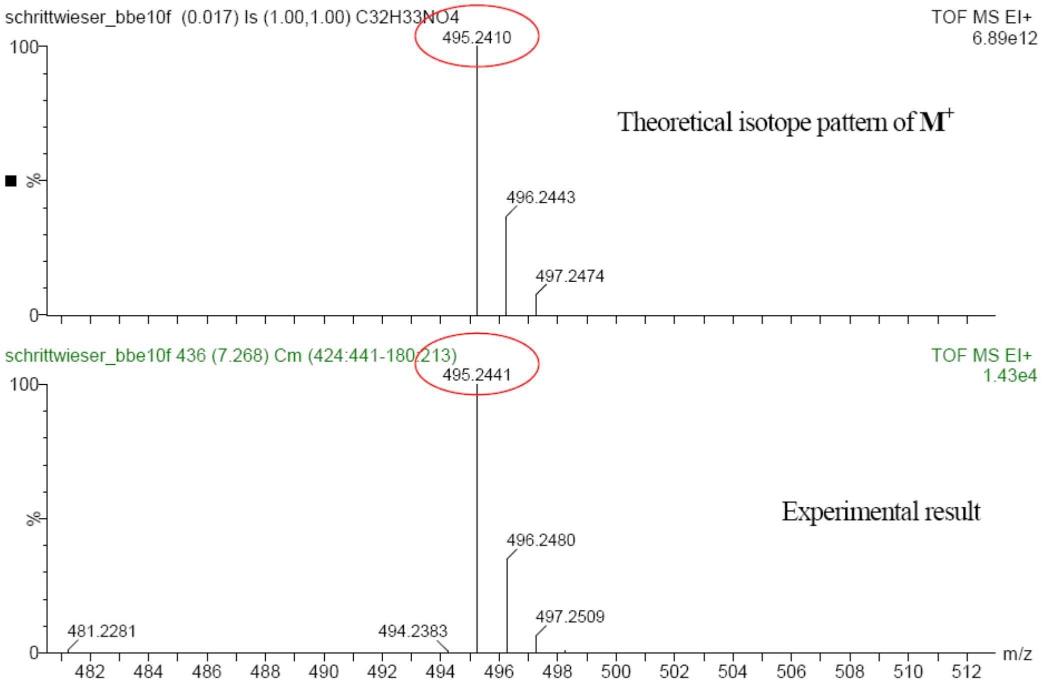

1-(3-Benzyloxybenzyl)-6-benzyloxy-7-methoxy-2-methyl-1,2,3,4-tetrahydroisoquinoline

^{1}H-NMR spectrum

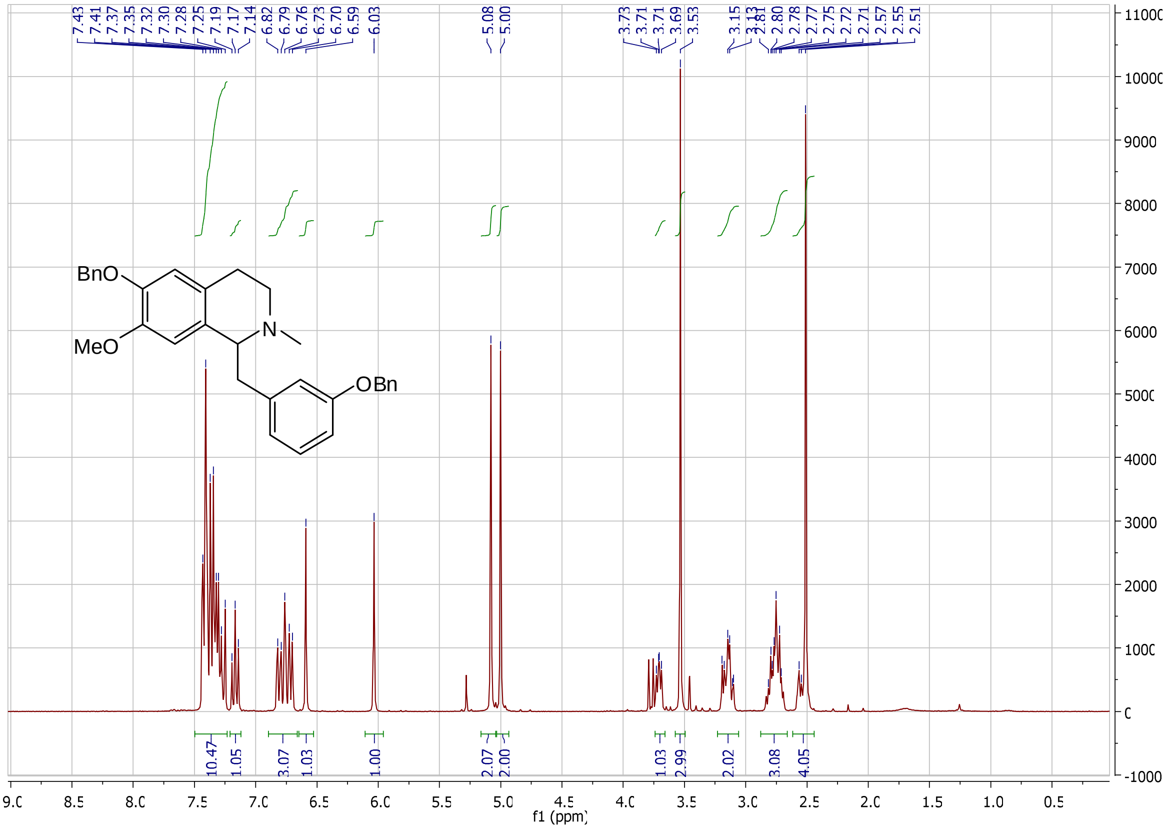

^{13}C-NMR spectrum

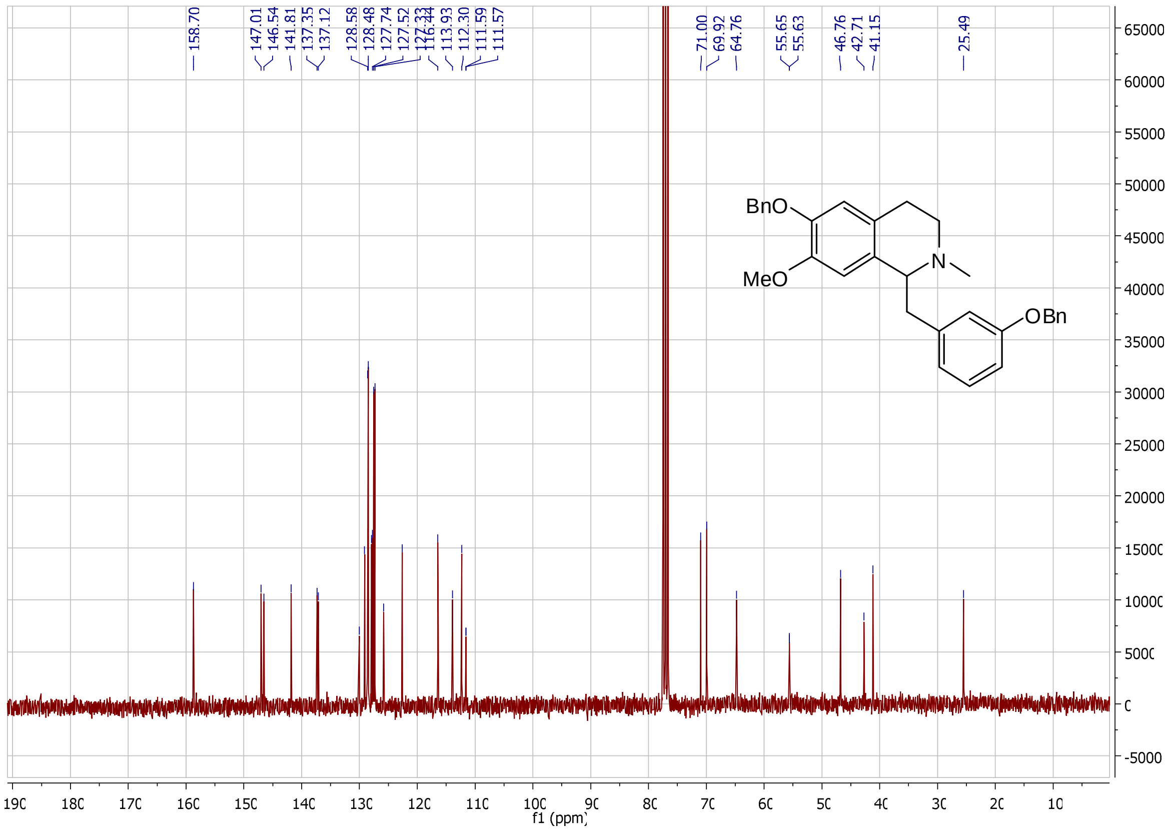

HRMS results

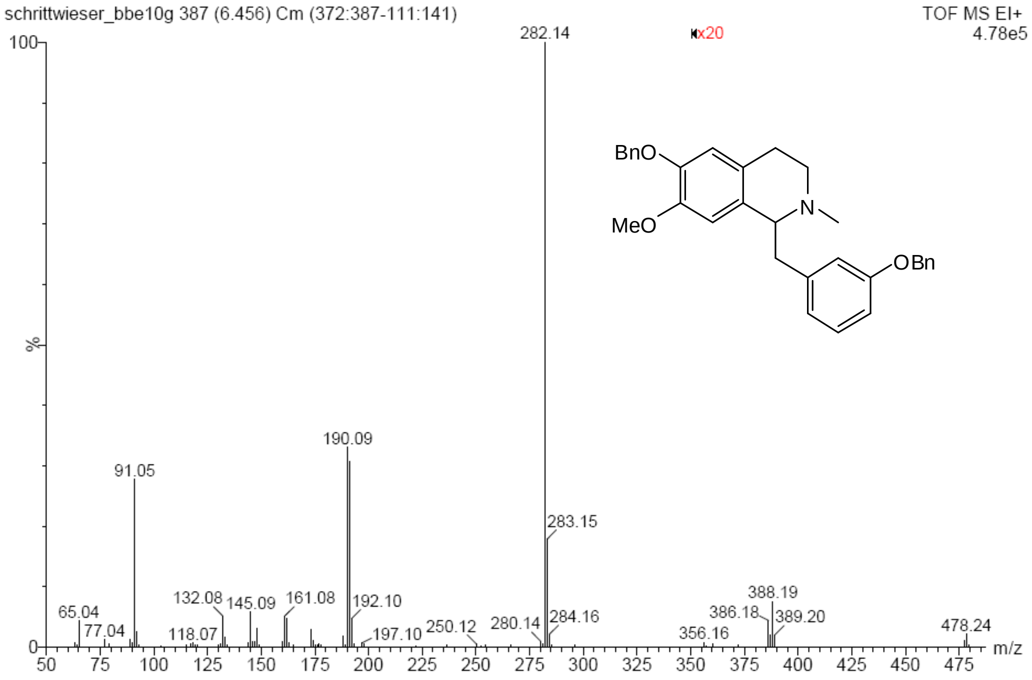

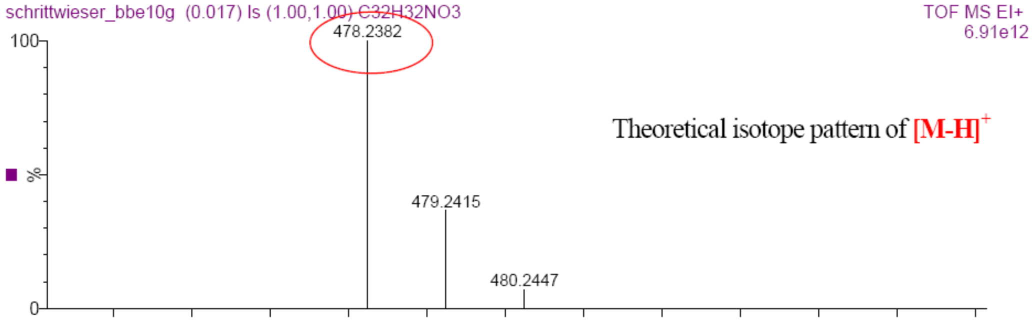

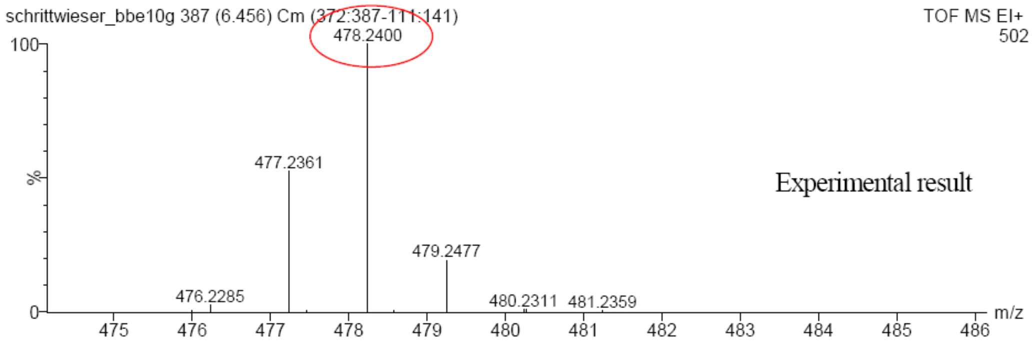

1-(3-Hydroxybenzyl)-6-hydroxy-7-methoxy-2-methyl-1,2,3,4-tetrahydroisoquinoline

^{1}H-NMR spectrum

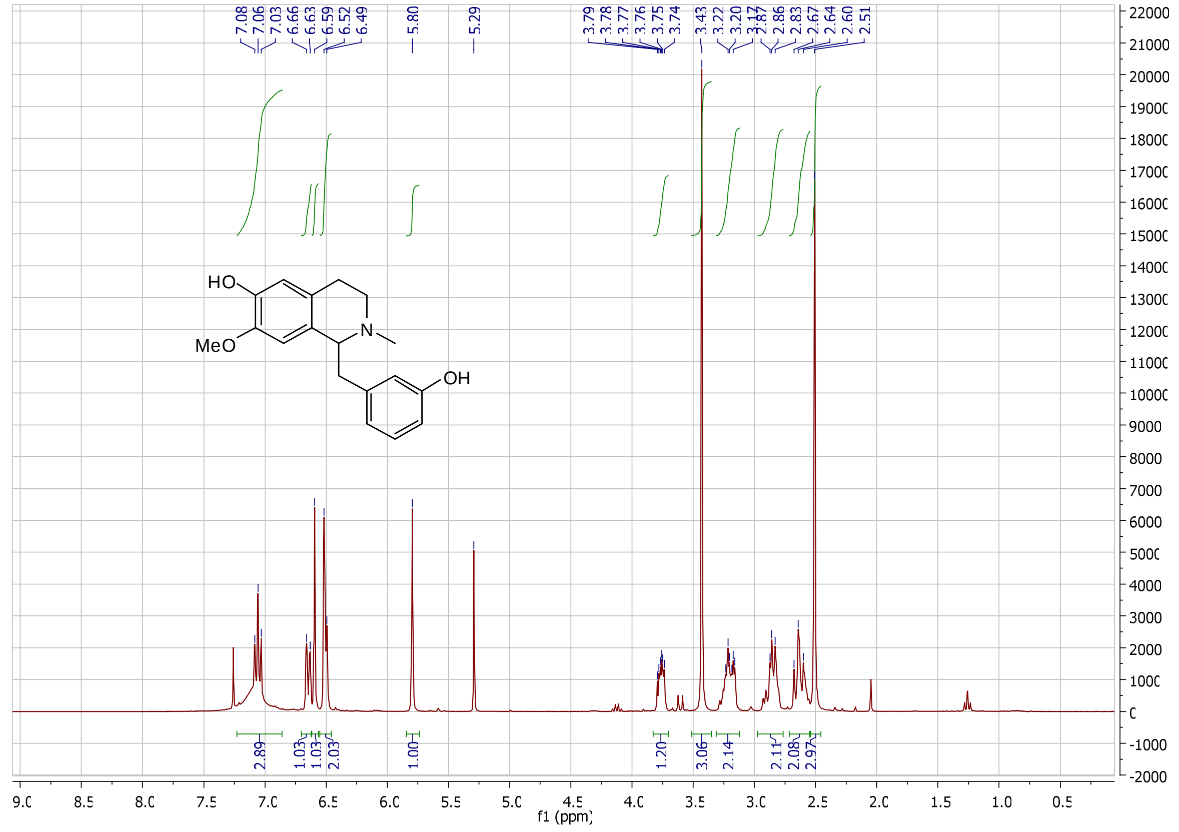

^{13}C-NMR spectrum

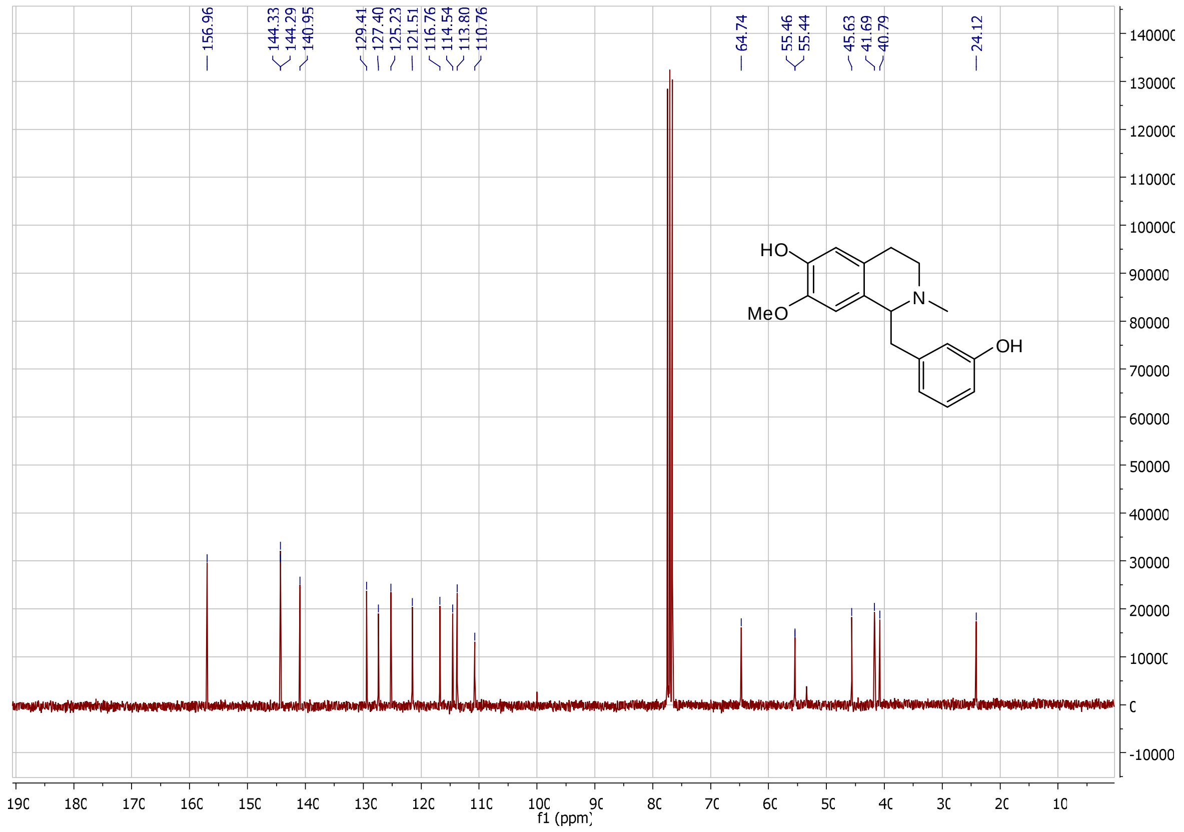

COSY spectrum

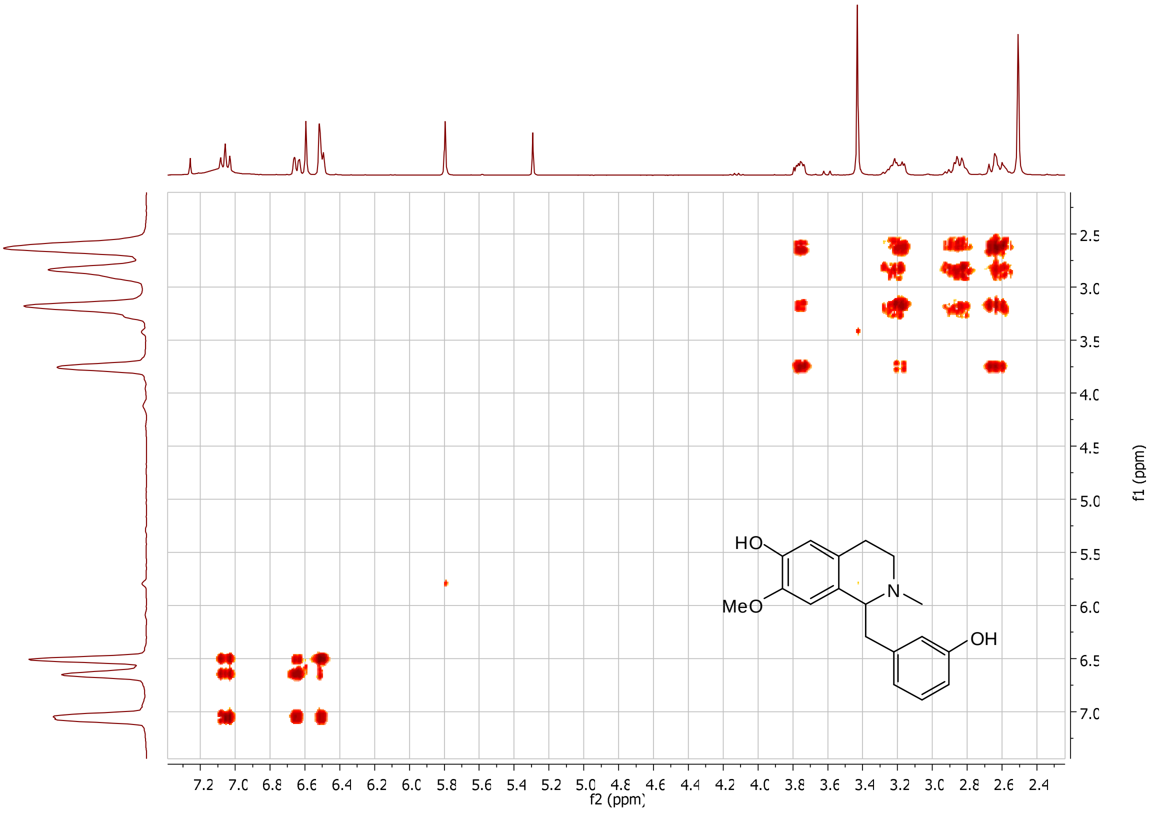

HSQC spectrum

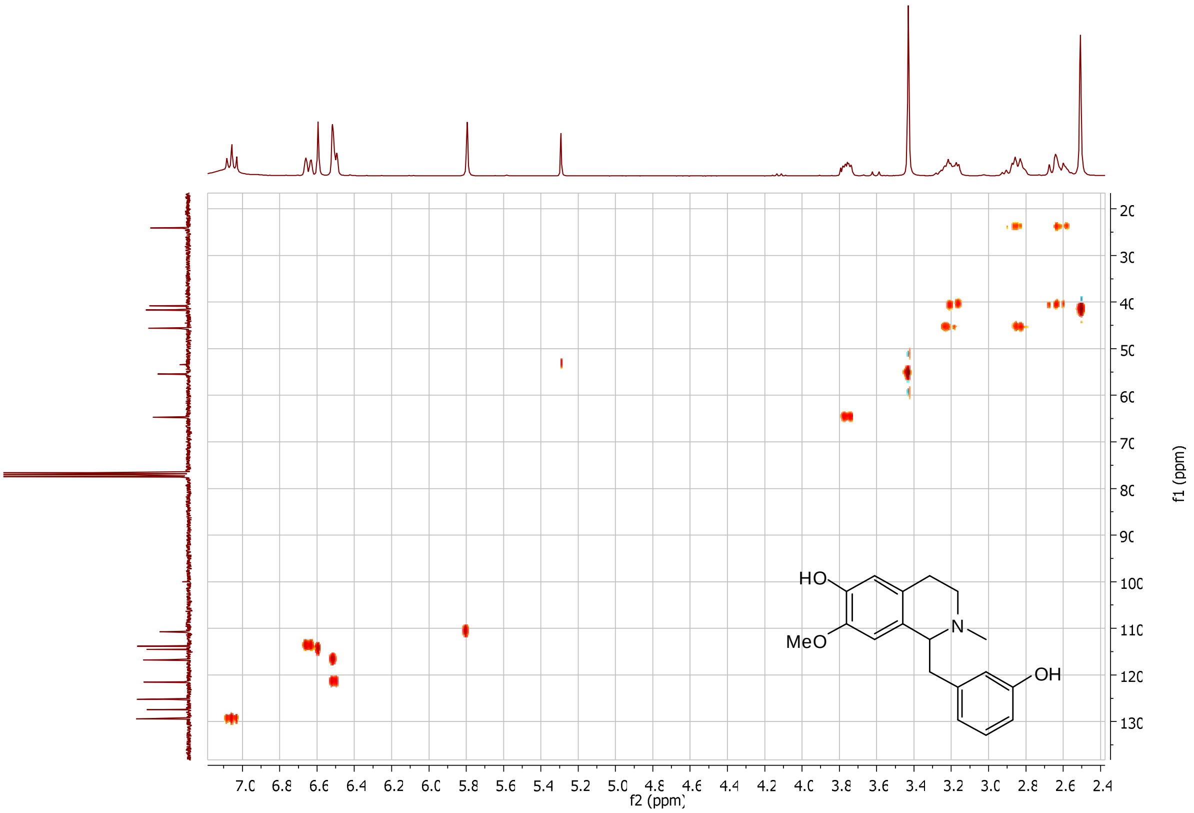

HRMS results

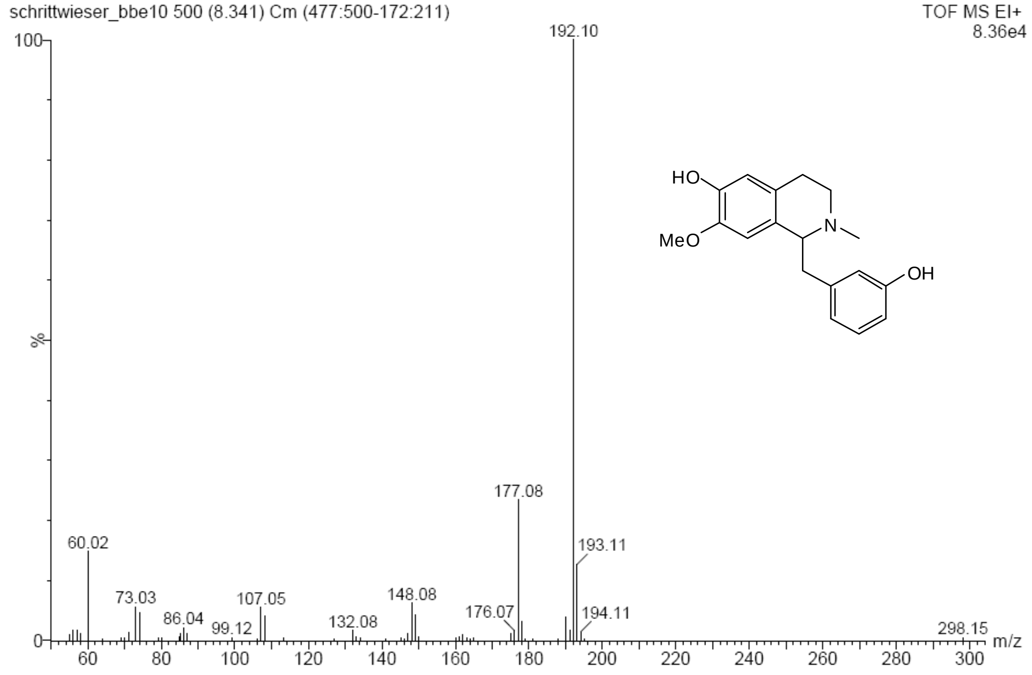

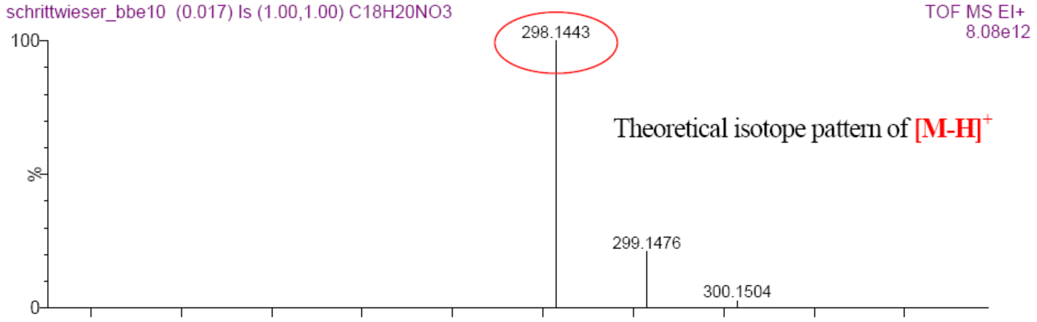

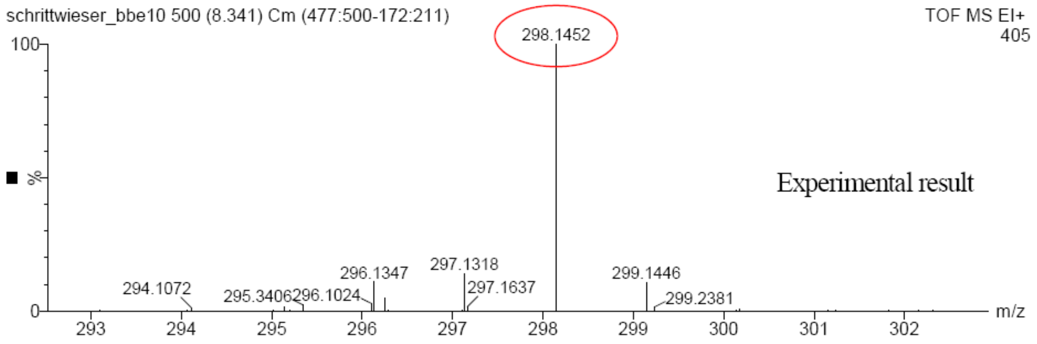

Synthesis of **1i**:

Provided Material:

1-(3-(Benzyloxy)-4-methoxyphenyl)-2,2,2-trichloroethanol
^{1}H-NMR spectrum, ^{13}C-NMR spectrum, HRMS results

2-(3-(Benzyloxy)-4-methoxyphenyl)acetic acid
^{1}H-NMR spectrum, ^{13}C-NMR spectrum

2-(3-(Benzyloxy)-4-methoxyphenyl)acetyl chloride
^{1}H-NMR spectrum, ^{13}C-NMR spectrum, HRMS results

***N*-(3-(Benzyloxy)-4-methoxyphenethyl)-2-(3-(benzyloxy)-4-methoxyphenyl)-*N*-methylacetamide**
^{1}H-NMR spectrum, ^{13}C-NMR spectrum, ^{13}C-NMR DEPT135 spectrum, ^{13}C-NMR DEPT90 spectrum, COSY spectrum, HSQC spectrum, MS spectrum, HRMS results

6-(Benzyloxy)-1-(3-(benzyloxy)-4-methoxybenzyl)-7-methoxy-2-methyl-1,2,3,4-tetrahydroisoquinoline
^{1}H-NMR spectrum, ^{13}C-NMR spectrum, HRMS results

1-(3-Hydroxy-4-methoxybenzyl)-7-methoxy-2-methyl-1,2,3,4-tetrahydroisoquinolin-6-ol
^{1}H-NMR spectrum, ^{13}C-NMR spectrum, HRMS results

1-(3-(Benzyloxy)-4-methoxyphenyl)-2,2,2-trichloroethanol

^{1}H-NMR

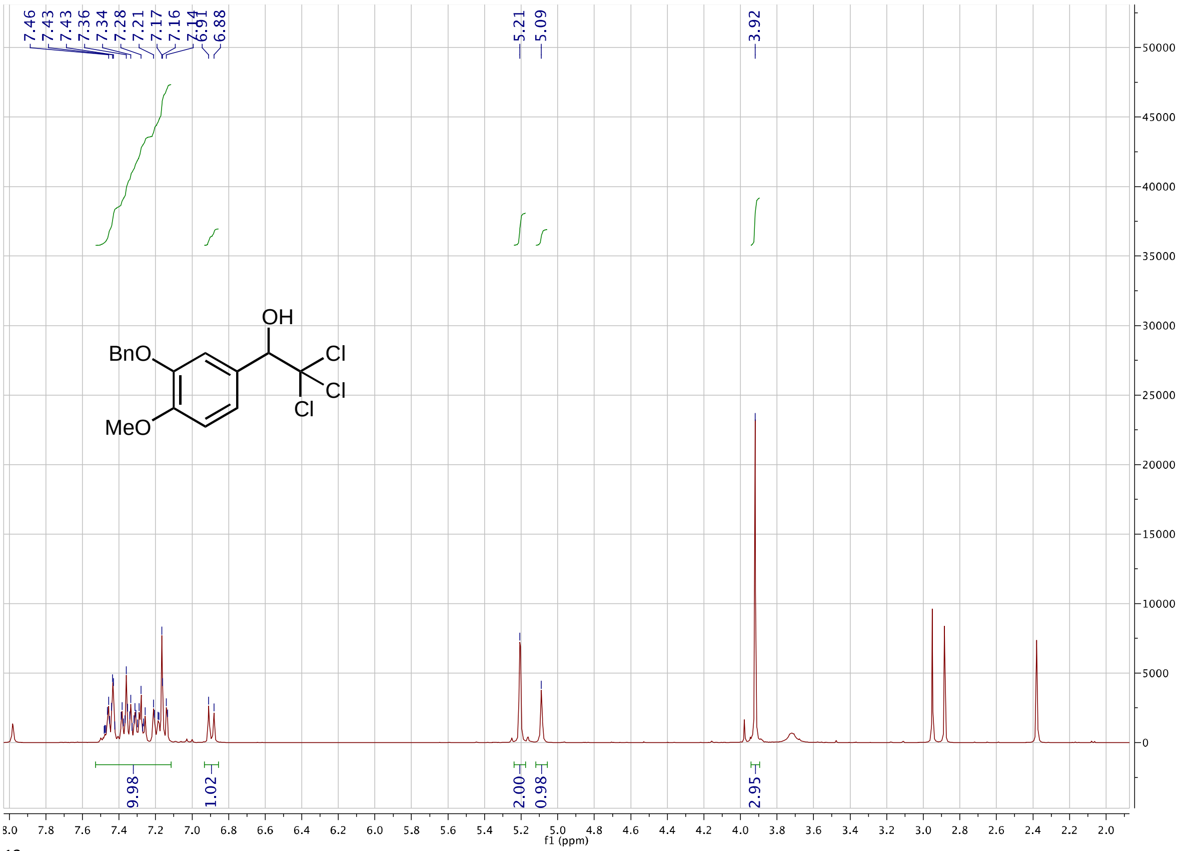

^{13}C-NMR

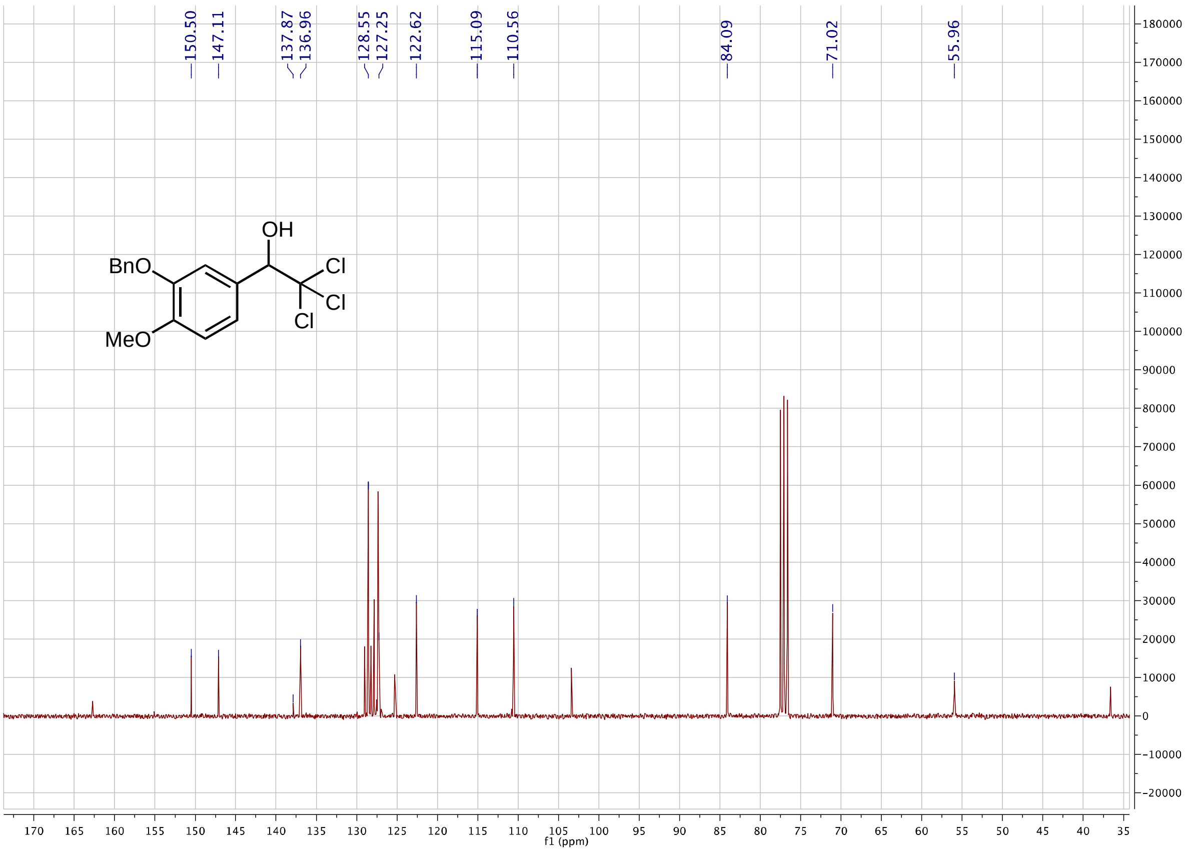

HRMS results

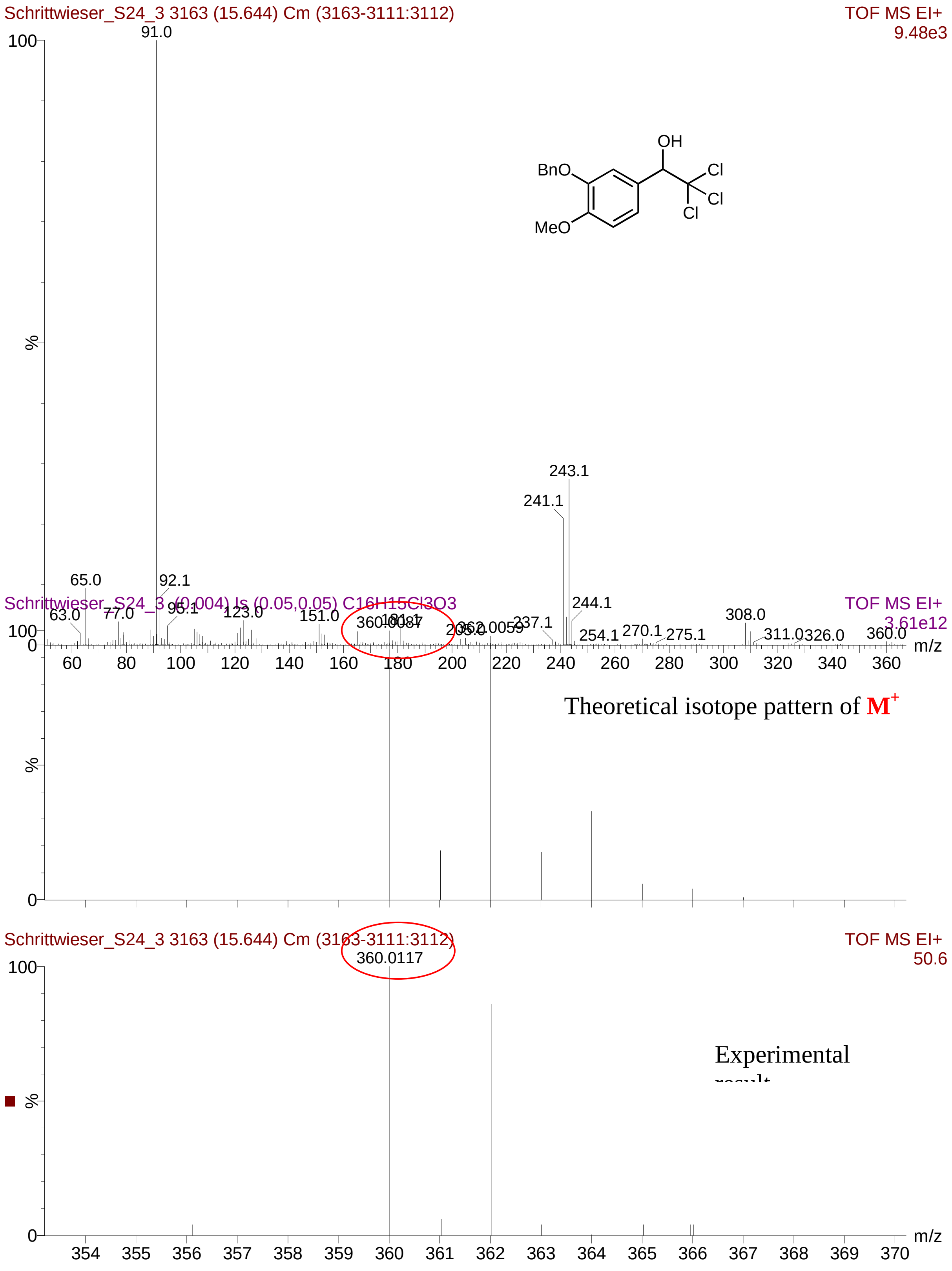

2-(3-(Benzyloxy)-4-methoxyphenyl)acetic acid

^{1}H-NMR

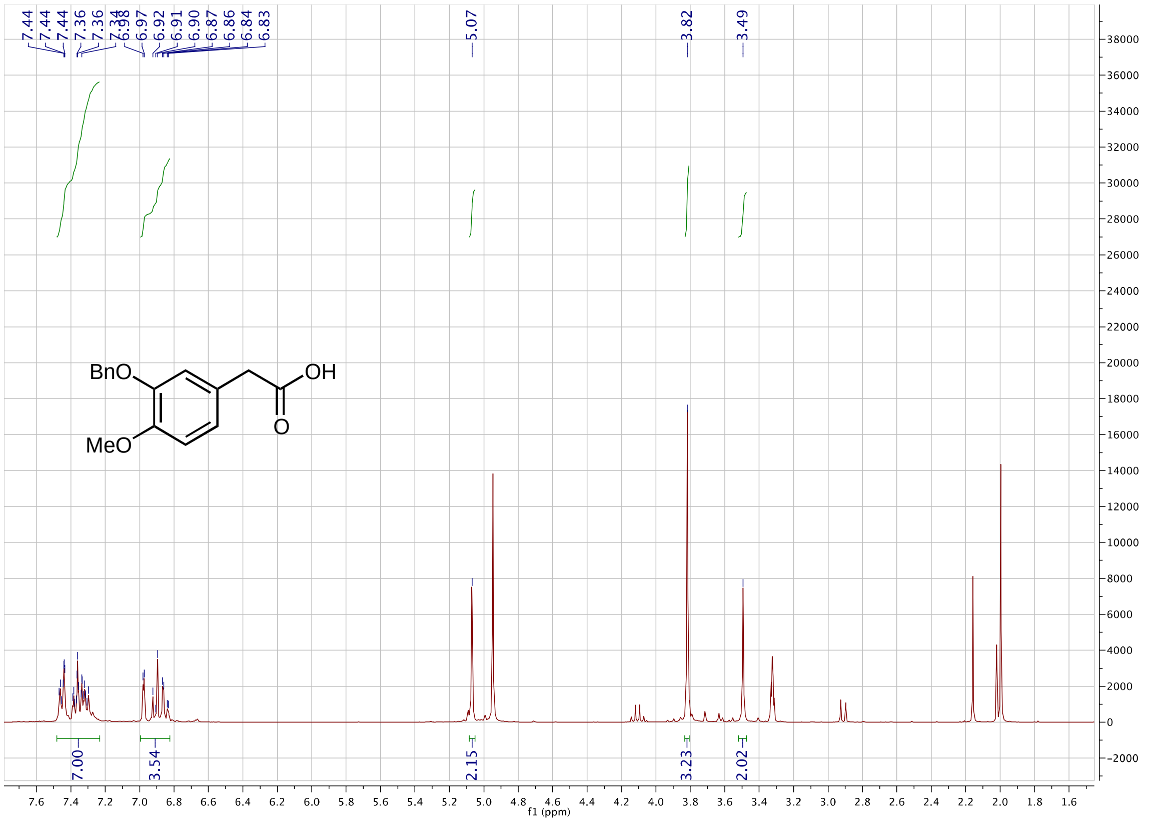

^{13}C-NMR

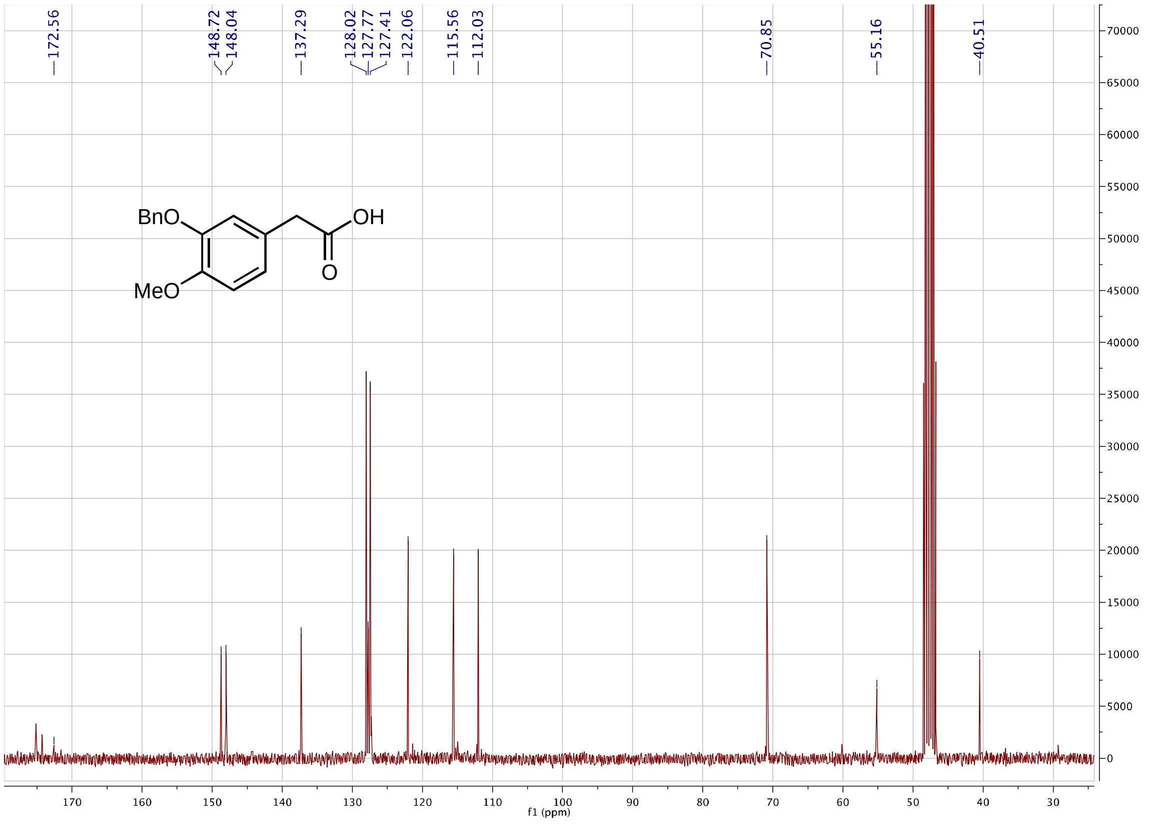

***N*-(3-(Benzyloxy)-4-methoxyphenethyl)-2-(3-(benzyloxy)-4-methoxyphenyl)-*N*-methylacetamide**

^{1}H-NMR

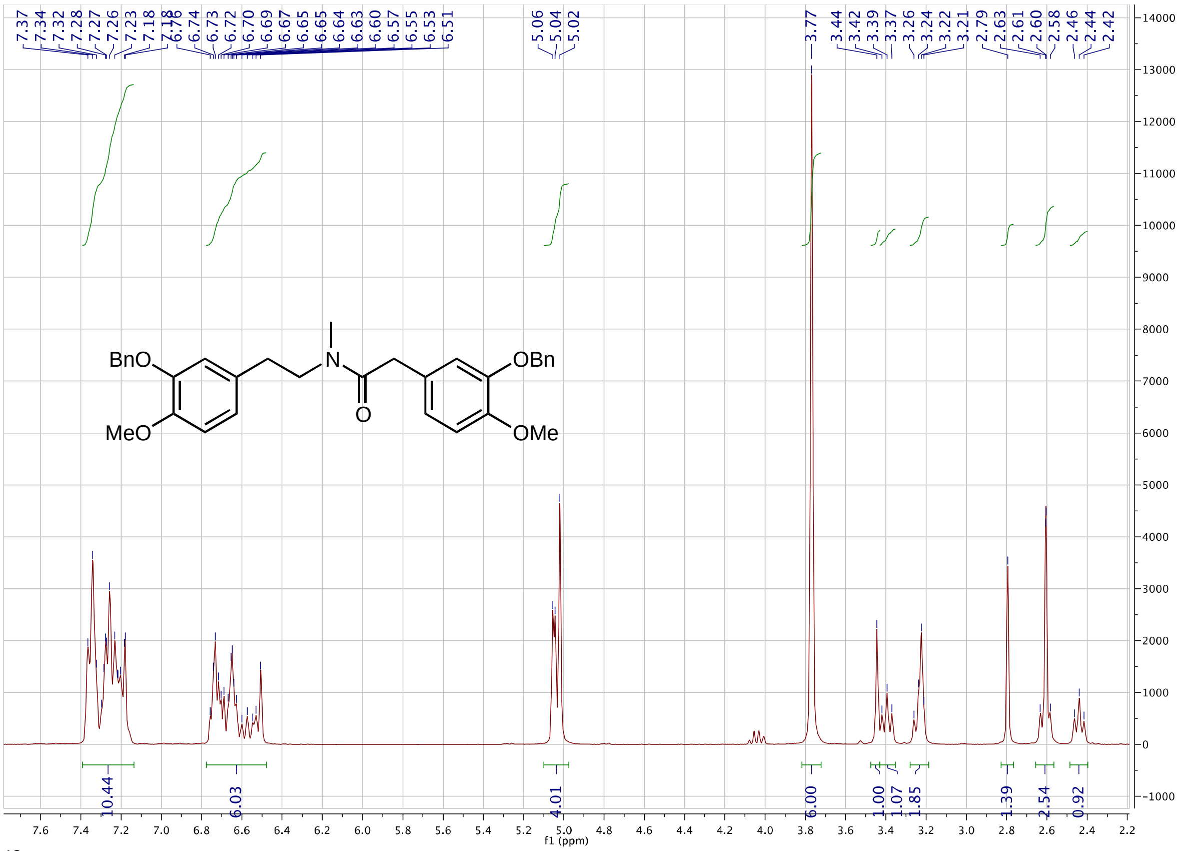

^{13}C-NMR

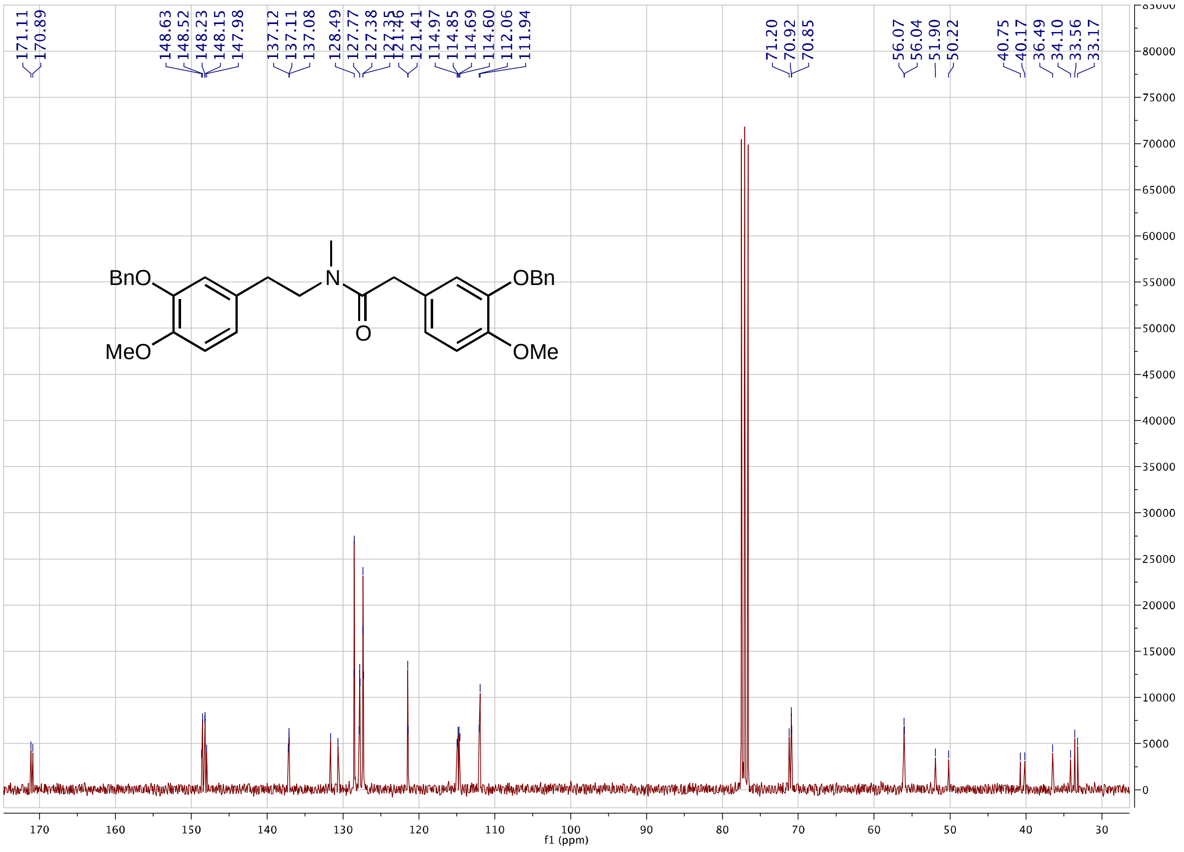

COSY spectrum

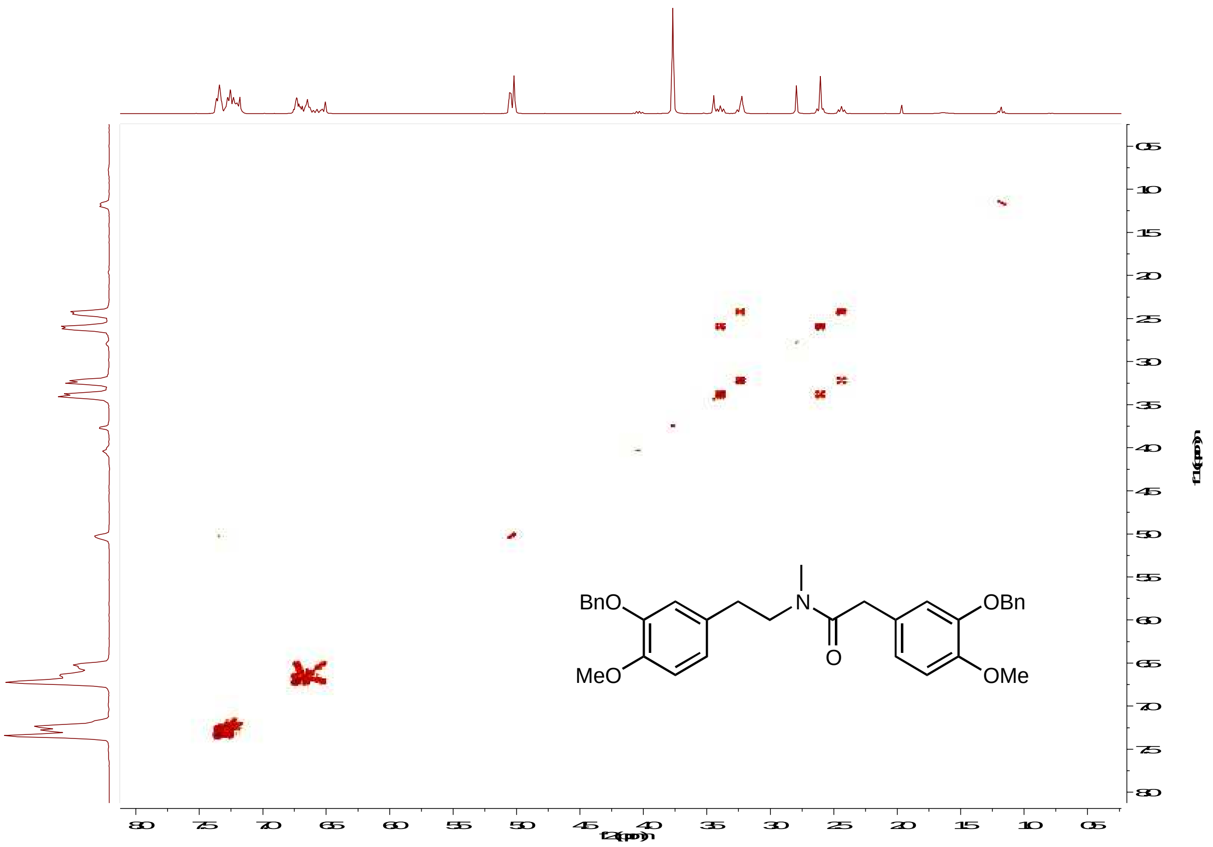

HSQC spectrum

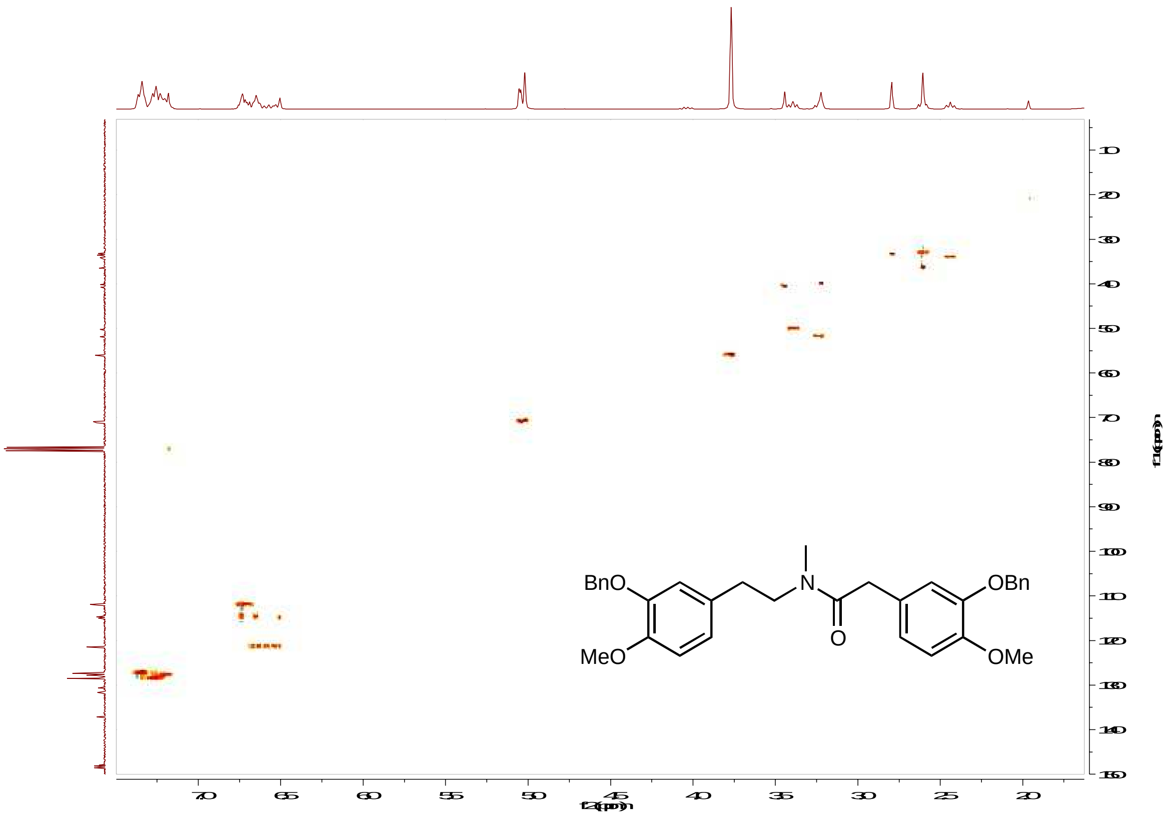

^{13}C-NMR DEPT90

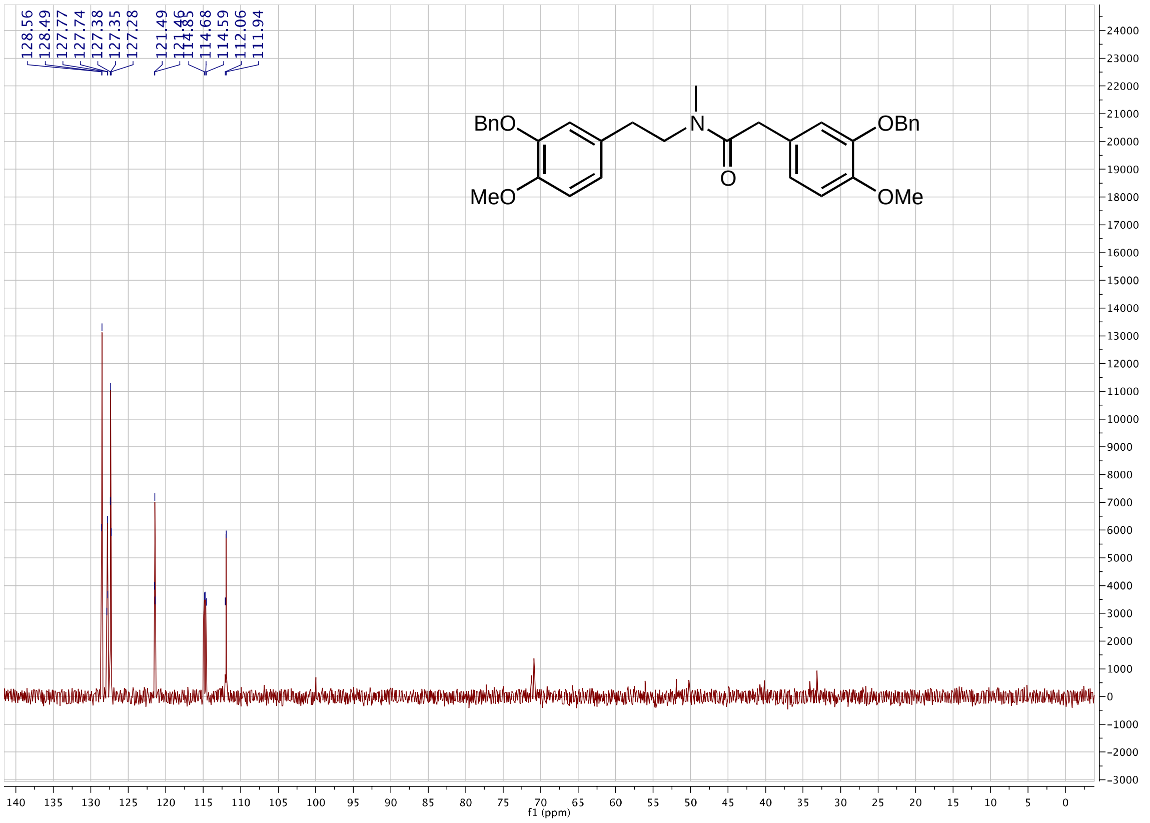

^{13}C-NMR DEPT135 spectrum

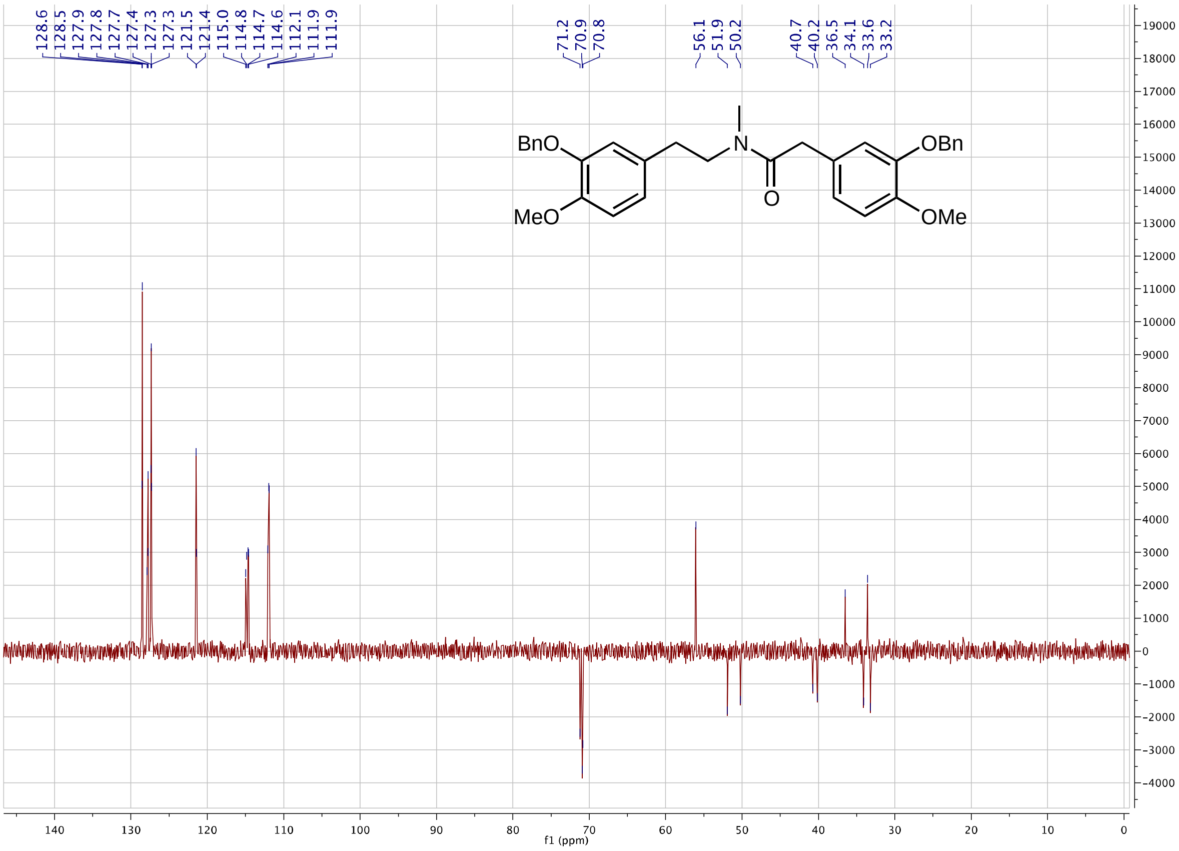

HRMS results

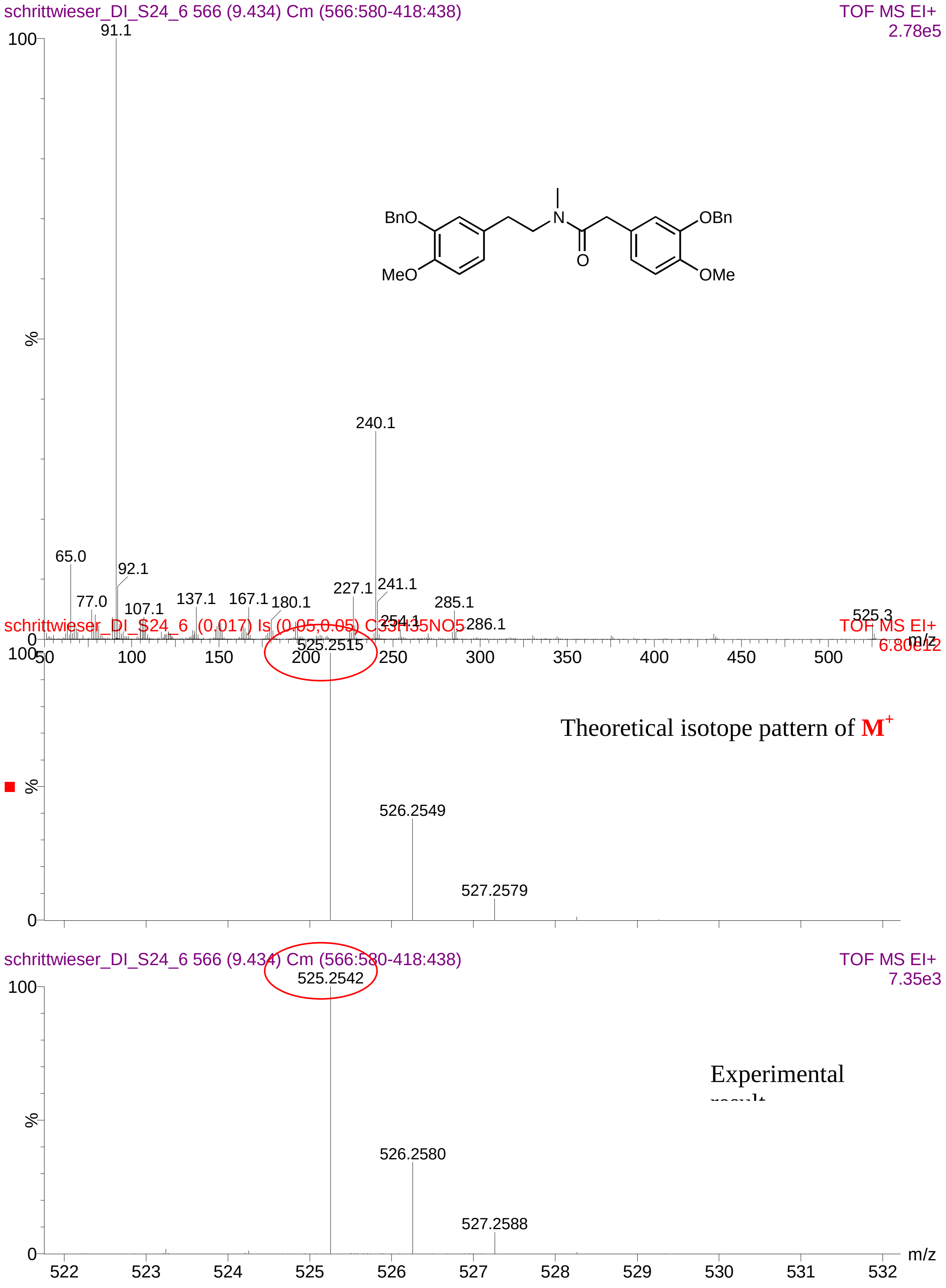

6-(Benzyloxy)-1-(3-(benzyloxy)-4-methoxybenzyl)-7-methoxy-2-methyl-1,2,3,4-tetrahydroisoquinoline

^{1}H-NMR

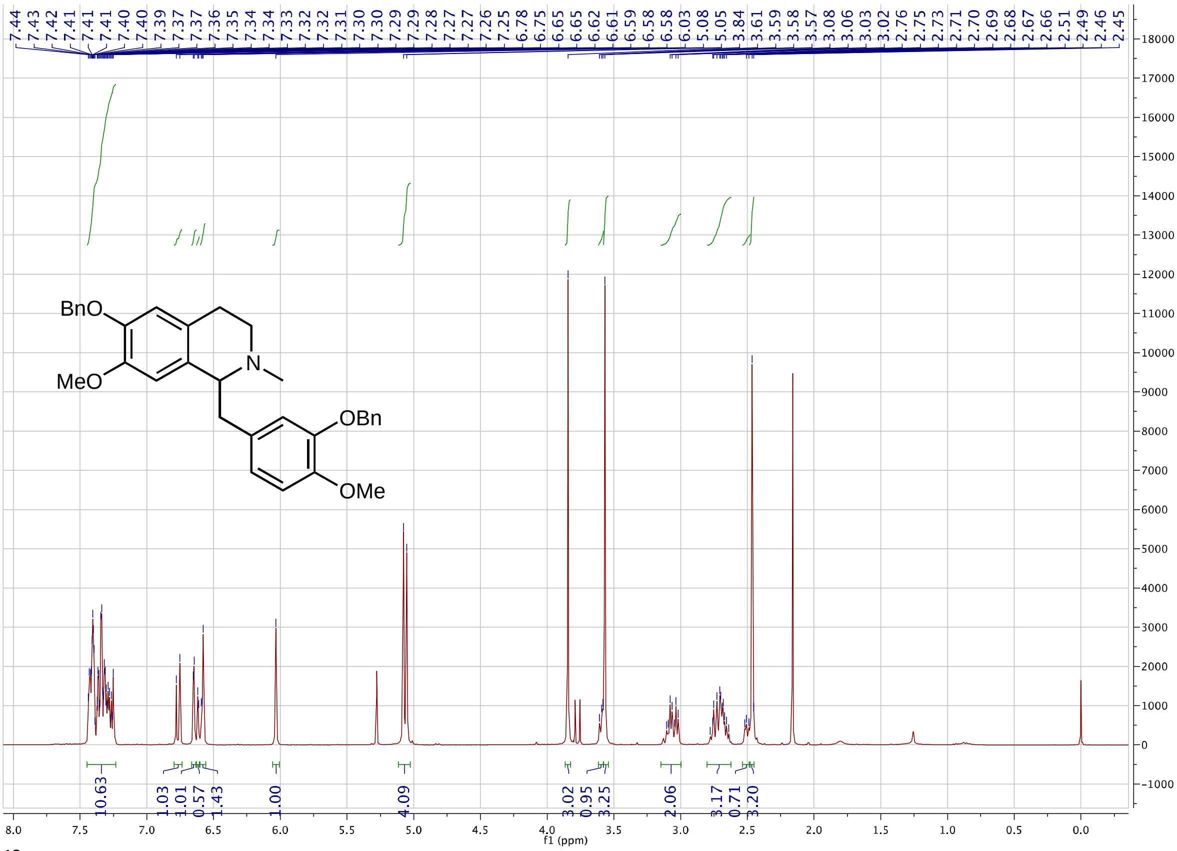

^{13}C-NMR

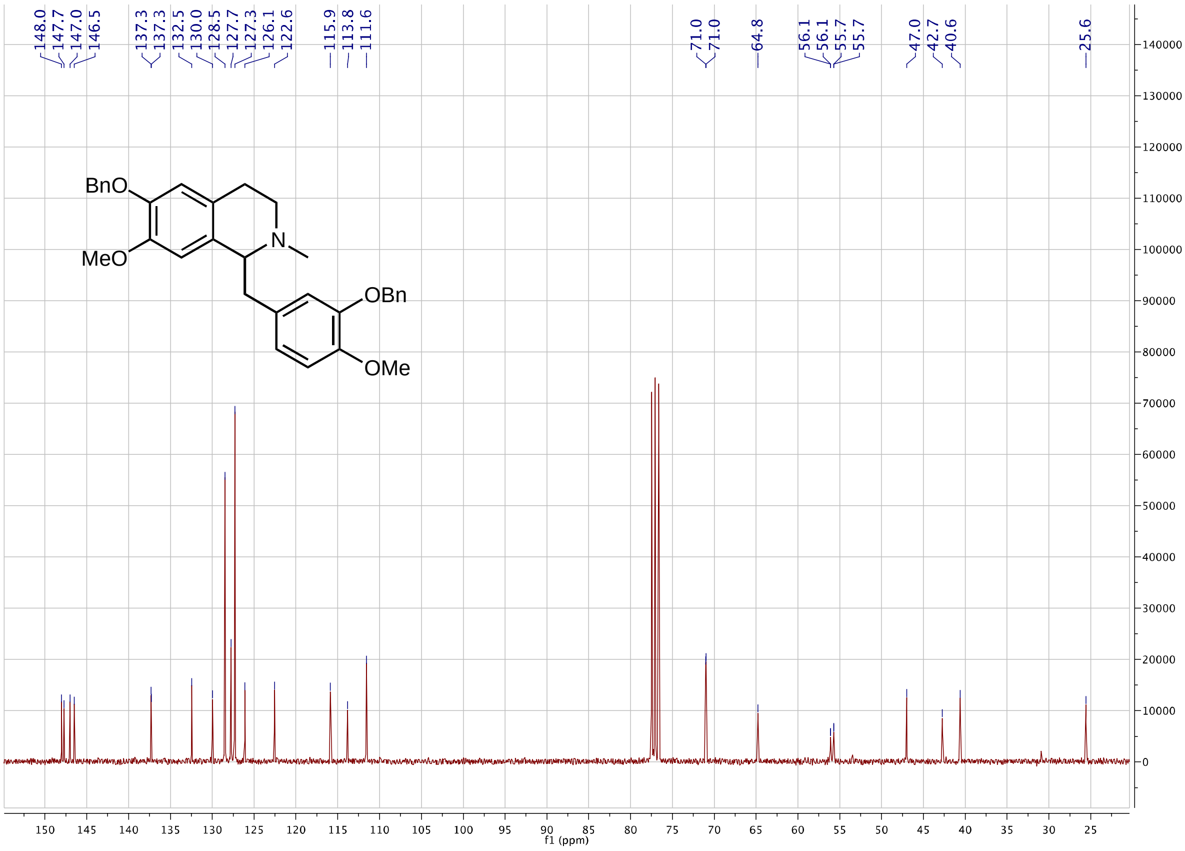

HSQC spectrum

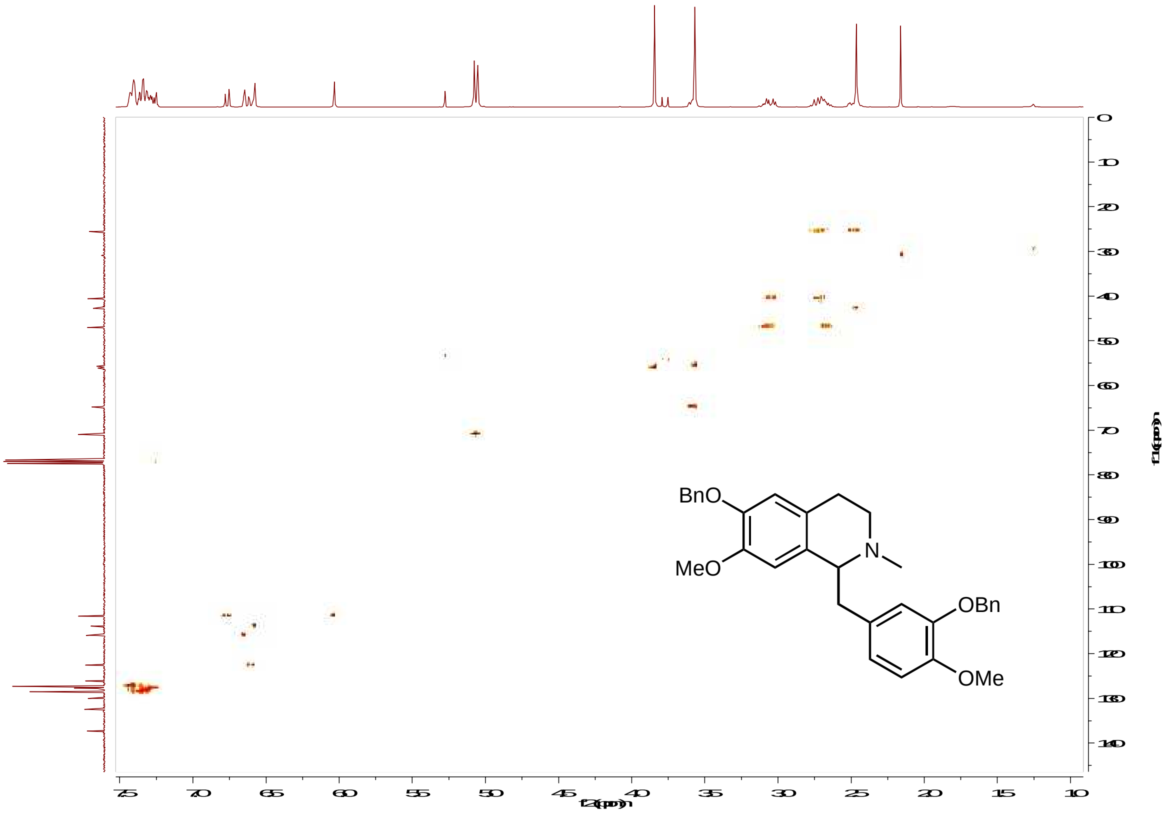

HRMS results

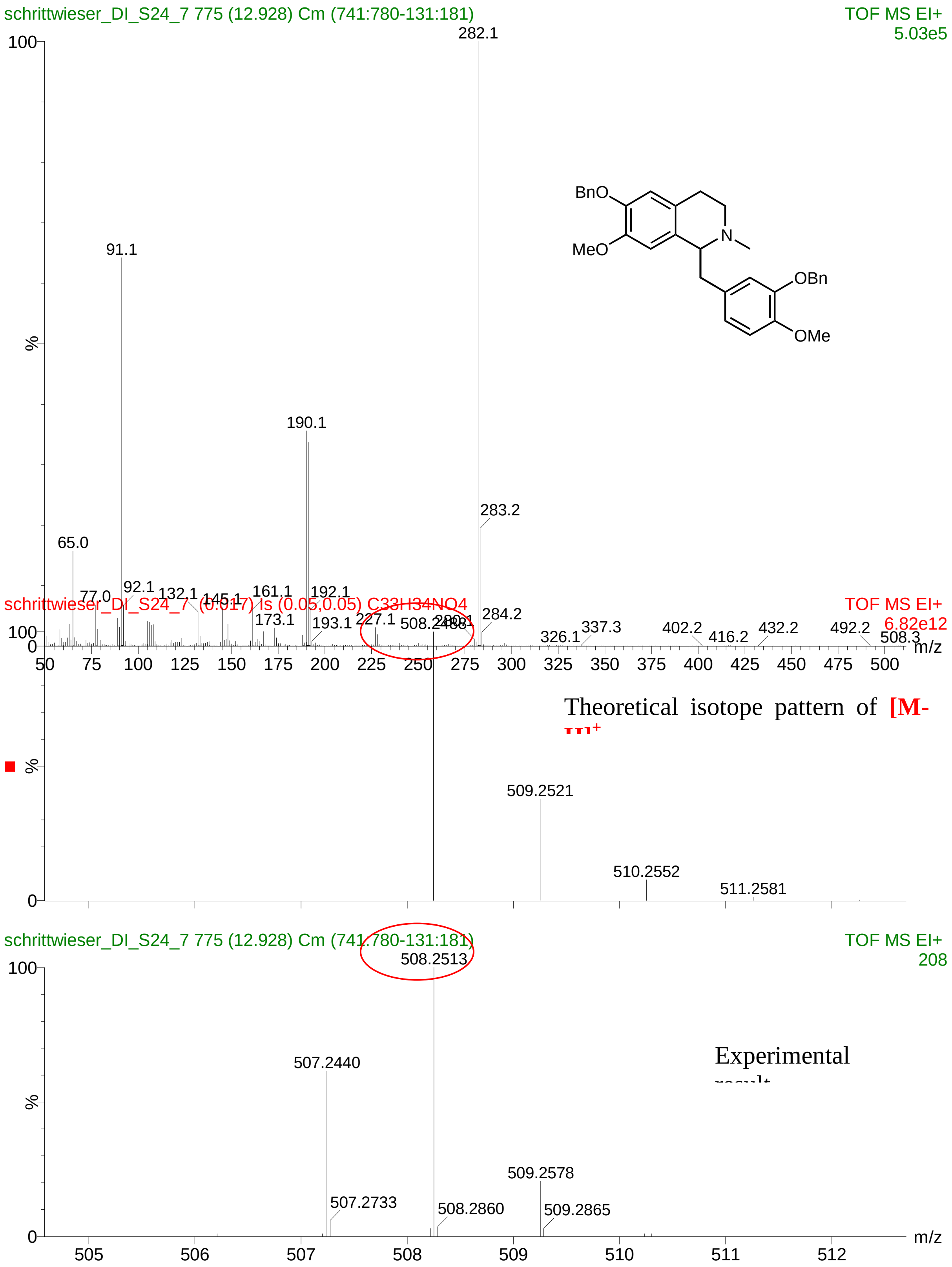

1-(3-Hydroxy-4-methoxybenzyl)-7-methoxy-2-methyl-1,2,3,4-tetrahydroisoquinolin-6-ol

^{1}H-NMR

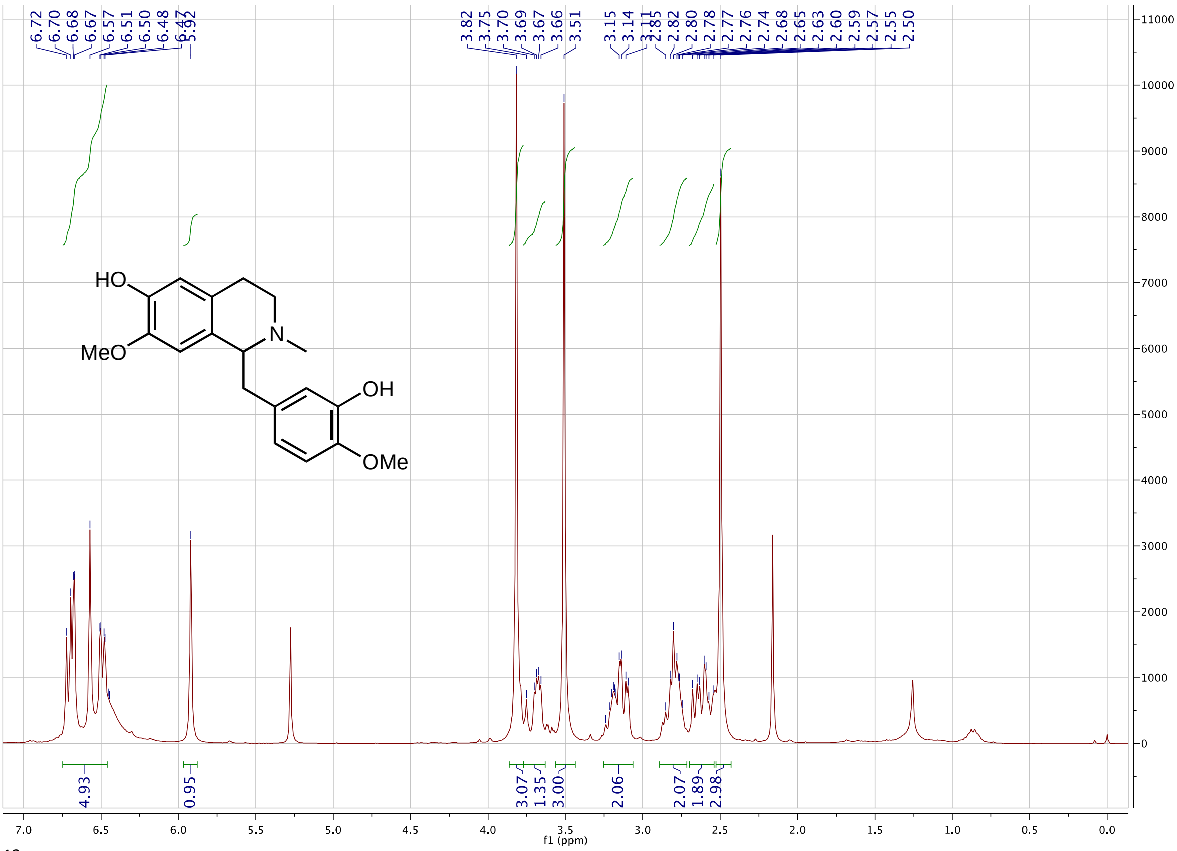

^{13}C-NMR

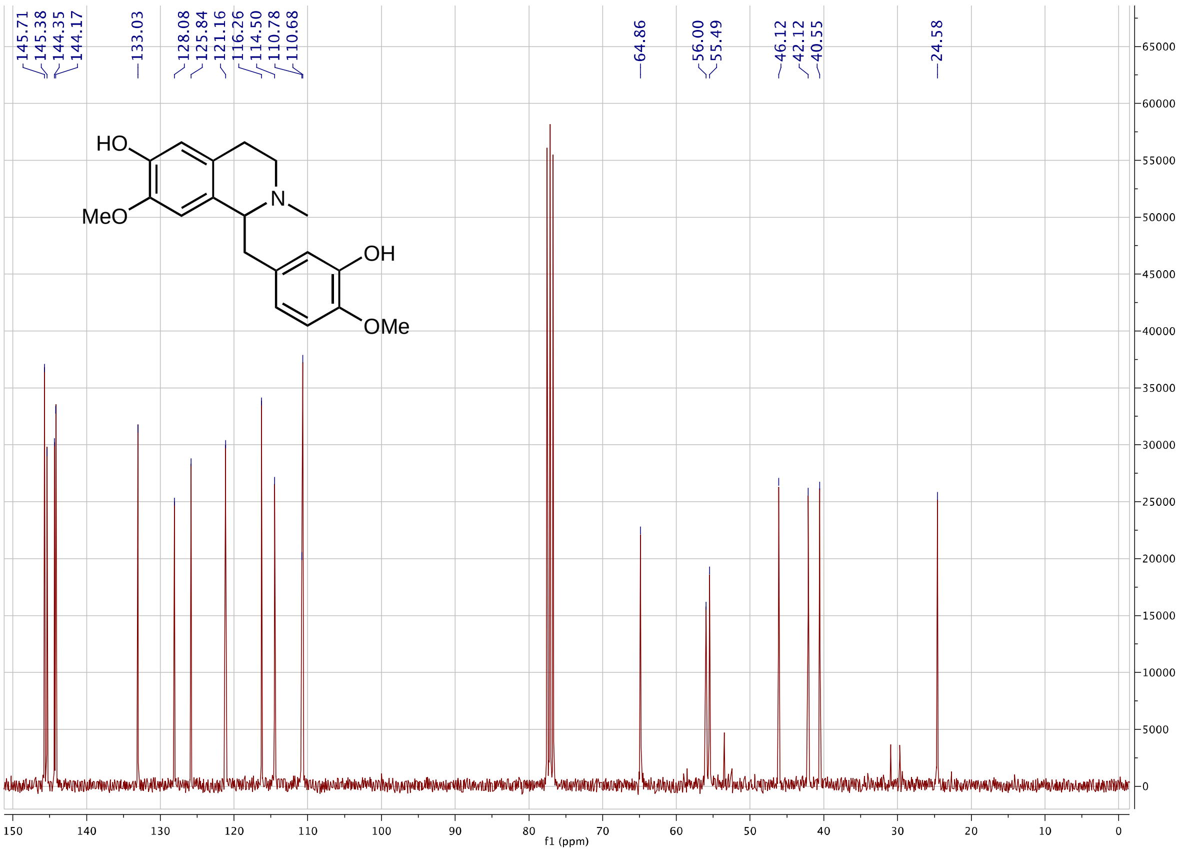

COSY spectrum

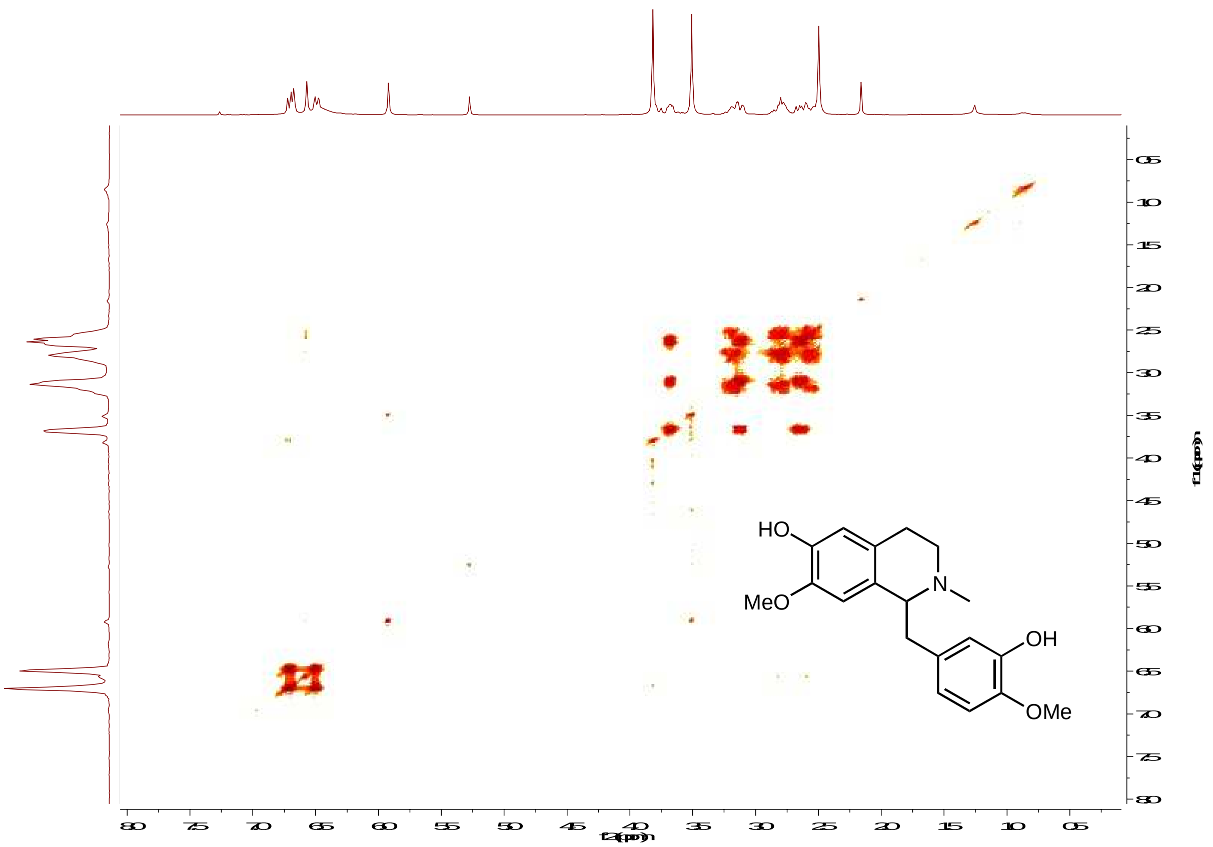

HSQC spectrum

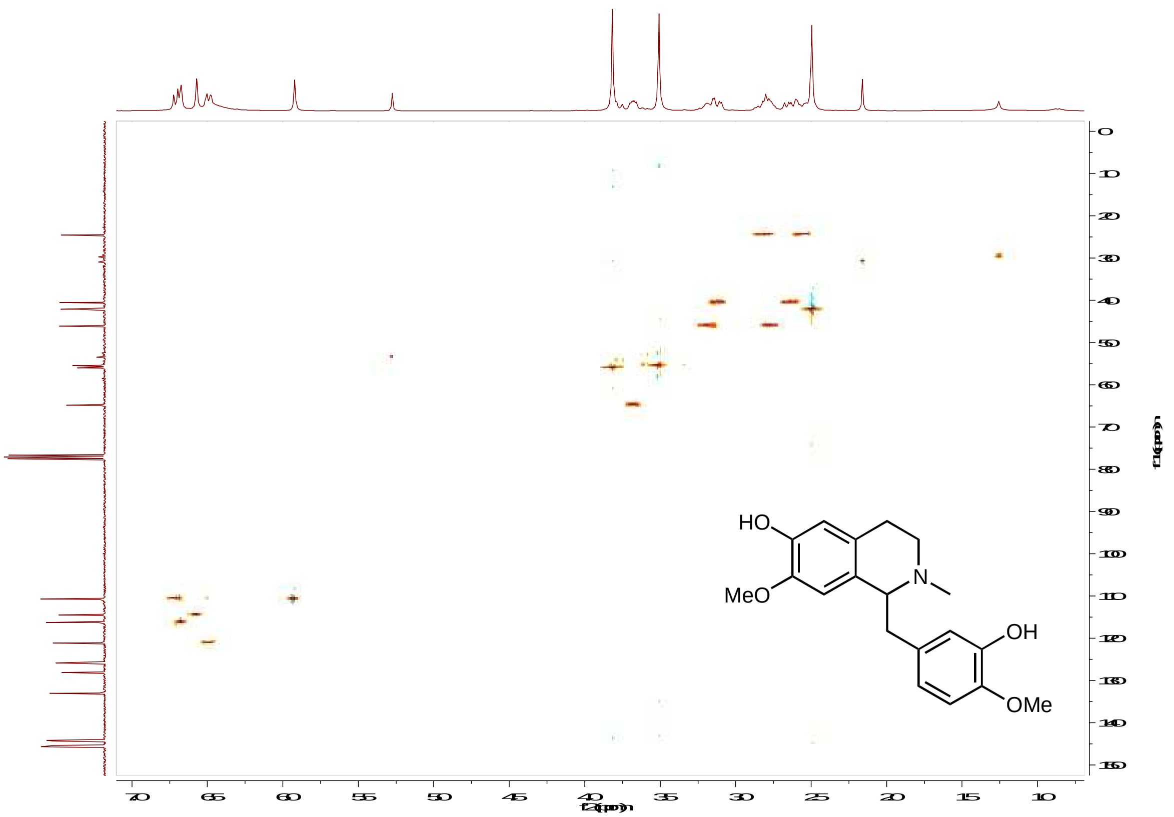

HRMS results

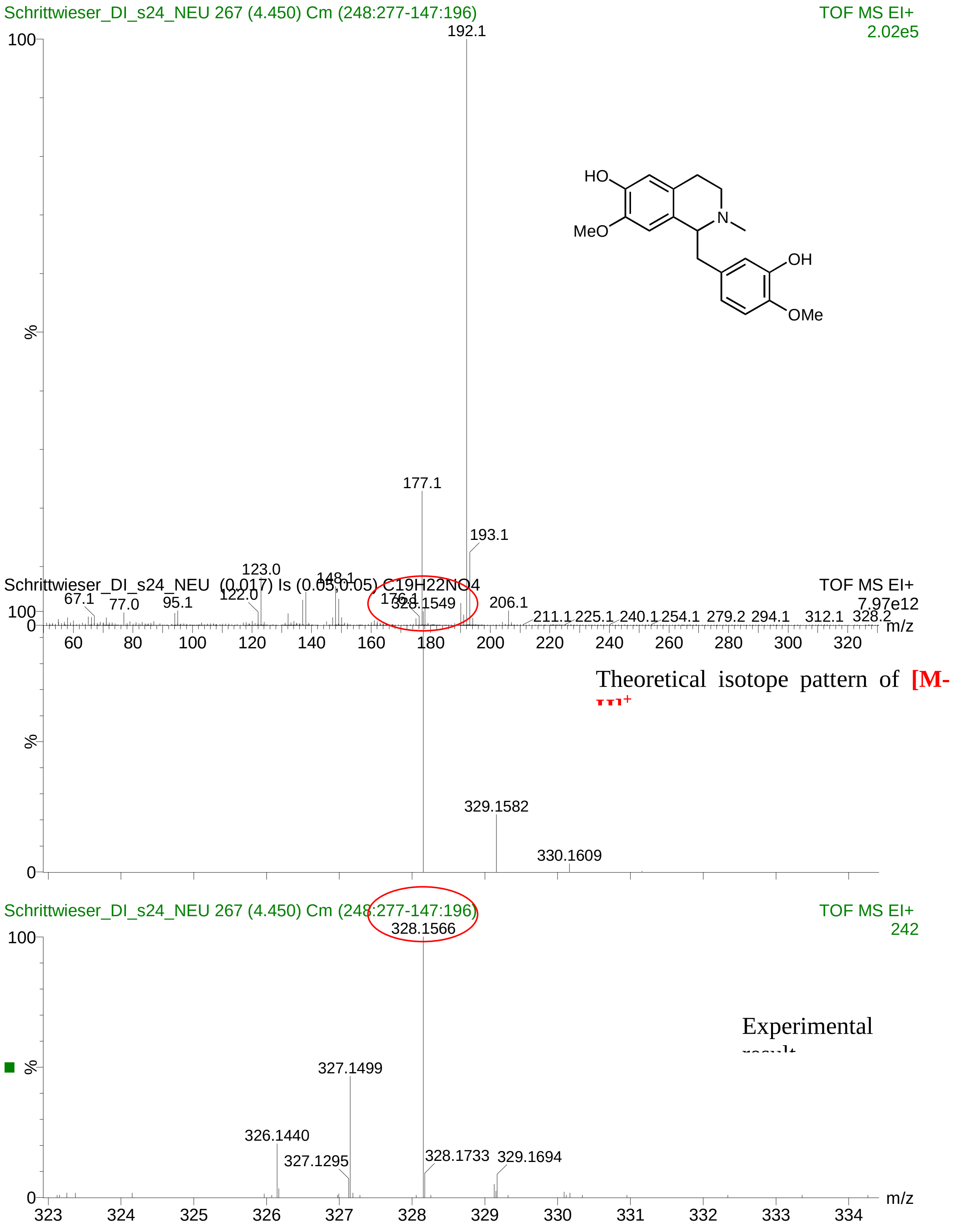

Synthesis of **1j**:

Provided Material:

Methyl (3,5-bisbenzyloxy)phenylacetate
^{1}H-NMR spectrum, ^{13}C-NMR spectrum

(3,5-Bisbenzyloxy)phenylacetic acid
^{1}H-NMR spectrum, ^{13}C-NMR spectrum

2-(3,5-Bisbenzyloxyphenyl)-*N*-(3,4-dimethoxyphenethyl)-*N*-methylacetamide
^{1}H-NMR spectrum, ^{13}C-NMR spectrum, ^{13}C-NMR DEPT135 spectrum, ^{13}C-NMR DEPT90 spectrum, COSY spectrum, HSQC spectrum, HRMS results

1-(3,5-Bisbenzyloxy)benzyl-6,7-dimethoxy-2-ethyl-1,2,3,4-tetrahydroisoquinoline
^{1}H-NMR spectrum, ^{13}C-NMR spectrum, HRMS results

1-(3,5-Dihydroxybenzyl)-6,7-dimethoxy-2-ethyl-1,2,3,4-tetrahydroisoquinoline
^{1}H-NMR spectrum, ^{13}C-NMR spectrum, HRMS results

Methyl (3,5-bisbenzyloxy)phenylacetate

^{1}H-NMR spectrum

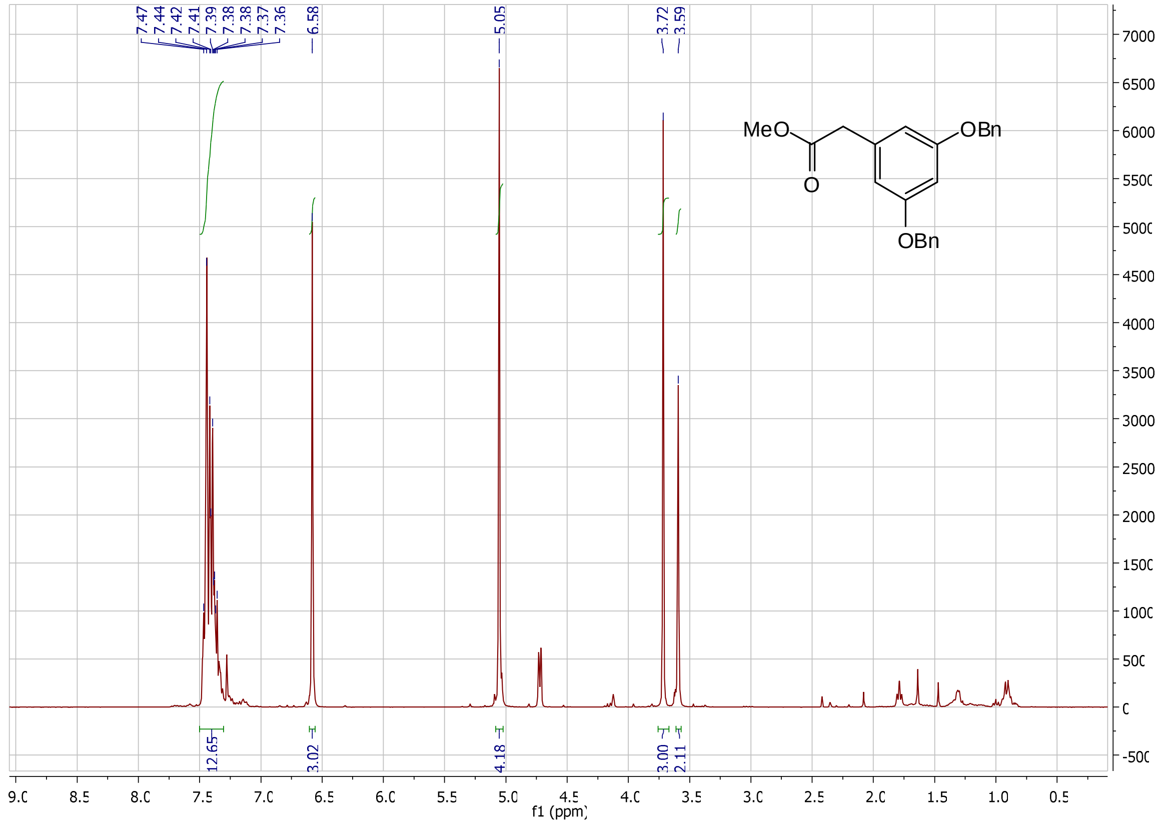

^{13}C-NMR spectrum

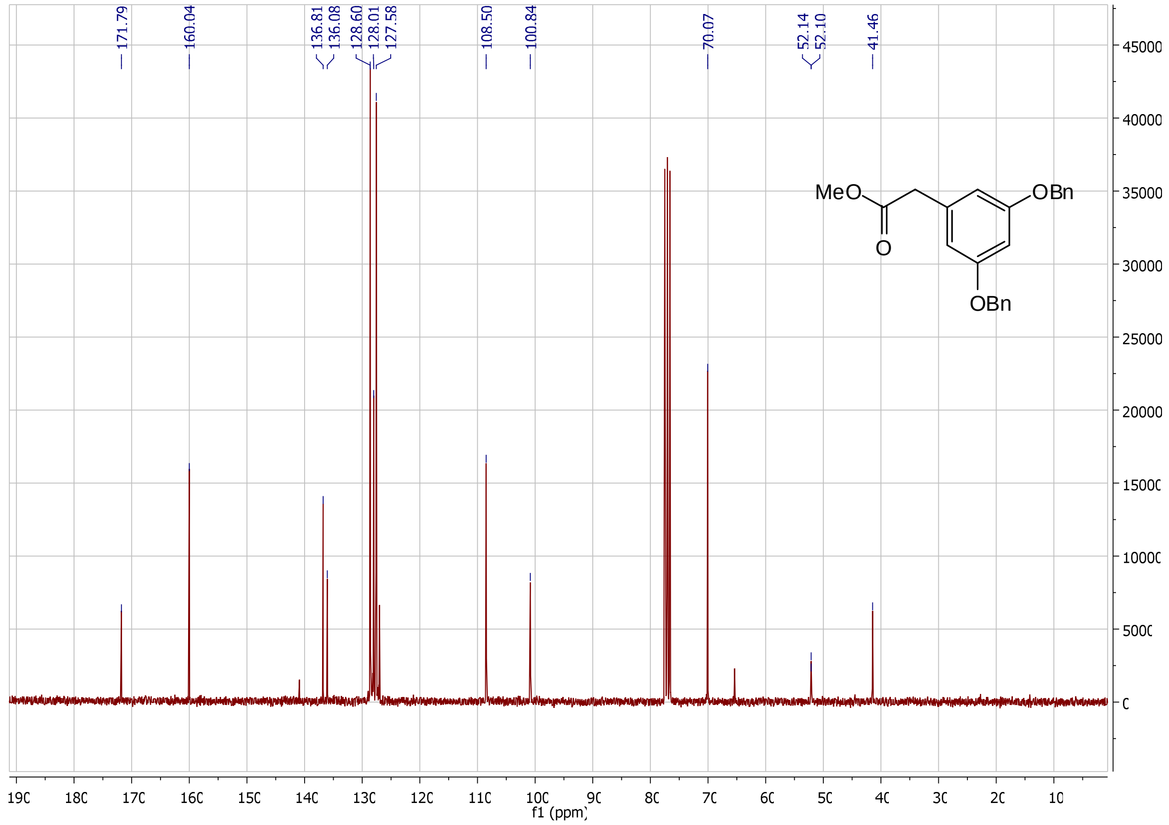

(3,5-Bisbenzyloxy)phenylacetic acid

^{1}H-NMR spectrum

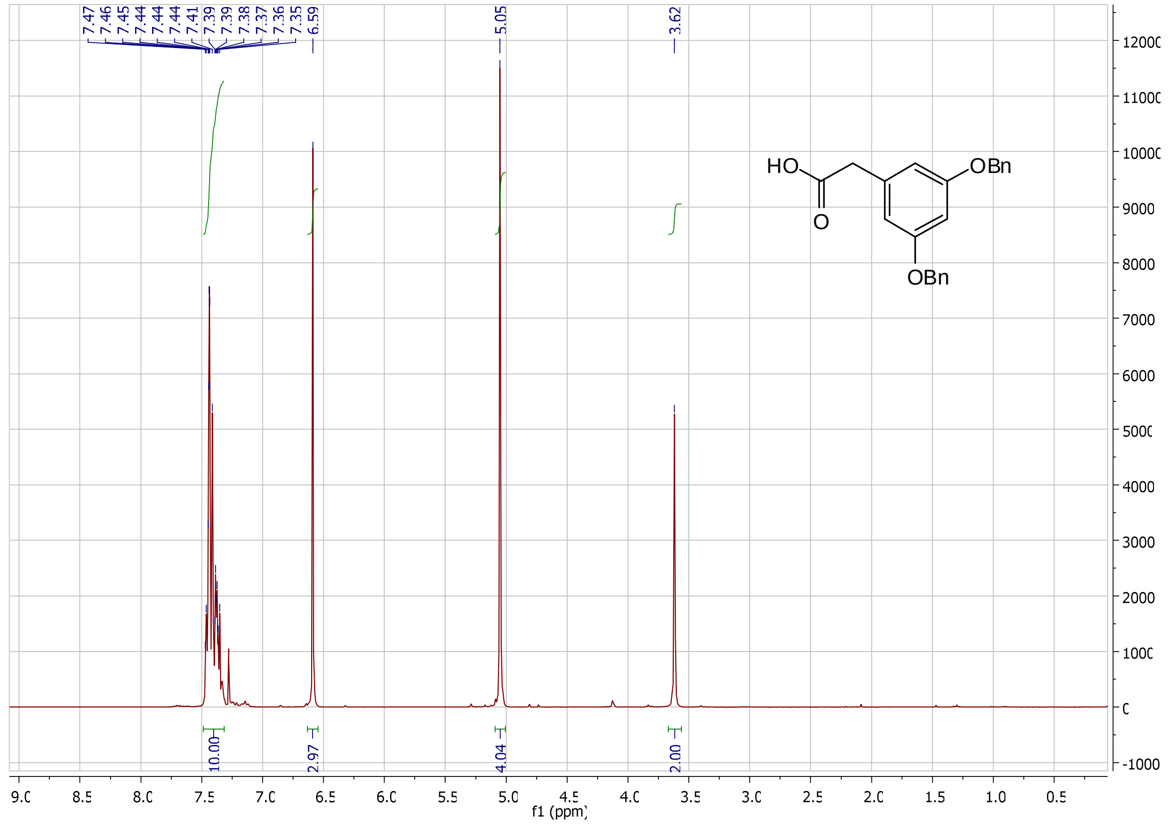

^{13}C-NMR spectrum

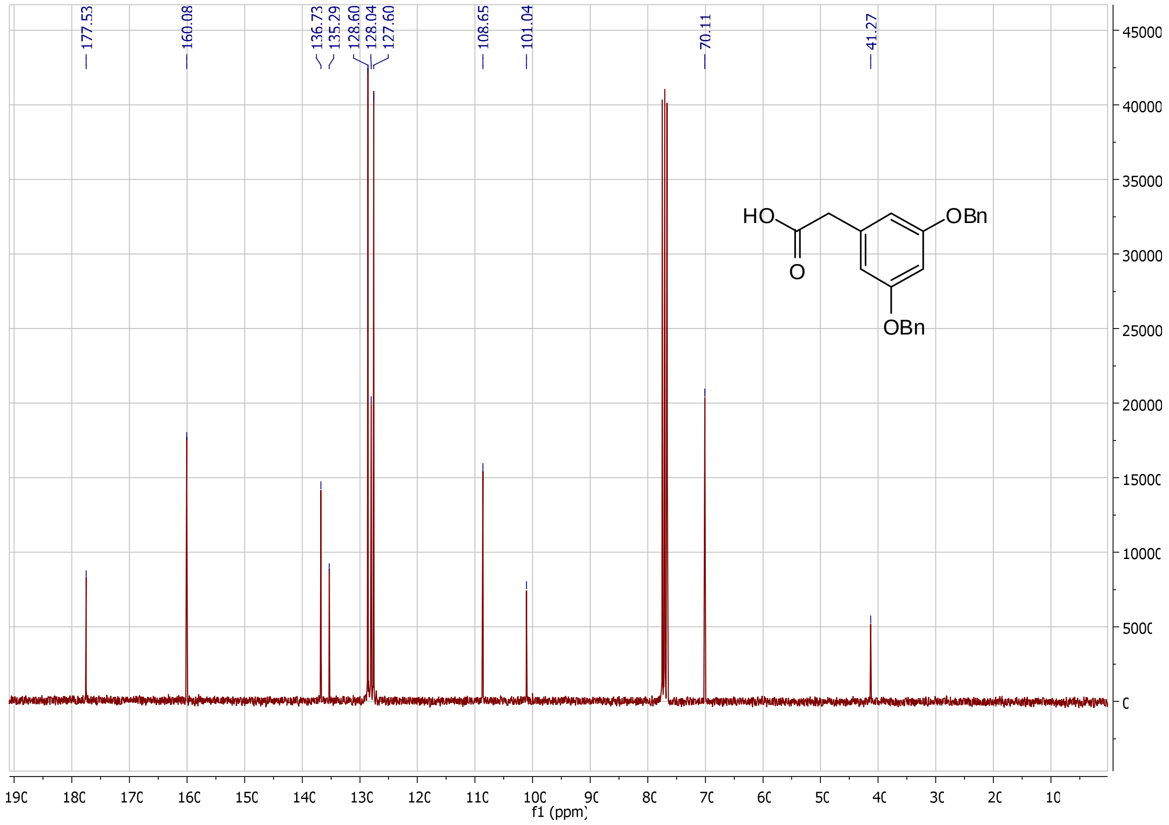

2-(3,5-Bisbenzyloxyphenyl)-*N*-(3,4-dimethoxyphenethyl)-*N*-methylacetamide

^{1}H-NMR spectrum

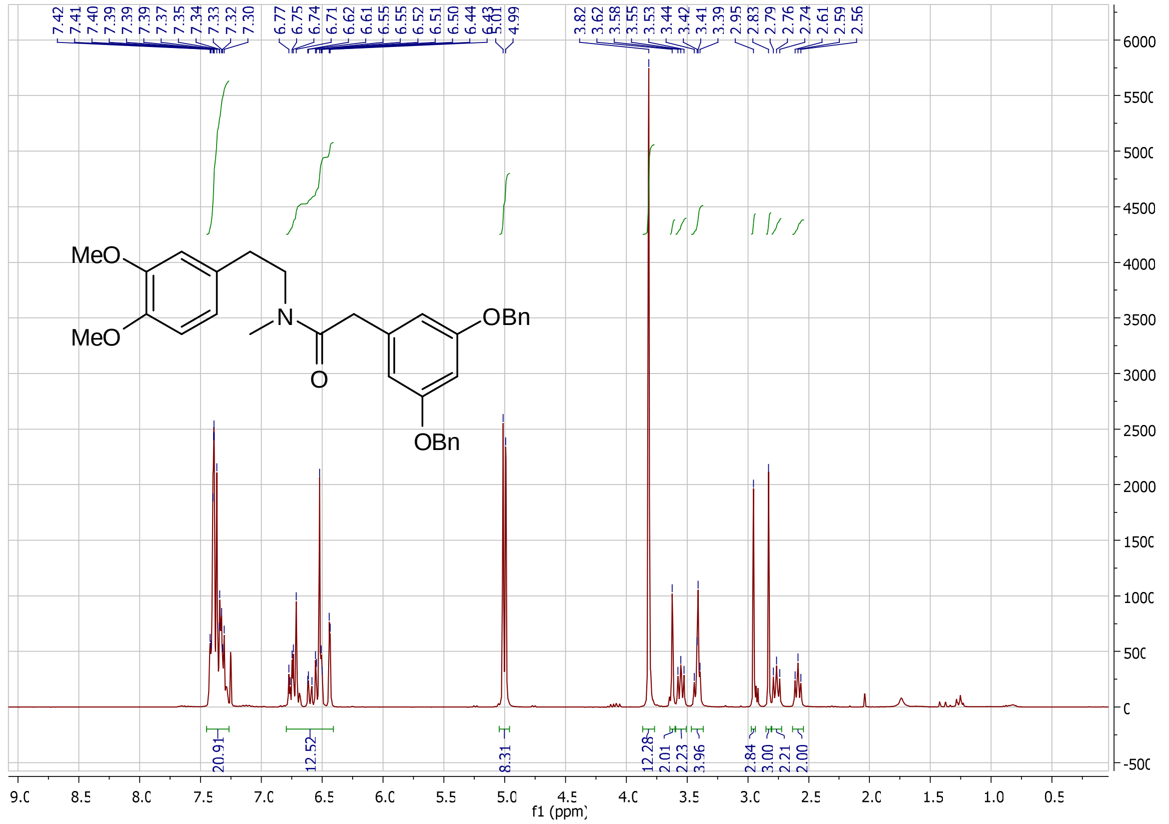

^{13}C-NMR spectrum

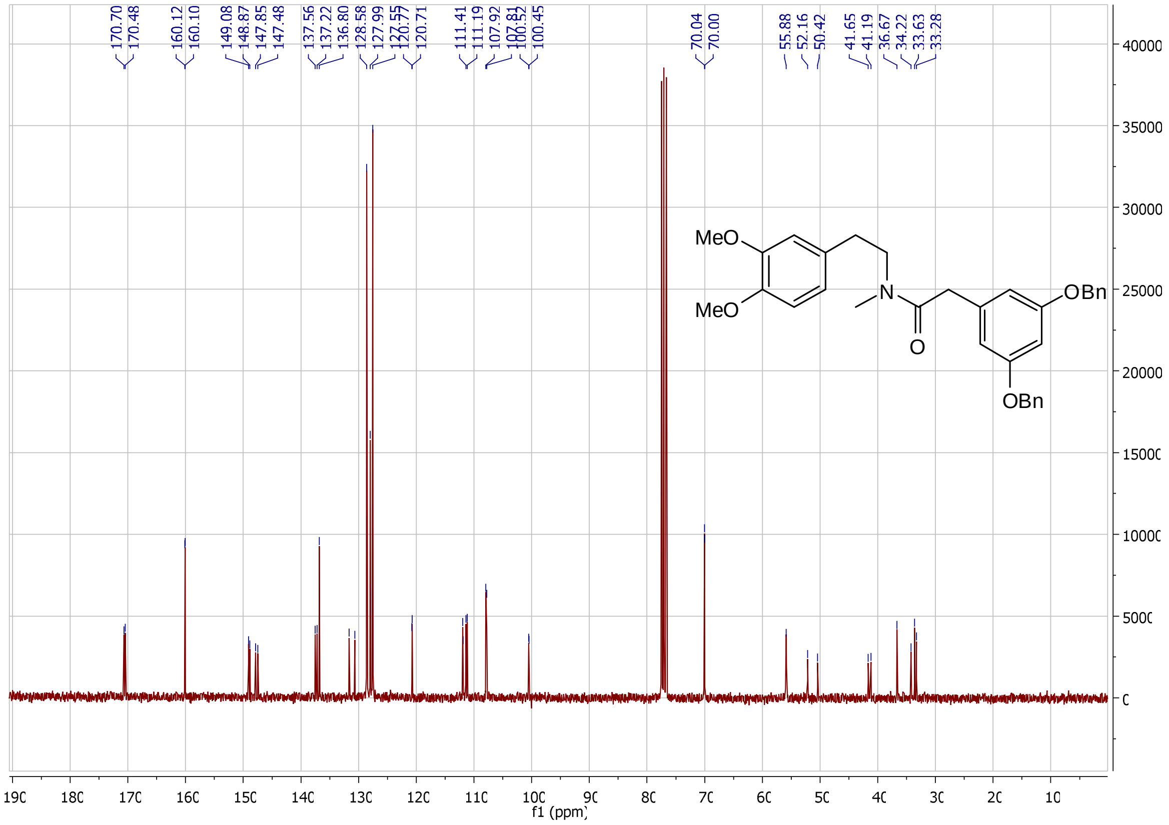

^{13}C-NMR DEPT135 spectrum

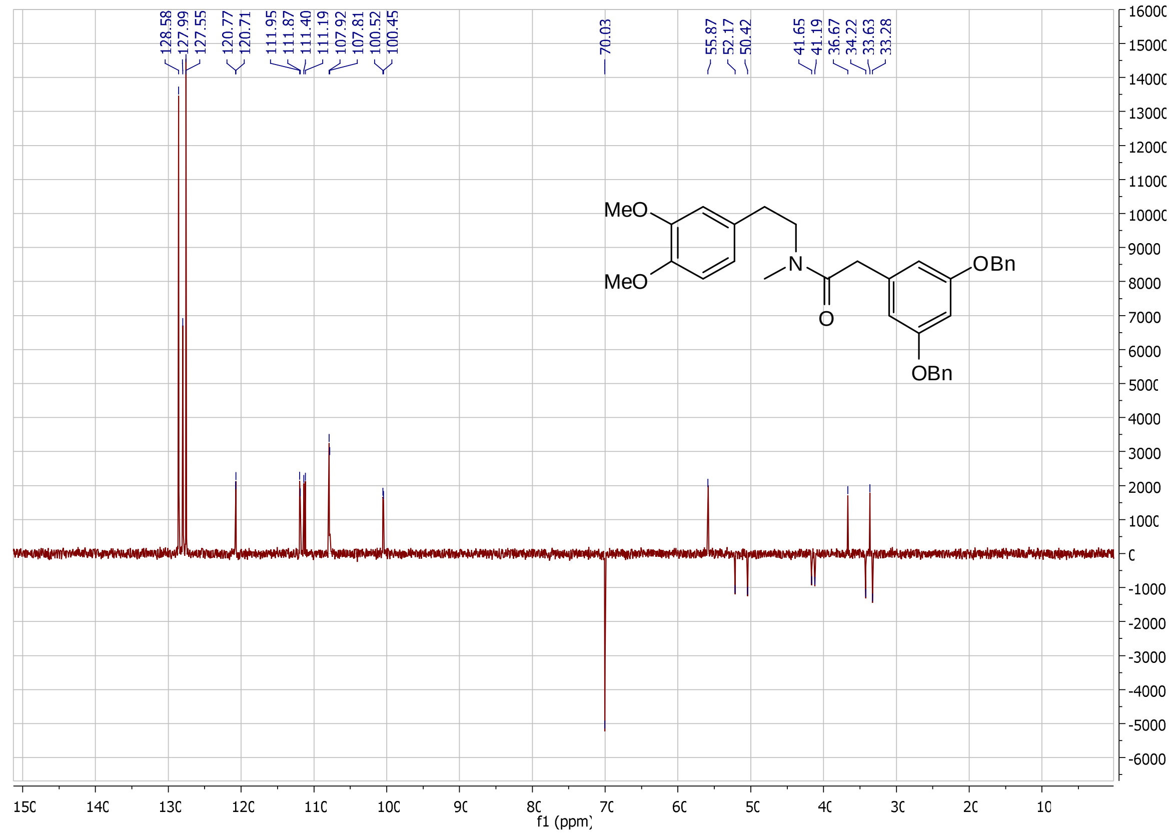

^{13}C-NMR DEPT90 spectrum

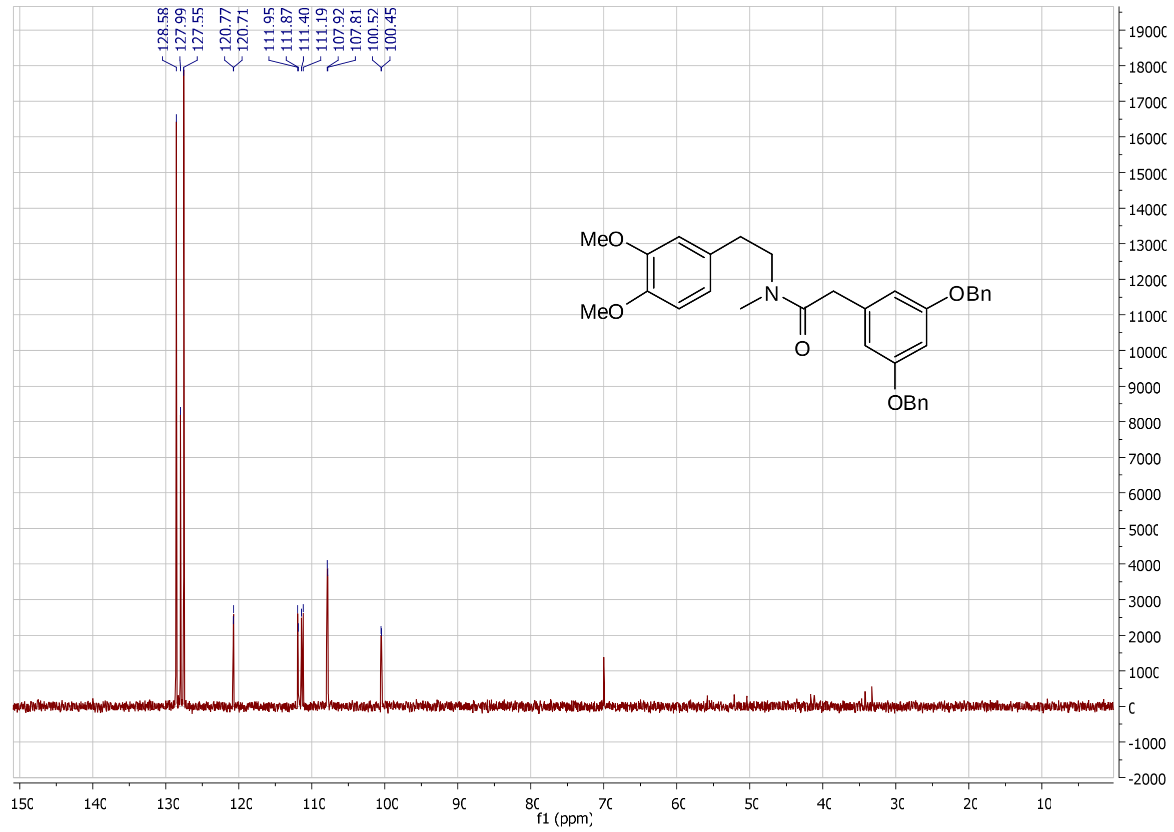

COSY spectrum

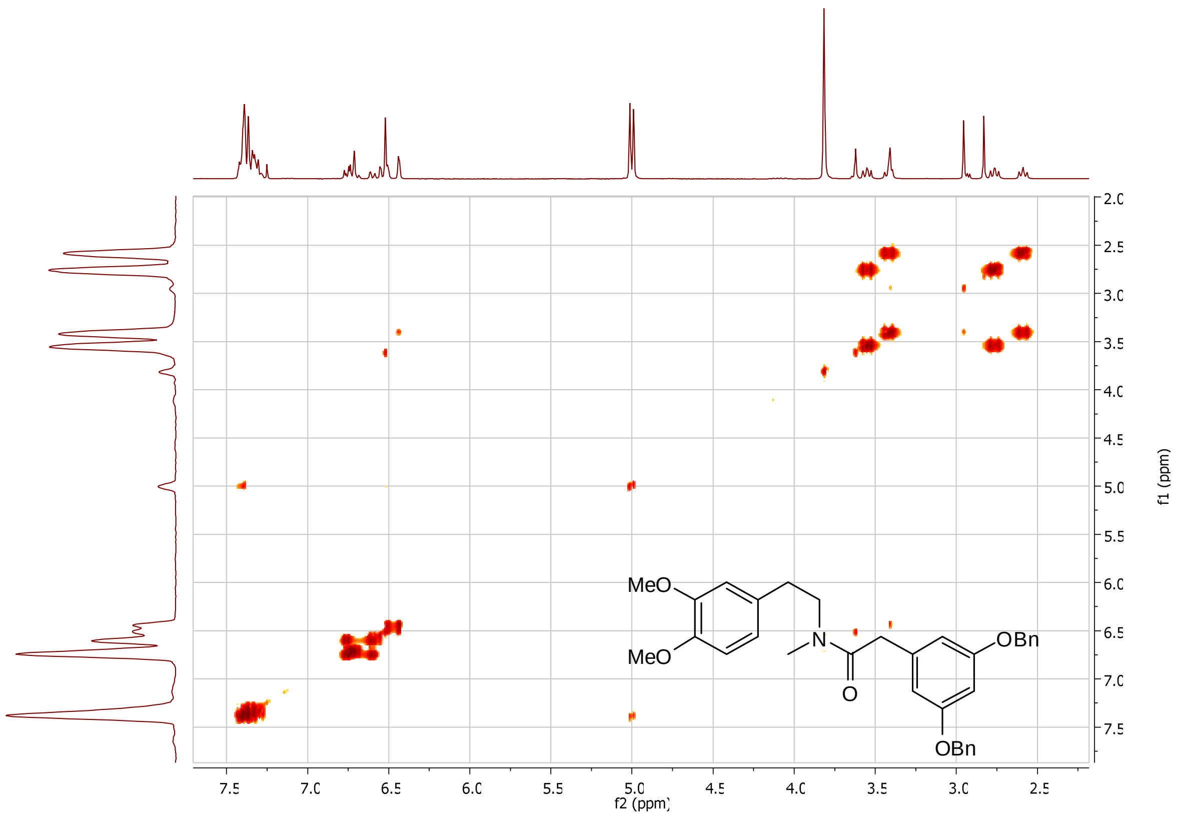

HSQC spectrum

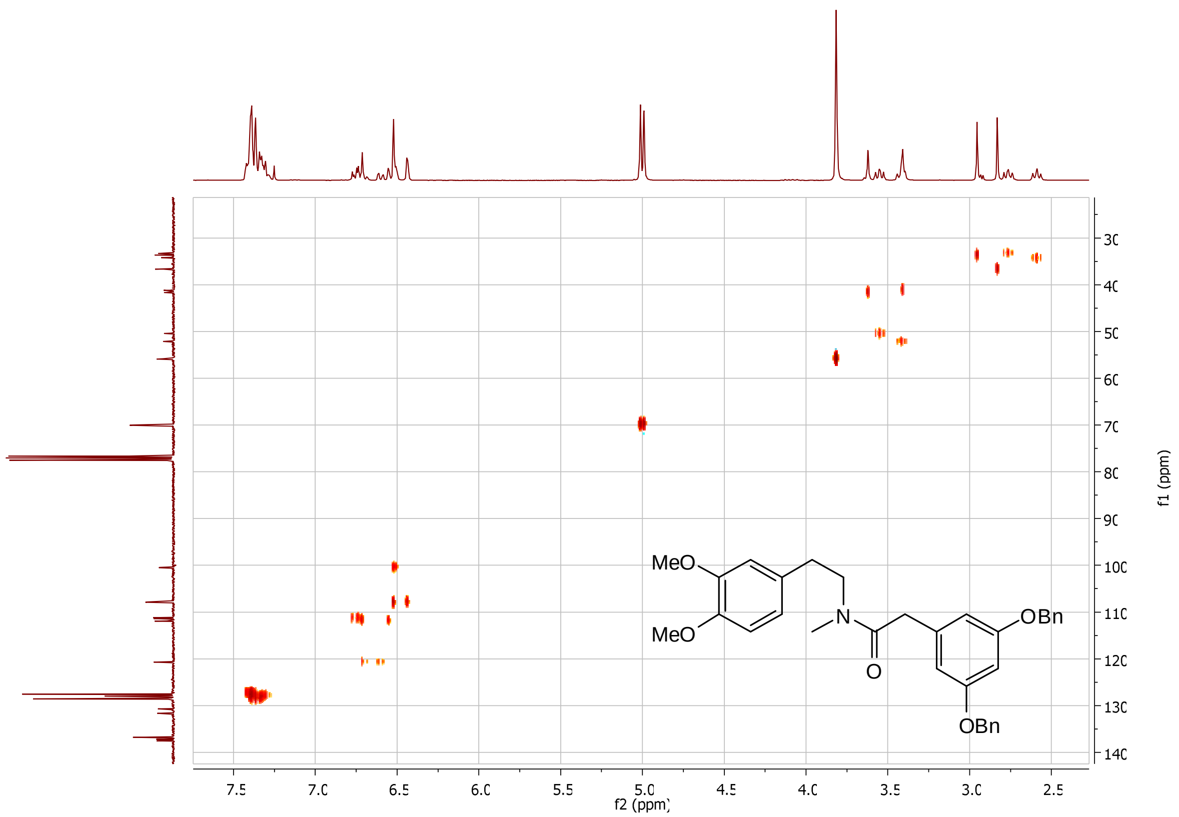

HRMS results

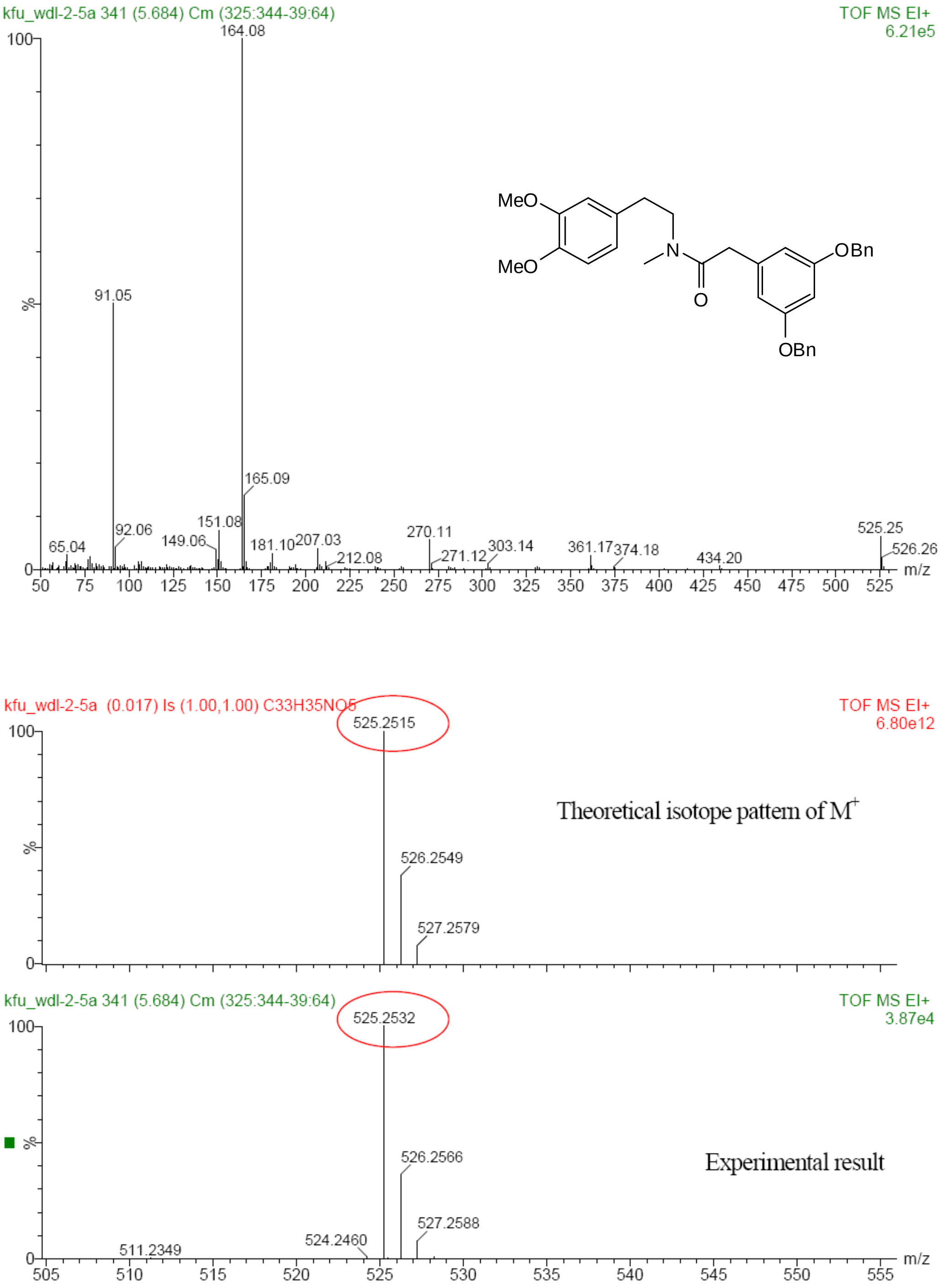

^{1}H-NMR spectrum

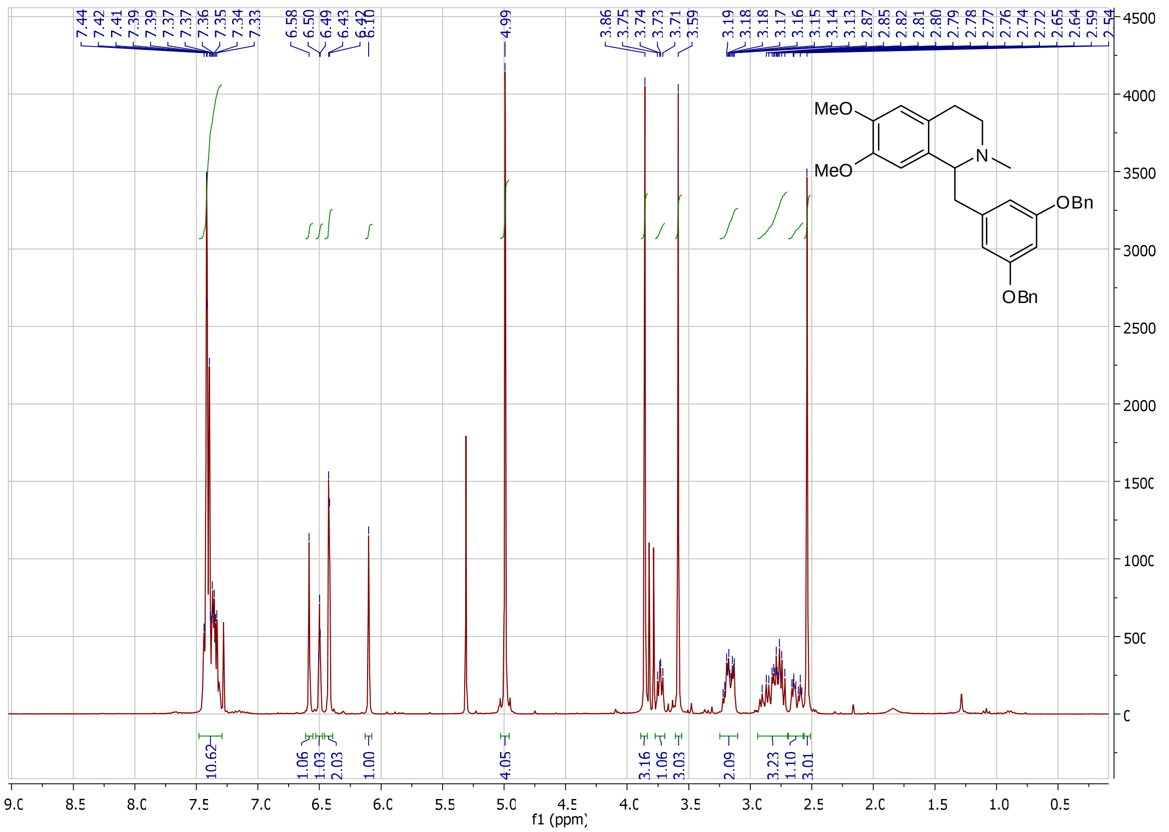

^{13}C-NMR spectrum

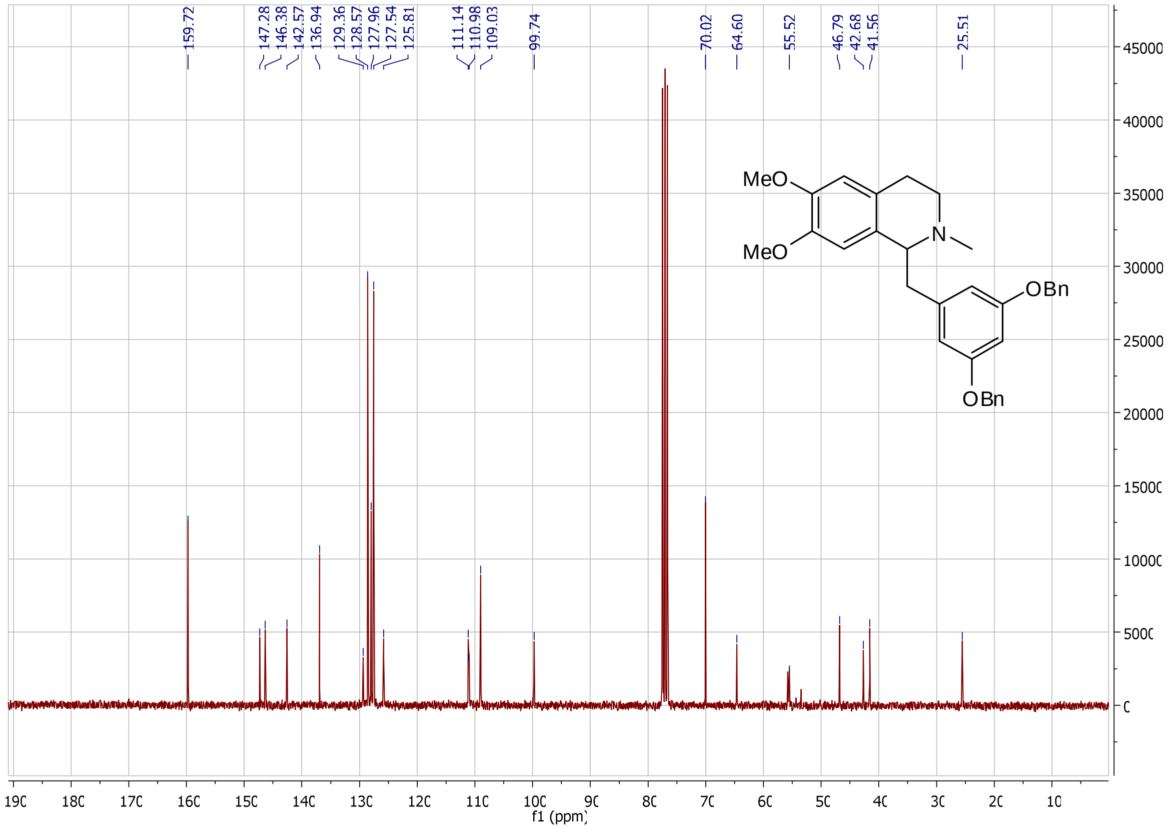

HRMS results

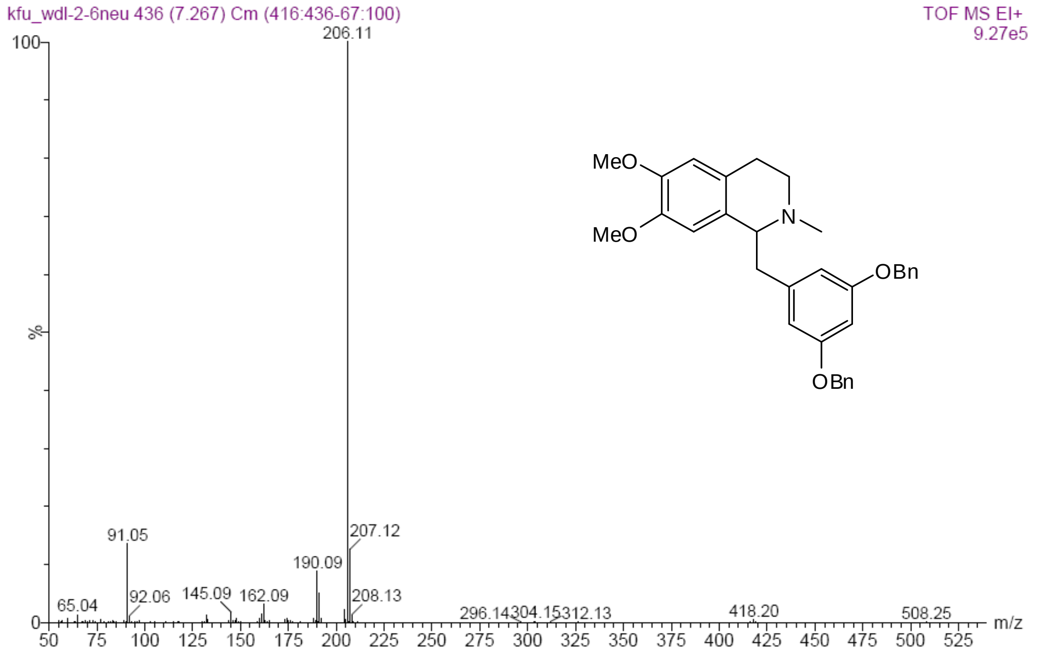

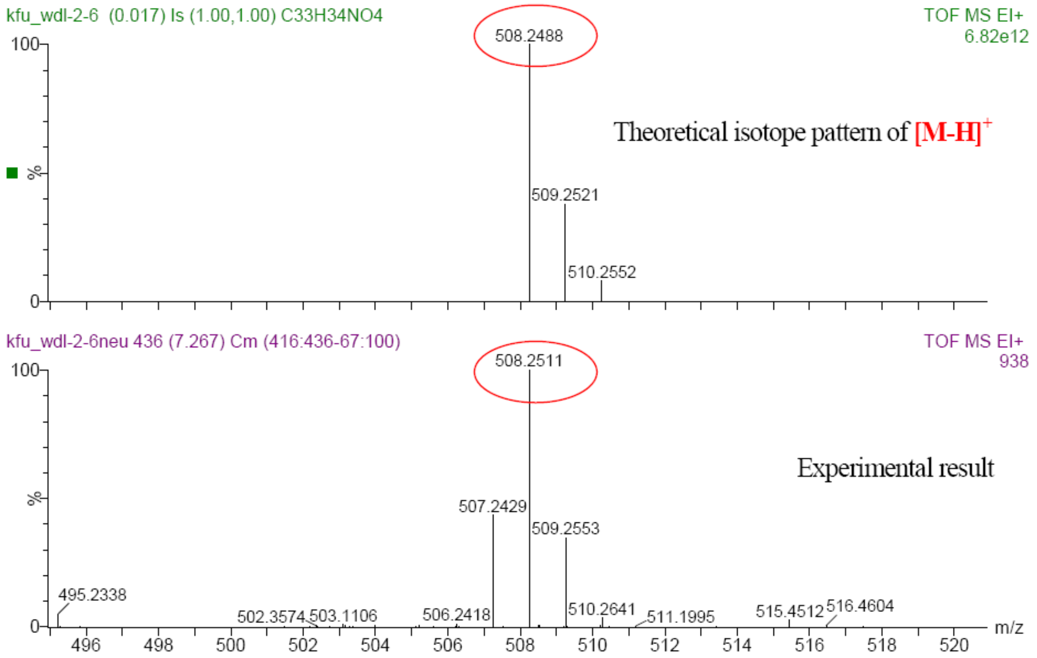

1-(3,5-Dihydroxybenzyl)-6,7-dimethoxy-2-ethyl-1,2,3,4-tetrahydroisoquinoline (2j):

^{1}H-NMR spectrum

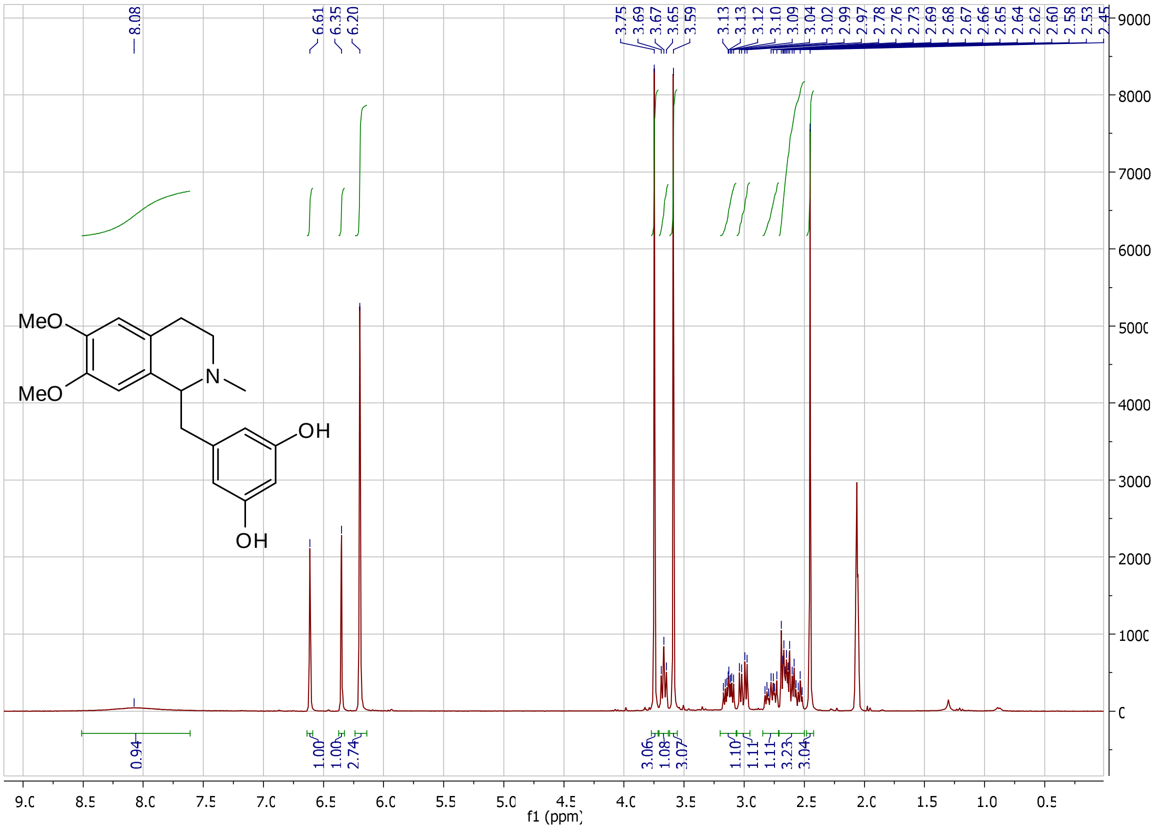

^{13}C-NMR spectrum

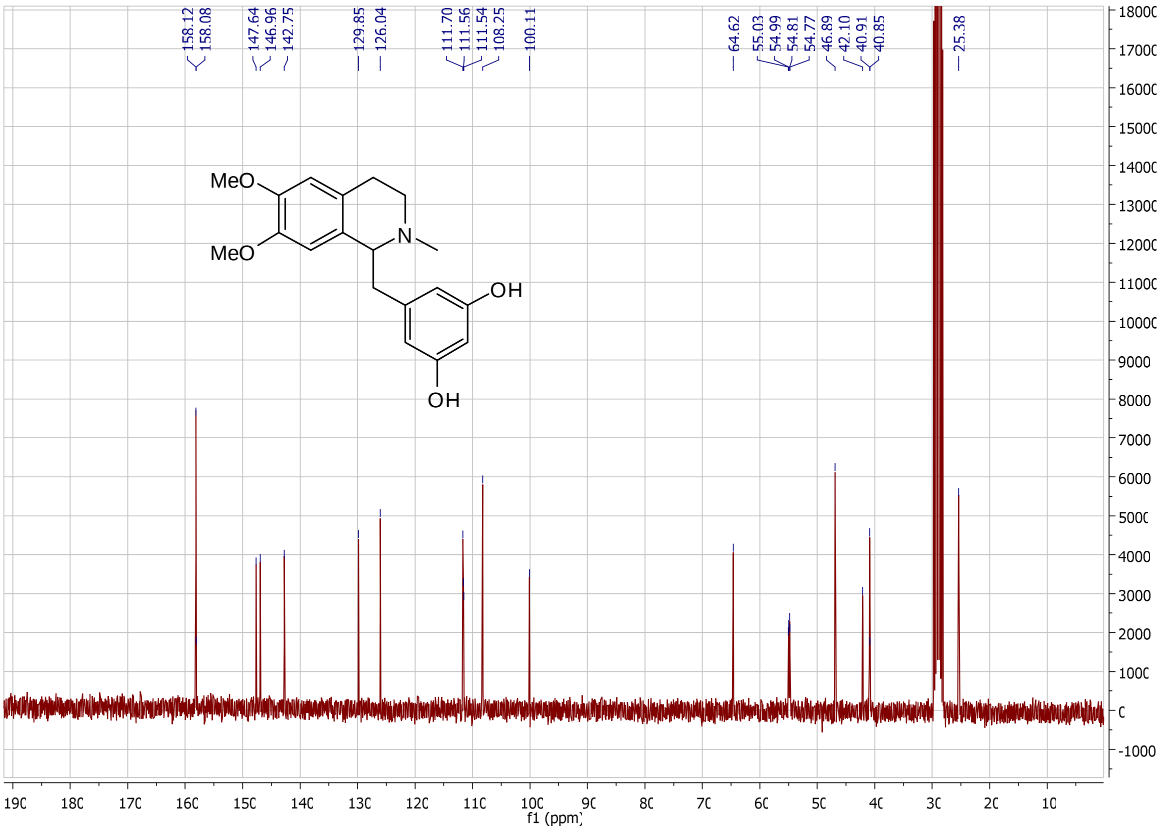

COSY spectrum

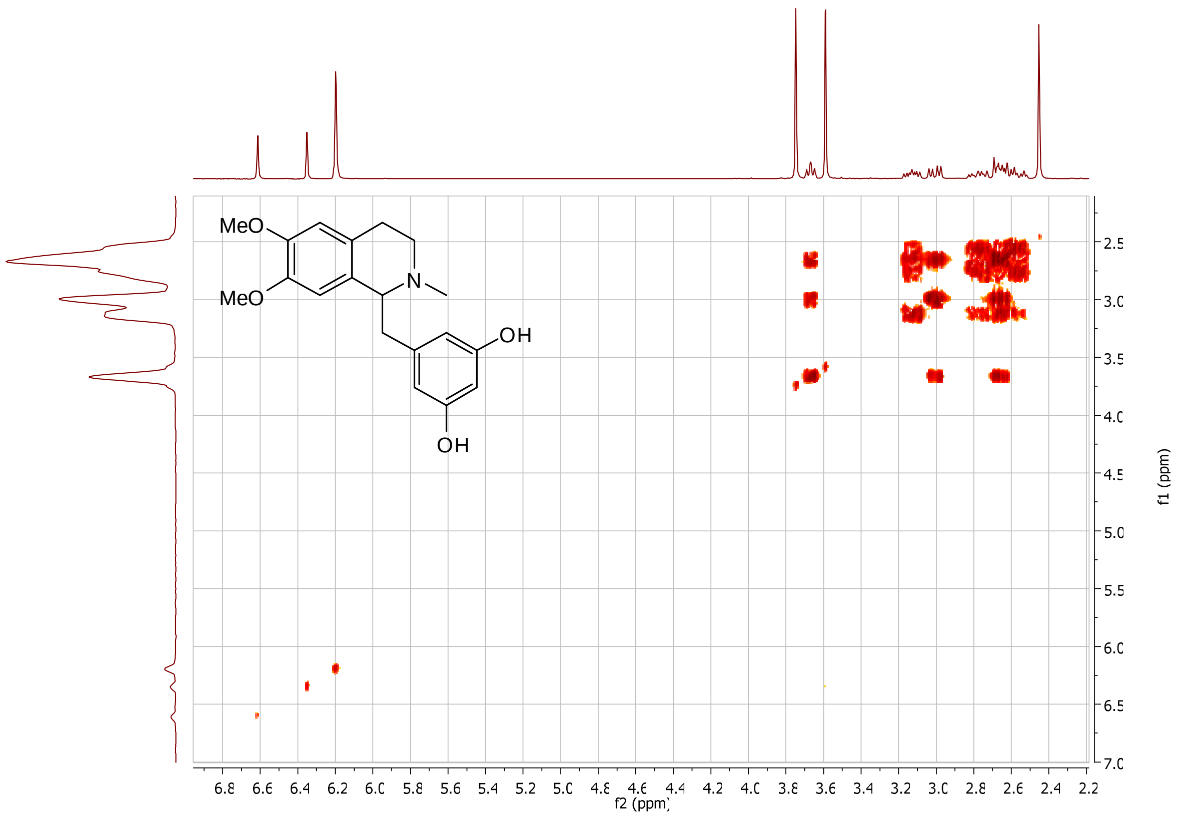

HSQC spectrum

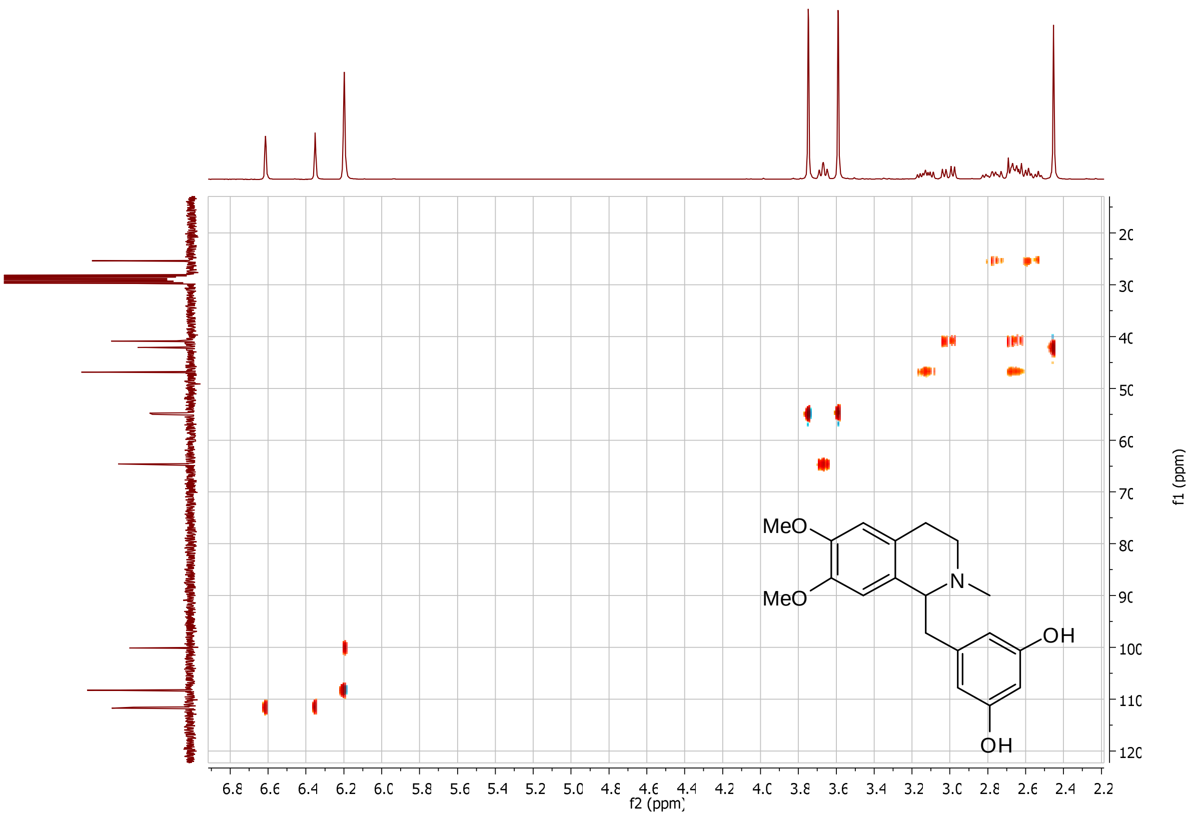

HRMS results

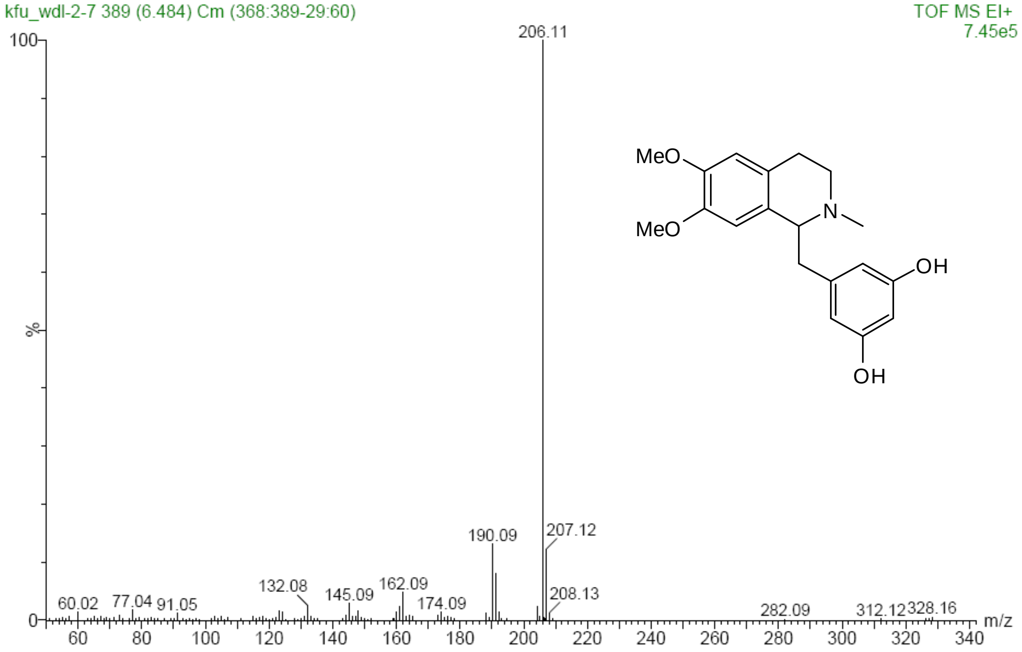

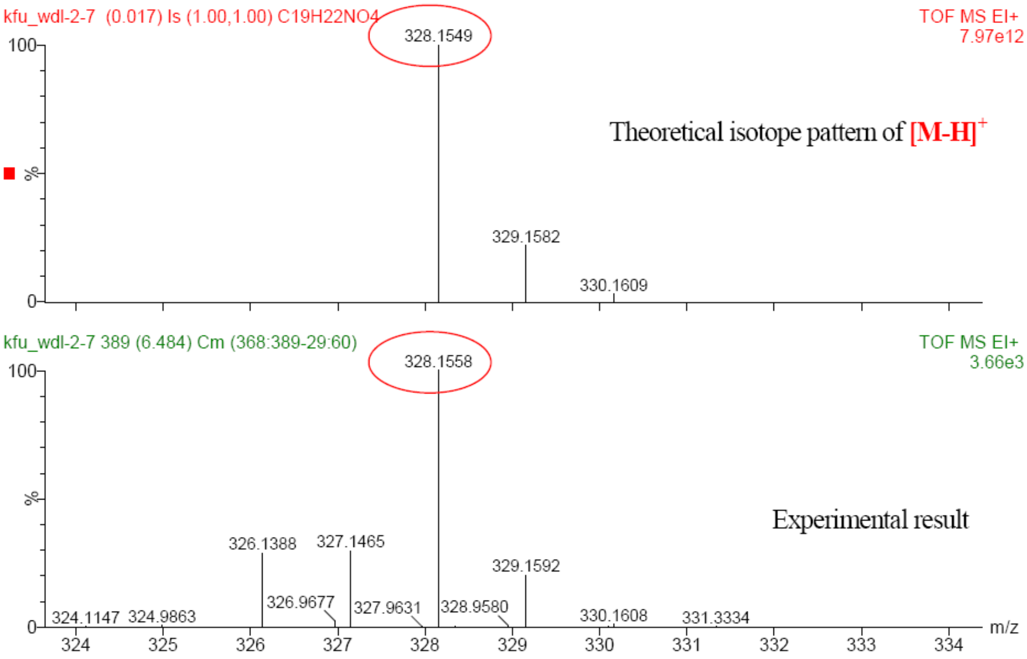

Synthesis of **1k**:

Provided Material:

2,2,2-Trichloro-1-(2-fluoro-5-methoxyphenyl)ethanol
^{1}H-NMR spectrum, ^{13}C-NMR spectrum, HRMS results

2-(2-Fluoro-5-methoxyphenyl)acetic acid
^{1}H-NMR spectrum, ^{13}C-NMR spectrum

2-(2-Fluoro-5-hydroxyphenyl)acetic acid
^{1}H-NMR spectrum, ^{13}C-NMR spectrum

2-(5-(Benzyloxy)-2-fluorophenyl)acetic acid
^{1}H-NMR spectrum, ^{13}C-NMR spectrum

2-(5-(Benzyloxy)-2-fluorophenyl)-*N*-(3,4-dimethoxyphenethyl)-*N*-methylacetamide
^{1}H-NMR spectrum, ^{13}C-NMR spectrum, ^{13}C-NMR DEPT135 spectrum, ^{13}C-NMR DEPT90 spectrum, COSY spectrum, HSQC spectrum, HRMS results

1-(5-(Benzyloxy)-2-fluorobenzyl)-6,7-dimethoxy-2-methyl-1,2,3,4-tetrahydroisoquinoline
^{1}H-NMR spectrum, ^{13}C-NMR spectrum, HRMS results

3-((6,7-Dimethoxy-2-methyl-1,2,3,4-tetrahydroisoquinolin-1-yl)methyl)-4-fluorophenol
^{1}H-NMR spectrum, ^{13}C-NMR spectrum, COSY spectrum, HSQC spectrum, HRMS results

2,2,2-Trichloro-1-(2-fluoro-5-methoxyphenyl)ethanol

^{1}H-NMR

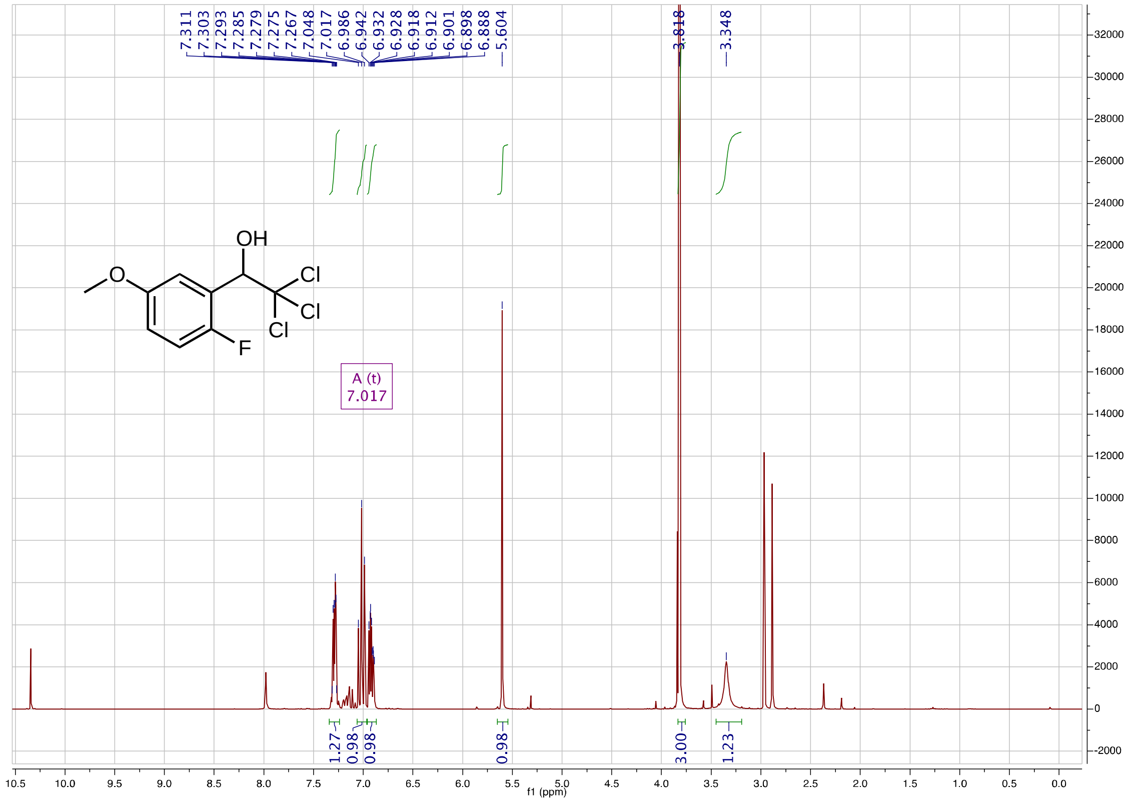

^{13}C-NMR

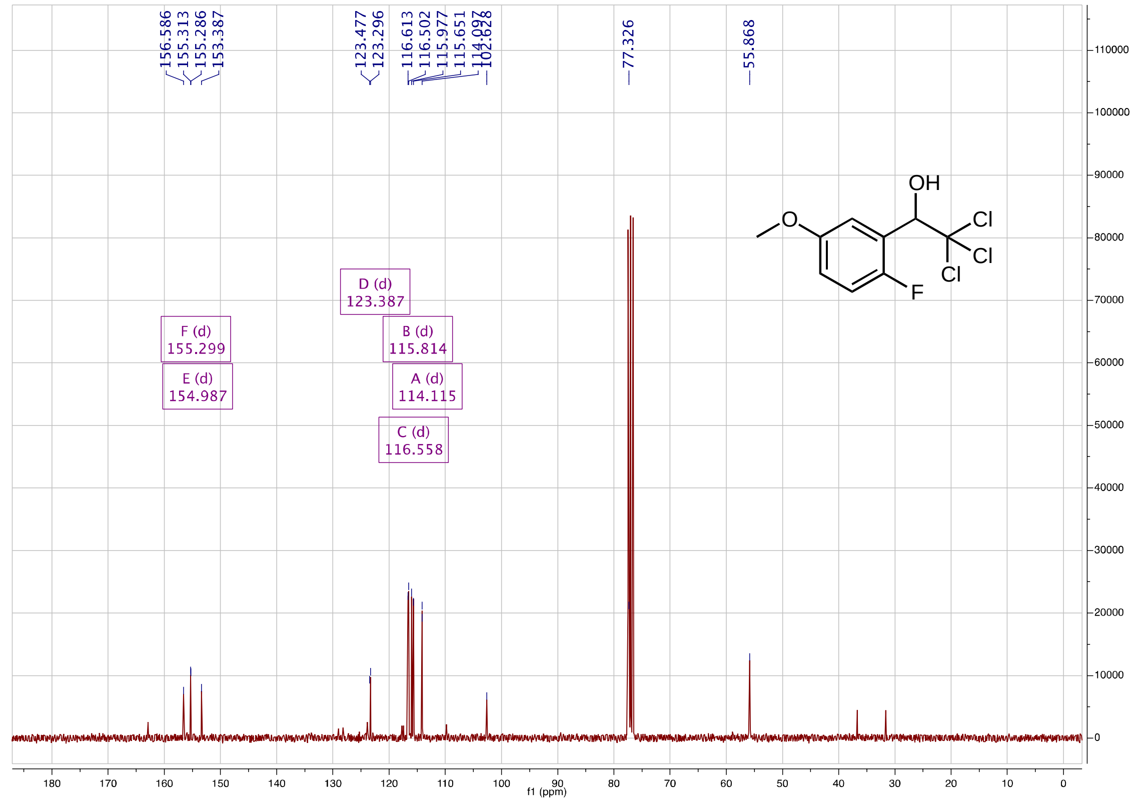

HRMS results

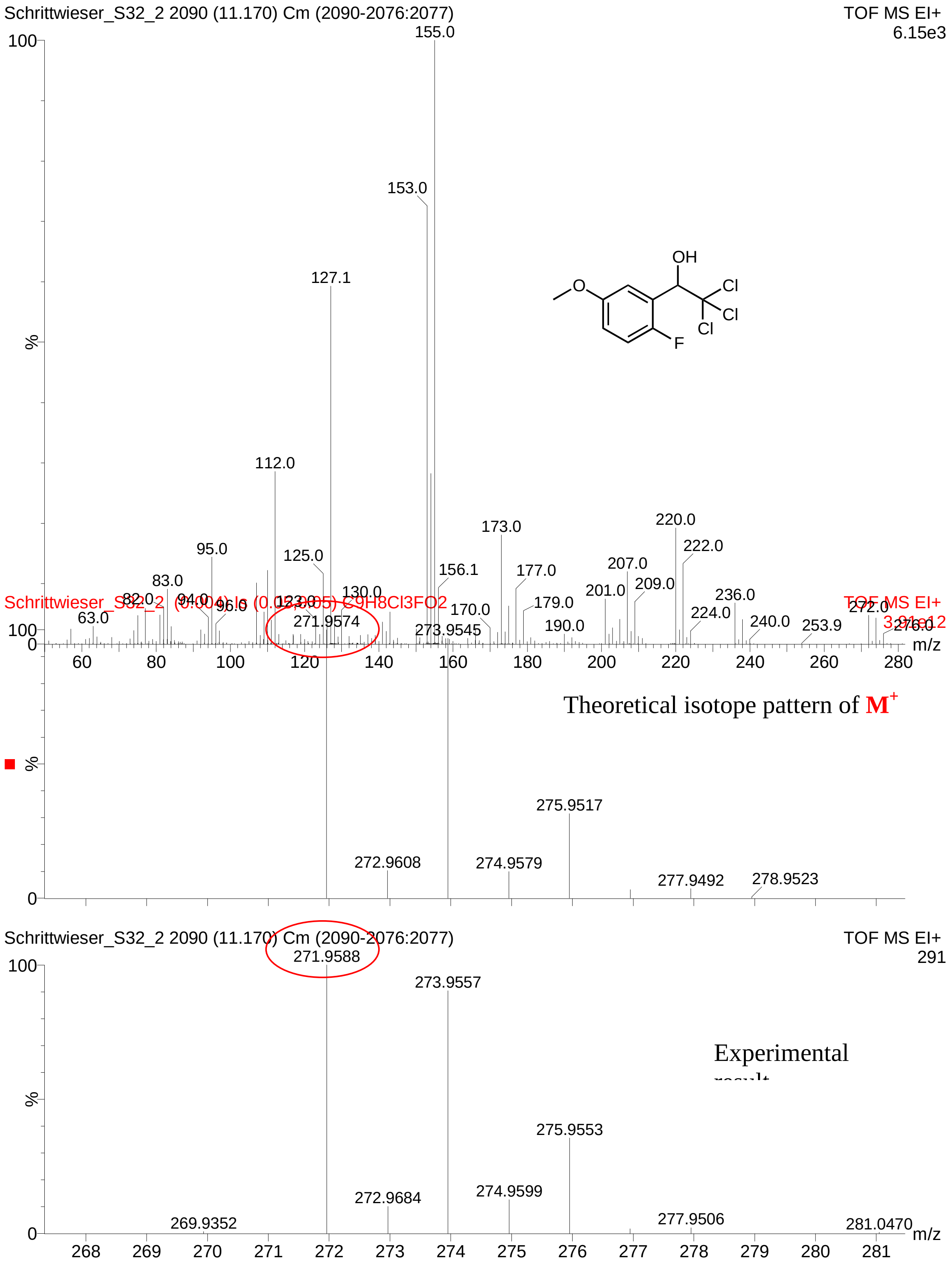

2-(2-Fluoro-5-methoxyphenyl)acetic acid

^{1}H-NMR

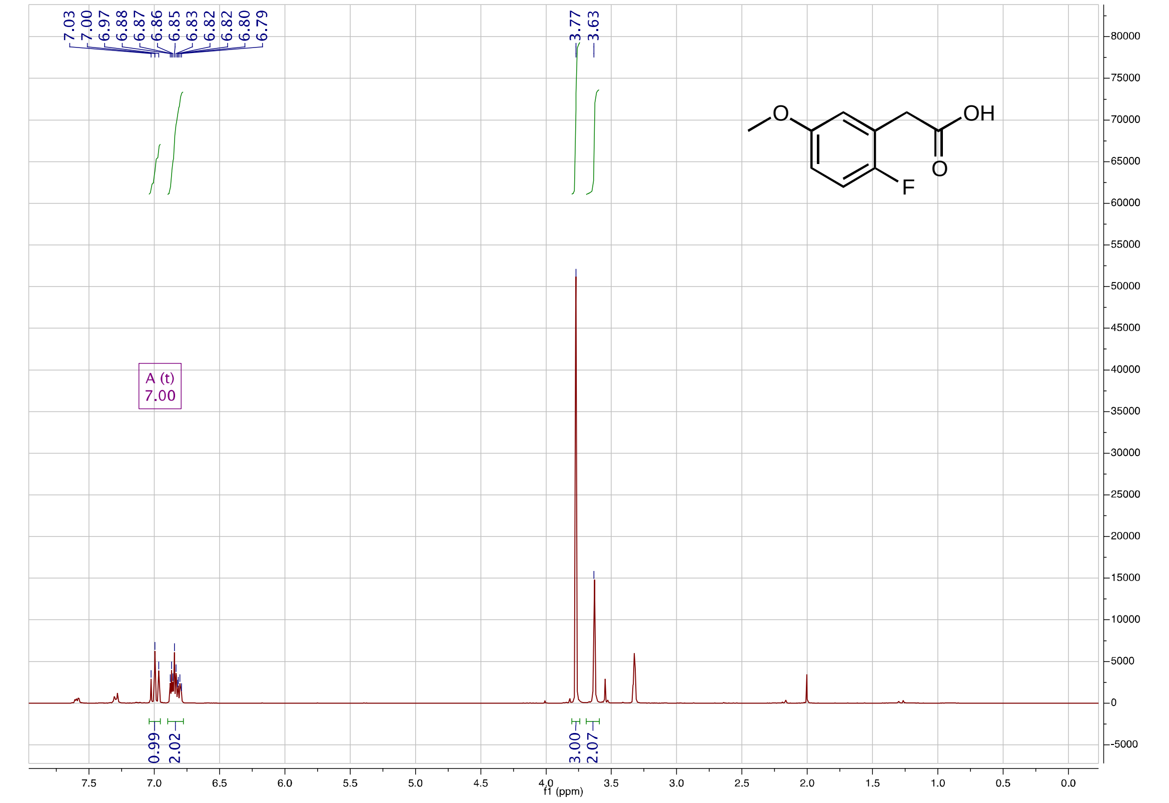

^{13}C-NMR

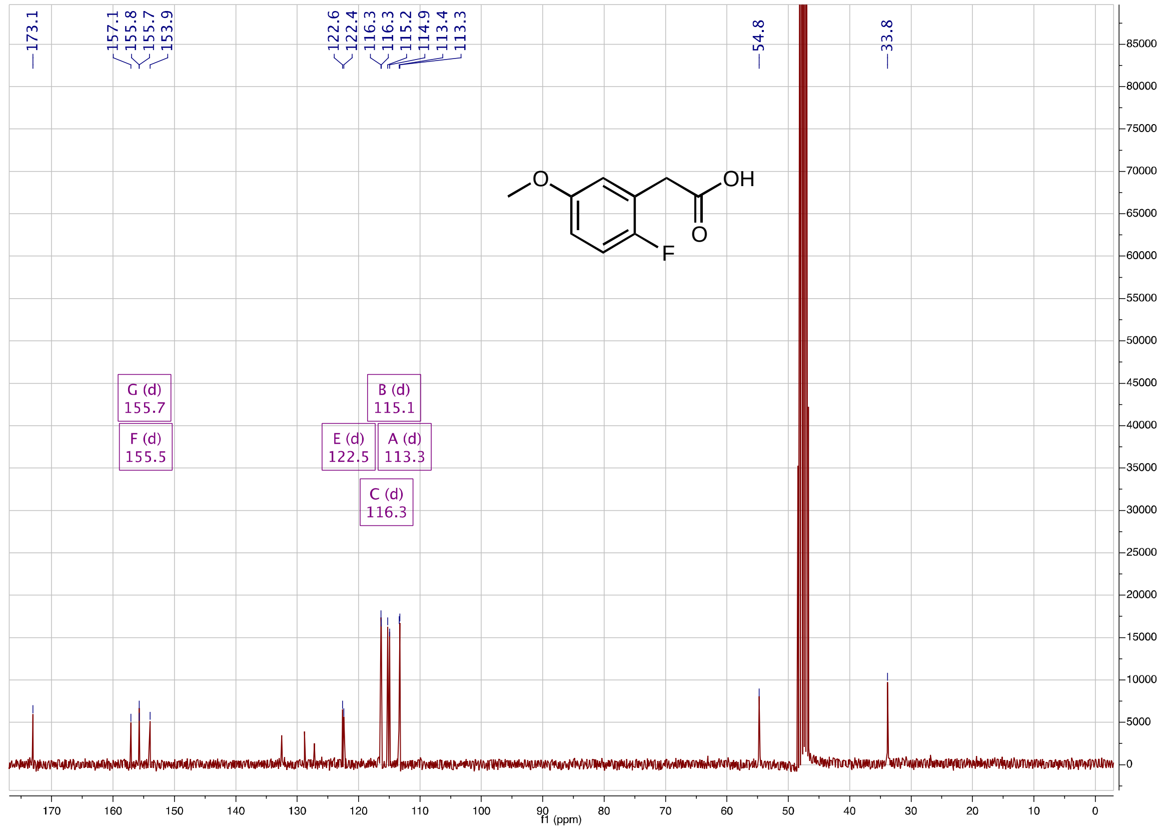

2-(2-Fluoro-5-hydroxyphenyl)acetic acid

^{1}H-NMR

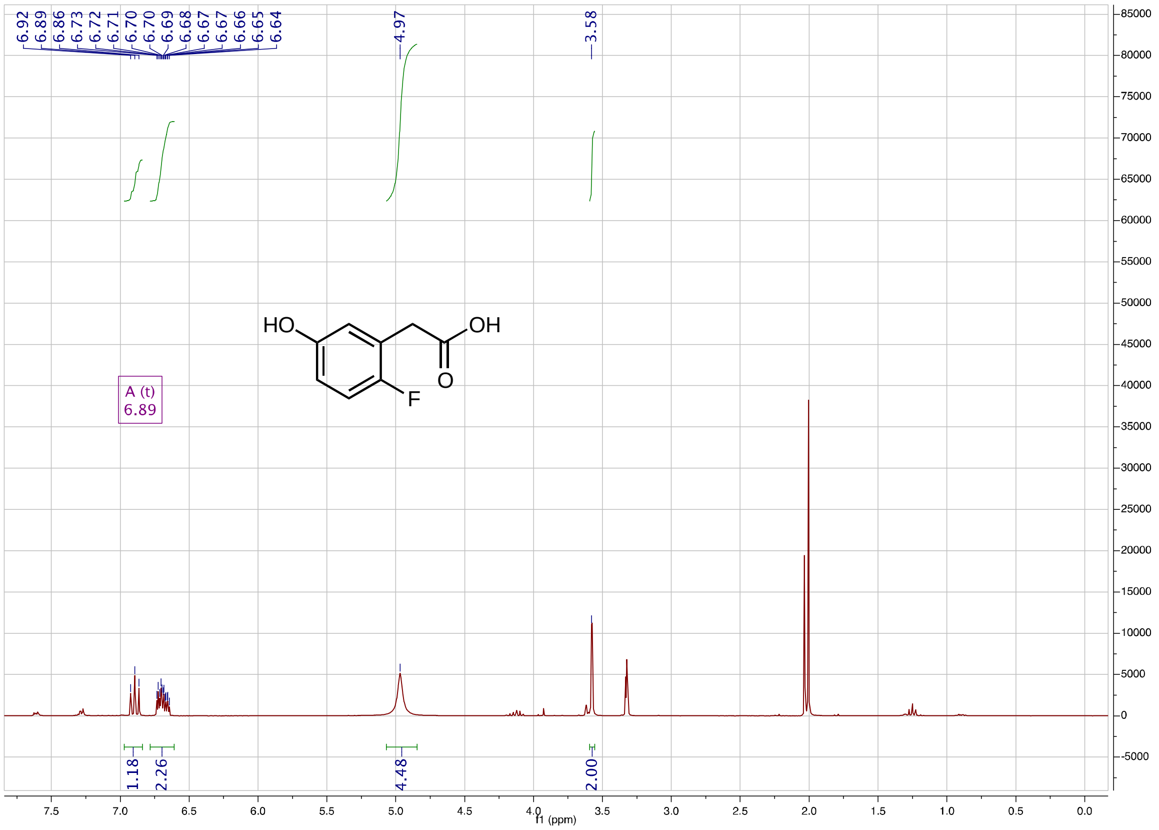

^{13}C-NMR

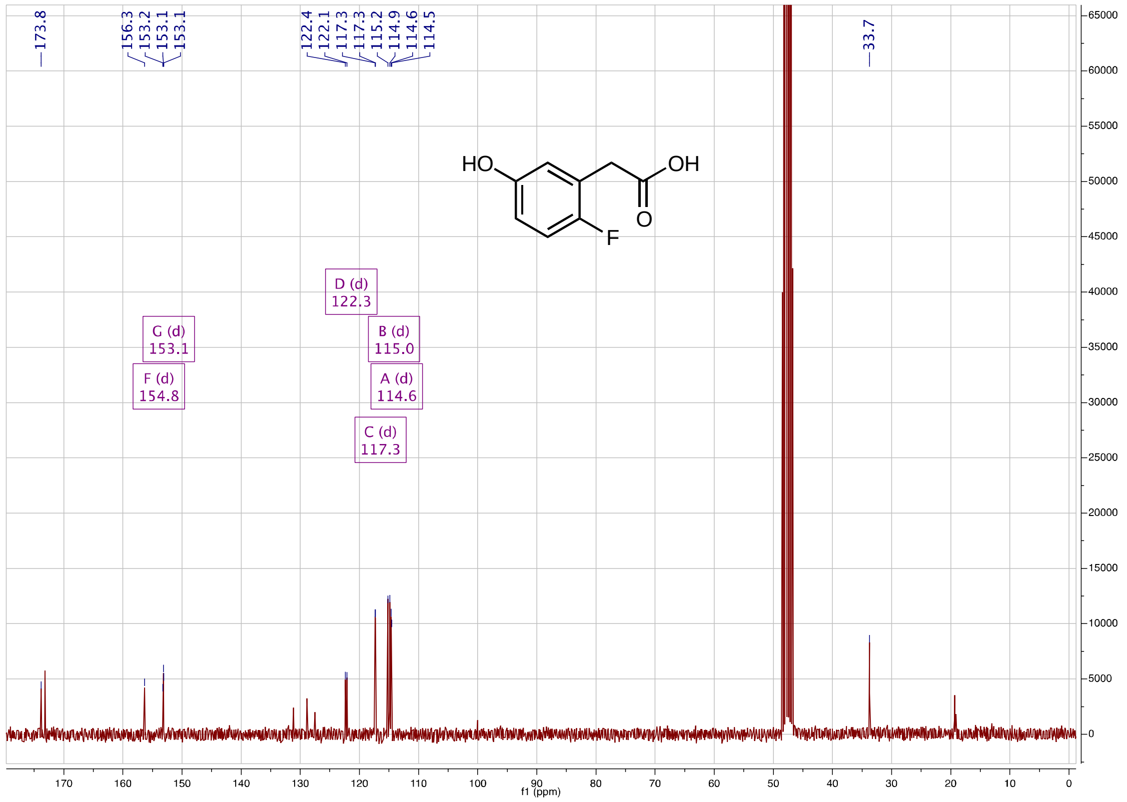

2-(5-(Benzyloxy)-2-fluorophenyl)acetic acid

^{1}H-NMR

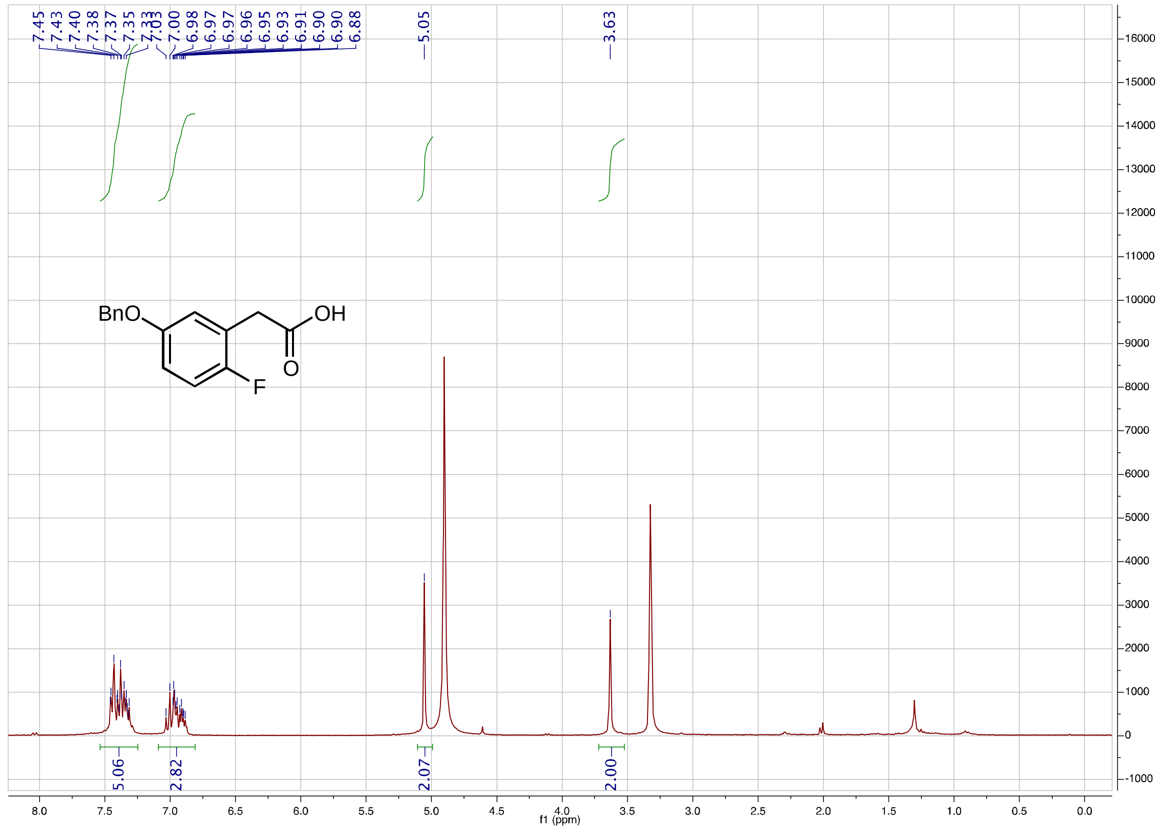

^{13}C-NMR

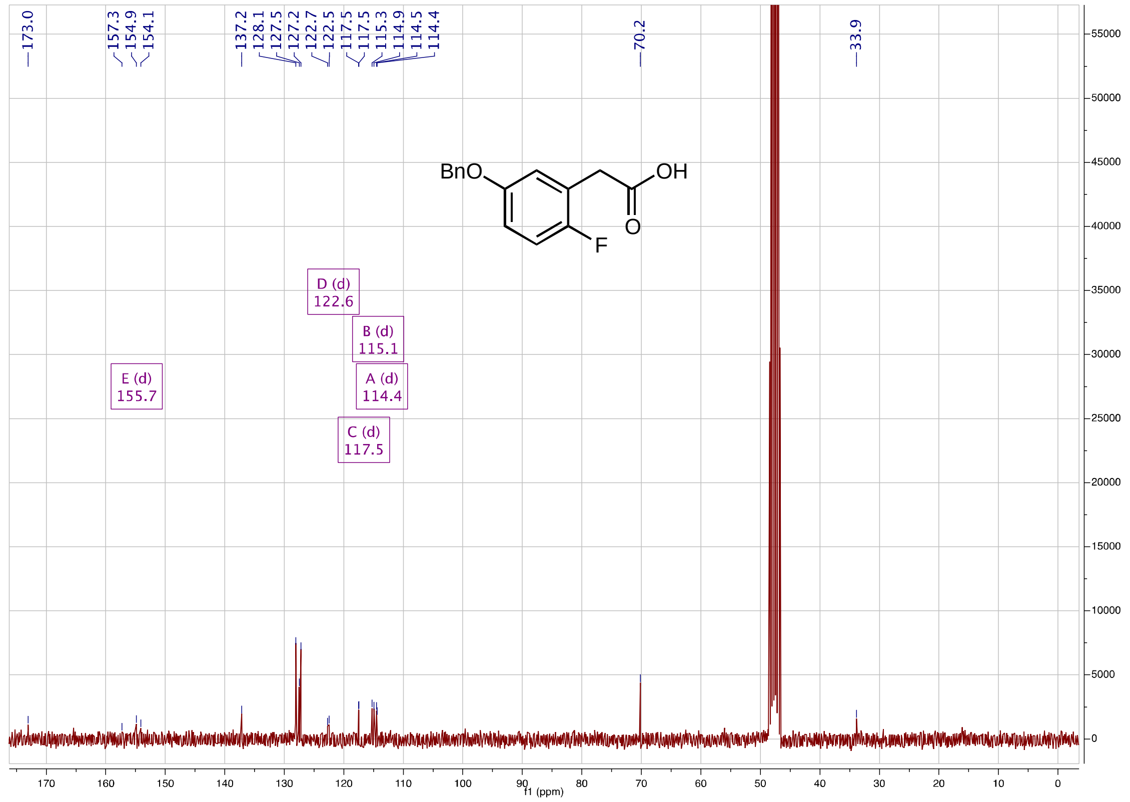

2-(5-(Benzyloxy)-2-fluorophenyl)-*N*-(3,4-dimethoxyphenethyl)-*N*-methylacetamide

^{1}H-NMR

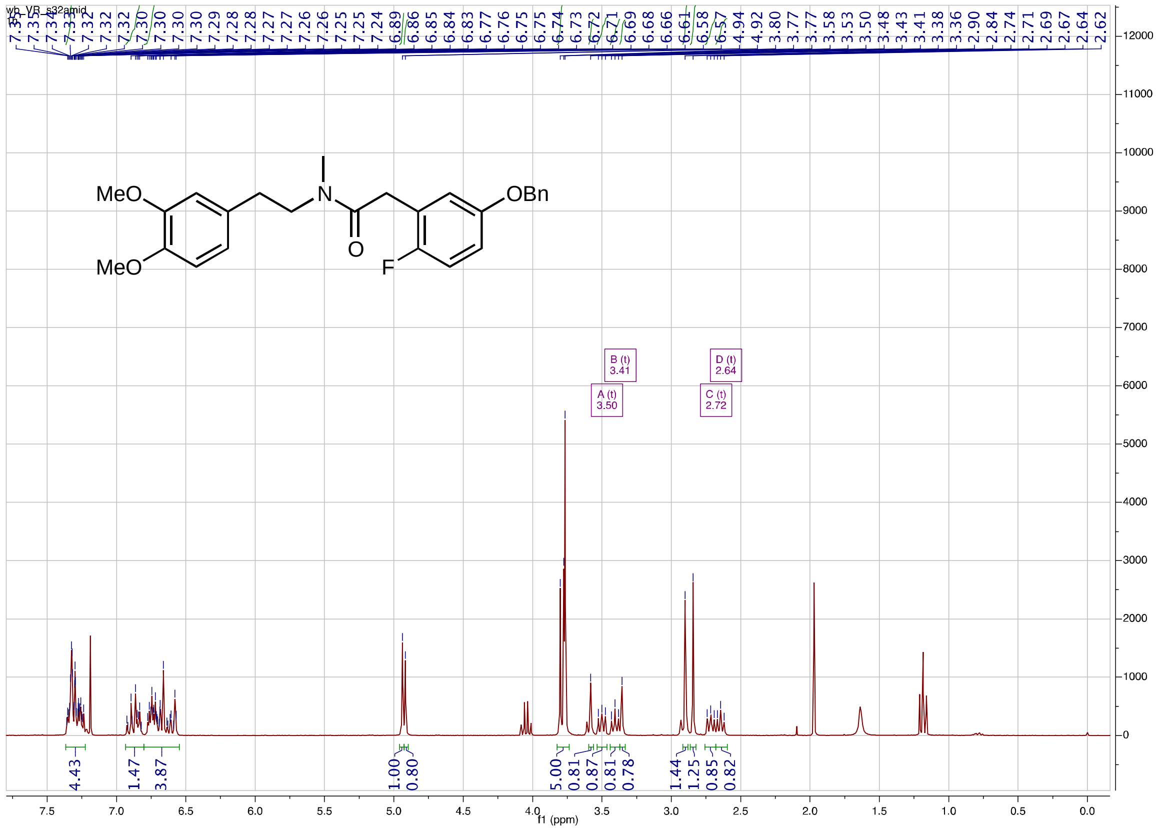

^{13}C-NMR

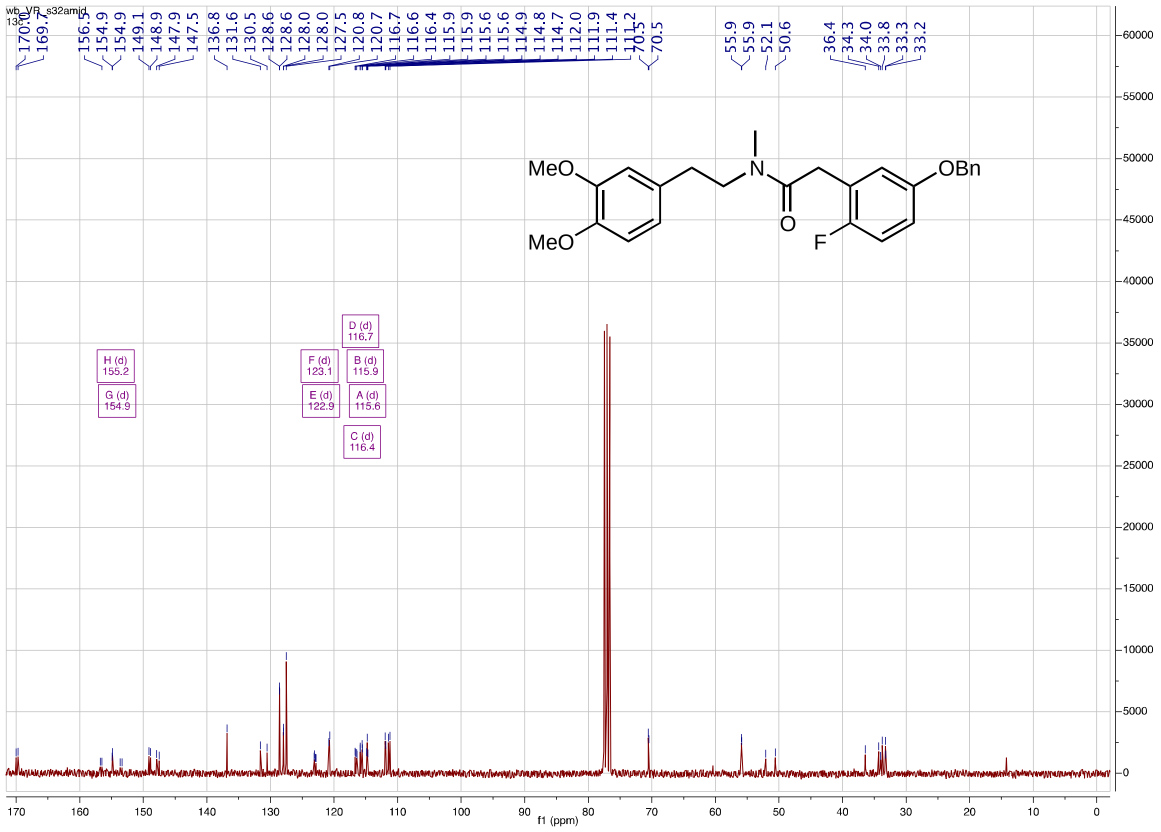

^{13}C-NMR DEPT90 spectrum

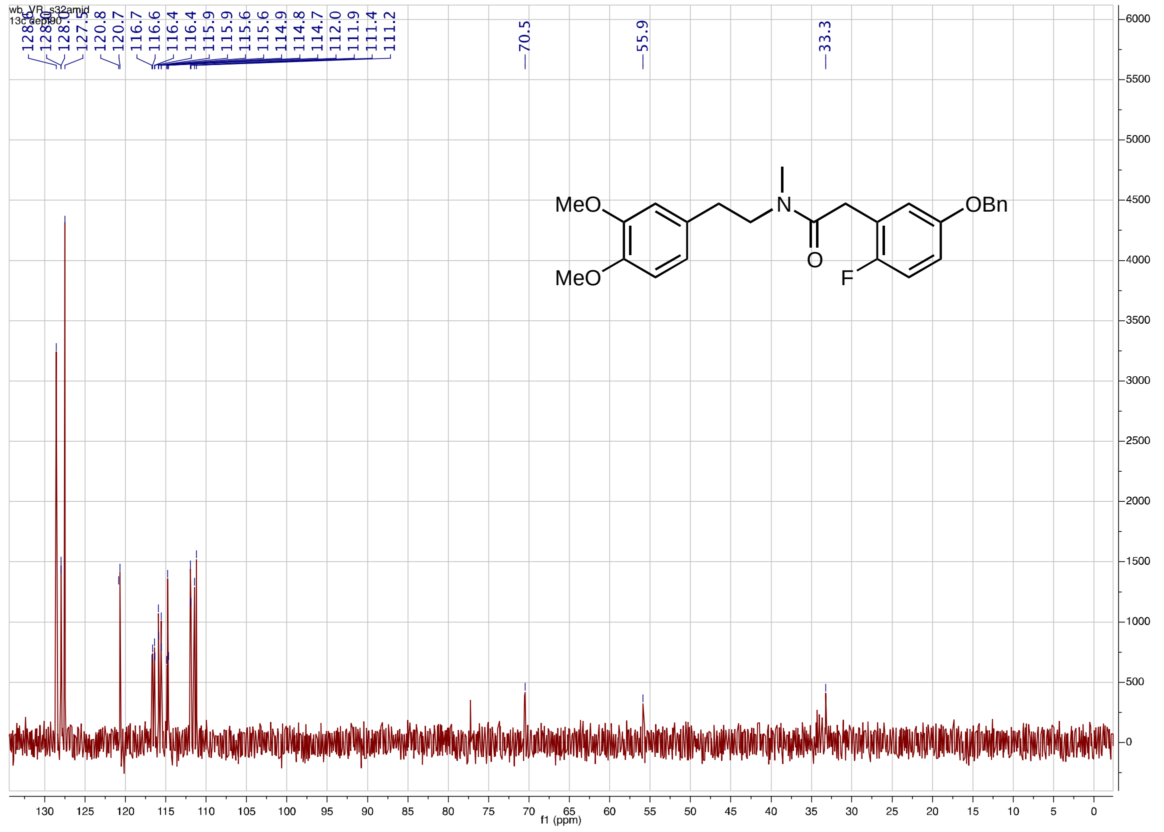

^{13}C-NMR DEPT135 spectrum

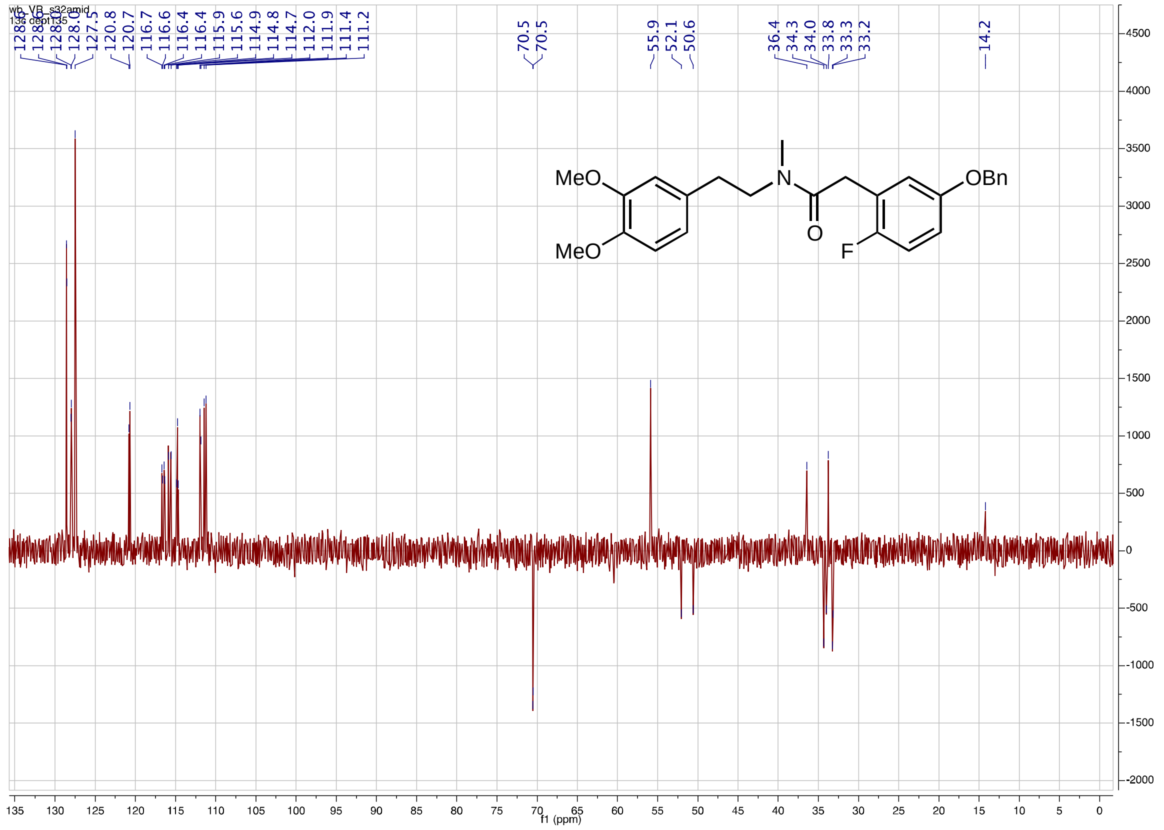

COSY spectrum

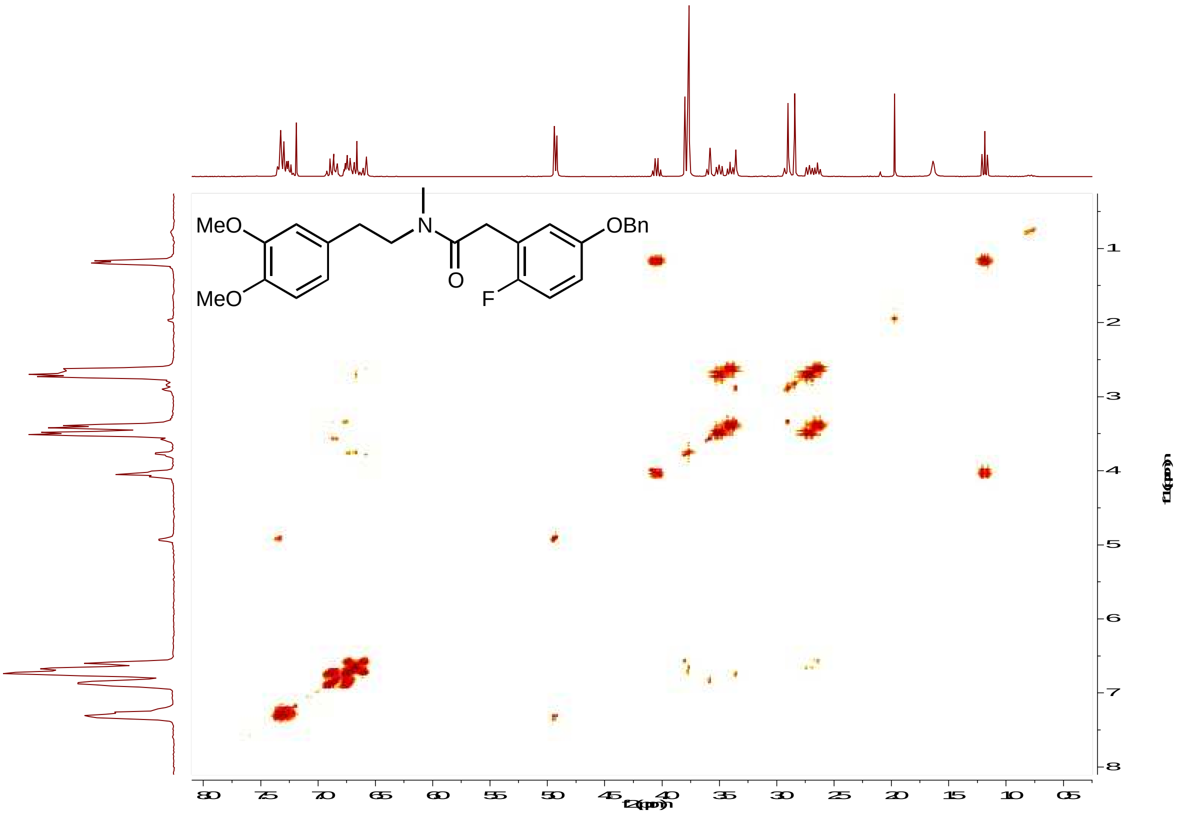

HSQC spectrum

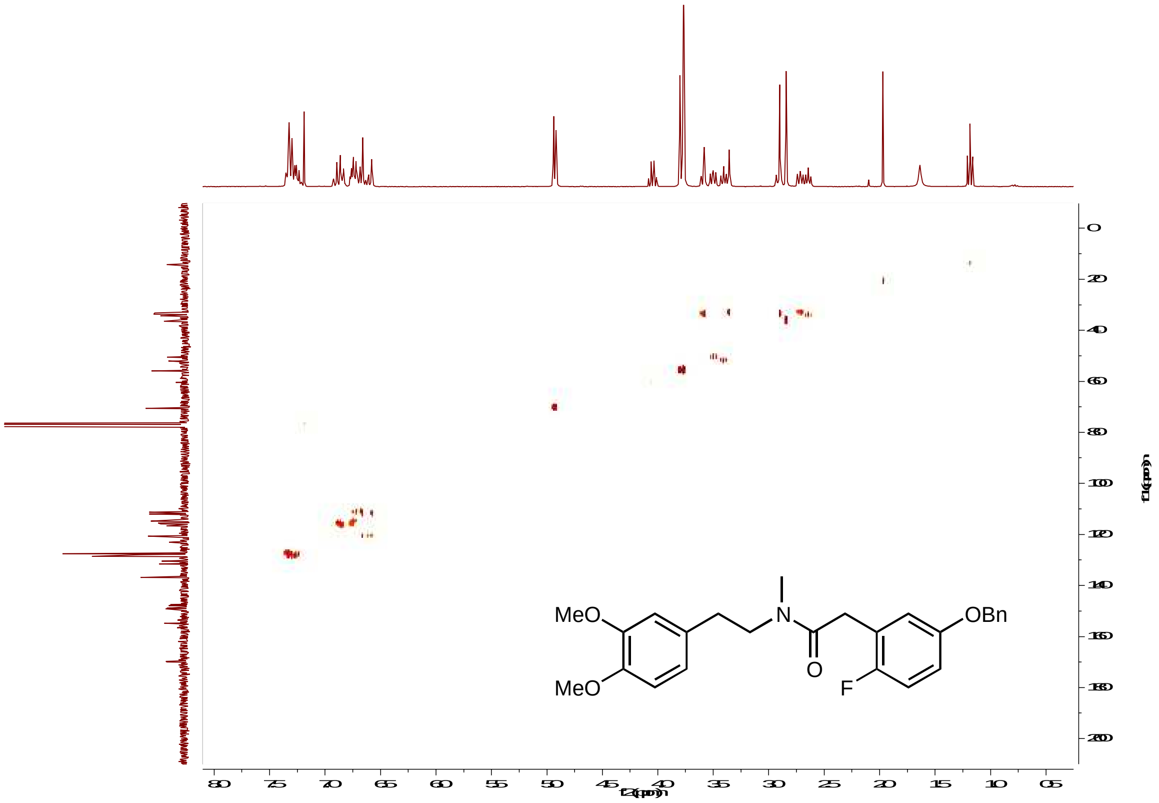

HRMS results

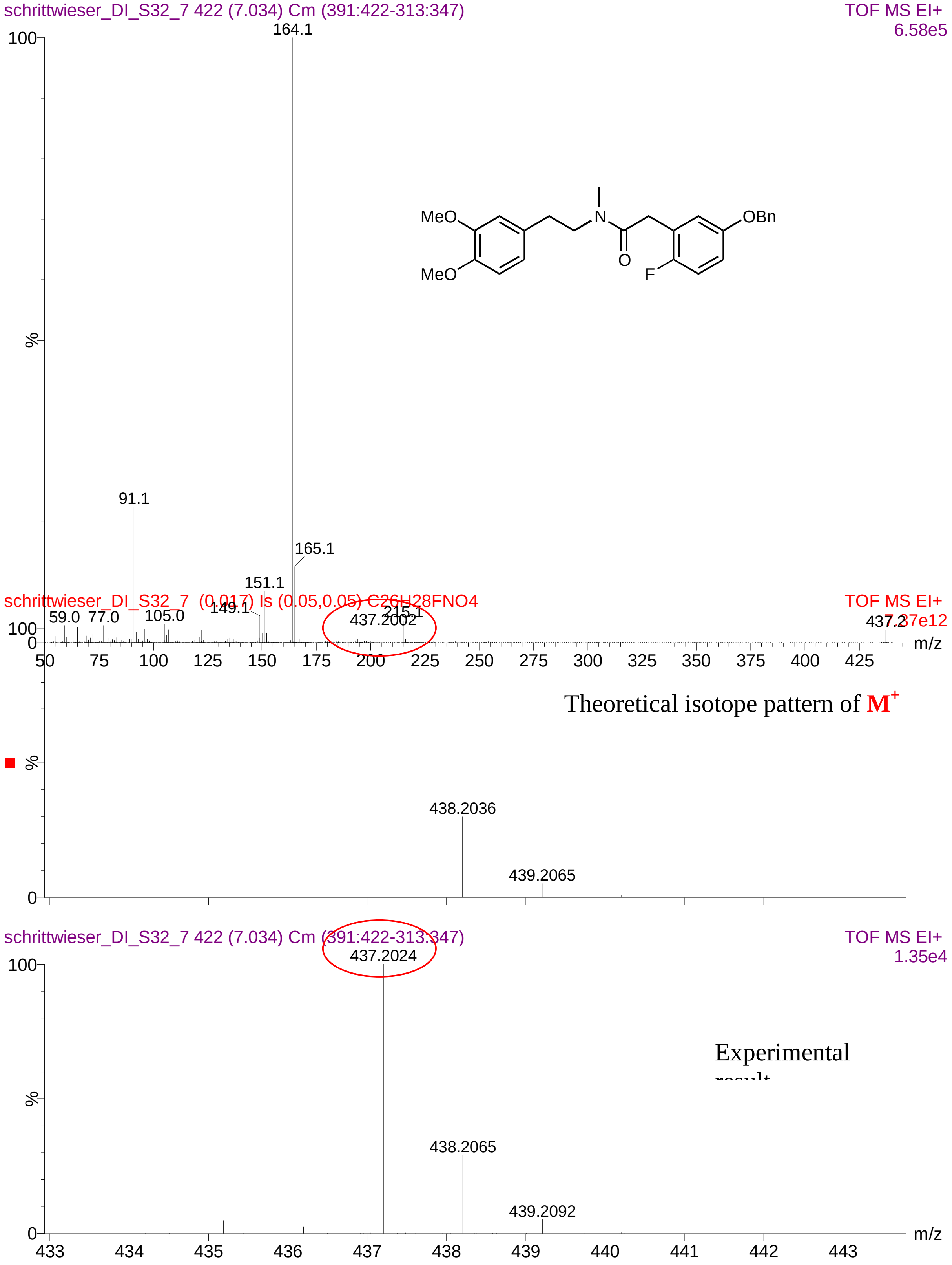

1-(5-(Benzyloxy)-2-fluorobenzyl)-6,7-dimethoxy-2-methyl-1,2,3,4-tetrahydroisoquinoline

^{1}H-NMR

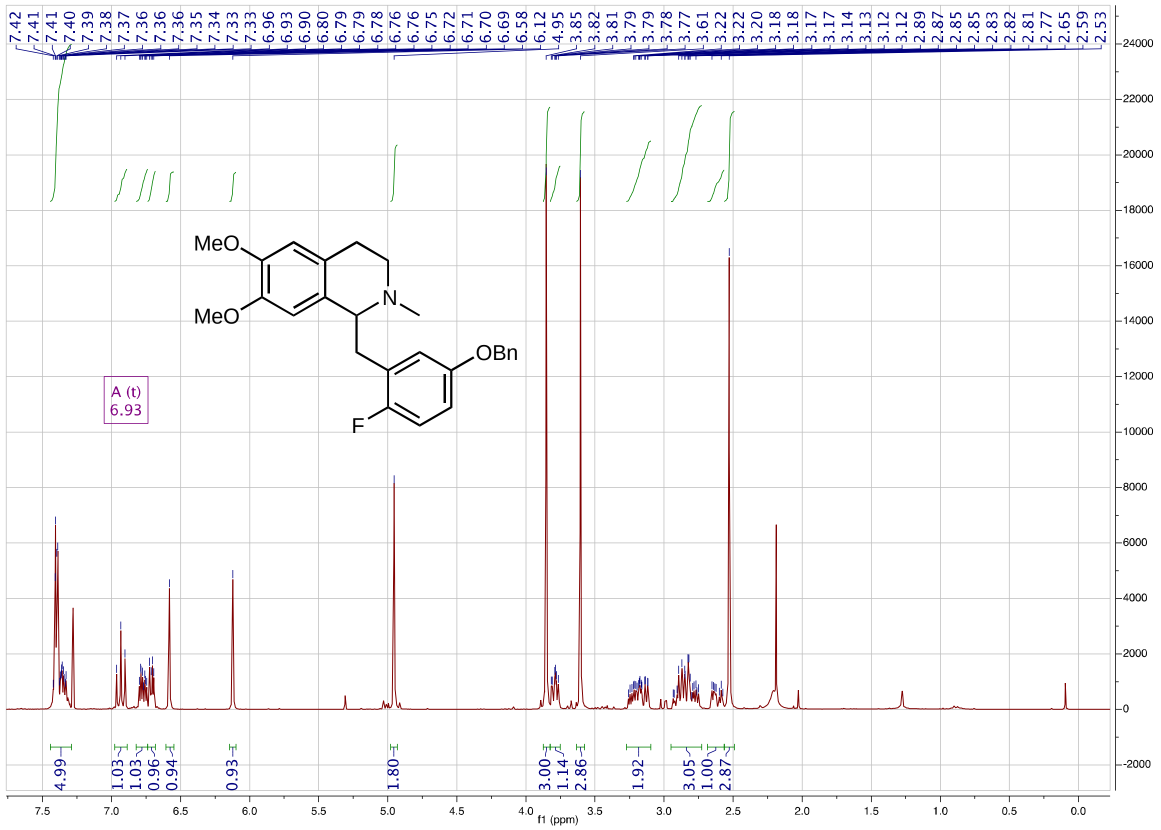

^{13}C-NMR

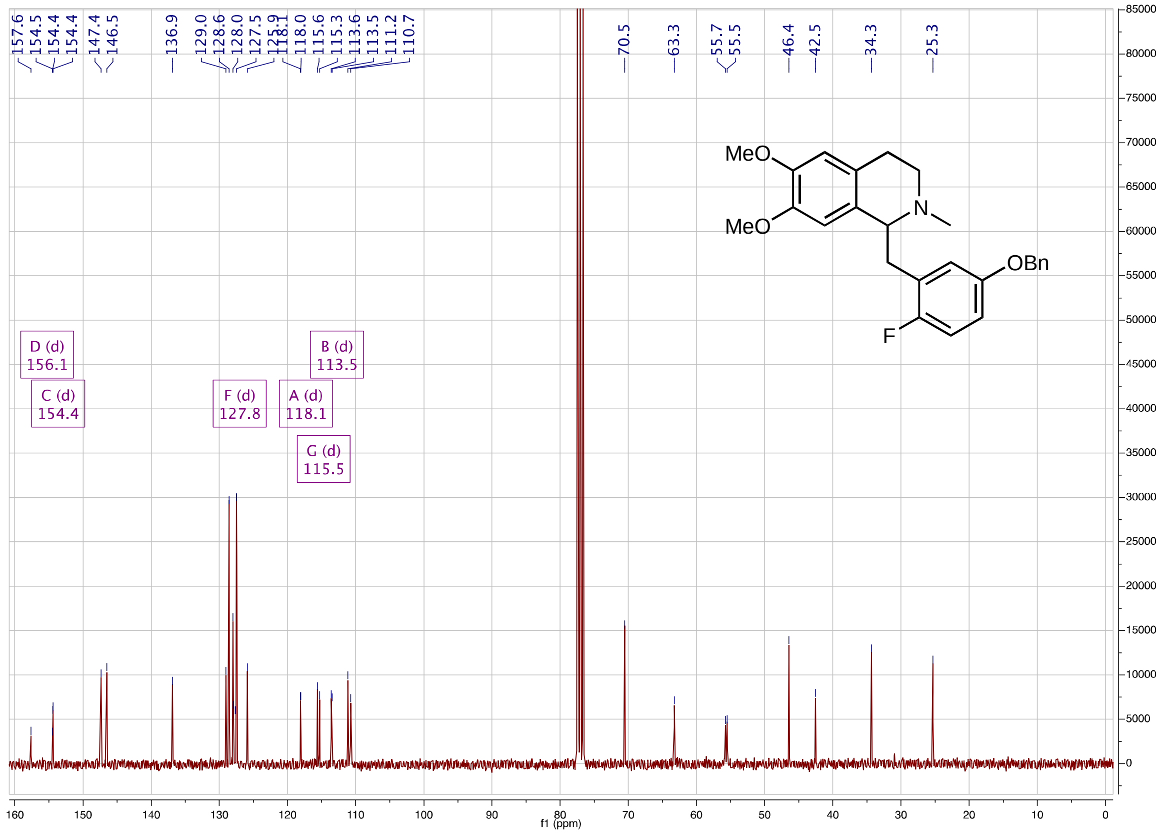

HRMS results

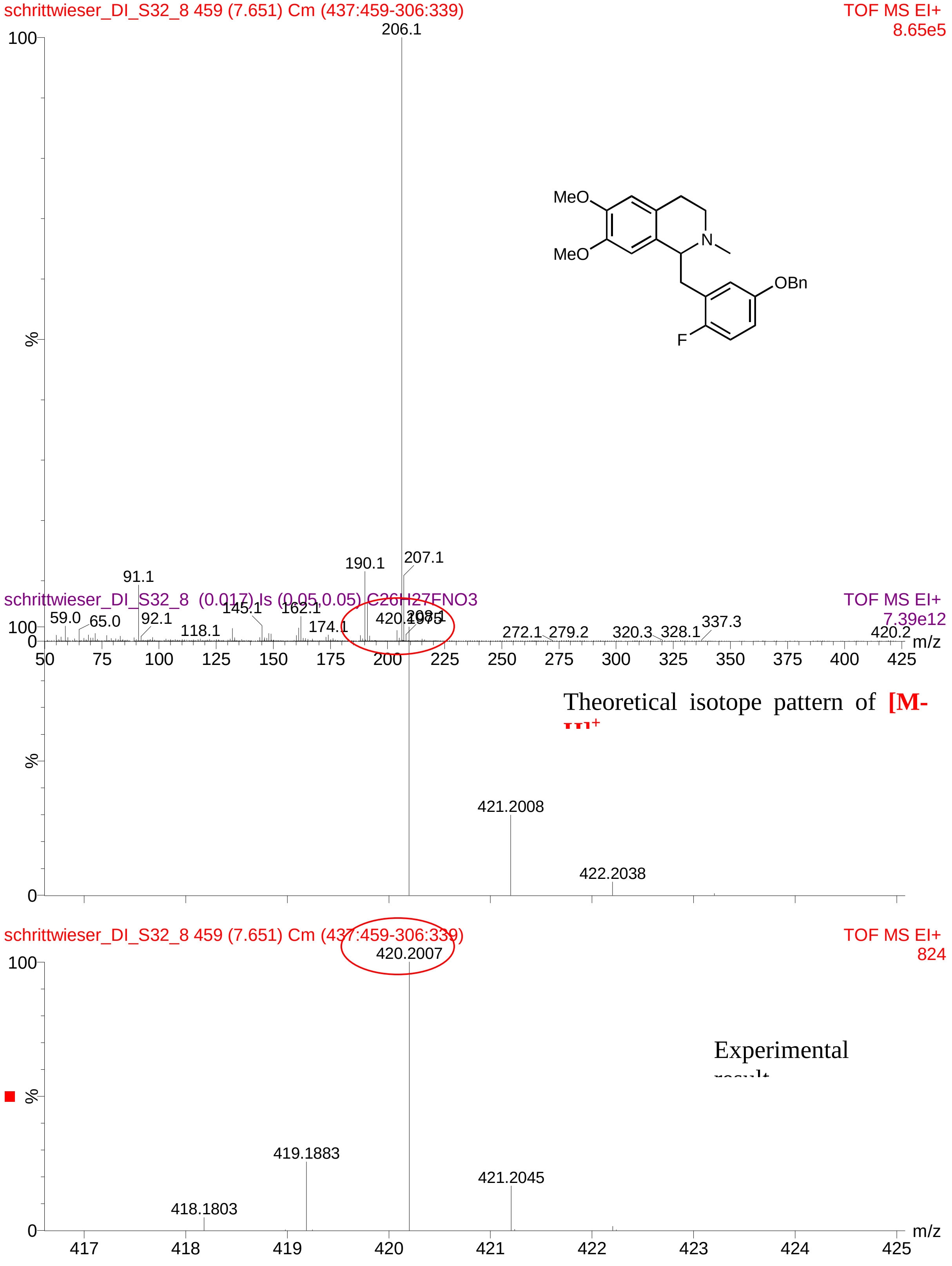

3-((6,7-Dimethoxy-2-methyl-1,2,3,4-tetrahydroisoquinolin-1-yl)methyl)-4-fluorophenol

^{1}H-NMR

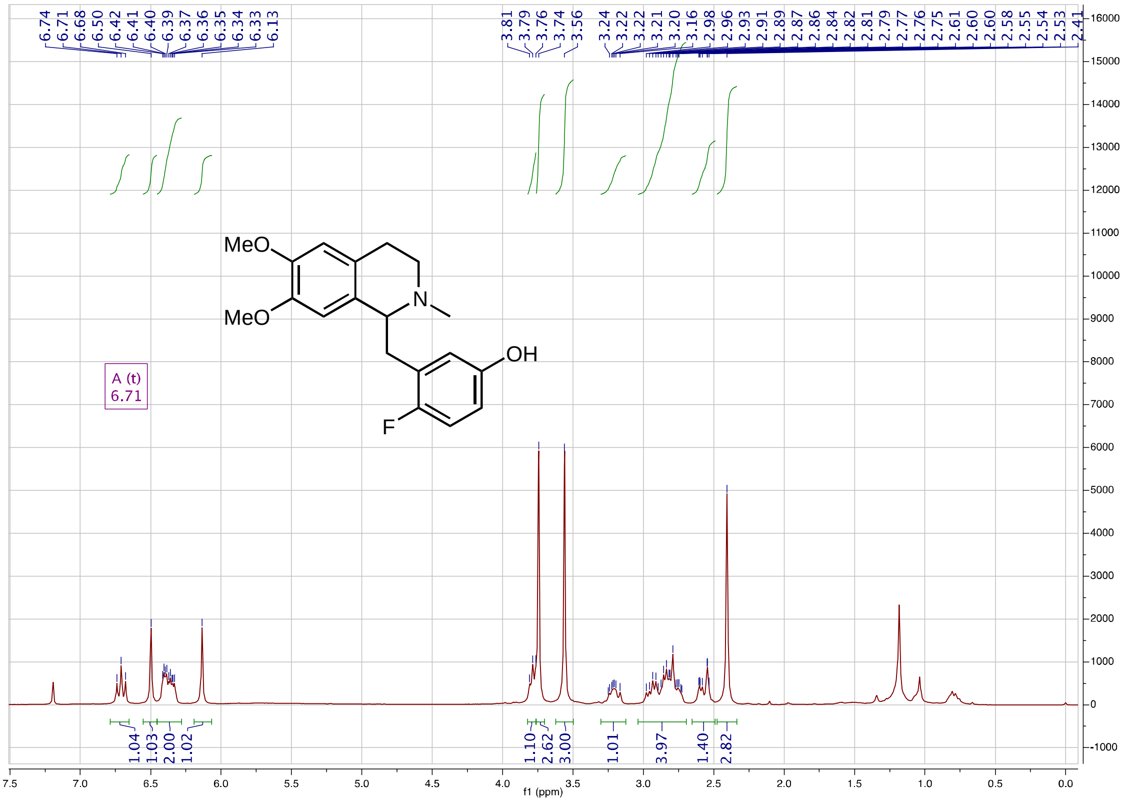

^{13}C-NMR

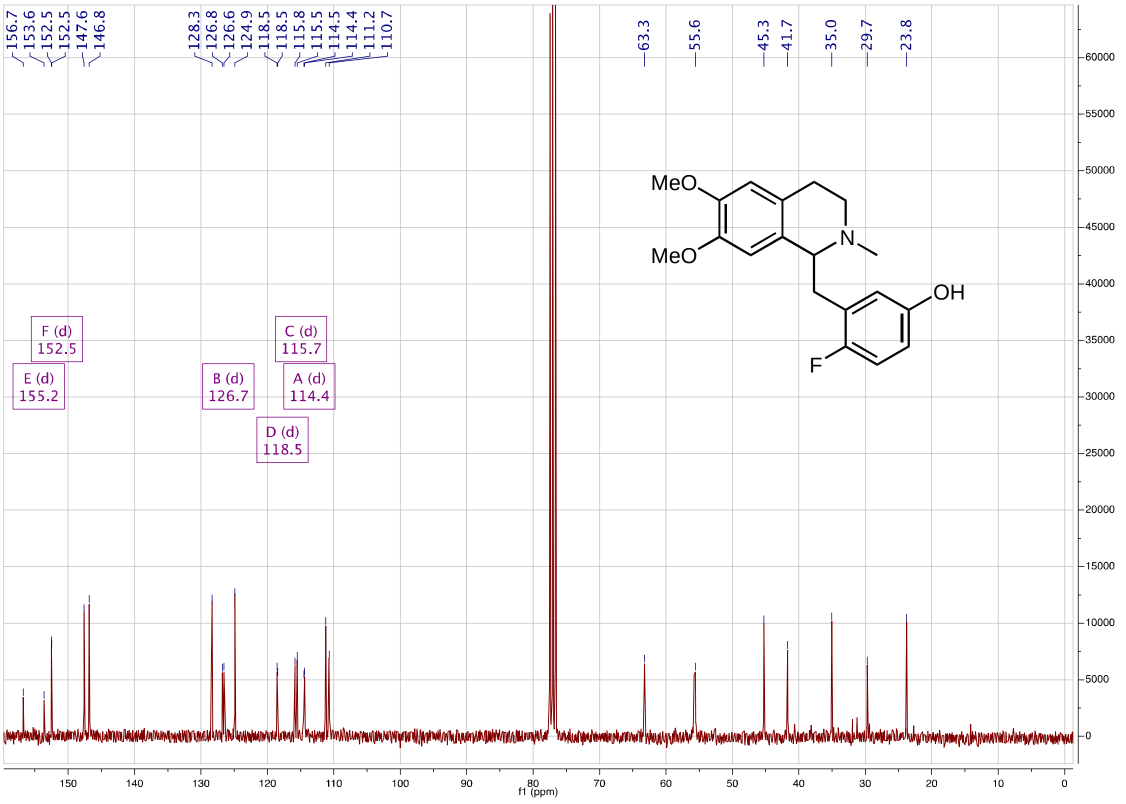

COSY spectrum

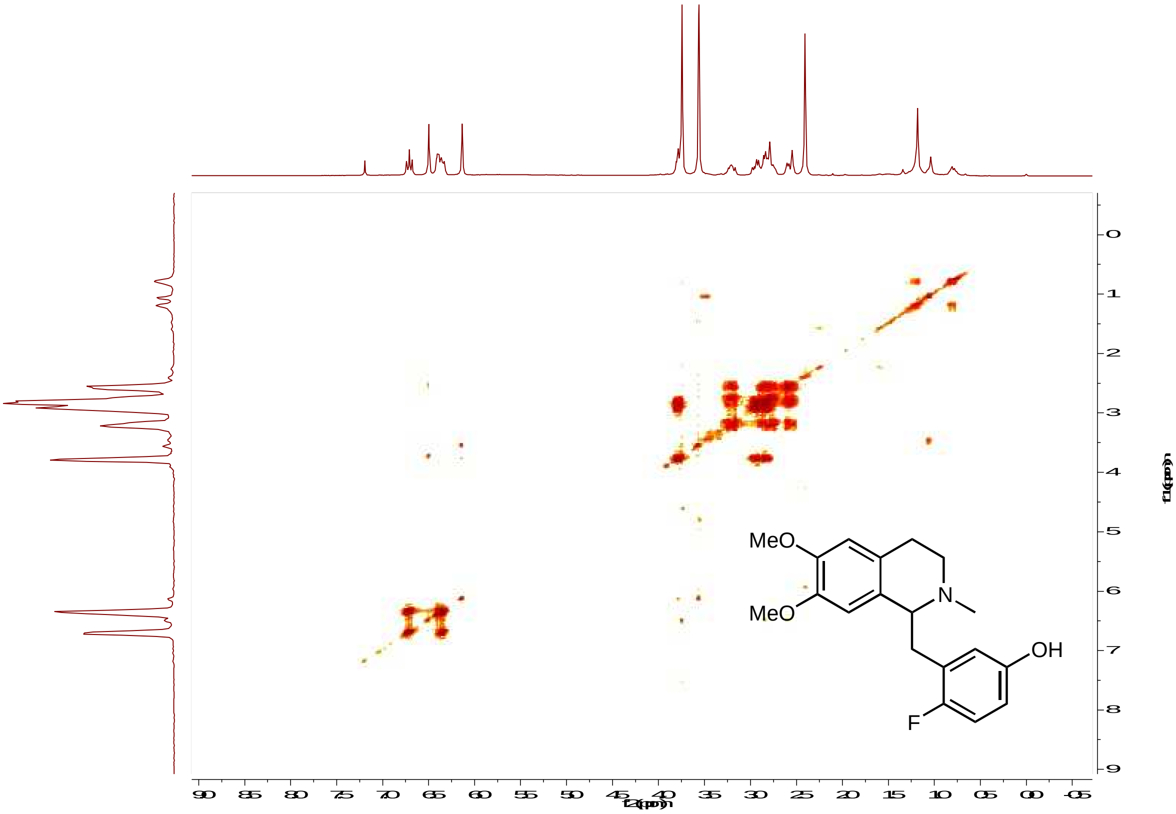

HSQC spectrum

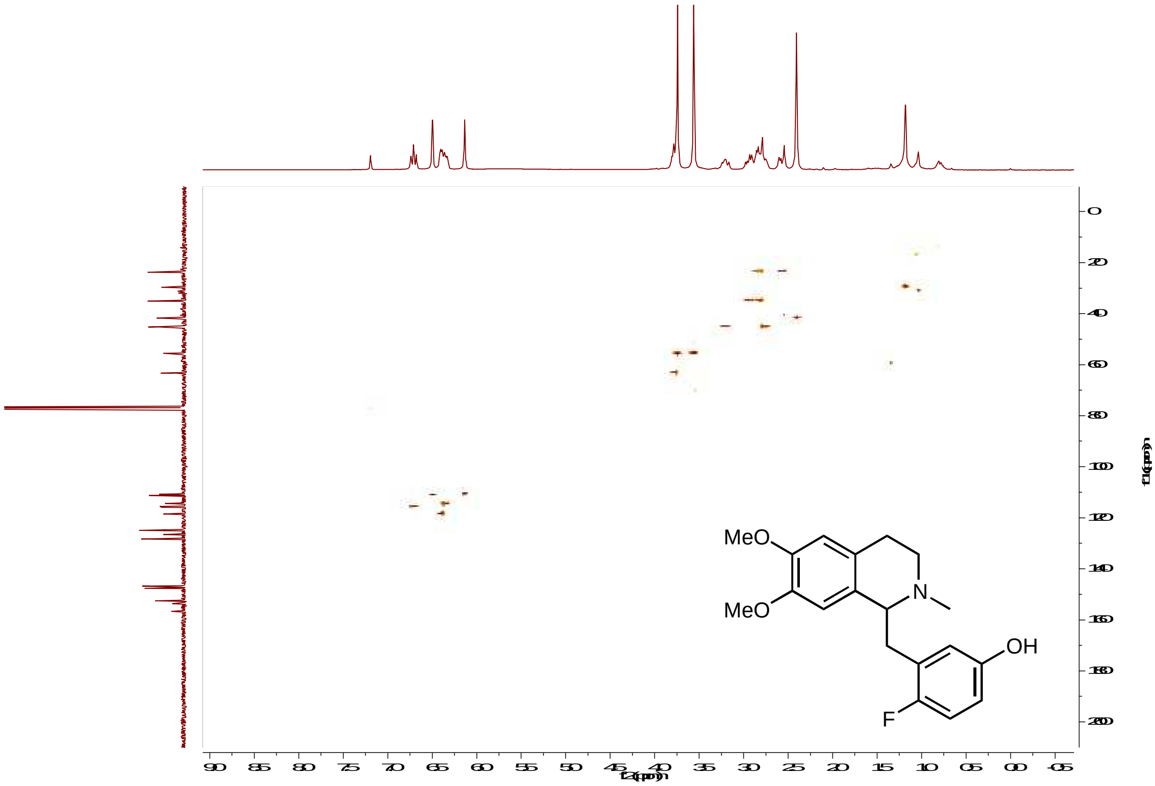

HRMS results

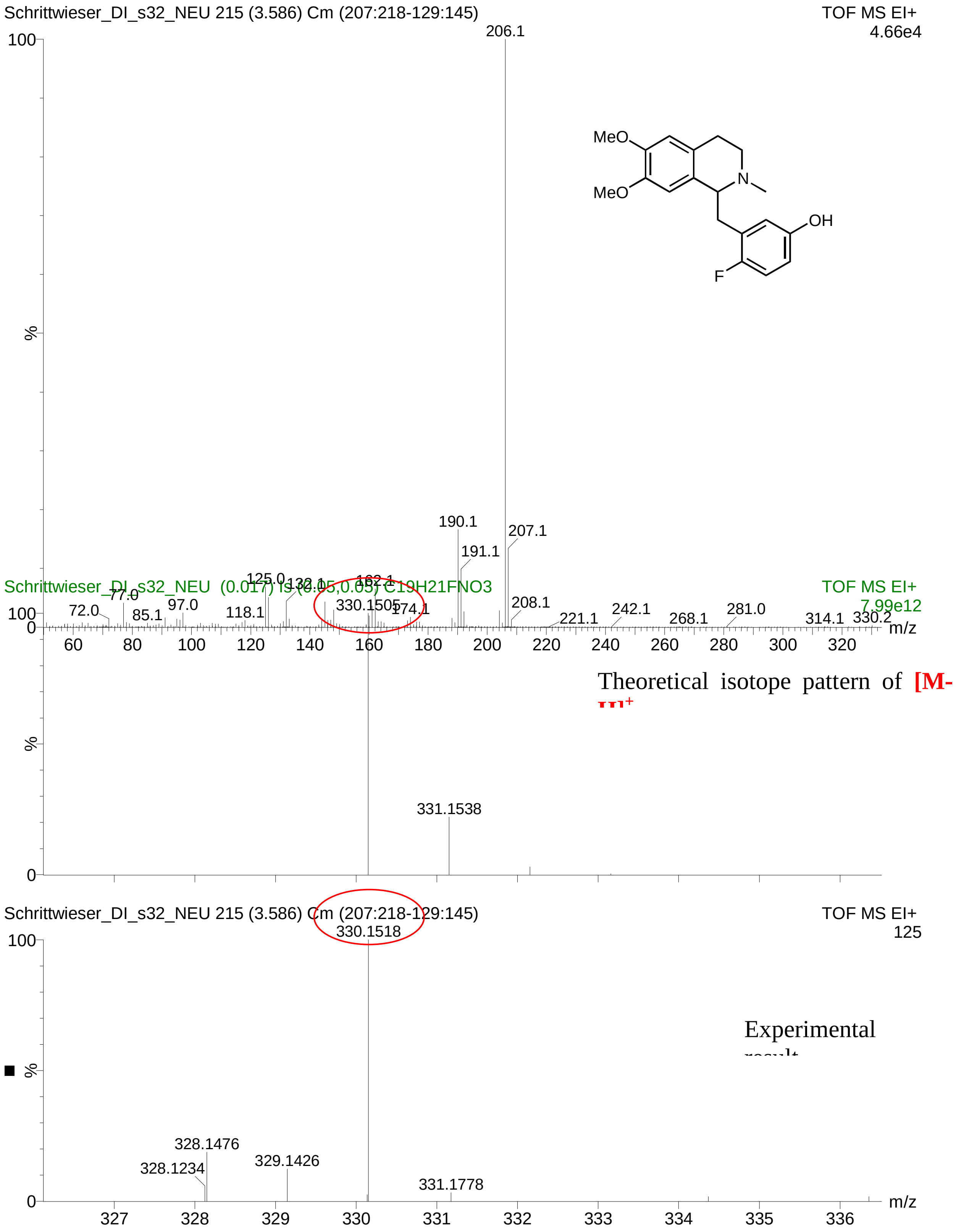

Synthesis of **1l**:

Provided Material:

3-(Benzyloxy)-2-chlorobenzaldehyde
^{1}H-NMR spectrum, ^{13}C-NMR spectrum

1-(3-(Benzyloxy)-2-chlorophenyl)-2,2,2-trichloroethanol
^{1}H-NMR spectrum, ^{13}C-NMR spectrum, HRMS results

2-(3-(Benzyloxy)-2-chlorophenyl)acetic acid
^{1}H-NMR spectrum, ^{13}C-NMR spectrum, HRMS results

2-(3-(Benzyloxy)-2-chlorophenyl)-*N*-(3,4-dimethoxyphenethyl)-*N*-methylacetamide
^{1}H-NMR spectrum, ^{13}C-NMR spectrum, ^{13}C-NMR DEPT135 spectrum, ^{13}C-NMR DEPT90 spectrum, COSY spectrum, HSQC spectrum, HRMS results

1-(3-(Benzyloxy)-2-chlorobenzyl)-6,7-dimethoxy-2-methyl-1,2,3,4-tetrahydroisoquinoline
^{1}H-NMR spectrum, ^{13}C-NMR spectrum, HRMS results

2-Chloro-3-((6,7-dimethoxy-2-methyl-1,2,3,4-tetrahydroisoquinolin-1-yl)methyl)phenol
^{1}H-NMR spectrum, ^{13}C-NMR spectrum, COSY spectrum, HSQC spectrum, HRMS results

3-(Benzyloxy)-2-chlorobenzaldehyde

^{1}H-NMR

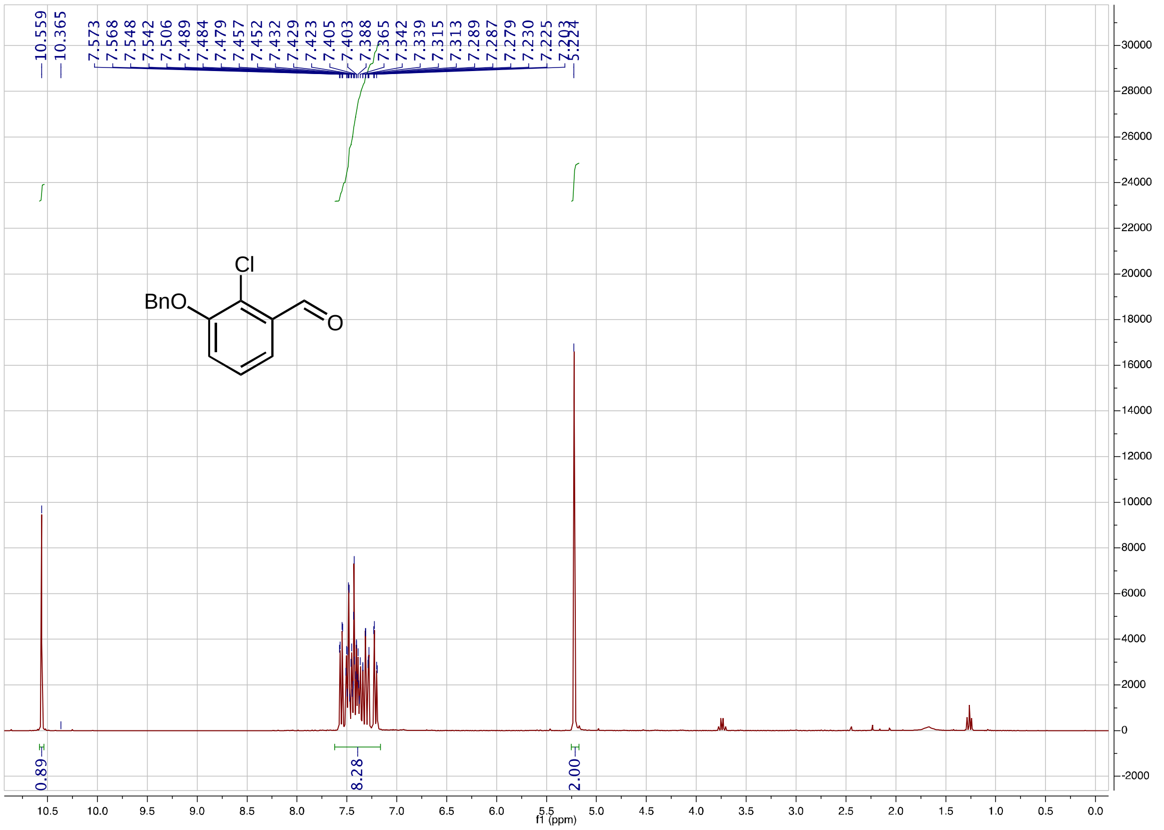

^{13}C-NMR

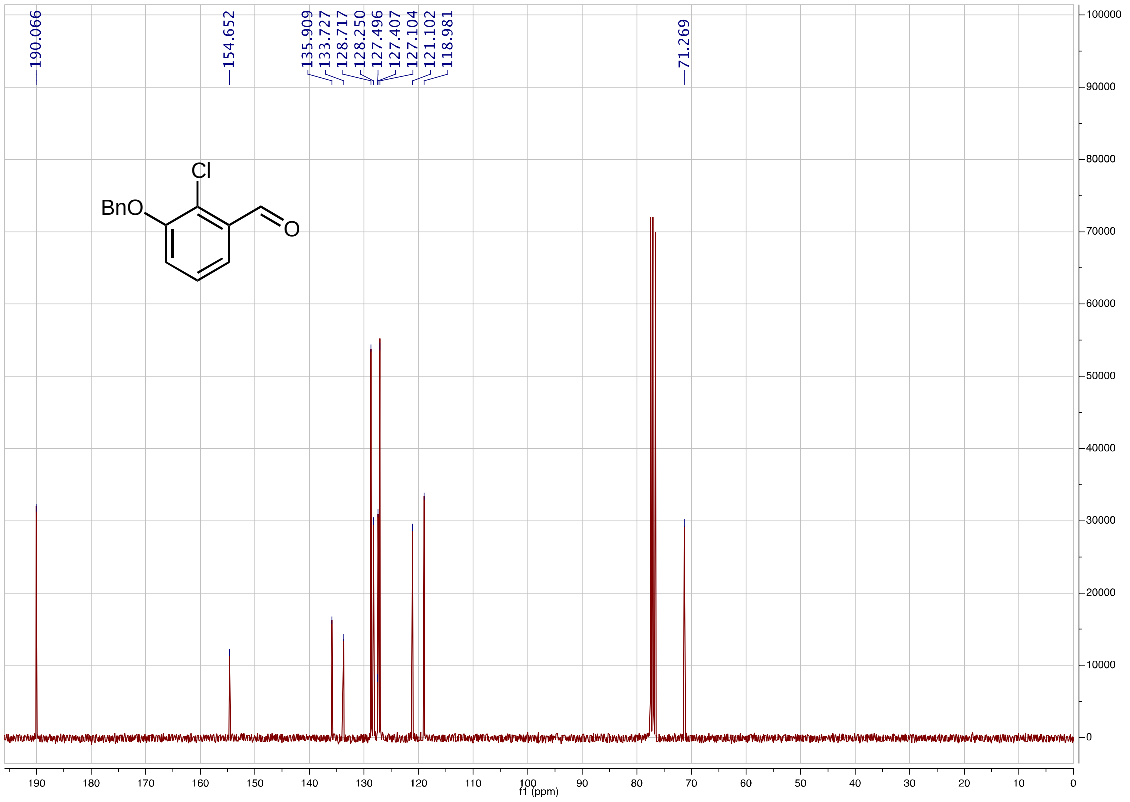

1-(3-(Benzyloxy)-2-chlorophenyl)-2,2,2-trichloroethanol

^{1}H-NMR

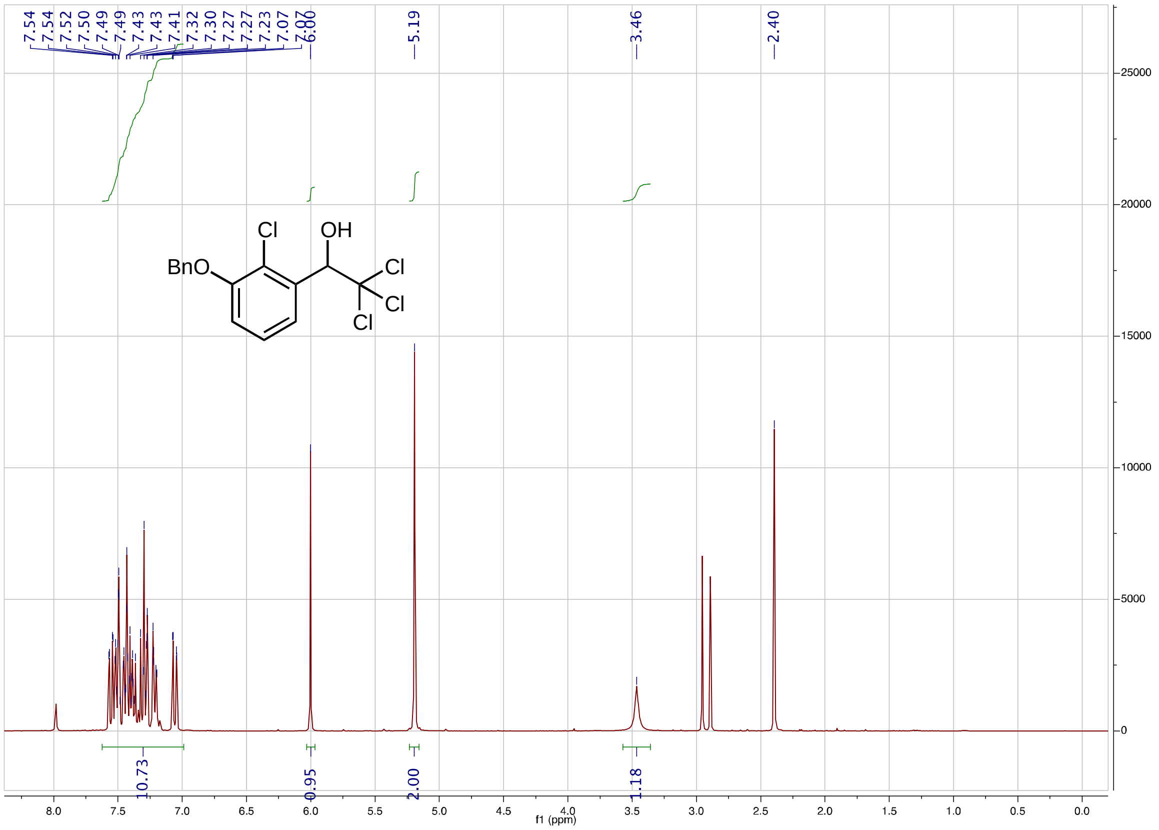

^{13}C-NMR

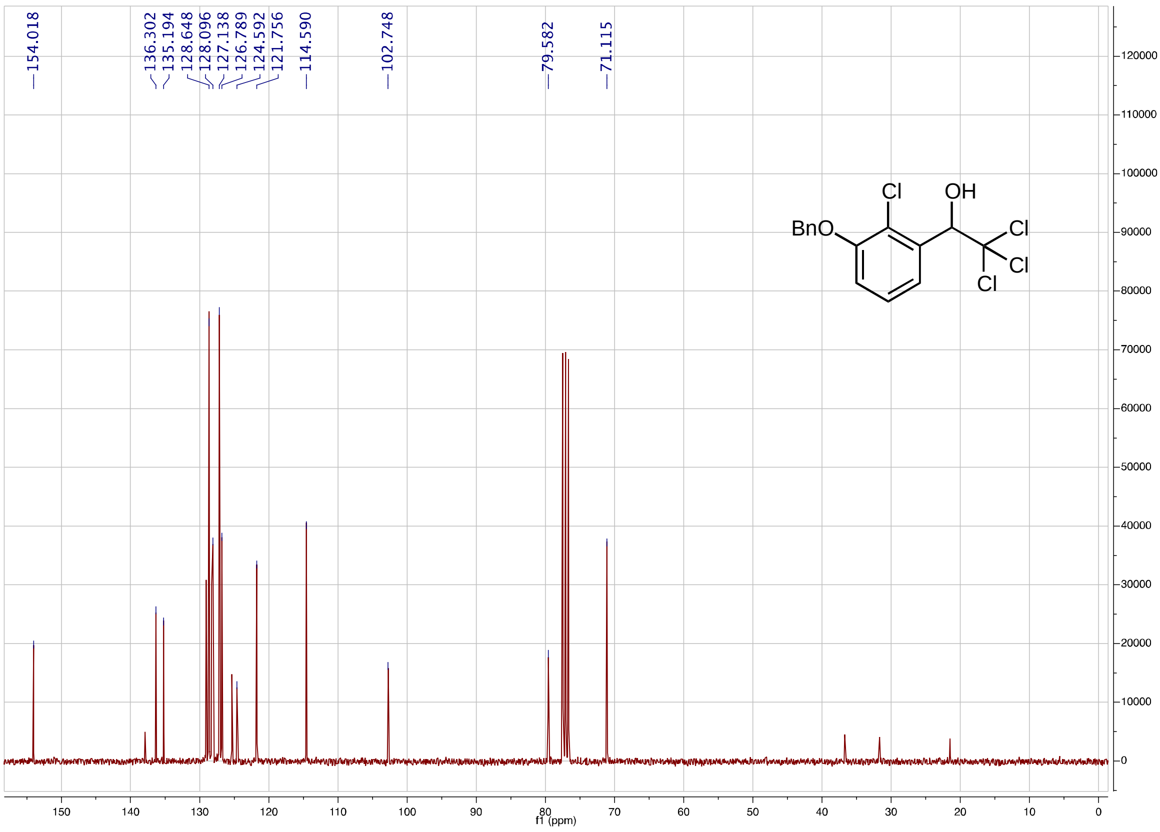

HRMS results

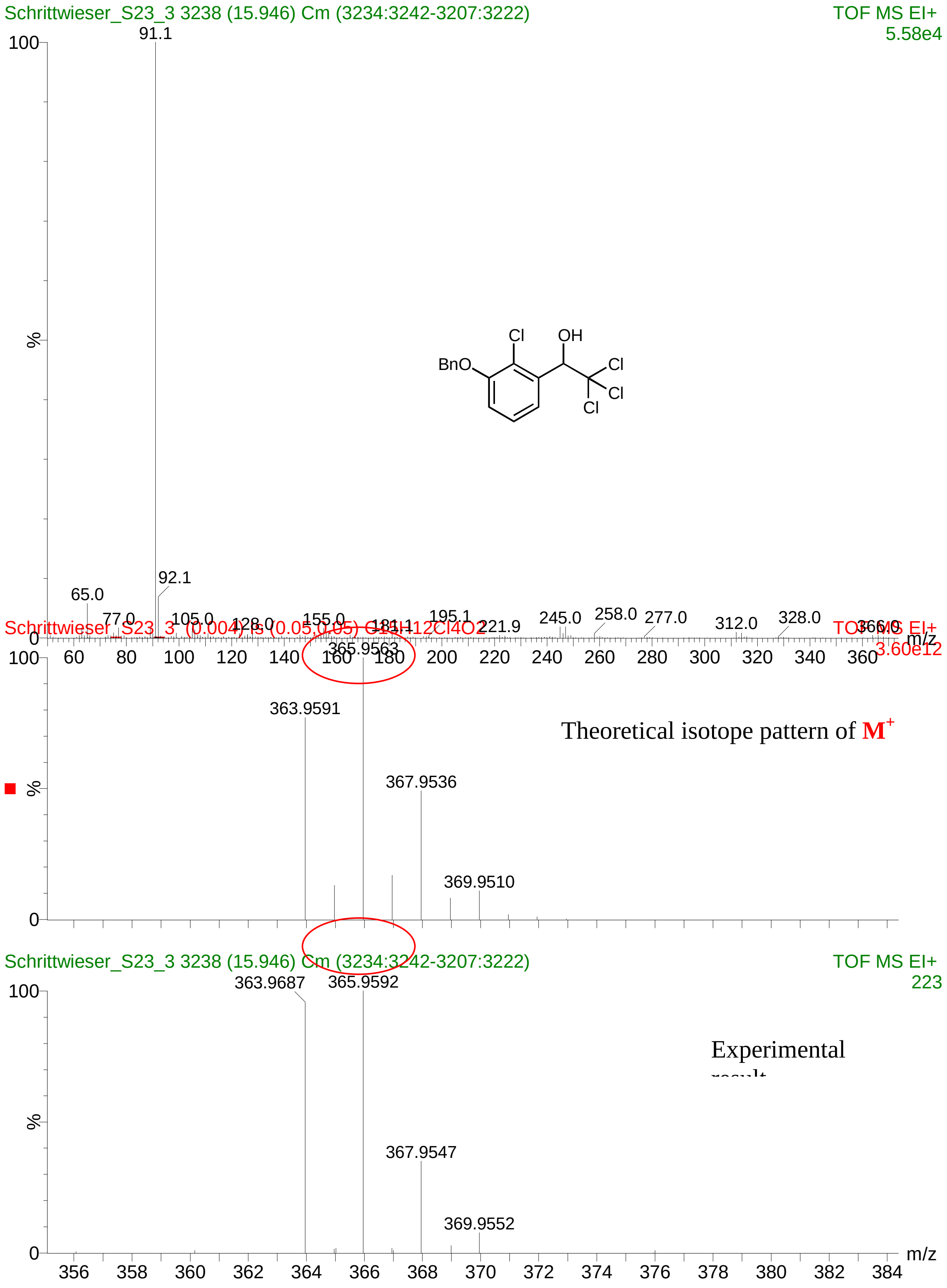

2-(3-(Benzyloxy)-2-chlorophenyl)acetic acid

^{1}H-NMR

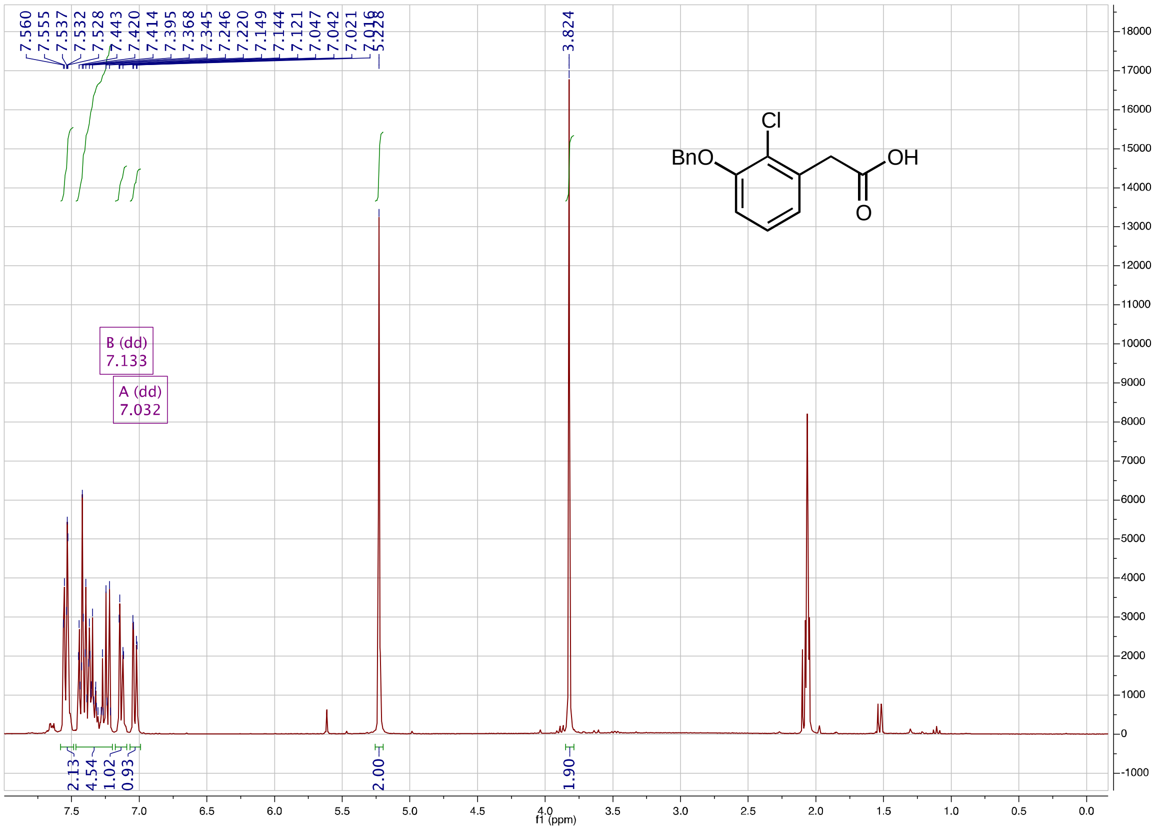

^{13}C-NMR

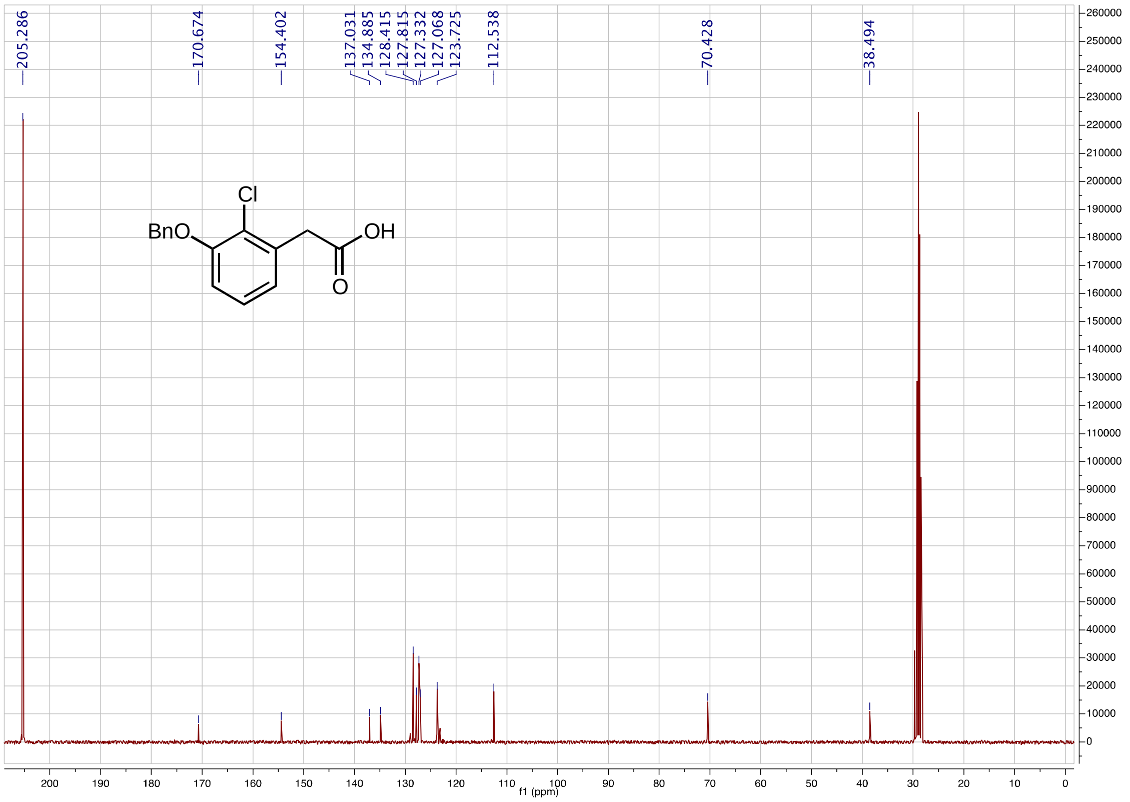

HRMS results

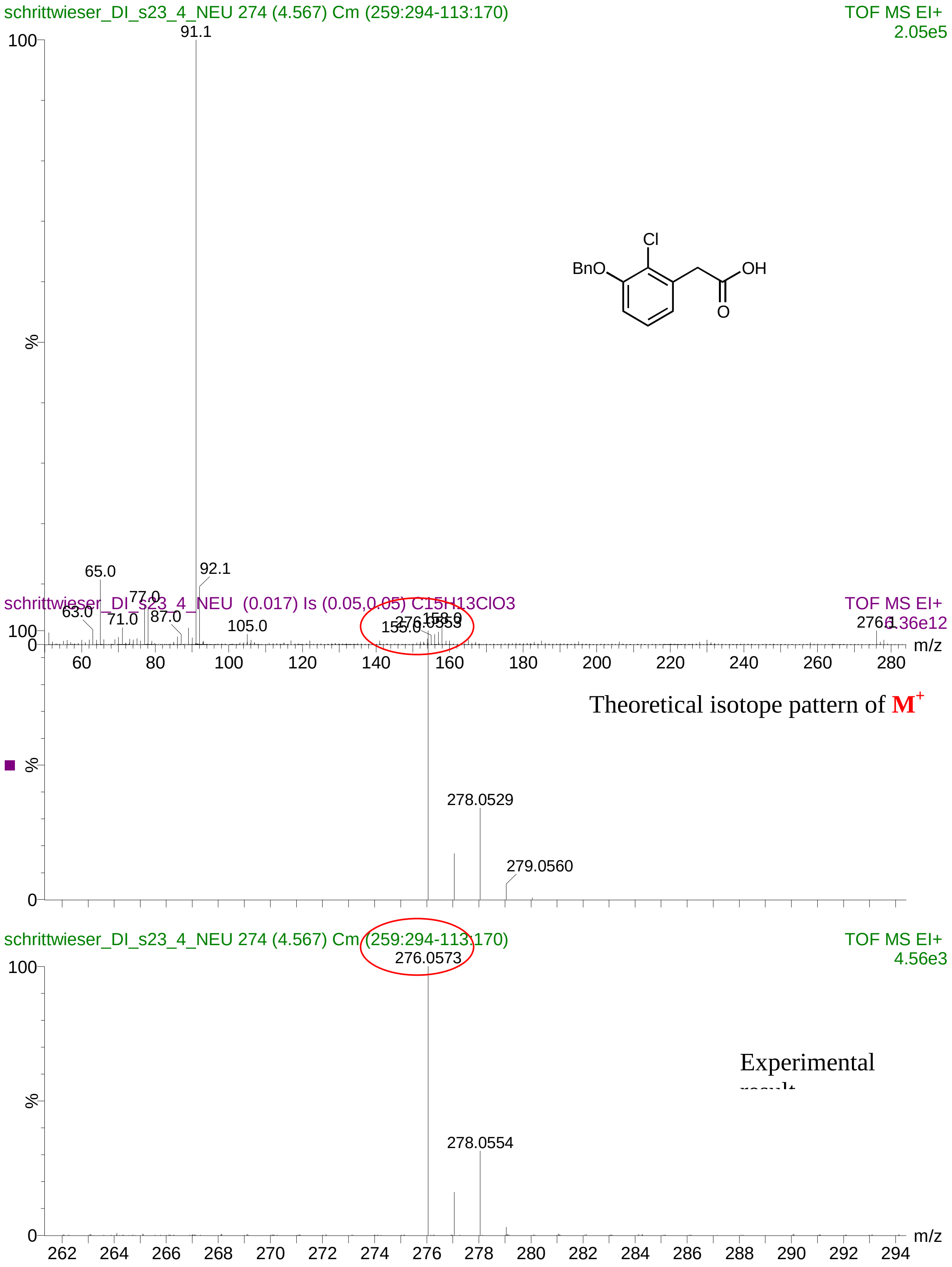

2-(3-(Benzyloxy)-2-chlorophenyl)-*N*-(3,4-dimethoxyphenethyl)-*N*-methylacetamide

^{1}H-NMR

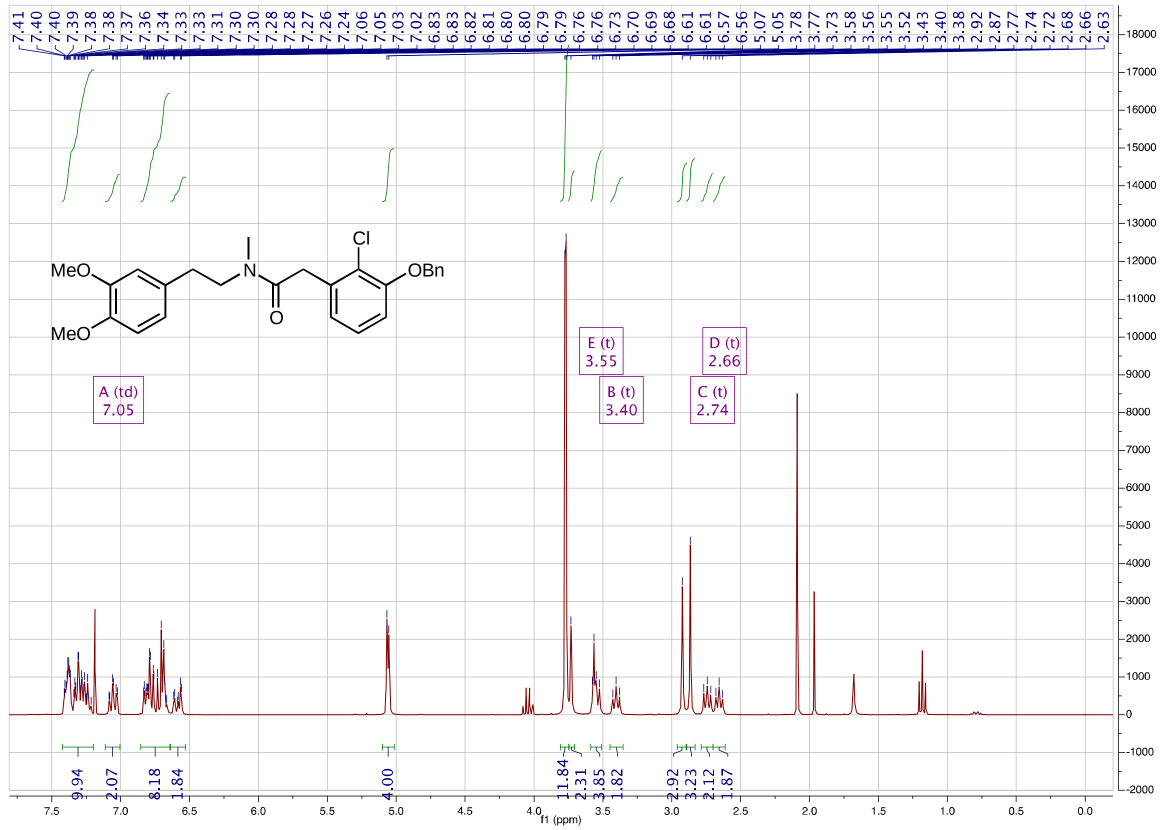

^{13}C-NMR

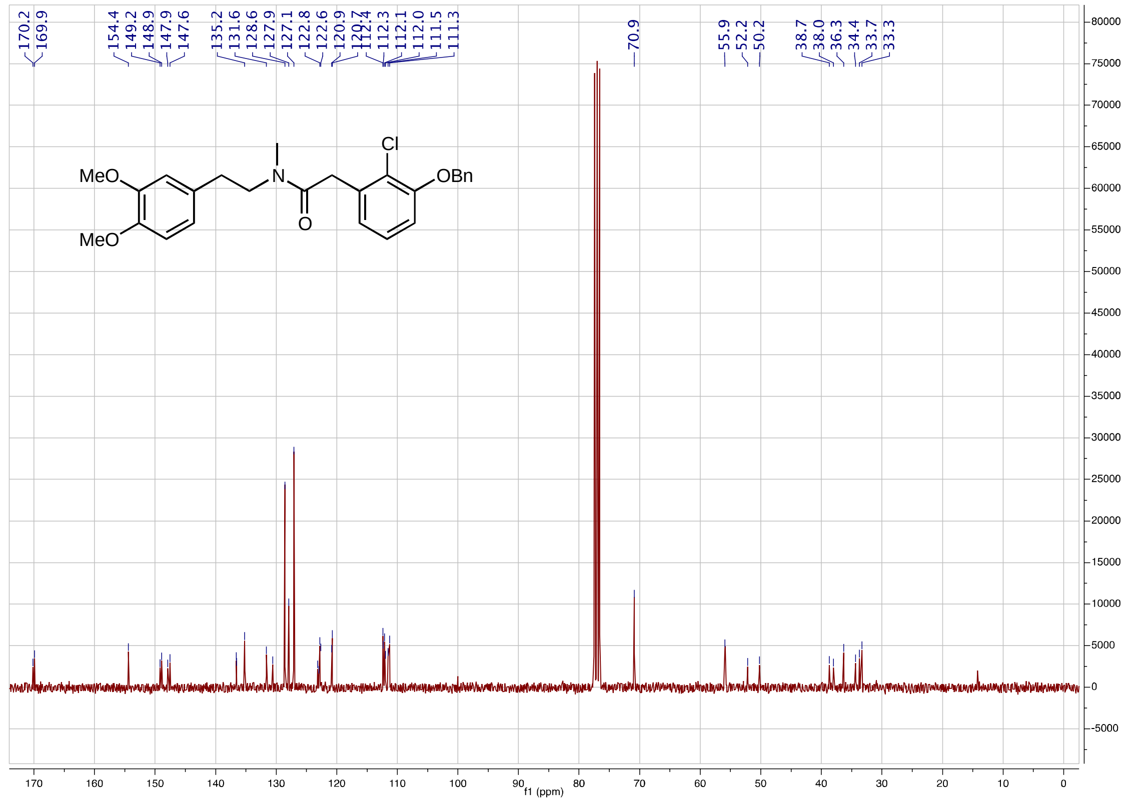

^{13}C-NMR DEPT90 spectrum

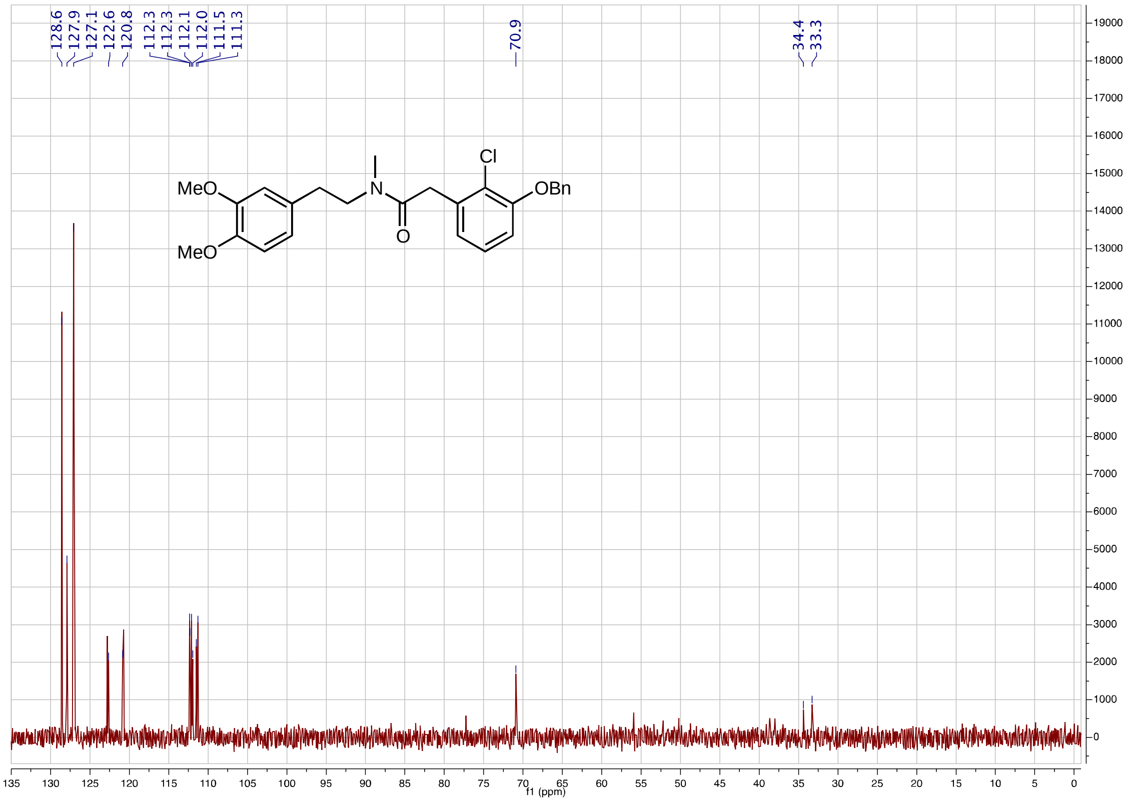

^{13}C-NMR DEPT135 spectrum

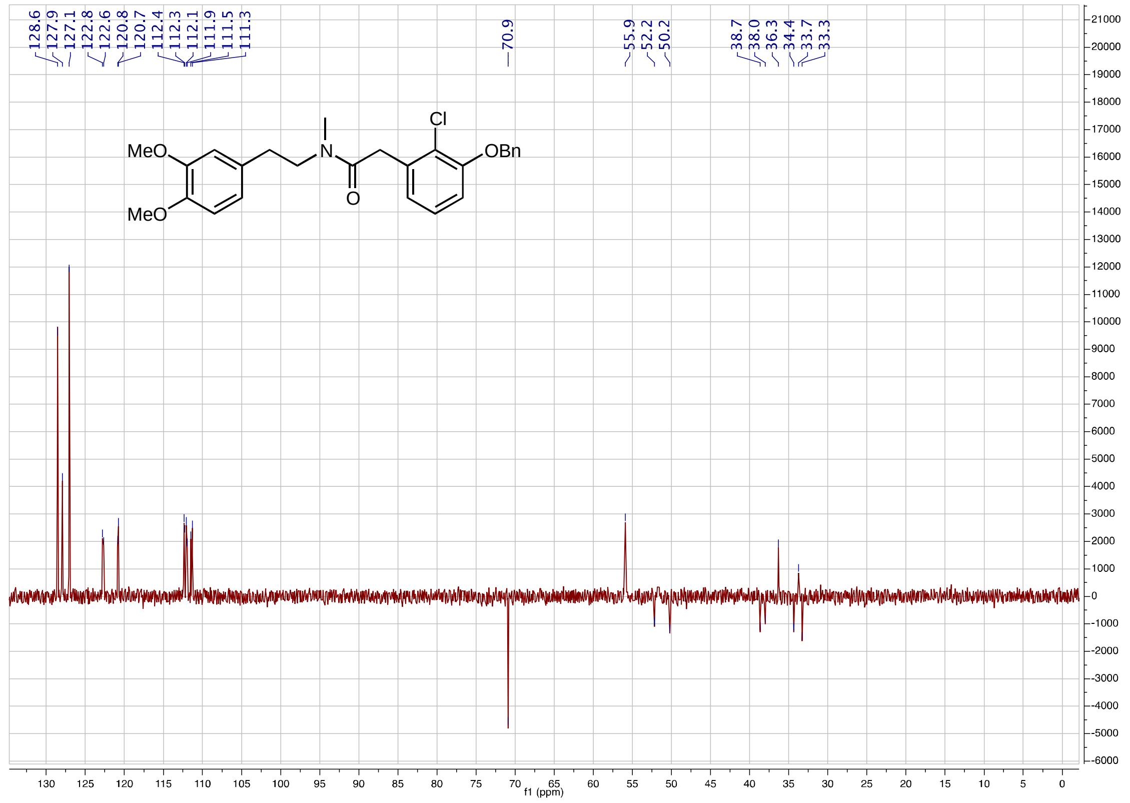

COSY spectrum

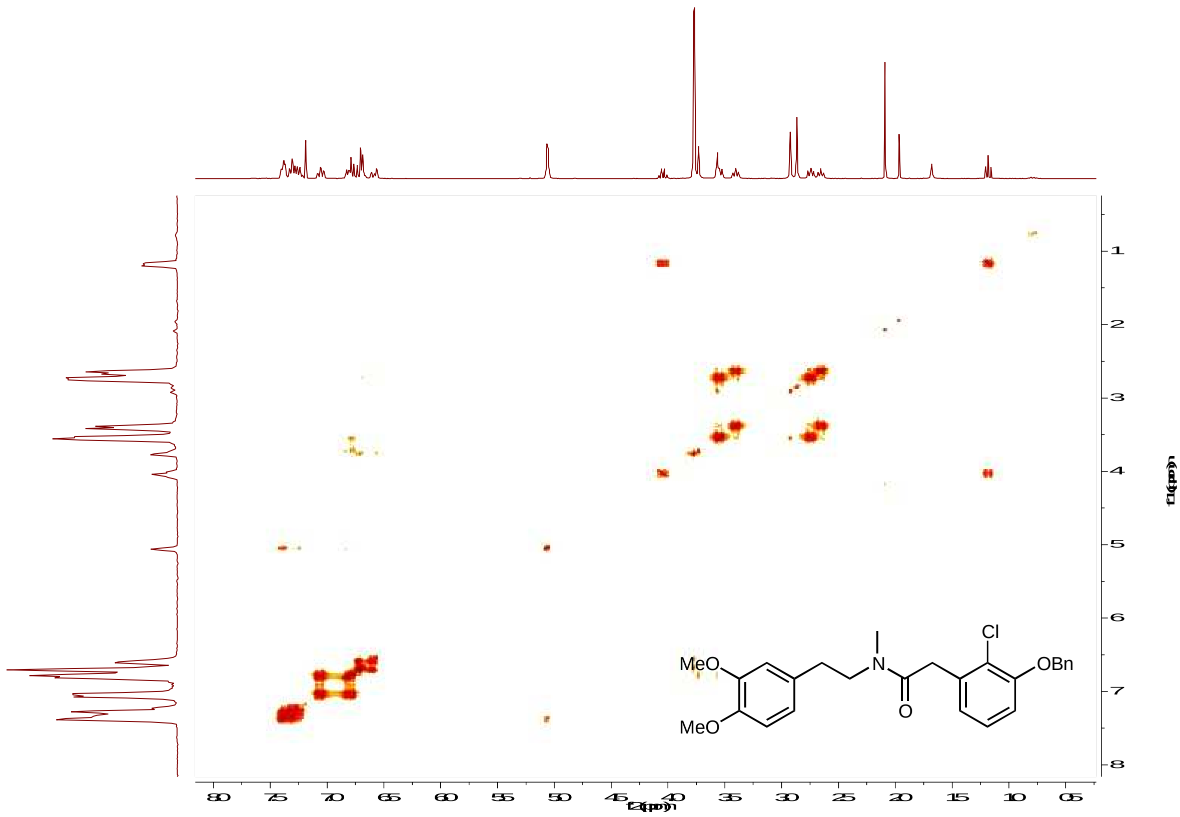

HSQC spectrum

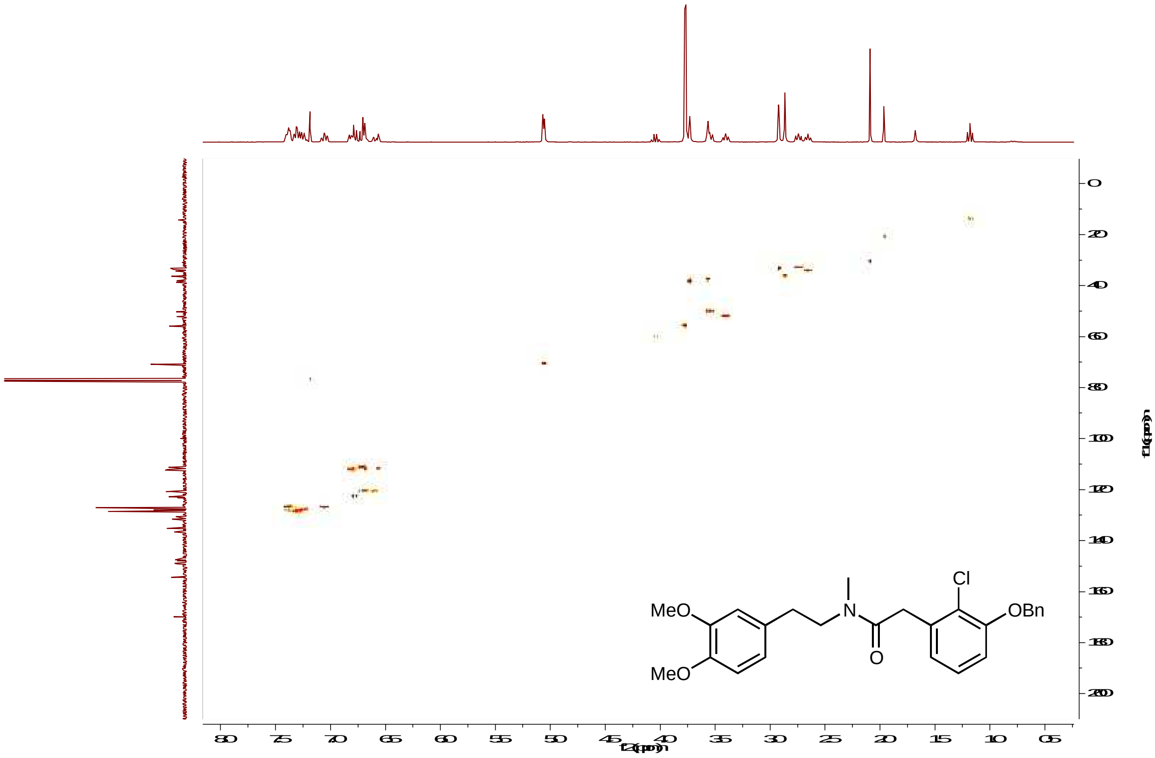

HRMS results

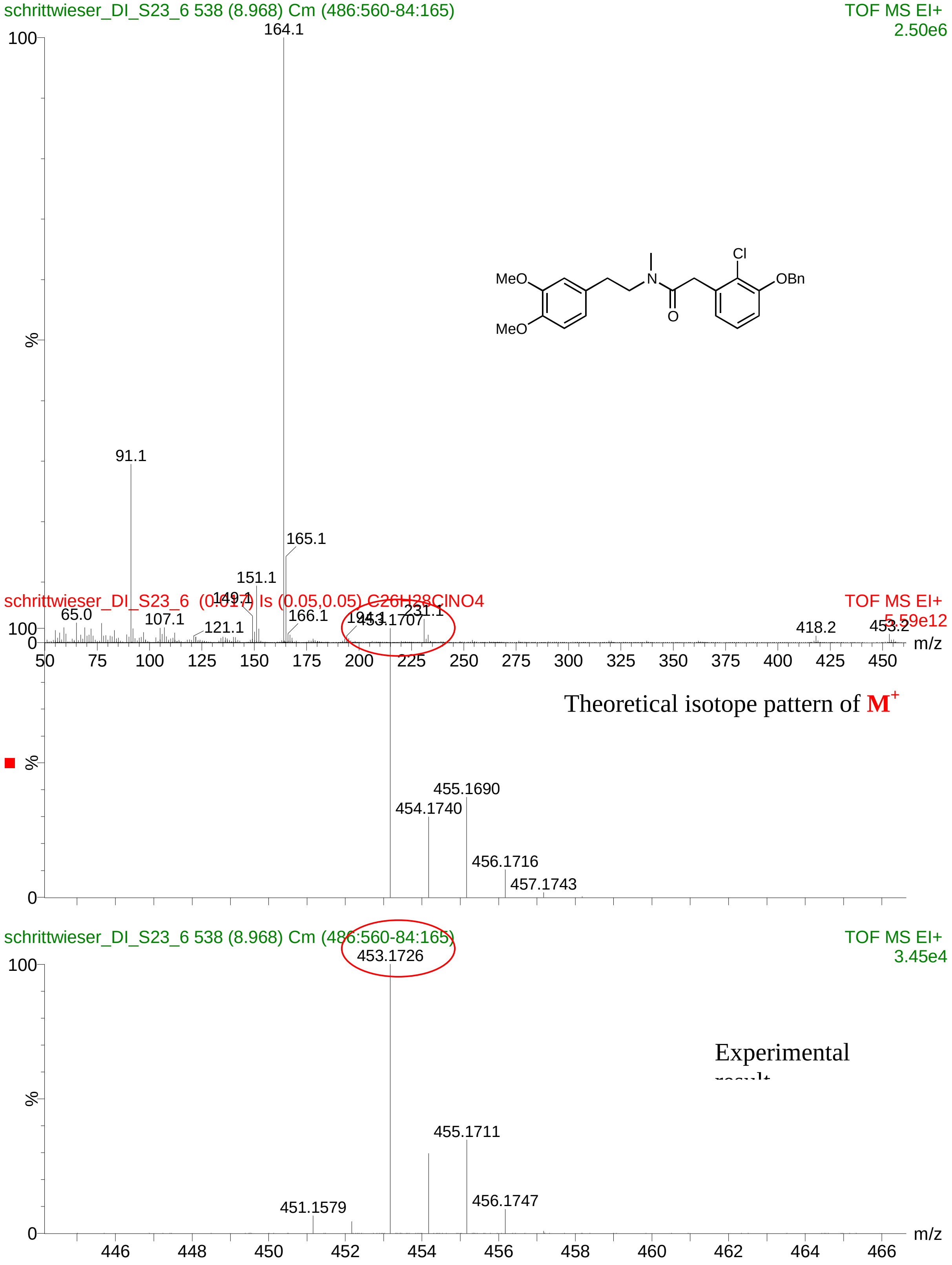

1-(3-(Benzyloxy)-2-chlorobenzyl)-6,7-dimethoxy-2-methyl-1,2,3,4-tetrahydroisoquinoline

^{1}H-NMR

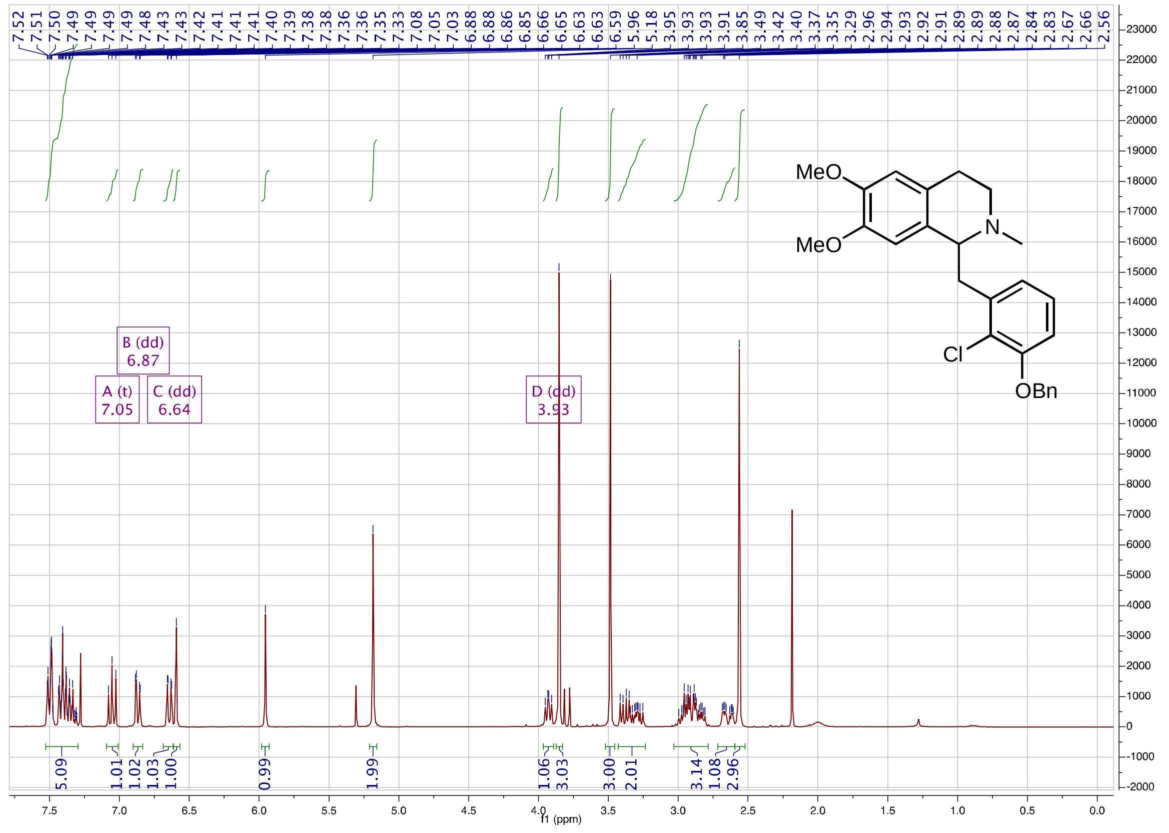

^{13}C-NMR

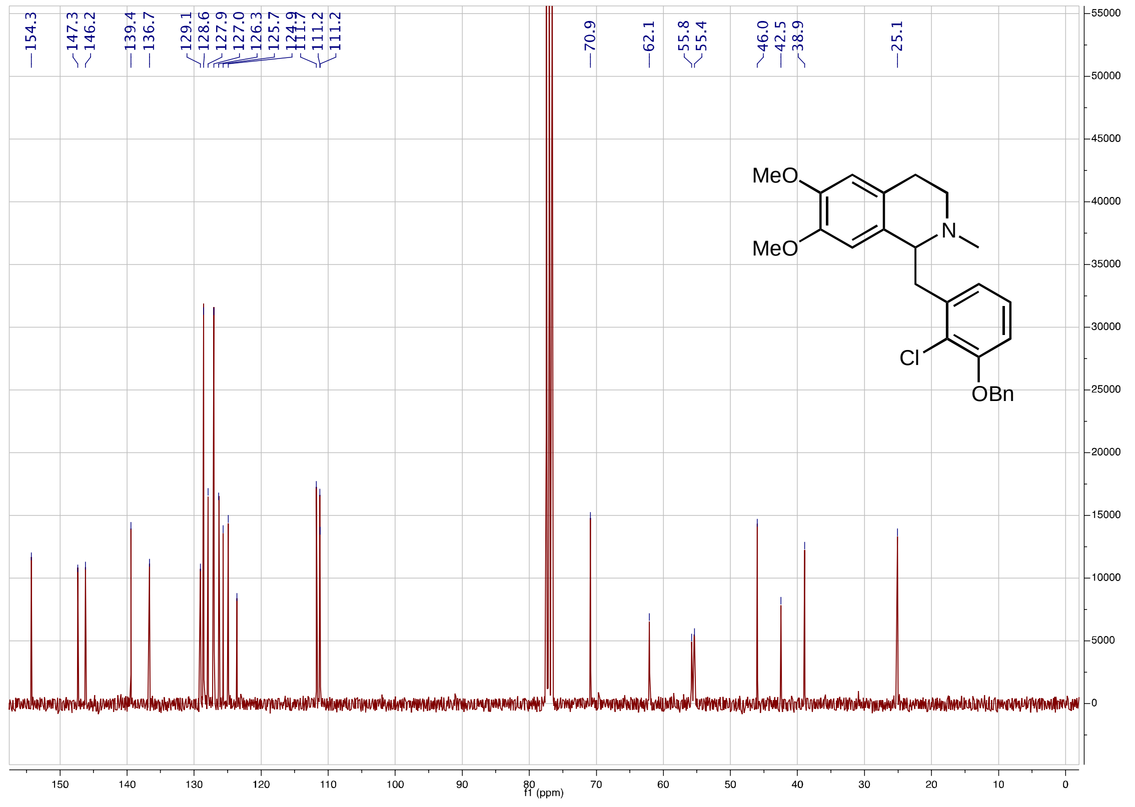

HRMS results

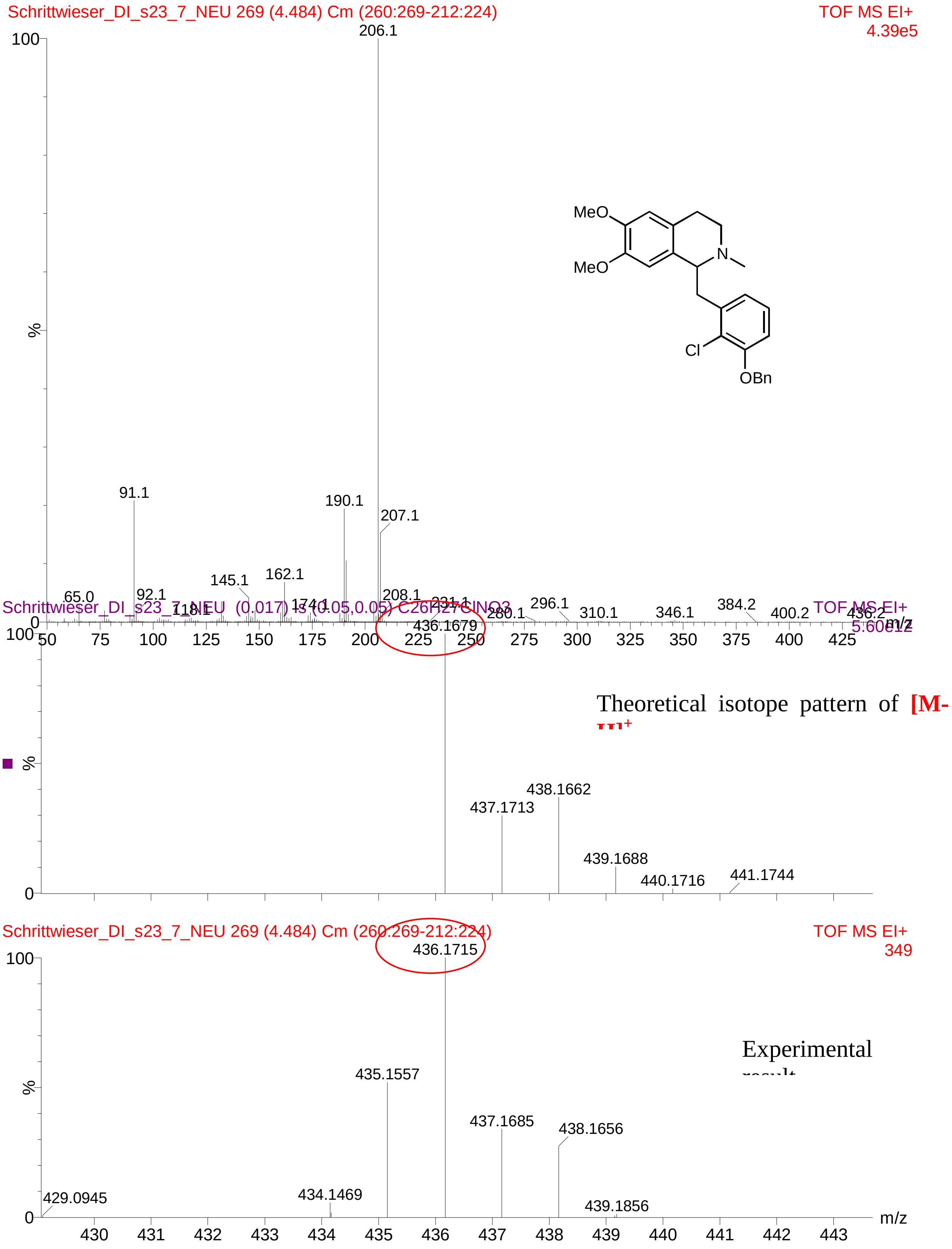

2-Chloro-3-((6,7-dimethoxy-2-methyl-1,2,3,4-tetrahydroisoquinolin-1-yl)methyl)phenol

^{1}H-NMR

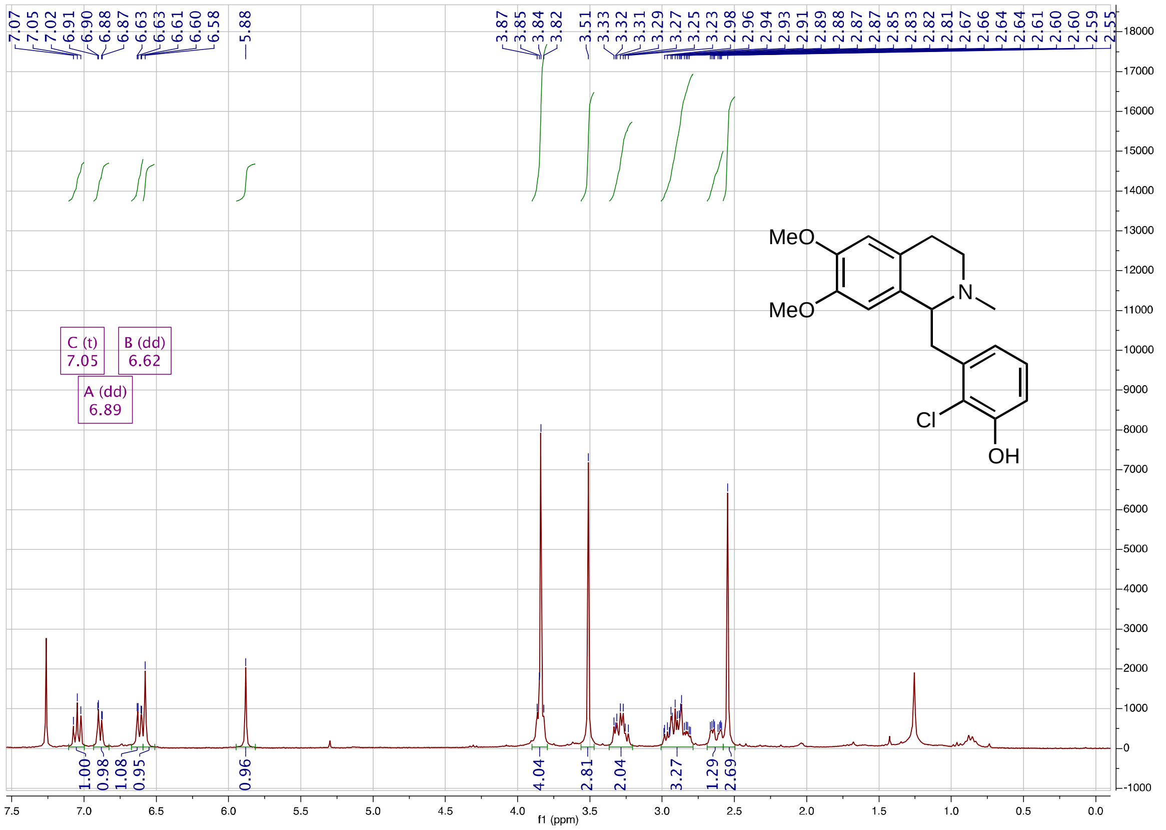

^{13}C-NMR

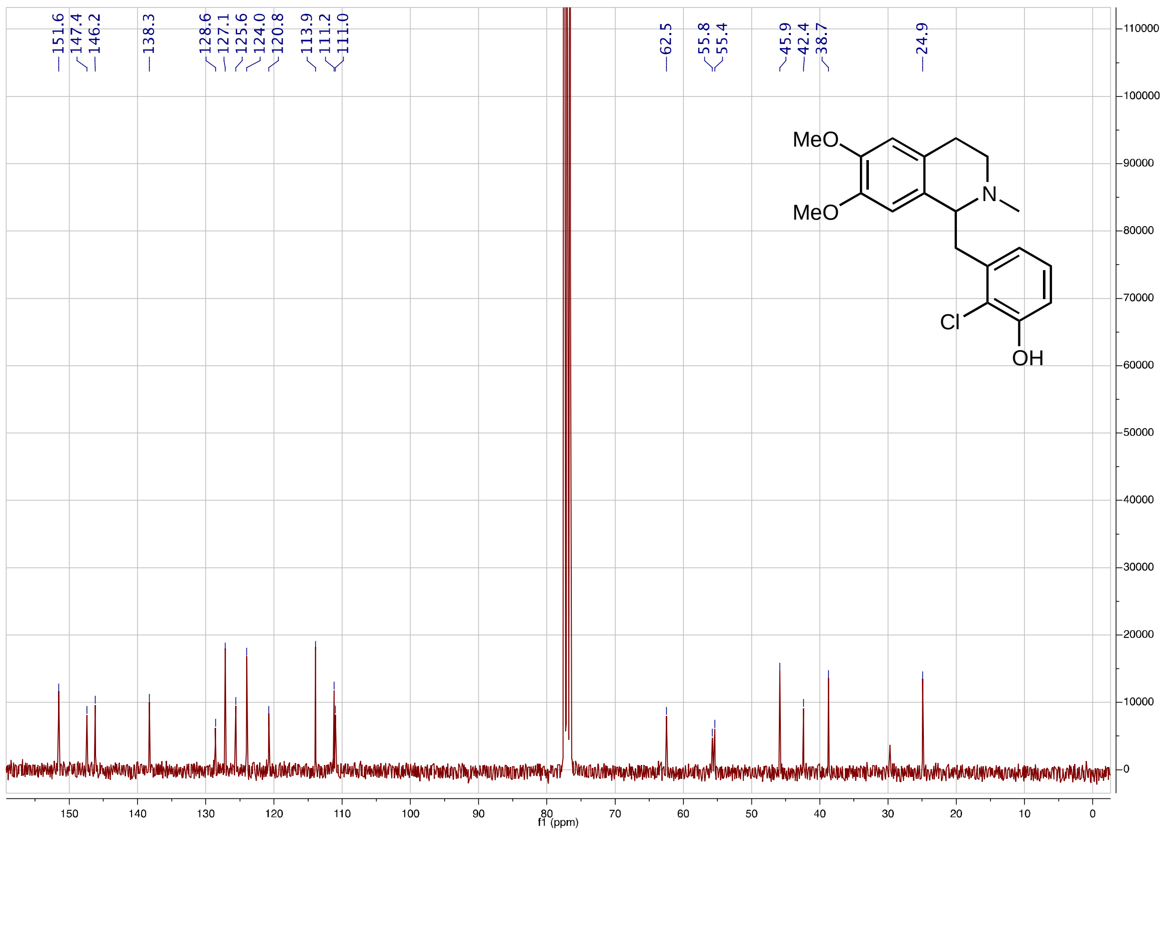

COSY spectrum

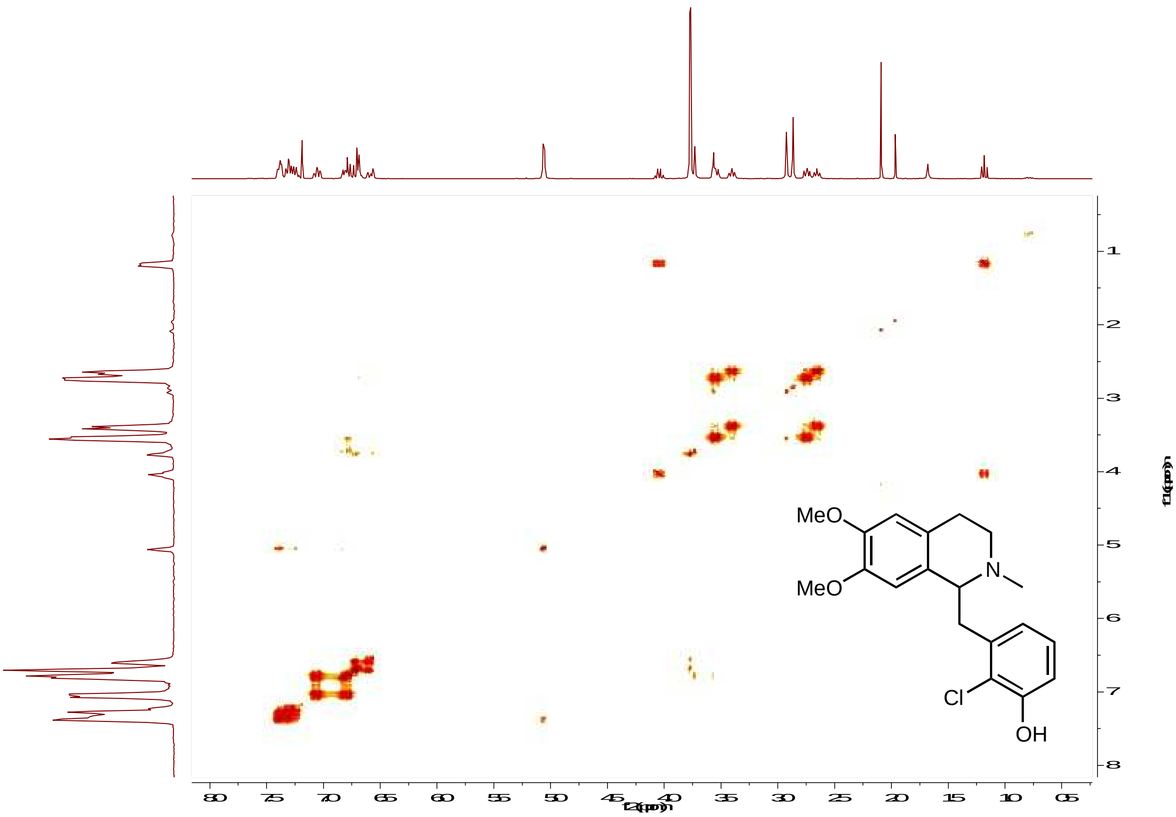

HSQC spectrum

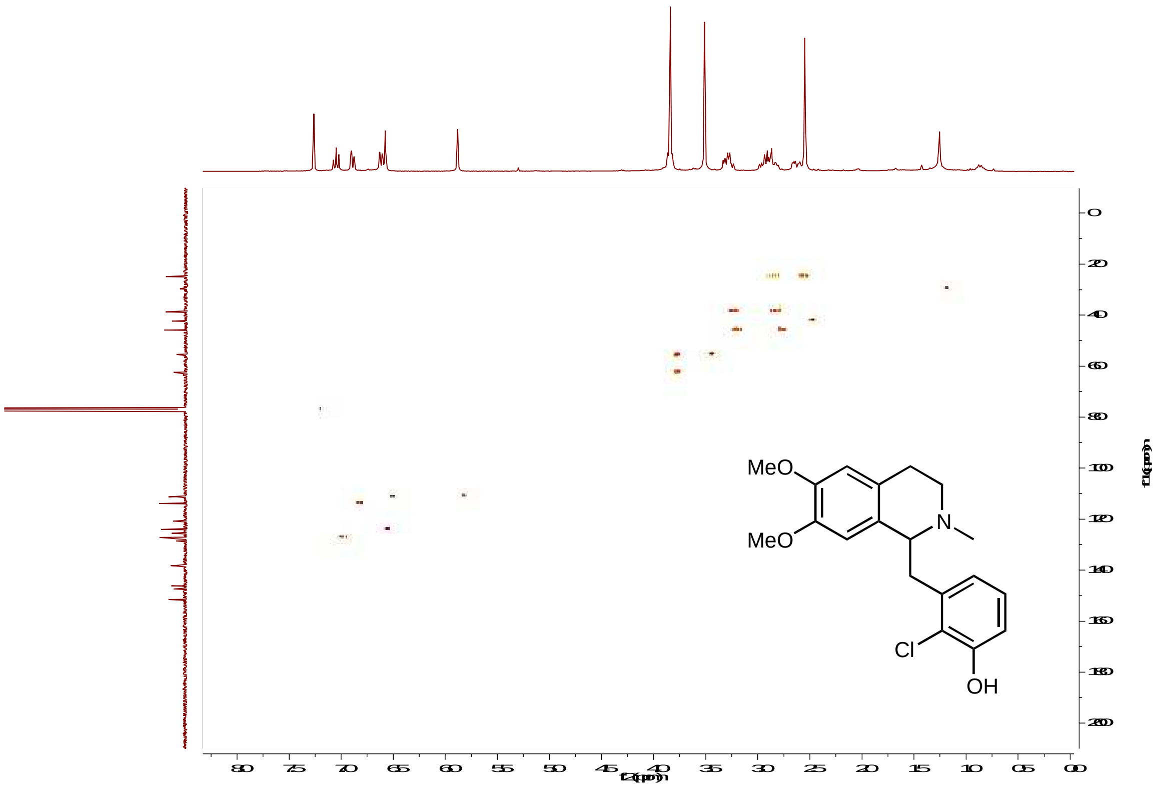

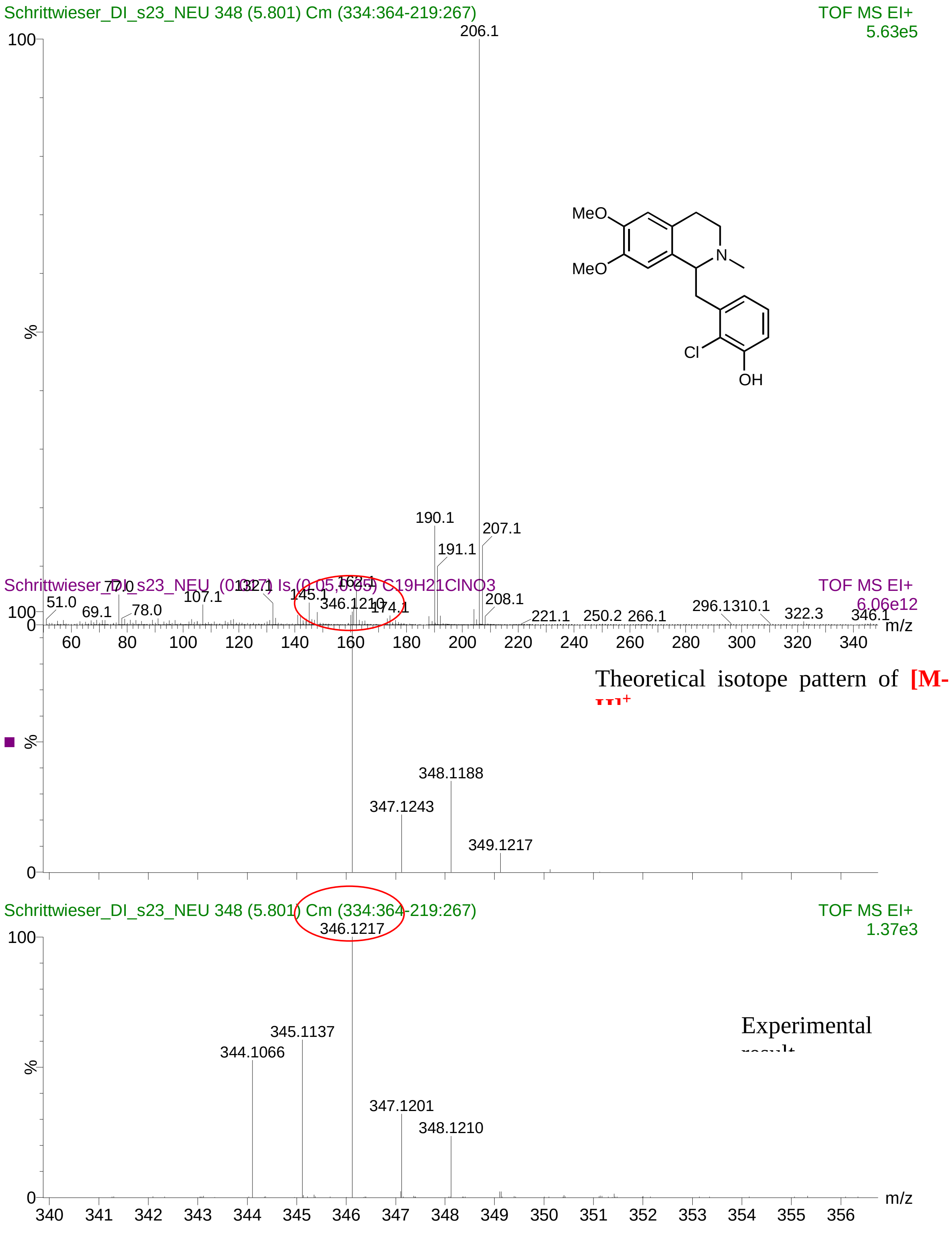
Schrittwieser_DI_s23_NEU 348 (5.801) Cm (334:364-219:267)
TOF MS EI+
5.63e5
MeO
MeO
N
Cl
OH
206.1
190.1
207.1
191.1
208.1
%
Schrittwieser_DI_s23_NEU (0.127) Is (0.05,0.05) C19H21ClNO3
TOF MS EI+
6.06e12
346.1210
347.1243
348.1188
349.1217
Theoretical isotope pattern of [M-
Schrittwieser_DI_s23_NEU 348 (5.801) Cm (334:364-219:267)
TOF MS EI+
1.37e3
346.1217
345.1137
344.1066
347.1201
348.1210
Experimental
m/z

Synthesis of **1m**:

Provided Material:

3-Methoxy-2-methylbenzaldehyde
^{1}H-NMR spectrum, ^{13}C-NMR spectrum

2,2,2-Trichloro-1-(3-methoxy-2-methylphenyl)ethanol
^{1}H-NMR spectrum, ^{13}C-NMR spectrum, HRMS results

2-(3-Methoxy-2-methylphenyl)acetic acid
^{1}H-NMR spectrum, ^{13}C-NMR spectrum

2-(3-Hydroxy-2-methylphenyl)acetic acid
^{1}H-NMR spectrum, ^{13}C-NMR spectrum, HRMS results

2-(3-(Benzyloxy)-2-methylphenyl)acetic acid
^{1}H-NMR spectrum, ^{13}C-NMR spectrum, HRMS results

2-(3-(Benzyloxy)-2-methylphenyl)-*N*-(3,4-dimethoxyphenethyl)-*N*-methylacetamide
^{1}H-NMR spectrum, ^{13}C-NMR spectrum, ^{13}C-NMR DEPT135 spectrum, ^{13}C-NMR DEPT90 spectrum, COSY spectrum, HSQC spectrum

1-(3-(Benzyloxy)-2-methylbenzyl)-6,7-dimethoxy-2-methyl-1,2,3,4-tetrahydroisoquinoline
^{1}H-NMR spectrum, ^{13}C-NMR spectrum, COSY spectrum, HSQC spectrum, HRMS results

3-((6,7-Dimethoxy-2-methyl-1,2,3,4-tetrahydroisoquinolin-1-yl)methyl)-2-methylphenol
^{1}H-NMR spectrum, ^{13}C-NMR spectrum, COSY spectrum, HSQC spectrum, HRMS results

3-Methoxy-2-methylbenzaldehyde

^{1}H-NMR

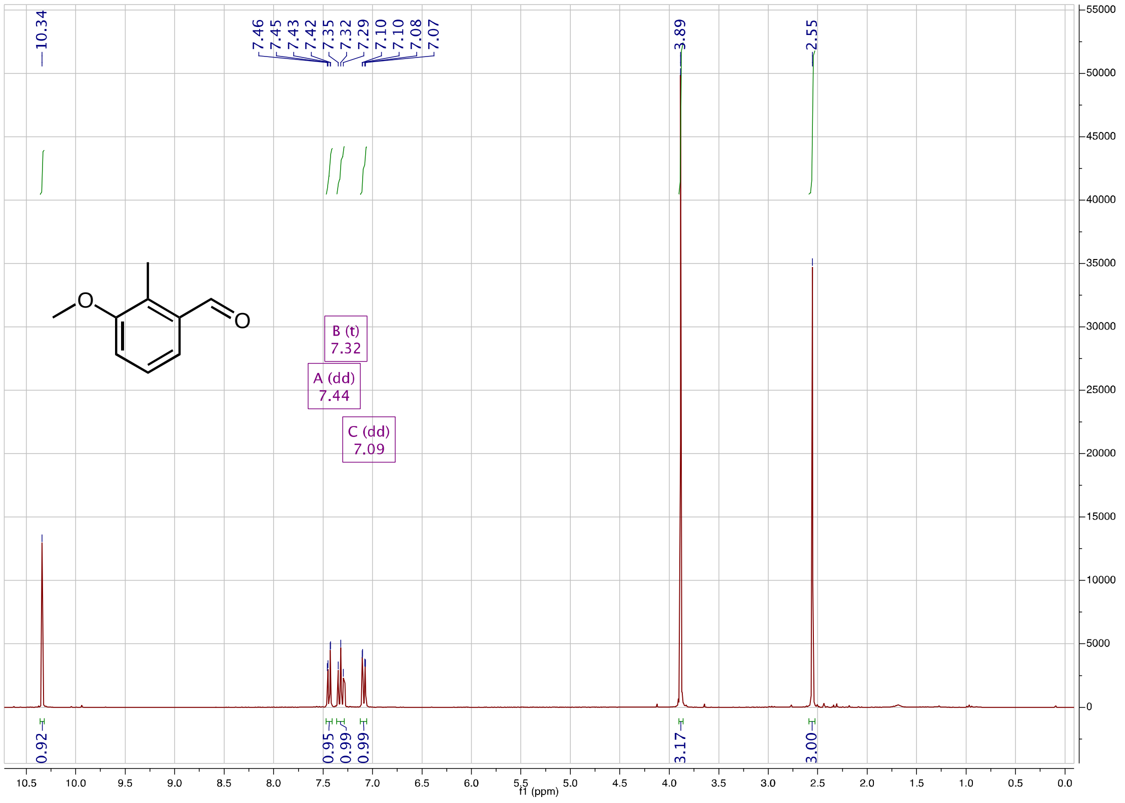

^{13}C-NMR

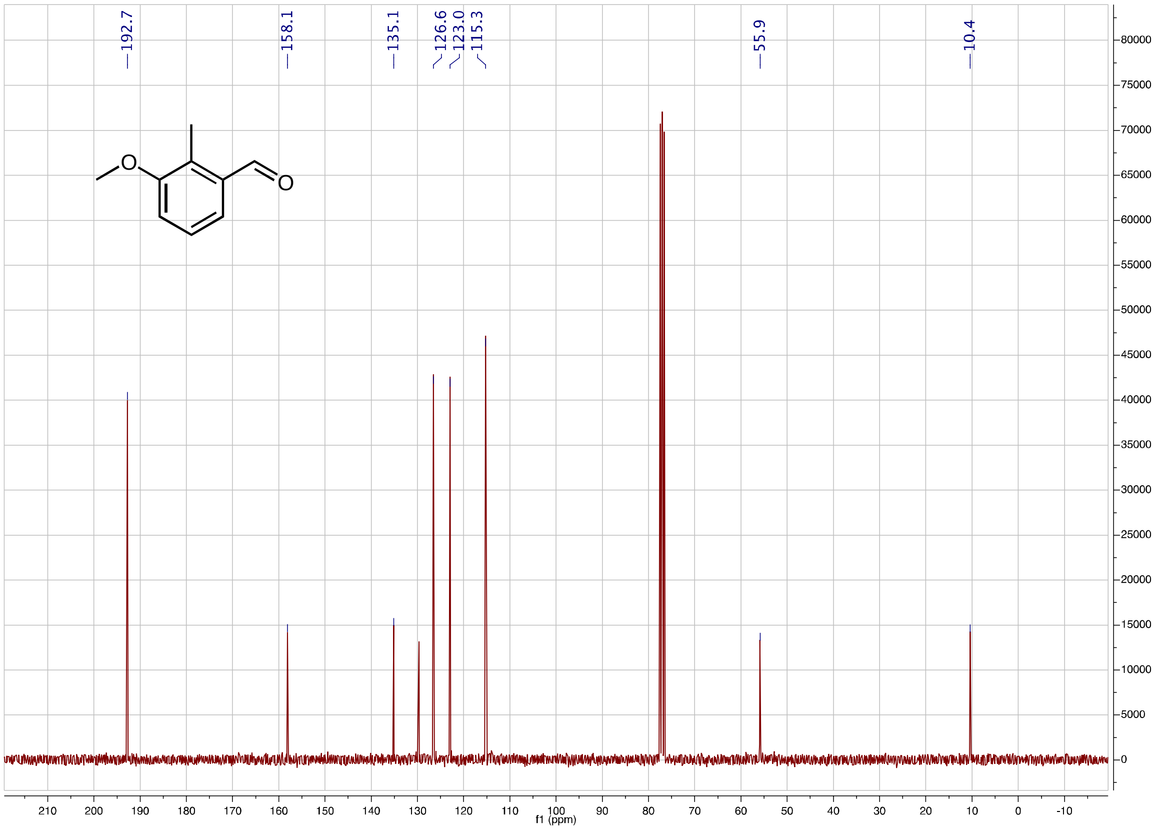

2,2,2-Trichloro-1-(3-methoxy-2-methylphenyl)ethanol

^{1}H-NMR

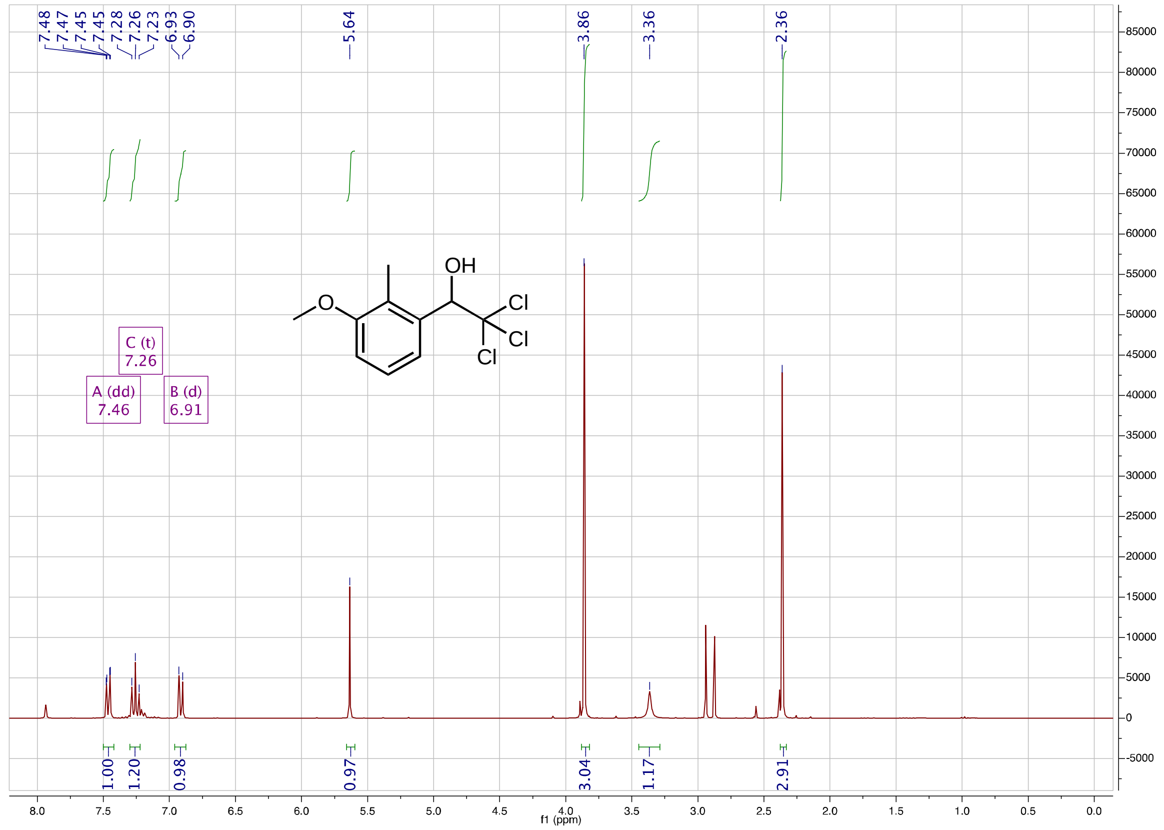

^{13}C-NMR

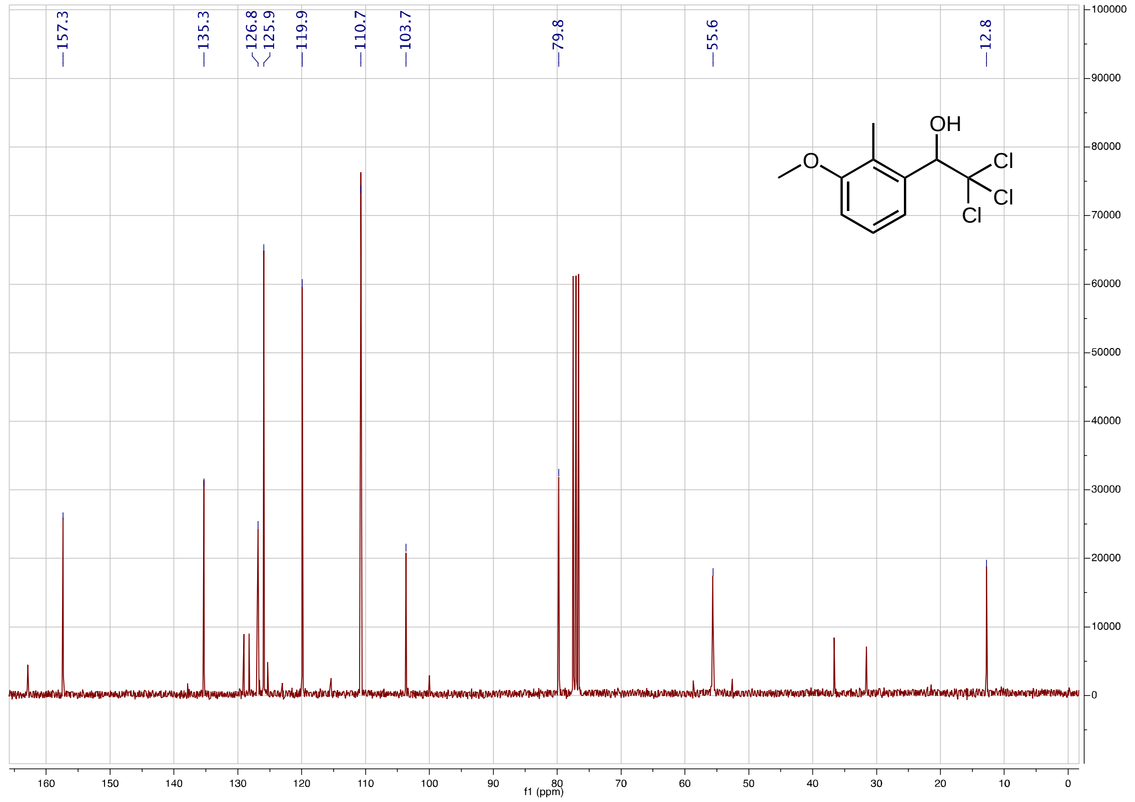

HRMS results

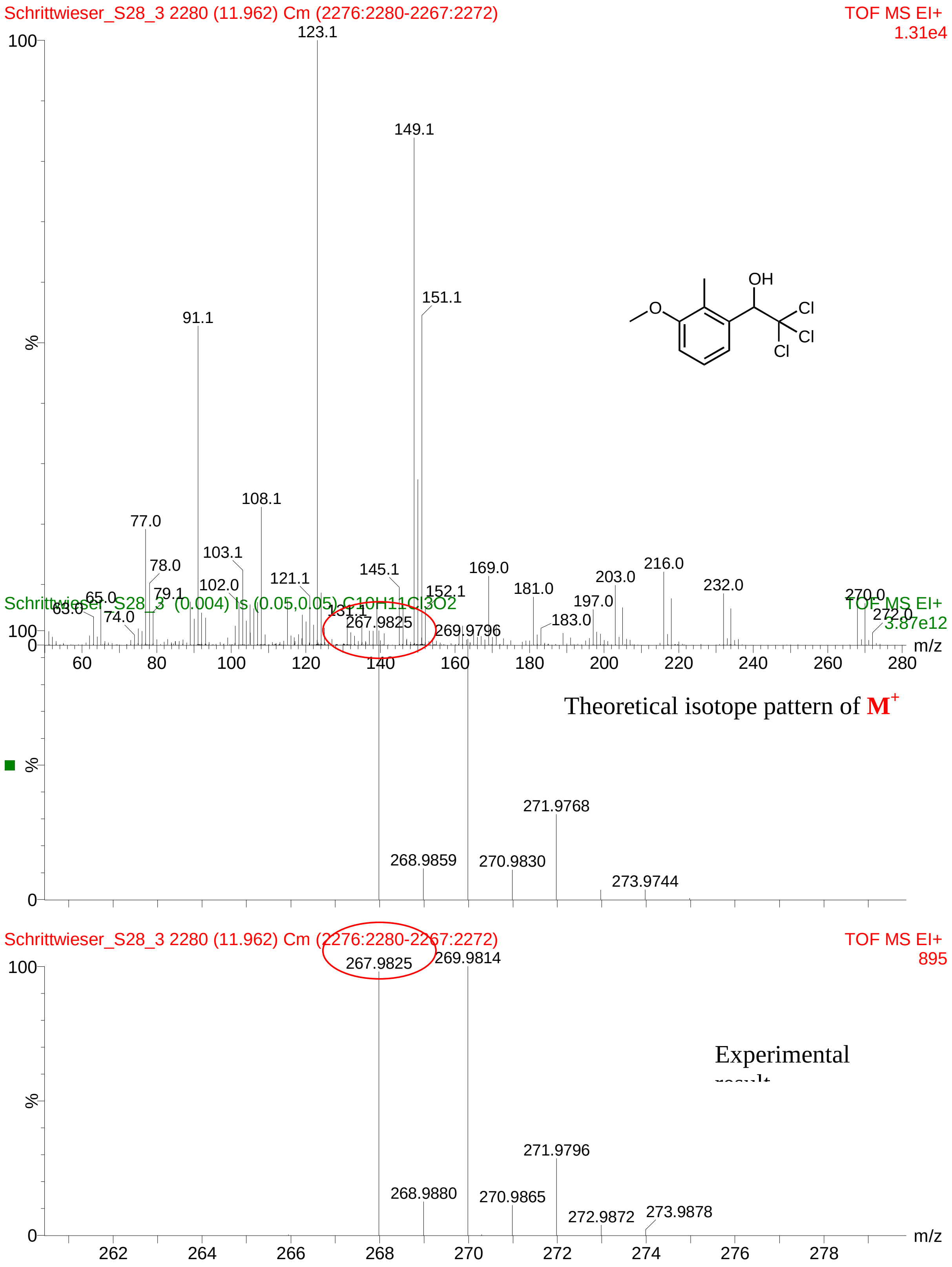

2-(3-Methoxy-2-methylphenyl)acetic acid

^{1}H-NMR

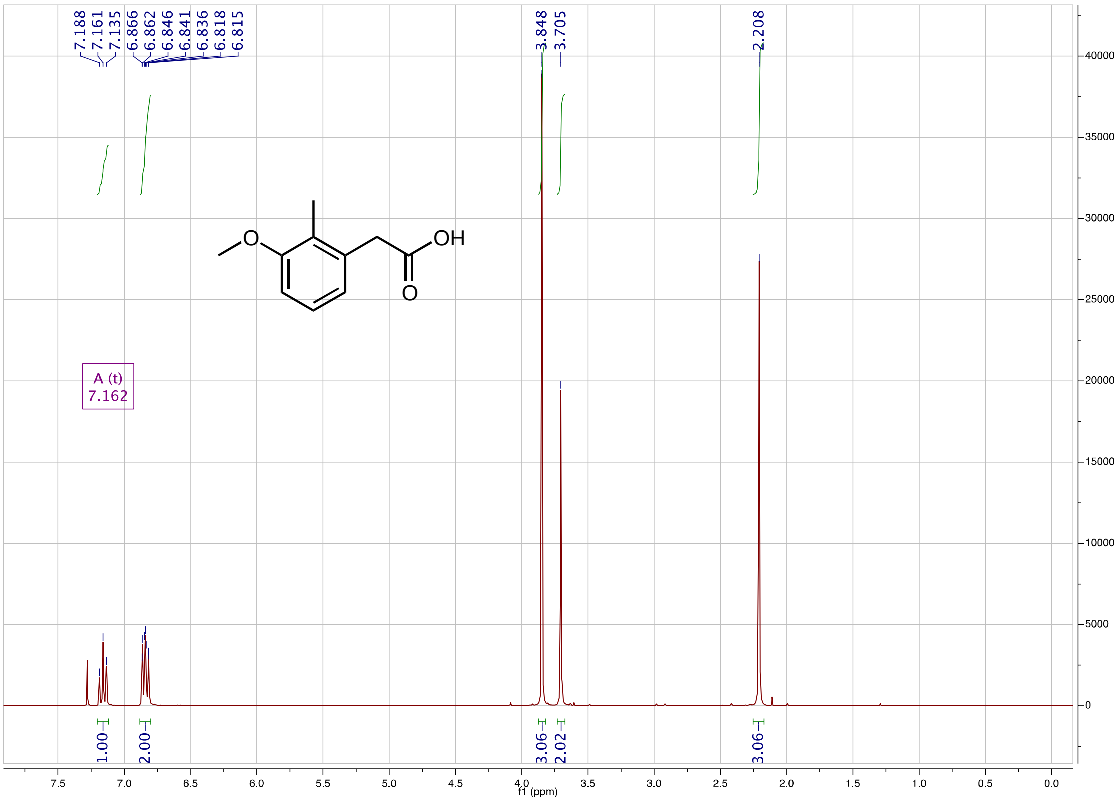

^{13}C-NMR

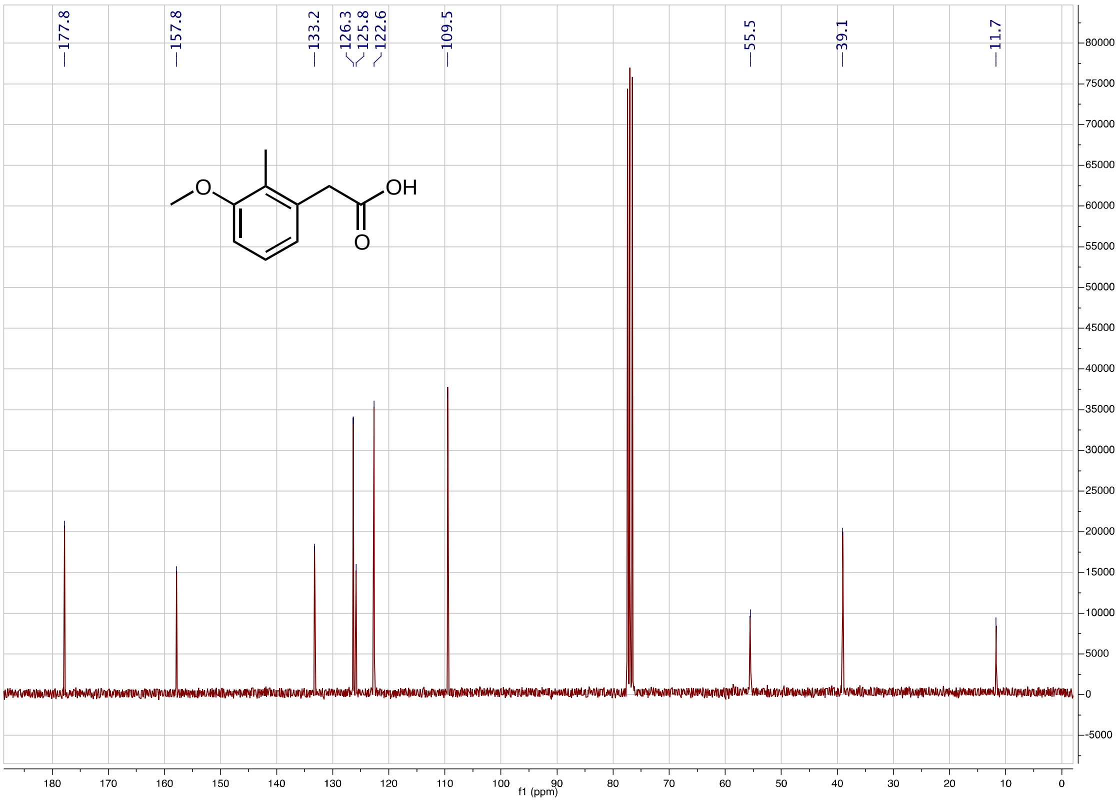

2-(3-Hydroxy-2-methylphenyl)acetic acid

^{1}H-NMR

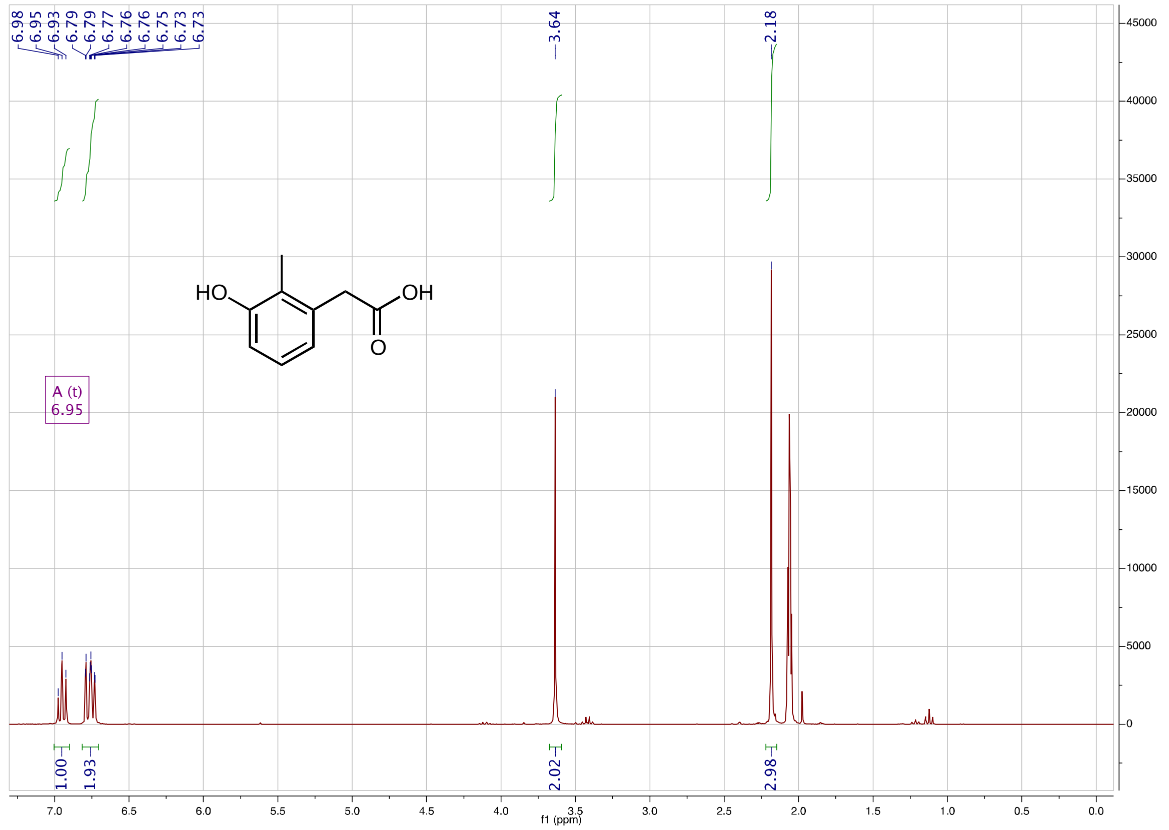

^{13}C-NMR

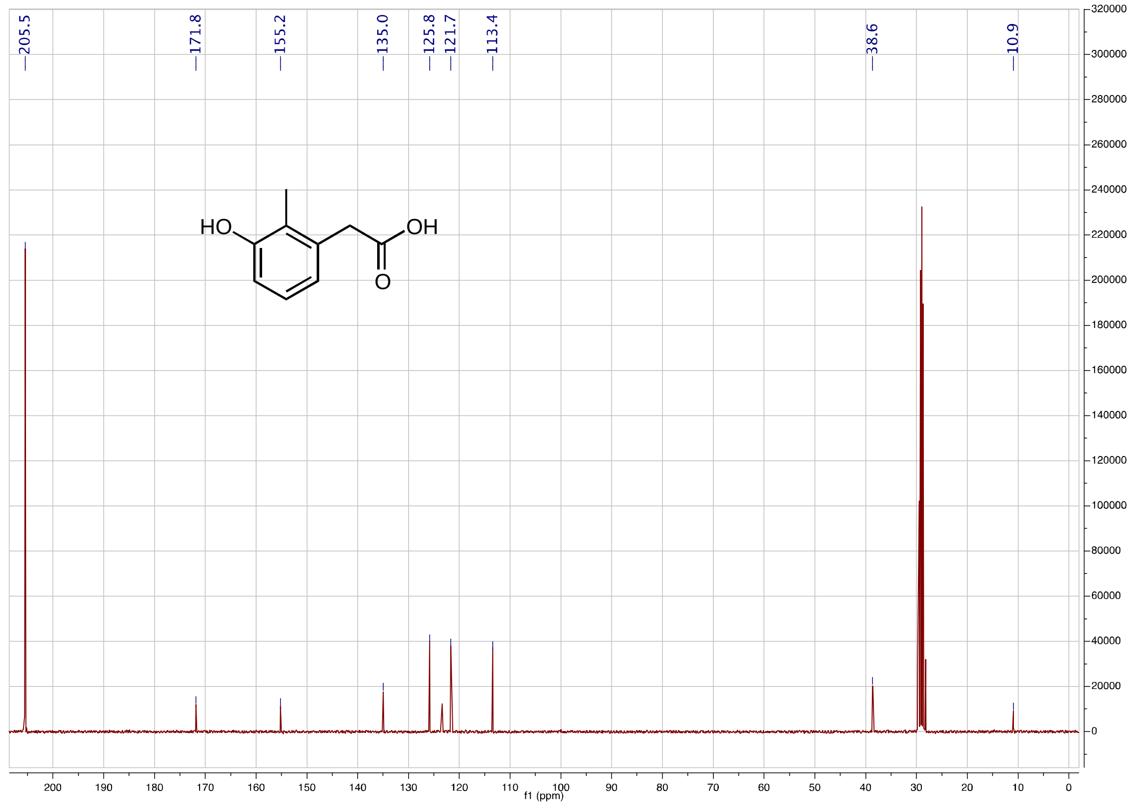

2-(3-(Benzyloxy)-2-methylphenyl)acetic acid

^{1}H-NMR

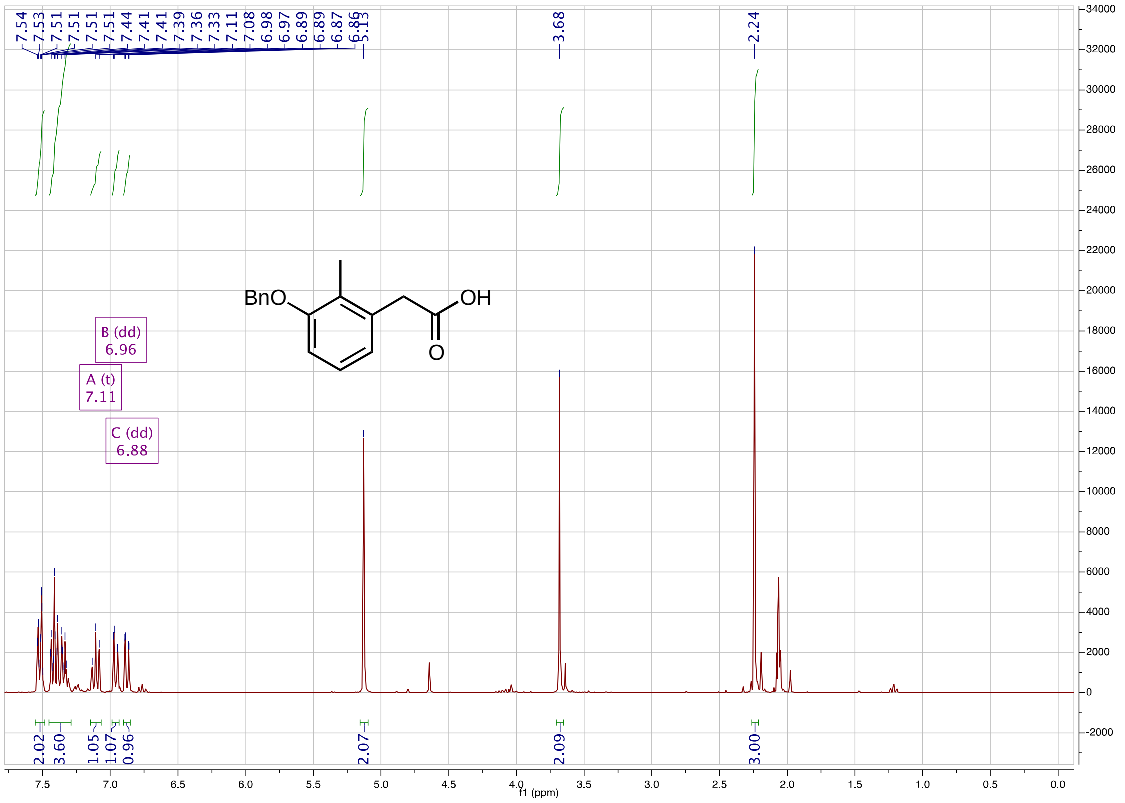

^{13}C-NMR

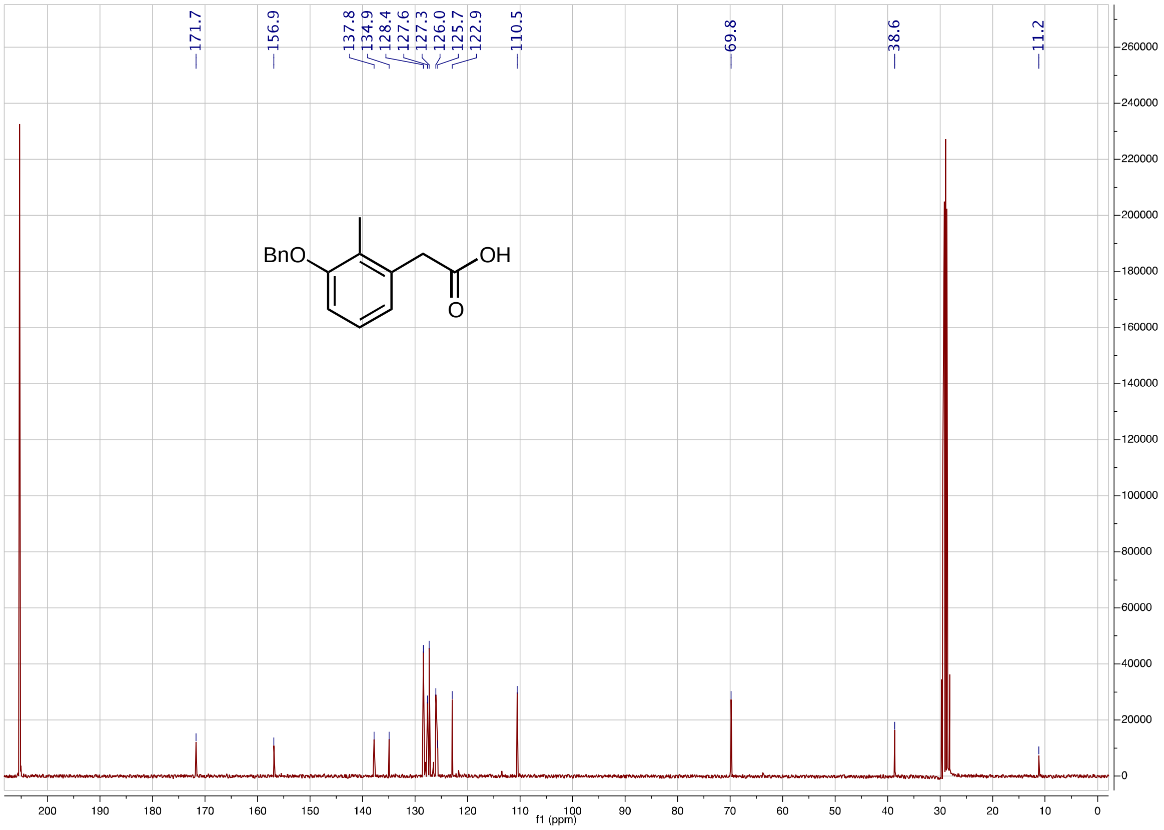

HRMS results

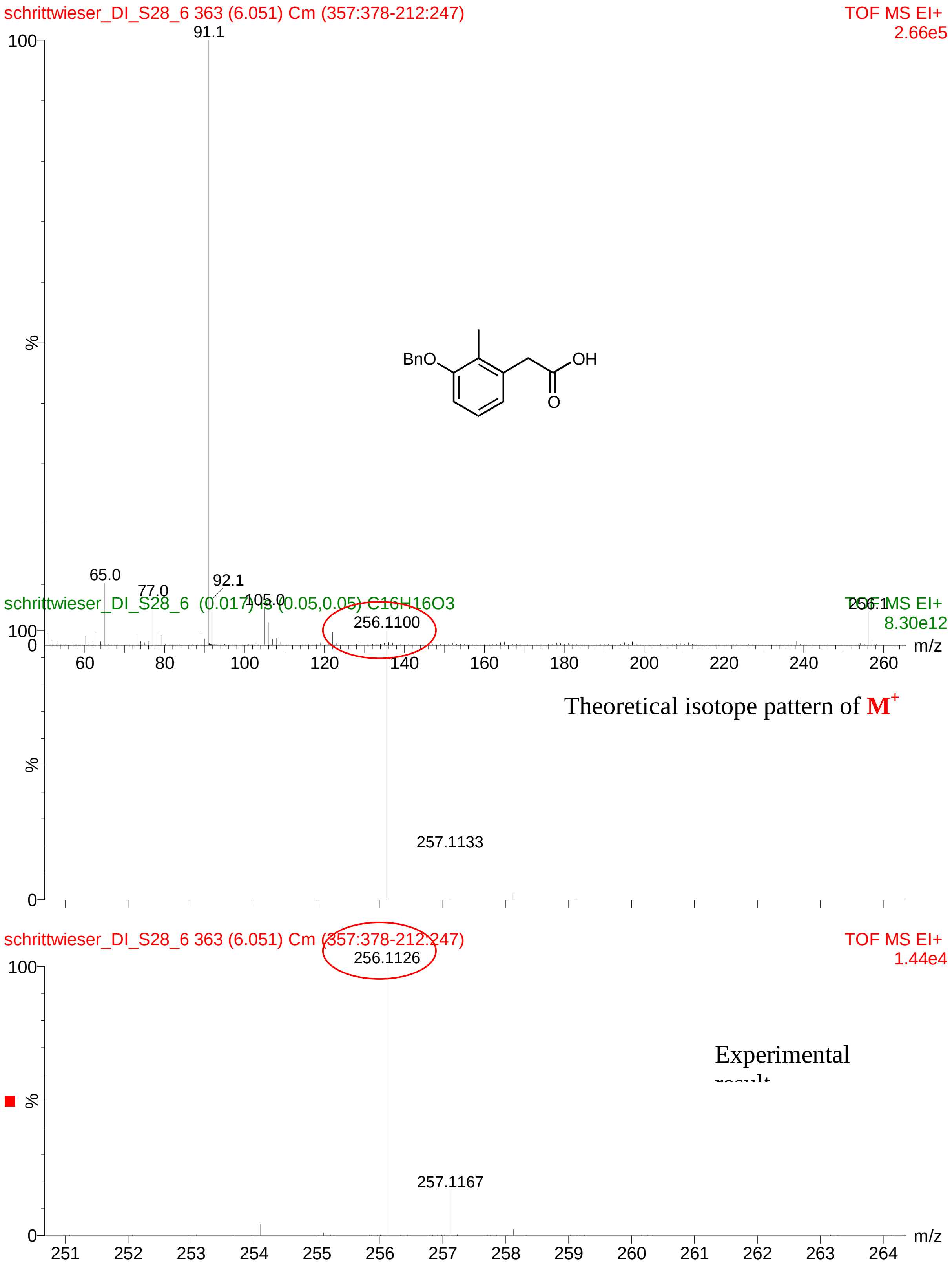

2-(3-(Benzyloxy)-2-methylphenyl)-*N*-(3,4-dimethoxyphenethyl)-*N*-methylacetamide

^{1}H-NMR

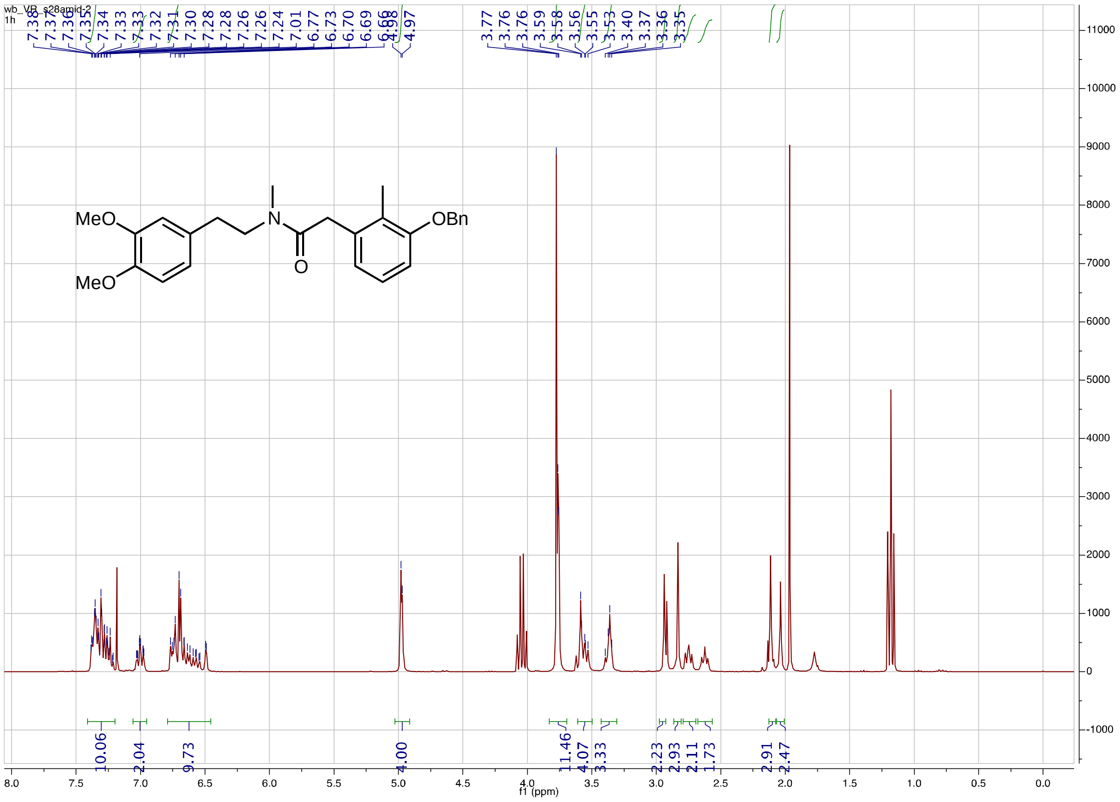

^{13}C-NMR

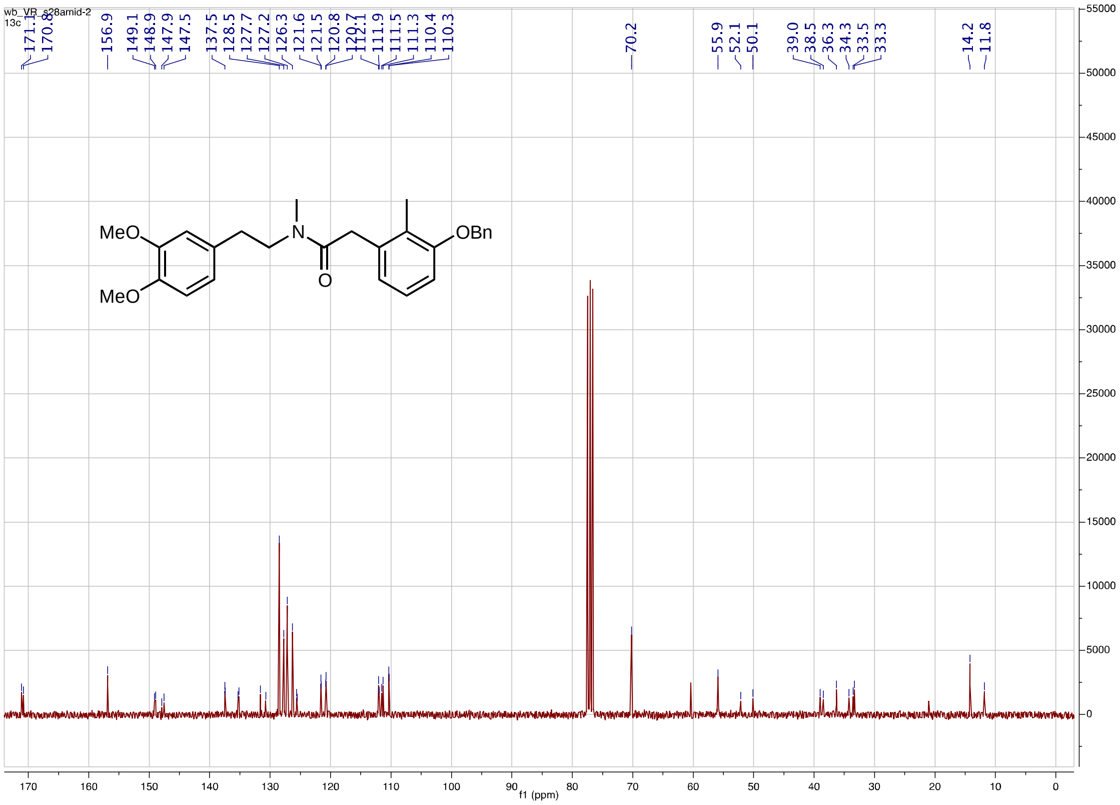

^{13}C-NMR DEPT90 spectrum

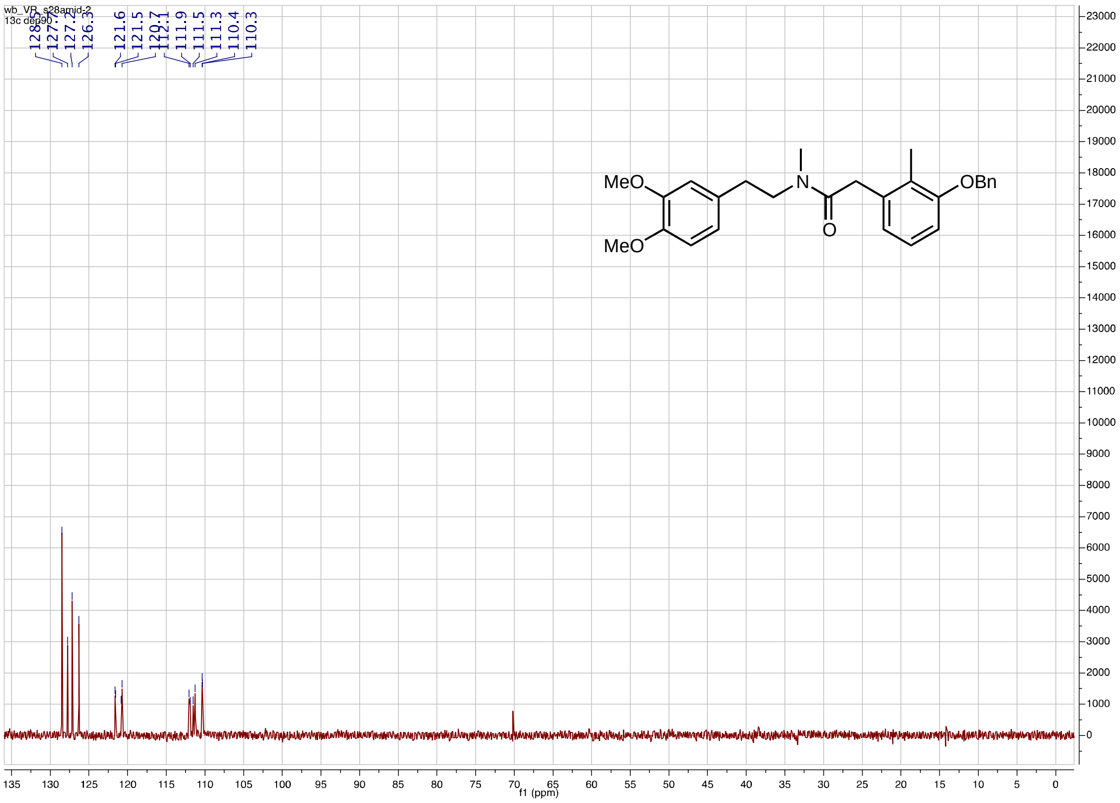

^{13}C-NMR DEPT135 spectrum

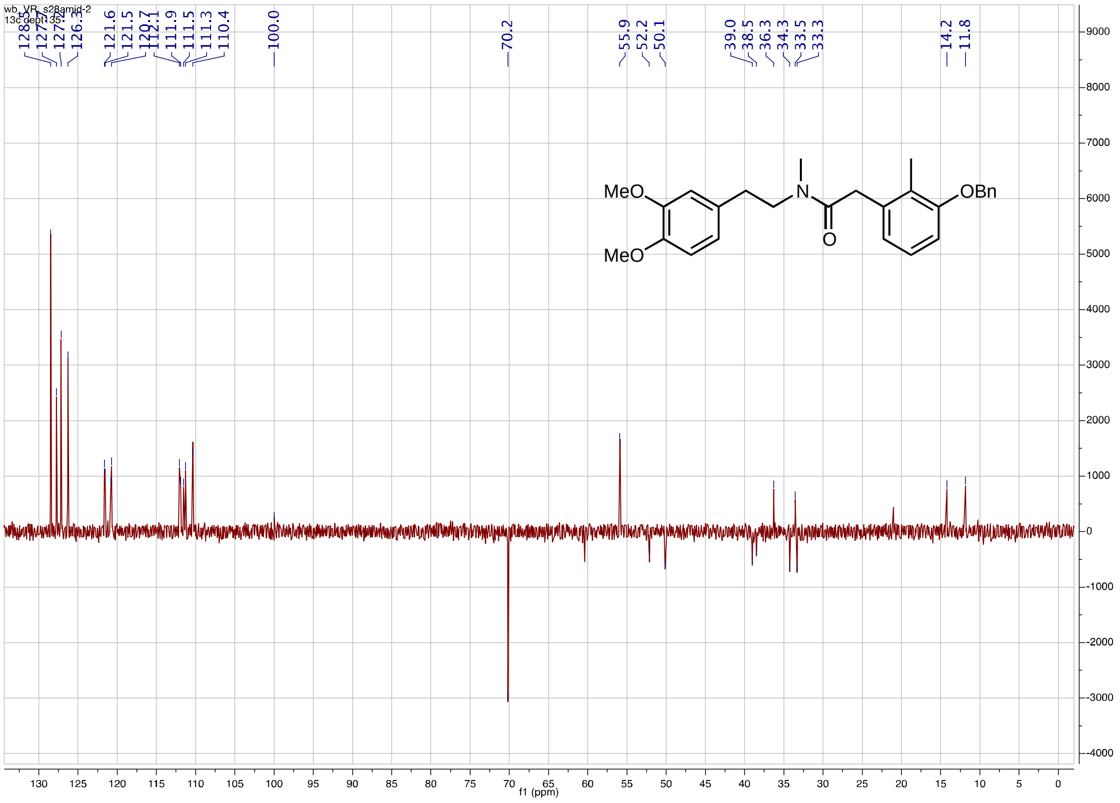

COSY spectrum

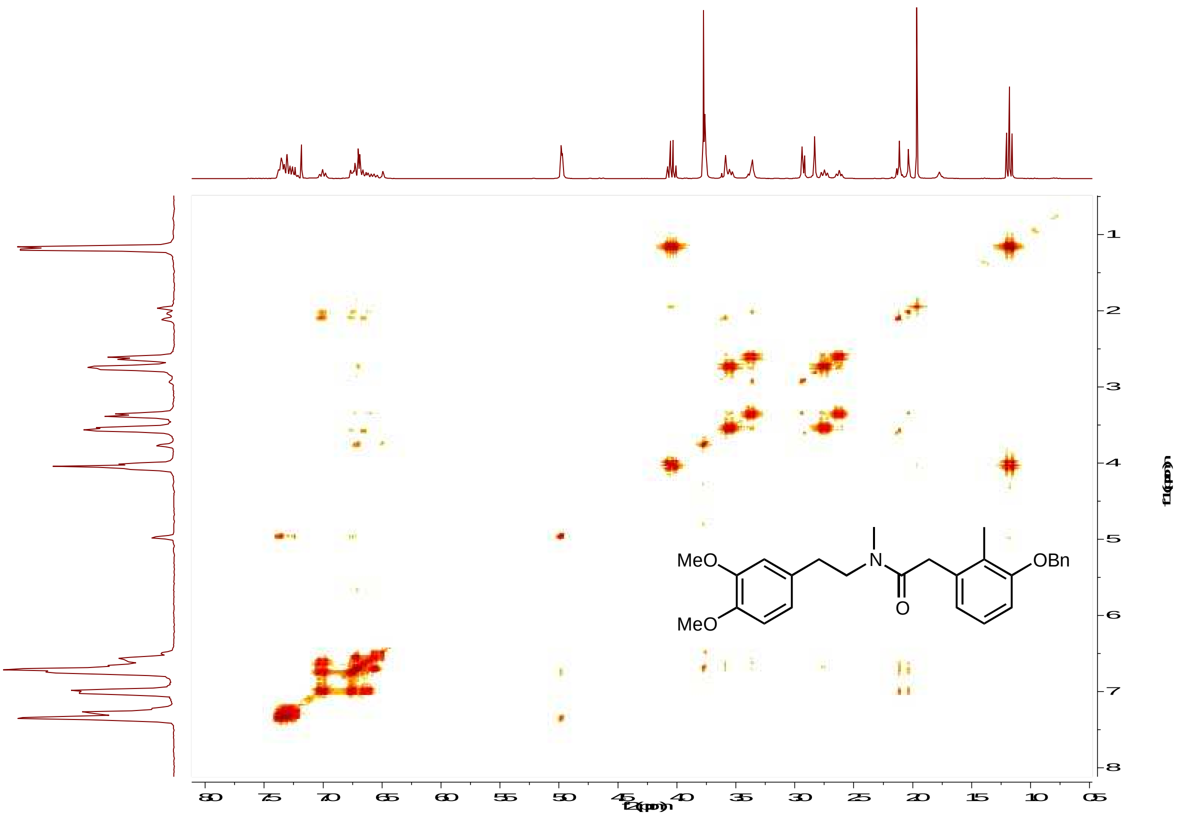

HSQC spectrum

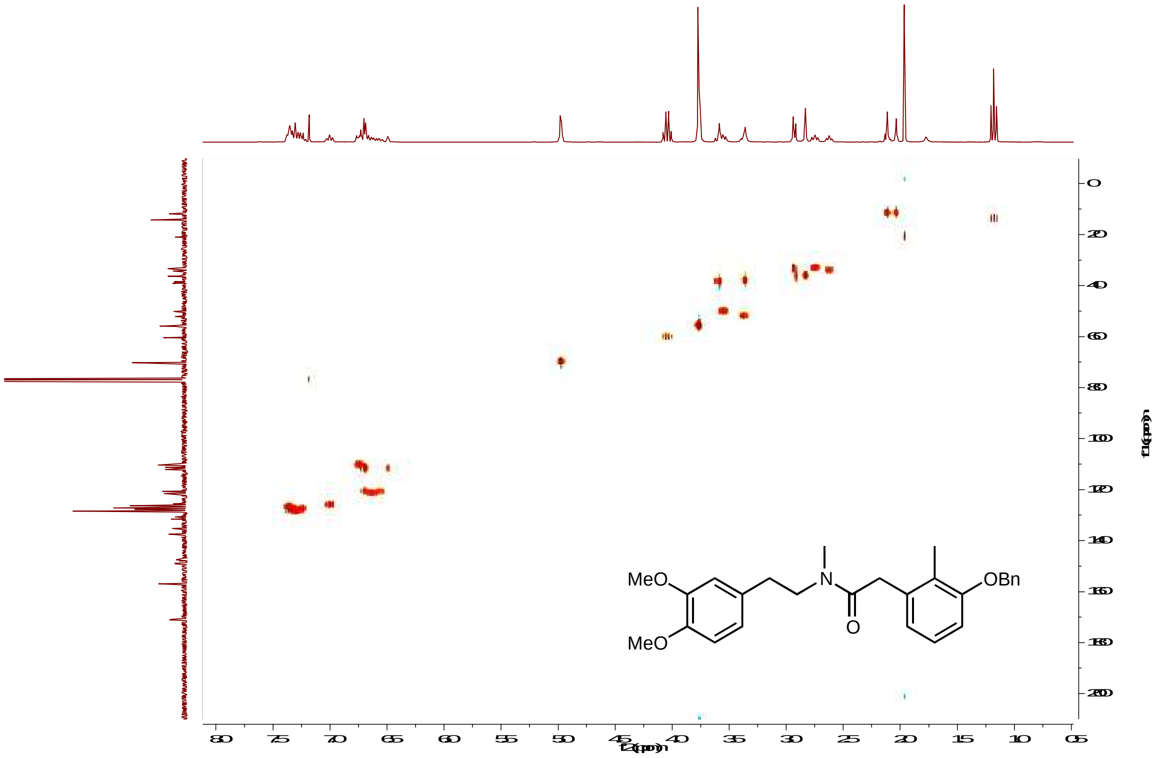

1-(3-(Benzyloxy)-2-methylbenzyl)-6,7-dimethoxy-2-methyl-1,2,3,4-tetrahydroisoquinoline

^{1}H-NMR

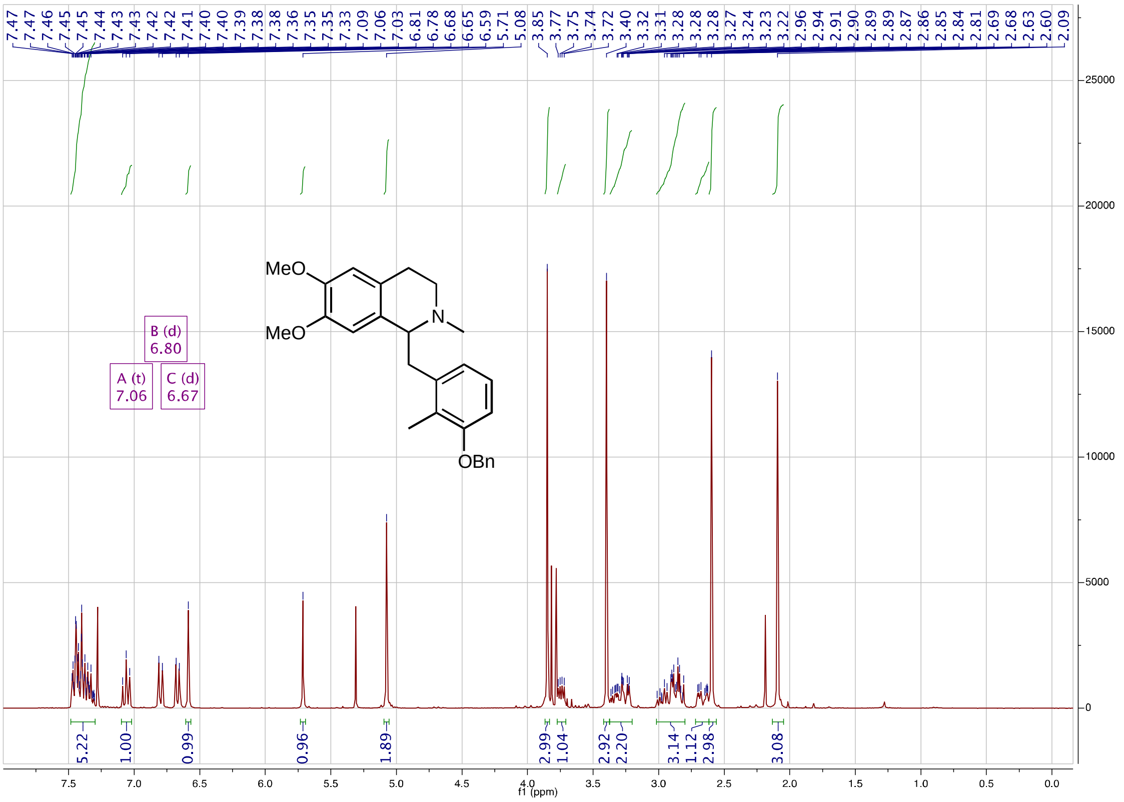

^{13}C-NMR

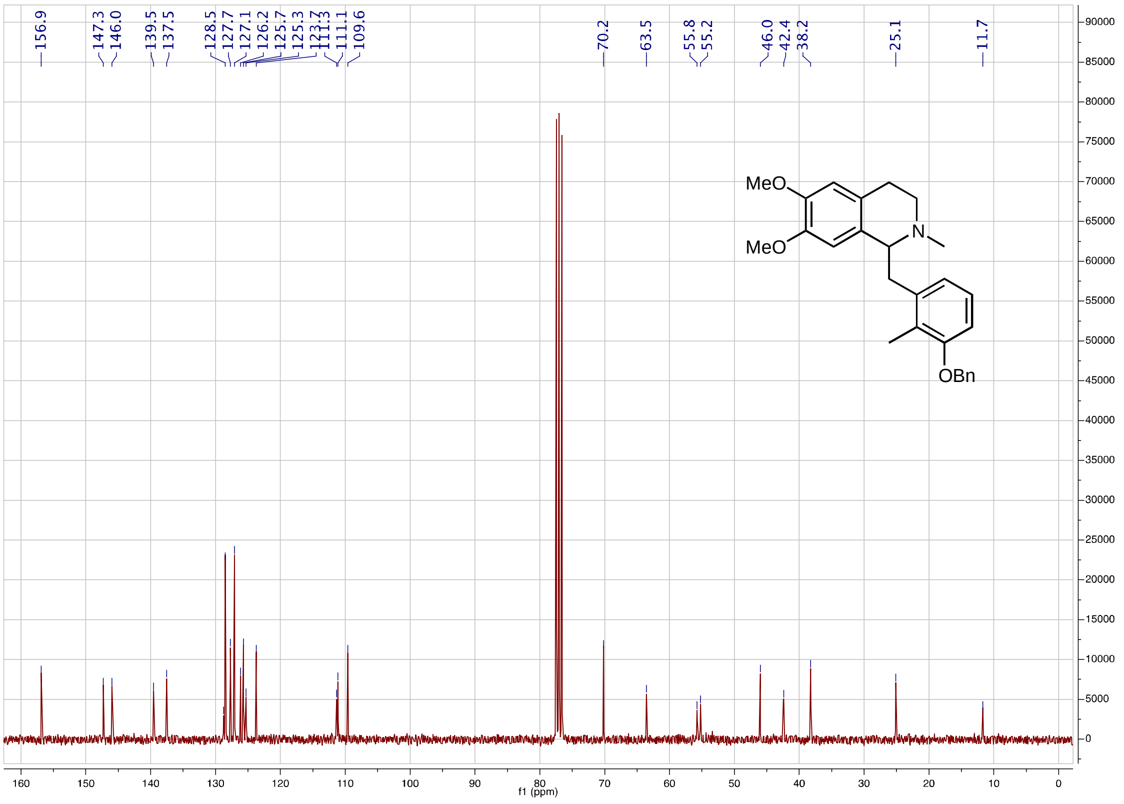

COSY spectrum

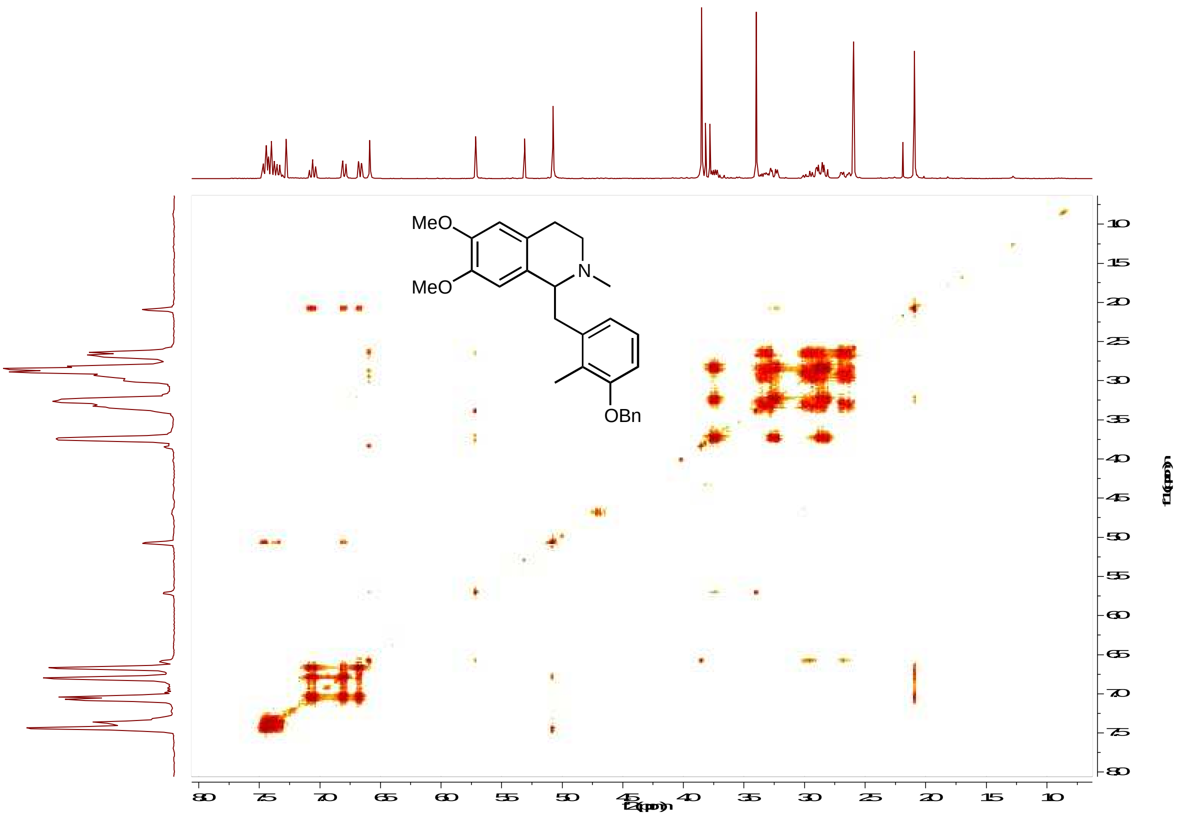

HSQC spectrum

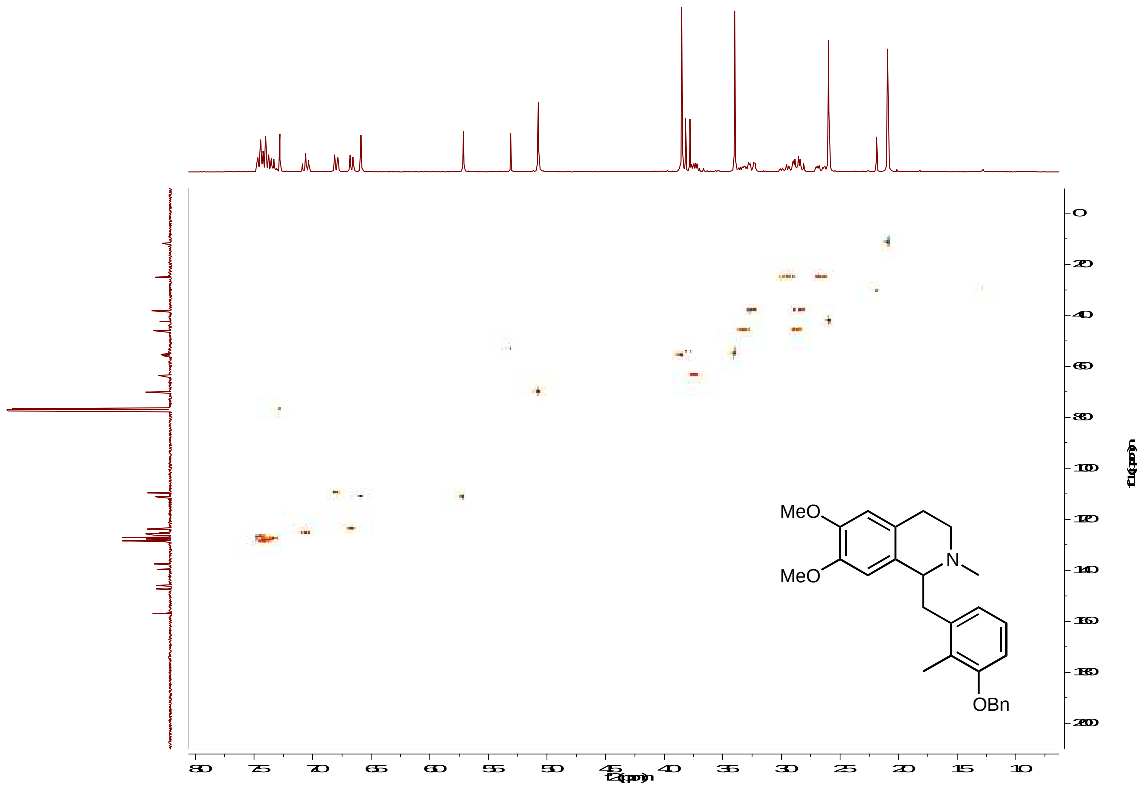

HRMS results

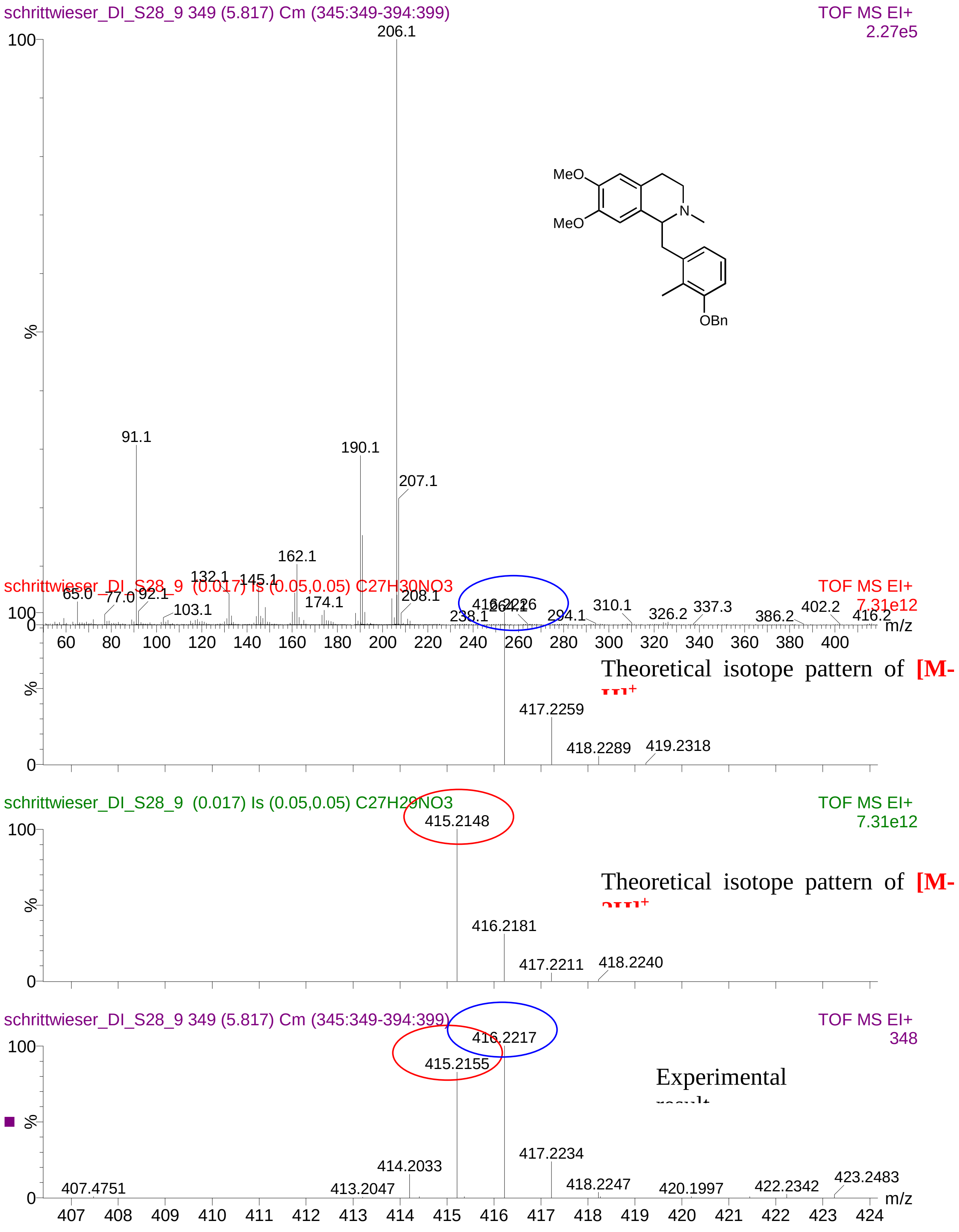

3-((6,7-Dimethoxy-2-methyl-1,2,3,4-tetrahydroisoquinolin-1-yl)methyl)-2-methylphenol

^{1}H-NMR

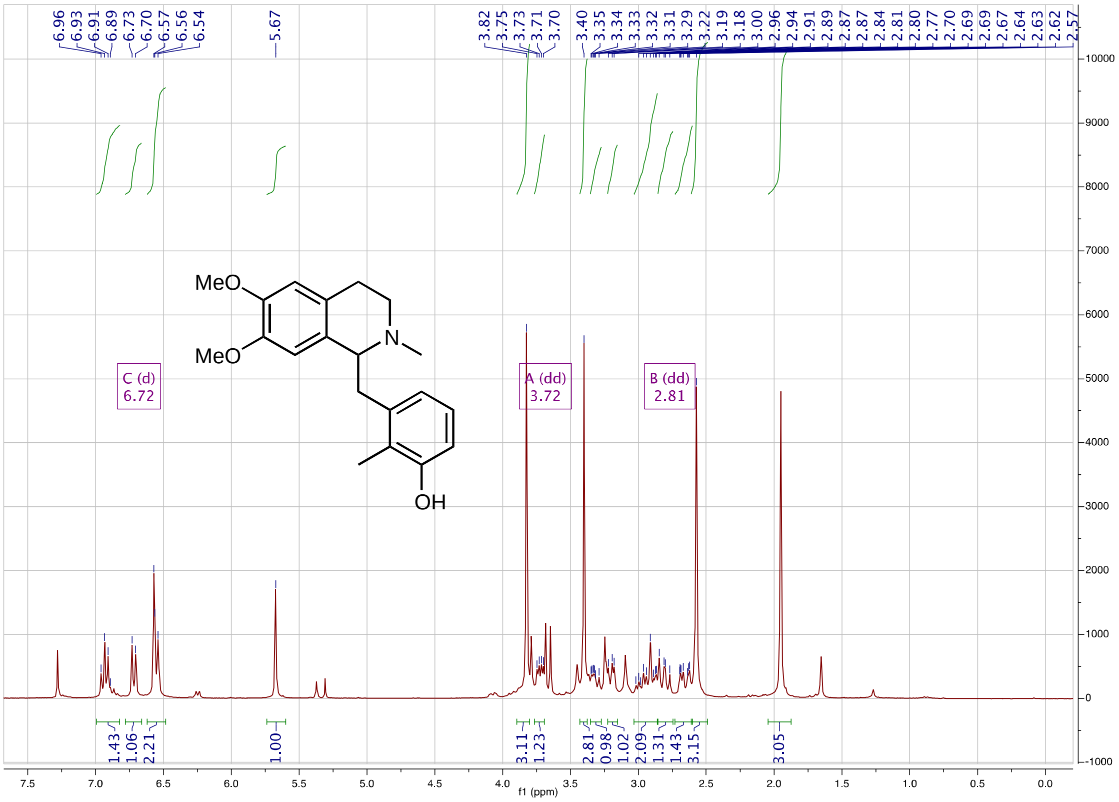

^{13}C-NMR

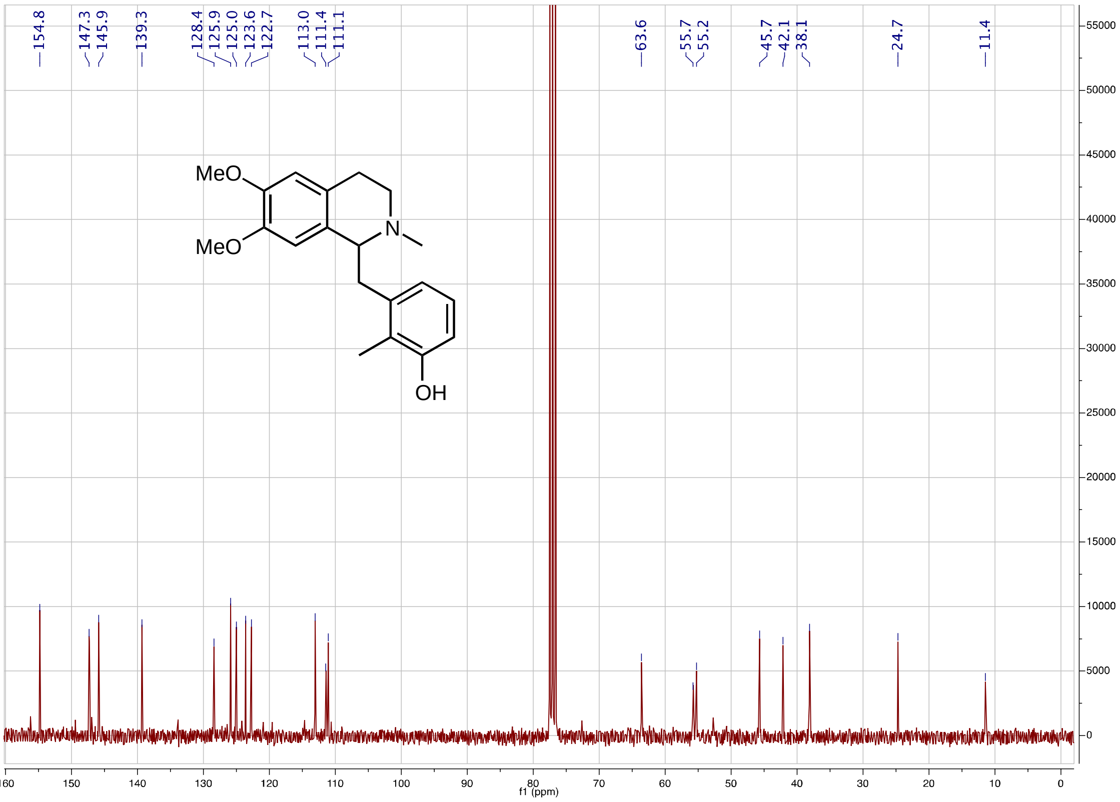

COSY spectrum

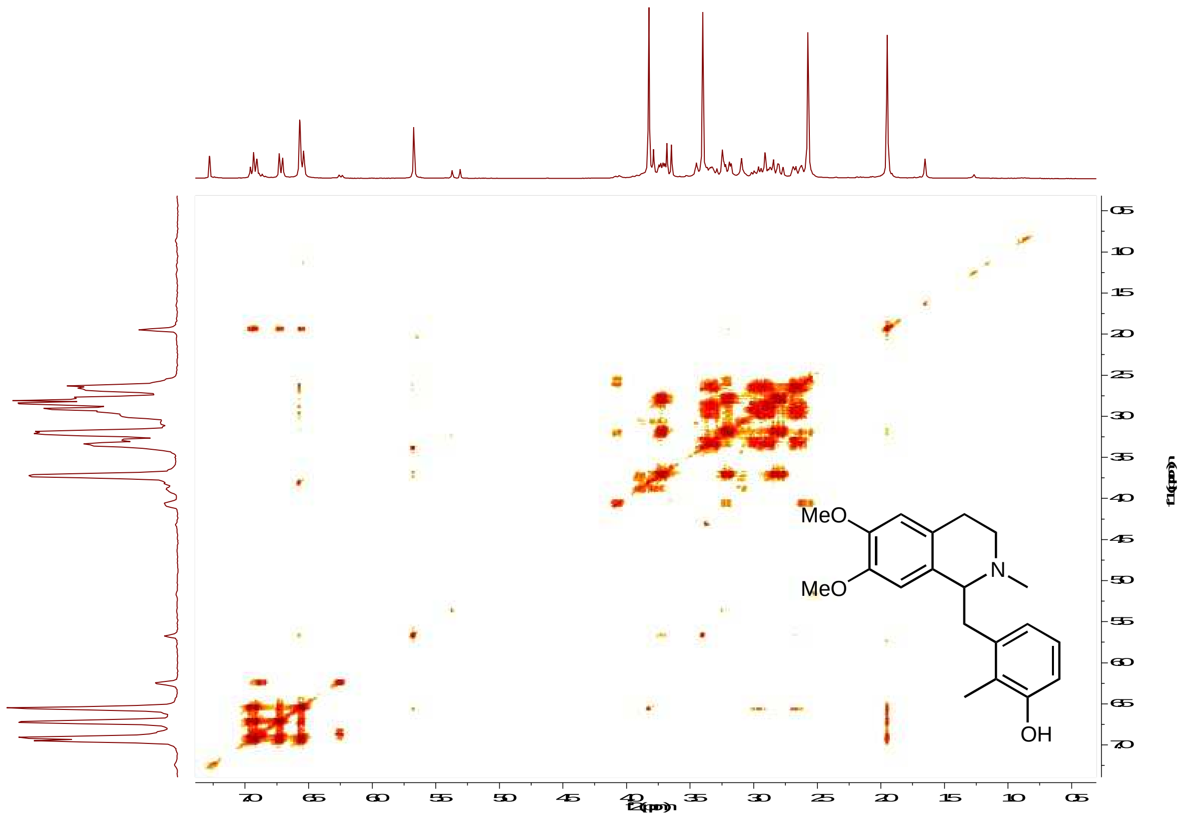

HSQC spectrum

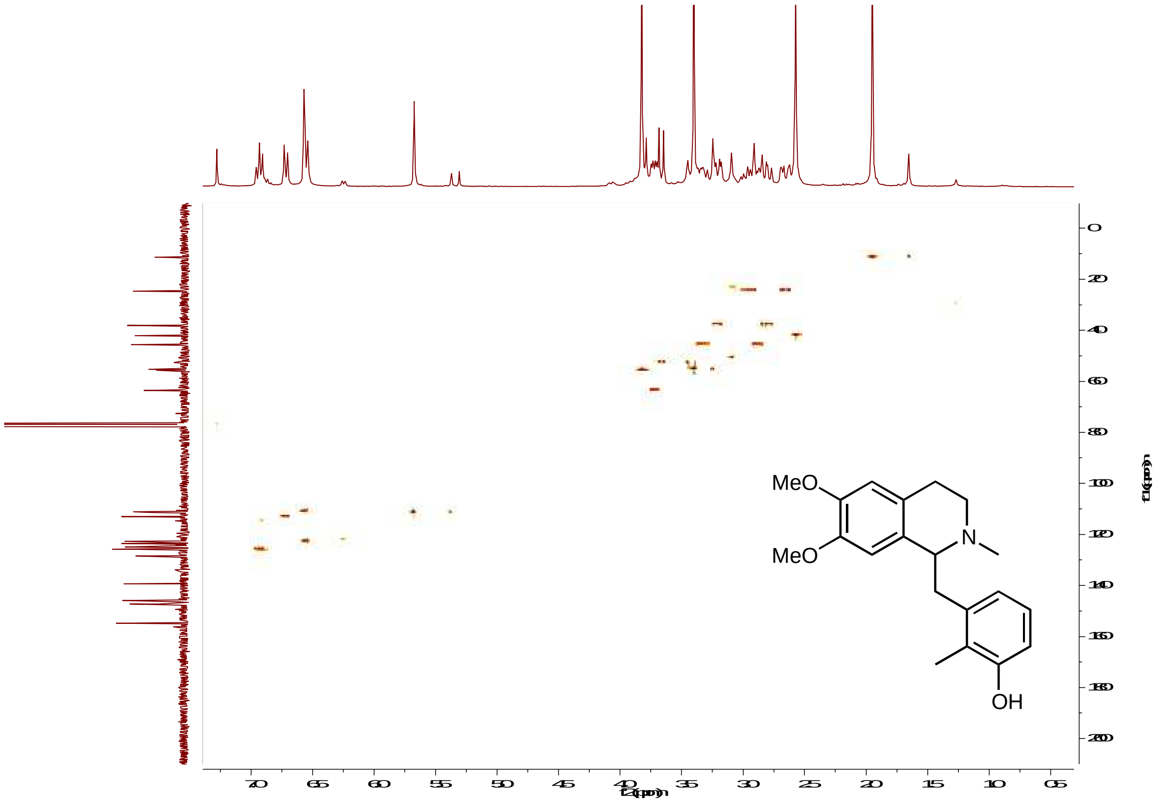

HRMS results

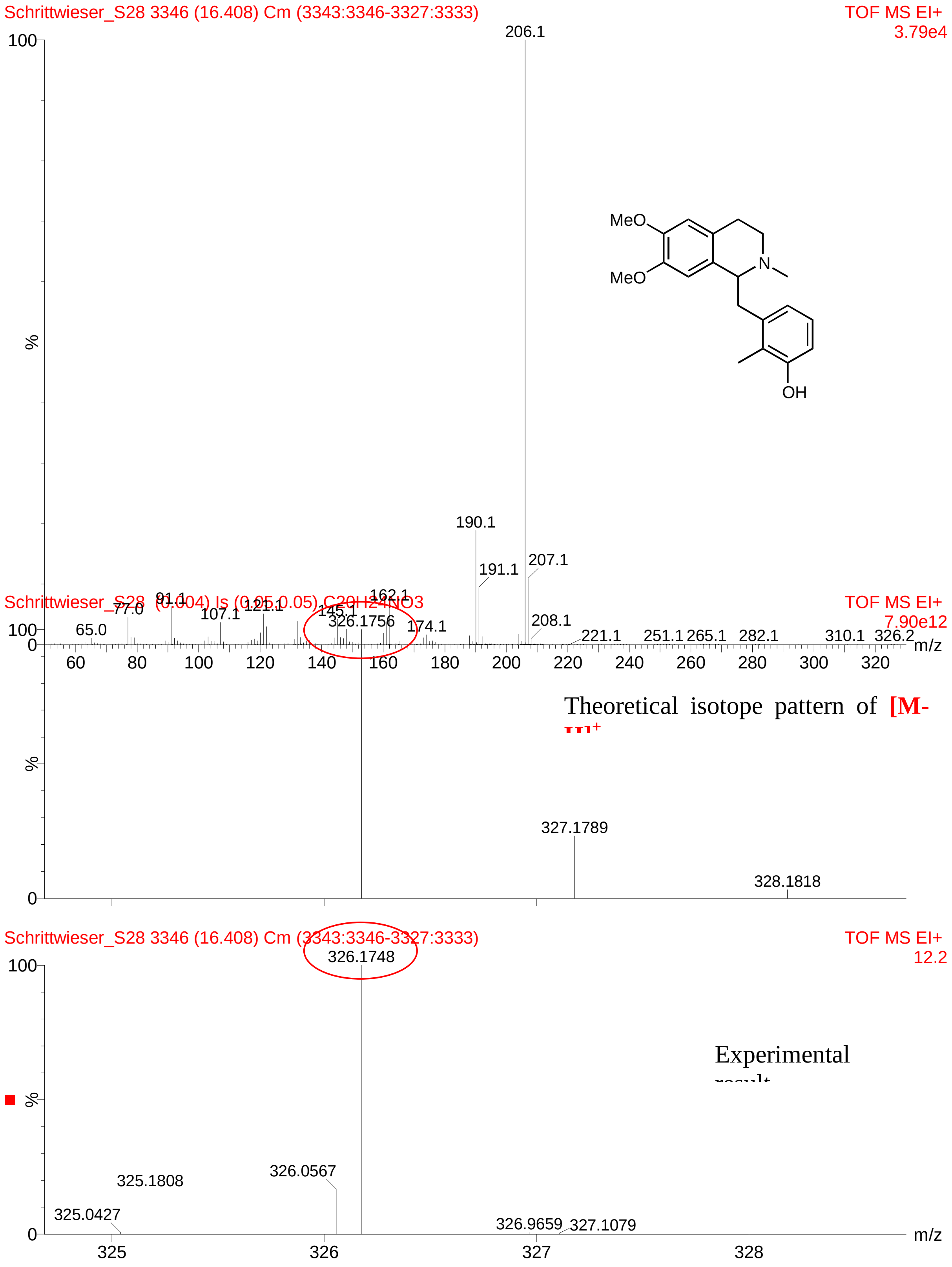

Synthesis of **1n**:

Provided Material:

2,2,2-Trichloro-1-(2-fluoro-3-methoxyphenyl)ethanol
^{1}H-NMR spectrum, ^{13}C-NMR spectrum

2-(2-Fluoro-3-methoxyphenyl)acetic acid
^{1}H-NMR spectrum, ^{13}C-NMR spectrum

2-(2-Fluoro-3-hydroxyphenyl)acetic acid
^{1}H-NMR spectrum, ^{13}C-NMR spectrum

2-(3-(Benzyloxy)-2-fluorophenyl)acetic acid
^{1}H-NMR spectrum, ^{13}C-NMR spectrum, HRMS results

2-(3-(Benzyloxy)-2-fluorophenyl)acetyl chloride
^{1}H-NMR spectrum, ^{13}C-NMR spectrum

2-(3-(Benzyloxy)-2-fluorophenyl)-*N*-(3,4-dimethoxyphenethyl)-*N*-methylacetamide
^{1}H-NMR spectrum, ^{13}C-NMR spectrum, ^{13}C-NMR DEPT135 spectrum, ^{13}C-NMR DEPT90 spectrum, COSY spectrum, HSQC spectrum, HRMS results

1-(3-(Benzyloxy)-2-fluorobenzyl)-6,7-dimethoxy-2-methyl-1,2,3,4-tetrahydroisoquinoline
^{1}H-NMR spectrum, ^{13}C-NMR spectrum, COSY spectrum, HSQC spectrum, HRMS results

3-((6,7-Dimethoxy-2-methyl-1,2,3,4-tetrahydroisoquinolin-1-yl)methyl)-2-fluorophenol
^{1}H-NMR spectrum, ^{13}C-NMR spectrum, COSY spectrum, HSQC spectrum, HRMS results

2,2,2-Trichloro-1-(2-fluoro-3-methoxyphenyl)ethanol

^{1}H-NMR

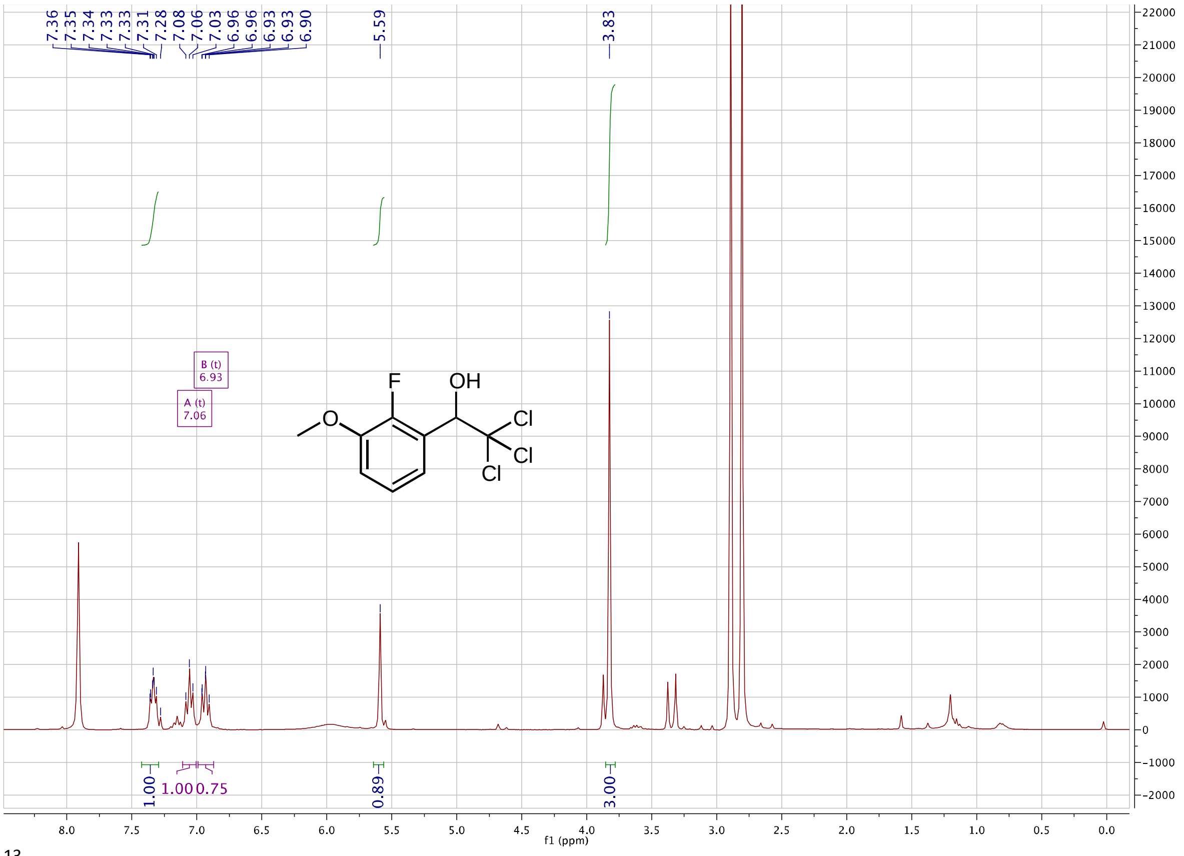

^{13}C-NMR

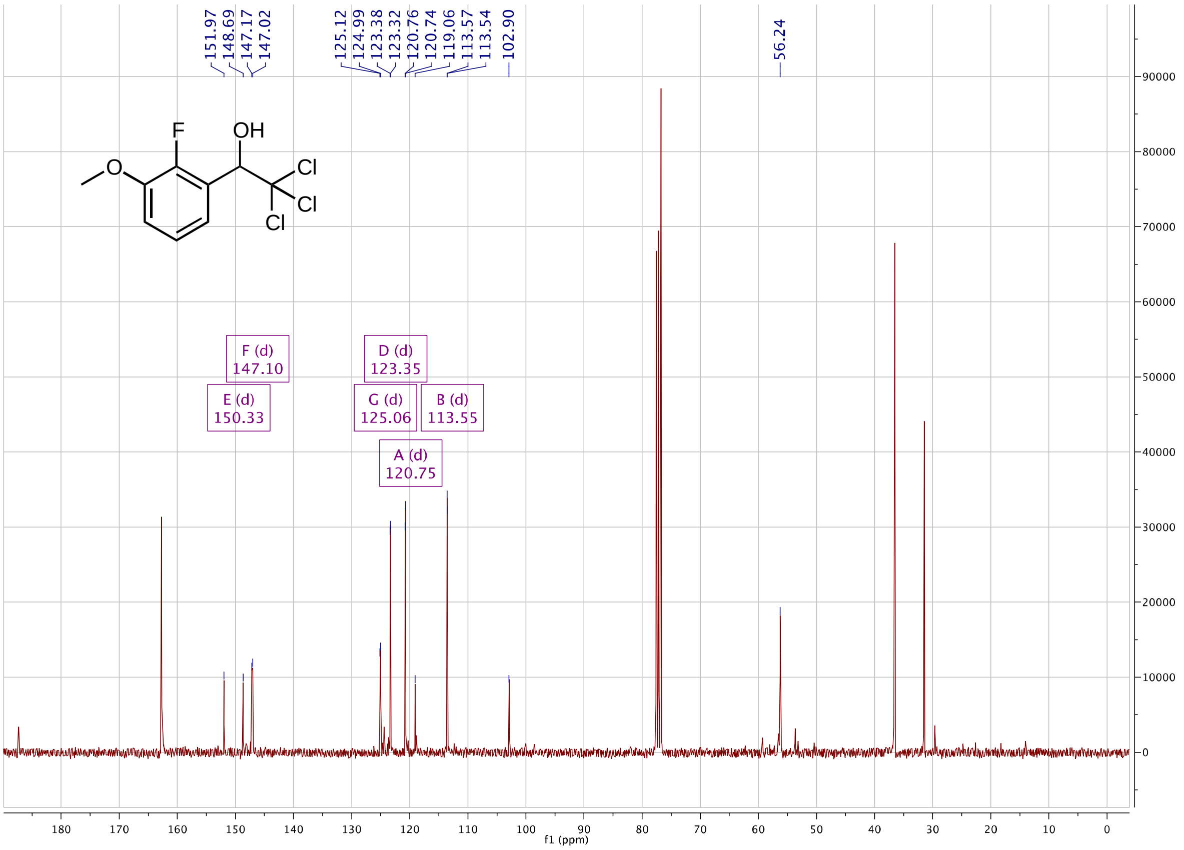

2-(2-Fluoro-3-methoxyphenyl)acetic acid

^{1}H-NMR

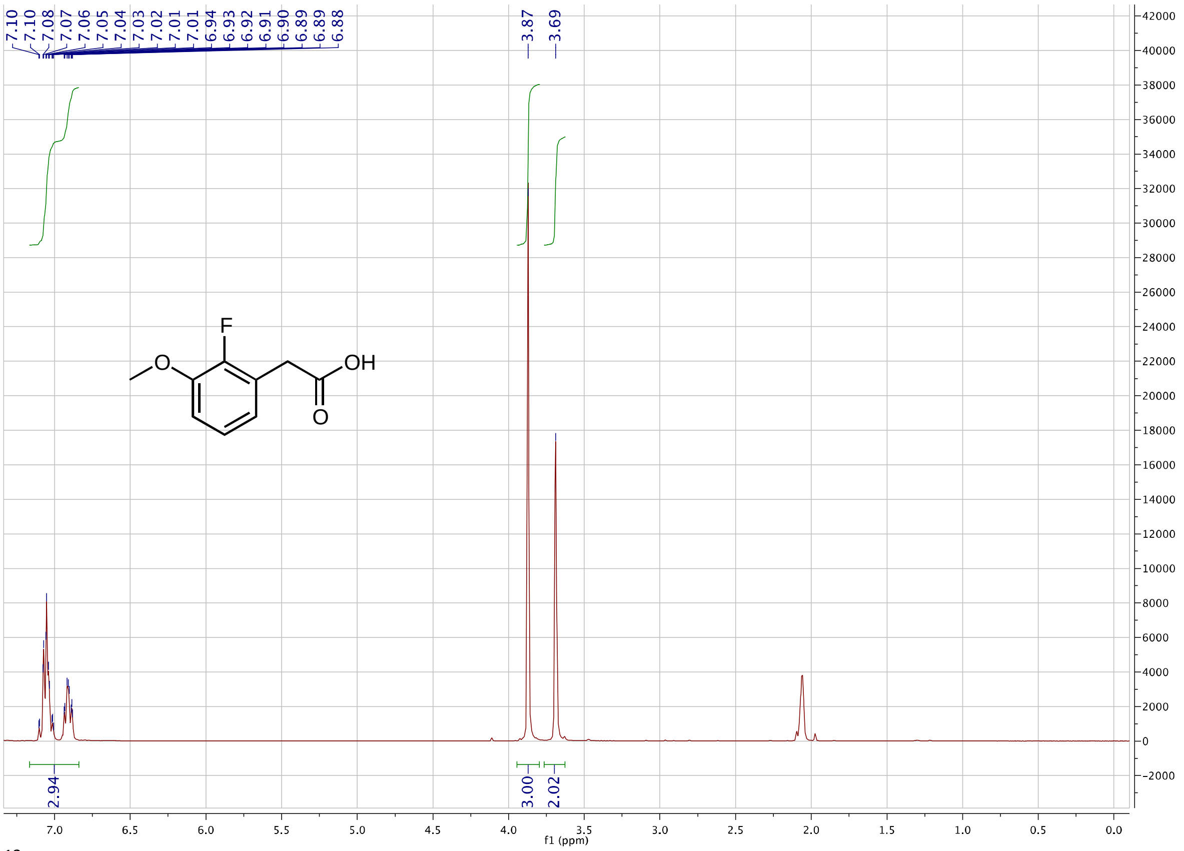

^{13}C-NMR

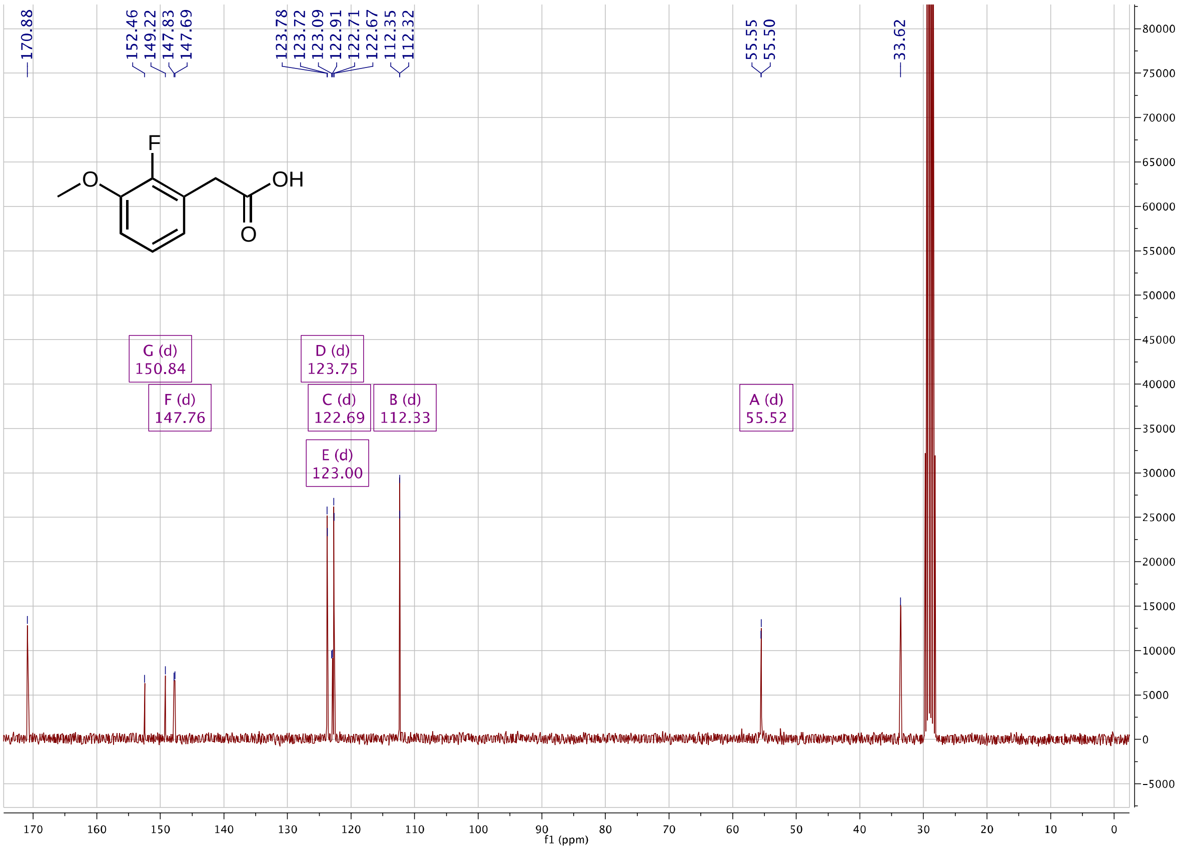

2-(2-Fluoro-3-hydroxyphenyl)acetic acid

^{1}H-NMR

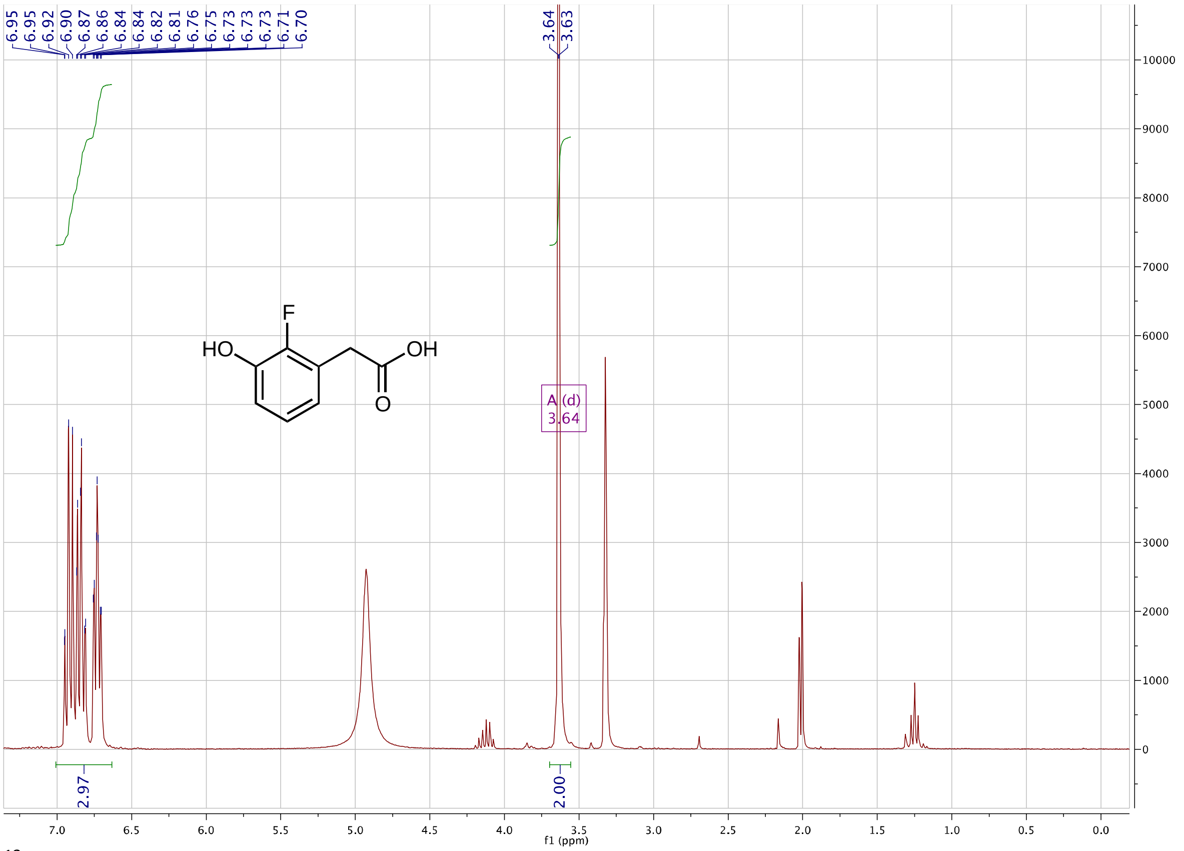

^{13}C-NMR

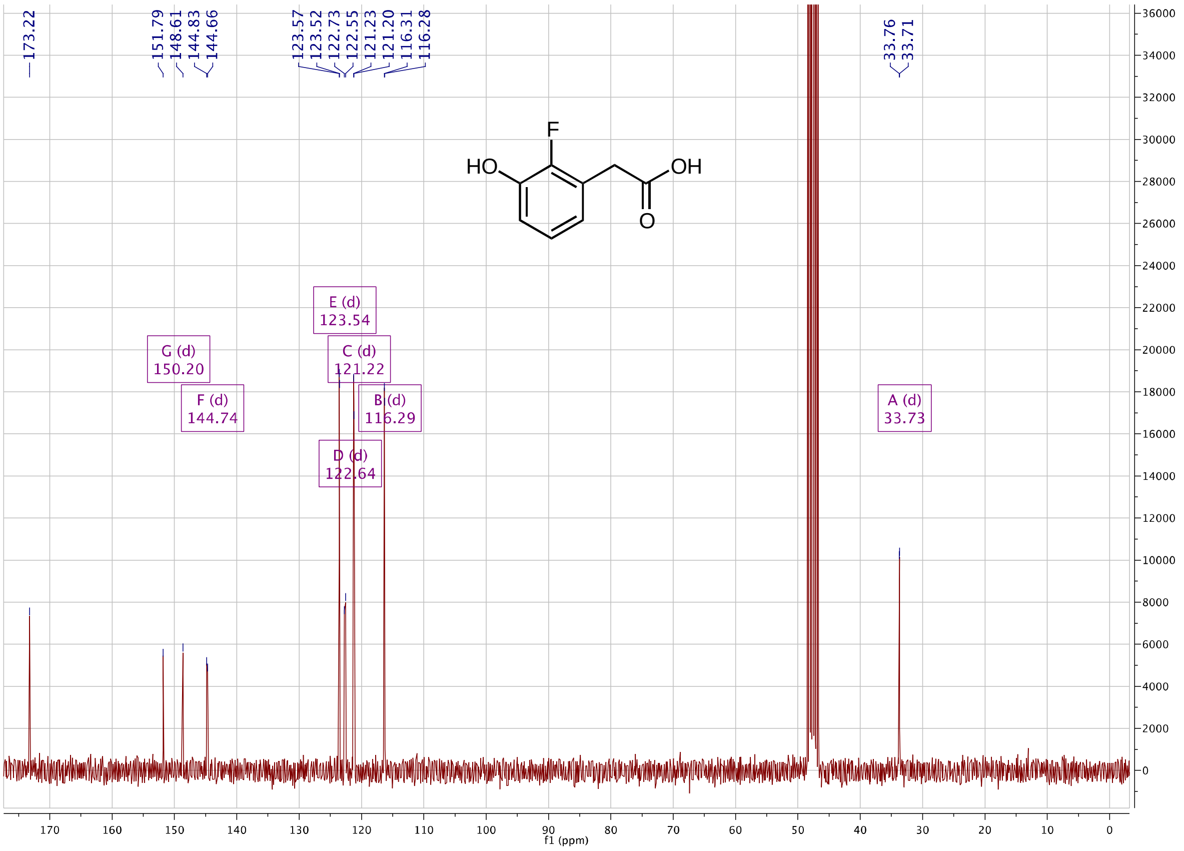

2-(3-(Benzyloxy)-2-fluorophenyl)acetic acid

^{1}H-NMR

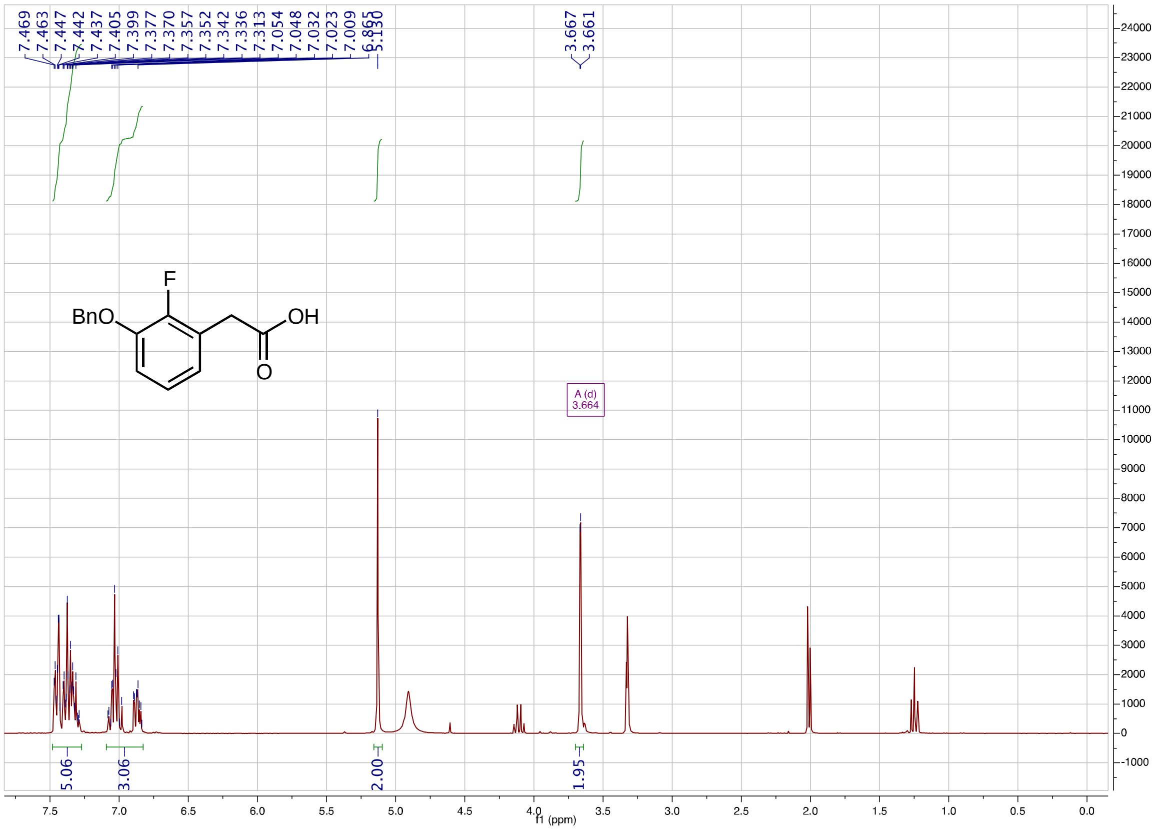

^{13}C-NMR

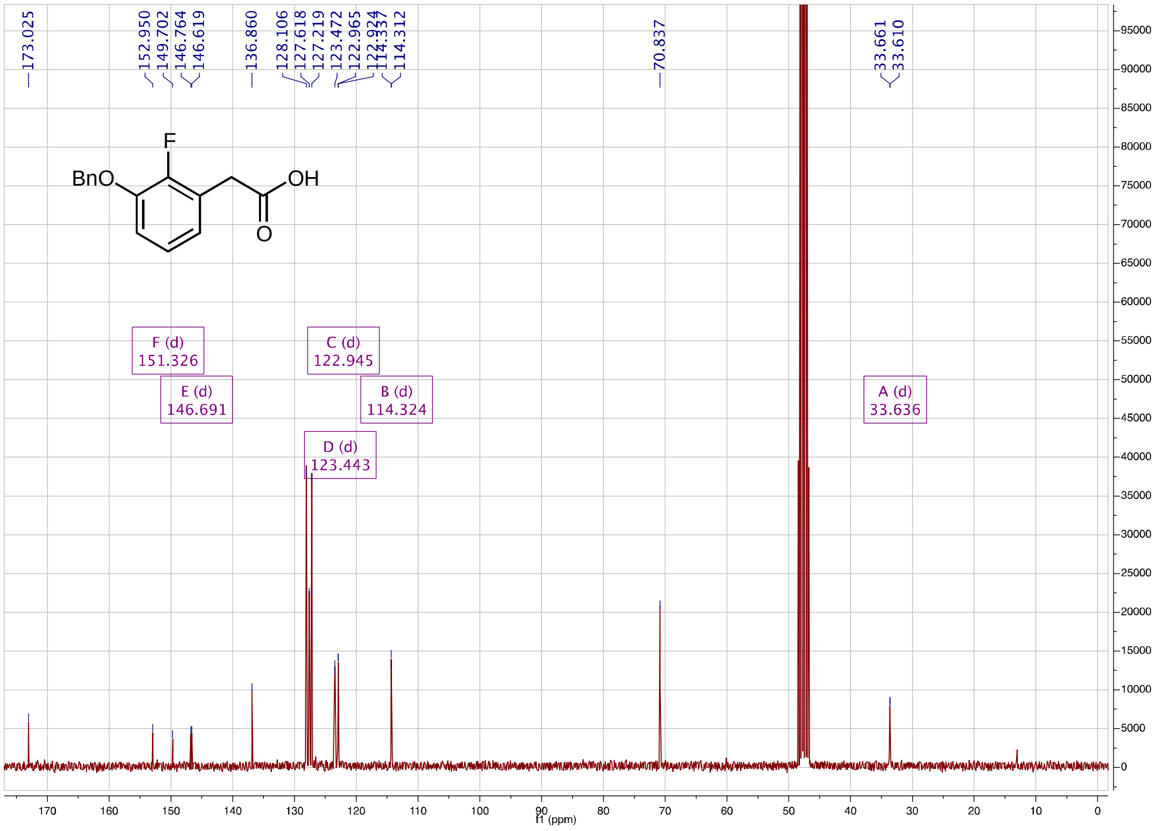

HRMS results

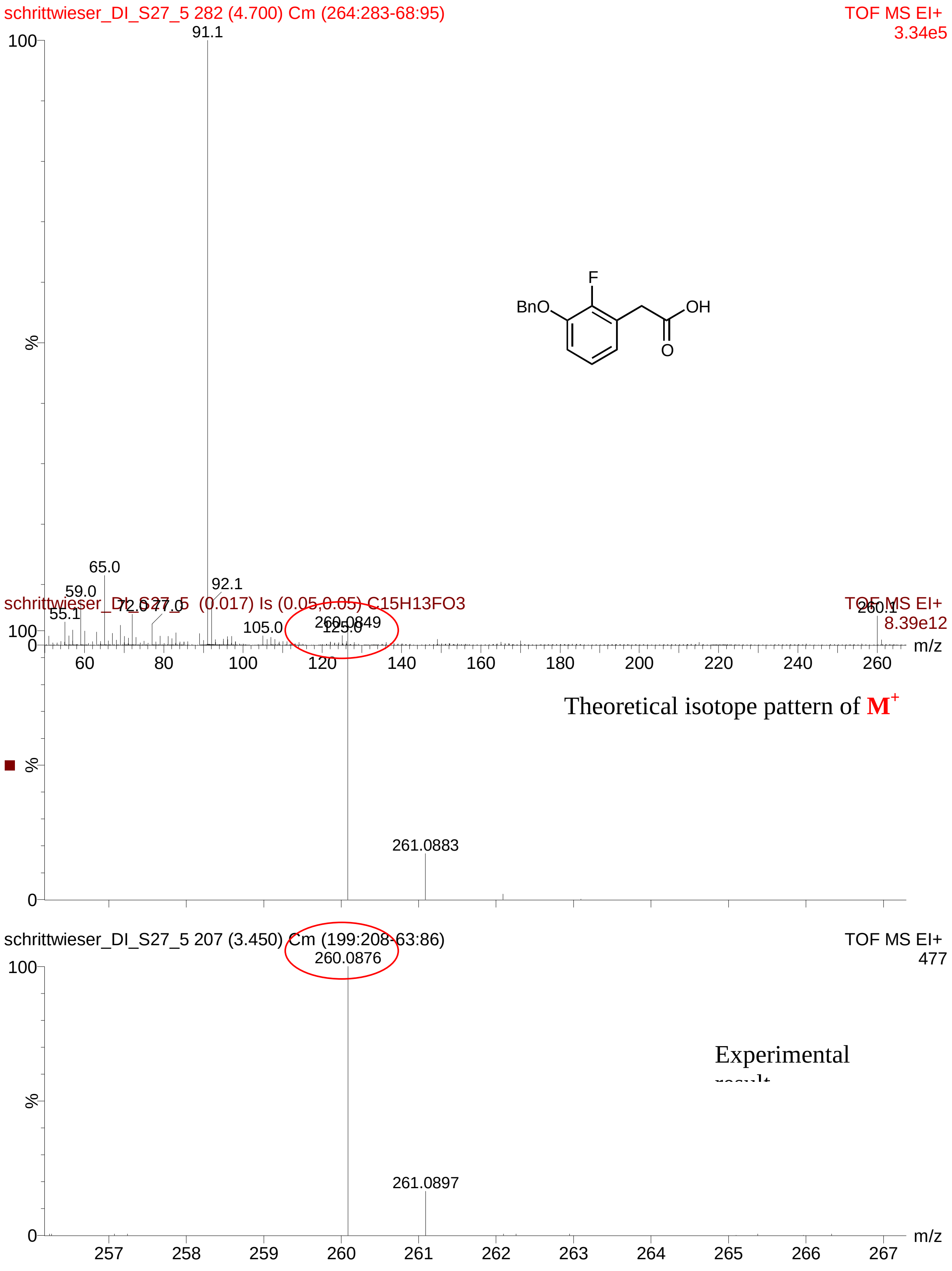

2-(3-(Benzyloxy)-2-fluorophenyl)-*N*-(3,4-dimethoxyphenethyl)-*N*-methylacetamide

^{1}H-NMR

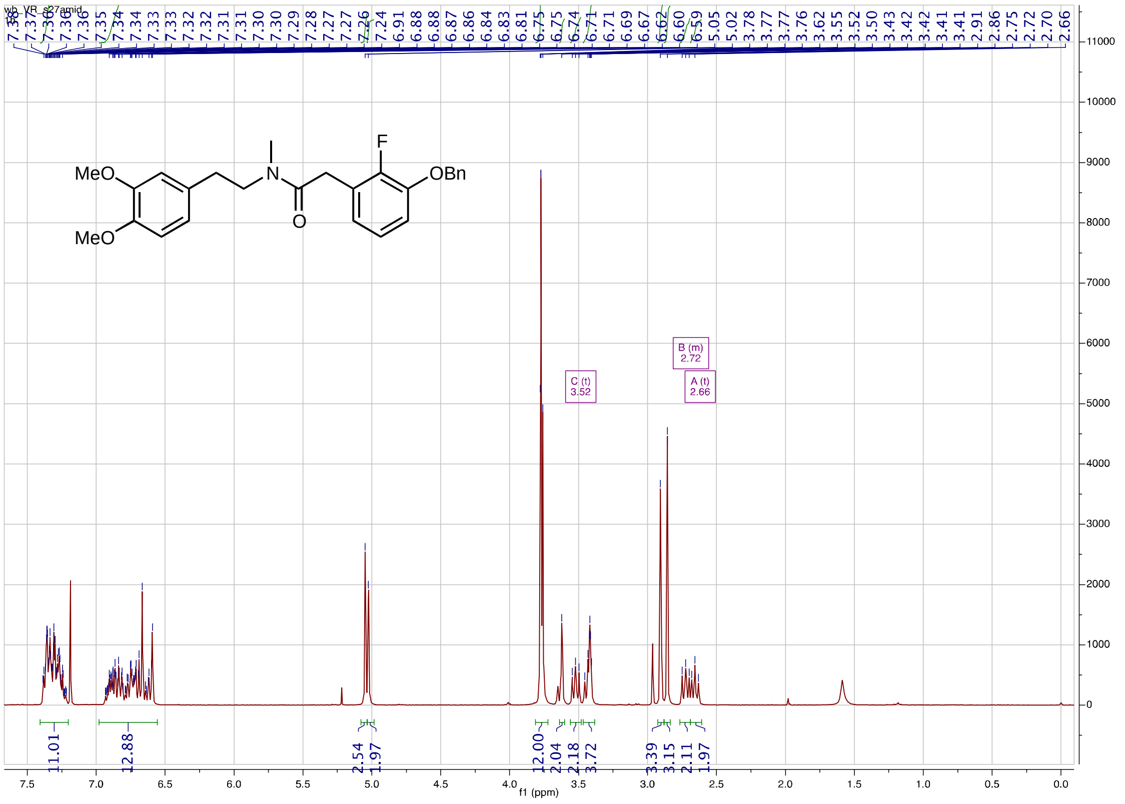

^{13}C-NMR

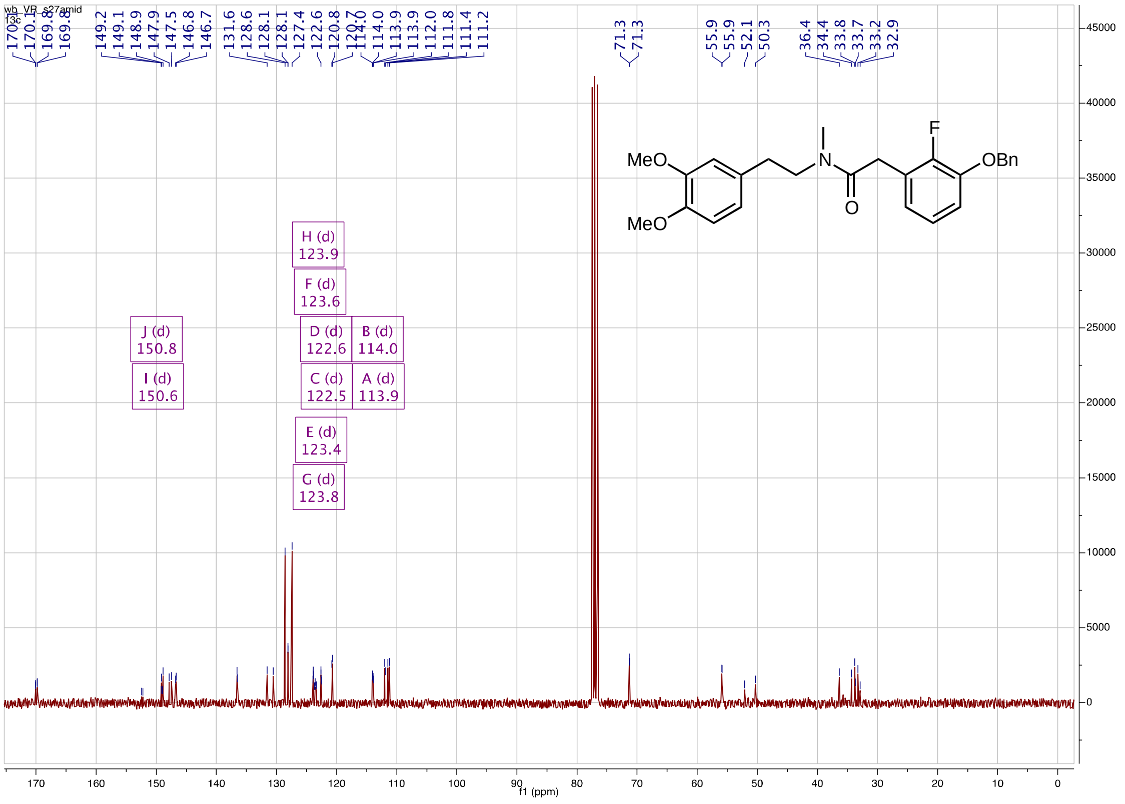

COSY spectrum

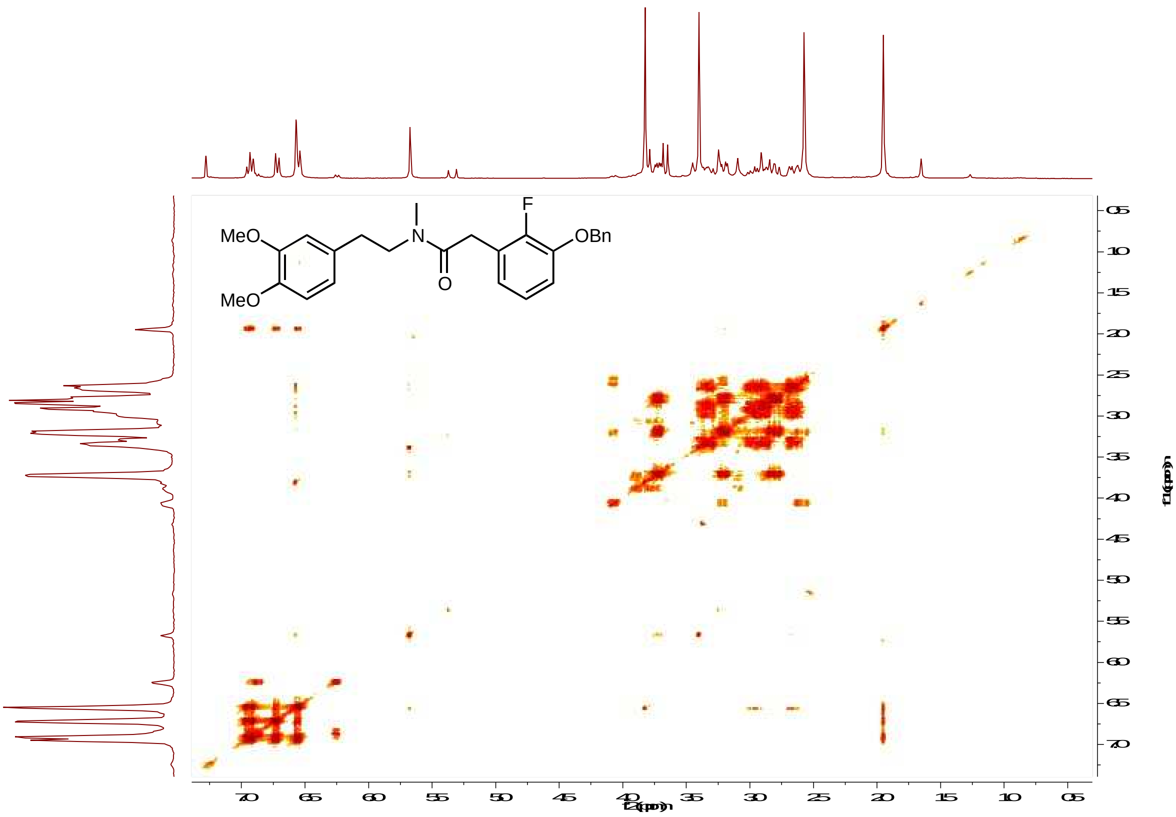

HSQC spectrum

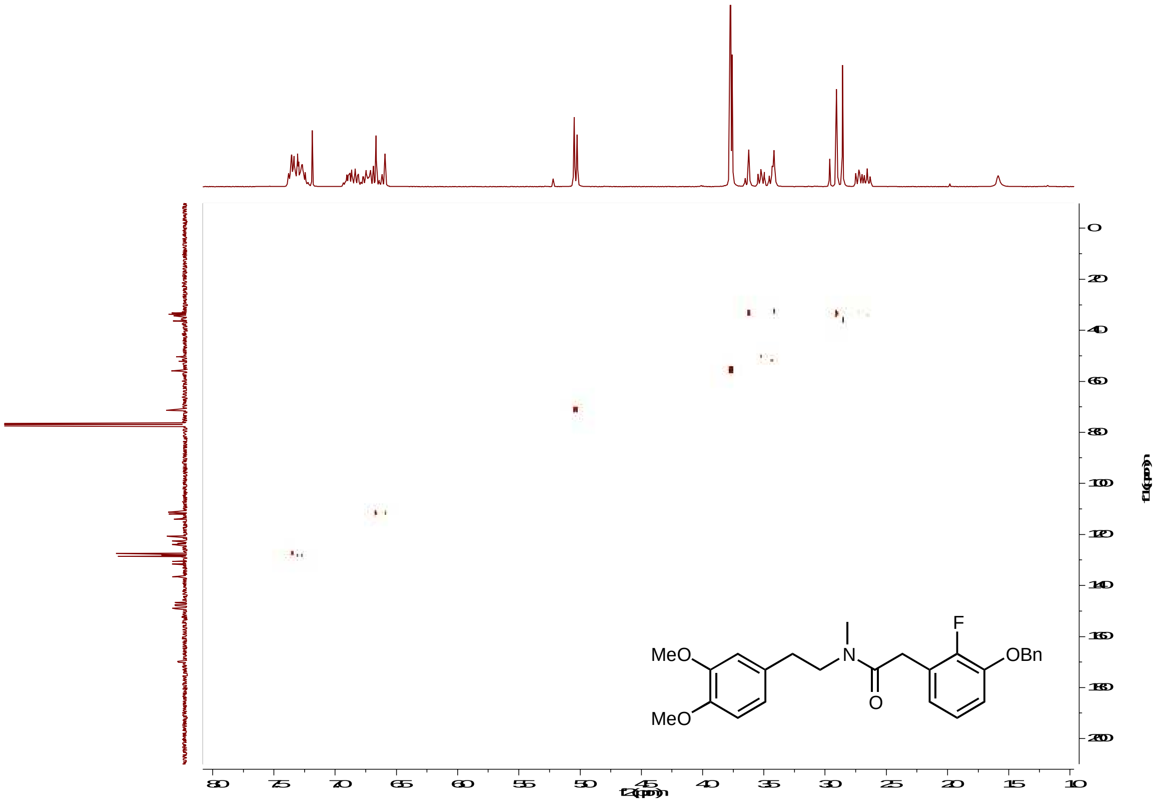

HRMS results

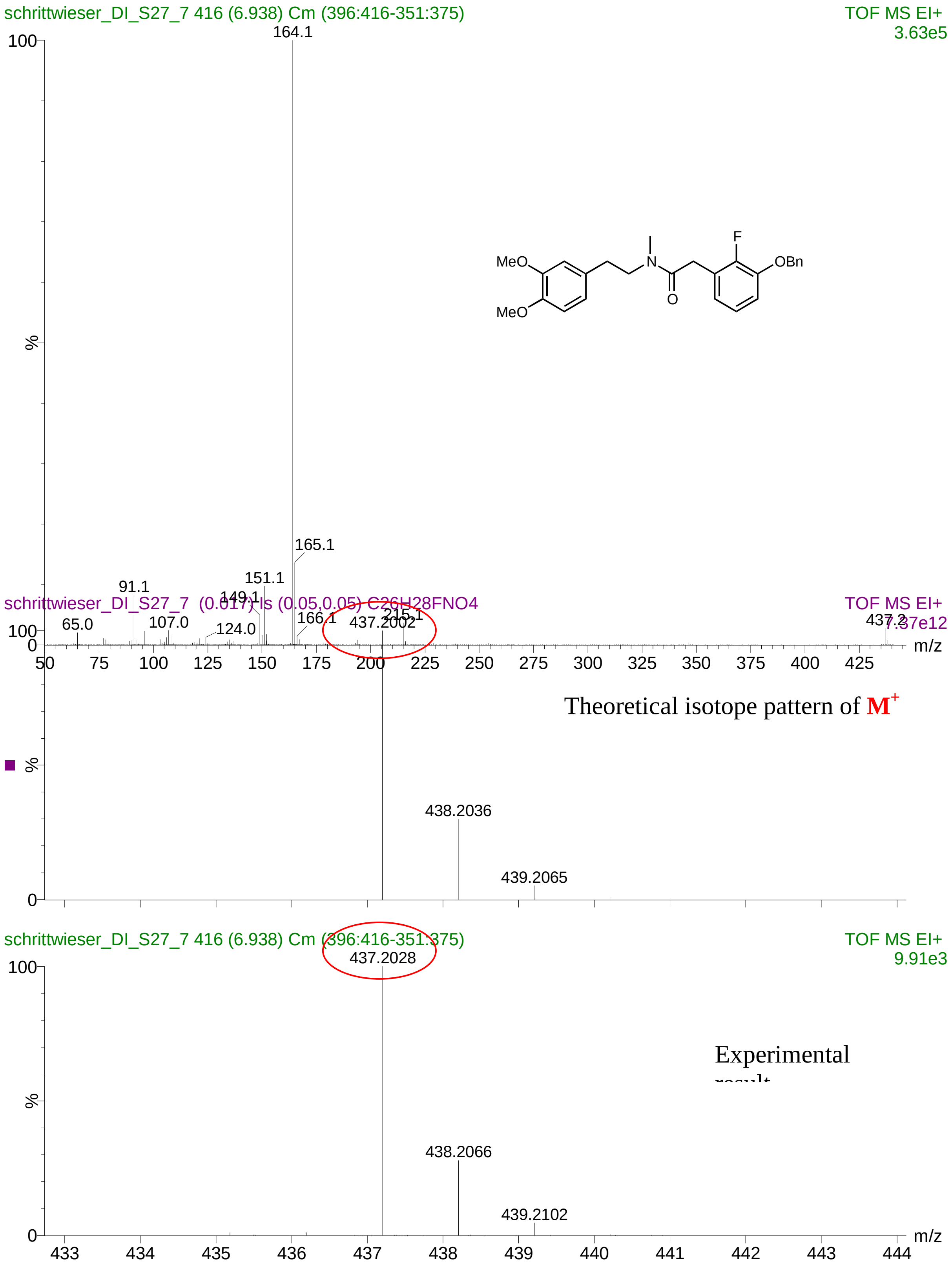

1-(3-(Benzyloxy)-2-fluorobenzyl)-6,7-dimethoxy-2-methyl-1,2,3,4-tetrahydroisoquinoline

^{1}H-NMR

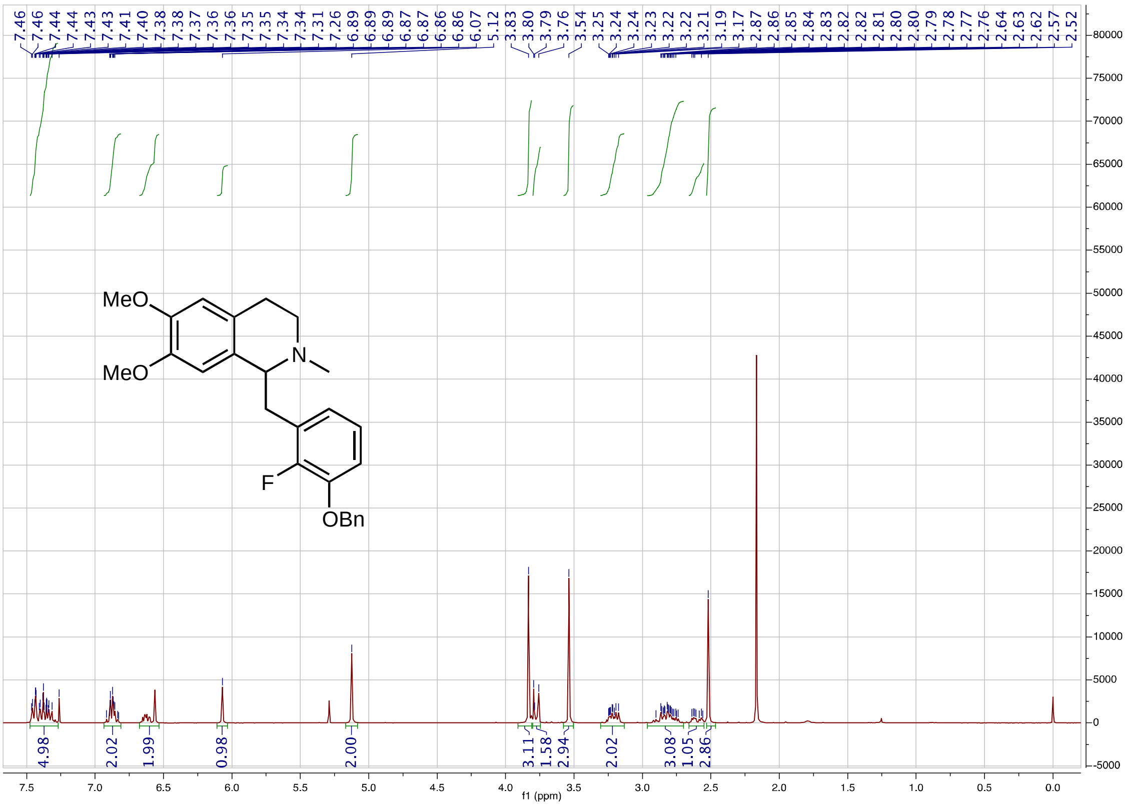

^{13}C-NMR

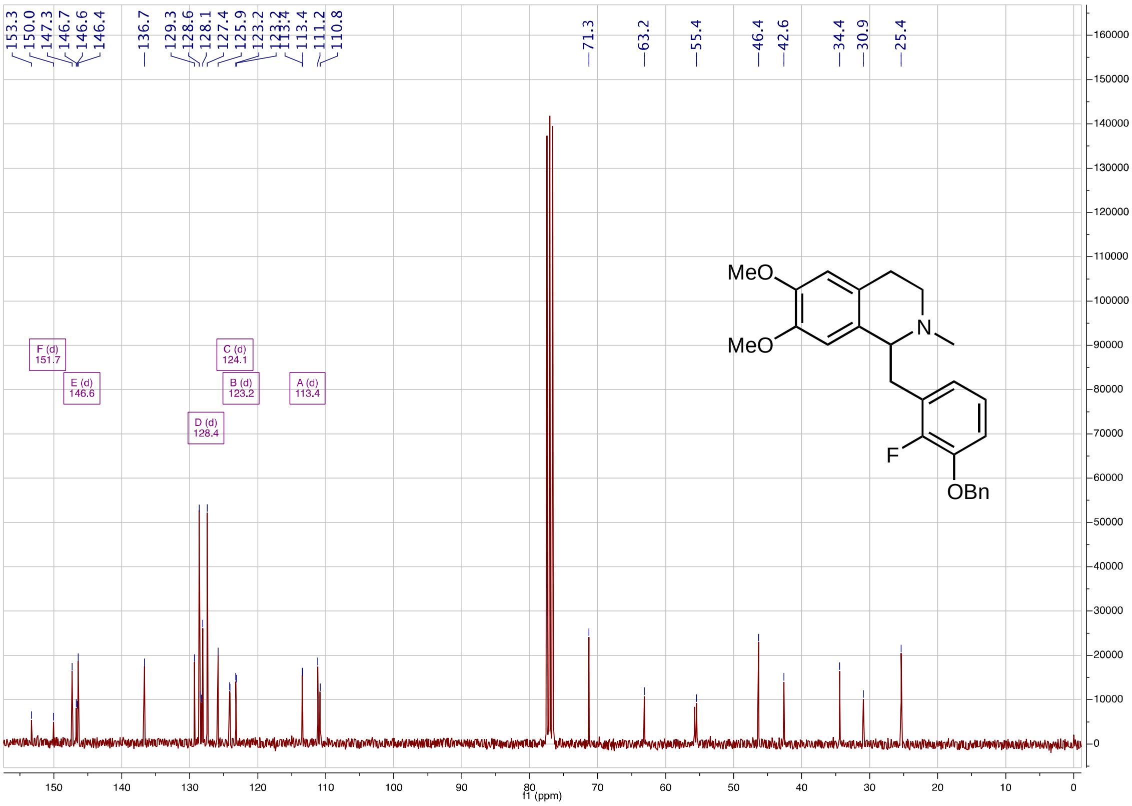

COSY spectrum

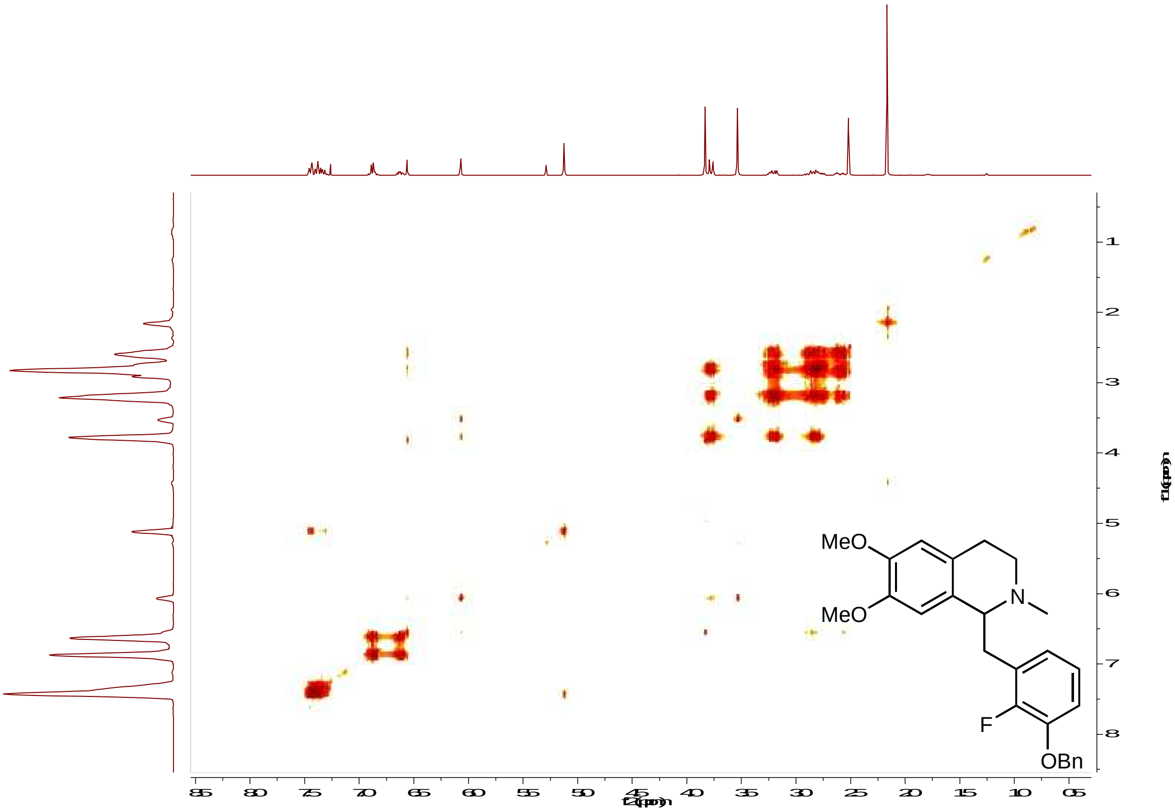

HSQC spectrum

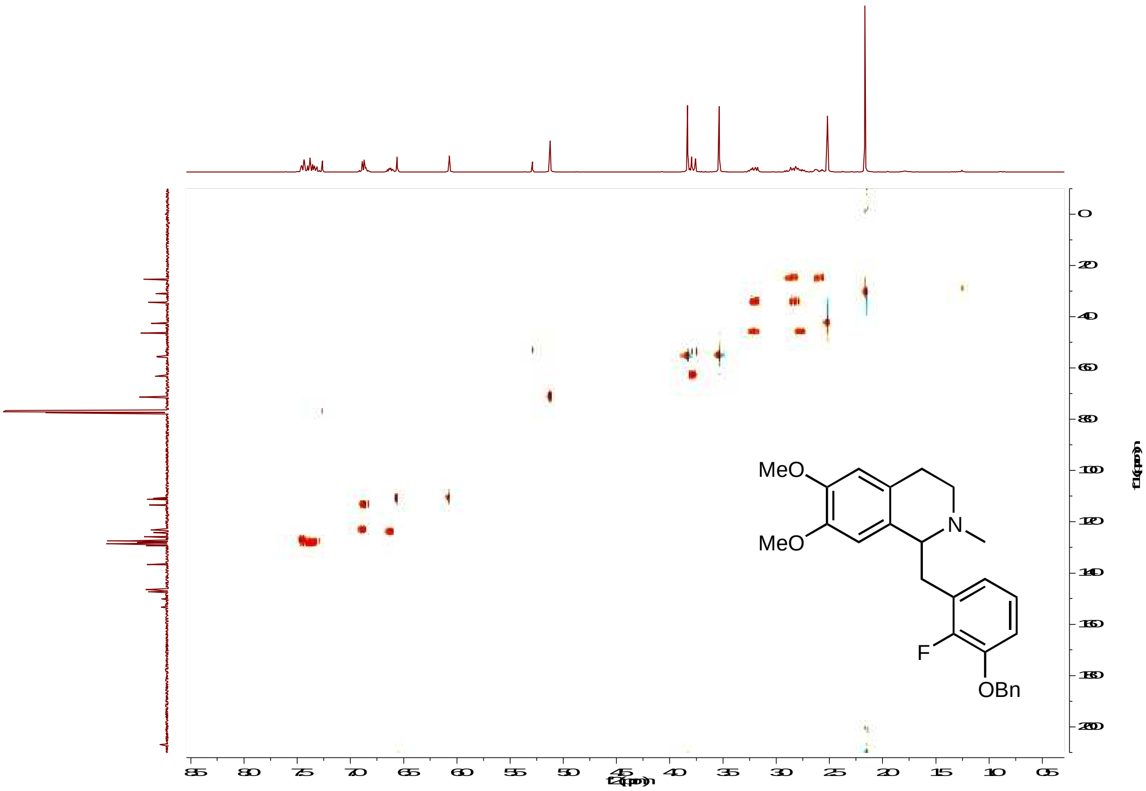

HRMS results

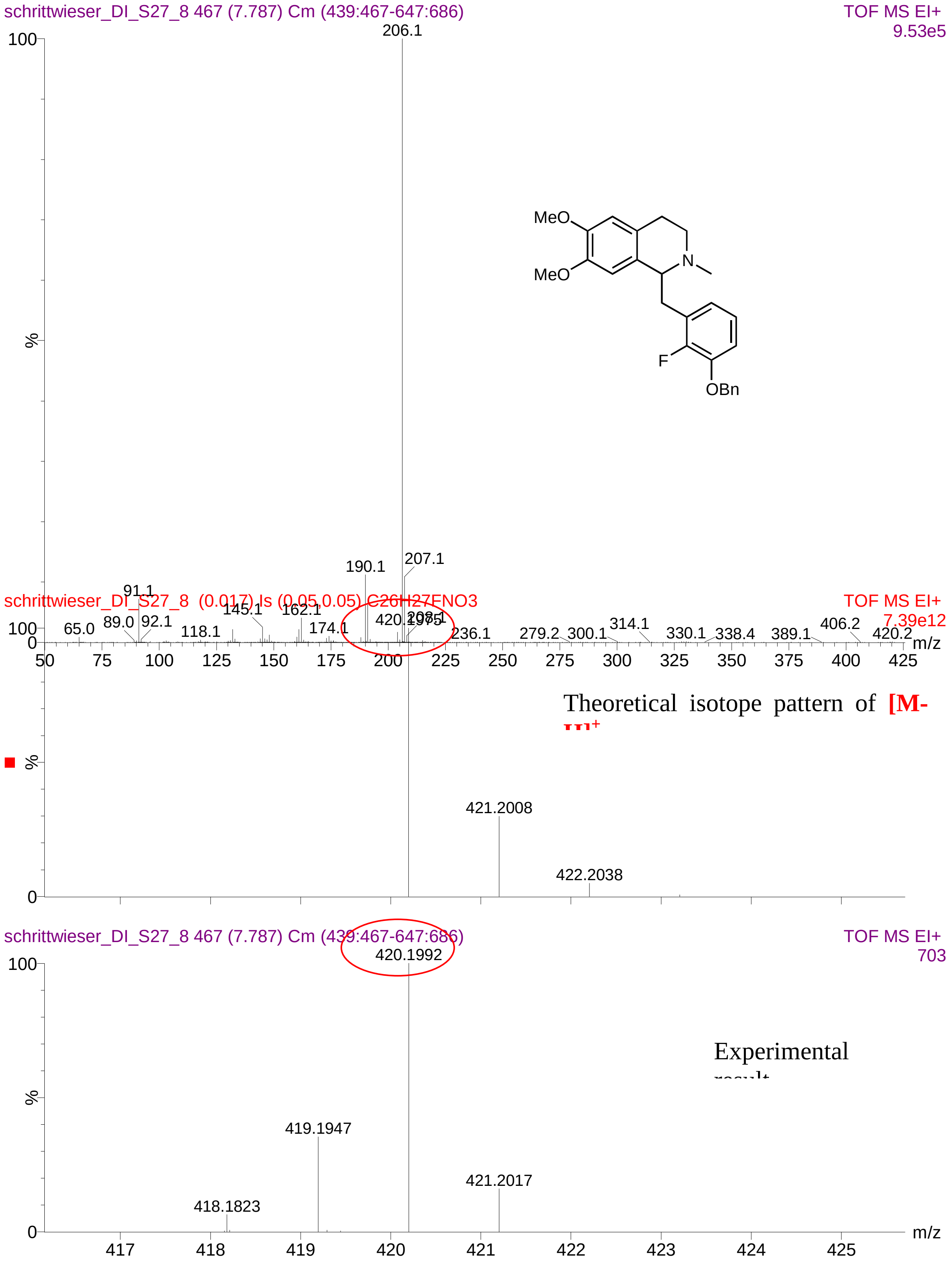

3-((6,7-Dimethoxy-2-methyl-1,2,3,4-tetrahydroisoquinolin-1-yl)methyl)-2-fluorophenol

^{1}H-NMR

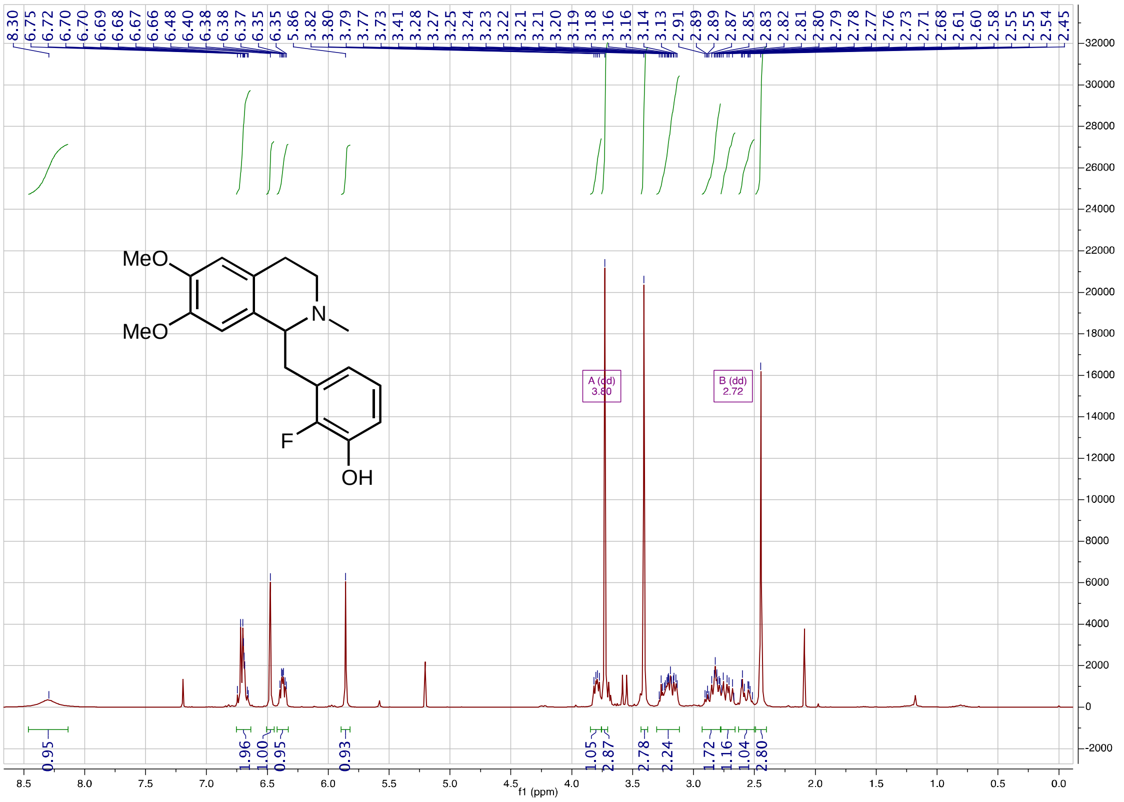

^{13}C-NMR

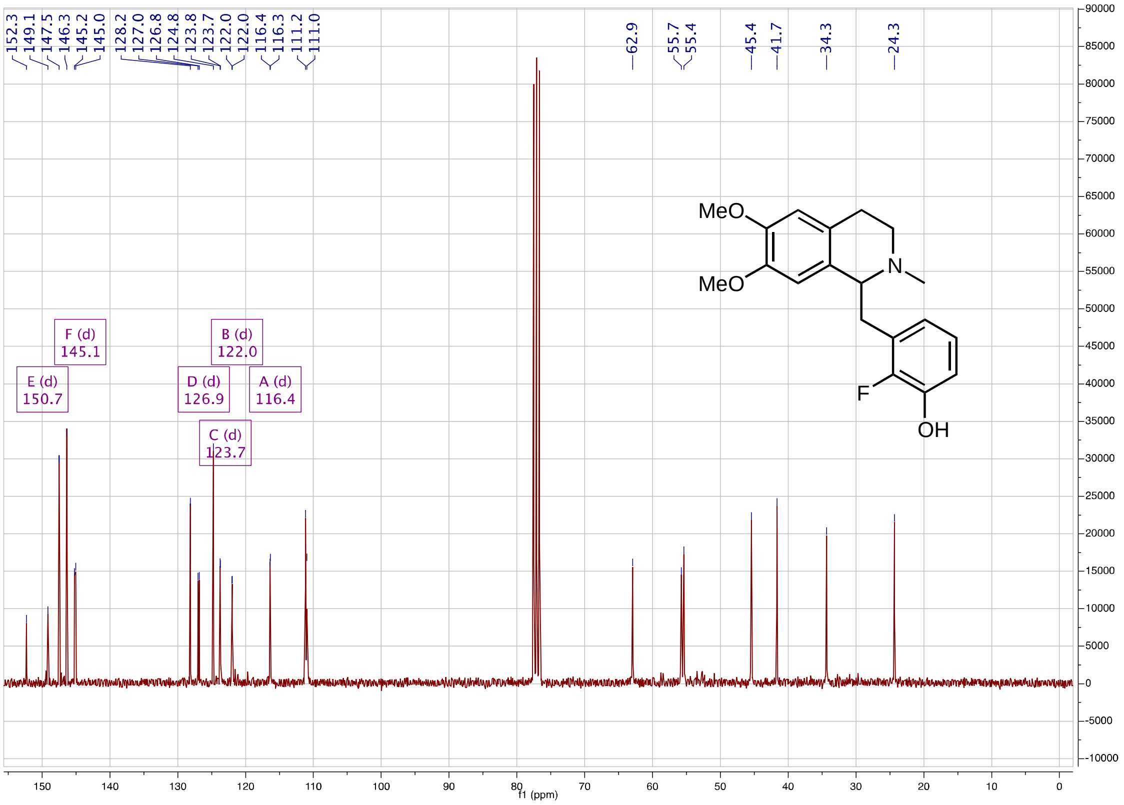

COSY spectrum

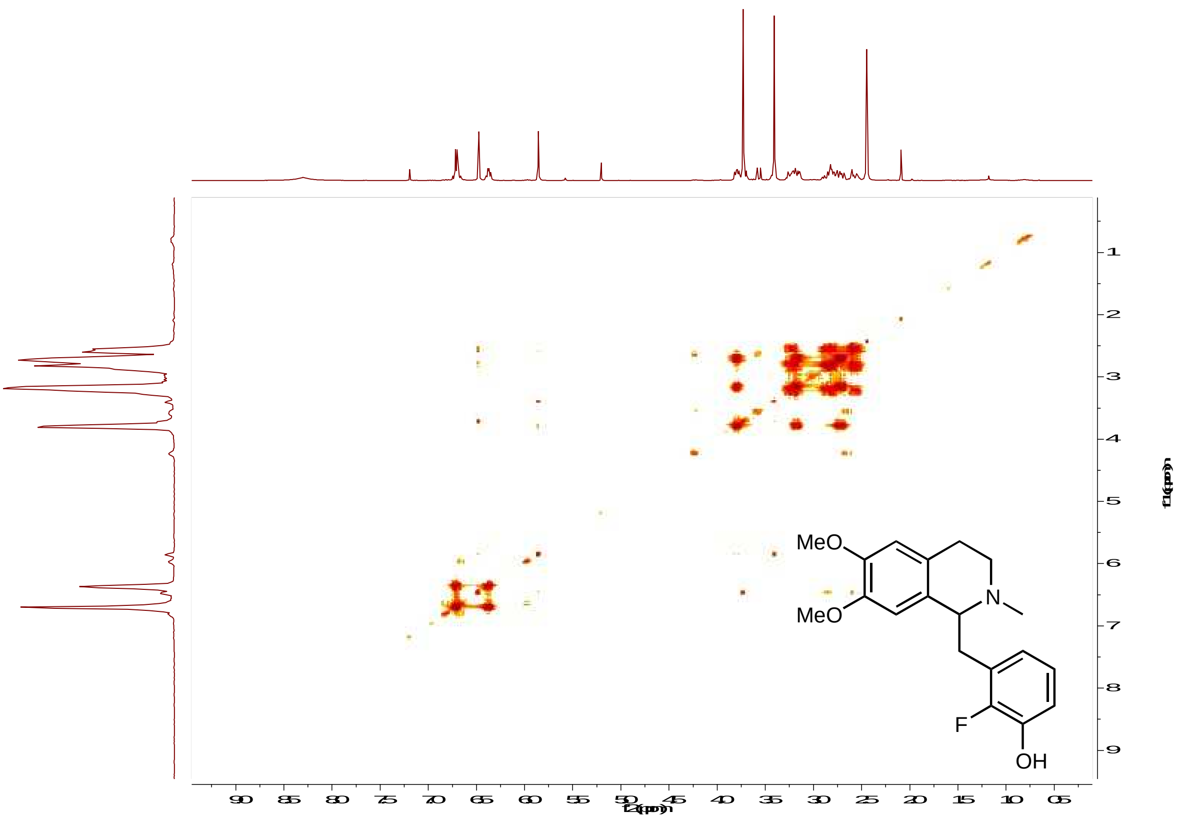

HSQC spectrum

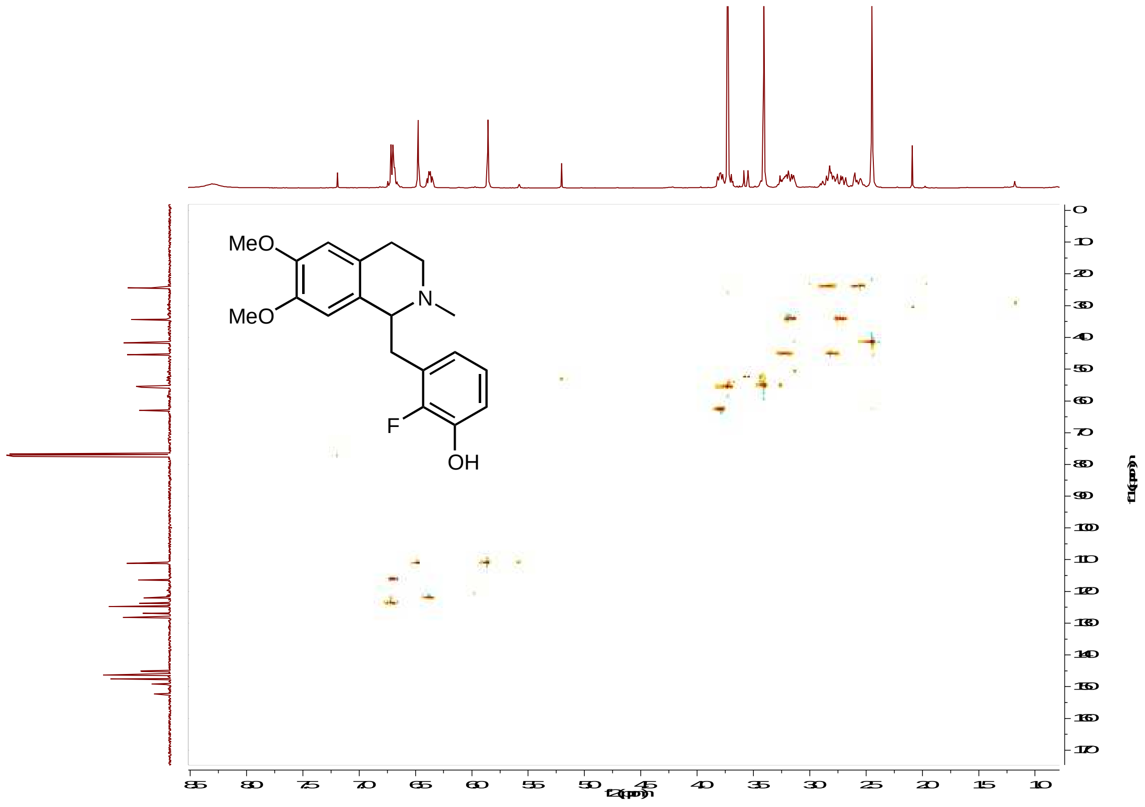

HRMS results

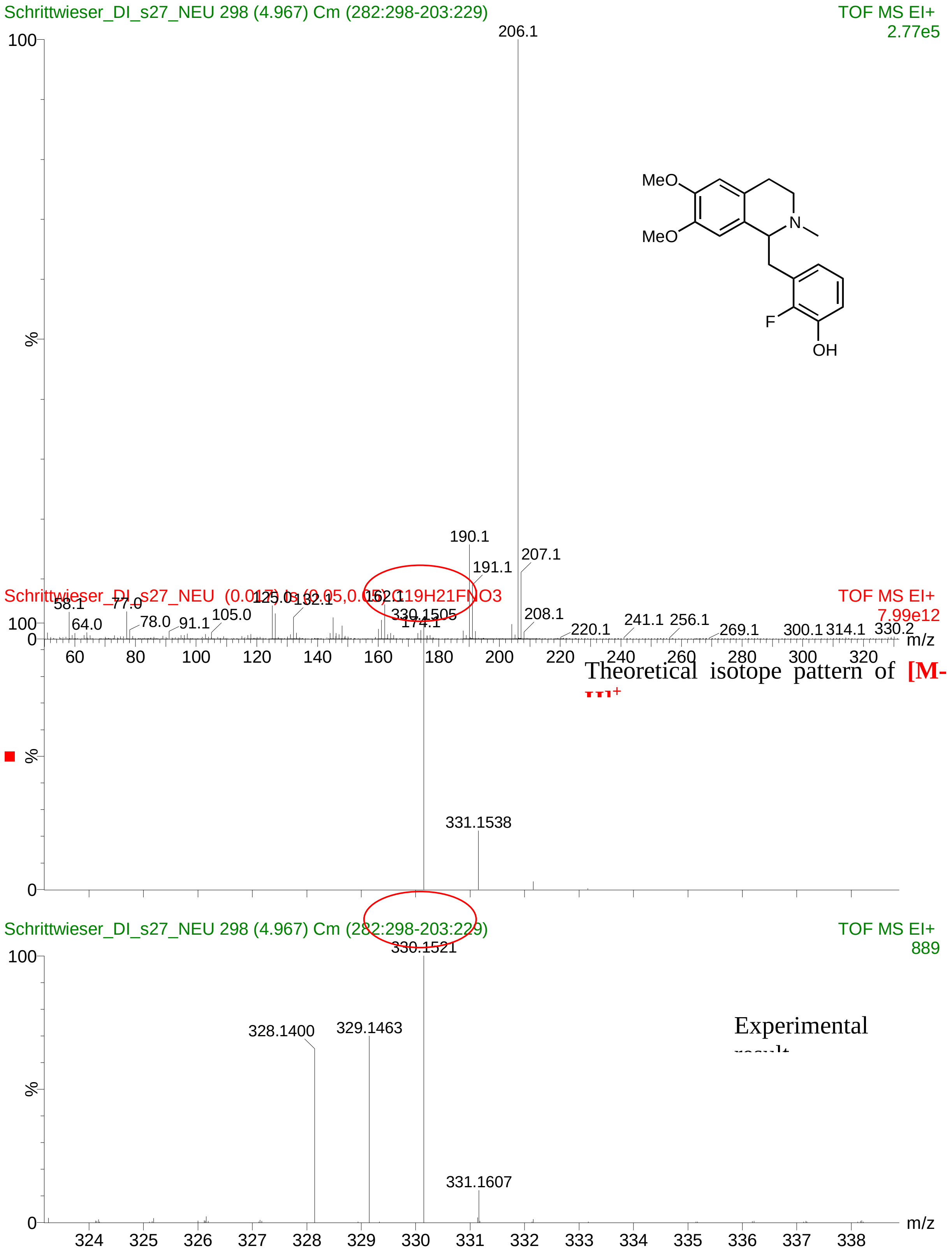

Synthesis of **1o**:

Provided Material:

2-(3-(benzyloxy)-2-fluorophenyl)-N-(4-(benzyloxy)-3-methoxyphenethyl)-*N*-methylacetamide
^{1}H-NMR spectrum, ^{13}C-NMR spectrum, ^{13}C-NMR DEPT135 spectrum, ^{13}C-NMR DEPT90 spectrum, COSY spectrum, HSQC spectrum, HRMS results

7-(benzyloxy)-1-(3-(benzyloxy)-2-fluorobenzyl)-6-methoxy-2-methyl-1,2,3,4-tetrahydroisoquinoline
^{1}H-NMR spectrum, ^{13}C-NMR spectrum, HSQC spectrum, HRMS results

1-(2-Fluoro-3-hydroxybenzyl)-6-methoxy-2-methyl-1,2,3,4-tetrahydroisoquinolin-7-ol
^{1}H-NMR spectrum, ^{13}C-NMR spectrum, COSY spectrum, HSQC spectrum, HRMS results

2-(3-(benzyloxy)-2-fluorophenyl)-N-(4-(benzyloxy)-3-methoxyphenethyl)-*N*-methylacetamide

^{1}H-NMR

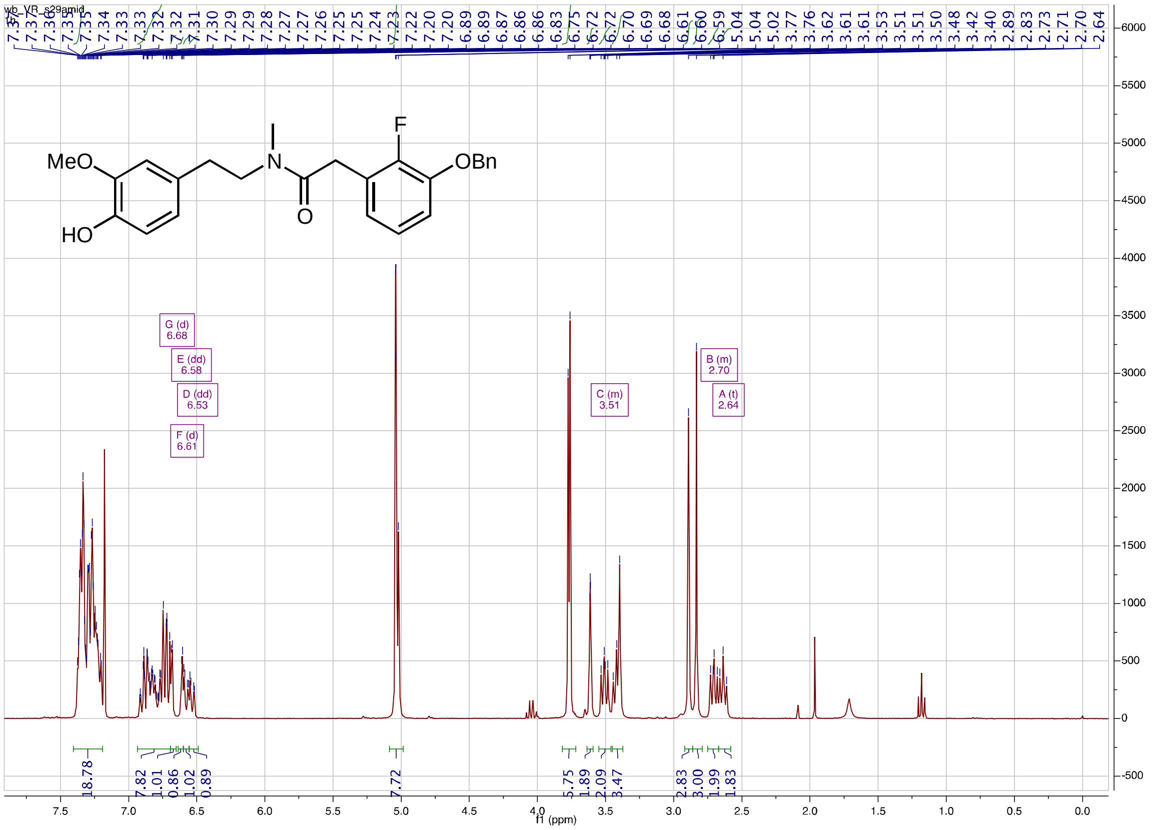

^{13}C-NMR

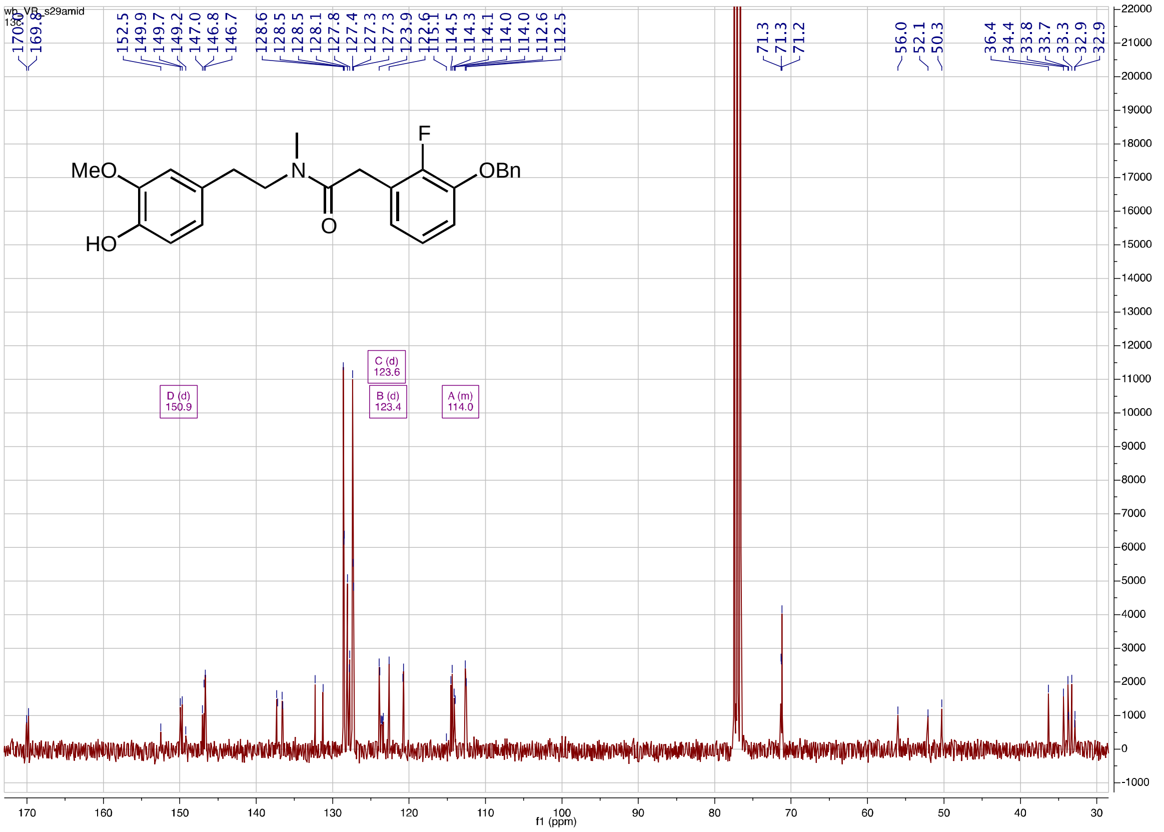

HSQC spectrum

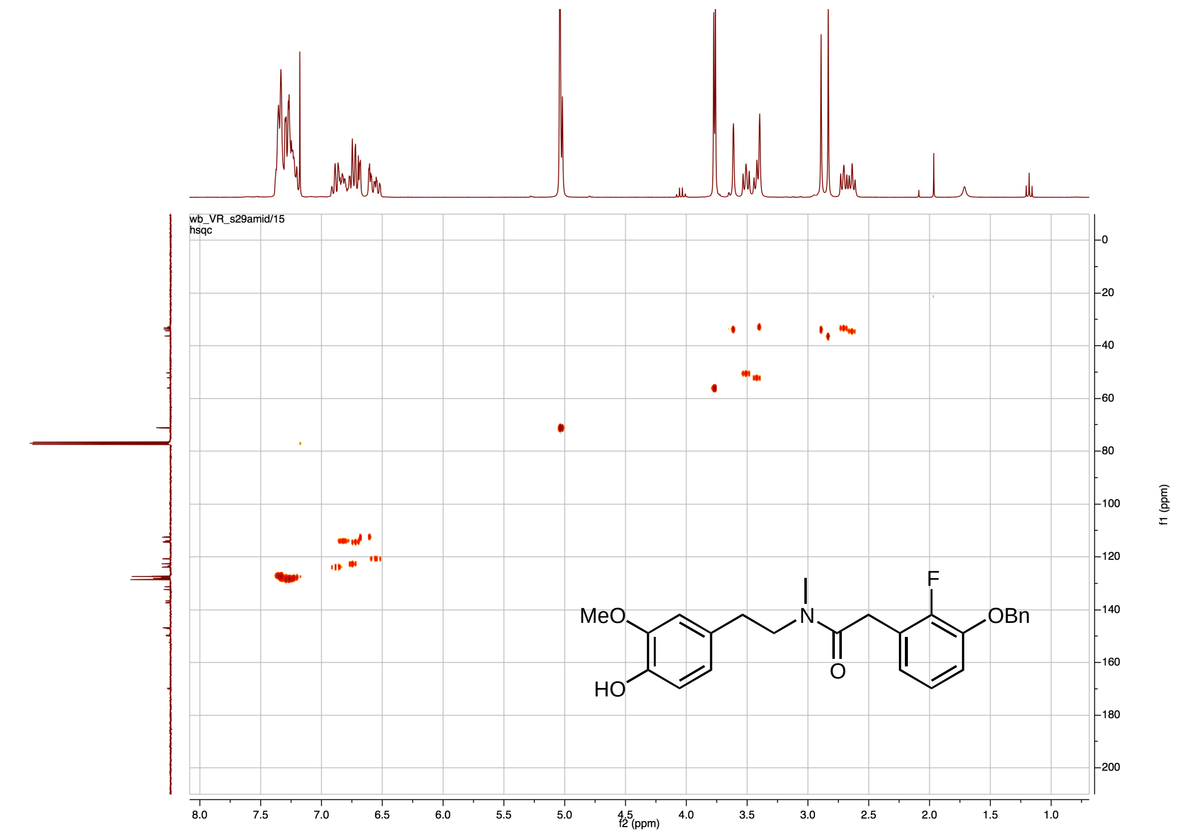

COSY spectrum

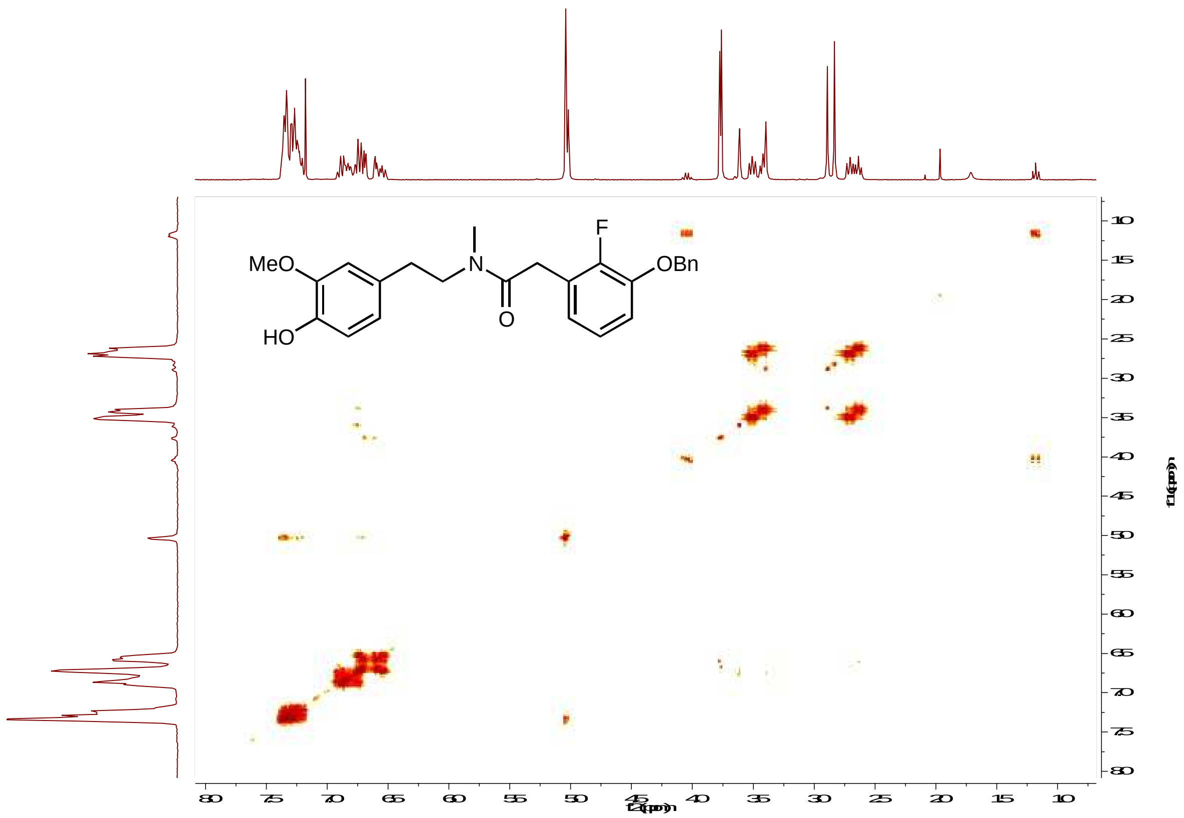

^{13}C-NMR DEPT135 spectrum

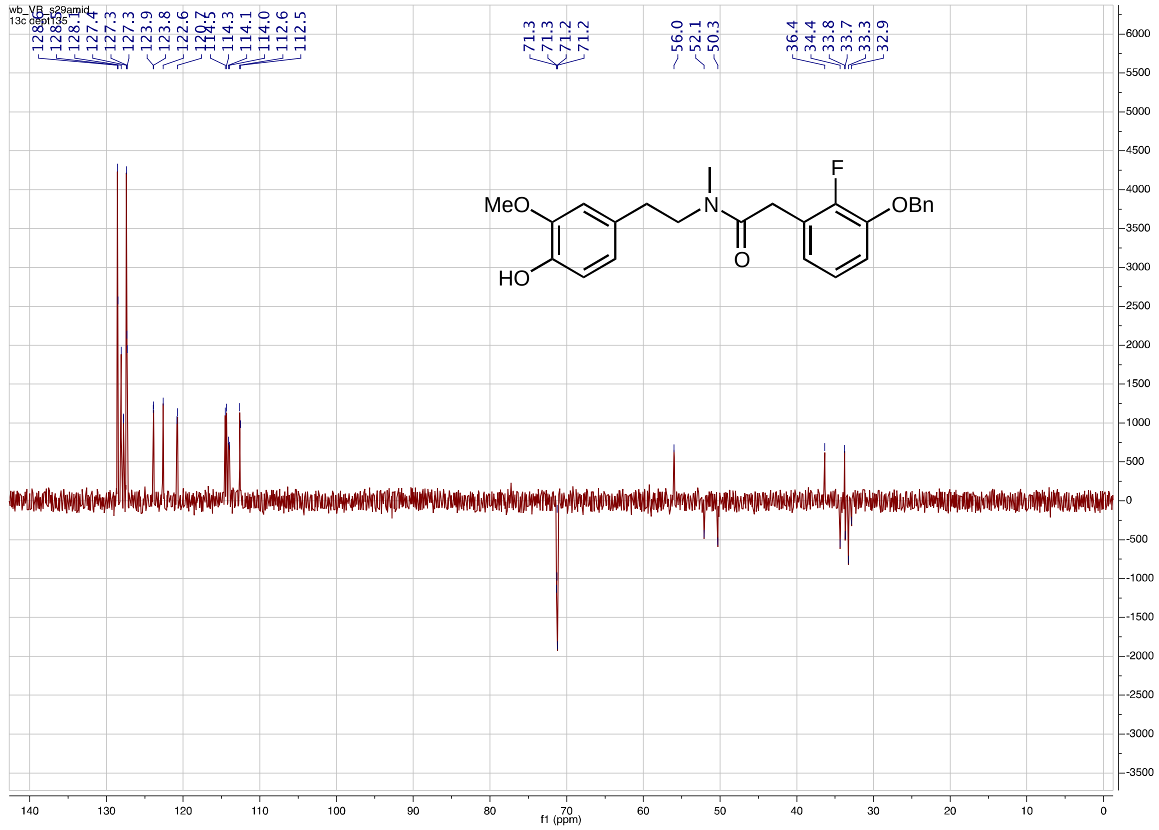

^{13}C-NMR DEPT90

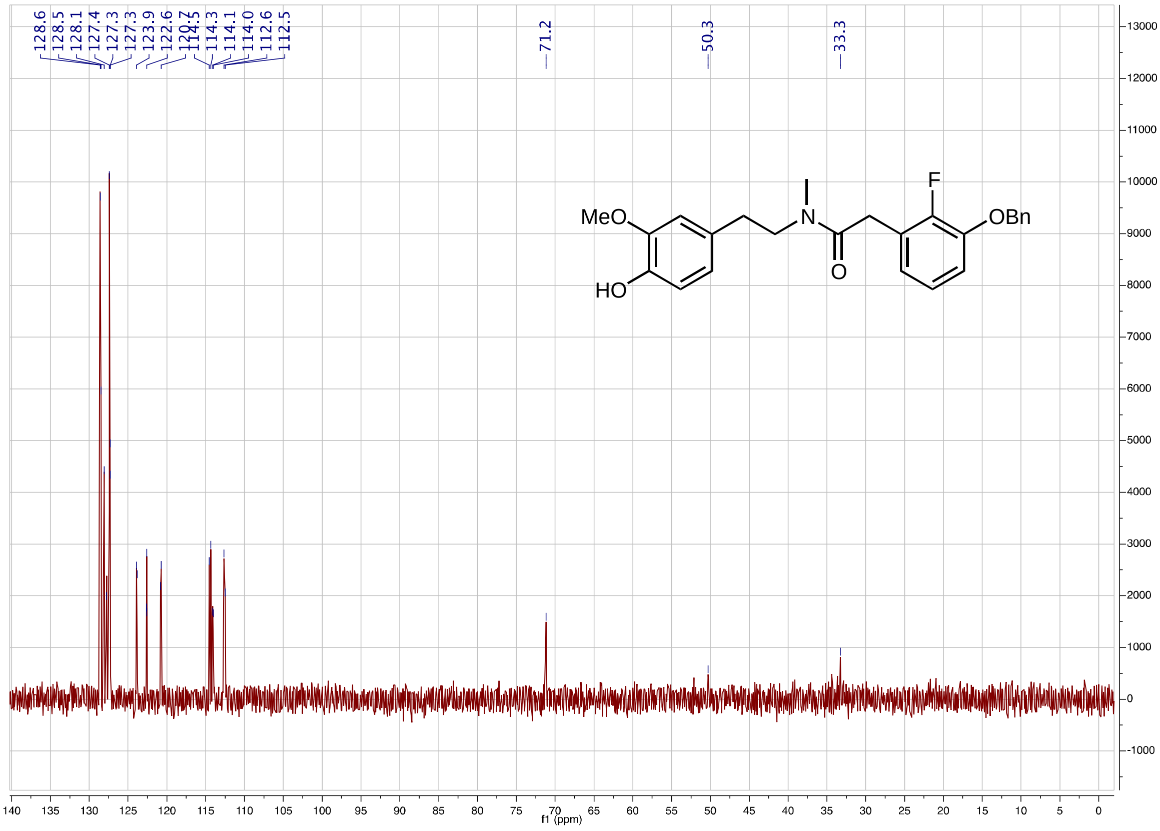

HRMS results

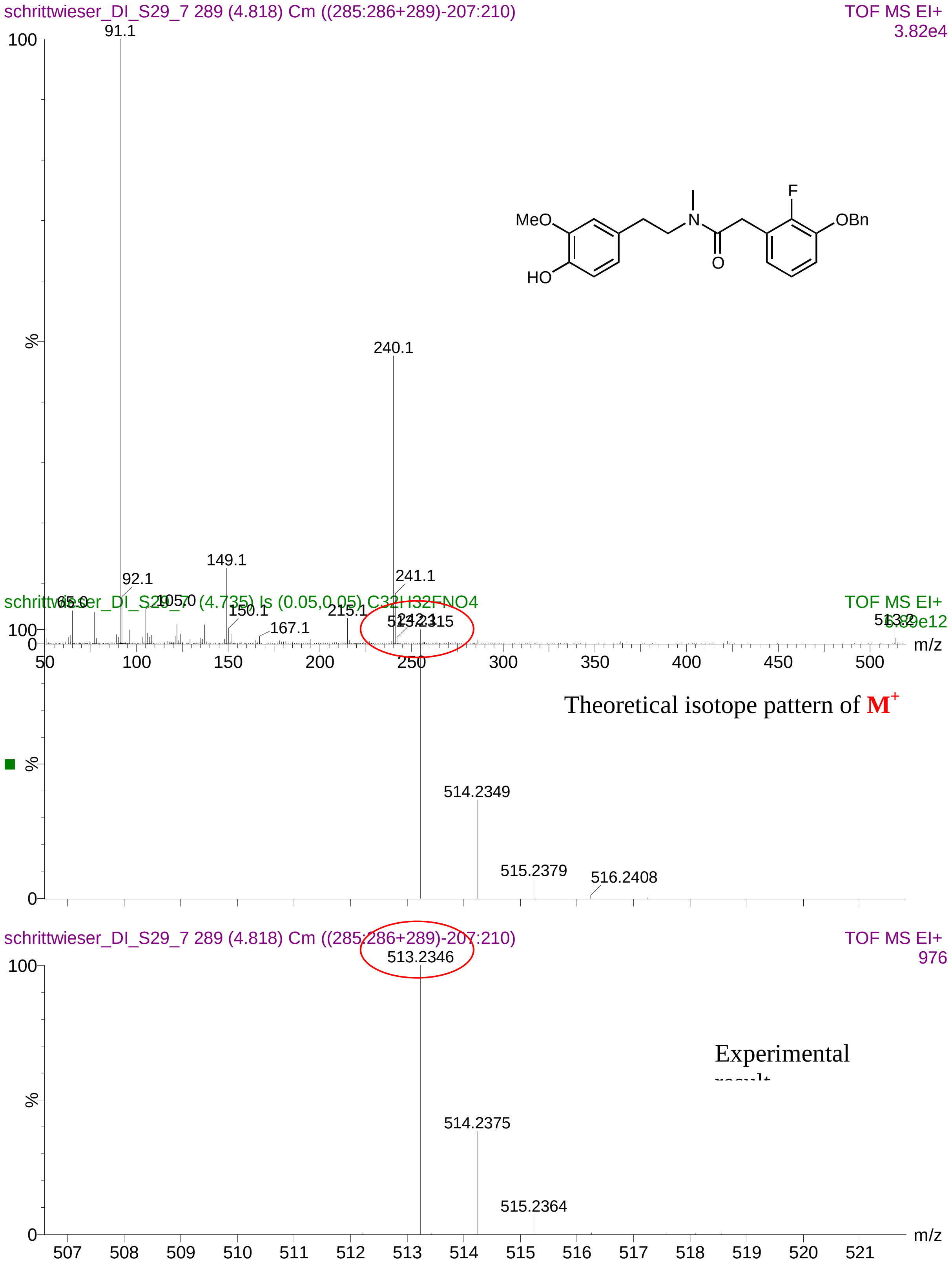

7-(benzyloxy)-1-(3-(benzyloxy)-2-fluorobenzyl)-6-methoxy-2-methyl-1,2,3,4-tetrahydroisoquinoline

^{1}H-NMR

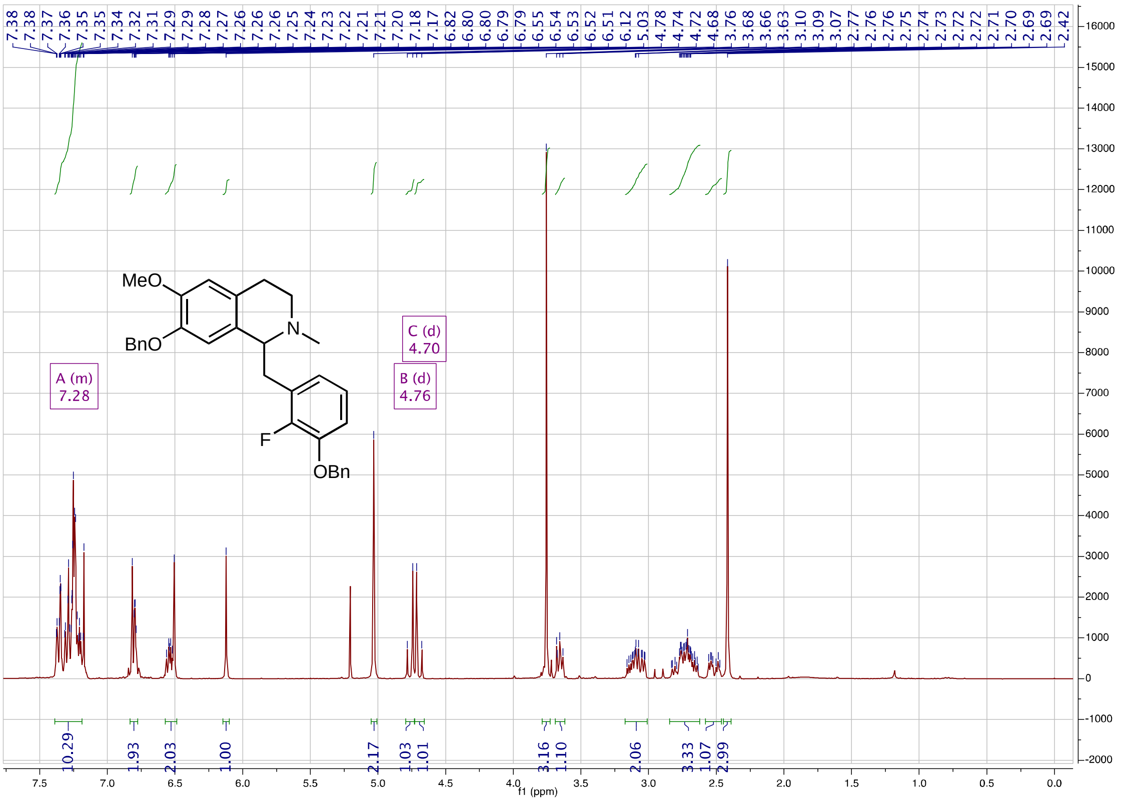

^{13}C-NMR

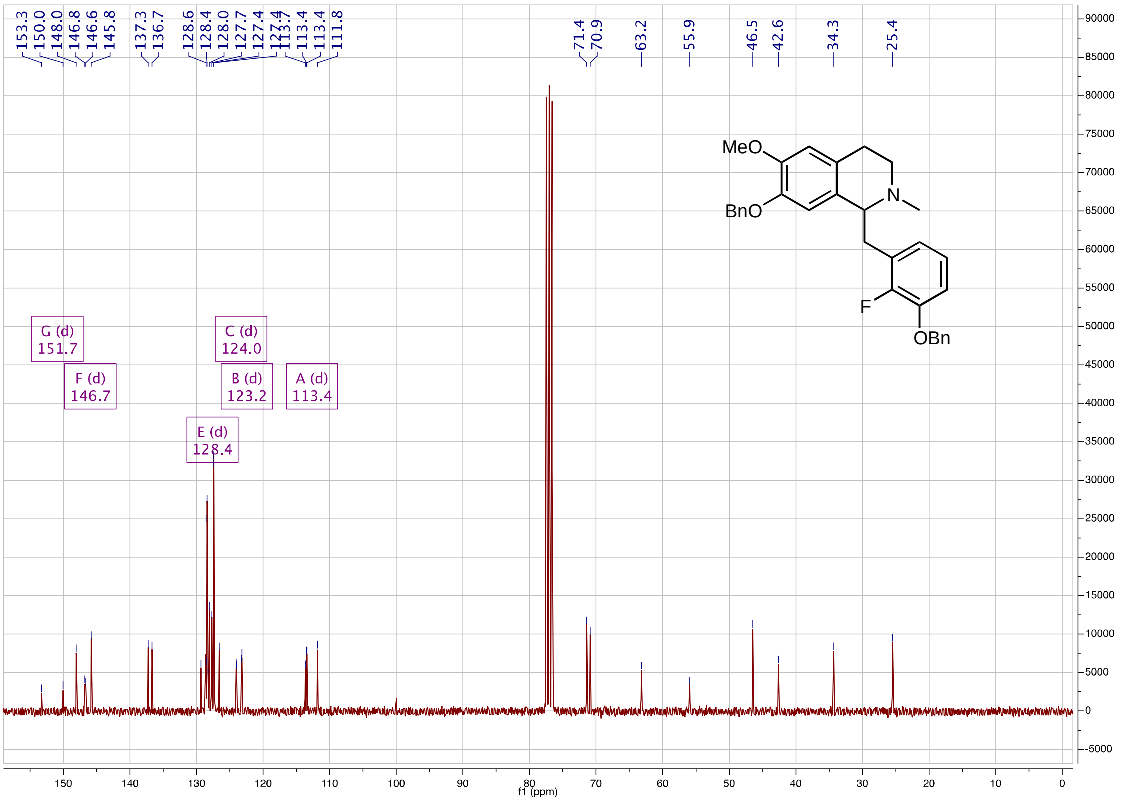

HSQC spectrum

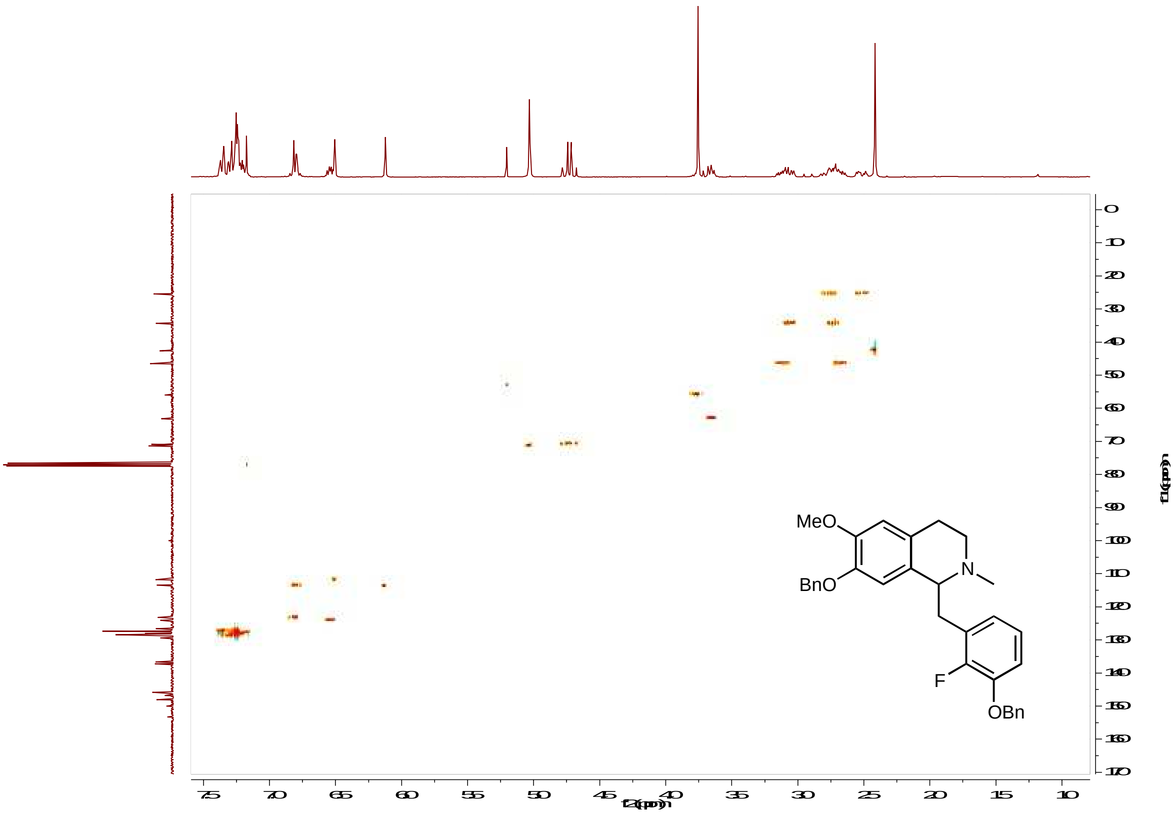

HRMS results

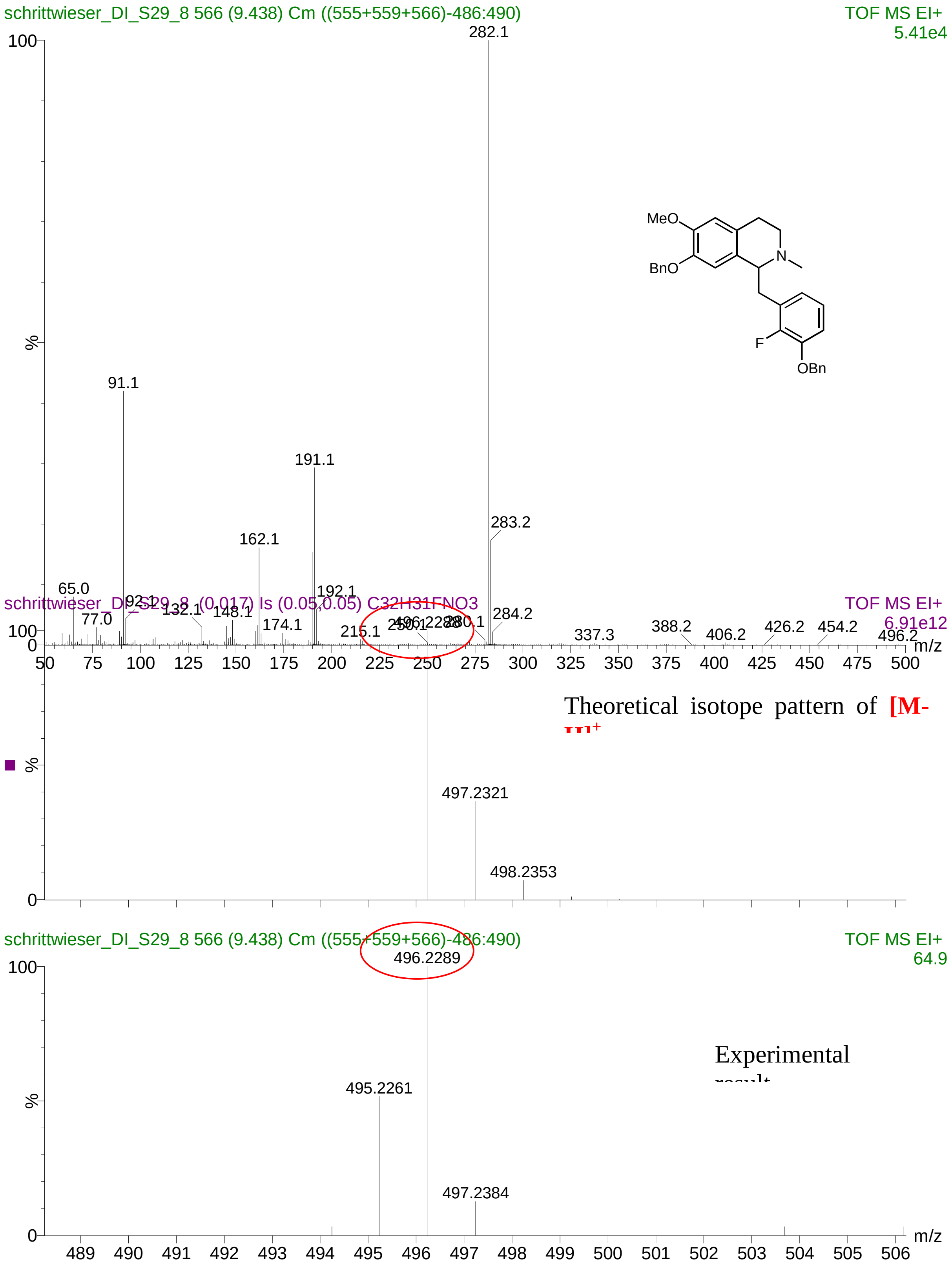

1-(2-Fluoro-3-hydroxybenzyl)-6-methoxy-2-methyl-1,2,3,4-tetrahydroisoquinolin-7-ol

^{1}H-NMR

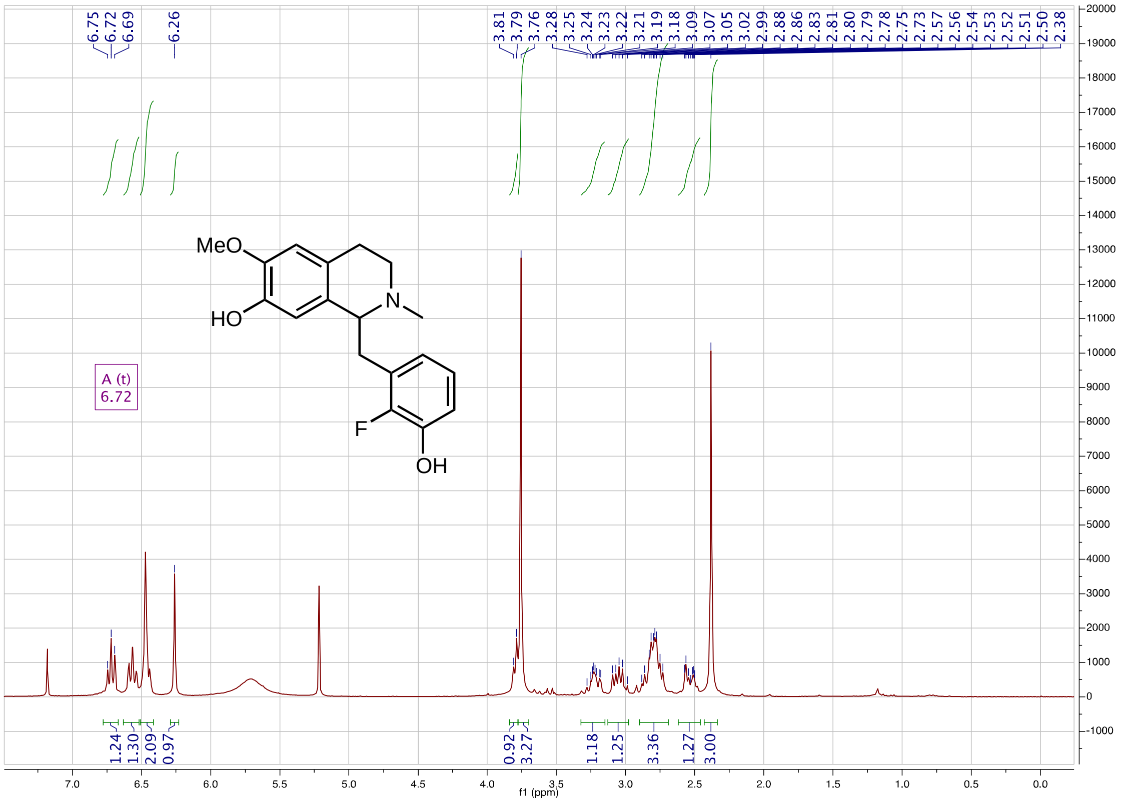

^{13}C-NMR

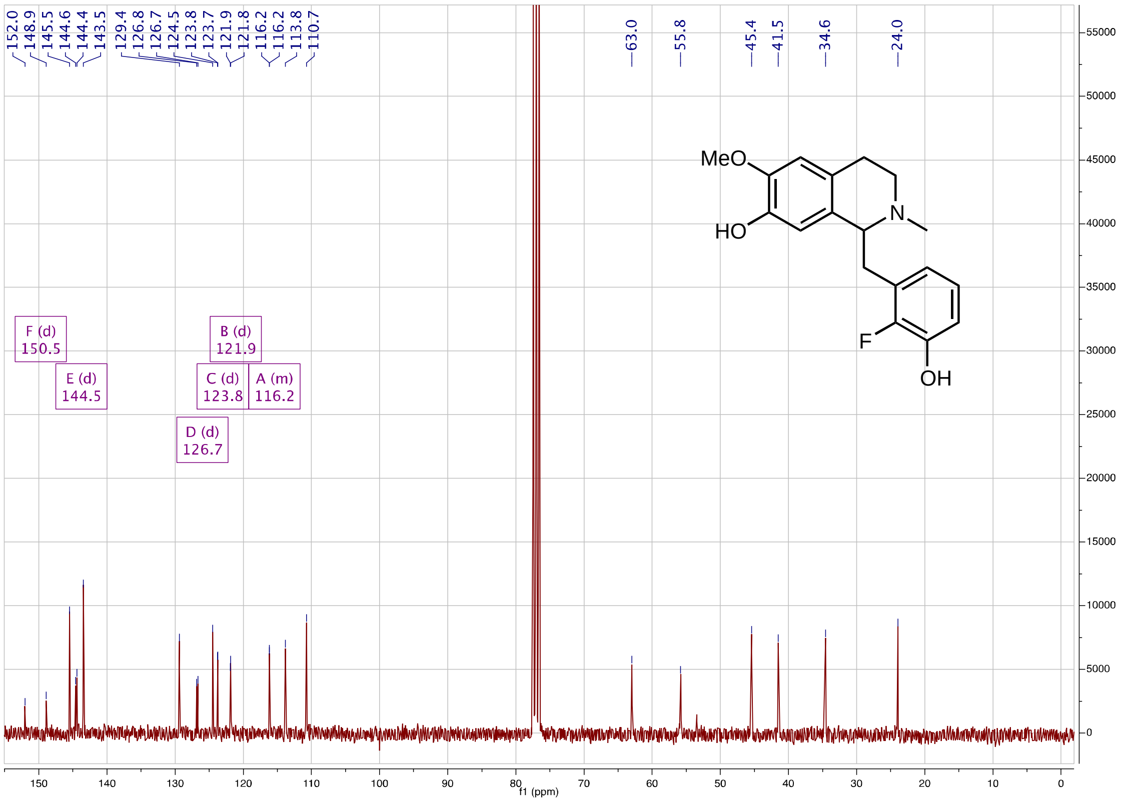

COSY spectrum

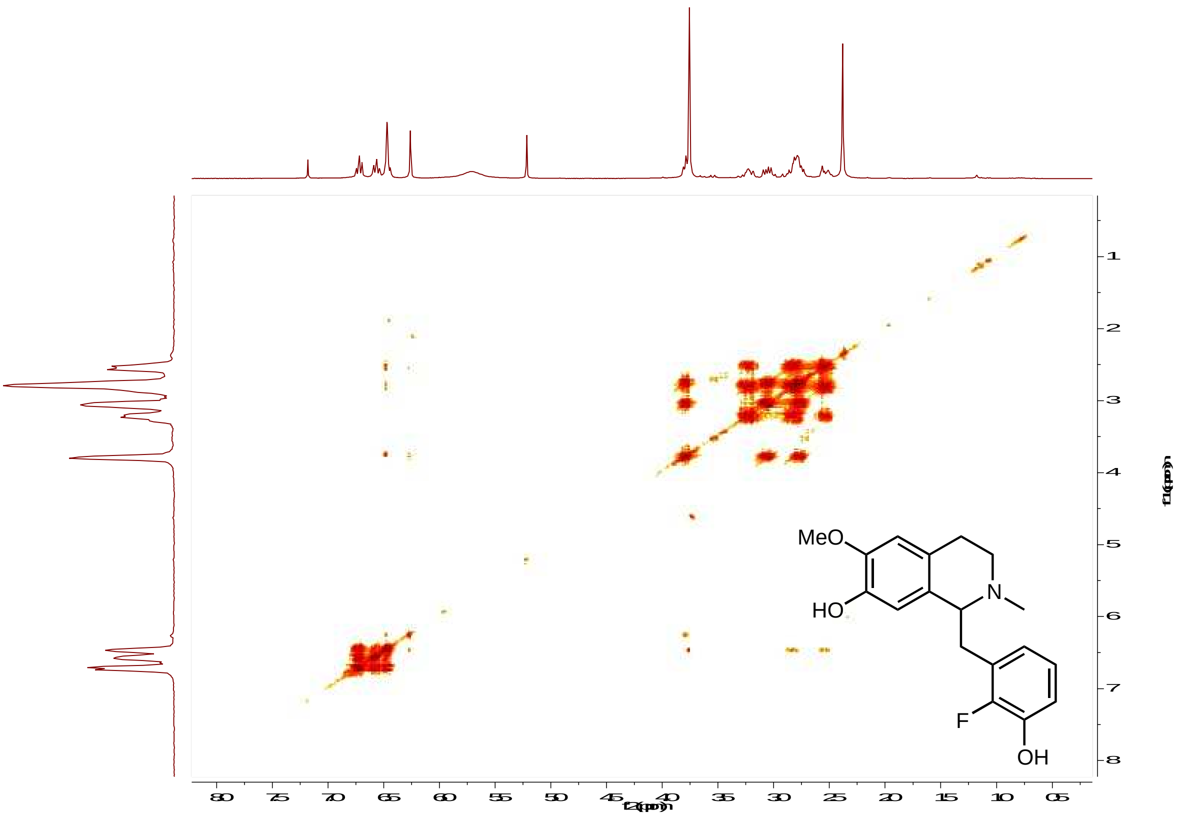

HSQC spectrum

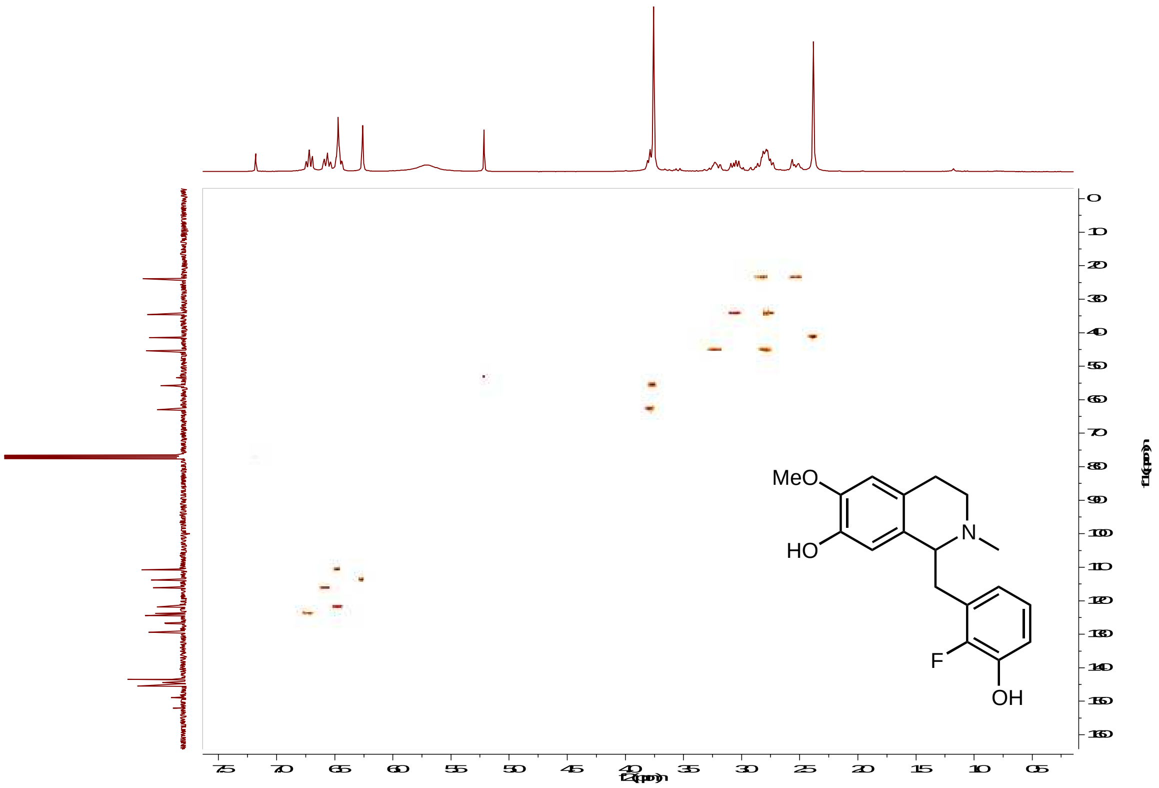

HRMS results

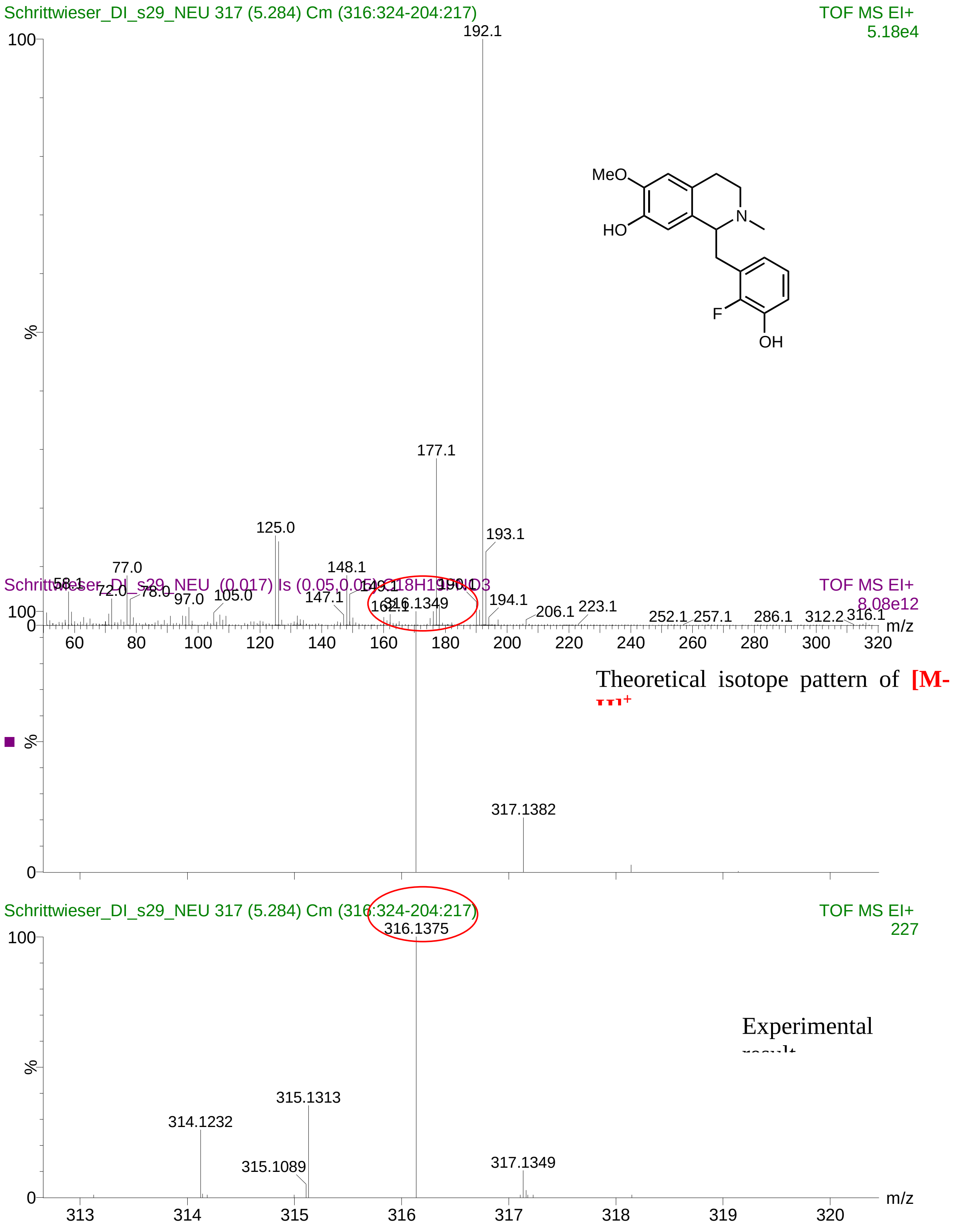

Synthesis of **1p**:

Provided Material:

2-(3-(benzyloxy)-2-fluorophenyl)-N-(3-(benzyloxy)-4-methoxyphenethyl)-*N*-methylacetamide
^{1}H-NMR spectrum, ^{13}C-NMR spectrum, ^{13}C-NMR DEPT135 spectrum, ^{13}C-NMR DEPT90 spectrum, COSY spectrum, HSQC spectrum, HRMS results

6-(Benzyloxy)-1-(3-(benzyloxy)-2-fluorobenzyl)-7-methoxy-2-methyl-1,2,3,4-tetrahydroisoquinoline
^{1}H-NMR spectrum, ^{13}C-NMR spectrum, HRMS results

1-(2-Fluoro-3-hydroxybenzyl)-7-methoxy-2-methyl-1,2,3,4-tetrahydroisoquinolin-6-ol
^{1}H-NMR spectrum, ^{13}C-NMR spectrum, COSY spectrum, HSQC spectrum, HRMS results

2-(3-(Benzyloxy)-2-fluorophenyl)-*N*-(3-(benzyloxy)-4-methoxyphenethyl)-*N*-methylacetamide

^{1}H-NMR

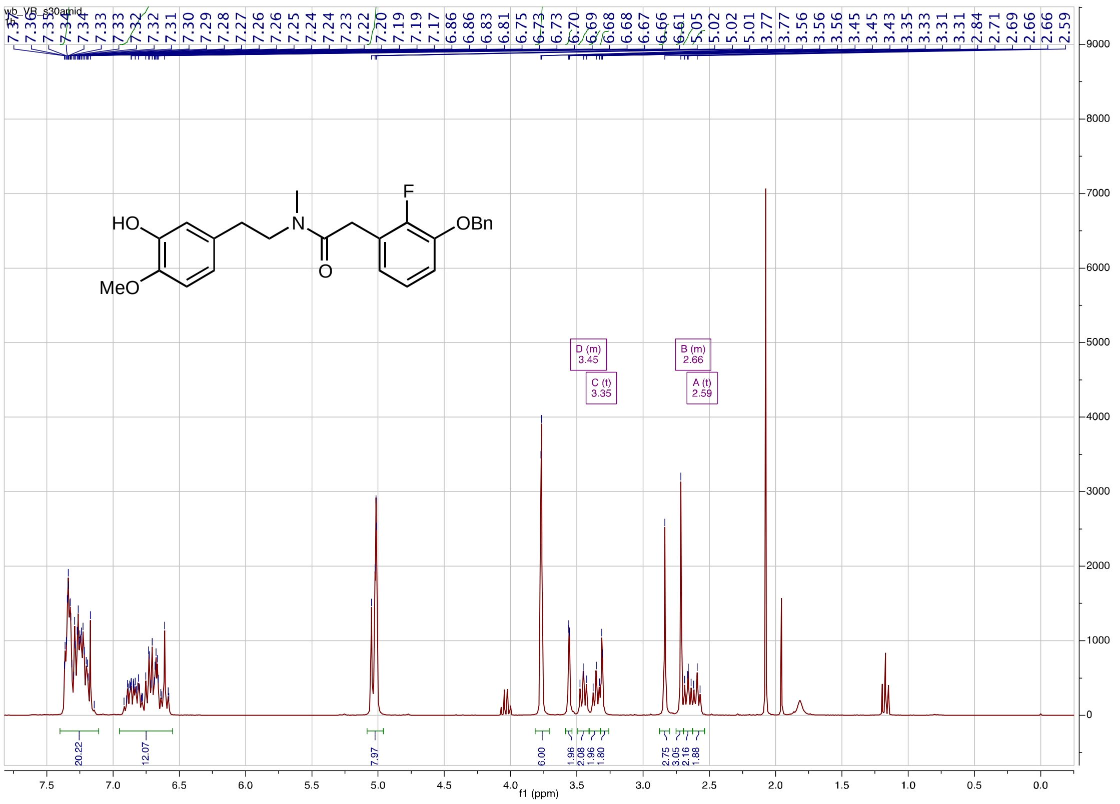

^{13}C-NMR

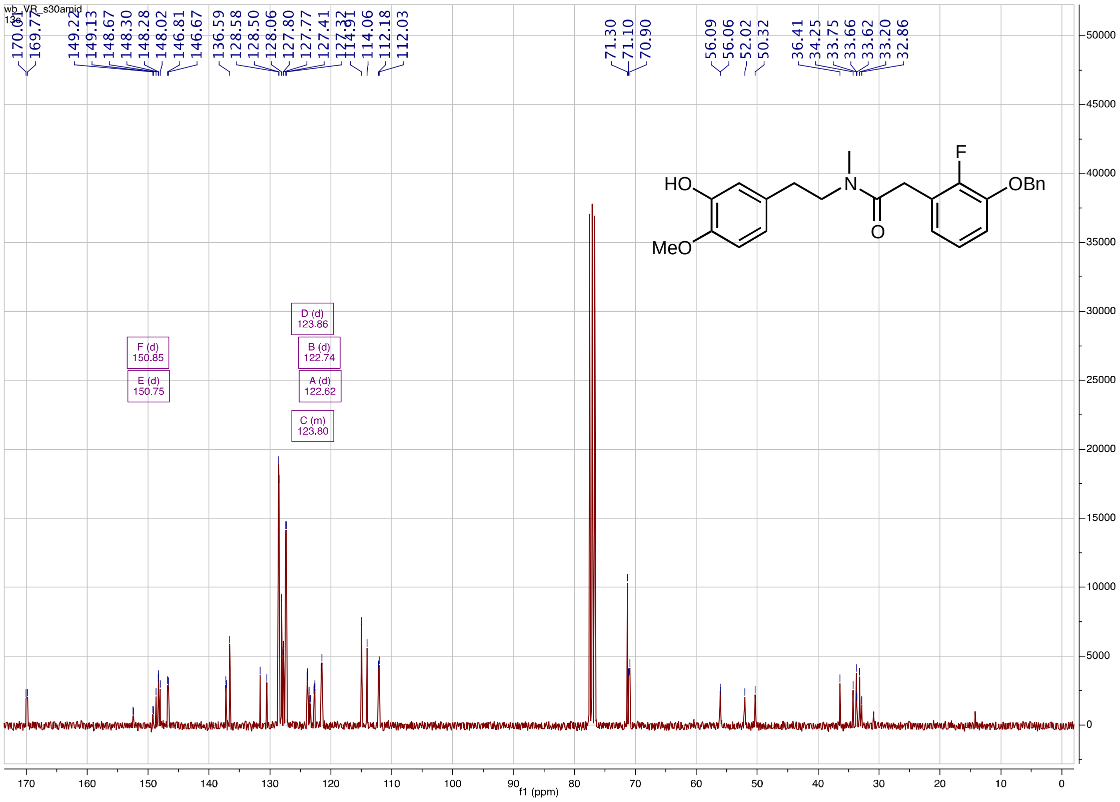

^{13}C-NMR DEPT90 spectrum

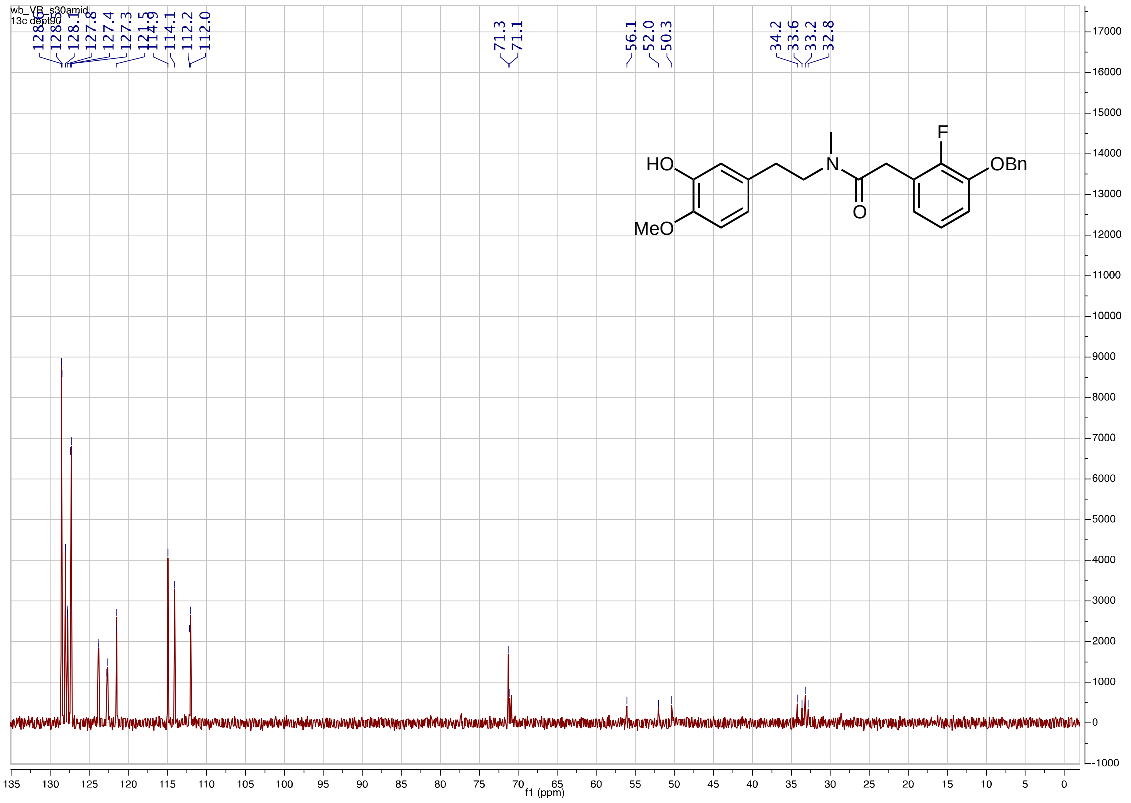

^{13}C-NMR DEPT135 spectrum

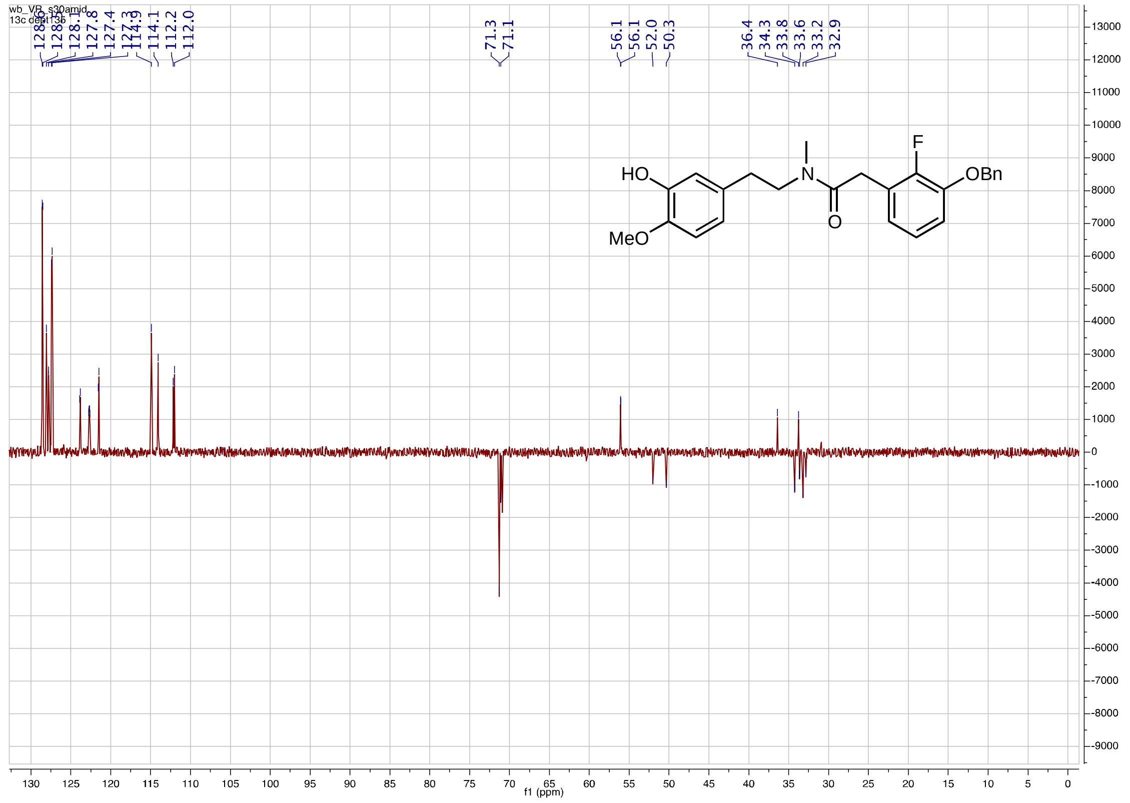

COSY spectrum

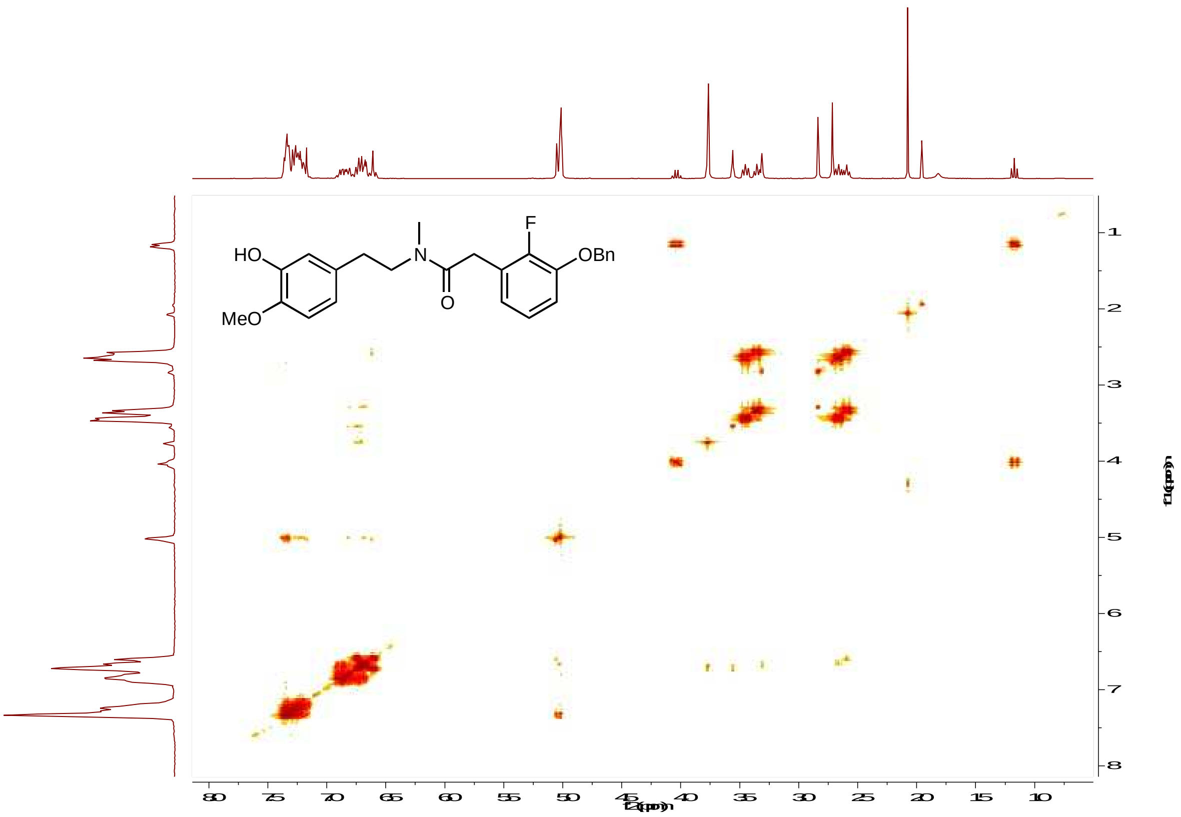

HSQC spectrum

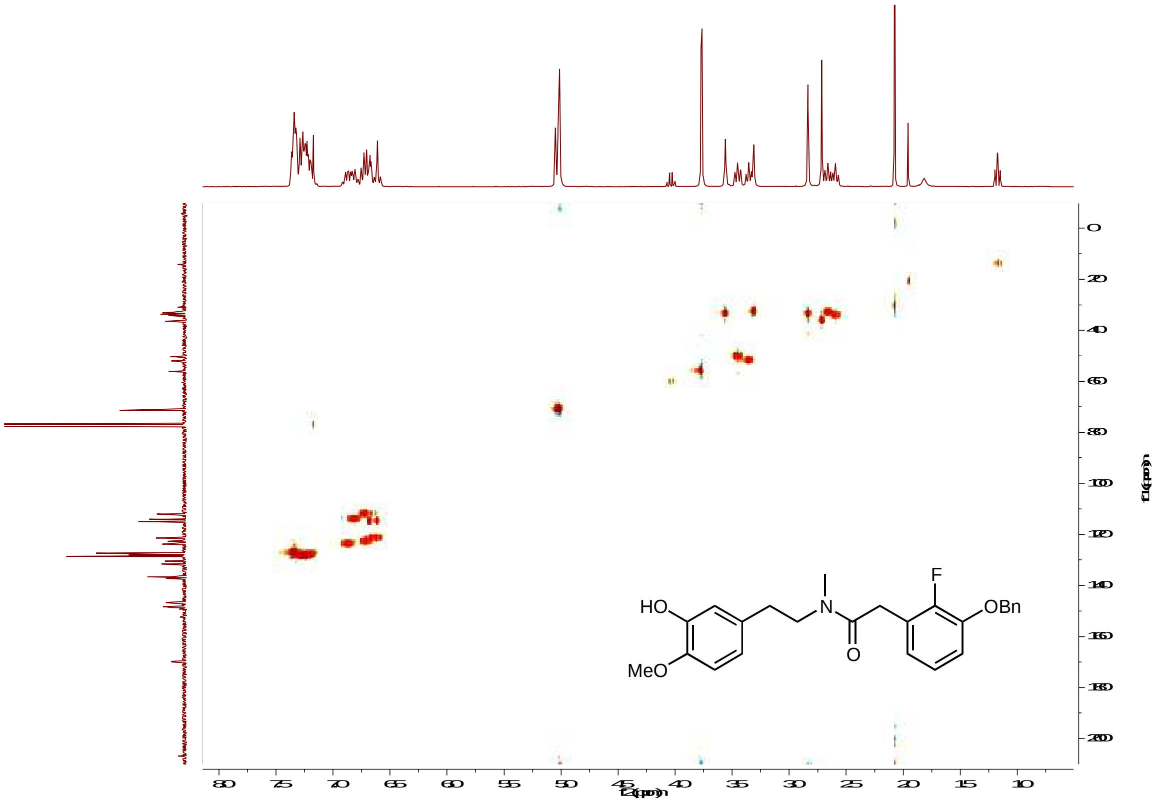

HRMS results

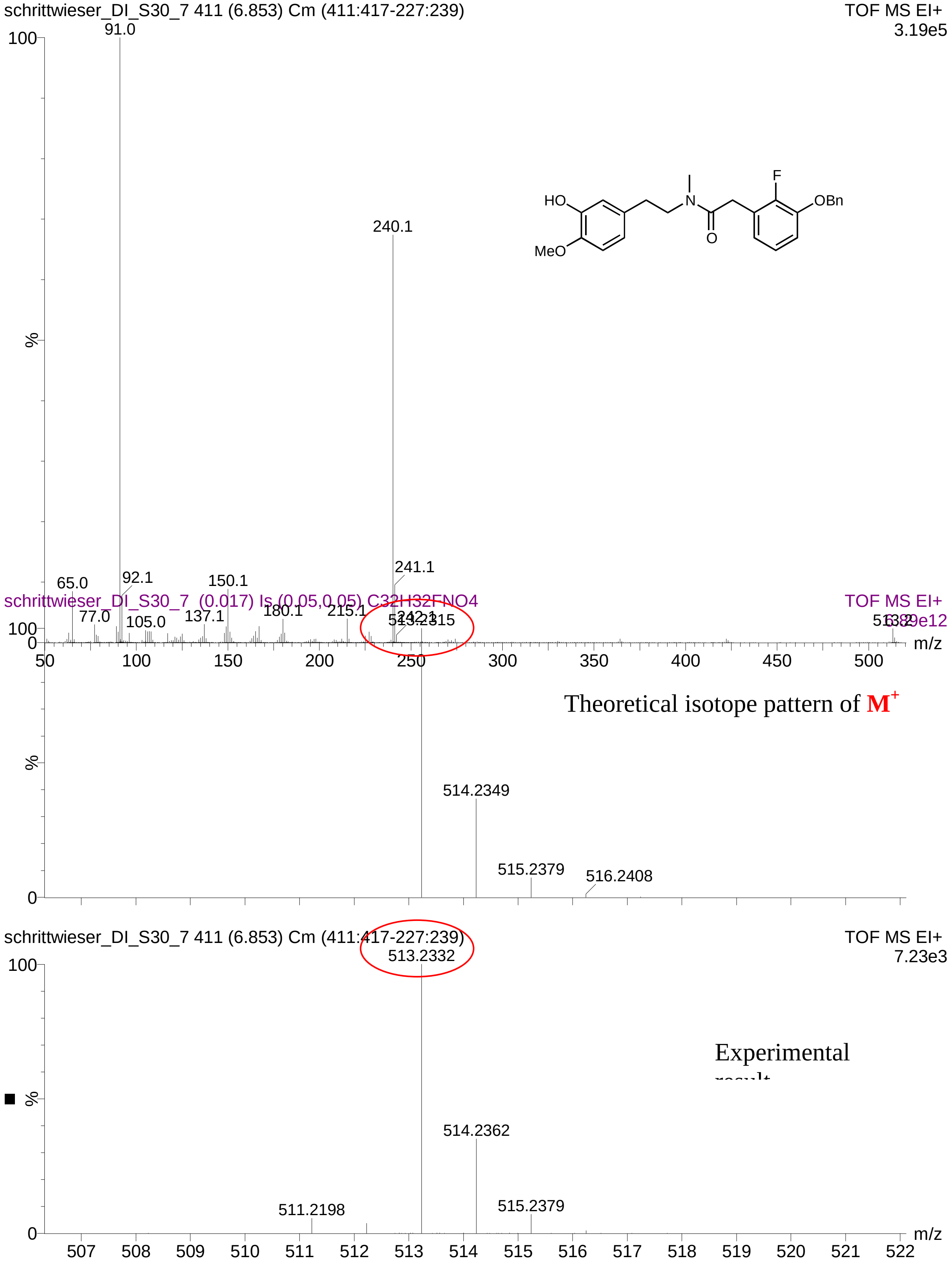

6-(Benzyloxy)-1-(3-(benzyloxy)-2-fluorobenzyl)-7-methoxy-2-methyl-1,2,3,4-tetrahydroisoquinoline

^{1}H-NMR

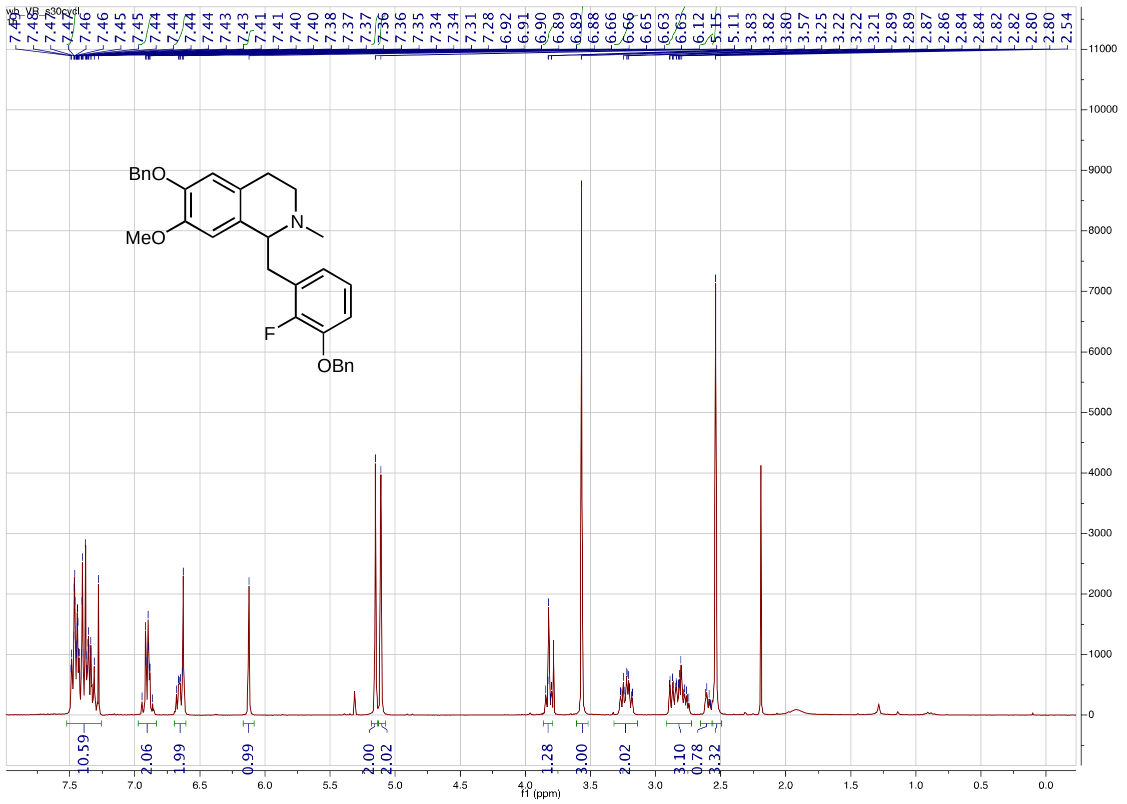

^{13}C-NMR

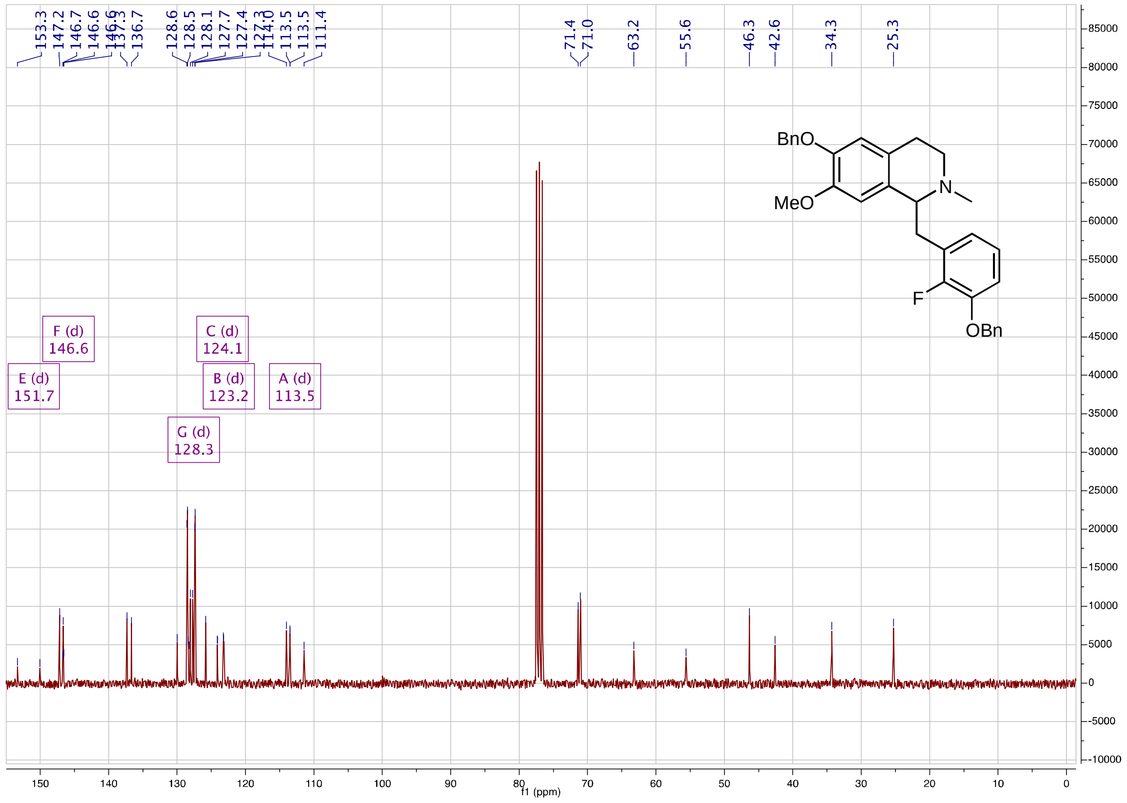

HRMS results

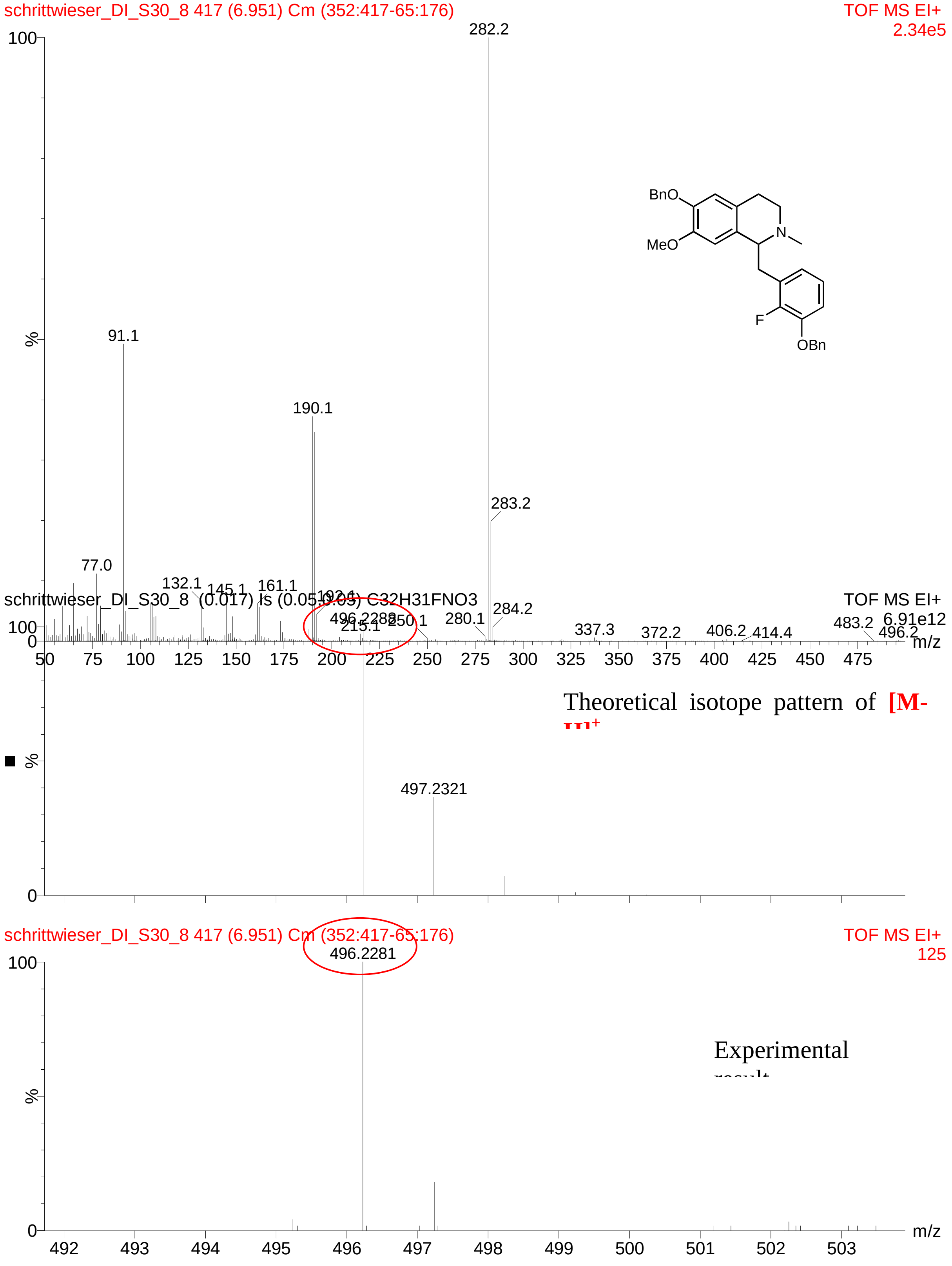

1-(2-Fluoro-3-hydroxybenzyl)-7-methoxy-2-methyl-1,2,3,4-tetrahydroisoquinolin-6-ol

^{1}H-NMR

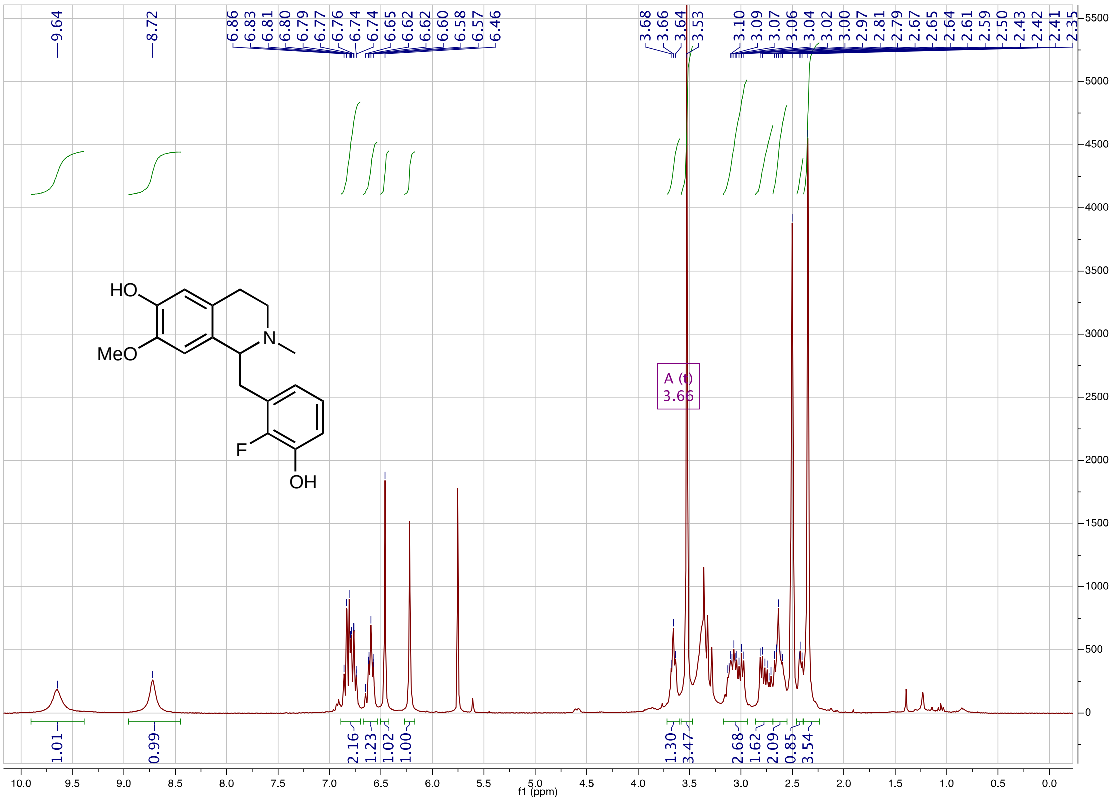

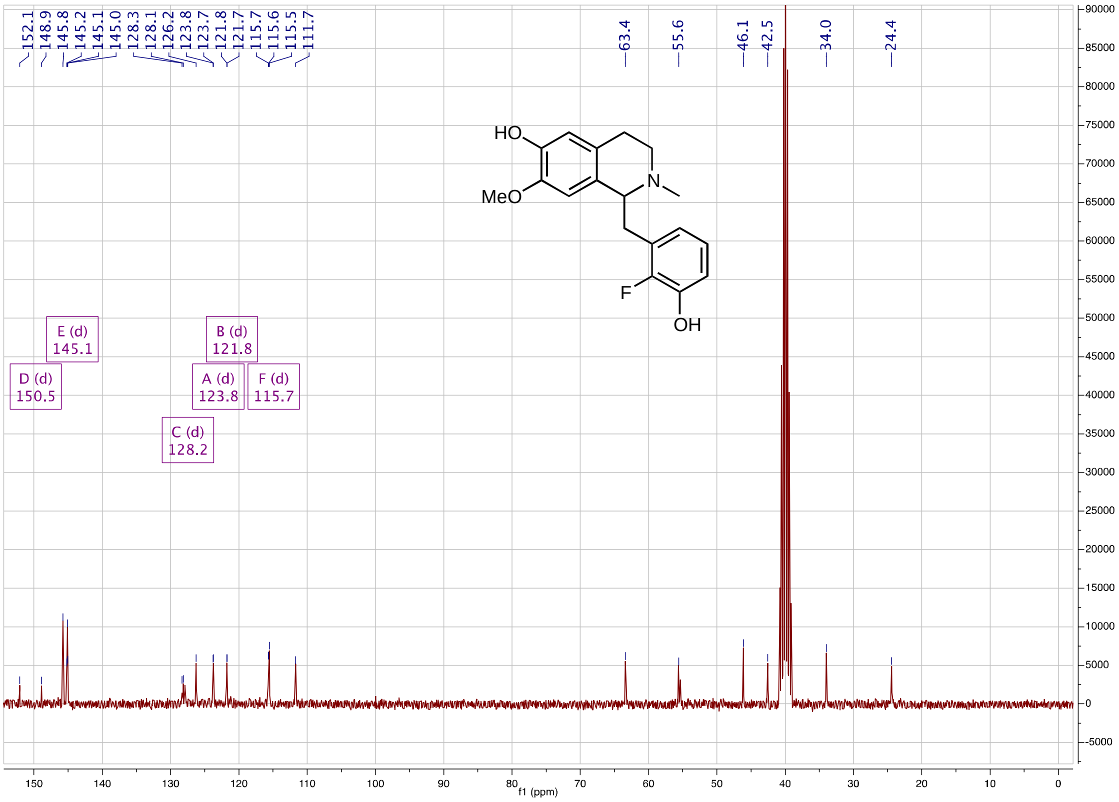

COSY spectrum

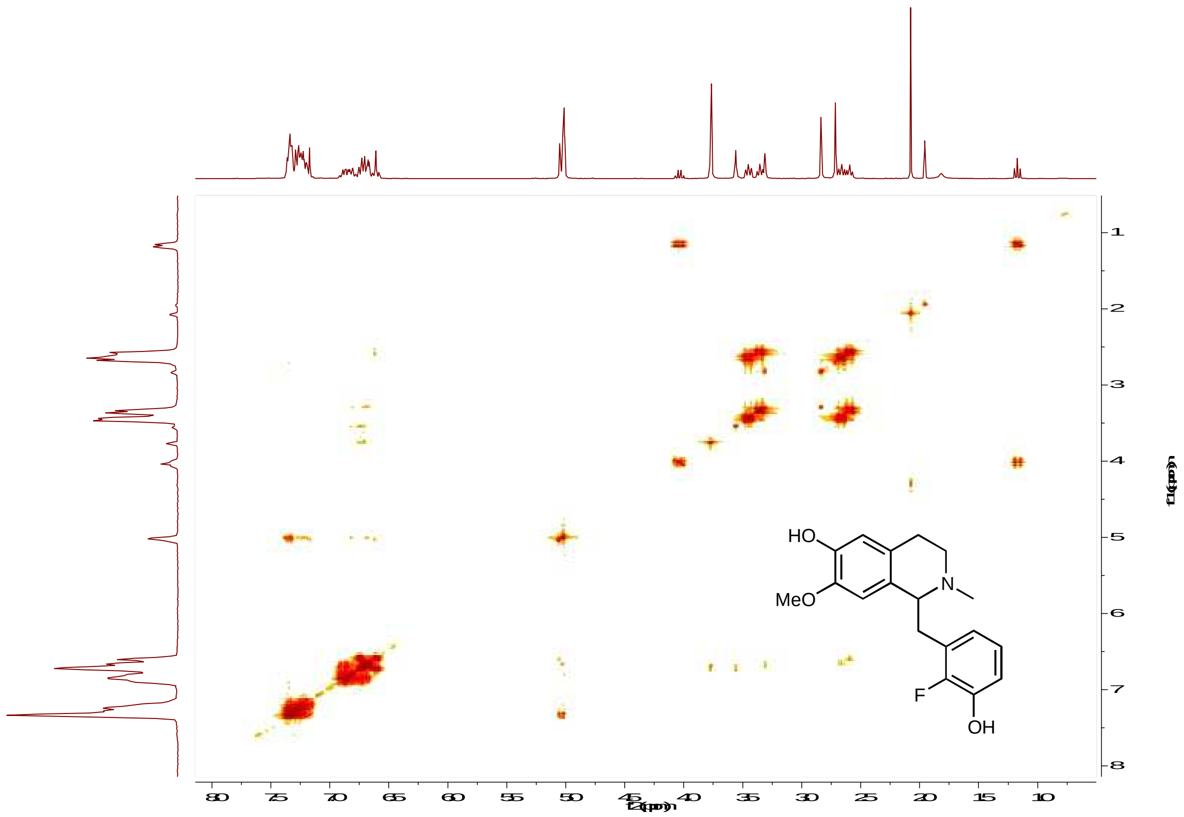

HSQC spectrum

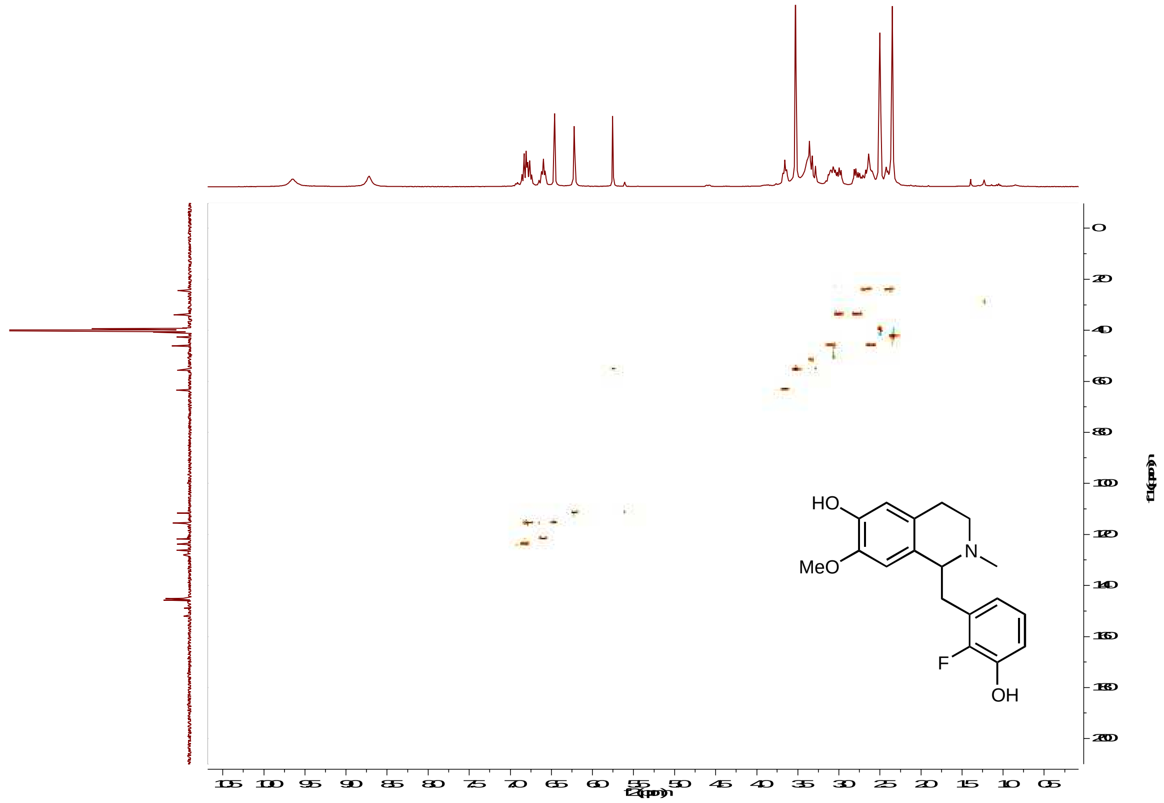

HRMS results

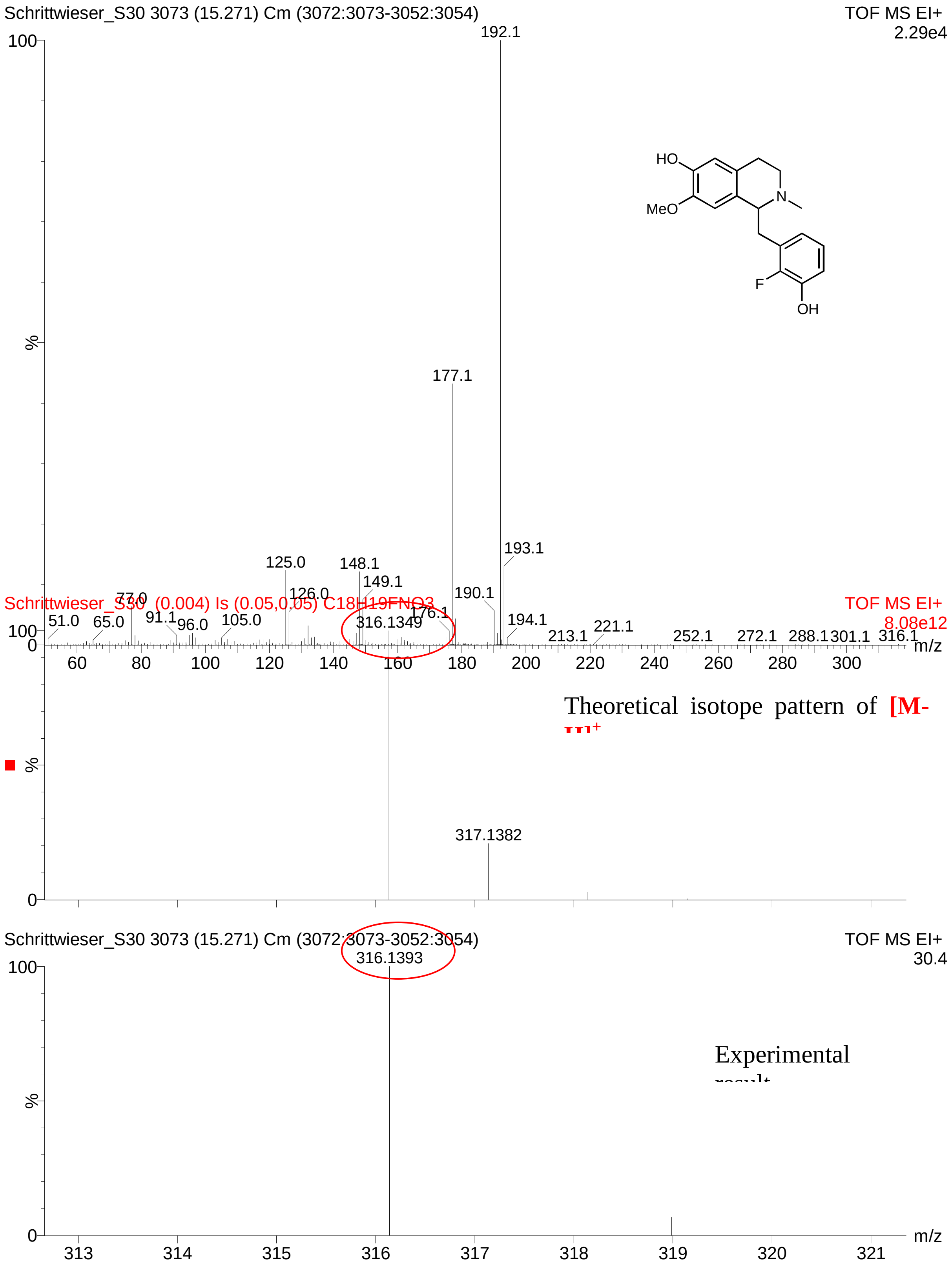

(*R*)-**1k**:

Provided Material:

(*R*)-**1k**
^{1}H-NMR spectrum, ^{13}C-NMR spectrum, COSY spectrum, HSQC spectrum, HRMS results

^{1}H-NMR

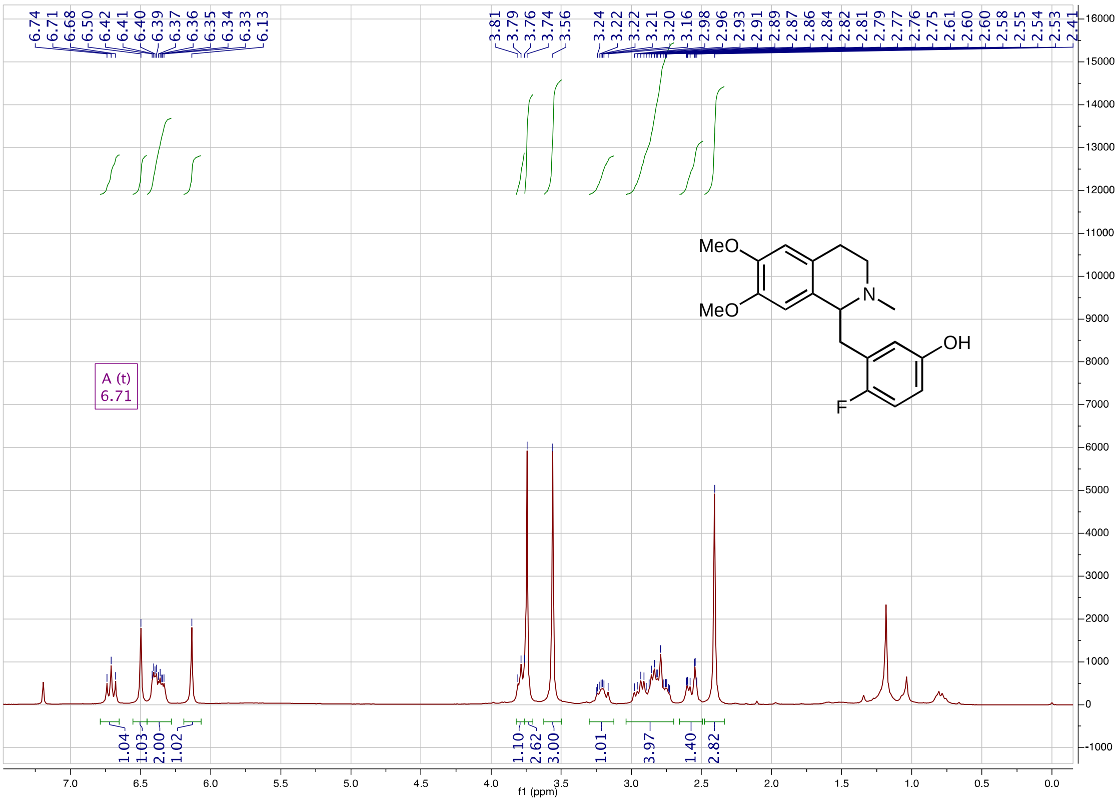

^{13}C-NMR

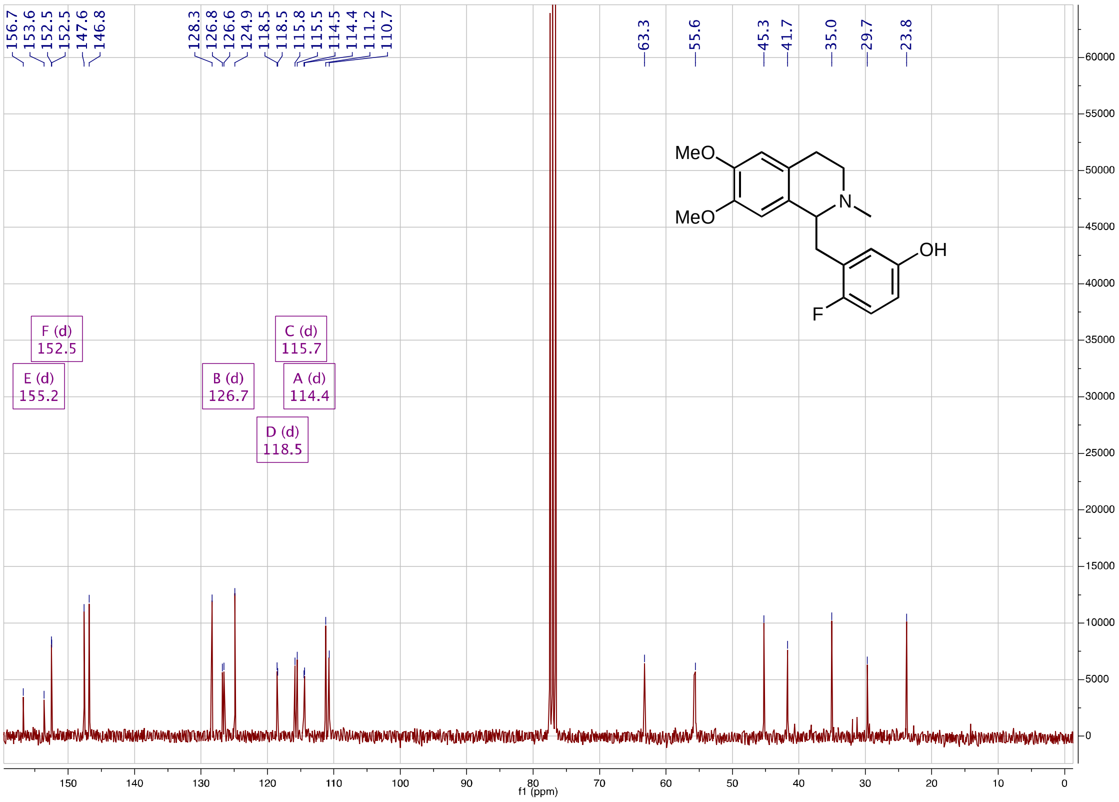

COSY spectrum

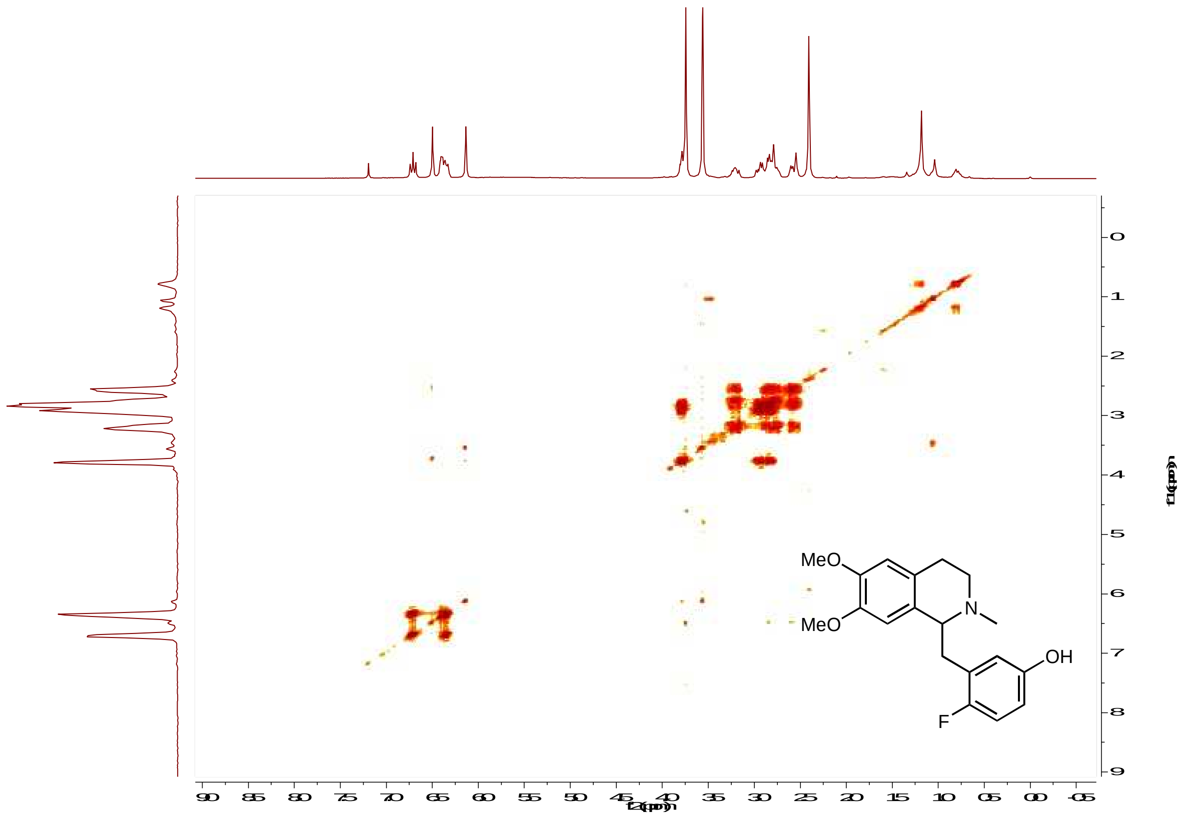

HSQC spectrum

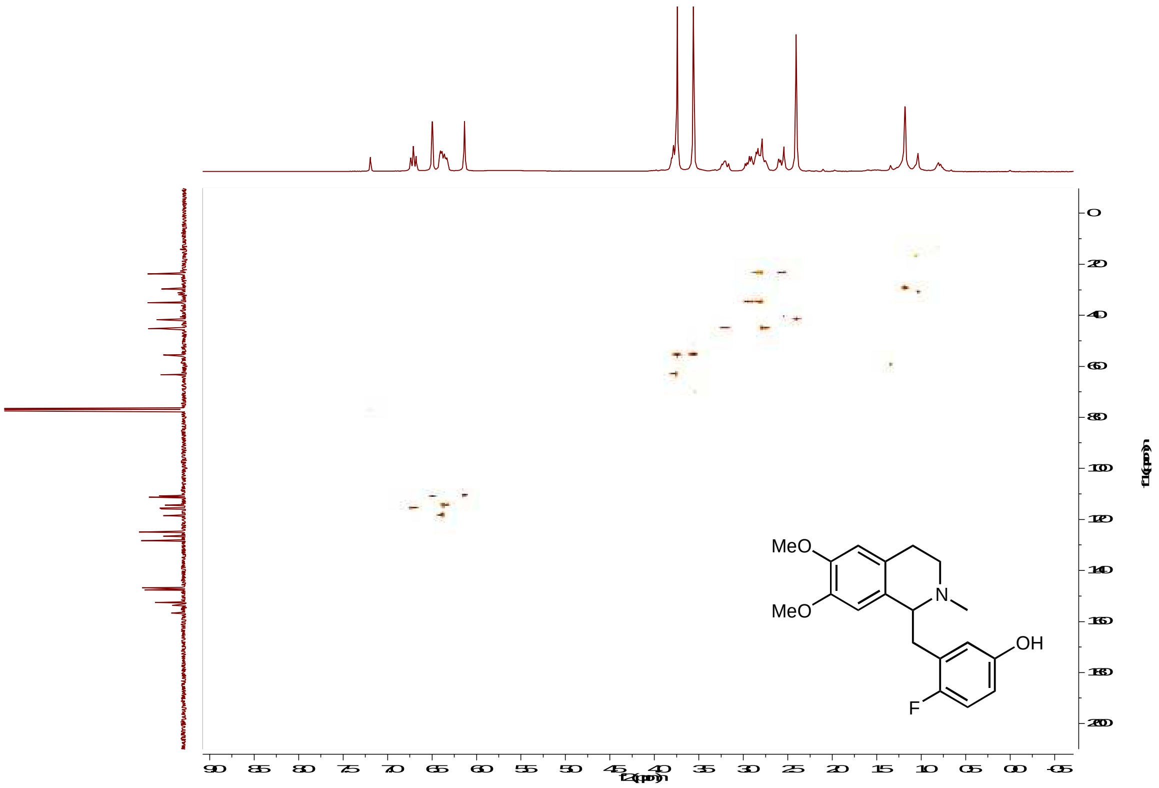

HRMS results

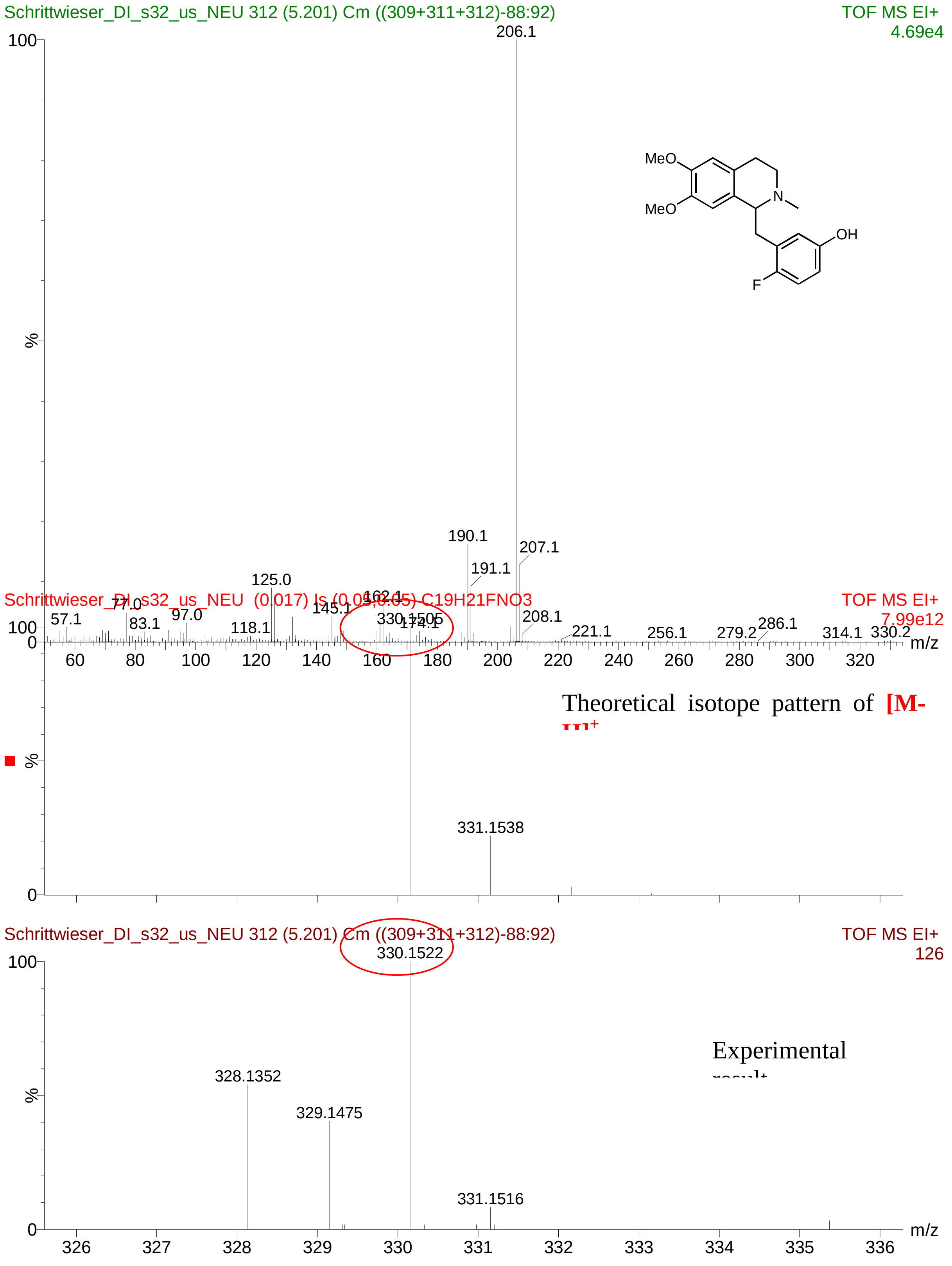

(*S*)-**2k**:

Provided Material:

(*S*)-**2k**
^{1}H-NMR spectrum, ^{13}C-NMR spectrum, COSY spectrum, HSQC spectrum, HRMS results

^{1}H-NMR

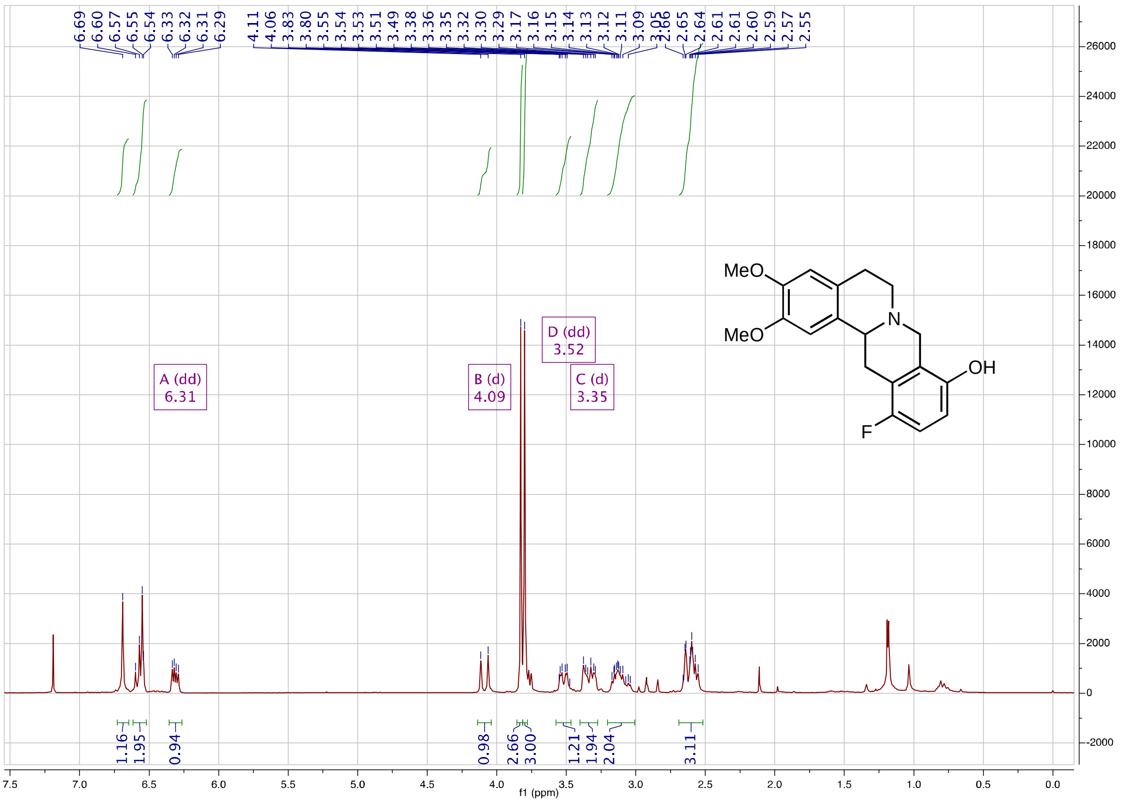

^{13}C-NMR

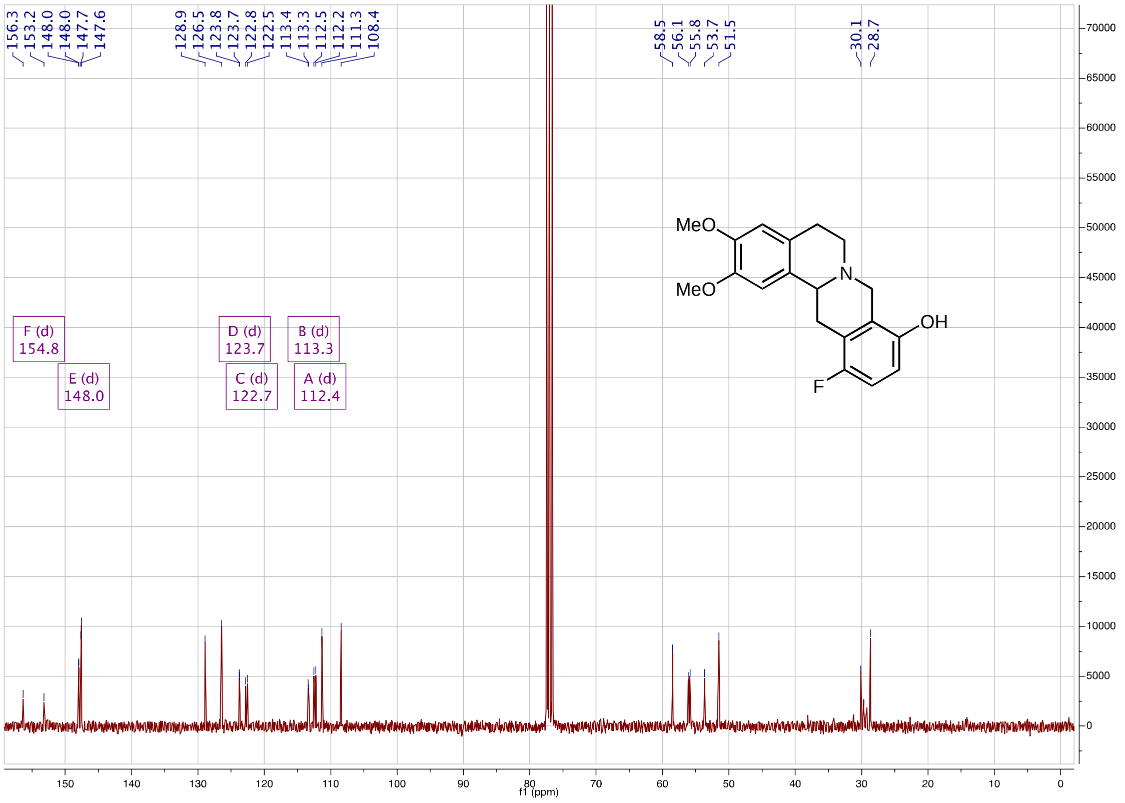

COSY spectrum

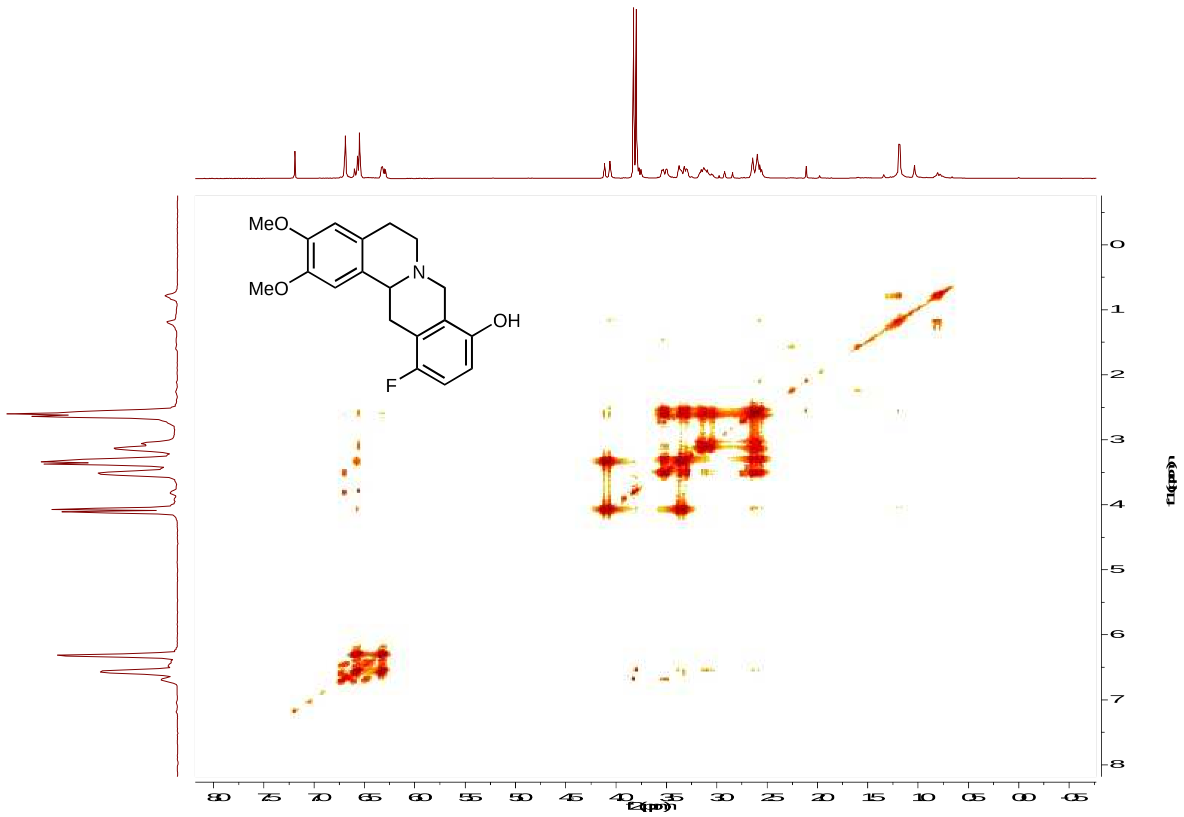

HSQC spectrum

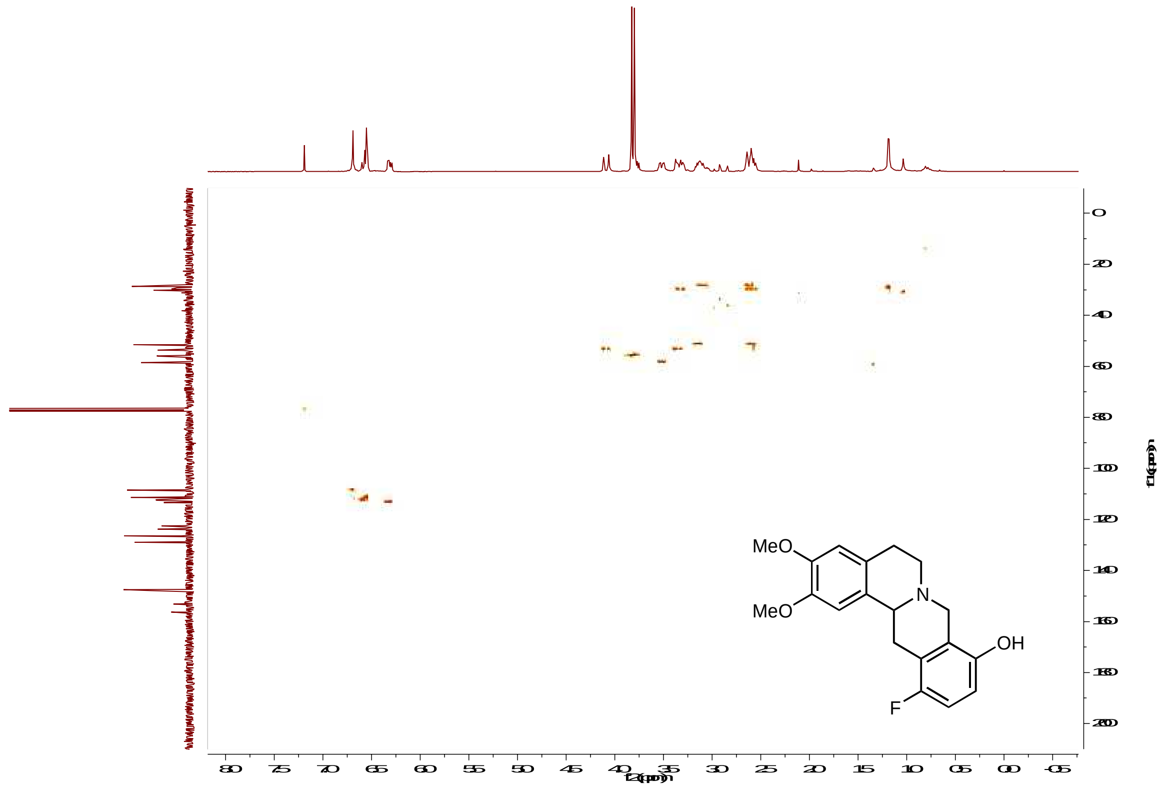

HRMS results

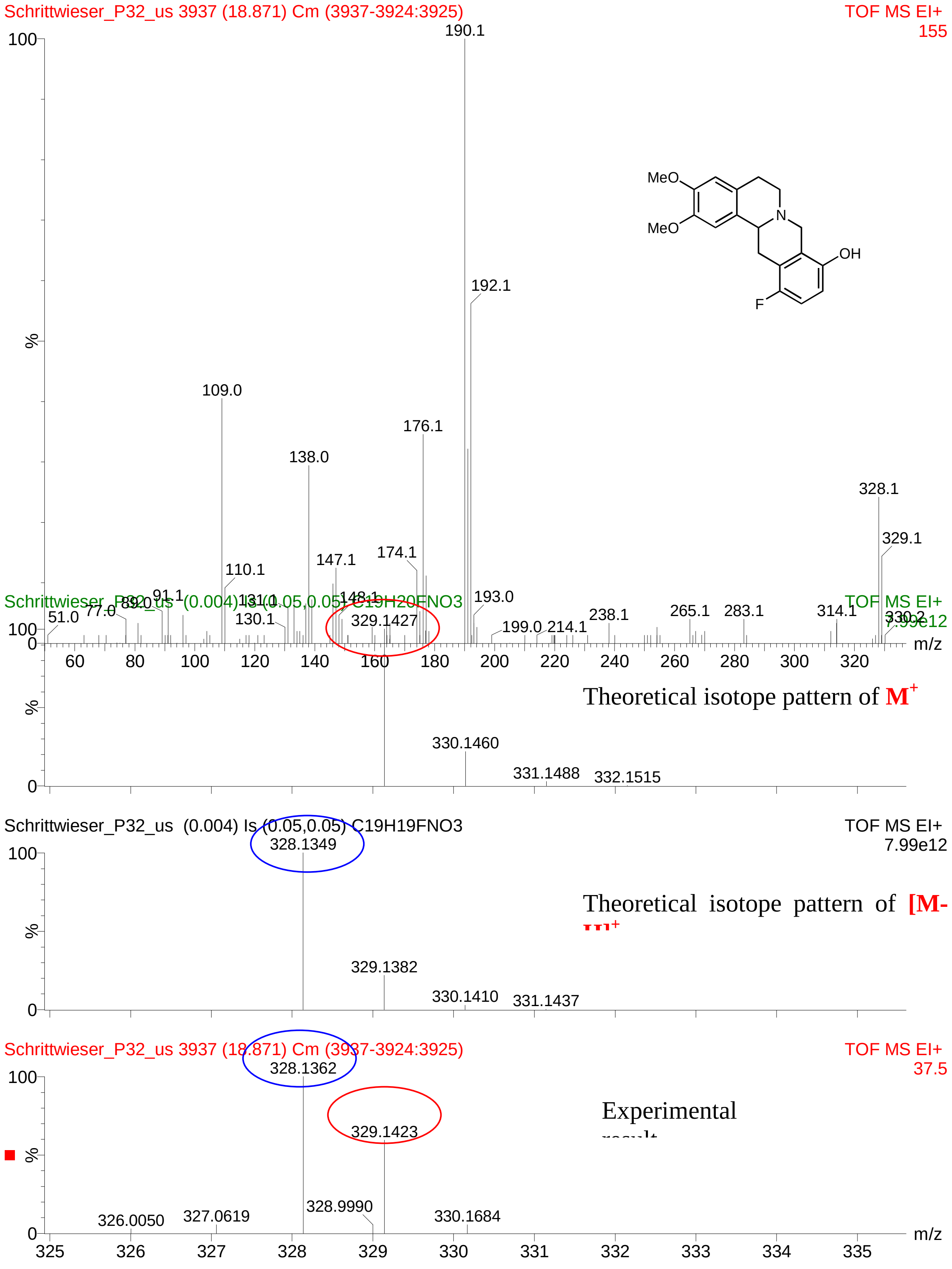

(*R*)-**1n**:

Provided Material:

(*R*)-**1n**
^{1}H-NMR spectrum, ^{13}C-NMR spectrum, COSY spectrum, HSQC spectrum, HRMS results

^{1}H-NMR

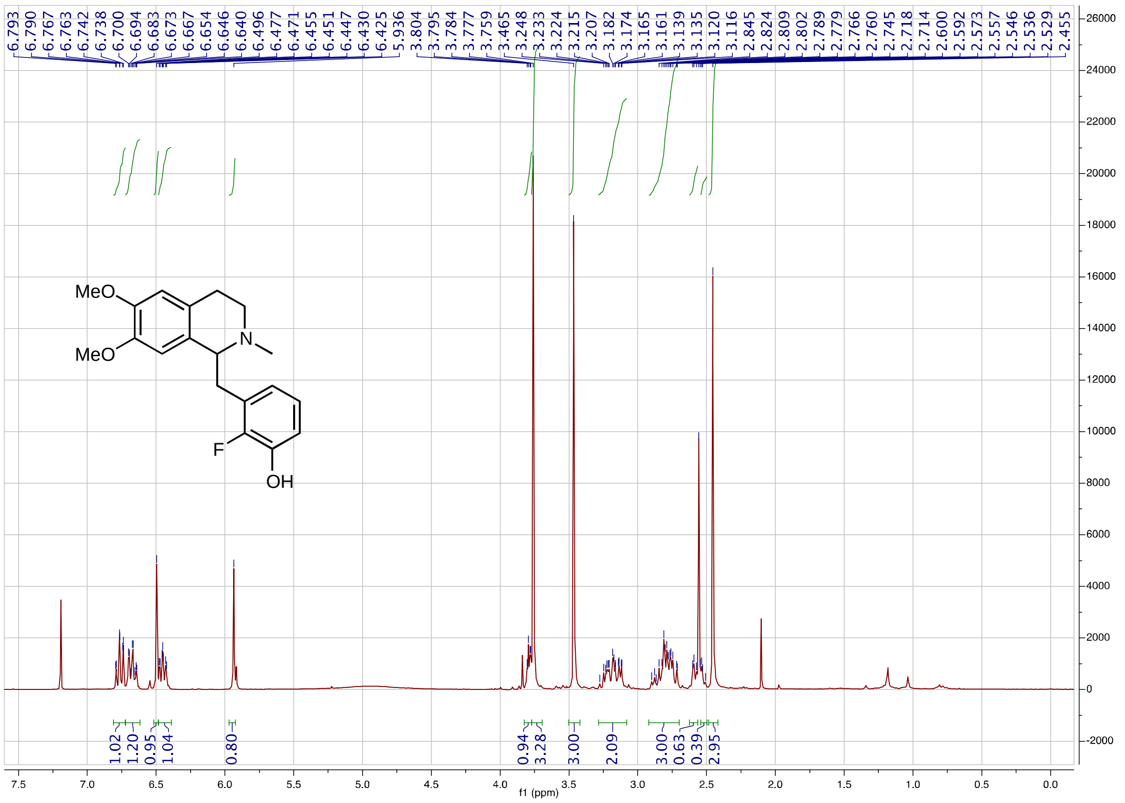

^{13}C-NMR

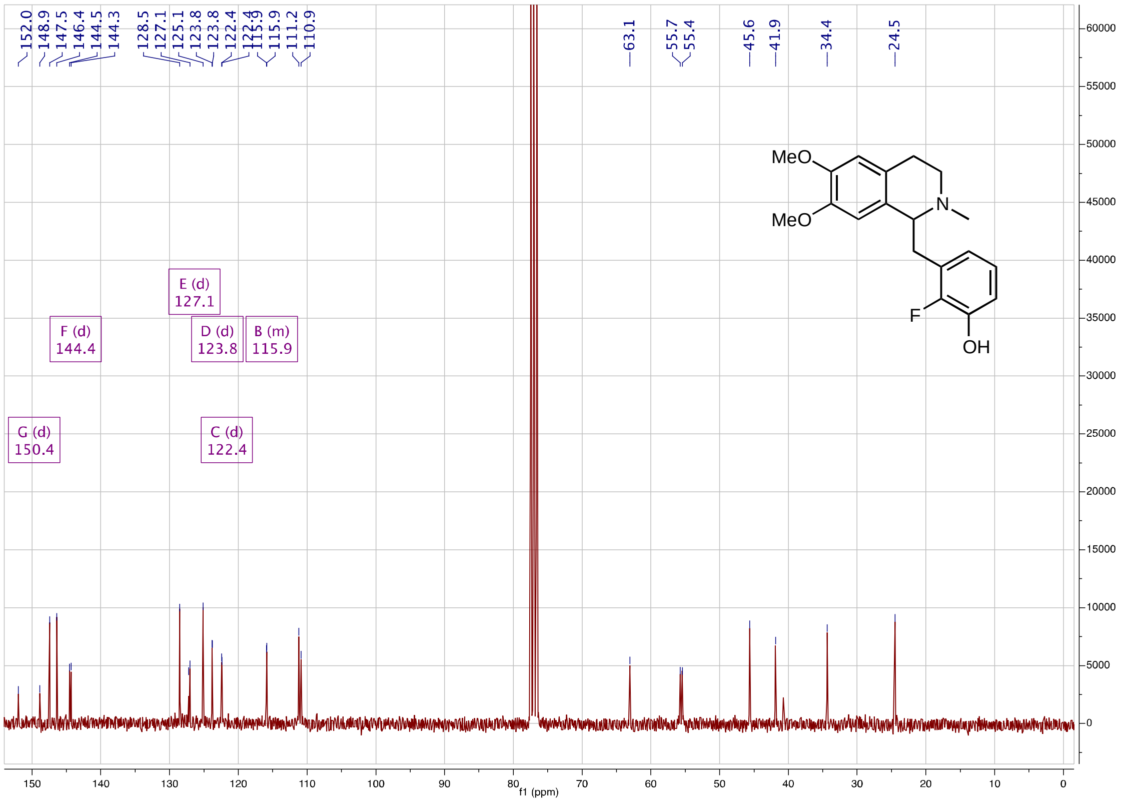

COSY spectrum

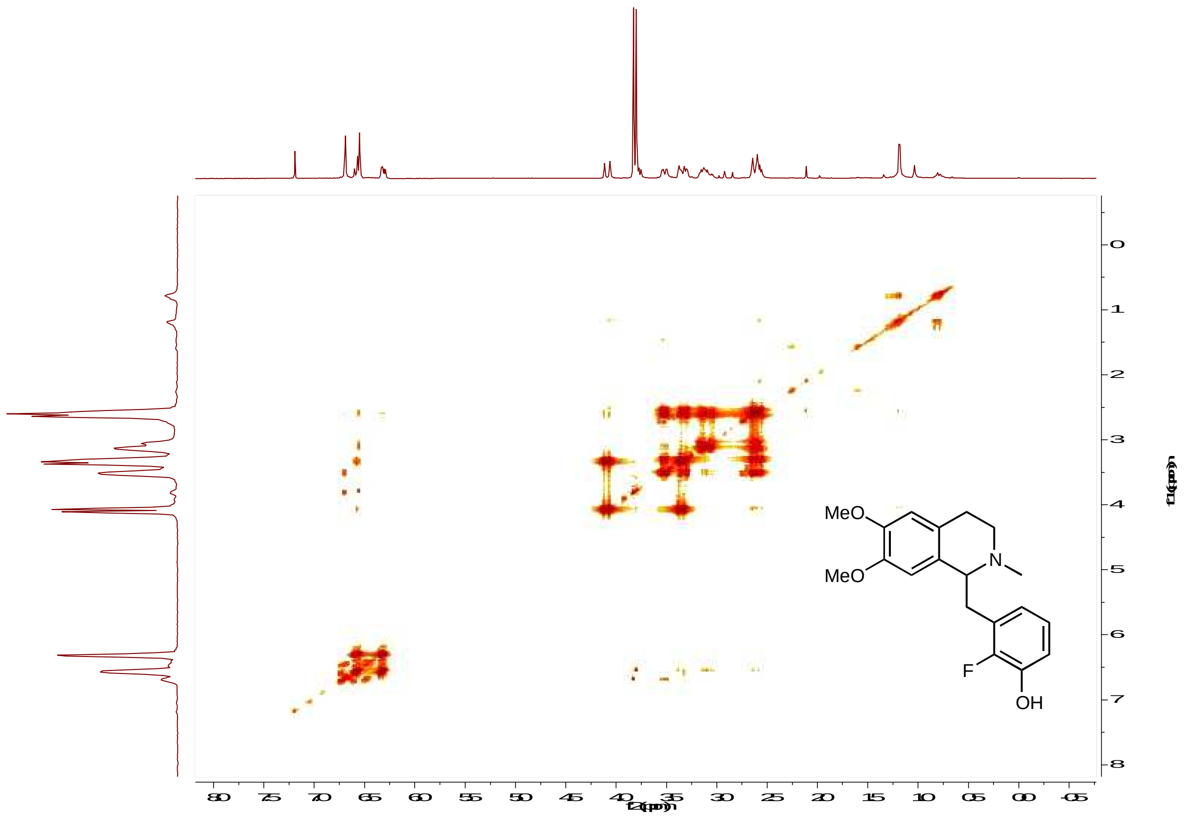

HSQC spectrum

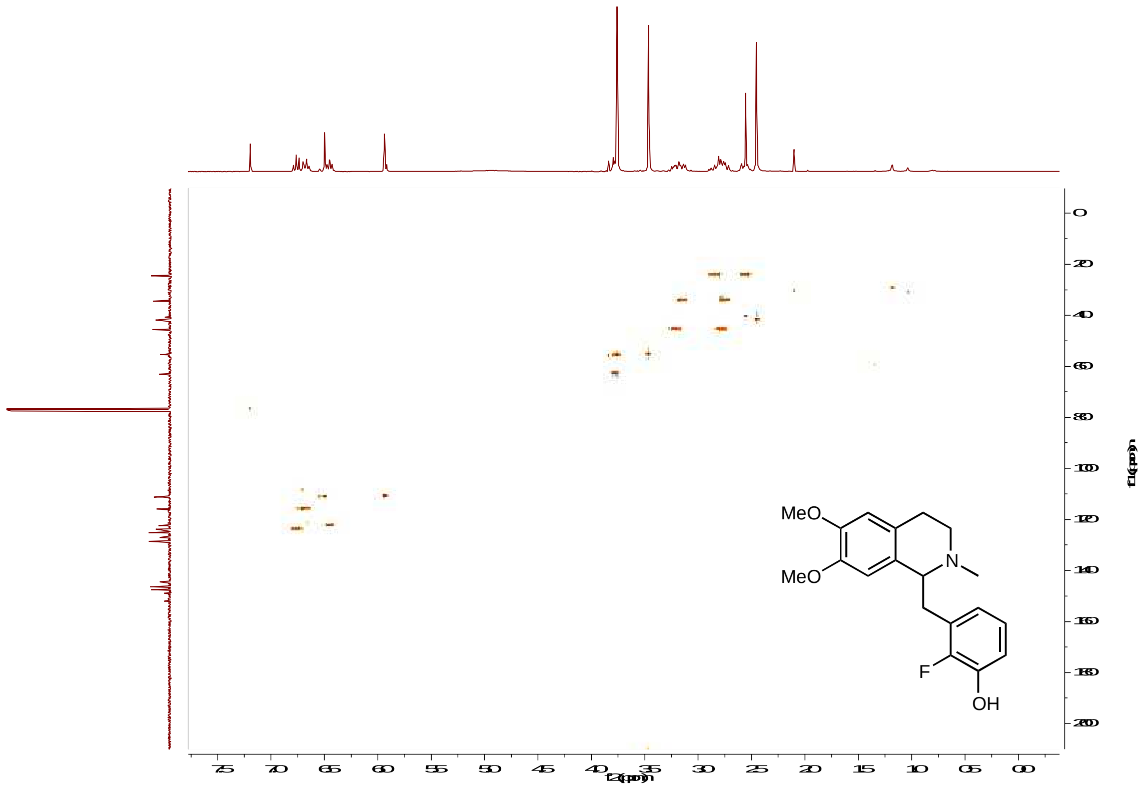

HRMS results

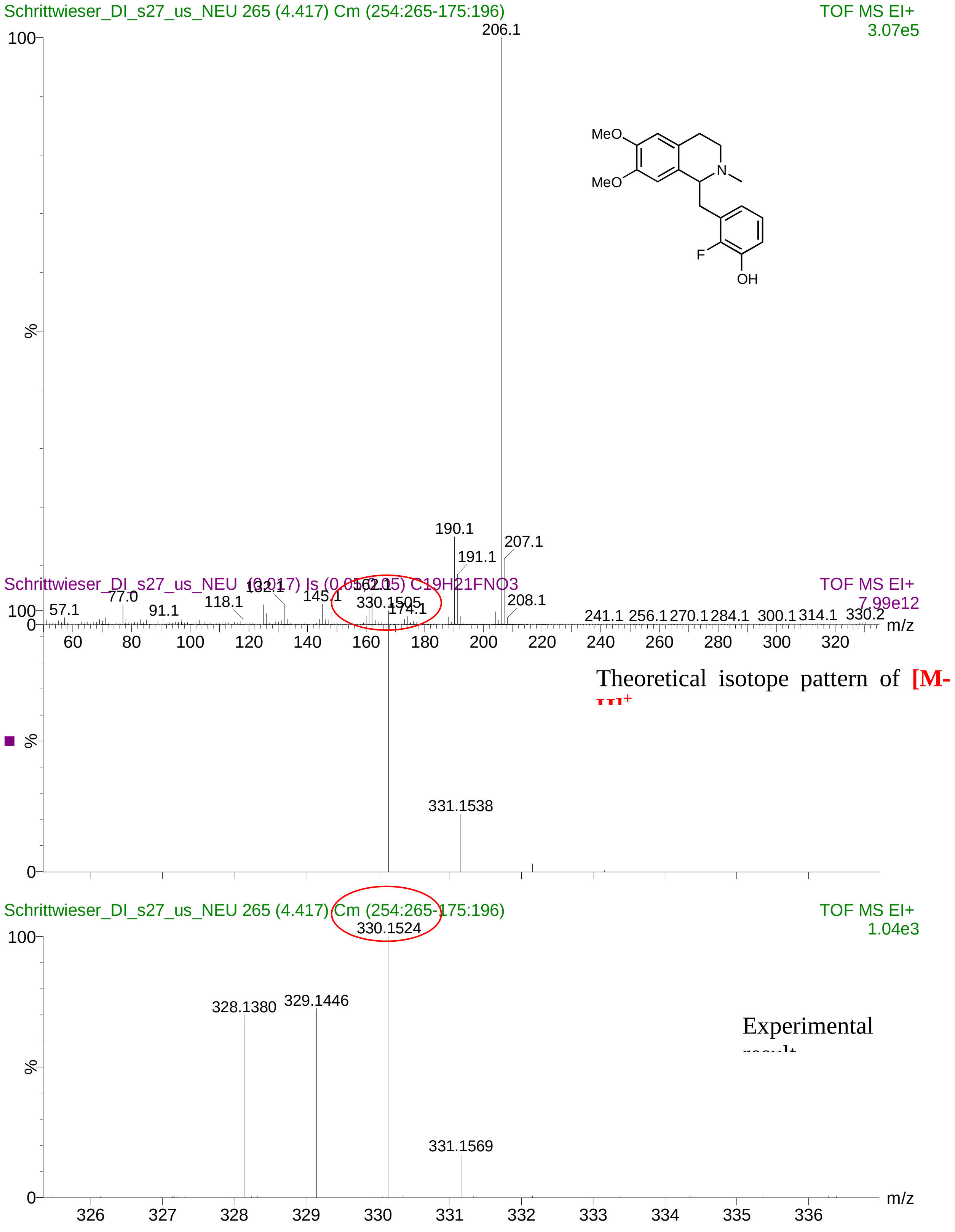

(*S*)-**3n**:

Provided Material:

(*S*)-**3n**
^{1}H-NMR spectrum, ^{13}C-NMR spectrum, COSY spectrum, HSQC spectrum, HRMS results

^{1}H-NMR

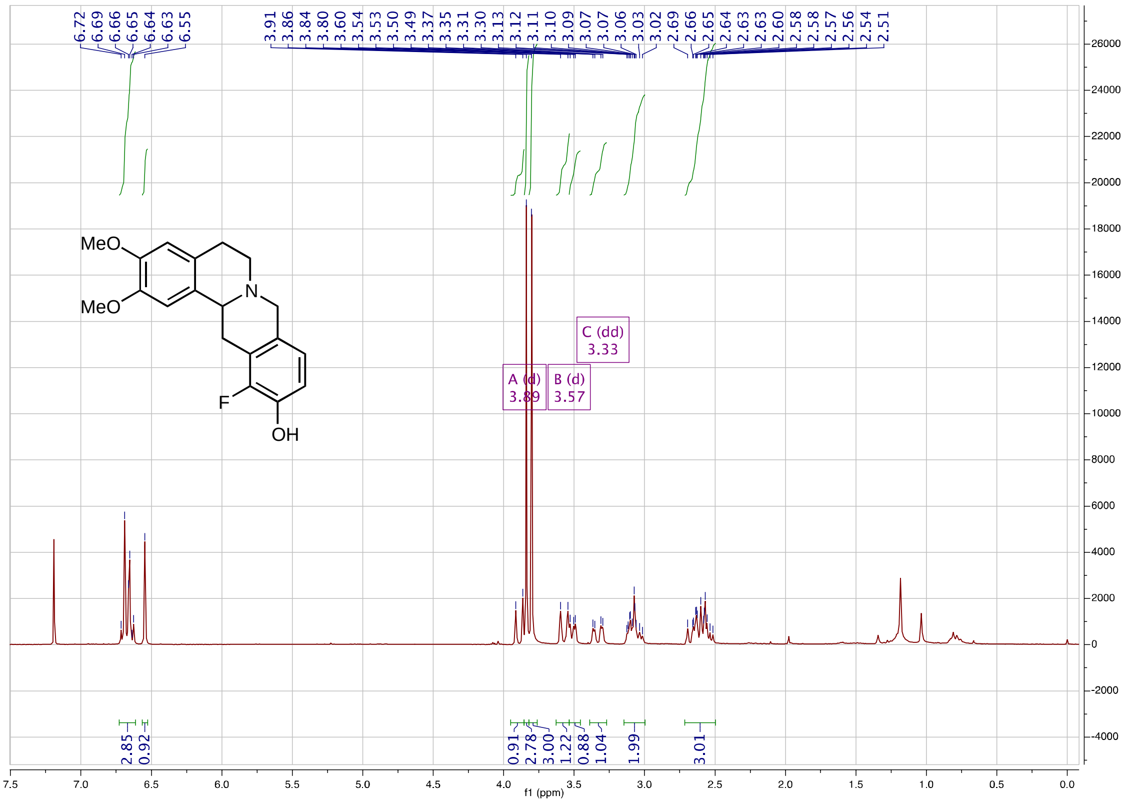

^{13}C-NMR

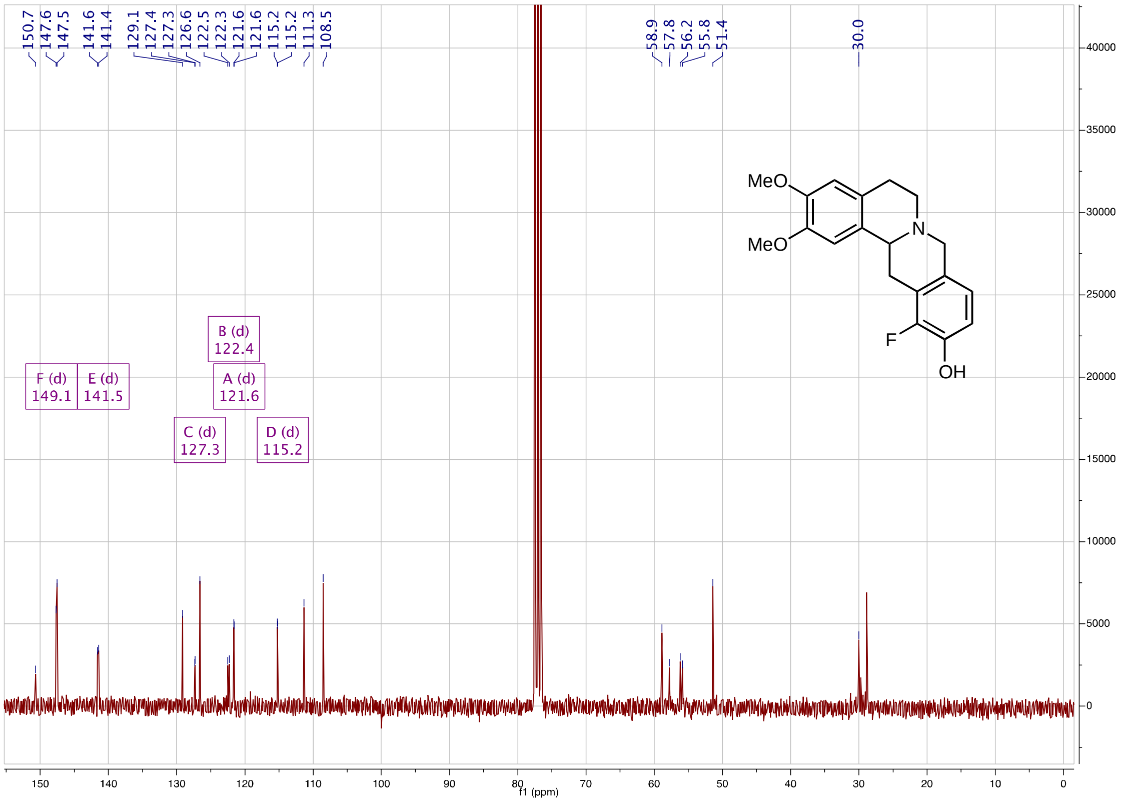

COSY spectrum

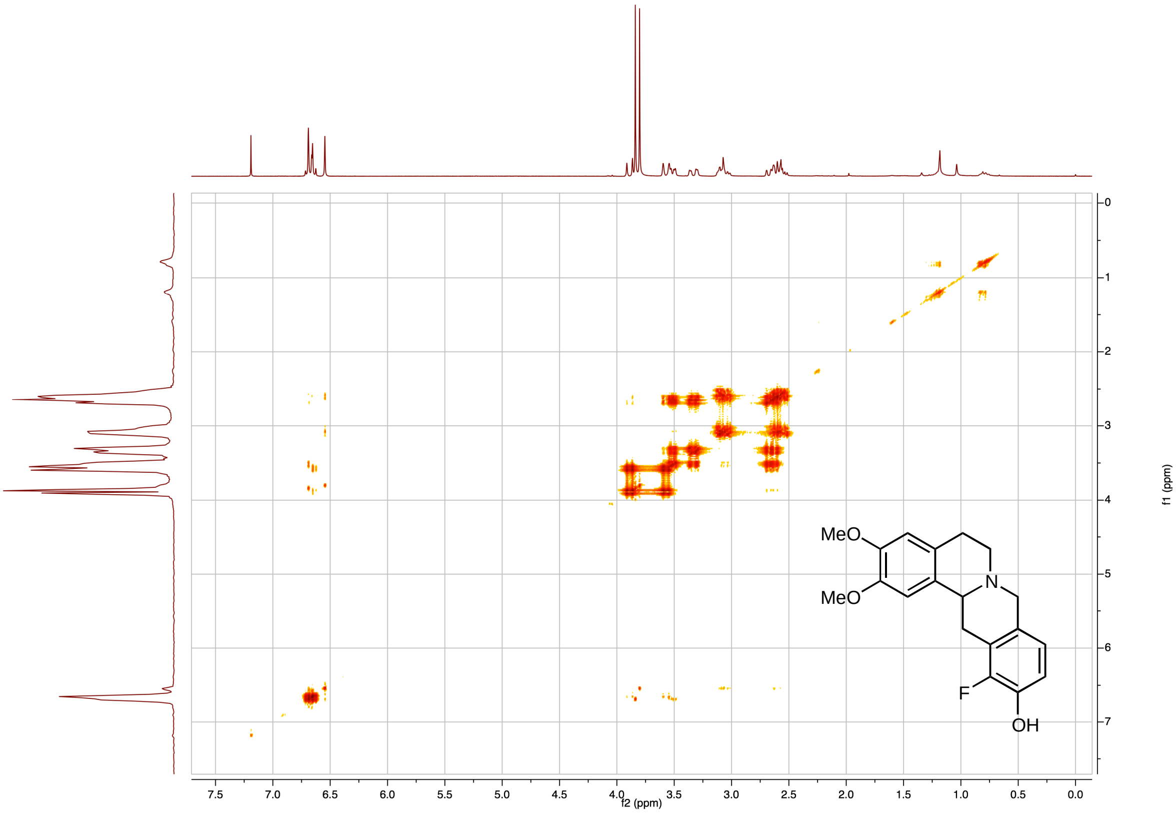

HSQC spectrum

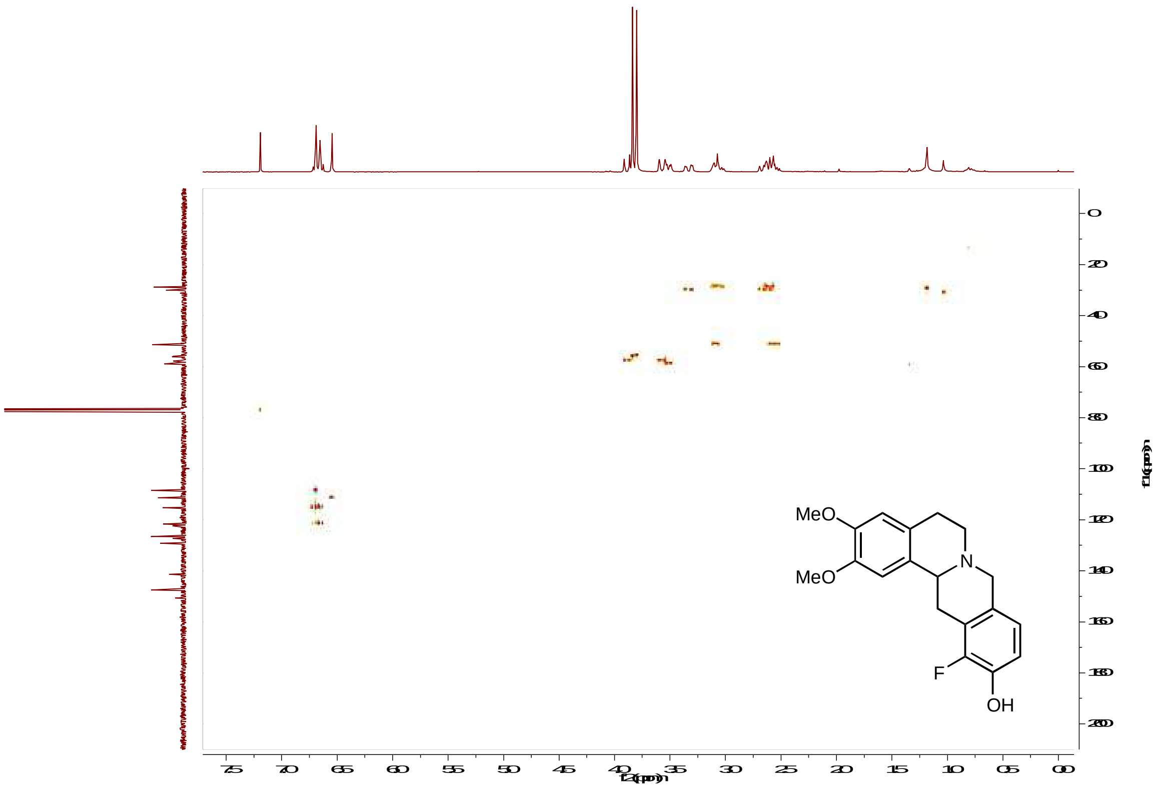

HRMS results

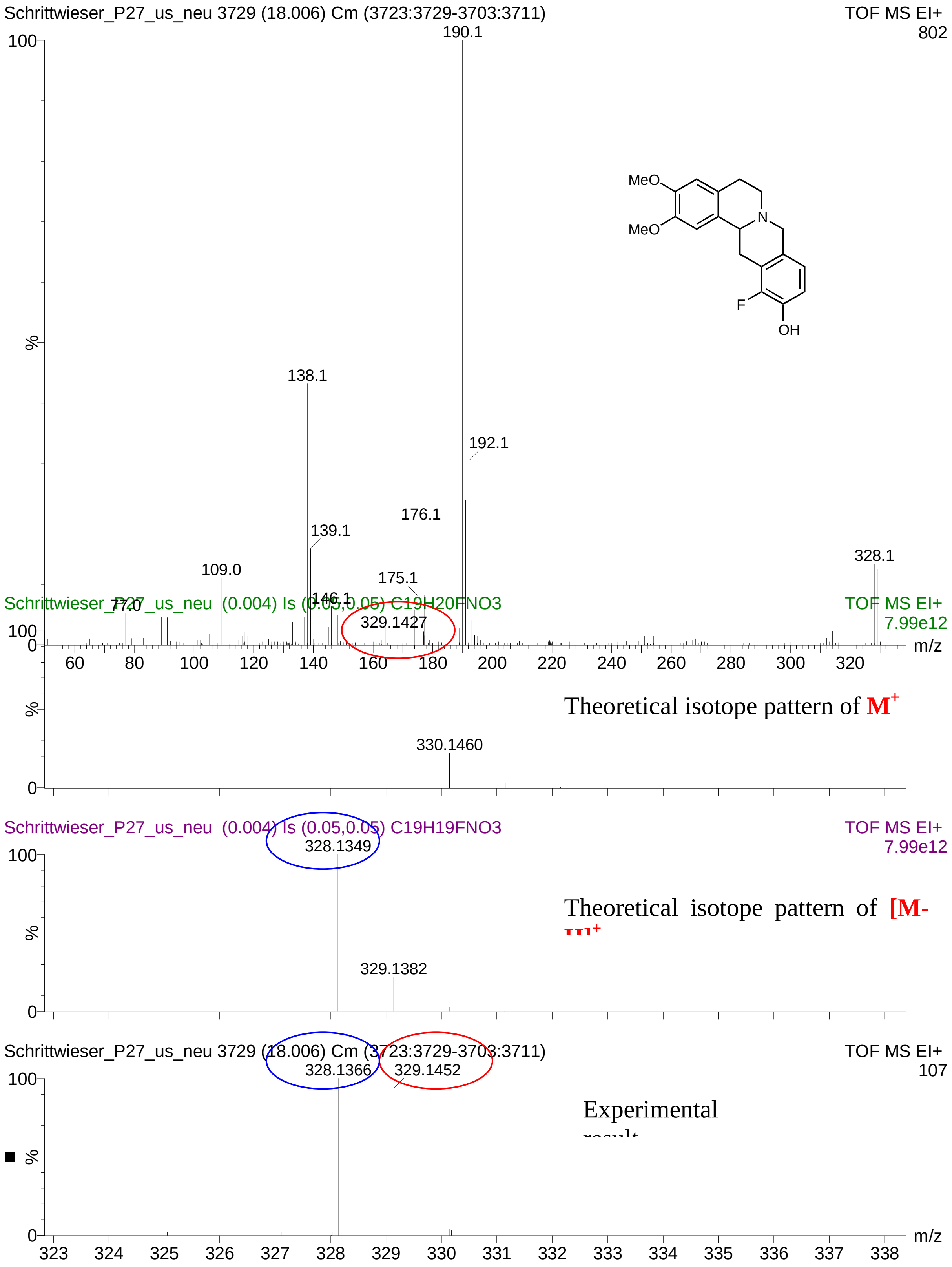

(*R*)-**1o**:

Provided Material:

(*R*)-**1o**
^{1}H-NMR spectrum, ^{13}C-NMR spectrum, COSY spectrum, HSQC spectrum, HRMS results

^{1}H-NMR

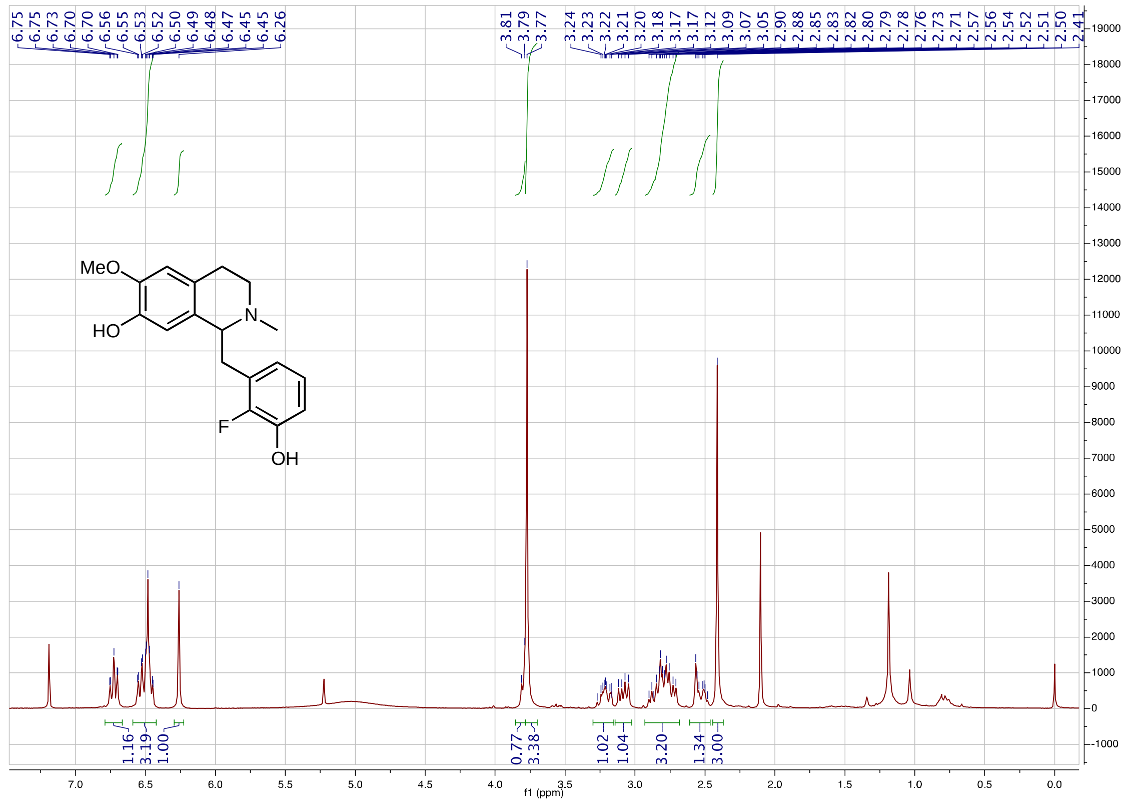

^{13}C-NMR

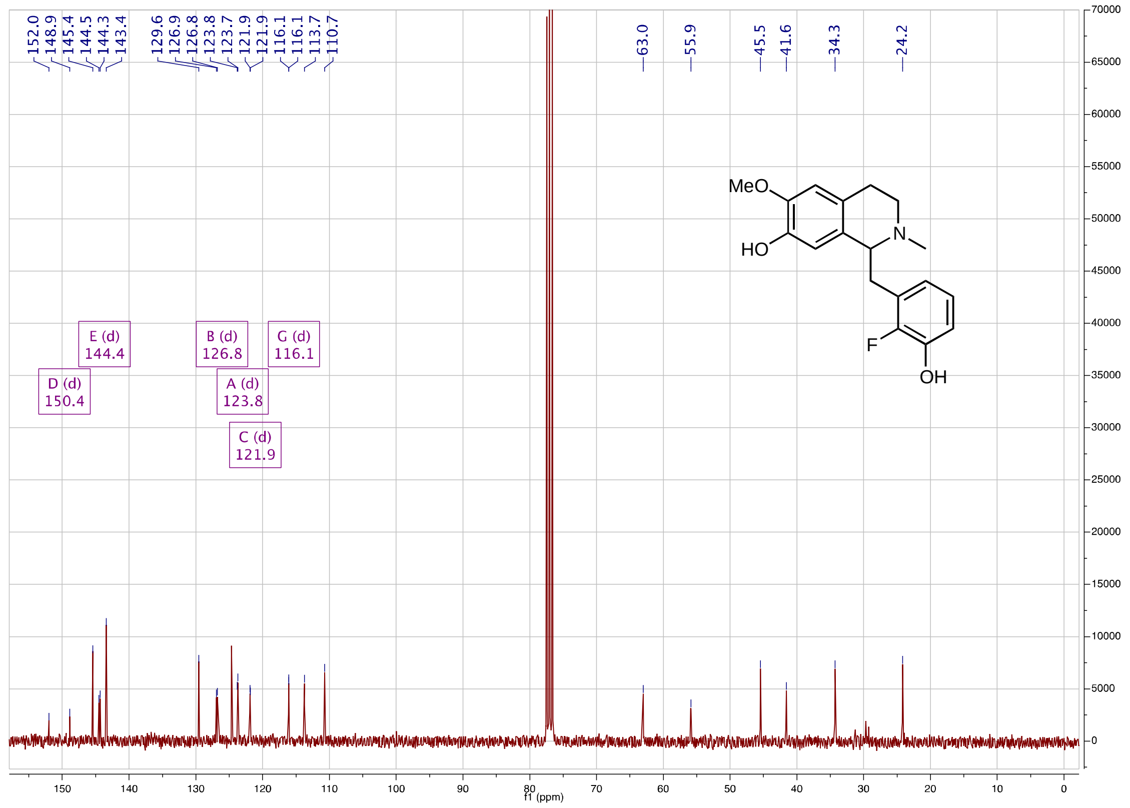

COSY spectrum

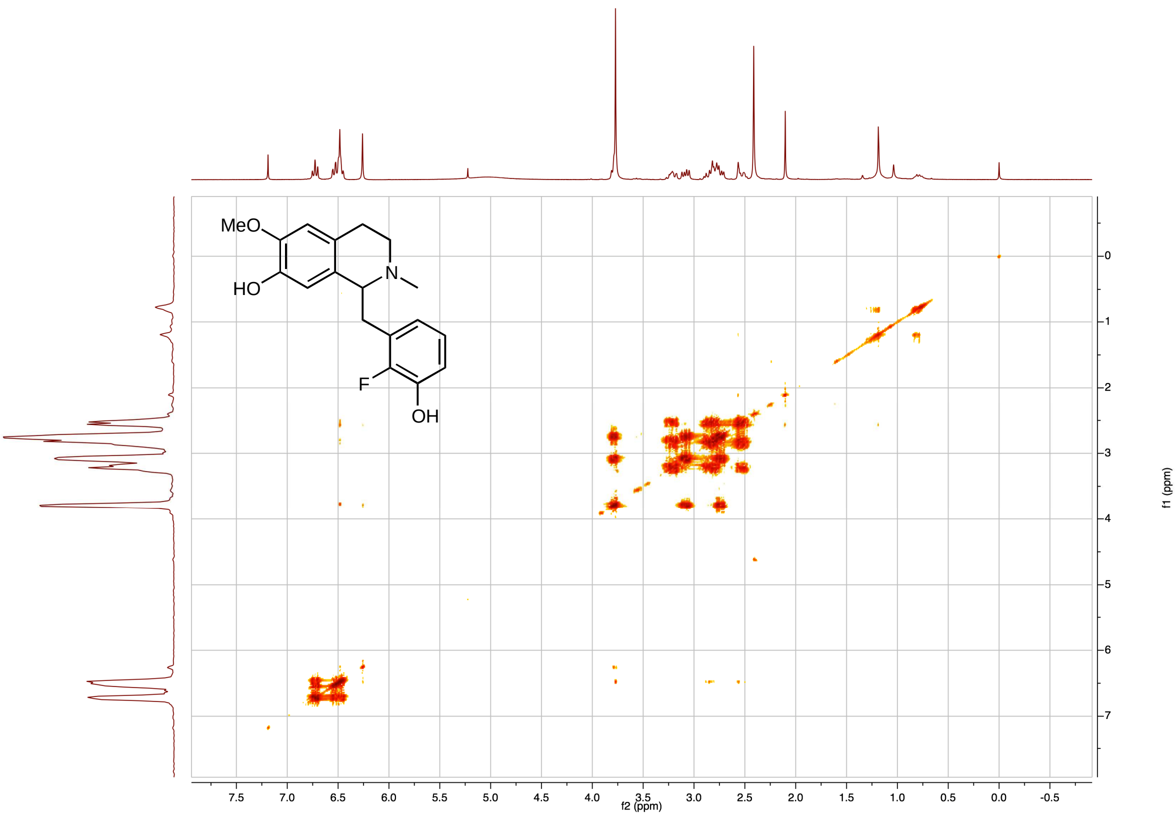

HSQC spectrum

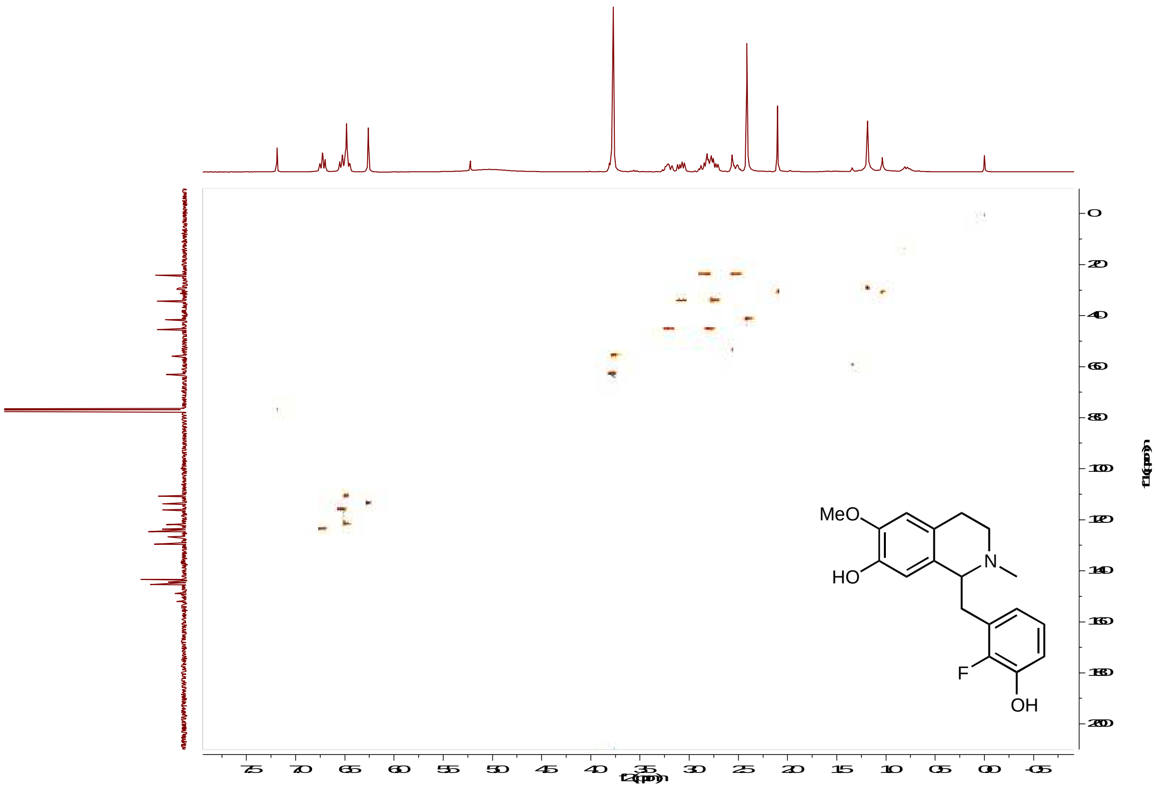

HRMS results

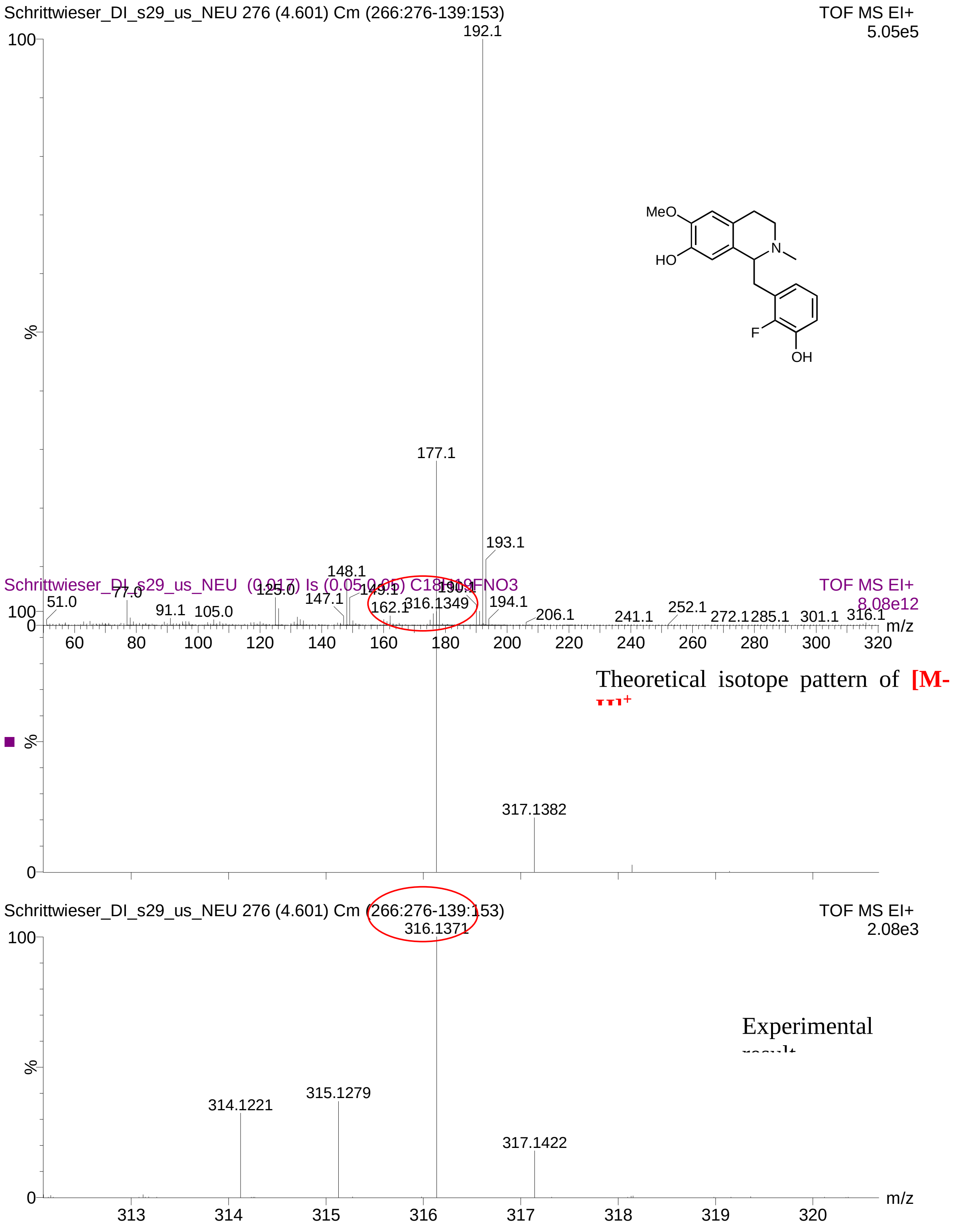

(*S*)-**3o**:

Provided Material:

(*S*)-**3o**
^{1}H-NMR spectrum, ^{13}C-NMR spectrum, COSY spectrum, HSQC spectrum, HRMS results

^{1}H-NMR

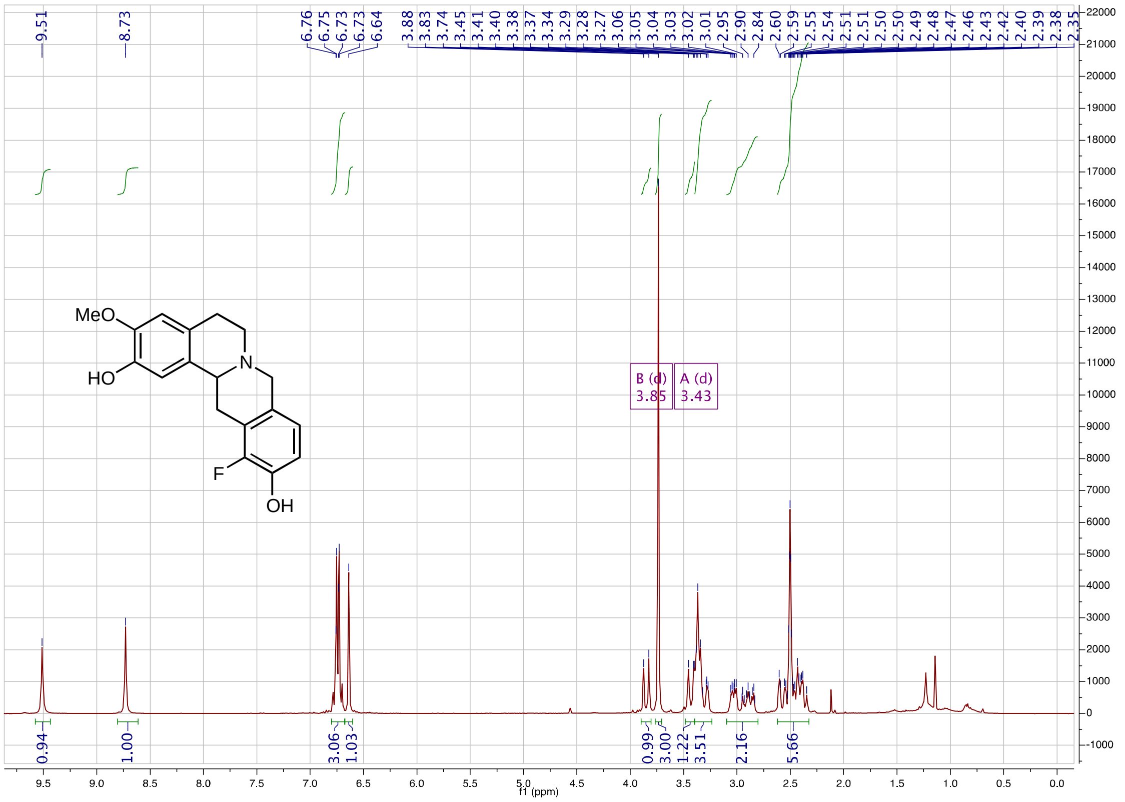

^{13}C-NMR

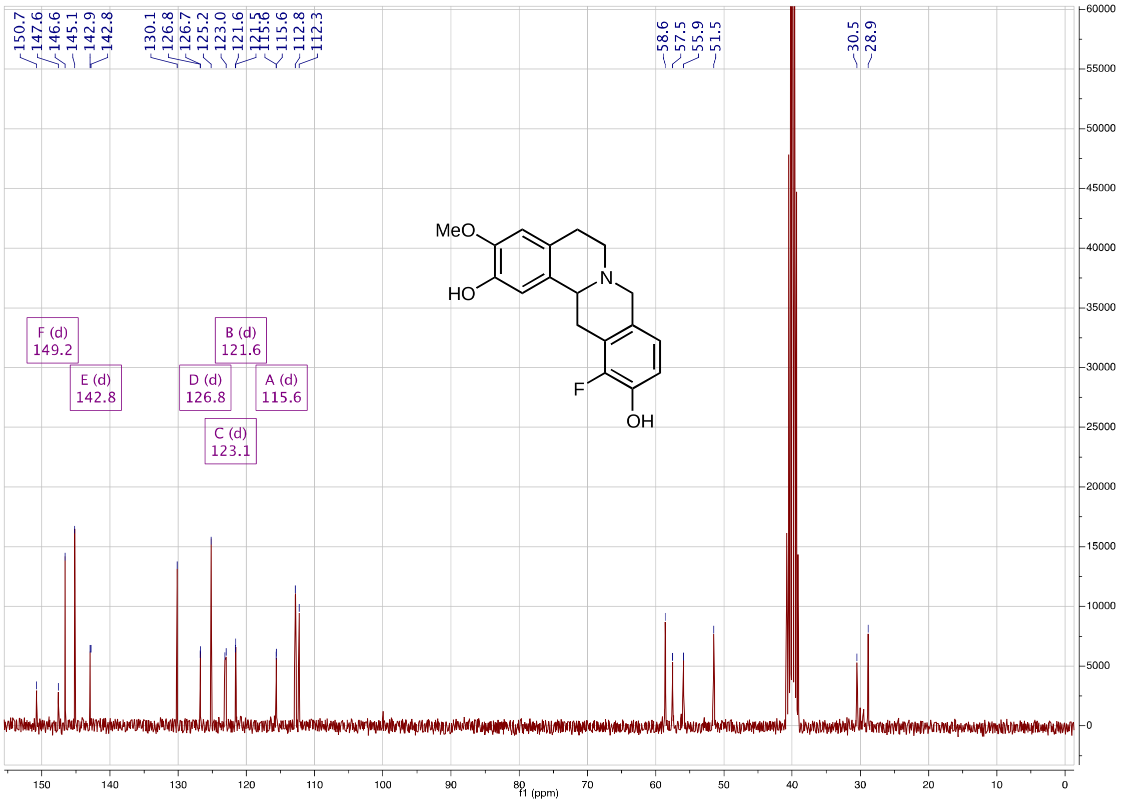

COSY spectrum

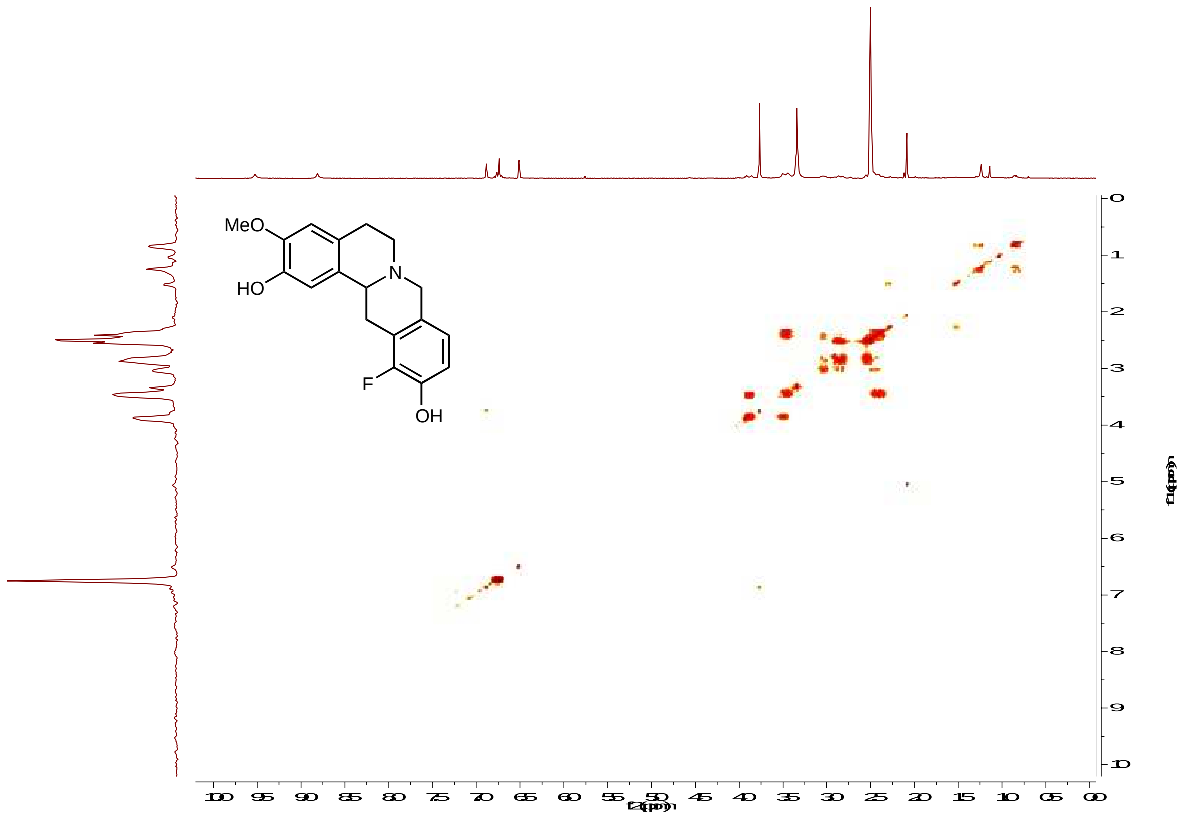

HSQC spectrum

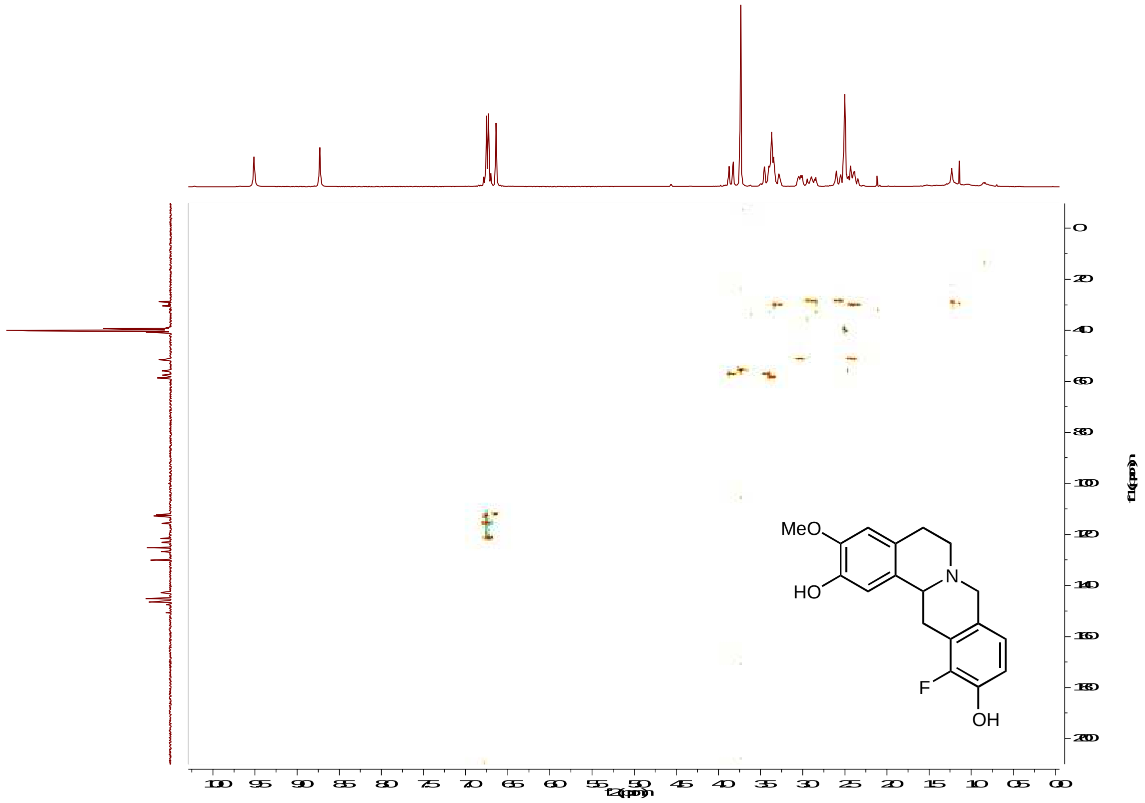

HRMS results

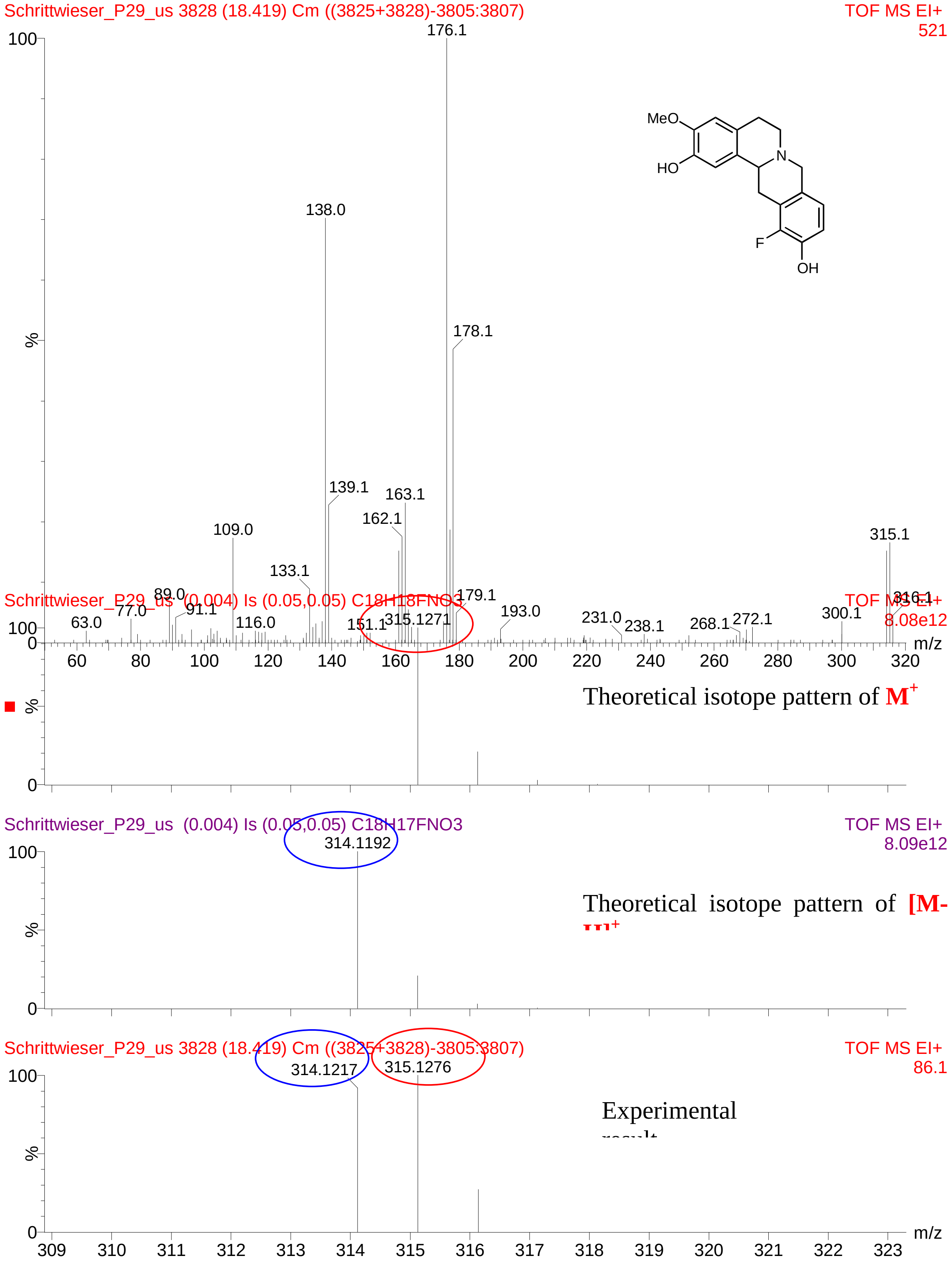

(*R*)-**1p**:

Provided Material:

(*R*)-**1p**
^{1}H-NMR spectrum, ^{13}C-NMR spectrum, COSY spectrum, HSQC spectrum, HRMS results

^{1}H-NMR

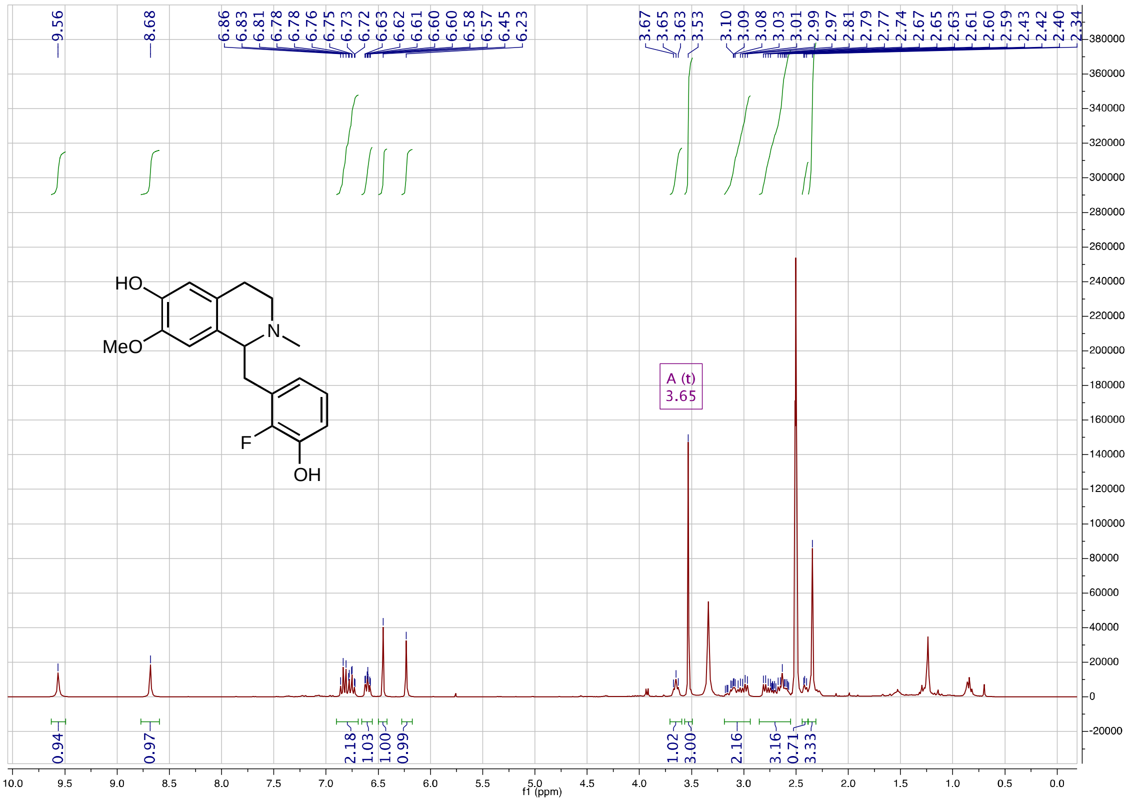

^{13}C-NMR

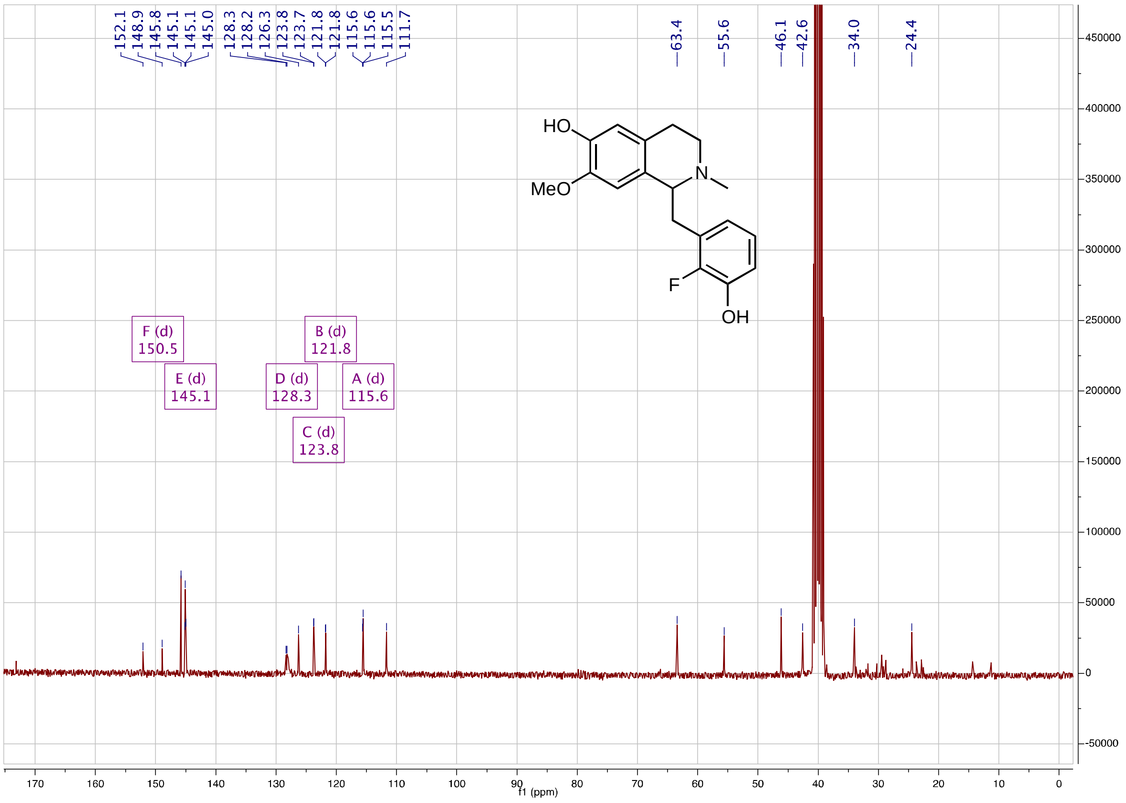

COSY spectrum

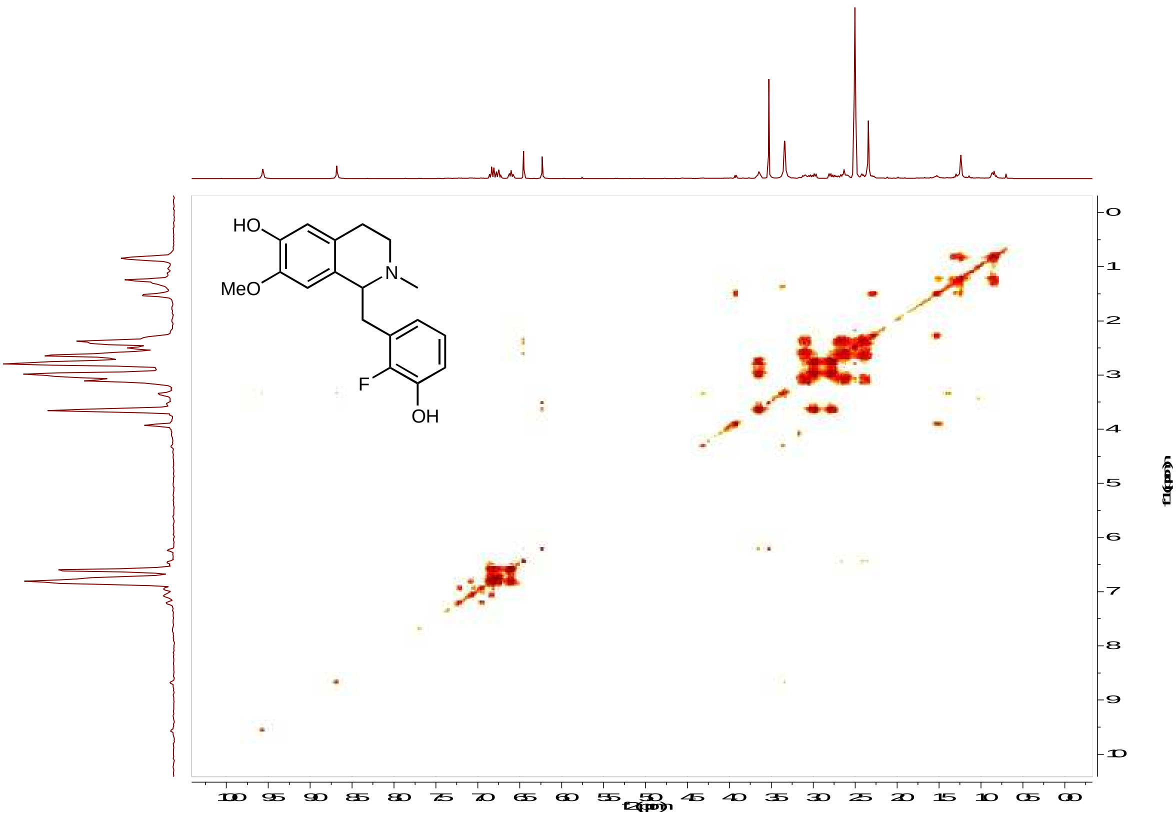

HSQC spectrum

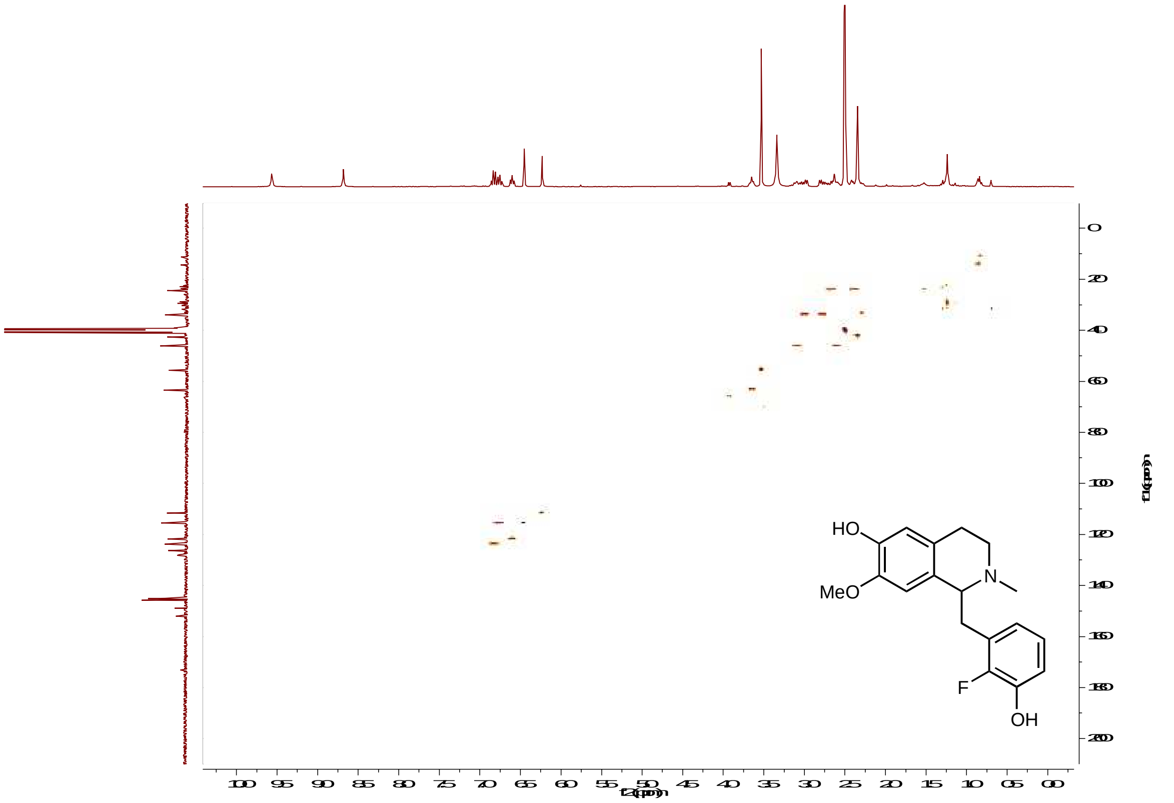

HRMS results

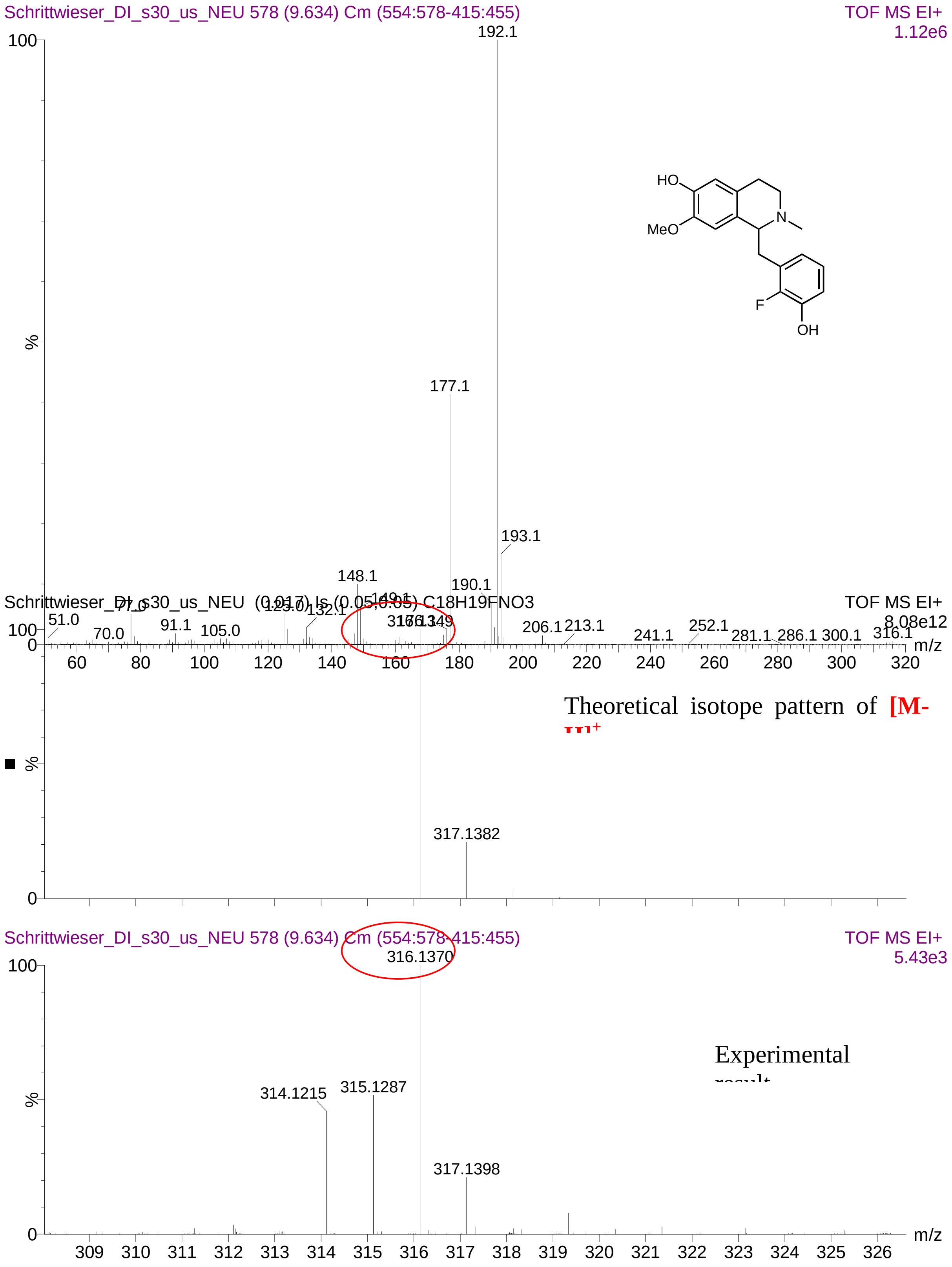

(*S*)-**3p**:

Provided Material:

(*S*)-**3p**
^{1}H-NMR spectrum, ^{13}C-NMR spectrum, COSY spectrum, HSQC spectrum

^{1}H-NMR

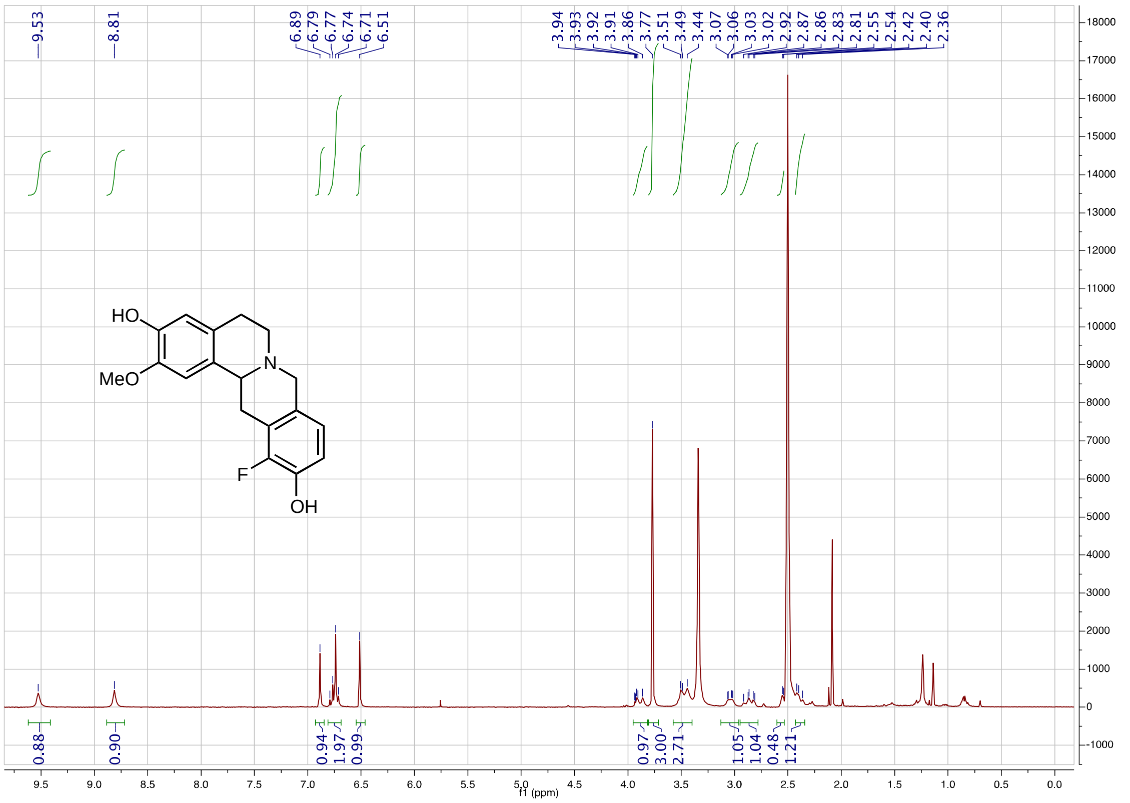

^{13}C-NMR

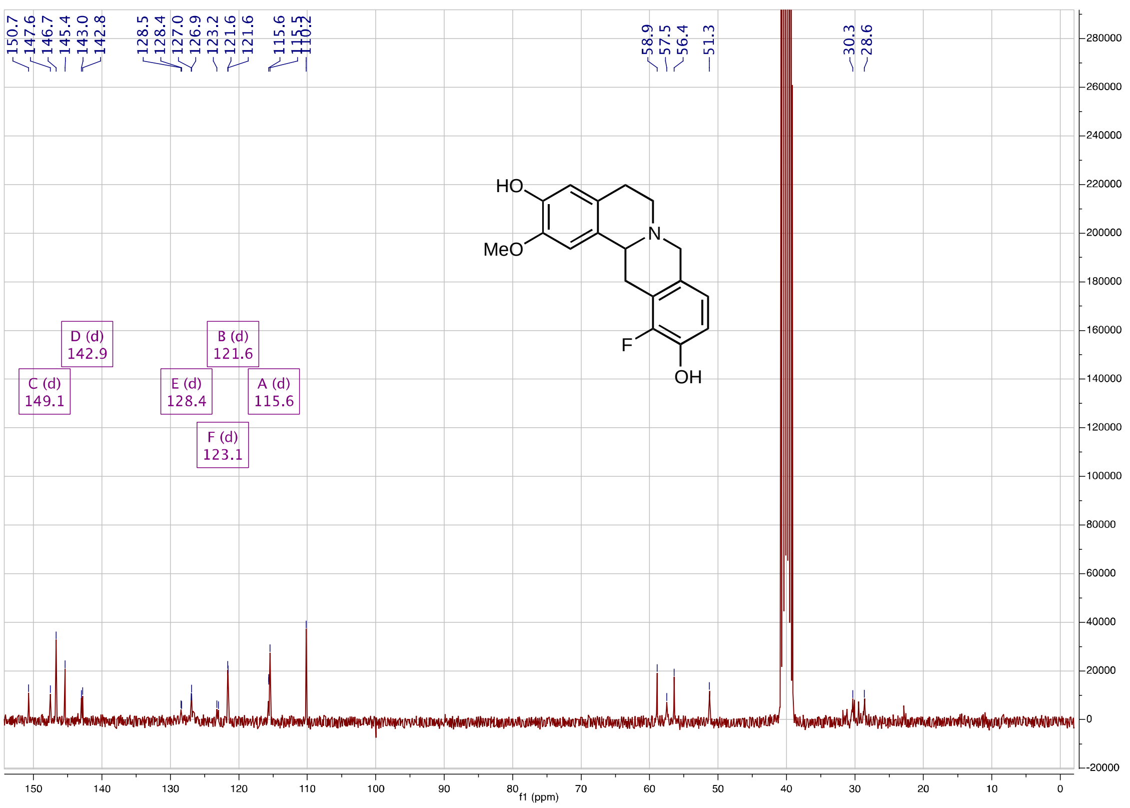

COSY spectrum

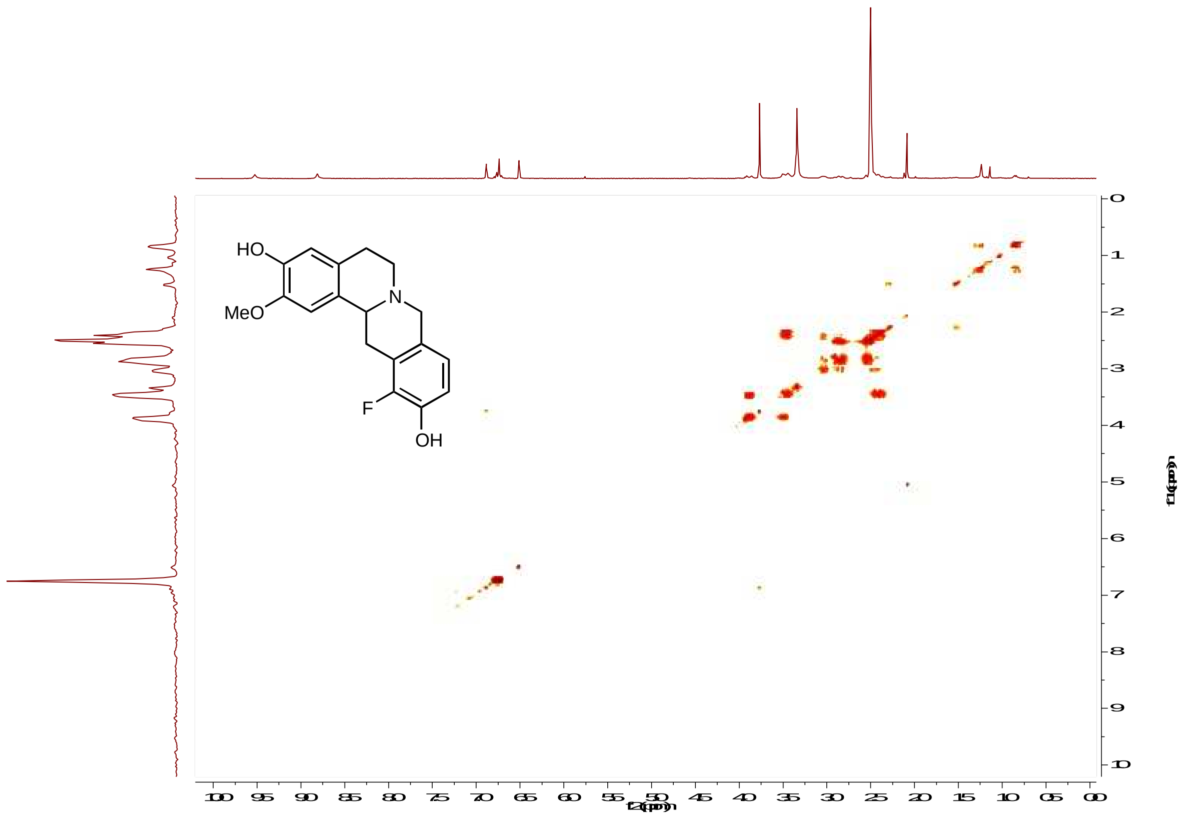

HSQC spectrum

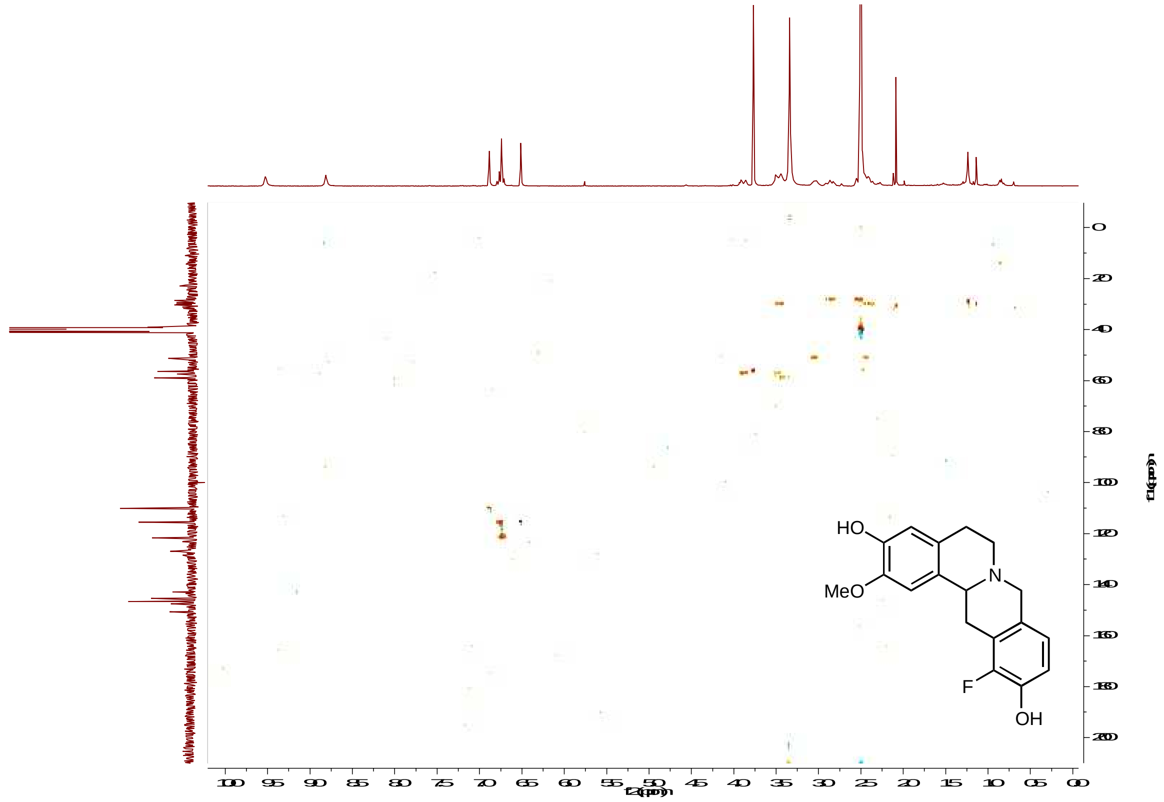

HRMS results

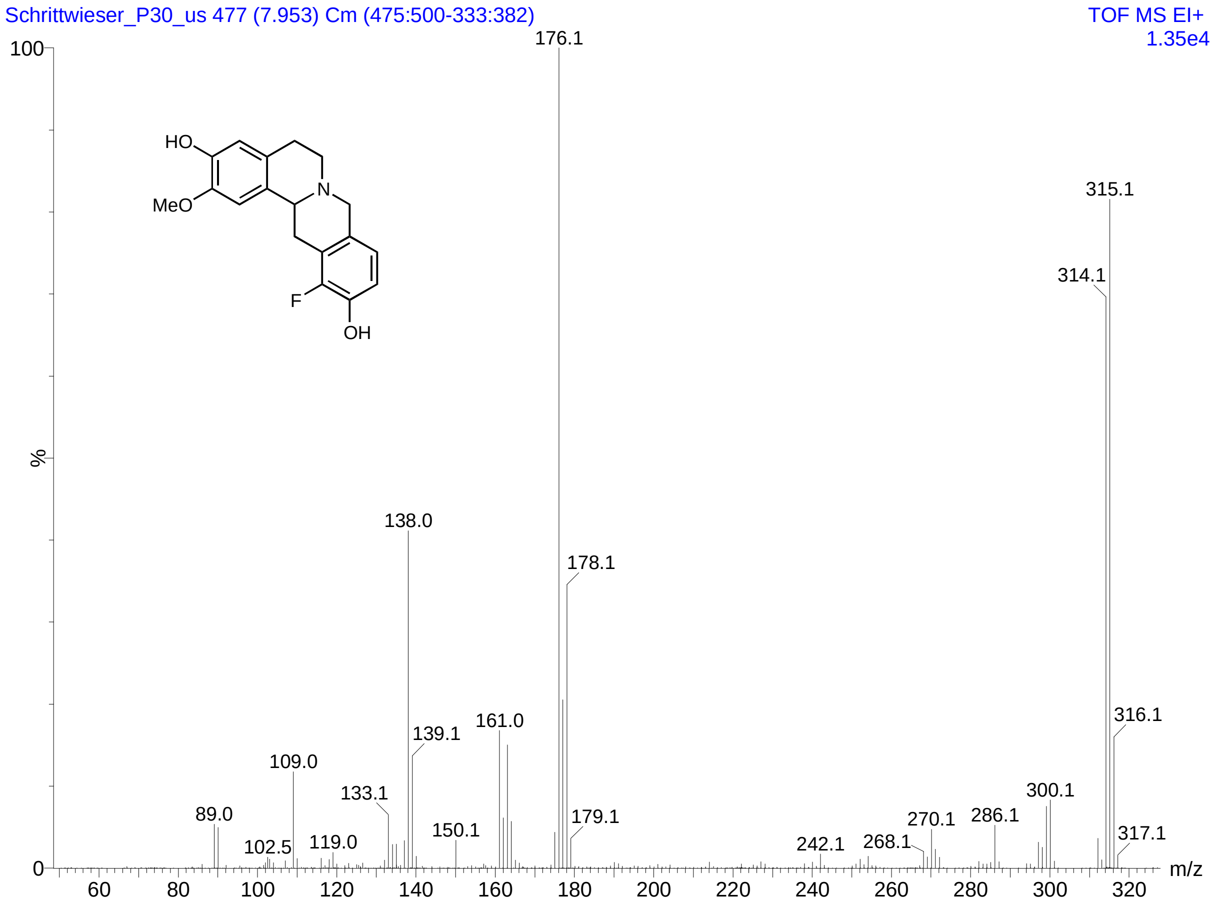

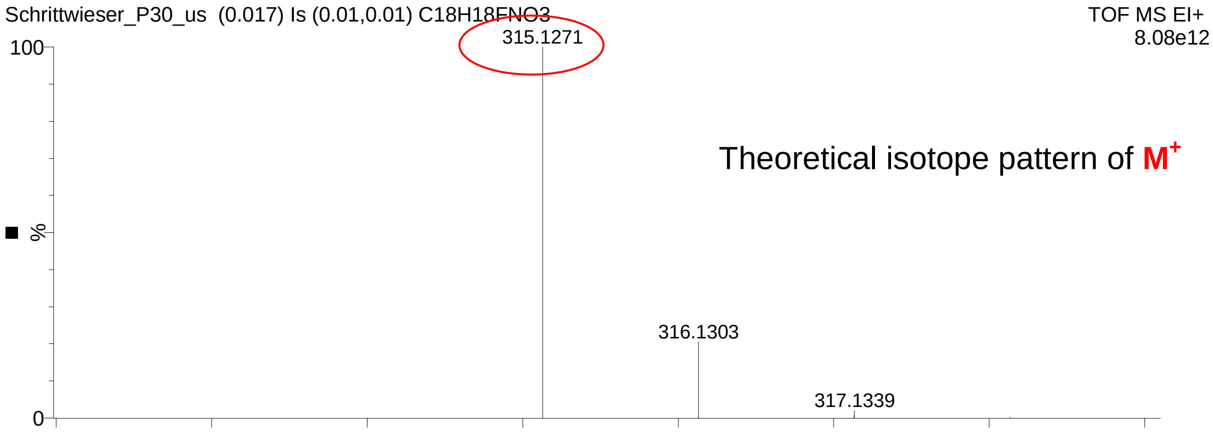

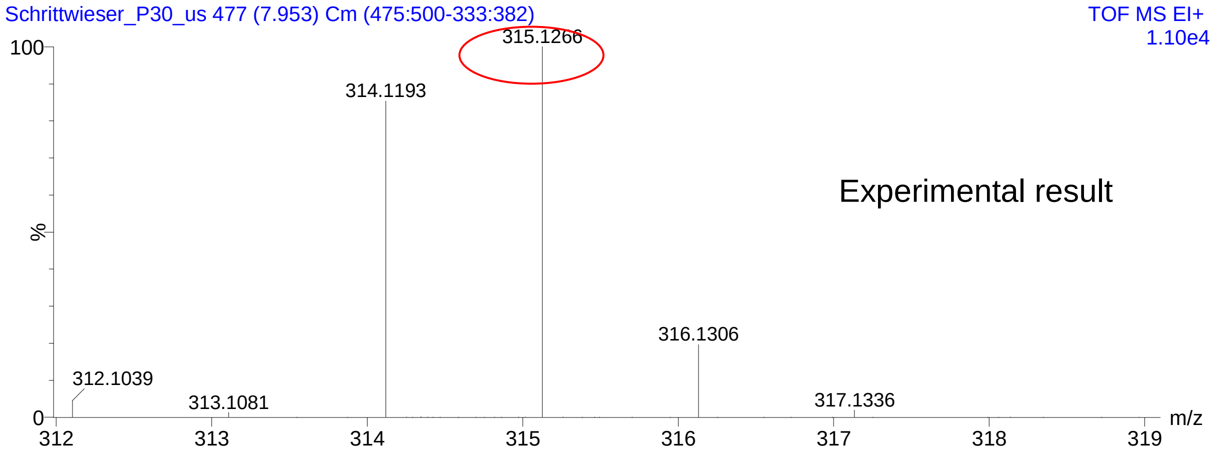

7. LITERATURE

[1] Schrittwieser, J. H.; Resch, V.; Sattler, J. H.; Lienhart, W.-D.; Durchschein, K.; Winkler, A.; Gruber, K.; Macheroux, P.; Kroutil, W. *Angew. Chem. Int. Ed.* **2011**, *50*, 1068–1071.

[2] Schrittwieser, J. H.; Resch, V.; Wallner, S.; Lienhart, W.-D.; Sattler, J. H.; Resch, J.; Macheroux, P.; Kroutil, W. *J. Org. Chem*. **2011**, *76*, 6703–6714

[3] Pouységu, L.; Avellan, A.-V.; Quideau, S. *J. Org. Chem.* **2002**, *67*, 3425–3436.

[4] Okano, K.; Tokuyama, H.; Fukuyama, T. *J. Am. Chem. Soc.* **2006**, *128*, 7136–7137.

[5] Bermejo, A.; Andreu, I.; Suvire, F.; Léonce, S.; Caignard, D. H.; Renard, P.; Pierré, A.; Enriz, R. D.; Cortes, D.; Cabedo, N. *J. Med. Chem.* **2002**, *45*, 5058–5068.

[6] Cabedo, N.; Protais, P.; Cassels, B. K.; Cortes, D. *J. Nat. Prod.* **1998**, *61*, 709–712.

[7] Pouységu, L.; Avellan, A.-V.; Quideau, *S. J. Org. Chem.* **2002**, 67, 3425-3426.

[8] Cafiero L. R.; Snowden, T. S. Org. Lett. **2008**, *10*, 3853–3856.

[9] Elzner, S.; Schmidt, D.; Schollmeyer, D.; Erkel, G.; Anke, T.; Kleinert, H.; Förstermann, U.; Kunz, H. *ChemMedChem* **2008**, *3*, 924–939.

[10] Lal, B.; Singh, P.; Bhaduri, A. P.; Kar, K. *Indian J. Chem.* **1975**, *13*, 898–903.
